# DNMT1 downregulation as well as its overexpression distinctly affect mostly overlapping genes implicated in schizophrenia, autism spectrum, epilepsy, and bipolar disorders

**DOI:** 10.3389/fnmol.2023.1275697

**Published:** 2023-12-06

**Authors:** Minali Singh, Sonal Saxena, Kommu Naga Mohan

**Affiliations:** ^1^Molecular Biology and Genetics Laboratory, Department of Biological Sciences, Birla Institute of Technology and Science, Pilani, Hyderabad, India; ^2^Centre for Human Disease Research, Birla Institute of Technology and Science, Pilani, Hyderabad, India

**Keywords:** *Dnmt1*, schizophrenia, autism spectrum disorder, bipolar disorder, epilepsy, gene dysregulation

## Abstract

Data on schizophrenia (SZ), epilepsy (EPD) and bipolar disorders (BPD) suggested an association of DNMT1 overexpression whereas certain variants of the gene were predicted to result in its increased expression in autism spectrum disorder (ASD). In addition, loss of DNMT1 in frontal cortex resulted in behavioral abnormalities in mice. Here we investigated the effects of increased as well as lack of DNMT1 expression using *Dnmt1^tet/tet^* neurons as a model for abnormal neurogenesis and 10,861 genes showing transcript level dysregulation in datasets from the four disorders. In case of overexpression, 3,211 (∼ 30%) genes were dysregulated, affecting pathways involved in neurogenesis, semaphorin signaling, ephrin receptor activity, etc. A disproportionately higher proportion of dysregulated genes were associated with epilepsy. When transcriptome data of *Dnmt1^tet/tet^* neurons treated with doxycycline that downregulated DNMT1 was used, 3,356 genes (∼31%) were dysregulated with a significant proportion involved in pathways similar to those in untreated cells. Both conditions resulted in ∼68% of dysregulated genes wherein a majority showed similar patterns of transcript level changes. Among the genes with transcripts returning to normal levels, ribosome assembly/biogenesis was most significant whereas in absence of DNMT1, a new set of 903 genes became dysregulated and are involved in similar pathways as mentioned above. These findings provide support for overexpression of DNMT1 as well as its downregulation as risk factor for the four disorders and that its levels within a tight range are essential for normal neurodevelopment/mental health.

## Introduction

Mental health conditions like schizophrenia (SZ), epilepsy (EPD), autism spectrum (ASD) and bipolar (BPD) disorders together constitute ∼4% of the world population (Sawa and Snyder, 2002; Marshall et al., 2017; Inal et al., 2022; Lee et al., 2022; Zeidan et al., 2022). There is no single cause, but multiple etiologies ranging from physical, chemical, genetic, epigenetic, and environmental factors were suggested (Hilty et al., 2006; Shorvon, 2011; Park et al., 2016; Rantala et al., 2022). Of these, increased familial risk with an affected first-degree relative suggests the contribution of genetic factors whereas lack of high concordance rates among monozygotic twins (ASD: 36–95%, BPD: 72%, EPD: 65%, SZ: ∼50%) suggest non-genetic factors (Sharma, 1986; Rehm et al., 2002; Rosenberg et al., 2009; Gejman et al., 2010). Among these, epigenetic processes have received wide attention and resulted in multiple studies identifying abnormalities associated with the epigenetic modifications in candidate genes and genome-scale studies involving the patients and controls (Eshraghi et al., 2018; Kobow and Blümcke, 2018; Smigielski et al., 2020; Legrand et al., 2021).

DNA methylation and several post-translational modifications of the N-terminal tails of the core histones constitute the main epigenetic modifications of the mammalian genomes (Kim et al., 2009). Of these, DNA methylation involves the addition of a methyl-group to the 5th carbon of cytosines (Singal and Ginder, 1999). This process, referred to as cytosine methylation, was the first to be discovered and has been extensively studied in the context of its establishment, maintenance, erasure, and influence on gene expression (Meng et al., 2015). Most of the cytosine methylation occurs in the CpG dinucleotides and in many cases (Deaton and Bird, 2011), CpG methylation at promoters is associated with gene repression (Blattler and Farnham, 2013). Methylation patterns in a cell’s DNA can be established *de novo* or inherited from the parental cell through maintenance methylation. Accordingly, DNA methyltransferases (DNMTs) 3A, 3B and 3L establish new methylation marks and are the *de novo* methyltransferases (Saravanaraman et al., 2020) that are mainly expressed in the gametogenesis stages and in stem cells post-fertilization (Goll and Bestor, 2005; Messerschmidt et al., 2014). On the other hand, DNMT1 is the only maintenance methyltransferase (Chaillet et al., 1991) and is expressed throughout life.

Abnormal DNA methylation patterns associated with all the four neurodevelopmental/neurological disorders mentioned above have been identified in case-control studies as well as in discordant monozygotic twins (Dempster et al., 2011; Liu et al., 2014; Castellani et al., 2015; Andrews et al., 2018; Kobow et al., 2019; Liang et al., 2019; Mohandas et al., 2019). In some cases, these abnormalities were also associated with dysregulated expression of some candidate genes (Sugawara et al., 2018; Tao et al., 2021). The presence of aberrant DNA methylation patterns also indicated dysregulation of the DNA methylation machinery and prompted investigations on the levels of DNMTs in the patients. Of these, increased DNMT1 levels in the GABAergic interneurons of SZ and BPD patients with psychosis was first to be described (Veldic et al., 2005). In these cases, increased DNMT1 binding and repression of *GAD*_67_, *RELN*, and *BDNF* promoters was reported (Dong et al., 2015). Subsequently, increased DNMT1 levels were also reported in EPD (Zhu et al., 2012). A recent study on ASD patients identified two intronic SNPs that are likely to result in increased expression of DNMT1 (Alex et al., 2019). Although the decreased levels identified in ASD patients is nominally significant, there was increased binding of DNMT1 and repression of *GAD*_67_ and *RELN* promoters (Zhubi et al., 2017). Together, these results suggest a general mechanism by which altered DNMT1 levels can influence the disease-associated genes in SZ, BPD, EPD and ASD. Despite these data, the broader effects of DNMT1 overexpression on transcript levels of genes subject to dysregulation in these disorders is not known.

Both loss as well as increased levels of DNMT1 result in mid-gestational lethality (Li et al., 1992; Biniszkiewicz et al., 2002) whereas downregulation, but not its absence was suggested to be essential for differentiation (Kim et al., 2009; D’Aiuto et al., 2011). These observations make the task of development of mouse models that overexpress DNMT1 specifically in the brain tissues quite challenging. Given these difficulties, we proposed that stem cell models with increased DNMT1 levels are ideal to study the effects of overexpression on the neurological disease—associated genes and processes (Mohan, 2016). A second advantage of stem cell models is that *in vitro* differentiation of neurons recapitulates certain important stages related to the brain development in mouse (Li et al., 2009) and provide adequate amounts of cellular material for molecular studies. Along these lines, we developed and characterized the mouse *Dnmt1^tet/tet^* (*Tet/Tet*) ESCs as a model for studying the relationship between DNMT1 overexpression and abnormal neurogenesis (Borowczyk et al., 2009). Importantly, doxycycline treatment of *Tet/Tet* cells results in downregulation of *Dnmt1* and genome-wide hypomethylation (Saxena et al., 2020b). In the context of these features, analysis of *Tet/Tet* ESCs and their neurons in presence or absence of doxycycline allows investigation of effects of DNMT1 overexpression and loss in the same genetic background. By analyzing the transcriptomes of untreated and doxycycline-treated neurons using data on *R1* (wild-type) neurons as reference, we studied whether dysregulation of DNMT1 affects genes reported with altered transcript levels in SZ, BPD, EPD and ASD. In addition, the effects of DNMT1 overexpression on the markers associated with pluripotency and neuron differentiation were studied to relate their levels with the observed differences with the phenotypic abnormalities in *Tet/Tet* neurons. The results were discussed in the context of potential to reverse the DNMT1 overexpression-associated aberrant transcript levels to normal ranges by downregulation of DNMT1.

## Materials and methods

All reagents used were either of Molecular Biology or tissue culture-grade. Primary antibodies were from Cell Signaling Technologies, USA (OCT4, NANOG, SOX2, NESTIN, βIII-TUBULIN and MAP2) and BioLegend, USA (β-ACTIN and NEUN). Secondary antibodies were from Cell Signaling Technologies (USA).

### Embryonic stem cells

Wild-type *R1* and the mutant *Dnmt1^tet/tet^* (*Tet/Tet*) mouse ESCs were grown in ESC medium containing Knockout DMEM/F12 (ThermoFisher, USA), 10% fetal bovine serum (HiMedia, India), 1X non-essential amino acids (ThermoFisher, USA), 1X glutamax (ThermoFisher, USA), 100 μM β-mercaptoethanol (ThermoFisher, USA), 1X penicillin/streptomycin (HiMedia, India) and 1X leukemia inhibitory factor (LIF; Merck, USA). In case of *Tet/Tet* ESCs, the medium contained 150 μg/ml of G418 (ThermoFisher, USA) and 1 μg/ml puromycin (Merck, USA). The cells were seeded at 30% confluence in cell culture—treated flasks/dishes (ThermoFisher, USA) coated with 0.1% gelatin (Merck, USA) for at least 1 h. Gelatin was removed before plating the cells and the cells were grown in a CO_2_ incubator at 37°C with replenishment of media every alternate day or as needed. At confluence, the cells were rinsed twice with 1X phosphate-buffered saline (PBS), trypsinized with 1X trypsin-EDTA (Himedia, India) for 10 min, prepared as cell suspensions by pipetting and transferred to equal volumes of 1X ESC medium to inhibit trypsin. The cells were collected as pellets by centrifugation at 1,000 rpm in swing-out rotors, supernatants were discarded, the pellets were resuspended in fresh media and used for downstream experiments.

### Neuronal differentiation

Detailed protocol for neuron differentiation of mouse ESCs and monitoring of the process of differentiation was described (Saxena et al., 2020a). Briefly, G418 and puromycin were not added to the media during differentiation. ESC pellets were resuspended in embryoid body (EB) medium (ESC medium without LIF), plated on non-adherent dishes (ThermoFisher, USA) at a density of 50,000 cells/cm^2^ and grown in a 5% CO_2_ incubator at 37°C. After two days, the EBs were gently collected using 5-ml pipettes, transferred to 15-ml sterile conical bottom tubes (Tarsons, India) and were left standing at room temperature for 15 min to allow the EBs to settle at the bottom. The spent medium was aspirated out and the EBs were gently suspended in NDiff 227 medium (Takara, USA) and plated onto 0.2% gelatin-coated cell culture-treated dishes at five EBs/cm^2^. Half of the medium was replenished every day. After 8 days, clear differentiation into neurons was observed and the cells were harvested after 10th day of differentiation.

### Generation and analysis of transcriptome data

The details of transcriptome data involving replicates of *R1* and *Tet/Tet* neurons, identification of genes with dysregulated transcript levels were described earlier (Saxena et al., 2021; NCBI SRA: PRJNA941810). Data on dysregulated transcripts in ASD, EPD, BPD and SZ were extracted from publicly available information and published literature for comparisons with those from *Tet/Tet* neurons (Fromer et al., 2016; Gandal et al., 2018; Guelfi et al., 2019; Lanz et al., 2019; Pfisterer et al., 2020; Rahman et al., 2020). Dysregulated genes identified for each disorder were analyzed by generating Venn diagrams using the Bioinformatics and Evolutionary Genomics web-based tool.^1^ *Tet/Tet* neurons were treated with 1 μg/ml doxycycline over the last 3 days of differentiation and the RNA samples were subjected to RNA sequencing using Illumina-compatible NEBNext^®^ Ultra™ II directional RNA Library Prep Kit (New England Biolabs, USA) at Genotypic Technology, Pvt. Ltd., Bangalore, India as per manufacturer’s recommendations. After amplification, the libraries were purified and resolved on TapeStation^®^ for size verification. The libraries were sequenced using Novaseq 6000^®^ to obtain ≥ 50 million paired-end reads. After removal of adapter sequences and QC control to remove data with poor sequence quality, the sequences were mapped to the mouse reference genome version mm10. An overall mappable efficiency of 93.6–94.1% was observed. Sequence data for the doxycycline-treated neurons was deposited with NCBI SRA (PRJNA1012577). A summary of read statistics for all the three cell types used in this study are given in Supplementary Table 1. After normalization of all the three datasets (*R1* neurons, untreated and doxycycline-treated *Tet/Tet* neurons) using DeSeq2 module in the Partek Genomics Suite, LS mean values were obtained to calculate *p*-, q- values and log_2_-fold changes of transcript levels. Genes with significantly altered transcript levels (q value ≤ 0.05 and log_2_-fold changes ≥ 1.0 for upregulation and ≤ −1.0 for downregulation) were identified using *R1* neuron data as reference. Disease-associations (DisGeNet) (Piñero et al., 2017), KEGG Pathway analyses and Gene Ontology terms among the dysregulated transcripts were identified using ENRICHR (Kuleshov et al., 2016). Protein-protein interactions were studied using STRING (Szklarczyk et al., 2021).

### Immunofluorescence

Neurons were differentiated on coverslips (HiMedia, India) placed in sterile dishes. At the end of differentiation, medium was aspirated from the culture dishes and the neurons were gently rinsed thrice with 1X phosphate-buffered saline (PBS). The cells were then fixed with 4% paraformaldehyde (Sigma-Aldrich, USA) for 15 min at room temperature in dark on a rocker. The cells were again rinsed thrice with 1X PBS and blocked for an hour with 5% goat serum (HiMedia, India) in 1X PBS containing 0.5% Triton X-100 (Himedia, Cat no. MB031). The cells were then incubated overnight at 4°C with primary antibodies made in dilution buffer with 1% BSA (Himedia, India), 0.5% Triton X-100 and 1X PBS. The cells were rinsed thrice with 1X PBS and incubated for 2 h with fluorescently labeled secondary antibodies in freshly prepared antibody dilution buffers at room temperature and stained in dark with 1 μg/ml DAPI (ThermoFisher, USA) prepared in 1% DABCO (Sigma-Aldrich, USA) in a background buffer of 1X PBS. The coverslips were placed on glass slides and viewed using a confocal microscope (TCS SP8, Leica, Germany) and the data was analyzed using LasX Life Science software (Leica, Germany).

### Western blotting

Protein lysates prepared from neurons were subjected to western blot analyses using the indicated primary antibodies or β-ACTIN (loading control), followed by secondary antibodies, visualized using ECL chemiluminescence kit (Bio-Rad, USA) and Fusion Pulse 6 imaging system (Eppendorf, Germany) as described. The protocols followed were from Saxena et al. (2020b). The band intensities were normalized using signals obtained with β-ACTIN and relative amounts of the target proteins were determined using ImageJ software (Schneider et al., 2012). Western blot analyses were done on three replicates. The protein levels were compared by student’s *t*-test (two-tailed) and *p* ≤ 0.05 was taken as statistically significant.

## Results

*Dnmt1^tet/tet^* (*Tet/Tet*) cell line was a genetically engineered mouse embryonic stem cell (ESC) line with *Tet-off* cassettes inserted upstream to the *Dnmt1’s* start codon and downstream to its transcription start site. Due to transactivation, the levels of DNMT1 are higher in the ESCs as well as neurons (Saxena et al., 2020b). Because the tet-A transactivator is sensitive to doxycycline, treatment of the ESCs with this drug results in absence of *Dnmt1* transcription and genome-wide hypomethylation within 3 days (Borowczyk et al., 2009). These properties allowed us to use untreated and doxycycline-treated *Tet/Tet* neurons for determining the spectrum of genes showing significantly altered transcript levels in the context of those with transcript level dysregulation reported in SZ, ASD, EPD and BPD.

### Dysregulation of pluripotency and neuron markers due to DNMT1 overexpression

**FIGURE 1:**
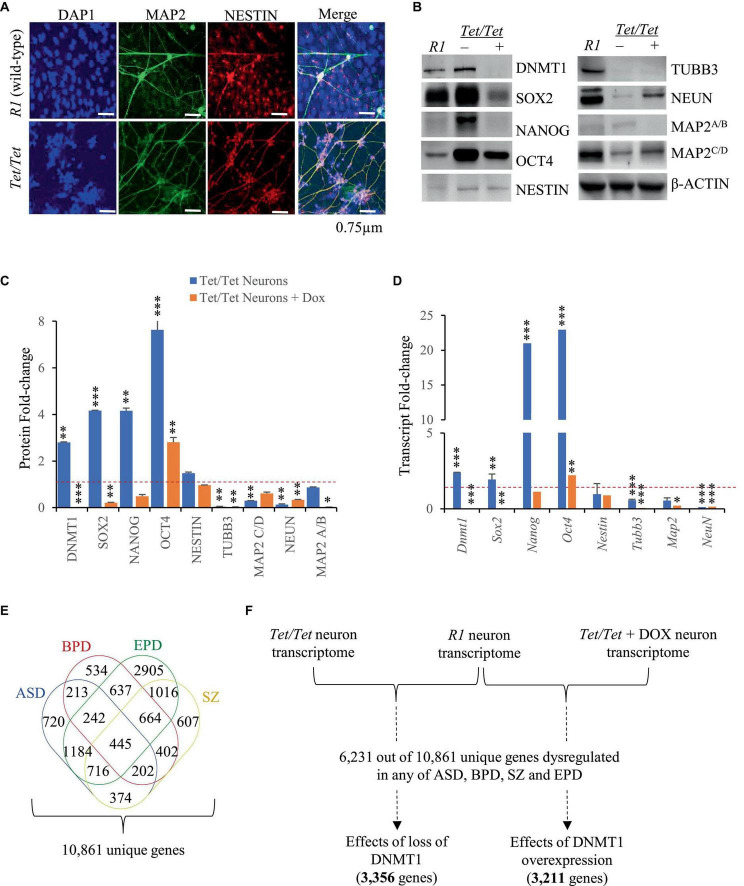
Neurodevelopmental defects associated with overexpression of DNMT1. **(A)** Immunofluorescence images of *R1* (wild-type) and *Dnmt1^tet/tet^* (*Tet/Tet*) neurons with DAPI (nucleus) and antibodies to NESTIN and MAP2 (mature neuron). **(B)** Representative western blot data using antibodies for DNMT1, OCT4, SOX2, NANOG, NESTIN, NEUN, βIII TUBULIN and MAP2 in *R1* (wild-type), untreated (- DOX) and treated (+ DOX) *Tet/Tet* neurons. β-ACTIN was used as loading control. Unprocessed western blots are given in Supplementary Figure 1. **(C)** Quantification of the western blot data from **(B)** and another two replicates after normalization with signals obtained using β-ACTIN. *p = 0.05–0.01; **p = 0.01–0.001; ***p = < 0.001. **(D)** Quantitative real-time PCR data of the indicated gene transcripts. Significance values are same as denoted for asterisks in **(C)**. **(E)** Identification of 10,861 genes with dysregulated transcript levels in schizophrenia (SZ) or autism spectrum (ASD) or epilepsy (EPD) or bipolar disorders (BPD). **(F)** Scheme showing analysis of 6,231 genes identified among transcripts in *R1*, untreated *Tet/Tet*, dox-treated neurons and any of the four neurodevelopmental disorders.

*Tet/Tet* neurons were previously reported to show certain phenotypic abnormalities like excessive dendritic branching and dysregulated N-methyl-D-aspartate (excitatory) receptor activity (D’Aiuto et al., 2011). Here, immunofluorescence experiments were carried to study the morphology of the neurons to detect NESTIN and MAP2 proteins and the data confirmed that *Tet/Tet* neurons were thinner and more branched than the *R1* counterparts (Figure 1A). To gain more insights into the effects of DNMT1 expression, western blot and qRT-PCR analyses were carried out for comparing the levels of pluripotency and neuron markers. Untreated *Tet/Tet* neurons confirmed increased levels of DNMT1 and after doxycycline treatment, DNMT1 was undetectable (Figure 1B). The data summarized in Figures 1C, D suggest ∼2.8- and 2.4- fold increase in the DNMT1 protein and its transcripts levels, respectively. These values agree with the RNA seq data that indicated ∼1.9-fold increase in the transcript levels (upregulation). The levels of pluripotency markers (OCT4, NANOG and SOX2) were higher in untreated *Tet/Tet* neurons whereas the levels of TUBB3 (immature neurons) and NEUN (mature neurons) were lower (Figures 1B, C). In the case of MAP2 (mature neurons), the levels of isoform C/D, but not A/B were lower. NESTIN (neuronal progenitor) levels were not significantly different from *R1* neurons. After doxycycline treatment, the pluripotency markers showed a decreased trend wherein the levels of only NANOG were not significantly different from the *R1* neurons. In case of neuronal markers, TUBB3 and MAP2 A/B showed a further decrease in the levels than the untreated cells. NEUN showed increased levels after doxycycline treatment but were still significantly lower than *R1* cells. These western blot data are consistent with the transcript level changes observed in the q-RT PCR experiments with the three different groups of neurons used (Figure 1D). Taken together, these results suggest that higher DNMT1 levels resulted in altered pluripotency, neuronal progenitor as well as neuron markers and these defects were partially recoverable in absence of DNMT1.

### DNMT1 overexpression results in altered transcript levels of genes dysregulated in neurodevelopmental disorders

**FIGURE 2:**
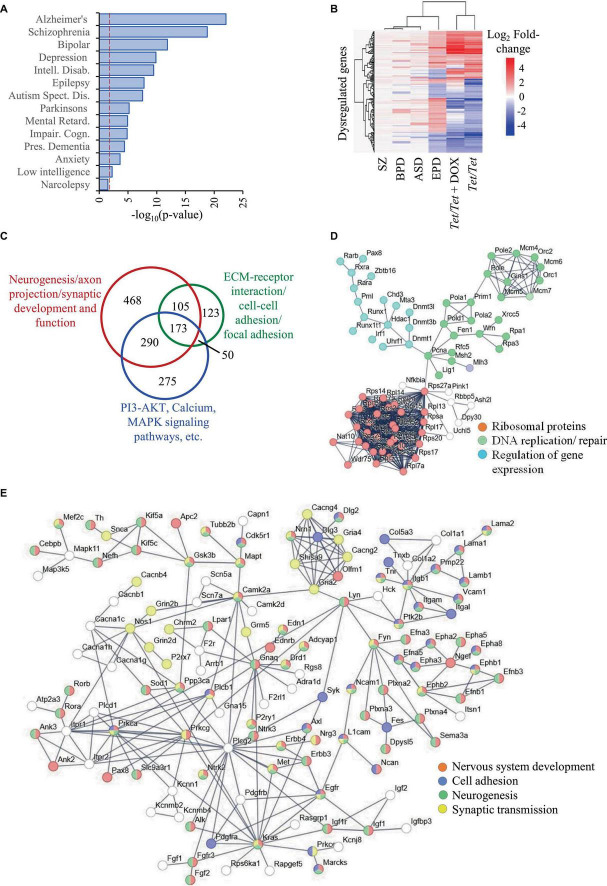
Dysregulation of genes due to DNMT1 overexpression and their relationship with SZ, BPD, EPD and ASD. **(A)** Disease ontology terms identified in 3,211 genes subjected to transcript level dysregulation in untreated *Tet/Tet* neurons from the list of 6,231 genes identified in Figure 1F. **(B)** Hierarchical clustering of the 296 genes and their corresponding log_2_ fold-changes among in the four neurodevelopmental disorders, untreated and doxycycline-treated *Tet/Tet* neurons. **(C)** Overview of the three main sets of pathways involving a majority of the 3,211 genes. **(D,E)** STRING analysis of genes involved in some main biological processes identified among the 3,211 genes.

We selected ASD, BPD, SZ and EPD as a group of four common neurodevelopmental disorders and obtained information on genes subjected to transcript level dysregulation from publicly available databases. This search yielded 19,670 dysregulated genes of which 8,809 were shared between any two disorders, leaving 10,861 unique set for comparisons with untreated and doxycycline-treated *Tet/Tet* neurons (Figure 1E). Of the 6,231 *q*-significant transcripts observed in untreated *Tet/Tet* neurons, 3,211 genes showed significantly altered transcripts (Figure 1F and Supplementary Table 2). Within the four disorders, enrichment was observed for SZ with highest significance, followed by BPD, EPD and ASD (Figure 2A and Supplementary Table 3). We also observed a significant enrichment for other neurological/behavioral and neurodegenerative phenotypes, with Alzheimer’s showing the most significant enrichment among all disorders. A comparison of the log_2_-fold values of the dysregulated genes among the four neurodevelopmental disorders, untreated and doxycycline-treated *Tet/Tet* neurons is shown in Figure 2B. The dysregulation patterns in the treated as well as untreated *Tet/Tet* neurons were more similar to those in EPD. Bioinformatic analysis of the genes dysregulated in the untreated *Tet/Tet* neurons showed significant enrichment of pathways implicated in neurodevelopment, axon guidance, excitatory as well as inhibitory synapses, etc (Figure 2C and Supplementary Table 4). Similar mutually agreeing results were obtained using gene ontology terms (Supplementary Table 5) and protein-protein interaction analyses (Figures 2D, E).

When the genes with common patterns of dysregulation in all the four disorders were considered, 281 genes were identified of which, 89 were downregulated and 192 were upregulated. Of the 89 genes, 36 were also dysregulated in *Tet/Tet* neurons of which 33 showed downregulation, three with upregulation and the remaining 53 did not show any dysregulation. A similar comparison of the 192 genes showed 124 as neutral, 35 as upregulated and 33 downregulated. Together, out of 104 dysregulated genes among *Tet/Tet* neurons and the four disorders, 68 (∼65%) showed similar patterns of dysregulation (Supplementary Table 6).

### DNMT1 downregulation also results in altered transcript levels of genes dysregulated in neurodevelopmental disorders

**FIGURE 3:**
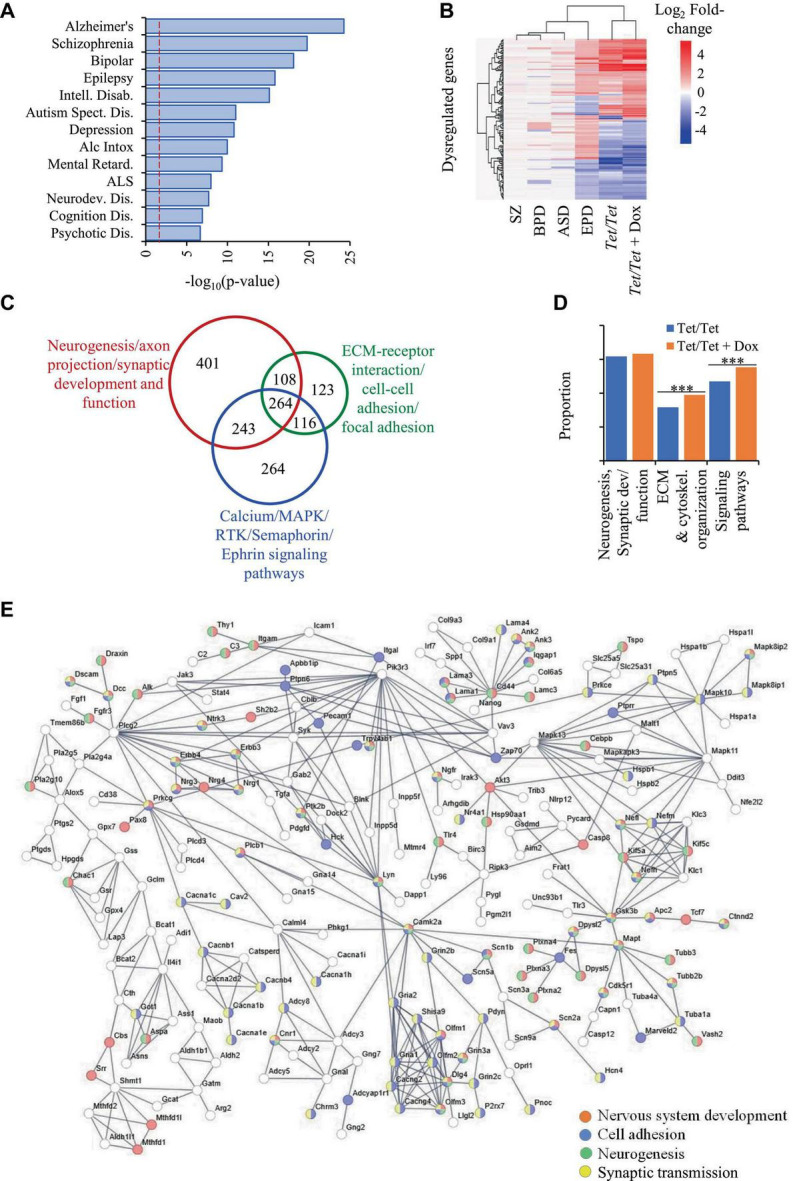
Dysregulation of genes due to loss of DNMT1 and their relationship with SZ, BPD, EPD and ASD. **(A)** Disease ontology terms identified in 3,356 genes with transcript level dysregulation in the four disorders, untreated and DOX-treated *Tet/Tet* neurons. **(B)** Hierarchical clustering of the 157 genes using log_2_ fold-changes in the six different datasets as indicated. **(C)** STRING analysis of the 3,356 genes involved in biological processes implicated in the four disorders. **(D)** Proportions of some main dysregulated signaling pathways observed in untreated and doxycycline-treated *Tet/Tet* neurons. *** indicates *p* values < 0.001. **(E)** STRING analysis of dysregulated genes involved in some main biological processes identified among 3,356 genes dysregulated in doxycycline-treated *Tet/Tet* neurons.

Comparisons of transcriptome data on *Tet/Tet* neurons deficient in DNMT1 after doxycycline treatment with the 6,270 q-significant genes yielded 3,356 with significantly altered transcript levels (Supplementary Table 7). As in the case of untreated neurons, the genes dysregulated in *Tet/Tet* neurons also showed significant enrichment of the four disorders under investigation as well as those with neurodegenerative and behavioral phenotypes (Figure 3A and Supplementary Table 8). Hierarchical clustering and bioinformatic analyses of these dysregulated genes also gave results similar to those using untreated *Tet/Tet* neurons (Figures 2B, C, 3B, C and Supplementary Tables 9, 10). Although similar biological processes were observed, the treated cells showed higher significant values for Extra Cellular Matrix (ECM)/cytoskeletal organization and signaling pathways (Figure 3D). STRING analysis confirmed the neurodevelopmental, cell adhesion, cell-cell signaling and synaptic function/developmental pathways through protein-protein interactions (Figure 3E). The results on transcriptional dysregulation patterns and bioinformatic analyses suggest that the genes affected in untreated and doxycycline-treated neurons participate in similar pathways but may have dissimilar dysregulation patterns.

As in case of comparisons involving the 281 genes mentioned above, we observed similar proportions of genes (∼69%) with identical pattens of dysregulation in doxycycline-*Tet/Tet* neurons (Supplementary Table 6).

### Common and distinct patterns of gene dysregulation due to DNMT1 overexpression and its downregulation

**FIGURE 4:**
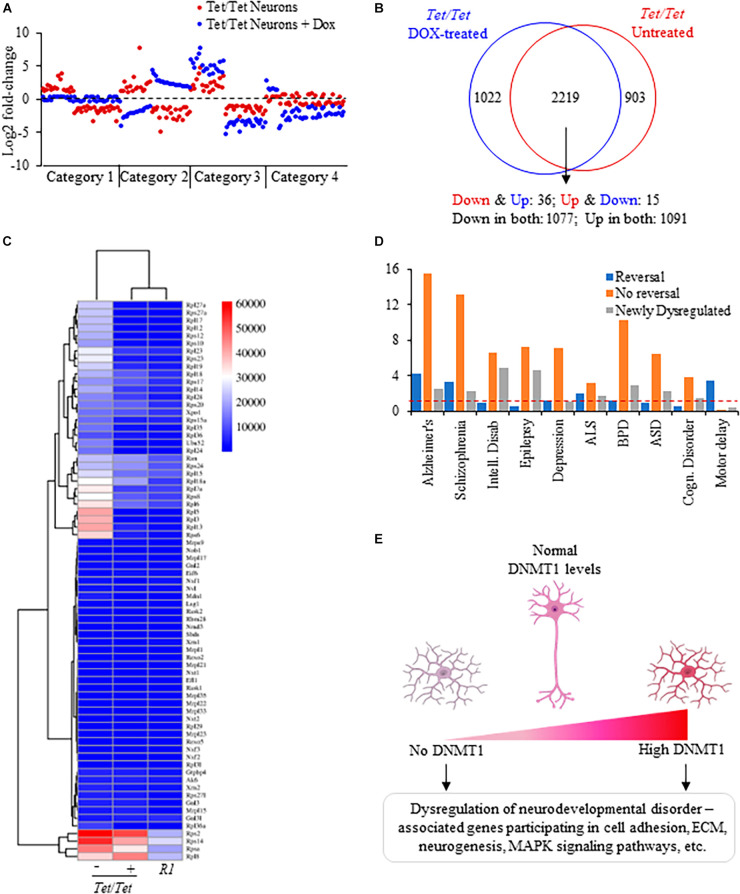
Analysis of dysregulated genes due to DNMT1 overexpression and its loss. **(A)** Representative log_2_ fold transcript level changes of genes in untreated and DOX-treated *Tet/Tet* neurons. Blue: transcript levels after DOX-treatment; Red: transcript levels in untreated cells. **(B)** Comparison of genes subject to transcript level dysregulation between untreated and dox-treated *Tet/Tet* neurons. Top: Venn diagram with genes specifically dysregulated in cell types indicated. Bottom: number of genes with altered transcript levels in both untreated and doxycycline treated *Tet/Tet* neurons. Down and Down: decreased transcript levels in both untreated and dox-treated cells, Up and Up: increased transcript levels in both cell types, Down and Up: decreased transcript levels in untreated cells but with increased levels in dox-treated cells, Up and Down: increased transcript levels in untreated cells but with decreased levels in dox-treated cells. **(C)** Heatmap of the 73 genes involved in the ribosome biogenesis/assembly/translation pathways implicated in the four disorders of interest. Whereas 51 genes showed significantly altered levels in untreated *Tet/Tet* neurons, only 15 were dysregulated after doxycycline treatment (*p* ≤ 0.0001). Numbers next to the scale indicate LS mean values of the transcripts. **(D)** Comparisons of disorders enriched among genes showing reversal in transcript level changes, those showing no reversal and those dysregulated due to doxycycline treatment (newly dysregulated). Red dotted line indicates cutoff for significant *p*-value (0.05). **(E)** Summary of gene dysregulation observed in neurons lacking and those with increased levels of DNMT1. Both conditions resulted in dysregulation of genes that also showed altered transcript levels in patients with ASD, BPD, EPD or SZ disorder affecting the indicated pathways. These results suggest that for normal neurodevelopment, DNMT1 levels should be within a tight range.

Comparisons of the directions of transcript level changes in untreated and doxycycline-treated *Tet/Tet* neurons yielded four distinct categories of genes (Figure 4A). About 30% of genes dysregulated in the untreated cells showed opposite changes in the direction of transcript levels in absence of DNMT1 (category 1). As a result, the transcript levels were not significantly different from the wild-type *R1* neurons for these 903 genes (Figure 4B and Supplementary Table 11). A set of 51 genes showed an opposite change in the direction of dysregulation but were significantly altered relative to *R1* neurons (category 2). Nearly 68% of the genes (2,168 out of 3,211) continued to be in the same direction of dysregulation of which 304 showed further significant alterations compared to untreated cells (category 3). The fourth category of 1,022 genes (∼30%) included those that did not show dysregulation prior to doxycycline treatment but showed significant transcript level changes after doxycycline treatment. Protein-protein interaction analysis of genes subjected to a reversal in the direction of transcript-level dysregulation involved the ribosomal protein assembly (translation) pathway that was also shared with the untreated *Tet/Tet* neurons (Figures 2D, 4C). These “reversed” genes showed a significantly higher proportion of dysregulated genes for SZ, Alzheimer’s, Amyotrophic lateral sclerosis and motor delay disorders. Corresponding data on genes without reversal or specific dysregulation after doxycycline treatment are shown in Figure 4D. Thus, many genes showing dysregulation due to DNMT1 overexpression are more likely to be irreversible.

## Discussion

DNMT1 overexpression is one of the common features associated with schizophrenia, bipolar and epilepsy disorders (Veldic et al., 2005; Zhu et al., 2012). Although there is no clear data on the levels of DNMT1 in autism spectrum disorders, two risk-conferring variants of DNMT1 that have been predicted to result in its over expression were reported in patients (Alex et al., 2019). In addition to these data, tissue-specific knockout experiments in mice showed that forebrain-specific loss of DNMT1, but not DNMT3A resulted in behavioral abnormalities (Morris et al., 2016). However, the spectrum of genes subjected to transcript level dysregulation due to DNMT1 overexpression and their overlap or differences from those dysregulated due to DNMT1 loss and in turn, their relationship with the four disorders mentioned above is not known. The *Tet/Tet* mouse embryonic stem cell line used in this study is a useful model to identify dysregulated genes under both conditions because of overexpression of DNMT1 in absence of doxycycline and its downregulation when treated with doxycycline (Saxena et al., 2020b). Therefore, this cell line allowed investigation of DNMT1 loss as well as its overexpression effects on the transcriptome under the same genetic background.

Recent data on DNMT1’s role in preimplantation development suggest that the protein enables transition to and maintenance of pluripotent state in the mouse embryo (Fu et al., 2019). Given this evidence and the reported downregulation accompanying differentiation (D’Aiuto et al., 2011) suggest that DNMT1 overexpression may result in dysregulation of pluripotency markers and neuronal differentiation. In support of this possibility, we observed that OCT4, NANOG and SOX2 levels were all higher in *Tet/Tet* neurons. This feature, accompanied by lower levels of βIII-TUBULIN (TUBB3), MAP2 and NEUN and moderately increased levels of NESTIN indicate a neuronal differentiation defect in the *Tet/Tet* cells. In absence of DNMT1, doxycycline-treated *Tet/Tet* cells showed a further decline in two of the three neuronal markers tested whereas the pluripotency markers although significantly different from the *R1* neurons, changed toward the normal levels. These data suggest that absence of DNMT1 as well as its overexpression impacted the neuronal marker levels suggesting abnormal neuronal differentiation. The molecular basis of this defect needs to be investigated in detail.

Previous data on *Tet/Tet* neurons drew some parallels with schizophrenia, the most significant being dysregulation of ∼50% of the genes that were also reported with altered transcript levels in patients with ∼30% showing identical patterns (Saxena et al., 2021). Evidence suggests that dysregulation was not associated with DNA methylation changes and in fact, the *Tet/Tet* neuron genome was hypomethylated with increased LINE-1 burden (Saxena et al., 2021). However, the effects of DNMT1 loss on the neuron transcriptome and its relationship with behavioral abnormalities was not known. This was because absence of DNMT1 or its catalytic activity did not interfere with neuron differentiation, but the differentiated neurons did not survive (Fan et al., 2001). Even in our experiments, when doxycycline treatment was continued during differentiation from the ESC stage, the embryoid bodies did not survive. Thus, studying neuron differentiation in absence of DNMT1 was not possible. However, post-differentiation, the *Tet/Tet* neurons survived in absence of DNMT1 and enabled us to investigate for the first time the effects of DNMT1 loss in neurons. In this context, we extended our findings on DNMT1 dysregulation on three other neurodevelopmental disorders i.e., ASD, EPD and BPD, along with SZ in all of which, epigenetic mechanisms have been suggested to play a role.

Our initial analysis of genes with transcript level dysregulation in the four disorders revealed the presence of shared as well as unique genes. Importantly, several shared genes showed dissimilar patterns of dysregulation (Figures 2B, 3B). For example, transcript levels of *Abcb9* which are reduced in *Tet/Tet* neurons were also reported to be downregulated in ASD and SZ but upregulated in EPD and BPD. Similarly, *Ptk2b* that showed upregulation in *Tet/Tet* neurons was also reported to show increased transcript levels in BPD and SZ but downregulated in ASD and EPD. In addition, many genes were reported to be dysregulated in one disorder but not in the others. For instance, *A2M* was reported to be dysregulated with upregulation in EPD but with downregulation in BPD whereas there was no dysregulation in ASD and SZ. These data suggest that alteration in transcript levels may be the most plausible cause for these abnormalities rather than the direction of dysregulation. Future studies that test the possibility of gene dosage effects as an underlying mechanism may bring clarity on these discrepancies. Nevertheless, in this study, we considered dysregulation regardless of the direction of transcript level change while comparing the data on patients and wherever possible the directions of transcript level changes were considered. For example, the dysregulation profiles of *Tet/Tet* neurons were more similar to those in epilepsy and least to schizophrenia within the four disorders under consideration (Figure 1B). This pattern did not change even after loss of DNMT1. Although there was a reversal in a small subset of genes to near-normal transcript levels after doxycycline treatment, a similar proportion of genes were specifically dysregulated.

Data on mice and humans with altered levels of DNMT1 suggested that both increased and decreased levels are associated with phenotypic and developmental abnormalities (Lei et al., 1996; Grayson et al., 2005; Veldic et al., 2005; Trowbridge et al., 2009; D’Aiuto et al., 2011; Dong et al., 2015). While loss of DNMT1 or its overexpression results in mid-gestational lethality in mice (Biniszkiewicz et al., 2002), increased DNMT1 levels, as mentioned above, were reported in SZ, EPD and BPD (Veldic et al., 2005; Zhu et al., 2012). Mice heterozygous for *Dnmt1^null^* allele showed behavioral abnormalities (Morris et al., 2016) whereas in humans, rare dominant mutations in *Dnmt1* predicted to produce unstable and defective protein cause hereditary sensory and neuropathy type 1E with behavioral abnormalities as comorbid phenotypes (Klein et al., 2011). These data, taken together with the data presented in this report strongly support the requirement of maintenance of DNMT1 levels within a tight range to ensure normal development and health (summarized in Figure 4E). This requirement also forms the basis for future therapeutic approaches that reduce DNMT1 or its activity to treat the subset of patients with increased DNMT1 levels.

## Data availability statement

The datasets presented in this study can be found in online repositories. The names of the repository/repositories and accession number(s) can be found in this article/Supplementary material.

## Ethics statement

No animal studies are presented in this manuscript.

## Author contributions

MS: Data curation, Formal analysis, Investigation, Methodology, Software, Validation, Writing – original draft, Writing – review & editing. SS: Methodology, Writing – review & editing, Formal analysis, Visualization. KM: Writing – review & editing, Conceptualization, Formal analysis, Funding acquisition, Project administration, Resources, Supervision, Visualization, Writing – original draft, Investigation.

## Footnotes

1
https://bioinformatics.psb.ugent.be/webtools/Venn/
