# Supplementary material for: DNMT1 downregulation as well as its overexpression distinctly affect mostly overlapping genes implicated in schizophrenia, autism spectrum, epilepsy, and bipolar disorders
Source: Front Mol Neurosci. 2023 Dec 6;16:1275697. doi: 10.3389/fnmol.2023.1275697 (PMC10731955; doi:10.3389/fnmol.2023.1275697)
Supplement: Supplementary file 1 [file Data_Sheet_1.pdf]

Supplementary Table1. Read statistics of transcriptome data from *R1* , untreated and doxycycline-treated *Tet/Tet* neurons

**Sample name**	**Raw read count**	**QC-passed reads**	**Alignment percentage**	**Avg. quality (Phred)**
R1 Neurons Rep1	47269285	44251125	93.615	38.886
R1 Neurons Rep2	46495859	43630512	93.837	38.923
Tet/Tet Neurons Rep1	39795783	37260503	93.629	38.899
Tet/Tet Neurons Rep2	54156153	50819487	93.839	38.935
Tet/Tet+ Dox Neurons Rep 1	48656402	45673798	93.870	35.803
Tet/Tet+ Dox Neurons Rep 2	53808671	50626439	94.086	35.887

Supplementary Table 2. List of genes with significantly altered transcript levels in untreated *Tet/Tet* neurons

Gene	*p* -value	Log_2 Ratio	Q-value (Benjamini-Hochberg)
A2m	0.0000	1.3834	0.0218
A4galt	0.0000	2.0868	0.0133
Aaas	0.0000	1.1296	0.0149
Aard	0.0000	2.3622	0.0280
Aars	0.0000	1.2328	0.0033
Aass	0.0000	4.2593	0.0258
Aatk	0.0000	-2.4783	0.0102
Abcb9	0.0000	-1.7035	0.0333
Abcc4	0.0000	2.1755	0.0039
Abcg5	0.0000	4.7788	0.0283
Abhd18	0.0000	-1.0096	0.0323
Abhd2	0.0000	3.8934	0.0001
Abhd3	0.0001	1.8215	0.0456
Abi3	0.0002	3.2770	0.0482
Ablim1	0.0000	-1.2731	0.0104
Acacb	0.0000	1.4842	0.0215
Acap1	0.0000	5.9884	0.0163
Acap3	0.0000	-1.3965	0.0091
Acat2	0.0000	-1.3678	0.0036
Acbd4	0.0000	1.0617	0.0339
Accsl	0.0000	5.8054	0.0190
Ace	0.0000	2.4789	0.0149
Ache	0.0000	-1.9486	0.0109
Ackr3	0.0000	-1.7244	0.0132
Acot1	0.0000	-2.1048	0.0049
Acot11	0.0000	-1.1380	0.0389
Acot9	0.0000	1.3470	0.0137
Acp5	0.0004	1.4338	0.0498
Acp6	0.0000	1.9232	0.0036
Acpp	0.0000	5.6302	0.0076
Acsbg1	0.0000	-1.4204	0.0363
Acsl1	0.0000	1.2906	0.0121
Acsl5	0.0000	1.2545	0.0265
Acsl6	0.0000	-1.1138	0.0207
Acss3	0.0000	4.7799	0.0014
Actc1	0.0000	-2.8334	0.0213
Actl6b	0.0000	-2.2584	0.0070
Actn1	0.0000	1.0264	0.0083
Actn2	0.0000	-1.8614	0.0405
Actn4	0.0000	1.0082	0.0081
Actr1a	0.0000	-1.2143	0.0048
Actr3	0.0000	1.1875	0.0035
Acvr2a	0.0000	-1.0982	0.0120
Adad2	0.0000	3.8305	0.0447
Adam11	0.0000	-1.7743	0.0125
Adam12	0.0000	-2.0421	0.0154

Adam23	0.0000	1.1776	0.0072
Adamts1	0.0003	-1.2310	0.0487
Adamts10	0.0000	-1.0327	0.0256
Adamts12	0.0000	-2.0213	0.0073
Adamts16	0.0000	-2.9759	0.0095
Adamts17	0.0000	-1.0990	0.0453
Adamts18	0.0000	-4.8402	0.0192
Adamts2	0.0000	-2.4977	0.0067
Adamts20	0.0000	-2.3354	0.0284
Adamts3	0.0000	2.8846	0.0028
Adamts5	0.0000	-1.5086	0.0298
Adamts6	0.0000	-1.2040	0.0388
Adamts7	0.0000	-1.5712	0.0113
Adamtsl1	0.0000	-2.5251	0.0317
Adap2	0.0001	3.0790	0.0455
Adarb2	0.0000	-1.0799	0.0393
Adcy1	0.0000	-1.0967	0.0192
Adcy2	0.0000	-2.3451	0.0077
Adcy5	0.0000	-1.9614	0.0053
Adcy8	0.0000	3.2214	0.0048
Adcyap1	0.0000	1.6583	0.0388
Adcyap1r1	0.0000	-2.6213	0.0019
Add2	0.0000	-1.9703	0.0028
Adgrb1	0.0000	-1.6708	0.0080
Adgrb2	0.0000	-1.2301	0.0067
Adgre5	0.0000	1.4061	0.0147
Adgrg6	0.0000	-1.5091	0.0227
Adgrl3	0.0000	-1.0797	0.0164
Adgrv1	0.0000	-1.4658	0.0075
Adk	0.0000	1.3144	0.0098
Adora1	0.0000	-1.4942	0.0340
Adora2b	0.0000	-2.0291	0.0416
Adra1a	0.0000	1.4333	0.0299
Adra1d	0.0000	-3.9821	0.0250
Adra2a	0.0000	-3.2609	0.0063
Adrm1	0.0000	1.5156	0.0046
Afap1	0.0000	-1.4232	0.0057
Afap1l2	0.0000	1.1448	0.0433
Aff1	0.0000	1.7957	0.0035
Aff2	0.0000	-2.3594	0.0125
Aga	0.0000	2.0814	0.0064
Agap2	0.0000	-1.4314	0.0124
Agbl2	0.0000	2.8778	0.0265
Agk	0.0000	2.0440	0.0059
Ago4	0.0000	-1.1809	0.0246
Agpat2	0.0000	1.8304	0.0168
Ahcy	0.0000	5.5363	0.0001
Ahnak2	0.0000	1.1141	0.0310
Ahrr	0.0000	4.6302	0.0368

Ahsa1	0.0000	1.1832	0.0085
Aifm3	0.0000	3.8754	0.0119
Aim2	0.0000	3.8881	0.0033
Aire	0.0000	4.8170	0.0099
Ajap1	0.0000	-1.1031	0.0353
Ajuba	0.0000	1.2121	0.0078
Ak7	0.0000	2.7320	0.0198
Ak9	0.0000	2.6233	0.0422
Akap6	0.0000	-1.6809	0.0028
Akip1	0.0000	1.0087	0.0360
Alcam	0.0000	-2.2460	0.0191
Aldh1a1	0.0000	-2.2365	0.0404
Aldh1a2	0.0000	-3.9431	0.0030
Aldh1a3	0.0000	-4.3570	0.0177
Aldh1l1	0.0000	1.5215	0.0199
Aldh2	0.0000	1.3550	0.0051
Alg13	0.0000	2.8457	0.0009
Alg8	0.0000	1.1072	0.0373
Alk	0.0001	-1.5579	0.0468
Alkbh2	0.0000	1.8007	0.0208
Alox15	0.0000	3.7095	0.0423
Alpk1	0.0000	-1.7510	0.0405
Alpk3	0.0000	1.2933	0.0342
Alpl	0.0000	2.4354	0.0018
Als2cl	0.0000	1.7899	0.0284
Amd1	0.0000	1.6486	0.0029
Amer3	0.0000	-1.4874	0.0199
Amigo1	0.0000	-1.1941	0.0202
Amph	0.0000	-1.2492	0.0150
Anapc10	0.0000	1.0215	0.0232
Angpt1	0.0000	-3.7219	0.0035
Angpt2	0.0000	-1.1273	0.0454
Angpt4	0.0003	-2.9736	0.0496
Angptl1	0.0000	-3.6334	0.0212
Angptl4	0.0000	4.2708	0.0123
Ank1	0.0000	-1.5483	0.0319
Ank2	0.0000	-1.4325	0.0041
Ank3	0.0000	-1.2627	0.0044
Ankrd12	0.0000	-1.0355	0.0144
Ankrd13b	0.0000	-1.3179	0.0117
Ankrd34a	0.0003	-1.6110	0.0489
Ankrd35	0.0000	2.5398	0.0239
Ankrd45	0.0000	2.5591	0.0162
Ankrd46	0.0000	-1.0953	0.0096
Ankrd50	0.0000	-1.3569	0.0050
Ankrd6	0.0000	-1.0955	0.0157
Ankrd9	0.0000	1.0117	0.0446
Anks1b	0.0000	-1.6137	0.0221
Anln	0.0000	1.8509	0.0021

Ano1	0.0000	3.5247	0.0033
Anp32b	0.0000	1.0144	0.0110
Anpep	0.0000	-1.9006	0.0121
Anxa1	0.0000	1.2318	0.0214
Anxa11	0.0000	2.9313	0.0016
Anxa3	0.0000	1.4541	0.0219
Anxa4	0.0000	2.5631	0.0027
Anxa7	0.0000	2.1774	0.0025
Aoah	0.0000	4.6244	0.0349
Ap1g2	0.0000	1.3003	0.0368
Ap1m2	0.0000	2.7442	0.0264
Ap1s3	0.0000	3.4562	0.0129
Ap5s1	0.0000	-1.2506	0.0304
Apba1	0.0000	-1.7331	0.0092
Apba2	0.0000	-1.3977	0.0092
Apbb1	0.0000	-1.2390	0.0072
Apbb1ip	0.0000	3.8416	0.0373
Apc2	0.0000	-1.5983	0.0034
Aph1b	0.0000	-1.4865	0.0118
Apobec2	0.0000	2.4227	0.0264
Apobr	0.0000	2.0221	0.0421
Apoc1	0.0000	3.8916	0.0109
Apoe	0.0000	1.8470	0.0035
Apold1	0.0000	4.1074	0.0144
Apoo	0.0000	2.7070	0.0075
Aqp1	0.0000	-2.2606	0.0227
Aqp3	0.0000	2.0632	0.0153
Aqp4	0.0001	-1.6662	0.0467
Arc	0.0000	3.3207	0.0071
Arg2	0.0001	-1.7163	0.0464
Arhgap18	0.0000	1.5487	0.0122
Arhgap20	0.0000	-2.1183	0.0125
Arhgap24	0.0000	-1.1488	0.0383
Arhgap27	0.0002	1.3155	0.0479
Arhgap28	0.0000	-1.9305	0.0073
Arhgap29	0.0000	-1.7355	0.0054
Arhgap30	0.0000	3.7533	0.0090
Arhgap31	0.0000	-1.5281	0.0194
Arhgap33	0.0000	-1.3506	0.0071
Arhgap36	0.0000	-3.8720	0.0097
Arhgap4	0.0001	1.3650	0.0477
Arhgap6	0.0000	-2.0583	0.0392
Arhgap9	0.0000	3.0695	0.0307
Arhgdib	0.0000	2.4240	0.0152
Arhgdig	0.0000	-1.0464	0.0397
Arhgef10l	0.0000	-1.2391	0.0130
Arhgef15	0.0000	1.7457	0.0396
Arhgef16	0.0000	1.6909	0.0317
Arhgef18	0.0000	2.2721	0.0008

Arhgef25	0.0000	-1.0123	0.0150
Arhgef3	0.0000	2.0184	0.0116
Arhgef4	0.0000	-2.0528	0.0058
Arid3b	0.0000	1.1712	0.0142
Arid5a	0.0000	1.3679	0.0393
Arl4d	0.0000	-3.1589	0.0041
Armc3	0.0000	3.8458	0.0283
Armcx4	0.0000	-1.1627	0.0063
Arntl	0.0000	1.3605	0.0257
Arpc1b	0.0000	1.1348	0.0158
Arrb1	0.0000	-1.4811	0.0053
Arx	0.0000	-5.9717	0.0030
Asah2	0.0000	-1.9332	0.0305
Asap3	0.0000	-1.2353	0.0294
Asb13	0.0000	-1.6647	0.0133
Ascl2	0.0000	2.9413	0.0274
Asf1b	0.0000	1.3911	0.0172
Ash2l	0.0000	1.5663	0.0022
Asic1	0.0000	-2.2512	0.0017
Asic2	0.0000	-1.2244	0.0325
Asic4	0.0000	-2.0473	0.0049
Asna1	0.0000	1.3533	0.0077
Asns	0.0000	2.4300	0.0007
Aspa	0.0003	2.7669	0.0495
Asphd1	0.0000	-2.1348	0.0289
Aspm	0.0000	1.3283	0.0093
Ass1	0.0000	4.0842	0.0005
Astn1	0.0000	-1.0207	0.0144
Astn2	0.0000	-2.1253	0.0075
Asxl3	0.0000	-1.6481	0.0083
Atad2	0.0000	1.5520	0.0042
Atat1	0.0000	-1.3300	0.0071
Atcay	0.0000	-2.1841	0.0026
Atf1	0.0000	1.4939	0.0076
Atf5	0.0000	2.5569	0.0007
Atf7ip2	0.0000	3.2686	0.0175
Atic	0.0000	1.7421	0.0026
Atmin	0.0000	1.0578	0.0120
Atoh7	0.0000	4.4199	0.0390
Atp10a	0.0000	1.1791	0.0353
Atp10d	0.0000	2.7916	0.0026
Atp12a	0.0000	2.7088	0.0443
Atp13a2	0.0000	-1.1516	0.0086
Atp1a2	0.0000	-3.6448	0.0008
Atp1a3	0.0000	-1.1616	0.0106
Atp2a3	0.0000	1.2405	0.0311
Atp2b2	0.0000	-1.5945	0.0162
Atp5e	0.0000	-1.1454	0.0153
Atp5g1	0.0000	2.1484	0.0031

Atp6v0a4	0.0000	3.6554	0.0313
Atp6v0e2	0.0000	-1.2733	0.0091
Atp6v1g2	0.0000	-2.2432	0.0155
Atp8a1	0.0000	-1.1768	0.0139
Atp8a2	0.0000	-1.6321	0.0140
Atxn1	0.0000	-1.4318	0.0281
Aurka	0.0000	1.3112	0.0114
Auts2	0.0000	-1.5520	0.0041
Aven	0.0000	1.0221	0.0301
Avl9	0.0000	-1.2678	0.0073
Avpi1	0.0000	1.5041	0.0127
Axl	0.0000	-1.3694	0.0125
B3galt2	0.0000	-1.6259	0.0343
B3galt4	0.0000	-1.3099	0.0407
B3galt5	0.0000	-3.1325	0.0289
B3gat1	0.0000	-1.5325	0.0133
B3gat2	0.0000	-1.0214	0.0411
B3gnt2	0.0000	2.8891	0.0037
B3gnt7	0.0000	3.3354	0.0013
B3gnt8	0.0003	-1.8108	0.0491
B4galnt3	0.0000	2.5185	0.0160
B4galt1	0.0000	1.0982	0.0158
B4galt2	0.0000	-1.9227	0.0044
B4galt4	0.0000	-1.3224	0.0168
B4galt7	0.0000	-1.1928	0.0169
Baalc	0.0000	-2.0051	0.0437
Bace1	0.0000	-1.5078	0.0052
Bace2	0.0000	-3.9639	0.0161
Bach2	0.0000	-2.0741	0.0036
Bambi	0.0000	3.5861	0.0024
Bard1	0.0000	1.9504	0.0050
Basp1	0.0000	-1.5163	0.0051
Bcam	0.0000	1.1676	0.0165
Bcan	0.0000	-3.7566	0.0342
Bcar3	0.0000	1.2873	0.0152
Bcas1	0.0000	1.9532	0.0345
Bcat1	0.0000	1.8167	0.0042
Bcat2	0.0000	1.6912	0.0082
Bckdhb	0.0000	1.2502	0.0151
Bcl11a	0.0000	-1.6892	0.0074
Bcl11b	0.0000	-2.1848	0.0123
Bcl3	0.0000	1.8321	0.0334
Bcl7a	0.0000	-1.4495	0.0043
Bdh2	0.0002	1.2166	0.0480
Bean1	0.0000	-2.0998	0.0278
Begain	0.0000	-1.7234	0.0161
Bend5	0.0000	-1.6457	0.0135
Bend7	0.0000	-2.5484	0.0156
Bex1	0.0000	1.0849	0.0143

Bex4	0.0000	1.1859	0.0100
Bfsp1	0.0000	3.2408	0.0429
Bgn	0.0000	-4.9638	0.0015
Bhlhe22	0.0000	-4.9065	0.0008
Bicc1	0.0000	-1.4428	0.0202
Bicd1	0.0000	-1.3310	0.0130
Bicd2	0.0000	-1.0005	0.0128
Bin2	0.0000	4.3355	0.0066
Blcap	0.0000	-1.3454	0.0051
Blnk	0.0000	4.8076	0.0353
Bloc1s6	0.0000	-1.0781	0.0146
Bmf	0.0000	-1.1096	0.0166
Bmp1	0.0000	-2.0890	0.0020
Bmp2	0.0003	-1.3673	0.0488
Bmp6	0.0000	2.3652	0.0070
Bmper	0.0001	-1.1603	0.0460
Bok	0.0000	1.4426	0.0256
Brca1	0.0000	1.4212	0.0079
Brca2	0.0000	1.0950	0.0145
Brinp1	0.0000	-2.2549	0.0140
Brip1	0.0000	1.3655	0.0196
Brix1	0.0000	1.0213	0.0104
Brsk1	0.0000	-1.3902	0.0061
Brsk2	0.0000	-1.3969	0.0072
Bsdc1	0.0000	-1.0367	0.0159
Bsn	0.0000	-2.3848	0.0024
Bst2	0.0000	2.4028	0.0041
Btbd19	0.0000	1.4056	0.0266
Btf3l4	0.0000	1.4667	0.0046
Btg3	0.0000	1.7380	0.0074
Bub1	0.0000	1.6678	0.0033
Bub1b	0.0000	1.5068	0.0047
C1ql1	0.0000	1.3736	0.0246
C1ql3	0.0000	-2.1177	0.0332
C1qtnf2	0.0003	-3.5518	0.0493
C2	0.0000	2.1294	0.0300
Cab39l	0.0000	1.0681	0.0158
Cabp1	0.0000	-1.0481	0.0284
Cacna1c	0.0000	-1.7397	0.0108
Cacna1g	0.0000	-1.0207	0.0176
Cacna1h	0.0000	-2.0122	0.0029
Cacna2d2	0.0000	-2.0788	0.0040
Cacna2d3	0.0000	-2.5162	0.0059
Cacnb1	0.0000	-1.2855	0.0158
Cacnb4	0.0000	-1.1757	0.0360
Cacng2	0.0000	-1.9545	0.0136
Cacng4	0.0000	-1.0006	0.0136
Cacng6	0.0000	2.9742	0.0297
Cadm2	0.0000	-2.2821	0.0113

Cadm3	0.0000	-2.0541	0.0032
Cadm4	0.0000	-1.9833	0.0020
Cadps	0.0000	-1.6798	0.0066
Cadps2	0.0000	2.6007	0.0103
Calb1	0.0003	1.4610	0.0489
Calcoco2	0.0000	2.8959	0.0057
Calhm2	0.0000	2.5615	0.0379
Calml4	0.0000	3.5106	0.0087
Caly	0.0000	-1.5171	0.0291
Camk1d	0.0000	1.3881	0.0125
Camk2a	0.0000	-1.6329	0.0196
Camk2d	0.0000	-1.0091	0.0236
Camk2n1	0.0000	-1.3609	0.0054
Camk2n2	0.0000	-1.7948	0.0178
Camk4	0.0000	-1.4508	0.0337
Camta1	0.0000	-1.5891	0.0052
Capg	0.0000	4.1306	0.0093
Capn1	0.0000	1.7159	0.0088
Capn3	0.0000	3.6965	0.0256
Caps2	0.0000	4.5795	0.0161
Capsl	0.0000	2.6825	0.0188
Capza1	0.0000	2.1913	0.0014
Carnmt1	0.0000	2.1286	0.0030
Cars	0.0000	1.4595	0.0042
Cars2	0.0000	1.3980	0.0147
Casp12	0.0000	-3.6714	0.0391
Casp8	0.0000	2.2160	0.0237
Cast	0.0000	1.3903	0.0111
Catip	0.0000	4.0335	0.0263
Catsperd	0.0000	3.4157	0.0190
Cav2	0.0000	2.1194	0.0435
Cbarp	0.0000	-1.3059	0.0100
Cbln2	0.0000	-4.5380	0.0073
Cbln4	0.0000	-2.7194	0.0158
Cbr3	0.0000	1.8401	0.0360
Cbs	0.0000	1.9796	0.0166
Cbx7	0.0000	1.0745	0.0214
Cc2d1a	0.0000	1.0436	0.0204
Cc2d1b	0.0000	1.1354	0.0177
Cc2d2a	0.0000	1.2379	0.0181
Ccbe1	0.0000	-1.5630	0.0384
Ccdc113	0.0000	5.2344	0.0214
Ccdc114	0.0000	2.4060	0.0157
Ccdc125	0.0000	2.3538	0.0292
Ccdc136	0.0000	-1.3600	0.0132
Ccdc141	0.0000	2.6791	0.0130
Ccdc170	0.0000	2.8146	0.0451
Ccdc18	0.0000	1.3479	0.0220
Ccdc184	0.0000	-1.1127	0.0306

Ccdc22	0.0000	1.0294	0.0226
Ccdc24	0.0000	-2.2644	0.0340
Ccdc28b	0.0000	-2.4767	0.0043
Ccdc3	0.0000	-1.0886	0.0183
Ccdc36	0.0000	3.9757	0.0242
Ccdc39	0.0000	1.6946	0.0229
Ccdc60	0.0000	1.4846	0.0438
Ccdc65	0.0003	1.4125	0.0492
Ccdc68	0.0000	3.4614	0.0310
Ccdc86	0.0000	1.4919	0.0099
Cchcr1	0.0000	1.0280	0.0309
Cck	0.0000	6.1805	0.0134
Ccnb1	0.0000	1.8756	0.0038
Ccnb1ip1	0.0000	3.4726	0.0126
Ccnb2	0.0000	1.5594	0.0078
Ccne1	0.0000	1.2840	0.0123
Ccnf	0.0000	1.4239	0.0078
Ccng2	0.0000	-1.2308	0.0071
Ccnjl	0.0000	-1.0193	0.0320
Ccno	0.0000	4.0226	0.0211
Ccser1	0.0000	-1.3077	0.0261
Cd109	0.0000	-1.1355	0.0391
Cd248	0.0000	-3.6734	0.0028
Cd2ap	0.0000	1.3412	0.0069
Cd36	0.0000	-3.8466	0.0194
Cd38	0.0000	3.2838	0.0139
Cd44	0.0000	1.8398	0.0100
Cd47	0.0000	-1.8041	0.0034
Cd55	0.0000	2.2441	0.0089
Cd68	0.0000	2.5250	0.0248
Cd74	0.0000	4.2692	0.0327
Cd83	0.0000	-1.7922	0.0392
Cd84	0.0000	7.0224	0.0287
Cd9	0.0000	1.7080	0.0039
Cd93	0.0001	-1.5006	0.0470
Cdc14b	0.0000	1.2120	0.0180
Cdc20	0.0000	1.4245	0.0057
Cdc42ep1	0.0000	-1.3333	0.0325
Cdc42ep2	0.0000	-1.6905	0.0305
Cdc42ep3	0.0000	-1.4030	0.0198
Cdc42ep4	0.0000	-1.2897	0.0094
Cdc5l	0.0000	1.0363	0.0083
Cdc6	0.0000	1.5301	0.0134
Cdc73	0.0000	1.1137	0.0067
Cdca2	0.0000	1.4247	0.0073
Cdca3	0.0000	1.0737	0.0222
Cdca5	0.0000	1.5663	0.0098
Cdca7	0.0000	1.1064	0.0131
Cdcp1	0.0000	3.0754	0.0258

Cdh1	0.0000	3.6049	0.0004
Cdh10	0.0000	-2.0802	0.0119
Cdh11	0.0000	-1.5006	0.0248
Cdh13	0.0000	-1.6054	0.0105
Cdh18	0.0001	-1.3479	0.0464
Cdh20	0.0000	1.1696	0.0390
Cdh23	0.0002	-2.0670	0.0484
Cdh24	0.0000	-1.1535	0.0292
Cdh3	0.0000	2.9376	0.0051
Cdh5	0.0000	-3.9483	0.0037
Cdh6	0.0000	-1.9608	0.0037
Cdh7	0.0000	-2.7510	0.0256
Cdh8	0.0000	-2.6868	0.0125
Cdhr1	0.0000	2.4048	0.0198
Cdk1	0.0000	1.2934	0.0098
Cdk17	0.0000	-1.0045	0.0161
Cdk18	0.0000	1.8247	0.0368
Cdk5r1	0.0000	-1.9383	0.0048
Cdk5rap1	0.0000	1.2285	0.0227
Cdkl2	0.0003	1.2776	0.0491
Cdkn1c	0.0000	-1.6195	0.0027
Cdkn2a	0.0000	2.0049	0.0073
Cdon	0.0000	2.3116	0.0193
Cds1	0.0000	1.2381	0.0256
Cdx1	0.0000	5.5544	0.0148
Cdyl2	0.0000	2.6260	0.0068
Ceacam1	0.0000	4.0625	0.0199
Cebpb	0.0000	1.1113	0.0324
Celf3	0.0000	-1.8419	0.0051
Celf4	0.0000	-1.7524	0.0029
Celf5	0.0000	-1.2628	0.0104
Celf6	0.0000	-1.5035	0.0239
Celsr2	0.0000	-1.2471	0.0099
Celsr3	0.0000	-1.5318	0.0057
Cend1	0.0000	-2.1486	0.0111
Cenpn	0.0000	1.7666	0.0085
Cenpp	0.0000	1.6312	0.0201
Cenpw	0.0000	2.7491	0.0034
Cep112	0.0000	3.0120	0.0045
Cep135	0.0000	1.1003	0.0207
Cep170	0.0000	-1.1654	0.0065
Cep57l1	0.0000	1.0039	0.0283
Cep89	0.0000	1.3230	0.0122
Cerk	0.0000	-1.8173	0.0057
Cfap161	0.0000	4.5953	0.0425
Cfap206	0.0000	2.8772	0.0323
Cfap52	0.0000	3.4973	0.0156
Cfap53	0.0000	2.8872	0.0430
Cfap69	0.0000	1.1351	0.0432

Cfap70	0.0000	6.9072	0.0123
Cfh	0.0000	-2.0521	0.0091
Cfl2	0.0000	-1.0106	0.0090
Cgn	0.0000	1.9434	0.0089
Chac1	0.0000	2.3942	0.0083
Chaf1b	0.0000	1.3688	0.0172
Chd3	0.0000	-1.0851	0.0158
Chd5	0.0000	-1.0675	0.0290
Chek1	0.0000	1.5296	0.0089
Chek2	0.0000	1.6146	0.0150
Chga	0.0000	-1.2123	0.0140
Chgb	0.0000	-1.7034	0.0053
Chl1	0.0000	-2.3251	0.0053
Chn1	0.0000	-1.1172	0.0102
Chodl	0.0000	-1.9246	0.0397
Chrd	0.0000	-1.6093	0.0261
Chrm2	0.0000	-2.6567	0.0403
Chrna3	0.0000	-1.1539	0.0363
Chrna4	0.0000	-1.7225	0.0049
Chrna5	0.0000	-2.8386	0.0114
Chrnb2	0.0000	-1.7206	0.0046
Chrnb4	0.0000	-2.0486	0.0244
Chst11	0.0000	-2.0676	0.0056
Chst2	0.0000	-1.5003	0.0205
Chst3	0.0000	-2.1340	0.0092
Chsy3	0.0000	-1.7142	0.0281
Chtf18	0.0000	1.5307	0.0111
Cilp2	0.0000	1.5396	0.0303
Cirbp	0.0000	-1.2967	0.0092
Cit	0.0000	1.2009	0.0151
Ckb	0.0000	-1.1381	0.0070
Cks2	0.0000	1.6719	0.0059
Cldn1	0.0000	2.2324	0.0091
Cldn11	0.0000	-2.8307	0.0141
Cldn19	0.0000	4.4673	0.0304
Cldn2	0.0000	4.0881	0.0222
Cldn4	0.0000	2.3874	0.0044
Cldn9	0.0002	1.9909	0.0486
Clec2d	0.0001	-2.0533	0.0458
Clec2l	0.0000	-1.8161	0.0387
Clip2	0.0000	-1.5492	0.0038
Clip3	0.0000	-1.3401	0.0063
Clip4	0.0000	-1.0583	0.0323
Clmn	0.0000	1.6081	0.0101
Clmp	0.0000	-1.7185	0.0080
Cln5	0.0000	1.1712	0.0109
Clstn1	0.0000	-1.0120	0.0070
Clstn2	0.0000	-2.4728	0.0132
Clu	0.0000	2.8184	0.0017

Cmss1	0.0000	1.0048	0.0295
Cmtm6	0.0000	1.1072	0.0119
Cmtm7	0.0000	1.7791	0.0118
Cmtm8	0.0000	-2.5286	0.0092
Cnga4	0.0000	3.4230	0.0437
Cnksr2	0.0000	-1.5011	0.0249
Cnn1	0.0000	1.5222	0.0395
Cnnm1	0.0000	-2.1930	0.0294
Cnot1	0.0000	1.2777	0.0041
Cnpy1	0.0000	1.5674	0.0170
Cnr1	0.0000	-1.7135	0.0080
Cnrip1	0.0000	1.3884	0.0445
Cntd1	0.0000	2.3011	0.0247
Cntfr	0.0000	-1.2388	0.0080
Cntn4	0.0000	-2.1504	0.0425
Cntnap2	0.0000	1.6792	0.0091
Cntnap3	0.0001	1.6090	0.0468
Coa4	0.0000	-1.4998	0.0226
Cobl	0.0000	2.6311	0.0010
Coch	0.0000	2.3958	0.0243
Col11a1	0.0000	-2.4090	0.0033
Col12a1	0.0000	-4.4488	0.0108
Col13a1	0.0000	-2.4912	0.0213
Col16a1	0.0000	-1.9002	0.0185
Col17a1	0.0000	3.0072	0.0411
Col1a1	0.0000	-3.1526	0.0043
Col1a2	0.0000	-1.9636	0.0212
Col23a1	0.0000	-2.6482	0.0085
Col25a1	0.0001	-1.0812	0.0474
Col4a1	0.0000	-1.7735	0.0039
Col4a2	0.0000	-1.4586	0.0055
Col4a4	0.0000	2.5766	0.0446
Col5a2	0.0000	2.8355	0.0017
Col5a3	0.0000	4.0710	0.0156
Col6a1	0.0000	-1.9247	0.0047
Col6a2	0.0000	-1.5077	0.0115
Col6a3	0.0000	-5.5626	0.0005
Col6a5	0.0000	6.8866	0.0037
Col6a6	0.0000	-3.5015	0.0245
Col8a1	0.0000	-2.0516	0.0077
Col9a1	0.0000	-5.4337	0.0044
Col9a3	0.0001	1.1475	0.0467
Colec12	0.0000	2.1997	0.0237
Colgalt2	0.0000	-1.7584	0.0259
Coq3	0.0000	1.0579	0.0242
Corin	0.0000	-2.0664	0.0104
Coro2b	0.0000	-1.4522	0.0079
Coro6	0.0000	2.7983	0.0409
Cox19	0.0000	-1.1591	0.0125

Cox7a1	0.0000	2.4368	0.0271
Cox7a2	0.0000	-1.4500	0.0076
Cox7c	0.0000	1.9499	0.0015
Cp	0.0000	1.3341	0.0141
Cpa2	0.0000	2.3073	0.0375
Cpeb1	0.0000	1.4234	0.0180
Cpeb3	0.0000	-1.3195	0.0300
Cpeb4	0.0000	-1.4015	0.0080
Cped1	0.0000	-4.1030	0.0112
Cplx1	0.0000	-2.1840	0.0112
Cplx2	0.0000	-2.1810	0.0019
Cpm	0.0000	-2.1171	0.0159
Cpn1	0.0000	3.6356	0.0339
Cpne9	0.0000	3.3353	0.0340
Cpq	0.0000	3.8276	0.0067
Cpt1a	0.0000	2.0622	0.0033
Crabp1	0.0000	-1.6672	0.0068
Crabp2	0.0000	-1.3601	0.0387
Crb1	0.0000	-1.4771	0.0158
Creb5	0.0000	-1.0044	0.0412
Crhr1	0.0002	-2.8728	0.0479
Crim1	0.0000	-2.0220	0.0037
Crispld2	0.0000	-2.0186	0.0165
Crlf3	0.0000	1.8184	0.0053
Crmp1	0.0000	-1.9489	0.0011
Crtac1	0.0000	-2.0478	0.0074
Crym	0.0000	-1.9254	0.0345
Csdc2	0.0000	-1.9254	0.0059
Csf1r	0.0002	2.4649	0.0478
Csgalnact1	0.0000	-2.0077	0.0268
Csnk1e	0.0000	-1.4581	0.0035
Cspg5	0.0000	-1.7120	0.0066
Cspp1	0.0000	1.0488	0.0152
Csrnp1	0.0000	1.8423	0.0163
Csrnp3	0.0000	-1.1127	0.0126
Ctdspl	0.0000	-1.5946	0.0195
Cth	0.0000	3.7691	0.0010
Cthrc1	0.0000	-2.5731	0.0056
Ctif	0.0000	-1.3842	0.0083
Ctnnal1	0.0000	1.3994	0.0106
Ctnnbip1	0.0000	-1.2825	0.0117
Ctsc	0.0000	3.9589	0.0024
Ctsf	0.0000	-1.5285	0.0133
Cttnbp2	0.0000	-1.4044	0.0226
Cuedc1	0.0000	-1.6399	0.0052
Cul4b	0.0000	2.4817	0.0007
Cux2	0.0000	-1.6170	0.0096
Cwc22	0.0000	1.1320	0.0107
Cx3cl1	0.0000	3.8763	0.0009

Cxcl12	0.0000	-5.6669	0.0005
Cxcl14	0.0000	-2.2335	0.0244
Cxxc4	0.0000	-1.9514	0.0027
Cyb561d1	0.0000	-1.0480	0.0366
Cyb5r1	0.0000	2.1636	0.0021
Cyba	0.0000	2.0827	0.0135
Cycs	0.0000	1.2398	0.0075
Cyfip2	0.0000	-1.4080	0.0040
Cygb	0.0000	-3.3068	0.0122
Cyp1b1	0.0000	-5.6026	0.0011
Cyp27a1	0.0000	3.6828	0.0293
Cys1	0.0000	-4.0294	0.0373
Cyth1	0.0000	-1.4054	0.0123
Daam1	0.0000	-1.2229	0.0065
Daam2	0.0000	-2.4114	0.0098
Dab2	0.0000	-2.5913	0.0051
Dach2	0.0000	-1.8481	0.0206
Dact2	0.0000	3.1414	0.0040
Dact3	0.0000	-1.8202	0.0056
Dap	0.0000	-1.7660	0.0107
Dapp1	0.0000	3.1351	0.0242
Dbf4	0.0000	1.2265	0.0091
Dbndd1	0.0000	-1.4601	0.0189
Dbndd2	0.0000	-1.2814	0.0326
Dbx2	0.0000	-3.2917	0.0280
Dchs1	0.0000	-1.3191	0.0054
Dchs2	0.0000	-4.8852	0.0035
Dclk1	0.0000	-1.5262	0.0054
Dcn	0.0000	-5.0053	0.0005
Dctd	0.0000	1.1229	0.0239
Dcx	0.0000	-2.3637	0.0010
Ddb1	0.0000	1.0323	0.0060
Ddhd1	0.0000	-1.0731	0.0187
Ddn	0.0000	6.3330	0.0020
Ddr2	0.0000	-1.4358	0.0382
Ddx11	0.0000	1.3457	0.0166
Ddx58	0.0000	2.6130	0.0042
Ddx60	0.0000	4.0906	0.0320
Dennd1b	0.0000	1.0991	0.0147
Dennd2c	0.0000	2.5582	0.0024
Dennd2d	0.0001	2.9249	0.0465
Dennd5b	0.0000	-1.3527	0.0094
Depdc7	0.0000	1.8957	0.0205
Deptor	0.0000	2.2020	0.0100
Dexi	0.0000	-1.1997	0.0282
Dgka	0.0000	1.8832	0.0121
Dgkb	0.0000	-2.9559	0.0134
Dgke	0.0000	-1.2321	0.0188
Dhdh	0.0000	1.7711	0.0179

Dhrs3	0.0000	-3.4427	0.0259
Dhx58	0.0002	2.1440	0.0483
Diaph1	0.0000	1.3774	0.0043
Diras1	0.0000	-1.9664	0.0105
Disp2	0.0000	-1.3304	0.0125
Dixdc1	0.0000	-1.4418	0.0103
Dkc1	0.0000	1.7457	0.0024
Dkk1	0.0000	2.0539	0.0402
Dkk2	0.0001	1.7491	0.0476
Dkk3	0.0000	-1.2450	0.0150
Dlc1	0.0000	1.7521	0.0033
Dlg2	0.0000	-2.0477	0.0222
Dlg3	0.0000	-1.3100	0.0089
Dlgap5	0.0000	1.3544	0.0076
Dll1	0.0000	-1.0533	0.0183
Dll4	0.0000	-3.1227	0.0201
Dlx1	0.0001	-3.3211	0.0459
Dmbx1	0.0000	4.6799	0.0043
Dmc1	0.0000	2.7904	0.0263
Dmd	0.0000	1.0840	0.0165
Dmgdh	0.0000	5.3332	0.0153
Dmrta2	0.0000	1.2248	0.0304
Dnaaf1	0.0000	2.8104	0.0452
Dnaaf3	0.0000	1.3167	0.0406
Dnah1	0.0000	2.0350	0.0276
Dnah6	0.0000	4.8767	0.0187
Dnah8	0.0000	4.7606	0.0009
Dnah9	0.0000	-1.3992	0.0297
Dnajb5	0.0000	-1.3710	0.0088
Dnajc12	0.0000	-1.4051	0.0212
Dnajc19	0.0000	1.2197	0.0211
Dnajc21	0.0000	1.3343	0.0078
Dnajc22	0.0000	1.6453	0.0452
Dnajc28	0.0000	-1.5828	0.0303
Dnali1	0.0004	2.3726	0.0500
Dner	0.0000	-1.3105	0.0091
Dnm1	0.0000	-2.1102	0.0047
Dnm3	0.0000	-2.3057	0.0085
Dnmt1	0.0000	1.8732	0.0021
Dnmt3b	0.0000	2.2820	0.0032
Dnmt3l	0.0000	4.1683	0.0010
Doc2b	0.0000	-1.2098	0.0347
Dock10	0.0000	2.4252	0.0129
Dock2	0.0000	3.5981	0.0386
Dock4	0.0000	-1.3754	0.0103
Dock6	0.0000	1.1693	0.0093
Dock9	0.0000	1.7530	0.0081
Dok5	0.0000	-2.1640	0.0108
Dok6	0.0000	-1.3768	0.0322

Dpf1	0.0000	-1.5008	0.0097
Dpf3	0.0000	-1.5622	0.0225
Dph6	0.0000	1.0478	0.0216
Dpp10	0.0000	-1.6057	0.0257
Dpp4	0.0000	1.2785	0.0375
Dppa2	0.0000	3.5155	0.0036
Dppa4	0.0000	4.8702	0.0011
Dpy19l1	0.0000	1.2716	0.0081
Dpy19l3	0.0000	1.2891	0.0121
Dpy30	0.0000	1.0488	0.0128
Dpys	0.0000	5.2196	0.0156
Dpysl2	0.0000	-1.2684	0.0083
Dpysl3	0.0000	-1.3933	0.0026
Dpysl4	0.0000	-3.4644	0.0007
Dpysl5	0.0000	-1.5974	0.0022
Draxin	0.0000	-1.6279	0.0085
Drc1	0.0000	2.5969	0.0225
Drc3	0.0000	1.0103	0.0443
Drd1	0.0000	-4.6274	0.0223
Dscaml1	0.0001	-1.2575	0.0458
Dsg2	0.0000	3.2979	0.0019
Dsp	0.0000	1.1198	0.0195
Dtl	0.0000	1.2655	0.0087
Dtwd2	0.0000	1.1489	0.0372
Dtx1	0.0000	-1.2511	0.0143
Dtx3	0.0000	-1.2676	0.0067
Dtx3l	0.0000	2.5611	0.0135
Dtymk	0.0000	1.1273	0.0080
Dusp10	0.0000	-1.6039	0.0198
Dusp14	0.0000	2.0244	0.0138
Dusp26	0.0000	1.5737	0.0177
Dusp27	0.0000	2.4006	0.0047
Dusp4	0.0000	-1.5762	0.0047
Dusp5	0.0000	1.6130	0.0257
Dusp6	0.0000	1.0609	0.0159
Dusp8	0.0000	-1.7464	0.0028
Dynlrb2	0.0000	6.3792	0.0272
Dyrk2	0.0000	-1.0326	0.0118
Dyrk3	0.0000	1.7502	0.0066
Dzip1	0.0000	-1.2542	0.0088
E2f2	0.0000	-1.1491	0.0295
Ebf1	0.0000	-1.0792	0.0129
Ebf3	0.0000	-1.1643	0.0090
Ebf4	0.0000	-1.6962	0.0224
Ebp	0.0000	1.7255	0.0073
Echdc2	0.0000	2.1981	0.0089
Echdc3	0.0000	3.0457	0.0396
Ecm1	0.0000	-1.0668	0.0441
Ecm2	0.0000	4.2318	0.0072

Ect2	0.0000	1.4631	0.0071
Eda	0.0000	-1.5220	0.0223
Eda2r	0.0000	-1.0469	0.0163
Edn1	0.0001	1.6700	0.0470
Ednra	0.0000	-3.5312	0.0040
Ednrb	0.0000	-1.3486	0.0080
Eed	0.0000	1.4188	0.0049
Eef1a2	0.0000	-1.7946	0.0150
Eef1g	0.0000	1.6999	0.0017
Eef2k	0.0000	-1.4044	0.0097
Efcab10	0.0000	4.3771	0.0367
Efcab11	0.0000	1.6710	0.0238
Efcab12	0.0000	3.0058	0.0330
Efcab6	0.0000	4.6064	0.0444
Efcc1	0.0000	-1.2868	0.0378
Efemp1	0.0000	-1.7343	0.0250
Efemp2	0.0000	-2.4776	0.0053
Efhc1	0.0000	1.9313	0.0230
Efhc2	0.0000	4.1510	0.0104
Efhd2	0.0000	-1.6624	0.0065
Efna3	0.0000	-1.5251	0.0128
Efna5	0.0000	1.4515	0.0124
Efnb1	0.0000	-1.2829	0.0081
Efnb3	0.0000	-1.8472	0.0020
Efr3a	0.0000	1.0287	0.0181
Efr3b	0.0000	-1.3549	0.0087
Egfl7	0.0000	1.0667	0.0293
Egflam	0.0000	-3.3667	0.0034
Egfr	0.0000	-2.2220	0.0085
Ehd2	0.0000	-1.8693	0.0163
Ehd3	0.0000	-1.0246	0.0296
Ehd4	0.0000	1.0322	0.0198
Eif1b	0.0000	-1.0180	0.0118
Eif2d	0.0000	1.5360	0.0054
Eif2s2	0.0000	1.6527	0.0014
Eif4ebp1	0.0000	2.7938	0.0007
Elavl2	0.0000	-1.5332	0.0049
Elavl3	0.0000	-1.9396	0.0019
Elavl4	0.0000	-1.9116	0.0018
Elf4	0.0000	2.0214	0.0136
Elfn1	0.0000	-1.1649	0.0365
Elfn2	0.0000	-1.9601	0.0137
Elk3	0.0000	-1.0780	0.0291
Ell2	0.0000	1.4199	0.0121
Ell3	0.0000	2.9959	0.0207
Elmo3	0.0000	2.1235	0.0373
Elmod1	0.0000	-3.7585	0.0064
Elovl4	0.0000	-1.4411	0.0114
Elovl7	0.0000	3.1299	0.0094

Emb	0.0000	2.5696	0.0009
Emc10	0.0000	-1.5603	0.0039
Emilin1	0.0000	-1.2862	0.0146
Emilin3	0.0000	-2.1563	0.0290
Eml1	0.0000	-1.0483	0.0152
Emp1	0.0000	-1.5325	0.0081
Endov	0.0000	-1.2282	0.0123
Eng	0.0000	3.6725	0.0039
Enho	0.0000	-1.6104	0.0088
Enkd1	0.0000	1.0849	0.0283
Enkur	0.0000	2.1917	0.0347
Eno1	0.0000	3.5792	0.0002
Eno2	0.0000	-1.2088	0.0132
Eno4	0.0000	2.5370	0.0426
Enpep	0.0000	-4.5619	0.0111
Enpp1	0.0000	-4.1724	0.0031
Entpd1	0.0000	-2.5704	0.0255
Eomes	0.0000	3.6046	0.0101
Epb41l1	0.0000	-1.0900	0.0107
Epha10	0.0000	-1.5307	0.0194
Epha2	0.0000	2.0575	0.0027
Epha3	0.0000	-1.4350	0.0090
Epha5	0.0000	-2.9362	0.0025
Epha8	0.0000	-3.4288	0.0075
Ephb1	0.0000	-2.4005	0.0024
Ephb2	0.0000	-1.6076	0.0032
Ephx2	0.0000	4.6874	0.0006
Epm2a	0.0000	-1.3040	0.0253
Epm2aip1	0.0000	-1.4164	0.0074
Epn3	0.0000	1.9772	0.0423
Erbb3	0.0000	2.3807	0.0123
Erbb4	0.0000	-1.6071	0.0117
Erc2	0.0000	-1.2114	0.0150
Errfi1	0.0000	-1.1858	0.0140
Espl1	0.0000	1.4504	0.0067
Esrrb	0.0000	3.4713	0.0005
Esrrg	0.0000	-2.4006	0.0065
Esyt3	0.0000	2.4762	0.0276
Etfa	0.0000	1.0645	0.0108
Ets1	0.0000	-2.1700	0.0100
Etv1	0.0000	1.6906	0.0109
Etv4	0.0000	3.5939	0.0025
Etv5	0.0000	3.3722	0.0004
Eva1c	0.0000	-3.0900	0.0319
Evc2	0.0000	1.2821	0.0209
Evl	0.0000	-1.0229	0.0097
Exosc5	0.0000	1.5152	0.0088
Exosc8	0.0000	1.0146	0.0184
Eya2	0.0000	-1.3384	0.0307

Ezr	0.0000	2.0862	0.0012
F2r	0.0000	-1.4620	0.0041
F2rl1	0.0000	2.2958	0.0181
F3	0.0000	1.1829	0.0297
Fabp3	0.0000	1.7239	0.0067
Fads6	0.0000	2.0825	0.0298
Fah	0.0000	1.9840	0.0165
Faim2	0.0000	-2.1383	0.0268
Fam102b	0.0000	-1.1509	0.0156
Fam107b	0.0000	-1.0273	0.0145
Fam111a	0.0000	1.4338	0.0117
Fam117a	0.0000	1.2320	0.0213
Fam124a	0.0000	-1.4622	0.0242
Fam129a	0.0000	1.1379	0.0359
Fam131a	0.0000	-1.3540	0.0202
Fam131b	0.0000	-1.3363	0.0162
Fam131c	0.0000	-1.8002	0.0362
Fam135b	0.0000	-1.7712	0.0369
Fam149a	0.0000	1.2852	0.0303
Fam155a	0.0000	-1.6967	0.0142
Fam160a1	0.0000	1.7337	0.0383
Fam161a	0.0000	1.3131	0.0311
Fam163a	0.0000	2.8576	0.0075
Fam169a	0.0000	1.0071	0.0155
Fam169b	0.0000	3.8634	0.0176
Fam171a1	0.0000	-1.2959	0.0075
Fam171a2	0.0000	-1.2764	0.0081
Fam171b	0.0000	-1.3965	0.0052
Fam178b	0.0000	5.0438	0.0074
Fam189a1	0.0000	-1.4897	0.0193
Fam189a2	0.0000	-1.4661	0.0302
Fam20a	0.0000	-4.5407	0.0403
Fam20c	0.0000	-1.7616	0.0072
Fam210b	0.0000	-2.9199	0.0019
Fam217b	0.0000	-1.5394	0.0254
Fam219a	0.0000	-1.5117	0.0068
Fam220a	0.0000	-1.1105	0.0145
Fam222a	0.0000	-1.1391	0.0301
Fam227a	0.0001	-1.1196	0.0455
Fam234b	0.0000	-1.8330	0.0049
Fam32a	0.0000	-1.1572	0.0095
Fam57b	0.0000	-2.6133	0.0036
Fam71e1	0.0000	-1.9247	0.0445
Fam72a	0.0000	1.3613	0.0343
Fam78a	0.0000	1.7844	0.0310
Fam78b	0.0000	-1.2563	0.0199
Fam81a	0.0000	2.4134	0.0055
Fam81b	0.0002	3.9269	0.0486
Fam83d	0.0000	1.2704	0.0159

Fam83g	0.0000	1.8016	0.0297
Fam89b	0.0000	-1.6794	0.0154
Fanca	0.0000	2.1526	0.0055
Fancd2	0.0000	1.9273	0.0060
Fanci	0.0000	2.1192	0.0052
Fancl	0.0000	1.6350	0.0098
Fancm	0.0000	1.1337	0.0137
Farsb	0.0000	1.3332	0.0062
Fastkd2	0.0000	1.2813	0.0113
Fat3	0.0000	-1.4340	0.0067
Fat4	0.0000	-2.7577	0.0014
Faxc	0.0000	-1.6148	0.0046
Fbll1	0.0000	-1.6500	0.0246
Fbln1	0.0000	-1.9768	0.0022
Fbln2	0.0000	-1.1793	0.0276
Fbln5	0.0000	-1.9567	0.0230
Fbln7	0.0000	2.1724	0.0362
Fbn2	0.0000	-1.8564	0.0244
Fbxl16	0.0000	-1.2364	0.0126
Fbxl18	0.0000	-1.3470	0.0088
Fbxl20	0.0000	-1.0804	0.0102
Fbxl7	0.0000	-2.1626	0.0110
Fbxo21	0.0000	-1.1312	0.0066
Fbxo27	0.0000	1.8389	0.0311
Fbxo32	0.0000	-1.6526	0.0167
Fbxo41	0.0000	-1.2442	0.0285
Fbxo44	0.0000	-1.6918	0.0246
Fbxo5	0.0000	1.4604	0.0083
Fcf1	0.0000	1.7333	0.0072
Fcgrt	0.0000	-1.2262	0.0302
Fen1	0.0000	1.2405	0.0114
Fes	0.0001	2.2144	0.0461
Fez1	0.0000	-1.8896	0.0018
Fgd3	0.0000	-1.7553	0.0237
Fgd6	0.0000	-1.6689	0.0164
Fgf1	0.0000	1.2557	0.0384
Fgf11	0.0000	-1.4041	0.0151
Fgf12	0.0000	-2.3679	0.0134
Fgf13	0.0000	-1.9268	0.0076
Fgf17	0.0000	3.9374	0.0189
Fgf2	0.0000	-4.3795	0.0227
Fgf9	0.0001	-1.5989	0.0463
Fgfr3	0.0000	-3.1390	0.0013
Fgfrl1	0.0000	-1.7151	0.0086
Fgl2	0.0000	-1.7785	0.0282
Fhl1	0.0000	-1.7526	0.0031
Fhl2	0.0000	-2.1753	0.0177
Fhod1	0.0000	1.1591	0.0206
Fibcd1	0.0000	-2.0300	0.0166

Fibin	0.0000	-3.0076	0.0163
Fign	0.0000	-1.3375	0.0139
Fignl1	0.0000	1.1225	0.0176
Filip1	0.0000	-1.1245	0.0309
Filip1l	0.0000	-1.8687	0.0180
Fkbp10	0.0000	-1.3936	0.0144
Fkrp	0.0000	-1.2762	0.0138
Fli1	0.0000	-3.0329	0.0401
Flrt1	0.0000	-2.1926	0.0181
Flrt2	0.0000	-3.3435	0.0024
Flt4	0.0000	2.2108	0.0271
Flywch1	0.0000	-1.2291	0.0077
Fmn1	0.0000	2.5248	0.0212
Fmn2	0.0000	-1.4860	0.0092
Fmo4	0.0003	3.4166	0.0495
Fn3k	0.0001	-1.5047	0.0476
Fndc4	0.0000	-1.1869	0.0152
Fndc5	0.0000	-4.2946	0.0055
Focad	0.0000	1.9999	0.0031
Folr1	0.0000	4.0827	0.0020
Foxc1	0.0000	-5.8417	0.0041
Foxc2	0.0000	-5.1903	0.0065
Foxf2	0.0000	-4.9690	0.0121
Foxh1	0.0000	3.7413	0.0041
Foxj1	0.0000	1.3368	0.0143
Foxl2	0.0000	-1.9718	0.0285
Foxn3	0.0000	-1.4923	0.0073
Foxo6	0.0000	-1.3742	0.0343
Foxp1	0.0000	-1.6533	0.0081
Foxp2	0.0000	-2.4034	0.0242
Foxs1	0.0000	-4.1996	0.0423
Fpgs	0.0000	1.5462	0.0101
Frat1	0.0000	-1.8555	0.0216
Frem1	0.0000	-1.9272	0.0124
Frk	0.0000	4.2649	0.0105
Frmd4b	0.0000	-2.1904	0.0016
Frmpd3	0.0000	-2.1478	0.0117
Frrs1	0.0000	3.9720	0.0011
Frrs1l	0.0000	-2.4272	0.0115
Frs3	0.0000	-1.2984	0.0250
Frzb	0.0000	-2.8505	0.0053
Fstl5	0.0000	-3.0701	0.0072
Ftsj3	0.0000	1.4118	0.0060
Fxyd1	0.0000	-1.5666	0.0450
Fxyd6	0.0000	-1.3209	0.0051
Fxyd7	0.0000	-2.2226	0.0425
Fyb	0.0000	-4.8237	0.0314
Fyn	0.0000	-1.2325	0.0069
Fzd10	0.0000	2.3190	0.0013

Fzd2	0.0000	-1.7168	0.0026
Fzd5	0.0000	2.6479	0.0025
Fzd6	0.0000	1.1982	0.0274
G0s2	0.0000	1.4913	0.0261
Gab2	0.0000	-1.9701	0.0040
Gabarapl1	0.0000	-1.2495	0.0060
Gabbr1	0.0000	-1.4848	0.0047
Gabbr2	0.0000	-1.3362	0.0354
Gabrb2	0.0000	-1.6276	0.0213
Gabrb3	0.0000	-1.1849	0.0179
Gabrg1	0.0000	1.6451	0.0450
Gad1	0.0000	-1.5644	0.0253
Gad2	0.0000	-2.5570	0.0061
Gadd45g	0.0000	-1.1744	0.0155
Galm	0.0000	2.0741	0.0125
Galnt12	0.0000	4.0990	0.0036
Galnt16	0.0000	-1.1526	0.0127
Galnt18	0.0000	-2.7953	0.0099
Galnt3	0.0000	2.9301	0.0232
Galnt4	0.0000	1.4547	0.0210
Galnt6	0.0000	1.9300	0.0260
Galnt9	0.0000	-1.0095	0.0377
Galt	0.0000	1.4189	0.0238
Gap43	0.0000	-1.7344	0.0019
Gars	0.0000	1.1683	0.0053
Gart	0.0000	1.6362	0.0018
Gas1	0.0000	1.3950	0.0061
Gas5	0.0000	-2.6810	0.0129
Gas7	0.0000	-1.9178	0.0127
Gata2	0.0000	-2.1882	0.0152
Gata3	0.0000	-3.4002	0.0051
Gatm	0.0000	1.9394	0.0318
Gba2	0.0000	-1.0100	0.0175
Gbp3	0.0000	2.8822	0.0370
Gbp4	0.0000	-2.0039	0.0354
Gca	0.0000	1.2922	0.0278
Gcat	0.0000	1.7102	0.0056
Gch1	0.0000	-1.1676	0.0411
Gclm	0.0000	1.0444	0.0175
Gcnt1	0.0000	1.0460	0.0408
Gdap1	0.0000	-1.4010	0.0108
Gdap1l1	0.0000	-2.6826	0.0026
Gdf10	0.0000	-3.0326	0.0280
Gdf6	0.0000	-2.7011	0.0359
Gdf7	0.0000	7.9776	0.0074
Gdi1	0.0000	-1.3175	0.0041
Gdpd2	0.0000	-1.7312	0.0159
Gdpd5	0.0000	-1.5739	0.0083
Gem	0.0000	1.1660	0.0444

Gfm1	0.0000	1.0572	0.0142
Gfod1	0.0000	-1.4063	0.0264
Gfod2	0.0000	-1.0359	0.0165
Gfpt2	0.0000	1.6870	0.0120
Gfra1	0.0000	-1.8488	0.0029
Ggt1	0.0003	1.7902	0.0496
Ggt5	0.0000	-3.2208	0.0266
Ghr	0.0000	-1.3658	0.0167
Gins1	0.0000	1.2429	0.0182
Gipr	0.0000	-1.8779	0.0270
Gjb5	0.0000	3.0813	0.0086
Gjd2	0.0000	-1.2782	0.0369
Gk	0.0000	1.4423	0.0157
Gldc	0.0000	1.0020	0.0224
Glipr1	0.0000	2.6348	0.0225
Glis3	0.0000	3.7940	0.0014
Glra1	0.0000	-3.5295	0.0213
Glra2	0.0000	-2.6443	0.0229
Glrx3	0.0000	1.4126	0.0037
Gls2	0.0000	2.6973	0.0098
Glt1d1	0.0000	1.9779	0.0113
Glt8d2	0.0000	-2.9661	0.0327
Gmnc	0.0000	7.8309	0.0142
Gmnn	0.0000	1.5758	0.0094
Gna15	0.0000	5.0791	0.0133
Gnal	0.0000	-1.7713	0.0092
Gnaq	0.0000	-1.2663	0.0061
Gnaz	0.0000	-1.1046	0.0250
Gnb5	0.0000	-1.2082	0.0159
Gng10	0.0000	-1.8809	0.0071
Gng11	0.0000	-2.0188	0.0272
Gng2	0.0000	-1.5061	0.0028
Gng3	0.0000	-1.3945	0.0091
Gng4	0.0000	-1.5568	0.0326
Gng7	0.0000	-1.9528	0.0165
Gnl3	0.0000	1.7125	0.0023
Gnpda1	0.0000	1.7240	0.0042
Gnpnat1	0.0000	1.5180	0.0086
Golga7b	0.0000	-2.9056	0.0088
Gpat2	0.0000	4.0671	0.0028
Gpc2	0.0000	-1.7199	0.0027
Gpn2	0.0000	-1.2188	0.0295
Gpr137	0.0000	-1.0316	0.0257
Gpr137c	0.0000	-1.5043	0.0181
Gpr153	0.0000	-2.4515	0.0044
Gpr155	0.0000	-1.2254	0.0408
Gpr160	0.0000	4.2224	0.0228
Gpr161	0.0000	-1.0427	0.0154
Gpr162	0.0000	-1.4494	0.0152

Gpr173	0.0000	-1.4380	0.0132
Gpr21	0.0002	-2.3823	0.0480
Gpr22	0.0000	-1.3731	0.0291
Gpr26	0.0000	2.1773	0.0438
Gpr37	0.0000	-4.5565	0.0077
Gpr83	0.0000	3.1924	0.0366
Gprin1	0.0000	-1.5404	0.0080
Gps1	0.0000	1.2271	0.0084
Gpsm1	0.0000	-1.6300	0.0038
Gpsm3	0.0001	1.7489	0.0459
Gpx4	0.0000	2.4214	0.0013
Gpx7	0.0000	1.8319	0.0186
Gramd1a	0.0000	-1.6253	0.0062
Gramd1c	0.0000	2.5058	0.0181
Gramd4	0.0000	-1.5882	0.0086
Grasp	0.0000	-1.1483	0.0294
Grb10	0.0000	-3.1926	0.0006
Grb7	0.0000	3.2393	0.0083
Greb1	0.0000	-1.0284	0.0412
Grhl1	0.0002	1.4940	0.0487
Grhl3	0.0000	3.2283	0.0057
Gria2	0.0000	-2.2239	0.0070
Gria4	0.0000	-1.4979	0.0172
Grik2	0.0000	-1.7629	0.0228
Grik3	0.0000	-1.5463	0.0039
Grik4	0.0000	-2.5092	0.0150
Grik5	0.0000	-2.1812	0.0027
Grin2b	0.0000	-2.6959	0.0091
Grin2d	0.0000	-1.7548	0.0256
Grin3a	0.0000	-2.4048	0.0182
Grip2	0.0000	-2.0157	0.0310
Grk5	0.0000	-1.2561	0.0195
Grm3	0.0000	-2.2316	0.0393
Grm4	0.0000	-1.0418	0.0369
Grm5	0.0001	-1.4580	0.0458
Grm7	0.0000	-2.8257	0.0395
Grtp1	0.0000	2.0467	0.0091
Gsdmd	0.0000	3.2068	0.0278
Gsg1l	0.0000	-1.0195	0.0325
Gsk3b	0.0000	-1.1916	0.0051
Gss	0.0000	1.1518	0.0181
Gsta4	0.0000	1.6565	0.0031
Gstm1	0.0000	1.8903	0.0050
Gstm2	0.0000	2.3178	0.0092
Gstm3	0.0000	4.9940	0.0146
Gstm4	0.0000	1.5252	0.0248
Gstp1	0.0000	1.7061	0.0034
Gstt1	0.0000	2.7971	0.0298
Gstt2	0.0000	2.2251	0.0202

Gsx1	0.0000	-1.3921	0.0434
Gsx2	0.0000	-3.2187	0.0450
Gtf2ird2	0.0000	-1.0025	0.0355
Habp4	0.0000	-1.3988	0.0130
Hacd2	0.0000	1.1302	0.0193
Hadhb	0.0000	2.2311	0.0040
Haghl	0.0000	-1.5865	0.0138
Hapln1	0.0000	-3.4111	0.0137
Has2	0.0000	-2.3197	0.0168
Haspin	0.0000	1.3589	0.0196
Hat1	0.0000	1.7587	0.0019
Haus2	0.0000	-1.2519	0.0112
Haus4	0.0000	1.2707	0.0213
Haus5	0.0000	1.4780	0.0185
Hax1	0.0000	1.2791	0.0112
Hck	0.0000	1.5342	0.0240
Hcn3	0.0000	-1.6616	0.0197
Hcn4	0.0000	-1.0056	0.0233
Hdac1	0.0000	2.7591	0.0009
Hdac4	0.0000	1.1412	0.0142
Hdac5	0.0000	-1.1307	0.0083
Hdac9	0.0000	-1.0380	0.0279
Hdc	0.0000	4.1173	0.0382
Hddc3	0.0000	-1.5777	0.0132
Hdgfl3	0.0000	-1.2518	0.0058
Hectd2	0.0000	-1.0685	0.0299
Heg1	0.0000	-1.2716	0.0124
Helb	0.0000	1.4919	0.0125
Hells	0.0000	2.0563	0.0019
Helz2	0.0000	2.7737	0.0176
Hepacam	0.0003	1.2425	0.0495
Herc4	0.0000	1.1873	0.0082
Herpud1	0.0000	1.2403	0.0102
Hexb	0.0000	1.8142	0.0030
Hey2	0.0000	-2.8514	0.0188
Heyl	0.0000	-5.1153	0.0141
Hfe	0.0003	2.6244	0.0494
Hhat	0.0000	1.5614	0.0400
Hhex	0.0000	2.1173	0.0349
Hhip	0.0000	-1.5899	0.0171
Hic1	0.0000	-5.4542	0.0026
Hip1	0.0000	-1.0042	0.0098
Hivep2	0.0000	-1.4758	0.0116
Hivep3	0.0000	-2.1756	0.0359
Hlf	0.0000	-1.5865	0.0211
Hlx	0.0002	-2.0492	0.0483
Hmces	0.0000	1.9937	0.0051
Hmcn1	0.0000	-2.6918	0.0070
Hmcn2	0.0000	2.1270	0.0367

Hmga1	0.0000	3.1521	0.0005
Hmgb2	0.0000	2.7085	0.0009
Hmgcs1	0.0000	-1.5595	0.0013
Hmgn2	0.0000	2.0236	0.0020
Hmox1	0.0000	2.4926	0.0024
Hnmt	0.0000	3.8560	0.0398
Hnrnpa1	0.0000	1.4019	0.0032
Hnrnpa3	0.0000	1.9962	0.0007
Hook1	0.0000	1.5852	0.0043
Hook2	0.0000	1.6131	0.0124
Hopx	0.0000	-2.0191	0.0240
Hormad1	0.0000	4.7809	0.0105
Hoxd1	0.0002	-3.2976	0.0480
Hpca	0.0000	-4.0406	0.0052
Hpcal1	0.0000	-1.3167	0.0153
Hpcal4	0.0000	-1.6567	0.0342
Hpgd	0.0001	1.3489	0.0455
Hpgds	0.0000	1.8764	0.0370
Hrc	0.0000	-2.6796	0.0142
Hrh3	0.0000	-2.1406	0.0308
Hs3st3a1	0.0000	-2.7099	0.0259
Hs3st3b1	0.0000	-1.4935	0.0191
Hs3st5	0.0000	-1.7850	0.0328
Hsd11b2	0.0000	1.4691	0.0244
Hsd17b11	0.0000	1.3245	0.0151
Hsd17b14	0.0000	4.7238	0.0081
Hsp90aa1	0.0000	2.0702	0.0005
Hspa12a	0.0000	-1.7825	0.0031
Hspa12b	0.0000	-1.4483	0.0330
Hspa14	0.0000	1.3380	0.0052
Hspa1b	0.0000	1.7185	0.0153
Hspa9	0.0000	1.1535	0.0047
Hspb1	0.0000	3.5402	0.0006
Hspb8	0.0000	2.3146	0.0027
Hspbap1	0.0000	2.0696	0.0056
Hspd1	0.0000	1.7449	0.0011
Hspe1	0.0000	1.9849	0.0009
Htr1b	0.0001	1.4546	0.0461
Htr5a	0.0000	3.7337	0.0344
Hydin	0.0000	4.0451	0.0114
Hyi	0.0000	-1.7044	0.0290
Hypk	0.0000	-4.3955	0.0017
Iars	0.0000	1.4823	0.0023
Ica1l	0.0000	-1.4842	0.0421
Id4	0.0000	-3.0051	0.0008
Idnk	0.0000	-1.3016	0.0263
Ids	0.0000	-1.2273	0.0113
Ier5l	0.0000	-1.3021	0.0104
Ifi30	0.0000	1.5832	0.0097

Ifi35	0.0000	2.3871	0.0246
Ifi44	0.0000	3.2231	0.0269
Ifih1	0.0000	3.3083	0.0210
Ifit1	0.0000	3.4914	0.0284
Ifit3	0.0000	1.5864	0.0429
Ifitm1	0.0000	3.1206	0.0014
Ifitm3	0.0000	2.3701	0.0046
Ifnlr1	0.0000	1.8786	0.0405
Igdcc4	0.0000	-1.2088	0.0091
Igf1	0.0000	-2.1353	0.0422
Igf1r	0.0000	-1.5050	0.0032
Igf2	0.0000	-2.8593	0.0009
Igf2r	0.0000	-1.0116	0.0115
Igfbp3	0.0000	-2.0356	0.0107
Igfbp4	0.0000	-2.8697	0.0008
Igfbp5	0.0000	-2.2596	0.0101
Igfbp7	0.0000	-3.1150	0.0082
Iglon5	0.0000	-2.0391	0.0041
Igsf11	0.0000	-4.2223	0.0031
Igsf21	0.0000	-1.1203	0.0269
Igsf23	0.0000	3.3545	0.0294
Il17rc	0.0000	2.4142	0.0286
Il1r1	0.0000	2.7232	0.0248
Il1rap	0.0000	1.5506	0.0167
Il1rapl2	0.0000	-4.6420	0.0383
Il34	0.0001	2.0177	0.0471
Impa2	0.0000	2.8578	0.0016
Impact	0.0000	1.0699	0.0070
Ina	0.0000	-1.7110	0.0028
Inafm2	0.0000	-1.8761	0.0069
Inava	0.0000	2.0219	0.0104
Ing5	0.0000	1.5277	0.0067
Inha	0.0000	-1.3964	0.0395
Inhba	0.0000	-3.7188	0.0268
Inpp5d	0.0000	3.2050	0.0037
Inpp5j	0.0000	-3.6836	0.0339
Insm2	0.0000	-3.8680	0.0422
Iqcc	0.0000	-1.4804	0.0186
Iqcg	0.0000	1.1854	0.0216
Iqgap1	0.0000	1.6273	0.0022
Iqsec3	0.0000	-2.9077	0.0035
Iqub	0.0000	4.5103	0.0196
Irak2	0.0000	1.1692	0.0336
Irak3	0.0000	2.6731	0.0141
Irf1	0.0000	2.0421	0.0094
Irf2bp1	0.0000	-1.0702	0.0118
Irf6	0.0000	3.1031	0.0107
Irf7	0.0000	3.5858	0.0306
Irgq	0.0000	-1.1004	0.0122

Isg15	0.0000	3.9420	0.0169
Islr	0.0000	-6.5190	0.0006
Ism1	0.0000	-1.3954	0.0436
Ism2	0.0000	2.8168	0.0187
Isyna1	0.0000	1.0949	0.0101
Itga11	0.0000	-2.3697	0.0287
Itga8	0.0000	-3.3615	0.0053
Itga9	0.0000	-2.1500	0.0110
Itgae	0.0000	3.4399	0.0431
Itgal	0.0000	3.6321	0.0368
Itgam	0.0000	3.4563	0.0264
Itgb1	0.0000	1.0249	0.0116
Itgb7	0.0000	4.2686	0.0184
Itgb8	0.0000	-1.8264	0.0057
Itih2	0.0000	-6.6105	0.0137
Itih5	0.0000	-3.8762	0.0013
Itm2a	0.0000	-1.7527	0.0073
Itpa	0.0000	1.3557	0.0086
Itpk1	0.0000	1.0560	0.0158
Itpka	0.0000	2.4811	0.0191
Itpr1	0.0000	1.3440	0.0086
Itpr2	0.0000	-2.1525	0.0167
Itsn1	0.0000	-1.2196	0.0050
Itsn2	0.0000	1.1557	0.0141
Jade1	0.0000	2.1210	0.0014
Jade2	0.0000	2.5418	0.0093
Jade3	0.0000	1.8854	0.0026
Jag1	0.0000	-1.1580	0.0138
Jak3	0.0000	2.1872	0.0057
Jakmip1	0.0000	2.1242	0.0094
Jazf1	0.0000	-1.2155	0.0349
Jmjd8	0.0000	-1.0624	0.0238
Jph1	0.0000	1.7110	0.0199
Jph3	0.0000	-1.9121	0.0074
Jph4	0.0000	-1.6340	0.0148
Jrk	0.0000	-1.0752	0.0231
Jun	0.0000	-1.7062	0.0029
Kalrn	0.0000	-1.0219	0.0127
Kank3	0.0000	2.3308	0.0046
Kat2b	0.0000	1.2030	0.0108
Katnal1	0.0000	-1.1615	0.0127
Kazald1	0.0000	-2.4709	0.0251
Kbtbd11	0.0000	-2.1793	0.0014
Kcnab2	0.0000	-2.0820	0.0111
Kcnb1	0.0000	-1.4914	0.0163
Kcnb2	0.0000	-1.4423	0.0355
Kcnc1	0.0000	-2.7780	0.0038
Kcnd2	0.0000	-2.6406	0.0249
Kcnd3	0.0000	-1.0007	0.0233

Kcne4	0.0000	-4.1098	0.0176
Kcnh3	0.0000	2.4468	0.0295
Kcnh7	0.0000	-1.7259	0.0310
Kcnip1	0.0000	-1.5970	0.0317
Kcnip2	0.0004	1.0030	0.0497
Kcnip4	0.0000	-3.4687	0.0203
Kcnj11	0.0000	-2.5646	0.0272
Kcnj3	0.0002	1.0964	0.0481
Kcnj5	0.0000	-4.1323	0.0411
Kcnj8	0.0000	-5.3820	0.0240
Kcnk1	0.0000	1.9780	0.0118
Kcnk10	0.0000	-1.2442	0.0285
Kcnk3	0.0000	-2.6283	0.0221
Kcnma1	0.0000	-1.2228	0.0320
Kcnmb2	0.0000	-1.8246	0.0350
Kcnmb4	0.0000	-1.5989	0.0251
Kcnn1	0.0000	-1.5903	0.0109
Kcnn3	0.0000	-1.1075	0.0329
Kcnq2	0.0000	-1.1868	0.0123
Kcnq3	0.0000	-1.1014	0.0303
Kcns3	0.0000	1.7232	0.0330
Kcp	0.0000	-2.4318	0.0287
Kctd1	0.0000	-1.0607	0.0242
Kctd13	0.0000	-1.0522	0.0211
Kctd4	0.0000	1.6170	0.0439
Kctd7	0.0000	-1.3906	0.0232
Kdf1	0.0000	2.3229	0.0363
Kdm6a	0.0000	1.4524	0.0049
Khdrbs2	0.0000	1.0556	0.0375
Khk	0.0001	-1.2171	0.0461
Kidins220	0.0000	-1.0556	0.0080
Kif11	0.0000	1.1649	0.0123
Kif14	0.0000	1.5037	0.0090
Kif15	0.0000	1.0708	0.0139
Kif18b	0.0000	1.1477	0.0196
Kif1b	0.0000	-1.1715	0.0038
Kif20a	0.0000	1.3782	0.0063
Kif22	0.0000	1.1687	0.0167
Kif2c	0.0000	1.2593	0.0143
Kif3c	0.0000	-1.2781	0.0048
Kif5a	0.0000	-1.5733	0.0043
Kif5c	0.0000	-2.0231	0.0014
Kif9	0.0000	1.6700	0.0354
Kifc1	0.0000	1.2082	0.0100
Kifc2	0.0000	-1.2606	0.0306
Kirrel2	0.0000	2.5386	0.0073
Kirrel3	0.0000	-1.8555	0.0134
Kiss1r	0.0000	-2.6467	0.0332
Klc1	0.0000	-1.3631	0.0041

Klc3	0.0000	2.2502	0.0303
Klf15	0.0000	1.4517	0.0226
Klf2	0.0000	4.4947	0.0004
Klf4	0.0000	1.6448	0.0106
Klf5	0.0000	1.8021	0.0247
Klf8	0.0000	2.1434	0.0098
Klf9	0.0000	2.3122	0.0021
Klhdc8a	0.0000	-1.4182	0.0303
Klhl1	0.0000	-1.5644	0.0354
Klhl14	0.0000	-2.3469	0.0234
Klhl3	0.0000	-1.4327	0.0226
Klhl32	0.0000	-1.2146	0.0296
Klhl5	0.0000	-1.3701	0.0118
Kmt2e	0.0000	-1.1544	0.0082
Kndc1	0.0000	-1.2122	0.0267
Knop1	0.0000	-1.0620	0.0076
Kpna2	0.0000	8.3761	0.0002
Kras	0.0000	-1.1452	0.0068
Krt18	0.0003	1.0013	0.0496
Ksr2	0.0000	-1.2413	0.0234
L1cam	0.0000	-1.4974	0.0052
L2hgdh	0.0000	1.0571	0.0186
L3hypdh	0.0000	-1.1104	0.0418
Lactb2	0.0000	2.3906	0.0013
Lair1	0.0001	-2.9208	0.0460
Lama1	0.0000	-1.9106	0.0038
Lama2	0.0000	-1.8752	0.0119
Lama3	0.0000	3.0102	0.0182
Lama5	0.0000	1.9742	0.0040
Lamb1	0.0000	-1.2437	0.0068
Lamc3	0.0000	-5.3556	0.0107
Lamp1	0.0000	1.1479	0.0054
Lamp2	0.0000	1.3272	0.0048
Lamp5	0.0000	-3.0733	0.0059
Lap3	0.0000	1.7949	0.0027
Laptm5	0.0000	4.1865	0.0027
Lars	0.0000	1.0374	0.0082
Lbh	0.0000	-1.8506	0.0017
Lbr	0.0000	1.3498	0.0069
Lbx1	0.0000	-3.3600	0.0197
Ldb2	0.0000	2.1809	0.0151
Ldhd	0.0000	5.5973	0.0267
Lef1	0.0000	3.1030	0.0052
Lefty1	0.0000	3.2741	0.0012
Lepr	0.0000	-3.8278	0.0177
Letmd1	0.0000	-1.2435	0.0189
Lgals1	0.0000	-1.3792	0.0088
Lgals3	0.0000	1.9167	0.0202
Lgals3bp	0.0000	2.3849	0.0122

Lgi1	0.0000	3.1889	0.0113
Lgi2	0.0000	-1.2340	0.0222
Lgi3	0.0000	-4.8741	0.0182
Lgr4	0.0000	-1.0421	0.0108
Lgr5	0.0000	-2.3316	0.0241
Lhfpl2	0.0000	-1.7624	0.0103
Lhfpl3	0.0000	-1.9954	0.0410
Lhfpl4	0.0000	-1.6128	0.0068
Lhpp	0.0000	1.0551	0.0221
Lhx2	0.0000	1.2559	0.0336
Lhx9	0.0000	6.3124	0.0022
Lig1	0.0000	1.2213	0.0068
Lima1	0.0000	1.2769	0.0053
Limch1	0.0000	-1.8739	0.0047
Limk1	0.0000	-1.0521	0.0135
Lin7a	0.0000	-3.6566	0.0036
Lingo2	0.0000	2.5357	0.0162
Lipc	0.0000	5.1477	0.0348
Lipe	0.0000	-1.1109	0.0350
Liph	0.0000	2.6187	0.0165
Lix1l	0.0000	-1.7352	0.0022
Llgl2	0.0000	2.4312	0.0036
Lmcd1	0.0000	-2.5484	0.0216
Lmo1	0.0000	-1.2070	0.0306
Lmo3	0.0000	-1.1432	0.0333
Lmo4	0.0000	-1.2315	0.0108
Lmo7	0.0000	1.9552	0.0101
Lnx1	0.0000	2.7891	0.0268
Lonrf3	0.0000	2.6301	0.0099
Lox	0.0000	-5.2620	0.0040
Loxl2	0.0000	-1.1068	0.0143
Lpar1	0.0000	-1.7013	0.0182
Lpar5	0.0001	3.4981	0.0470
Lpcat2	0.0001	1.2555	0.0455
Lpin1	0.0000	-1.1645	0.0151
Lpin3	0.0000	2.8294	0.0191
Lrch2	0.0000	-1.1915	0.0102
Lrfn2	0.0000	-1.7105	0.0380
Lrfn3	0.0000	-1.8361	0.0147
Lrfn4	0.0000	-1.5125	0.0066
Lrfn5	0.0000	-1.9577	0.0303
Lrmp	0.0000	4.0858	0.0162
Lrp12	0.0000	-1.2817	0.0089
Lrp1b	0.0000	2.6775	0.0111
Lrp2	0.0000	2.4496	0.0042
Lrp3	0.0000	-1.9146	0.0043
Lrp4	0.0000	1.9260	0.0032
Lrp8	0.0000	-1.4893	0.0057
Lrrc10b	0.0000	1.5280	0.0371

Lrrc2	0.0000	3.3923	0.0146
Lrrc20	0.0000	-1.4172	0.0142
Lrrc23	0.0000	5.5043	0.0155
Lrrc32	0.0000	-3.6211	0.0122
Lrrc34	0.0000	4.7065	0.0027
Lrrc4	0.0000	-1.9014	0.0111
Lrrc49	0.0000	-1.1126	0.0162
Lrrc4b	0.0000	-1.7762	0.0033
Lrrc4c	0.0000	-2.5138	0.0186
Lrrc75b	0.0000	-2.0898	0.0121
Lrrc8c	0.0000	1.2423	0.0134
Lrriq1	0.0000	1.8395	0.0352
Lrrk1	0.0000	-1.7219	0.0179
Lrrn1	0.0000	-2.1819	0.0018
Lrrn3	0.0000	-1.9545	0.0047
Lrrn4	0.0000	2.0482	0.0349
Lrrtm2	0.0000	-2.0846	0.0151
Lrrtm3	0.0000	-1.2617	0.0310
Lrtm2	0.0000	-1.2511	0.0430
Lsamp	0.0000	-1.8690	0.0050
Lsm11	0.0000	-1.0423	0.0162
Lsm5	0.0000	3.7507	0.0027
Lsp1	0.0000	-1.0989	0.0402
Lsr	0.0000	1.4667	0.0145
Lss	0.0000	-1.1272	0.0075
Ltv1	0.0000	1.0914	0.0127
Lum	0.0000	-7.4595	0.0008
Luzp2	0.0000	3.0923	0.0049
Ly6h	0.0000	-1.1682	0.0113
Ly75	0.0000	3.9759	0.0006
Lyn	0.0000	1.6629	0.0276
Lynx1	0.0000	-3.2120	0.0250
Lypd1	0.0000	-1.5330	0.0201
Lypd6	0.0000	-1.7379	0.0282
Lypla1	0.0000	1.3613	0.0061
Lysmd1	0.0000	-1.2380	0.0220
Lysmd2	0.0000	-1.7829	0.0281
Lysmd4	0.0000	-1.3117	0.0163
Lzts3	0.0000	-1.5633	0.0169
M1ap	0.0000	5.2519	0.0105
Madcam1	0.0000	3.8189	0.0425
Madd	0.0000	-1.1168	0.0120
Mael	0.0000	3.9988	0.0152
Magee1	0.0000	-1.8729	0.0032
Magee2	0.0000	1.8029	0.0414
Magef1	0.0000	2.1119	0.0225
Mak	0.0000	2.7517	0.0244
Mal	0.0003	1.7750	0.0492
Mal2	0.0000	2.2300	0.0417

Mall	0.0000	3.1985	0.0449
Malt1	0.0000	1.0573	0.0423
Mamdc2	0.0000	3.6676	0.0203
Maml3	0.0000	-1.3397	0.0099
Man2a2	0.0000	-1.8510	0.0035
Man2b1	0.0000	1.1309	0.0140
Man2b2	0.0000	1.0219	0.0201
Manba	0.0000	3.3570	0.0010
Maob	0.0000	4.1310	0.0243
Map10	0.0000	2.7234	0.0050
Map1b	0.0000	-1.0348	0.0045
Map3k10	0.0000	-1.5630	0.0063
Map3k13	0.0000	-1.2109	0.0199
Map3k15	0.0000	2.8177	0.0079
Map3k19	0.0000	4.9598	0.0238
Map3k5	0.0000	1.0570	0.0411
Map3k8	0.0002	1.4961	0.0479
Map7d2	0.0000	-1.0601	0.0226
Map7d3	0.0000	1.7136	0.0279
Mapk11	0.0000	-1.2683	0.0202
Mapk13	0.0000	2.4988	0.0299
Mapk8	0.0000	-1.4965	0.0043
Mapk8ip2	0.0000	-1.2543	0.0073
Mapkapk3	0.0000	1.1206	0.0195
Mapkbp1	0.0000	-1.0230	0.0169
Mapt	0.0000	-2.2349	0.0021
Marcks	0.0000	-1.1102	0.0065
Mark1	0.0000	-1.6210	0.0057
Marveld2	0.0000	2.8550	0.0165
Marveld3	0.0000	3.0456	0.0373
Mast1	0.0000	-1.0531	0.0188
Mast4	0.0000	-1.1550	0.0165
Mastl	0.0000	1.1286	0.0232
Mat2a	0.0000	1.0574	0.0080
Matk	0.0003	-1.4130	0.0491
Matn2	0.0000	-1.2194	0.0252
Mb21d2	0.0000	-1.3725	0.0100
Mblac2	0.0000	-1.1334	0.0324
Mbnl3	0.0000	1.5161	0.0275
Mboat1	0.0000	2.0918	0.0229
Mboat2	0.0000	-1.0417	0.0155
Mcf2	0.0000	4.8194	0.0011
Mcm4	0.0000	1.2780	0.0063
Mcm5	0.0000	2.3917	0.0015
Mcm6	0.0000	2.0689	0.0015
Mcm7	0.0000	1.3750	0.0066
Mcmdc2	0.0000	4.6228	0.0401
Mcoln3	0.0000	2.5646	0.0324
Mcph1	0.0000	1.5844	0.0076

Mdfi	0.0000	-1.1212	0.0201
Mdga2	0.0000	-2.2519	0.0206
Mdn1	0.0000	1.0558	0.0090
Me1	0.0000	1.0439	0.0223
Mecom	0.0000	-2.1386	0.0109
Med14	0.0000	1.1903	0.0095
Mef2c	0.0000	-1.9426	0.0315
Meg3	0.0000	-2.4071	0.0019
Megf10	0.0000	-1.0440	0.0223
Megf9	0.0000	-1.4357	0.0048
Meiob	0.0000	3.5556	0.0359
Meis1	0.0000	-1.7731	0.0042
Meis2	0.0000	-1.8090	0.0023
Meis3	0.0000	-1.7330	0.0082
Melk	0.0000	1.3674	0.0114
Meox1	0.0000	-2.8451	0.0405
Meox2	0.0000	-2.4221	0.0328
Mest	0.0000	1.4854	0.0032
Met	0.0000	-1.8724	0.0183
Metrnl	0.0000	-1.0251	0.0326
Mex3b	0.0000	-1.1089	0.0107
Mfap4	0.0000	-3.2032	0.0014
Mfhas1	0.0000	-1.3130	0.0109
Mfng	0.0000	-1.2037	0.0276
Mfsd10	0.0000	1.1746	0.0207
Mgarp	0.0000	1.2310	0.0319
Mgat3	0.0000	-2.4983	0.0016
Mgat4c	0.0000	-2.4912	0.0211
Mgat5b	0.0000	-1.7213	0.0046
Mgll	0.0000	-1.2312	0.0433
Mgst1	0.0000	-1.0814	0.0309
Mia	0.0000	4.8286	0.0116
Miat	0.0000	-1.6378	0.0441
Mib1	0.0000	-1.0130	0.0077
Mical2	0.0000	-3.0994	0.0077
Micall1	0.0000	-1.1206	0.0142
Mif4gd	0.0000	1.8156	0.0093
Mip	0.0000	2.9722	0.0420
Mir124-2hg	0.0000	-2.6871	0.0020
Mis18bp1	0.0000	1.4089	0.0087
Mitf	0.0000	1.7145	0.0224
Mkrn3	0.0000	-1.0111	0.0266
Mks1	0.0000	1.0966	0.0310
Mlc1	0.0000	1.7990	0.0258
Mlh3	0.0000	1.8605	0.0053
Mllt11	0.0000	-1.6773	0.0026
Mmab	0.0000	-1.1388	0.0184
Mme	0.0000	2.1257	0.0053
Mmp24	0.0000	-2.1671	0.0045

Mmrn2	0.0000	1.7019	0.0242
Mms22l	0.0000	1.4191	0.0116
Mnd1	0.0000	4.7767	0.0038
Moap1	0.0000	-1.1568	0.0299
Mobp	0.0000	2.6023	0.0271
Morf4l1	0.0000	1.8972	0.0014
Morn5	0.0000	2.8324	0.0453
Mov10	0.0000	2.5488	0.0012
Moxd1	0.0000	-2.5306	0.0051
Mphosph6	0.0000	1.4650	0.0079
Mpp2	0.0000	-2.1993	0.0036
Mpp3	0.0000	-1.5559	0.0210
Mpped1	0.0000	-1.5207	0.0354
Mpped2	0.0000	-1.0692	0.0326
Mras	0.0000	-2.1130	0.0044
Mrc2	0.0000	-1.6109	0.0067
Mreg	0.0000	1.8362	0.0051
Mrpl1	0.0000	1.0401	0.0190
Mrpl15	0.0000	1.5143	0.0040
Mrpl21	0.0000	1.0212	0.0194
Mrpl22	0.0000	1.2805	0.0186
Mrpl23	0.0000	4.7701	0.0035
Mrpl33	0.0000	-1.0975	0.0212
Mrpl35	0.0000	1.0990	0.0221
Mrpl42	0.0000	1.0238	0.0164
Mrpl48	0.0000	1.3540	0.0147
Mrps31	0.0000	1.3007	0.0095
Mrps36	0.0000	1.3064	0.0219
Mrps9	0.0000	1.0739	0.0105
Mrto4	0.0000	1.4547	0.0078
Mrvi1	0.0000	-3.2092	0.0409
Msh2	0.0000	1.3619	0.0039
Msrb2	0.0000	1.4294	0.0265
Msto1	0.0000	1.1140	0.0142
Msx1	0.0000	3.3925	0.0227
Mta3	0.0000	1.4085	0.0058
Mtbp	0.0000	1.0195	0.0211
Mtfp1	0.0000	1.2247	0.0398
Mthfd1	0.0000	1.9342	0.0023
Mthfd1l	0.0000	1.8624	0.0030
Mthfd2	0.0000	2.1969	0.0015
Mturn	0.0000	-1.4757	0.0046
Mtus2	0.0000	-1.7174	0.0088
Mutyh	0.0000	1.6348	0.0235
Mvb12b	0.0000	-1.2593	0.0072
Mvp	0.0000	1.3963	0.0187
Mxd1	0.0000	-1.3914	0.0210
Mxra7	0.0000	-1.6389	0.0070
Mxra8	0.0000	-1.1917	0.0220

Myb	0.0000	2.6427	0.0082
Mybbp1a	0.0000	1.2056	0.0056
Mybl1	0.0000	-1.0936	0.0288
Mybl2	0.0000	3.4050	0.0005
Mybpc1	0.0000	-1.9968	0.0337
Mybpc2	0.0000	3.4834	0.0409
Mycbpap	0.0001	2.0046	0.0458
Mycl	0.0000	-1.6470	0.0044
Myh13	0.0000	4.9162	0.0054
Myh14	0.0000	1.6610	0.0222
Myh7	0.0000	-1.9064	0.0305
Myh7b	0.0000	2.2133	0.0174
Myh8	0.0000	-2.9615	0.0387
Myh9	0.0000	1.1359	0.0077
Myl3	0.0001	-2.5640	0.0471
Myl6b	0.0000	-1.0599	0.0431
Mylk	0.0000	-1.5249	0.0253
Myo15b	0.0000	2.7831	0.0435
Myo1f	0.0000	2.4097	0.0421
Myo5a	0.0000	-1.2655	0.0046
Myo5c	0.0000	1.0164	0.0415
Myo6	0.0000	-1.1846	0.0115
Myof	0.0000	1.4718	0.0154
Myrf	0.0000	2.0542	0.0082
Myt1	0.0000	-2.1140	0.0029
Myt1l	0.0000	-1.7968	0.0127
N4bp3	0.0000	2.0228	0.0076
Nab1	0.0000	1.1022	0.0190
Nabp1	0.0000	1.5345	0.0083
Naca	0.0000	1.8589	0.0010
Nacad	0.0000	-1.7249	0.0076
Nacc2	0.0000	-1.1014	0.0267
Nae1	0.0000	1.0786	0.0110
Naf1	0.0000	1.3793	0.0076
Nanog	0.0000	4.0511	0.0003
Nanos1	0.0000	-1.1378	0.0307
Nap1l1	0.0000	1.3484	0.0041
Nap1l2	0.0000	-1.4534	0.0139
Nap1l3	0.0000	-1.7078	0.0184
Napb	0.0000	-1.4628	0.0093
Nat10	0.0000	1.1595	0.0134
Nat8l	0.0000	-1.1889	0.0154
Nav2	0.0000	-1.2048	0.0107
Nav3	0.0000	-1.1178	0.0258
Nbl1	0.0000	-3.4940	0.0023
Ncam1	0.0000	-1.5165	0.0034
Ncam2	0.0000	-2.1582	0.0155
Ncan	0.0000	-1.0053	0.0152
Ncapg	0.0000	1.3167	0.0088

Ncf2	0.0000	3.0386	0.0252
Nckap1l	0.0000	2.8973	0.0260
Ncl	0.0000	1.5745	0.0029
Ncoa4	0.0000	1.5575	0.0042
Ncs1	0.0000	-1.5900	0.0062
Ndnf	0.0000	-2.6964	0.0034
Ndrg1	0.0000	1.2507	0.0205
Ndrg3	0.0000	-1.1330	0.0123
Ndrg4	0.0000	-1.3342	0.0071
Ndufa1	0.0000	1.0916	0.0151
Ndufa4l2	0.0000	3.6051	0.0055
Ndufab1	0.0000	1.3456	0.0072
Ndufaf6	0.0000	1.3117	0.0272
Ndufb4	0.0000	2.7998	0.0023
Neat1	0.0000	2.9734	0.0068
Necab1	0.0001	1.2868	0.0458
Nefh	0.0000	1.3629	0.0215
Nefl	0.0000	-1.7647	0.0027
Nefm	0.0000	-1.1241	0.0358
Negr1	0.0000	-1.7727	0.0062
Neil2	0.0000	2.3423	0.0240
Nek10	0.0000	4.4084	0.0332
Nek2	0.0000	1.4672	0.0066
Nek5	0.0000	4.4524	0.0402
Nell2	0.0000	-2.5498	0.0008
Nes	0.0000	-1.0605	0.0117
Neurl1b	0.0000	-1.0577	0.0403
Neurod1	0.0000	-1.0540	0.0332
Neurod2	0.0000	-1.8742	0.0431
Neurod6	0.0000	-4.9777	0.0178
Nexn	0.0000	-1.7356	0.0212
Nfatc2ip	0.0000	1.5190	0.0061
Nfia	0.0000	-1.7803	0.0084
Nfib	0.0000	-1.5746	0.0360
Nfkbia	0.0000	1.3367	0.0151
Ngef	0.0000	1.6174	0.0309
Ngfr	0.0000	-1.9746	0.0032
Nhp2	0.0000	1.0267	0.0181
Nhs	0.0000	-1.2570	0.0195
Nicn1	0.0000	-1.1524	0.0114
Nid1	0.0000	-1.6385	0.0025
Nifk	0.0000	1.1095	0.0089
Nip7	0.0000	1.0475	0.0121
Nipa1	0.0000	-1.3926	0.0151
Nkain1	0.0000	-1.0400	0.0196
Nkain3	0.0000	-1.1448	0.0410
Nkd1	0.0000	1.3215	0.0101
Nkiras1	0.0000	-1.2769	0.0211
Nkx2-2	0.0000	-5.1620	0.0083

Nkx6-1	0.0000	-6.1467	0.0008
Nlgn1	0.0000	-1.8764	0.0216
Nlgn2	0.0000	-1.5043	0.0045
Nlrc4	0.0000	3.4826	0.0311
Nlrp12	0.0001	2.0685	0.0473
Nlrp14	0.0000	3.5251	0.0429
Nlrx1	0.0003	2.9475	0.0488
Nmd3	0.0000	1.2187	0.0087
Nme7	0.0000	1.2637	0.0160
Nmi	0.0000	3.5427	0.0288
Nmrk1	0.0002	1.0086	0.0487
Noct	0.0000	3.6954	0.0003
Nodal	0.0000	3.4171	0.0023
Nol3	0.0000	1.1515	0.0320
Nol4	0.0000	-1.2326	0.0344
Nol4l	0.0000	-1.3798	0.0082
Nop2	0.0000	1.4596	0.0050
Nop58	0.0000	1.4622	0.0021
Nos1	0.0000	1.3825	0.0251
Notch1	0.0000	-1.0055	0.0168
Notum	0.0000	5.0669	0.0204
Nova1	0.0000	-2.7093	0.0019
Nova2	0.0000	-1.7990	0.0034
Npas3	0.0000	-1.8782	0.0081
Npdc1	0.0000	-1.3608	0.0068
Npepl1	0.0000	2.5051	0.0116
Nphs1	0.0000	-2.1440	0.0025
Npm3	0.0000	1.3617	0.0103
Npr2	0.0000	-1.4205	0.0133
Nptx2	0.0000	1.1976	0.0245
Nqo1	0.0000	2.8355	0.0047
Nr0b1	0.0000	4.4266	0.0013
Nr2c1	0.0000	-1.3434	0.0224
Nr2e1	0.0000	4.2922	0.0432
Nr2f1	0.0000	-2.0178	0.0018
Nr2f2	0.0000	-2.1503	0.0030
Nr3c2	0.0000	2.1105	0.0251
Nr4a2	0.0000	-1.7978	0.0115
Nr4a3	0.0000	1.4098	0.0222
Nrbp2	0.0000	-1.7000	0.0103
Nrcam	0.0000	-1.4434	0.0036
Nrep	0.0000	-1.9536	0.0012
Nrg3	0.0000	-1.8925	0.0430
Nrgn	0.0000	-1.4955	0.0282
Nrn1	0.0000	1.8932	0.0136
Nrp1	0.0000	-3.1097	0.0015
Nrp2	0.0000	-1.0071	0.0170
Nrxn1	0.0000	-1.1756	0.0160
Nrxn2	0.0000	-1.5567	0.0066

Nrxn3	0.0000	-1.9487	0.0058
Nsg1	0.0000	-1.7050	0.0030
Nsmf	0.0000	-1.3424	0.0109
Nt5dc1	0.0000	1.9265	0.0162
Nt5m	0.0000	-1.0494	0.0309
Ntn1	0.0000	-3.4235	0.0003
Ntn4	0.0000	-2.5197	0.0062
Ntng1	0.0000	-1.4308	0.0274
Ntng2	0.0000	-1.9765	0.0193
Ntrk2	0.0000	-1.7956	0.0084
Ntrk3	0.0000	-1.6347	0.0065
Nts	0.0000	-2.3825	0.0317
Nudc	0.0000	1.3794	0.0098
Nudt16	0.0000	-1.3708	0.0265
Nudt3	0.0000	-1.0050	0.0101
Nudt7	0.0002	1.1140	0.0485
Nup133	0.0000	1.2169	0.0091
Nup160	0.0000	1.1684	0.0090
Nup188	0.0000	1.0904	0.0102
Nup205	0.0000	1.0640	0.0081
Nup35	0.0000	1.6066	0.0106
Nup54	0.0000	1.0065	0.0143
Nup62cl	0.0000	1.5778	0.0237
Nup85	0.0000	1.1453	0.0121
Nup93	0.0000	1.5713	0.0025
Nutf2	0.0000	5.3239	0.0001
Nwd2	0.0000	-2.7019	0.0126
Nxph1	0.0000	-6.9555	0.0131
Nyap1	0.0000	-1.5225	0.0059
Oas2	0.0000	3.5527	0.0364
Oas3	0.0000	3.3798	0.0368
Ocln	0.0000	3.6762	0.0084
Odc1	0.0000	1.5035	0.0023
Ogdhl	0.0000	1.0259	0.0099
Ogn	0.0000	-1.8509	0.0407
Oip5	0.0000	1.1749	0.0286
Olfm1	0.0000	-1.6256	0.0053
Olfm2	0.0000	-1.8210	0.0051
Olfm3	0.0000	-1.4928	0.0392
Olfml1	0.0000	-2.2265	0.0217
Olfml2a	0.0000	1.2874	0.0281
Olfml2b	0.0000	-1.9515	0.0200
Olfml3	0.0000	-1.8964	0.0123
Olig2	0.0000	-1.7820	0.0337
Oma1	0.0000	1.1448	0.0283
Onecut3	0.0000	1.1263	0.0317
Opa3	0.0000	-1.5454	0.0065
Ophn1	0.0000	-1.0113	0.0259
Oprl1	0.0000	-1.1958	0.0255

Optn	0.0000	1.3407	0.0139
Orc1	0.0000	2.7174	0.0029
Orc2	0.0000	1.5659	0.0037
Ormdl2	0.0000	1.1798	0.0227
Osbpl6	0.0000	-1.6388	0.0122
Osmr	0.0003	1.9359	0.0496
Osr1	0.0000	-2.4887	0.0145
Ostf1	0.0000	1.3522	0.0130
Otud1	0.0000	1.7789	0.0180
Otud4	0.0000	1.3835	0.0050
Otud7a	0.0000	-1.1451	0.0375
Otx2	0.0000	4.4568	0.0007
Oxa1l	0.0000	-1.0160	0.0140
P2rx7	0.0000	1.6606	0.0229
P2ry1	0.0000	-1.2518	0.0453
P2ry14	0.0000	-3.2098	0.0358
P3h2	0.0000	1.2963	0.0209
P3h4	0.0000	-1.4814	0.0137
P4ha3	0.0000	-2.4036	0.0088
P4htm	0.0000	-2.1168	0.0184
Pafah1b2	0.0000	-1.0165	0.0078
Pafah2	0.0000	-1.0978	0.0323
Pak3	0.0000	-2.4097	0.0020
Pak7	0.0000	-1.9053	0.0078
Palb2	0.0000	1.4744	0.0198
Palm3	0.0000	1.7315	0.0127
Palmd	0.0000	5.8556	0.0013
Pam16	0.0000	4.3479	0.0068
Pank3	0.0000	-1.0398	0.0103
Papss2	0.0000	-2.0794	0.0361
Paqr4	0.0000	-1.1635	0.0216
Paqr9	0.0000	-2.0964	0.0336
Pard6g	0.0000	-1.0886	0.0143
Parm1	0.0000	-2.6321	0.0024
Parp10	0.0000	1.7333	0.0299
Parp12	0.0000	2.3311	0.0093
Parp14	0.0000	3.3007	0.0244
Parp3	0.0000	1.6169	0.0325
Parp4	0.0000	2.0805	0.0159
Parp6	0.0000	-1.2640	0.0126
Parp9	0.0000	2.2648	0.0150
Parvb	0.0000	-1.2317	0.0265
Pask	0.0000	1.3576	0.0112
Patl2	0.0000	3.8150	0.0266
Pawr	0.0000	1.1634	0.0165
Pax2	0.0000	-3.7348	0.0025
Pax6	0.0000	-1.4562	0.0119
Pax8	0.0000	-3.3540	0.0076
Pbx3	0.0000	-1.4497	0.0065

Pcdh10	0.0000	-2.3509	0.0062
Pcdh17	0.0000	-3.1651	0.0034
Pcdh18	0.0000	-1.2946	0.0085
Pcdh19	0.0000	-1.7906	0.0171
Pcdh9	0.0000	-1.9585	0.0090
Pcdha3	0.0000	-1.6440	0.0138
Pcdha8	0.0000	2.1521	0.0371
Pcdhb10	0.0000	-1.2761	0.0243
Pcdhb3	0.0000	-1.5977	0.0173
Pcdhb5	0.0000	-5.8539	0.0049
Pcdhb7	0.0000	-1.5481	0.0235
Pcdhb8	0.0000	-2.3463	0.0196
Pcdhga10	0.0000	-1.0138	0.0323
Pcdhga4	0.0000	-1.0316	0.0327
Pcdhgb6	0.0000	-2.3443	0.0077
Pcdhgb7	0.0000	-1.4562	0.0269
Pced1a	0.0000	-1.3877	0.0154
Pced1b	0.0000	2.2179	0.0095
Pclo	0.0000	-1.5566	0.0107
Pcna	0.0000	1.5943	0.0032
Pcnt	0.0000	1.3043	0.0065
Pcolce	0.0000	-1.7378	0.0093
Pcolce2	0.0000	3.3219	0.0103
Pcp4	0.0000	-5.3758	0.0027
Pcp4l1	0.0000	3.4020	0.0016
Pcsk1	0.0000	5.4942	0.0087
Pcsk6	0.0000	1.7263	0.0057
Pdcd10	0.0000	1.4200	0.0092
Pdcd11	0.0000	1.0393	0.0120
Pdcl2	0.0000	5.3138	0.0212
Pde10a	0.0000	-1.8408	0.0079
Pde1a	0.0000	-1.8668	0.0344
Pde1b	0.0000	-1.1985	0.0136
Pde5a	0.0000	-1.0779	0.0157
Pde8a	0.0000	3.7584	0.0062
Pde8b	0.0000	-1.4145	0.0353
Pdgfd	0.0001	1.8583	0.0463
Pdgfra	0.0000	-2.3092	0.0048
Pdgfrb	0.0000	-3.2949	0.0021
Pdia6	0.0000	1.0776	0.0079
Pdik1l	0.0000	-1.0311	0.0234
Pdk2	0.0000	-1.0652	0.0302
Pdk4	0.0000	3.5951	0.0219
Pdlim1	0.0000	1.7778	0.0100
Pdlim3	0.0000	-2.5355	0.0221
Pdxp	0.0000	-1.0400	0.0167
Pdzd9	0.0000	-3.3172	0.0444
Pdzrn3	0.0000	-1.5416	0.0074
Pdzrn4	0.0000	-2.5048	0.0052

Pecam1	0.0000	3.2486	0.0019
Pecr	0.0000	1.7686	0.0110
Peli2	0.0000	-1.7860	0.0070
Peli3	0.0000	-1.4626	0.0271
Per3	0.0000	-2.0908	0.0114
Pfkfb1	0.0000	1.1318	0.0446
Pfkfb3	0.0000	-1.4796	0.0057
Pfkfb4	0.0000	-1.0014	0.0387
Pfkp	0.0000	2.9829	0.0009
Pgam2	0.0000	1.6276	0.0439
Pgf	0.0000	-4.1708	0.0158
Pgk1	0.0000	1.8448	0.0010
Pgm2l1	0.0000	-1.9077	0.0026
Pgm5	0.0000	2.2196	0.0174
Phactr1	0.0000	-1.3028	0.0232
Phb	0.0000	1.7529	0.0031
Phf1	0.0000	-1.1207	0.0261
Phf21b	0.0000	-1.9669	0.0039
Phf24	0.0000	-2.4715	0.0071
Phgdh	0.0000	1.3020	0.0058
Phlda1	0.0000	-1.9304	0.0039
Phldb2	0.0000	-1.2393	0.0114
Phyh	0.0000	1.3352	0.0133
Phyhd1	0.0000	2.9942	0.0239
Phyhipl	0.0000	-2.0368	0.0037
Pi15	0.0000	-5.6550	0.0174
Pid1	0.0000	-1.3434	0.0102
Piezo1	0.0000	1.0281	0.0138
Piezo2	0.0000	-3.3334	0.0221
Pik3ip1	0.0000	-1.6008	0.0109
Pik3r5	0.0000	-2.1209	0.0442
Pim2	0.0000	2.1988	0.0049
Pin4	0.0000	3.0853	0.0048
Pink1	0.0000	-1.6585	0.0042
Pip4k2b	0.0000	-1.0558	0.0095
Pir	0.0000	2.2693	0.0152
Pirt	0.0000	4.6631	0.0228
Pitpnc1	0.0000	-1.0553	0.0109
Pitpnm1	0.0000	-1.1955	0.0251
Piwil2	0.0000	4.5080	0.0068
Pkdcc	0.0000	-1.9556	0.0034
Pkia	0.0000	-2.0219	0.0026
Pkig	0.0000	-1.2489	0.0270
Pkn3	0.0000	1.4294	0.0271
Pknox2	0.0000	-1.0168	0.0203
Pkp2	0.0000	2.1408	0.0047
Pla1a	0.0000	4.6307	0.0143
Pla2g10	0.0000	3.5032	0.0271
Pla2g5	0.0000	3.9842	0.0224

Pla2g7	0.0000	2.7382	0.0069
Pla2r1	0.0000	3.9369	0.0442
Plbd1	0.0000	2.8943	0.0297
Plcb1	0.0000	-1.8372	0.0107
Plcd1	0.0000	-1.9655	0.0091
Plcd3	0.0000	1.2857	0.0270
Plcd4	0.0000	2.6663	0.0149
Plcg2	0.0000	3.4688	0.0035
Plcl1	0.0000	-2.0905	0.0101
Pld1	0.0000	-1.1555	0.0286
Pld6	0.0000	2.5063	0.0274
Plek2	0.0000	4.3241	0.0385
Plekhb1	0.0004	1.0524	0.0498
Plekhf2	0.0000	1.0888	0.0116
Plekhg3	0.0000	1.9357	0.0074
Plekhh1	0.0000	1.2059	0.0148
Plekhh2	0.0000	-1.0808	0.0282
Plekho1	0.0000	-1.1481	0.0173
Plet1	0.0000	2.1906	0.0364
Plin2	0.0000	1.5423	0.0075
Plin3	0.0000	1.1964	0.0092
Plk1	0.0000	1.2736	0.0098
Plp2	0.0000	3.7253	0.0004
Plpp3	0.0000	-1.5723	0.0056
Plppr1	0.0000	-1.4081	0.0126
Plppr2	0.0000	-1.7201	0.0054
Plppr4	0.0000	2.2278	0.0240
Plppr5	0.0000	-2.1797	0.0442
Plscr1	0.0000	1.2010	0.0229
Pltp	0.0000	-1.0326	0.0086
Plxdc1	0.0000	3.0211	0.0192
Plxna2	0.0000	-1.1466	0.0061
Plxna3	0.0000	-1.1359	0.0141
Plxna4	0.0000	-1.1258	0.0186
Plxnd1	0.0000	-2.3018	0.0075
Pmaip1	0.0000	4.1928	0.0008
Pmf1	0.0000	1.1529	0.0174
Pml	0.0000	1.7540	0.0033
Pmp22	0.0000	-1.2622	0.0164
Pms1	0.0000	1.5129	0.0107
Pnldc1	0.0000	4.0631	0.0161
Pnma2	0.0000	-1.7594	0.0121
Pnma3	0.0000	5.3737	0.0229
Pnma5	0.0000	4.1972	0.0049
Pnmal1	0.0000	-1.7996	0.0106
Pnmal2	0.0000	-1.7008	0.0044
Pnoc	0.0000	-2.1720	0.0189
Pnpla6	0.0000	1.1821	0.0101
Pnpla7	0.0000	1.1732	0.0320

Pnpt1	0.0000	2.5398	0.0013
Podxl2	0.0000	-1.5702	0.0056
Pola1	0.0000	1.8187	0.0048
Pola2	0.0000	1.2268	0.0127
Pold1	0.0000	1.5248	0.0062
Pole	0.0000	1.9287	0.0035
Pole2	0.0000	2.0903	0.0094
Polr1a	0.0000	1.7028	0.0030
Polr1b	0.0000	1.4962	0.0082
Polr3g	0.0000	1.6726	0.0065
Polr3gl	0.0000	-1.0265	0.0300
Pon3	0.0000	4.1122	0.0304
Postn	0.0000	-4.0023	0.0069
Pou2f2	0.0000	-2.2798	0.0178
Pou2f3	0.0000	4.9090	0.0141
Pou3f1	0.0000	-1.1758	0.0246
Pou3f2	0.0000	-1.9694	0.0045
Pou4f1	0.0000	-1.4966	0.0154
Pou6f1	0.0000	-2.5613	0.0026
Pou6f2	0.0000	2.2585	0.0113
Ppan	0.0000	1.4879	0.0069
Ppat	0.0000	1.4046	0.0081
Ppfia2	0.0000	-1.2412	0.0208
Ppfia3	0.0000	-1.4552	0.0133
Ppia	0.0000	2.4850	0.0004
Ppid	0.0000	1.1725	0.0061
Ppih	0.0000	3.7368	0.0029
Ppil1	0.0000	-1.0128	0.0202
Ppm1e	0.0000	-1.5641	0.0060
Ppm1h	0.0000	-1.4268	0.0124
Ppm1j	0.0000	3.1376	0.0171
Ppm1k	0.0000	-1.2085	0.0271
Ppm1l	0.0000	-1.8005	0.0035
Ppp1r14c	0.0000	-2.3916	0.0117
Ppp1r15b	0.0000	1.0922	0.0064
Ppp1r17	0.0000	-2.0812	0.0340
Ppp1r32	0.0000	3.6608	0.0363
Ppp1r37	0.0000	-1.0018	0.0140
Ppp1r3c	0.0000	-3.2973	0.0025
Ppp1r3d	0.0000	-1.4051	0.0415
Ppp1r3e	0.0000	-1.6000	0.0415
Ppp1r42	0.0003	3.2054	0.0496
Ppp1r9b	0.0000	-1.0896	0.0112
Ppp2r2b	0.0000	-2.2130	0.0063
Ppp2r2c	0.0000	-1.2961	0.0204
Ppp2r3a	0.0000	-1.8139	0.0055
Ppp2r5b	0.0000	-1.4870	0.0064
Ppp3ca	0.0000	-1.4183	0.0054
Pqlc3	0.0000	1.1071	0.0258

Prcp	0.0000	1.3432	0.0107
Prdm1	0.0000	3.7418	0.0054
Prdm16	0.0000	1.4031	0.0189
Prdm5	0.0000	1.5790	0.0204
Prdm8	0.0000	-3.9162	0.0019
Prdx1	0.0000	1.6917	0.0018
Prelid2	0.0000	3.3439	0.0088
Prickle1	0.0000	-2.0099	0.0059
Prickle3	0.0000	4.6105	0.0187
Prim1	0.0000	1.2147	0.0158
Prkag2	0.0000	-1.0607	0.0176
Prkar1b	0.0000	-1.0740	0.0220
Prkar2a	0.0000	-1.0163	0.0083
Prkar2b	0.0000	-1.4451	0.0061
Prkca	0.0000	-1.2060	0.0266
Prkcb	0.0000	-1.1158	0.0250
Prkce	0.0000	-1.7768	0.0076
Prkcg	0.0000	2.3542	0.0258
Prmt7	0.0000	1.3311	0.0053
Prmt8	0.0000	-1.6915	0.0079
Prodh	0.0000	1.0955	0.0380
Prom1	0.0000	-1.2913	0.0058
Prox1	0.0000	-2.8438	0.0018
Prpf19	0.0000	1.0887	0.0061
Prph	0.0000	-2.1122	0.0135
Prr16	0.0000	-3.5405	0.0370
Prr18	0.0000	-1.6249	0.0285
Prrg1	0.0000	-1.1043	0.0352
Prrt2	0.0000	-1.6350	0.0445
Prrt4	0.0000	-1.7526	0.0376
Prrx1	0.0000	-1.9656	0.0405
Prrx2	0.0000	-1.5585	0.0402
Prtn3	0.0000	-1.7849	0.0336
Prune2	0.0000	1.2221	0.0197
Psd	0.0000	-1.9575	0.0079
Psd4	0.0000	3.2579	0.0194
Psma5	0.0000	1.6115	0.0040
Psme2	0.0000	2.4324	0.0029
Ptbp1	0.0000	1.3236	0.0036
Ptch1	0.0000	-2.0568	0.0017
Ptger3	0.0000	-3.9215	0.0175
Ptges3	0.0000	1.5863	0.0020
Ptgr1	0.0000	1.0369	0.0238
Ptgs1	0.0000	-1.3896	0.0369
Ptgs2	0.0000	2.0951	0.0372
Pthlh	0.0000	4.5403	0.0046
Ptk2b	0.0000	2.7459	0.0159
Ptpn3	0.0000	1.7582	0.0106
Ptpn5	0.0000	-1.2362	0.0292

Ptpn6	0.0000	3.9622	0.0028
Ptprh	0.0000	4.4108	0.0399
Ptprn2	0.0000	-1.4339	0.0130
Ptpro	0.0000	-1.5042	0.0100
Ptprq	0.0000	4.3919	0.0244
Ptprt	0.0000	1.2673	0.0180
Ptpru	0.0000	-1.0923	0.0226
Ptprz1	0.0000	-2.7366	0.0010
Pvt1	0.0000	2.7364	0.0226
Pwwp2b	0.0000	-1.5005	0.0247
Pxdn	0.0000	-1.6882	0.0027
Pxmp4	0.0000	-1.3695	0.0160
Pxylp1	0.0000	-2.3887	0.0041
Pycard	0.0000	4.3418	0.0074
Pygl	0.0000	5.5198	0.0018
Pygo1	0.0000	-1.8499	0.0050
Pyroxd2	0.0000	2.5220	0.0434
Qpct	0.0000	-2.4747	0.0148
Qpctl	0.0000	-1.7641	0.0166
Qsox1	0.0000	-1.1777	0.0104
Qtrt1	0.0000	1.0964	0.0235
Rab11fip1	0.0000	1.6654	0.0095
Rab11fip3	0.0000	-1.0169	0.0125
Rab25	0.0003	2.9142	0.0494
Rab27a	0.0000	3.2233	0.0042
Rab30	0.0000	-2.1350	0.0059
Rab39b	0.0000	-1.1188	0.0191
Rab3a	0.0000	-1.2614	0.0127
Rab3c	0.0000	-2.7088	0.0016
Rab3il1	0.0000	-1.2951	0.0191
Rab6b	0.0000	-1.6949	0.0036
Rad50	0.0000	1.3800	0.0071
Rad54b	0.0000	1.6574	0.0112
Ralgds	0.0000	-1.1114	0.0130
Ralgps1	0.0000	-1.0784	0.0193
Ralyl	0.0000	-1.8610	0.0218
Ramp2	0.0000	-2.2108	0.0183
Ramp3	0.0000	-1.5860	0.0380
Ranbp17	0.0000	1.5973	0.0097
Rangrf	0.0000	2.0733	0.0085
Rapgef5	0.0000	-1.4642	0.0120
Rapgefl1	0.0000	-1.2273	0.0339
Rara	0.0000	-1.1185	0.0128
Rarb	0.0000	-5.4590	0.0034
Rasd2	0.0000	1.9232	0.0322
Rasgef1a	0.0003	1.9175	0.0489
Rasgef1c	0.0000	-3.2246	0.0281
Rasgrf2	0.0000	-2.9707	0.0226
Rasgrp1	0.0000	3.9423	0.0081

Rasgrp2	0.0000	1.7744	0.0188
Rasgrp4	0.0000	2.0444	0.0287
Rasl10b	0.0000	-1.9405	0.0044
Rasl11a	0.0001	-1.4953	0.0462
Rassf10	0.0000	-2.0110	0.0277
Rassf2	0.0000	-1.1946	0.0109
Rassf3	0.0000	1.1286	0.0176
Rassf8	0.0000	-1.2875	0.0170
Rbbp5	0.0000	1.1086	0.0101
Rbbp9	0.0000	-1.5310	0.0074
Rbfox1	0.0000	-1.7628	0.0157
Rbfox2	0.0000	-1.0543	0.0071
Rbfox3	0.0000	-2.8186	0.0035
Rbm10	0.0000	1.6062	0.0030
Rbm24	0.0000	-2.4658	0.0202
Rbm3	0.0000	1.5495	0.0025
Rbm43	0.0000	2.2242	0.0290
Rbm47	0.0000	3.6308	0.0058
Rbms3	0.0000	-2.4535	0.0043
Rbp1	0.0000	-3.7807	0.0009
Rbpms	0.0000	2.3861	0.0017
Rbpms2	0.0000	2.7192	0.0012
Rcan2	0.0000	-2.8522	0.0010
Rccd1	0.0000	-1.4974	0.0206
Rcl1	0.0000	1.3781	0.0120
Rcn3	0.0000	-1.4369	0.0101
Rcsd1	0.0000	-2.0225	0.0123
Rdh12	0.0001	2.8000	0.0461
Rdm1	0.0000	1.1631	0.0227
Rec8	0.0000	4.1958	0.0031
Recql4	0.0000	1.1855	0.0349
Reep1	0.0000	-1.0261	0.0151
Reep2	0.0000	-1.2892	0.0209
Relb	0.0000	1.7955	0.0166
Rell2	0.0000	-1.4751	0.0278
Rem2	0.0000	-1.7551	0.0128
Renbp	0.0000	1.5305	0.0081
Reps2	0.0000	-2.0463	0.0160
Resp18	0.0000	-1.1444	0.0391
Rest	0.0000	1.4558	0.0074
Ret	0.0000	-1.5108	0.0206
Retsat	0.0000	2.6983	0.0074
Rev3l	0.0000	-1.1039	0.0133
Rfc5	0.0000	1.7107	0.0034
Rftn1	0.0000	1.2817	0.0277
Rfx2	0.0000	1.1526	0.0218
Rfx4	0.0000	-1.2445	0.0088
Rgma	0.0000	-2.6378	0.0012
Rgs10	0.0001	-1.6127	0.0459

Rgs17	0.0000	-1.2564	0.0355
Rgs20	0.0000	-1.0172	0.0401
Rgs7	0.0000	-2.0013	0.0207
Rgs7bp	0.0000	-2.0770	0.0185
Rgs8	0.0000	-1.1572	0.0197
Rhbdl3	0.0000	-3.5356	0.0018
Rhob	0.0000	-1.2890	0.0052
Rhobtb3	0.0000	-1.0357	0.0123
Rhoj	0.0000	-3.5616	0.0056
Ribc1	0.0000	1.1357	0.0425
Rif1	0.0000	1.1060	0.0059
Rimklb	0.0000	-1.0012	0.0105
Rims4	0.0000	-1.6022	0.0185
Rin1	0.0000	1.8094	0.0261
Rin3	0.0001	-1.2380	0.0471
Ripk3	0.0000	3.2152	0.0323
Rit2	0.0000	2.3516	0.0329
Rmi2	0.0000	1.0625	0.0373
Rnaseh2a	0.0000	1.1686	0.0130
Rnd2	0.0000	-1.3618	0.0132
Rnd3	0.0000	-2.5418	0.0005
Rnf112	0.0000	-1.8209	0.0343
Rnf122	0.0000	-1.7183	0.0077
Rnf125	0.0000	4.0890	0.0177
Rnf135	0.0000	2.4988	0.0252
Rnf144a	0.0000	-1.6285	0.0058
Rnf150	0.0000	-1.4166	0.0136
Rnf157	0.0000	-1.9393	0.0033
Rnf165	0.0000	-1.8247	0.0049
Rnf187	0.0000	-1.1204	0.0046
Rnf19a	0.0000	-1.0069	0.0137
Rnf213	0.0000	1.0330	0.0243
Rnf24	0.0000	-1.0855	0.0168
Rnft2	0.0000	-1.2258	0.0145
Rnls	0.0000	2.0223	0.0390
Robo1	0.0000	-1.6573	0.0037
Robo2	0.0000	-1.2509	0.0115
Ror1	0.0000	1.8532	0.0150
Rora	0.0000	-1.2615	0.0279
Rorb	0.0000	-1.2722	0.0451
Rpa1	0.0000	1.0881	0.0069
Rpa3	0.0000	1.2252	0.0171
Rpgr	0.0000	1.0101	0.0127
Rph3a	0.0000	-2.3390	0.0224
Rpl12	0.0000	4.9194	0.0001
Rpl13	0.0000	3.9938	0.0004
Rpl14	0.0000	2.1213	0.0012
Rpl15	0.0000	1.1867	0.0031
Rpl17	0.0000	5.0805	0.0001

Rpl18	0.0000	1.7376	0.0016
Rpl18a	0.0000	1.9361	0.0018
Rpl19	0.0000	2.6082	0.0008
Rpl23	0.0000	2.3286	0.0007
Rpl24	0.0000	4.3456	0.0002
Rpl27a	0.0000	3.4602	0.0001
Rpl28	0.0000	2.0118	0.0033
Rpl29	0.0000	4.5979	0.0022
Rpl3	0.0000	4.7606	0.0002
Rpl35	0.0000	2.3968	0.0011
Rpl36	0.0000	1.8572	0.0020
Rpl36a	0.0000	3.7047	0.0003
Rpl39l	0.0000	4.9830	0.0032
Rpl3l	0.0003	3.7830	0.0491
Rpl5	0.0000	4.9308	0.0002
Rpl6	0.0000	1.8811	0.0017
Rpl7a	0.0000	1.9619	0.0018
Rpp25	0.0000	1.5398	0.0124
Rpp40	0.0000	1.1629	0.0300
Rps10	0.0000	3.4464	0.0002
Rps12	0.0000	4.4377	0.0001
Rps14	0.0000	1.0752	0.0132
Rps15a	0.0000	1.3419	0.0055
Rps17	0.0000	1.4403	0.0027
Rps2	0.0000	1.5239	0.0045
Rps20	0.0000	1.2247	0.0079
Rps23	0.0000	3.0145	0.0002
Rps24	0.0000	1.0805	0.0049
Rps27a	0.0000	3.8594	0.0002
Rps27l	0.0000	1.0510	0.0093
Rps6	0.0000	3.6331	0.0004
Rps6ka1	0.0000	1.2895	0.0096
Rps8	0.0000	1.9168	0.0012
Rpsa	0.0000	1.2609	0.0048
Rrp12	0.0000	1.0543	0.0139
Rrp15	0.0000	1.3965	0.0079
Rrp1b	0.0000	1.3245	0.0080
Rsph9	0.0000	1.4222	0.0383
Rspo2	0.0000	5.4560	0.0005
Rspo3	0.0000	2.5157	0.0231
Rspo4	0.0000	3.3732	0.0413
Rtn1	0.0000	-1.3411	0.0059
Rtn2	0.0000	-1.1686	0.0150
Rtn4rl1	0.0000	1.8152	0.0178
Rtp4	0.0000	4.7218	0.0255
Rttn	0.0000	1.0860	0.0241
Rufy3	0.0000	-1.1954	0.0047
Rufy4	0.0000	-2.6873	0.0361
Runx1	0.0000	-2.2766	0.0243

Runx1t1	0.0000	-2.0620	0.0090
Rusc1	0.0000	-1.2394	0.0149
Rusc2	0.0000	-1.3907	0.0086
Ruvbl1	0.0000	1.6956	0.0045
Ruvbl2	0.0000	1.0467	0.0108
Rxra	0.0000	-1.0214	0.0141
S100a1	0.0000	1.4919	0.0400
S100a6	0.0000	1.3272	0.0402
S1pr1	0.0000	-2.1237	0.0120
S1pr3	0.0000	1.1592	0.0239
Sall2	0.0000	-1.2461	0.0079
Sall3	0.0000	-1.3938	0.0088
Sall4	0.0000	2.4471	0.0030
Samd10	0.0000	-1.6533	0.0095
Samd14	0.0000	-2.0890	0.0022
Samd9l	0.0000	2.2330	0.0318
Sap18	0.0000	2.8724	0.0008
Sarm1	0.0000	-1.7803	0.0095
Sat1	0.0000	1.2750	0.0135
Sbk1	0.0000	-1.9437	0.0012
Sc5d	0.0000	-1.7897	0.0038
Scarf1	0.0000	3.1221	0.0293
Scg2	0.0000	-1.0961	0.0099
Scg3	0.0000	-2.1127	0.0022
Scg5	0.0000	-1.6010	0.0180
Scml2	0.0000	1.2182	0.0435
Scn1a	0.0000	-2.1386	0.0356
Scn2a	0.0000	-1.5532	0.0148
Scn2b	0.0000	-1.7985	0.0232
Scn3a	0.0000	-1.1671	0.0145
Scn3b	0.0000	-2.1532	0.0023
Scn5a	0.0002	-2.0808	0.0478
Scn7a	0.0004	2.0994	0.0496
Scn9a	0.0000	-1.4764	0.0349
Scrt1	0.0000	-1.7801	0.0048
Scrt2	0.0000	-2.4794	0.0026
Scube1	0.0000	-2.5900	0.0020
Scube2	0.0000	-1.8788	0.0296
Sdc2	0.0000	-1.1014	0.0153
Sdc3	0.0000	-1.6280	0.0036
Sdc4	0.0000	1.2834	0.0087
Sdf2l1	0.0000	1.2375	0.0150
Sdk2	0.0000	-1.7392	0.0047
Sdr39u1	0.0000	4.3318	0.0093
Sdsl	0.0001	1.8977	0.0475
Sec14l4	0.0000	5.3117	0.0297
Sec14l5	0.0000	-1.6689	0.0329
Sec61g	0.0000	3.2328	0.0012
Sema3a	0.0000	-1.8419	0.0133

Sema3d	0.0000	-1.6418	0.0351
Sema5a	0.0000	-1.8632	0.0147
Sema6b	0.0000	-2.0069	0.0098
Sema6c	0.0000	-1.4541	0.0075
Sema6d	0.0000	-1.0204	0.0191
Senp7	0.0000	-1.0193	0.0160
Sephs2	0.0000	1.1055	0.0087
Serp2	0.0000	-1.1233	0.0451
Serpinb8	0.0000	3.2387	0.0410
Serpine1	0.0000	1.2710	0.0275
Serping1	0.0000	-1.9002	0.0160
Serpini1	0.0000	-2.1786	0.0033
Sertm1	0.0001	-1.1841	0.0456
Sestd1	0.0000	-1.3826	0.0078
Setbp1	0.0000	-1.3590	0.0065
Setd6	0.0000	1.1365	0.0245
Sez6	0.0000	-1.5714	0.0094
Sez6l	0.0000	-1.6662	0.0087
Sez6l2	0.0000	-1.4414	0.0094
Sf3b3	0.0000	1.4517	0.0025
Sfn	0.0000	1.7645	0.0288
Sfrp1	0.0000	-1.5837	0.0021
Sfrp4	0.0003	1.2587	0.0489
Sft2d1	0.0000	1.4115	0.0188
Sgip1	0.0000	-1.7769	0.0083
Sgk1	0.0000	2.6510	0.0017
Sgk3	0.0000	3.0529	0.0057
Sgms2	0.0000	2.9236	0.0078
Sgpp2	0.0000	2.1988	0.0404
Sgsm1	0.0000	-1.0087	0.0253
Sgtb	0.0000	-1.3785	0.0117
Sh2b2	0.0000	-1.4917	0.0191
Sh2d3c	0.0000	-1.5911	0.0103
Sh2d4a	0.0000	4.7529	0.0253
Sh2d5	0.0000	-2.1668	0.0236
Sh3bgrl	0.0000	-1.2765	0.0055
Sh3bgrl2	0.0000	1.6408	0.0148
Sh3bp1	0.0000	1.2878	0.0178
Sh3bp5	0.0000	-1.5738	0.0096
Sh3pxd2a	0.0000	-1.0762	0.0376
Sh3rf3	0.0000	-1.2919	0.0318
Sh3tc1	0.0000	3.3478	0.0091
Sh3tc2	0.0001	1.7211	0.0466
Sh3yl1	0.0000	-1.2461	0.0219
Shank1	0.0000	-1.5660	0.0106
Shc2	0.0000	-1.2909	0.0115
Shcbp1	0.0000	1.4912	0.0081
Shd	0.0000	-1.5383	0.0144
She	0.0000	1.5812	0.0333

Shf	0.0000	-1.4095	0.0142
Shisa7	0.0000	-1.4948	0.0163
Shisa8	0.0000	2.1187	0.0321
Shisa9	0.0000	-1.3451	0.0338
Shmt1	0.0000	2.4675	0.0020
Shmt2	0.0000	1.2142	0.0063
Shroom3	0.0000	1.1474	0.0112
Shroom4	0.0000	1.2029	0.0180
Siah3	0.0000	-2.4017	0.0214
Sidt1	0.0002	-2.5450	0.0482
Sigirr	0.0000	3.8823	0.0147
Sim1	0.0000	-5.8958	0.0117
Sirpa	0.0000	-1.2048	0.0116
Sirt1	0.0000	1.1980	0.0110
Sirt2	0.0000	-1.0018	0.0176
Sirt5	0.0000	1.2219	0.0312
Ska1	0.0000	1.0252	0.0342
Ska3	0.0000	1.3897	0.0140
Skap2	0.0000	-1.1322	0.0158
Slc10a4	0.0000	-2.8716	0.0269
Slc11a2	0.0000	-1.1735	0.0156
Slc12a5	0.0000	-2.8503	0.0026
Slc12a8	0.0000	3.9877	0.0108
Slc13a5	0.0000	3.2936	0.0208
Slc15a1	0.0000	4.3441	0.0218
Slc16a10	0.0000	3.2892	0.0032
Slc16a2	0.0000	1.9416	0.0055
Slc16a4	0.0000	2.0407	0.0391
Slc16a6	0.0000	1.5884	0.0068
Slc18a3	0.0000	-3.2538	0.0124
Slc18b1	0.0000	2.0542	0.0138
Slc19a3	0.0000	2.6797	0.0348
Slc1a2	0.0000	-1.4794	0.0065
Slc1a3	0.0000	-1.4340	0.0055
Slc1a4	0.0000	1.0095	0.0137
Slc1a5	0.0000	1.6327	0.0078
Slc20a2	0.0000	1.1990	0.0101
Slc22a15	0.0000	-2.1234	0.0175
Slc22a17	0.0000	-1.1412	0.0092
Slc22a3	0.0000	-2.7602	0.0422
Slc22a6	0.0000	-2.6330	0.0387
Slc24a3	0.0000	-1.6917	0.0179
Slc24a4	0.0000	-3.0886	0.0407
Slc25a13	0.0000	2.3745	0.0026
Slc25a15	0.0000	1.1276	0.0149
Slc25a27	0.0000	-1.5011	0.0192
Slc25a31	0.0000	8.2256	0.0041
Slc25a43	0.0000	4.5309	0.0364
Slc25a45	0.0001	2.8969	0.0468

Slc25a5	0.0000	1.2380	0.0046
Slc27a2	0.0000	2.7324	0.0200
Slc29a1	0.0000	2.5127	0.0021
Slc29a4	0.0000	-1.2910	0.0137
Slc2a1	0.0000	1.0132	0.0066
Slc2a10	0.0000	-2.3224	0.0359
Slc2a12	0.0000	1.2661	0.0281
Slc2a3	0.0000	1.9917	0.0008
Slc2a5	0.0000	3.4727	0.0410
Slc30a10	0.0000	-1.7058	0.0361
Slc30a3	0.0000	-2.1024	0.0250
Slc32a1	0.0000	-2.4330	0.0124
Slc35d2	0.0000	2.2878	0.0361
Slc35e4	0.0000	-1.0501	0.0294
Slc35f2	0.0000	3.1516	0.0026
Slc35f4	0.0001	-1.6977	0.0460
Slc35g1	0.0000	1.0850	0.0332
Slc36a4	0.0000	-1.0749	0.0182
Slc37a2	0.0000	1.6087	0.0278
Slc37a4	0.0000	-1.4285	0.0153
Slc38a3	0.0000	-2.5840	0.0164
Slc38a6	0.0000	13.0195	0.0000
Slc38a7	0.0000	1.0394	0.0167
Slc39a12	0.0000	3.4723	0.0096
Slc39a4	0.0000	3.4094	0.0060
Slc3a2	0.0000	1.6557	0.0020
Slc40a1	0.0000	1.9211	0.0097
Slc43a3	0.0003	1.2817	0.0489
Slc44a1	0.0000	1.8772	0.0217
Slc45a2	0.0000	4.9777	0.0409
Slc45a3	0.0000	1.5494	0.0296
Slc47a1	0.0000	7.9322	0.0051
Slc4a10	0.0000	2.7564	0.0130
Slc4a11	0.0000	3.3899	0.0115
Slc4a4	0.0000	-1.0147	0.0320
Slc4a5	0.0000	7.2530	0.0024
Slc4a8	0.0000	-1.3552	0.0115
Slc50a1	0.0000	2.9886	0.0132
Slc52a3	0.0000	4.4502	0.0262
Slc5a1	0.0000	3.7106	0.0331
Slc5a11	0.0000	3.4454	0.0214
Slc6a1	0.0000	-2.7412	0.0019
Slc6a11	0.0000	-4.7278	0.0072
Slc6a13	0.0000	-2.8430	0.0156
Slc6a17	0.0000	-1.6023	0.0285
Slc6a5	0.0000	-4.8393	0.0252
Slc7a1	0.0000	1.6240	0.0036
Slc7a14	0.0000	-2.1119	0.0145
Slc7a2	0.0000	-2.9068	0.0045

Slc7a3	0.0000	3.6509	0.0006
Slc7a5	0.0000	1.3752	0.0036
Slc7a7	0.0000	3.1165	0.0043
Slc7a8	0.0000	1.3853	0.0334
Slc8a1	0.0000	-1.2176	0.0140
Slc8a3	0.0000	-1.1260	0.0322
Slc9a3r1	0.0000	1.2511	0.0109
Slco1c1	0.0000	6.8421	0.0230
Slco2a1	0.0003	-1.2393	0.0490
Slco4c1	0.0000	3.0724	0.0184
Slco5a1	0.0000	-1.2572	0.0110
Slfn5	0.0004	4.2613	0.0498
Slit1	0.0000	-2.4584	0.0008
Slit2	0.0000	-1.3235	0.0048
Slit3	0.0000	-1.0089	0.0246
Slitrk1	0.0000	-2.0802	0.0174
Slitrk2	0.0000	-2.3340	0.0205
Slitrk3	0.0000	-2.8692	0.0188
Slitrk4	0.0000	-3.2271	0.0236
Slitrk5	0.0000	-1.1214	0.0193
Smad9	0.0001	-1.8751	0.0464
Smagp	0.0000	3.5461	0.0093
Smap2	0.0000	-1.0590	0.0138
Smarca5	0.0000	1.1686	0.0037
Smarcc2	0.0000	-1.1134	0.0096
Smc1b	0.0000	2.8921	0.0096
Smc2	0.0000	1.7471	0.0022
Smc4	0.0000	1.0104	0.0092
Smim18	0.0000	-4.6399	0.0200
Smim3	0.0000	1.7050	0.0334
Smoc2	0.0000	-4.3288	0.0055
Smpd4	0.0000	1.0684	0.0148
Smtnl2	0.0000	2.4484	0.0033
Smyd1	0.0001	1.5015	0.0470
Smyd4	0.0000	1.2378	0.0235
Snai1	0.0000	-1.9322	0.0234
Snai2	0.0000	-2.2485	0.0289
Snai3	0.0000	5.2461	0.0361
Snap25	0.0000	-1.8402	0.0057
Snap91	0.0000	-1.7213	0.0085
Snca	0.0000	-1.9653	0.0124
Sncaip	0.0000	-2.1957	0.0063
Sncb	0.0000	-2.7277	0.0179
Sncg	0.0000	-1.8876	0.0082
Sned1	0.0000	-2.4845	0.0079
Snhg16	0.0000	-1.2671	0.0386
Snhg18	0.0000	-1.9601	0.0408
Snhg5	0.0000	-1.2524	0.0159
Snn	0.0000	-1.5737	0.0037

Snph	0.0000	-1.6337	0.0282
Snrpd1	0.0000	1.0516	0.0098
Snrpf	0.0000	1.1281	0.0123
Snrpg	0.0000	2.7801	0.0023
Sntb1	0.0000	1.0676	0.0401
Snx2	0.0000	1.0917	0.0097
Snx20	0.0000	2.8331	0.0324
Snx21	0.0000	-1.4506	0.0281
Snx22	0.0000	-3.8141	0.0091
Snx32	0.0000	-1.4156	0.0352
Soat2	0.0000	2.7733	0.0429
Sobp	0.0000	-1.6667	0.0064
Sod1	0.0000	1.3128	0.0039
Sod3	0.0000	-3.6000	0.0230
Soga3	0.0000	-1.2981	0.0070
Sorbs1	0.0000	-1.0742	0.0232
Sorcs2	0.0000	-1.8074	0.0056
Sost	0.0000	4.6917	0.0128
Sowahb	0.0000	2.0552	0.0381
Sox11	0.0000	-1.8010	0.0022
Sox3	0.0000	-1.3149	0.0215
Sox4	0.0000	-1.9303	0.0015
Sox5	0.0000	-1.0586	0.0329
Sox8	0.0000	-2.4409	0.0208
Sox9	0.0000	-1.3114	0.0121
Sp5	0.0000	3.3100	0.0090
Sp8	0.0000	-2.4723	0.0087
Sp9	0.0000	-4.5569	0.0228
Spag1	0.0000	1.3091	0.0339
Spag16	0.0000	4.1490	0.0156
Spag4	0.0000	-2.2098	0.0343
Spag9	0.0000	-1.3579	0.0029
Sparcl1	0.0000	-2.0158	0.0197
Spata5	0.0000	1.1341	0.0133
Spata6	0.0000	1.0658	0.0240
Spc24	0.0000	1.2483	0.0135
Spef2	0.0000	2.3632	0.0273
Speg	0.0000	-1.3599	0.0122
Spesp1	0.0000	2.5883	0.0401
Sphkap	0.0000	-3.0664	0.0180
Spidr	0.0000	1.3510	0.0087
Spint2	0.0000	2.9655	0.0010
Spire1	0.0000	-1.1411	0.0106
Spn	0.0000	2.5504	0.0311
Spns2	0.0000	-1.6432	0.0214
Spock1	0.0000	-1.8258	0.0065
Spock2	0.0000	-2.0819	0.0013
Spon1	0.0000	-4.8117	0.0003
Spon2	0.0000	-2.2056	0.0182

Spopl	0.0000	-1.0168	0.0167
Spp1	0.0000	3.1807	0.0005
Sprn	0.0002	3.5578	0.0478
Spry2	0.0000	1.2834	0.0078
Spry4	0.0000	1.9860	0.0158
Spsb1	0.0000	-1.4823	0.0210
Spsb4	0.0000	-2.2773	0.0051
Sptbn2	0.0000	-1.7457	0.0026
Srcin1	0.0000	-1.4726	0.0089
Srd5a1	0.0000	-1.9532	0.0159
Srgap3	0.0000	-1.4438	0.0036
Srgn	0.0001	-1.0607	0.0469
Srpx	0.0000	2.5159	0.0220
Srpx2	0.0001	-3.0114	0.0476
Srr	0.0000	-1.2532	0.0139
Srrm3	0.0000	-1.2681	0.0216
Srrm4	0.0000	-1.8898	0.0054
Ss18l1	0.0000	-1.7890	0.0049
Ssbp1	0.0000	1.3135	0.0120
Ssbp4	0.0000	1.4296	0.0062
Ssc5d	0.0000	-1.5946	0.0150
Sst	0.0000	-1.6142	0.0166
Sstr1	0.0000	-4.2706	0.0301
Sstr2	0.0000	-1.8239	0.0217
Sstr3	0.0000	-4.7985	0.0362
Sstr4	0.0000	-4.5695	0.0321
St14	0.0000	2.5589	0.0037
St18	0.0000	-1.8974	0.0069
St3gal1	0.0000	-2.3223	0.0064
St6gal2	0.0000	-1.8191	0.0143
St6galnac2	0.0000	2.3244	0.0421
St6galnac3	0.0000	-1.5196	0.0113
St6galnac5	0.0000	-3.0959	0.0034
St8sia1	0.0000	-1.5288	0.0089
St8sia2	0.0000	-1.2467	0.0064
St8sia3	0.0000	-1.1545	0.0237
St8sia4	0.0000	-1.5972	0.0102
St8sia5	0.0000	-3.0335	0.0343
St8sia6	0.0000	4.5522	0.0110
Stac2	0.0000	-1.2707	0.0347
Stag2	0.0000	1.0596	0.0063
Stag3	0.0000	1.8685	0.0058
Stard9	0.0000	-1.4645	0.0205
Stat1	0.0000	1.4730	0.0113
Stat4	0.0000	4.7120	0.0142
Stc2	0.0000	1.7093	0.0070
Steap1	0.0000	2.9097	0.0161
Steap3	0.0000	2.6421	0.0175
Steap4	0.0000	4.6465	0.0254

Stk17b	0.0000	1.9473	0.0048
Stk32b	0.0000	-2.4148	0.0129
Stmn2	0.0000	-1.1042	0.0044
Stmn3	0.0000	-1.4421	0.0050
Stra6	0.0000	-2.4705	0.0145
Stx1a	0.0000	-1.2311	0.0261
Stx1b	0.0000	-1.7720	0.0072
Stx3	0.0000	2.2332	0.0033
Stxbp1	0.0000	-2.0779	0.0015
Stxbp2	0.0000	2.9427	0.0022
Stxbp3	0.0000	1.1293	0.0233
Stxbp5l	0.0000	-2.1740	0.0392
Stxbp6	0.0000	-1.6504	0.0095
Styk1	0.0000	3.4533	0.0278
Suclg2	0.0000	1.4215	0.0060
Sulf2	0.0000	-1.3347	0.0042
Sun2	0.0000	-1.0675	0.0139
Susd3	0.0000	3.6841	0.0387
Susd5	0.0000	-3.1072	0.0116
Sv2a	0.0000	-1.7088	0.0060
Sv2c	0.0000	-3.1628	0.0049
Svep1	0.0000	-3.4481	0.0082
Svil	0.0000	-1.0951	0.0202
Svop	0.0000	-2.1710	0.0042
Swap70	0.0000	1.0698	0.0147
Syce1	0.0000	5.1207	0.0096
Sycp1	0.0000	2.7169	0.0197
Sycp2	0.0000	4.9530	0.0225
Sycp3	0.0000	2.6167	0.0090
Syk	0.0000	3.5337	0.0014
Syn2	0.0000	-1.2777	0.0261
Syn3	0.0000	-1.4587	0.0347
Syndig1l	0.0000	-2.5183	0.0372
Syne1	0.0000	1.4719	0.0106
Syngr3	0.0000	-1.3380	0.0230
Synm	0.0000	-1.3960	0.0153
Synpo	0.0000	-2.3436	0.0068
Synpo2	0.0000	-2.3739	0.0119
Synpr	0.0000	-2.2909	0.0133
Syt1	0.0000	-2.0890	0.0030
Syt11	0.0000	-1.4541	0.0030
Syt16	0.0000	-1.6329	0.0313
Syt2	0.0000	-2.2197	0.0186
Syt3	0.0002	-1.1763	0.0486
Syt4	0.0000	-1.4098	0.0060
Syt6	0.0000	-1.2720	0.0442
Syt9	0.0000	2.6469	0.0054
Sytl2	0.0000	2.3931	0.0167
Sytl3	0.0002	3.5525	0.0485

Tacr1	0.0000	-2.8844	0.0059
Tada2a	0.0000	1.0146	0.0173
Taf4b	0.0000	3.0531	0.0060
Taf5l	0.0000	1.2787	0.0069
Taf7	0.0000	1.0142	0.0105
Taf7l	0.0000	5.5113	0.0058
Tafa2	0.0000	-1.7840	0.0312
Tagln2	0.0000	1.5706	0.0042
Tagln3	0.0000	-1.8800	0.0021
Tal1	0.0000	-3.7173	0.0038
Tanc1	0.0000	1.3290	0.0061
Tanc2	0.0000	-1.6008	0.0050
Tango6	0.0000	1.3471	0.0139
Tap2	0.0000	1.0859	0.0364
Tarbp1	0.0000	1.3566	0.0170
Tars	0.0000	1.1990	0.0057
Tars2	0.0000	1.0575	0.0201
Tbc1d16	0.0000	-1.5892	0.0023
Tbc1d2	0.0000	1.9157	0.0206
Tbc1d25	0.0000	1.3053	0.0177
Tbc1d30	0.0000	-1.2804	0.0221
Tbkbp1	0.0000	-1.2722	0.0181
Tbrg1	0.0000	1.1263	0.0063
Tbx15	0.0000	-1.9078	0.0187
Tbx18	0.0000	-3.1735	0.0348
Tbx2	0.0000	-2.9939	0.0215
Tbx3	0.0000	1.8057	0.0064
Tcaf1	0.0000	-1.3153	0.0031
Tcea1	0.0000	1.4357	0.0038
Tcea3	0.0000	4.3812	0.0030
Tceal1	0.0000	-1.0471	0.0373
Tcf15	0.0000	1.1970	0.0337
Tcf24	0.0000	2.8471	0.0233
Tcf4	0.0000	-1.3159	0.0054
Tcf7	0.0000	2.4603	0.0034
Tcof1	0.0000	1.3424	0.0043
Tcp11l2	0.0000	-2.5404	0.0121
Tcta	0.0000	-1.3672	0.0172
Tdg	0.0000	2.1568	0.0019
Tdrd1	0.0000	2.8998	0.0368
Tdrd12	0.0000	3.8989	0.0031
Tdrd5	0.0000	3.6587	0.0249
Tdrd9	0.0003	2.1013	0.0496
Tdrp	0.0000	3.6377	0.0013
Tead3	0.0000	-1.0257	0.0292
Tead4	0.0000	2.6108	0.0092
Tefm	0.0000	1.0061	0.0256
Tekt1	0.0000	5.9899	0.0204
Tenm1	0.0000	-2.4648	0.0189

Tent5a	0.0000	-2.2586	0.0132
Tent5c	0.0000	2.4608	0.0206
Tep1	0.0000	1.0777	0.0283
Tesk1	0.0000	-1.1388	0.0140
Tesk2	0.0000	1.7195	0.0181
Tet3	0.0000	-1.3108	0.0056
Tex14	0.0000	4.9601	0.0016
Tex15	0.0000	2.9355	0.0170
Tex30	0.0000	1.0686	0.0268
Tfap2a	0.0000	1.2914	0.0245
Tfcp2	0.0000	-1.0040	0.0167
Tfcp2l1	0.0000	5.2162	0.0004
Tfdp1	0.0000	1.2232	0.0058
Tfe3	0.0000	1.3995	0.0052
Tgfa	0.0000	-1.6458	0.0298
Tgfb1i1	0.0000	-1.9693	0.0180
Tgfbi	0.0001	-1.2266	0.0474
Tgfbr2	0.0000	-1.8984	0.0115
Tgif1	0.0000	1.3636	0.0072
Tgm1	0.0000	3.8817	0.0163
Tgm2	0.0000	-2.8074	0.0031
Tgm3	0.0000	3.3399	0.0238
Th	0.0002	1.0365	0.0479
Thbd	0.0000	-1.6091	0.0312
Thbs4	0.0000	4.7168	0.0328
Thnsl2	0.0000	1.7353	0.0323
Thra	0.0000	-1.5923	0.0045
Thsd7a	0.0000	-1.0980	0.0137
Tifa	0.0000	-3.6829	0.0182
Timm23	0.0000	1.9431	0.0028
Timp1	0.0000	4.1007	0.0033
Timp2	0.0000	-1.6534	0.0085
Timp3	0.0000	-1.2988	0.0411
Tipin	0.0000	1.1594	0.0112
Tjp2	0.0000	1.0722	0.0089
Tjp3	0.0000	1.2306	0.0286
Tk1	0.0000	1.1405	0.0248
Tktl1	0.0000	6.2700	0.0344
Tle1	0.0000	-1.0464	0.0117
Tle2	0.0000	-3.1860	0.0243
Tll2	0.0000	1.2506	0.0435
Tlr3	0.0000	4.7623	0.0271
Tlr4	0.0003	2.9827	0.0491
Tlr5	0.0000	2.5817	0.0388
Tm4sf1	0.0000	3.1815	0.0087
Tma16	0.0000	1.2240	0.0119
Tmbim1	0.0000	1.1458	0.0195
Tmc6	0.0000	2.9612	0.0113
Tmc7	0.0000	-1.8875	0.0169

Tmc8	0.0000	3.7070	0.0377
Tmcc1	0.0000	-3.3038	0.0006
Tmco4	0.0000	4.2246	0.0112
Tmed3	0.0000	-1.0637	0.0237
Tmed5	0.0000	-1.1948	0.0131
Tmeff1	0.0000	-1.0122	0.0072
Tmeff2	0.0000	-1.6754	0.0155
Tmem100	0.0000	-3.8848	0.0231
Tmem102	0.0002	1.2471	0.0484
Tmem106a	0.0000	2.7177	0.0250
Tmem119	0.0000	-2.8173	0.0089
Tmem121	0.0000	-1.0005	0.0319
Tmem130	0.0000	-1.5638	0.0119
Tmem132e	0.0000	-3.5201	0.0040
Tmem141	0.0000	-1.4210	0.0418
Tmem144	0.0000	2.1494	0.0298
Tmem14a	0.0000	1.2884	0.0280
Tmem151b	0.0000	-1.2324	0.0126
Tmem154	0.0000	2.7342	0.0428
Tmem169	0.0000	-1.5037	0.0137
Tmem170b	0.0000	-1.5521	0.0072
Tmem178b	0.0000	-1.1530	0.0188
Tmem179	0.0000	-1.5930	0.0293
Tmem185b	0.0000	1.0746	0.0201
Tmem192	0.0000	1.2710	0.0161
Tmem198	0.0000	-1.7272	0.0166
Tmem200b	0.0001	-2.3805	0.0462
Tmem204	0.0000	-2.6375	0.0449
Tmem229b	0.0000	-1.5676	0.0097
Tmem231	0.0000	1.0468	0.0160
Tmem246	0.0000	-1.0810	0.0214
Tmem25	0.0000	-1.7075	0.0387
Tmem26	0.0001	1.5512	0.0471
Tmem266	0.0000	-1.4182	0.0365
Tmem30a	0.0000	-1.0601	0.0090
Tmem37	0.0000	2.1686	0.0211
Tmem44	0.0000	-2.7231	0.0025
Tmem51	0.0000	1.1728	0.0397
Tmem59l	0.0000	1.7063	0.0319
Tmem63a	0.0000	1.2667	0.0345
Tmem63c	0.0004	-1.8923	0.0500
Tmem65	0.0000	-1.0361	0.0119
Tmem74b	0.0000	-1.3668	0.0431
Tmem86a	0.0000	-1.4968	0.0260
Tmem88	0.0000	1.7210	0.0349
Tmem8b	0.0000	-1.2292	0.0100
Tmem92	0.0000	2.4549	0.0276
Tmem98	0.0000	-1.1193	0.0275
Tmod2	0.0000	-2.1753	0.0024

Tmtc1	0.0000	-1.9376	0.0136
Tmtc2	0.0000	-1.4774	0.0098
Tmtc4	0.0000	-1.2312	0.0122
Tnfaip3	0.0000	-1.1228	0.0354
Tnfaip6	0.0002	-1.2865	0.0484
Tnfrsf11b	0.0002	-2.5503	0.0486
Tnfrsf12a	0.0000	1.5209	0.0205
Tnfrsf13b	0.0001	4.6180	0.0468
Tnfrsf1b	0.0000	2.3968	0.0245
Tnfrsf21	0.0000	-1.2753	0.0124
Tnfsf15	0.0001	-4.3266	0.0462
Tnip1	0.0000	1.2513	0.0128
Tnnt1	0.0000	1.8145	0.0308
Tnnt2	0.0000	-2.6345	0.0308
Tnr	0.0000	-1.1520	0.0404
Tns1	0.0000	-1.3881	0.0074
Tnxb	0.0000	-3.1928	0.0351
Tom1	0.0000	2.1934	0.0056
Tom1l2	0.0000	-1.3126	0.0100
Tomm20	0.0000	1.0436	0.0049
Top2a	0.0000	1.1345	0.0090
Tor3a	0.0000	2.4330	0.0087
Tor4a	0.0000	1.3775	0.0370
Tox	0.0000	-1.2393	0.0200
Tox2	0.0000	-3.4174	0.0021
Tpbg	0.0000	1.0204	0.0188
Tpd52	0.0000	1.0020	0.0112
Tpd52l1	0.0000	1.9634	0.0093
Tpm3	0.0000	1.1164	0.0055
Tpmt	0.0000	-1.1861	0.0215
Tppp	0.0000	-1.0276	0.0401
Tppp3	0.0000	-1.7707	0.0049
Tpr	0.0000	1.2239	0.0039
Tpt1	0.0000	1.3007	0.0023
Traf3ip2	0.0000	1.2605	0.0332
Trafd1	0.0000	-1.1952	0.0122
Traip	0.0000	1.1601	0.0203
Tram1	0.0000	1.3536	0.0055
Trap1	0.0000	1.0942	0.0090
Trappc6a	0.0000	-1.1120	0.0242
Trdmt1	0.0000	1.1379	0.0353
Trhde	0.0000	-2.3930	0.0304
Trib2	0.0000	-1.2979	0.0084
Trib3	0.0000	2.8893	0.0044
Tril	0.0000	-1.3614	0.0156
Trim25	0.0000	2.6051	0.0027
Trim36	0.0000	-1.6110	0.0086
Trim46	0.0000	-1.2516	0.0214
Trim47	0.0000	1.4255	0.0385

Trim52	0.0000	5.5141	0.0272
Trim6	0.0000	3.0952	0.0011
Trim62	0.0000	-2.7187	0.0042
Trim66	0.0000	5.7376	0.0010
Trim67	0.0000	-1.2851	0.0114
Trim71	0.0000	2.0622	0.0247
Trim9	0.0000	-1.2608	0.0118
Triml2	0.0000	4.1175	0.0020
Trio	0.0000	-1.1338	0.0096
Trip10	0.0000	1.6423	0.0071
Trip13	0.0000	1.6346	0.0067
Trmt11	0.0000	1.2191	0.0235
Trnp1	0.0000	-1.5711	0.0399
Tro	0.0000	-1.0290	0.0096
Troap	0.0000	1.1889	0.0240
Trpc1	0.0000	-1.1480	0.0338
Trpc6	0.0000	5.4207	0.0133
Trpm2	0.0000	4.3949	0.0104
Trpm3	0.0000	4.7998	0.0016
Tsen15	0.0000	1.7772	0.0127
Tshz1	0.0000	-3.0606	0.0015
Tshz2	0.0000	-4.1201	0.0066
Tshz3	0.0000	-3.0511	0.0029
Tspan1	0.0003	3.8605	0.0493
Tspan11	0.0000	-2.7172	0.0070
Tspan17	0.0000	-1.7101	0.0159
Tspan2	0.0000	-1.7345	0.0145
Tspo	0.0000	2.6999	0.0149
Tspyl4	0.0000	-1.4161	0.0044
Tsr1	0.0000	1.1396	0.0074
Tst	0.0000	1.5767	0.0255
Ttbk2	0.0000	-1.5401	0.0044
Ttc21a	0.0000	1.9585	0.0371
Ttc28	0.0000	-1.1850	0.0096
Ttc39b	0.0000	2.4045	0.0037
Ttc6	0.0000	-2.8595	0.0308
Ttc9b	0.0000	-1.6776	0.0228
Ttl	0.0000	-1.1541	0.0155
Ttll6	0.0000	2.6726	0.0388
Ttll7	0.0000	-1.4742	0.0286
Ttn	0.0000	1.4902	0.0411
Ttpa	0.0000	3.6197	0.0100
Ttpal	0.0000	-1.4402	0.0126
Ttr	0.0000	7.7546	0.0003
Ttyh1	0.0000	-2.0057	0.0019
Tub	0.0000	-1.9674	0.0031
Tuba1a	0.0000	-1.4898	0.0021
Tuba4a	0.0000	3.6436	0.0017
Tubb2b	0.0000	-1.5961	0.0013

Tufm	0.0000	1.1568	0.0113
Tuft1	0.0000	1.6971	0.0210
Tulp2	0.0000	4.1625	0.0203
Tulp4	0.0000	-1.3299	0.0056
Twist2	0.0000	-5.0390	0.0173
Txlnb	0.0000	1.3179	0.0253
Txndc16	0.0000	-1.0363	0.0163
Tyms	0.0000	1.8804	0.0038
U2af1	0.0000	3.0763	0.0007
Uap1l1	0.0000	1.6740	0.0064
Uba2	0.0000	1.0015	0.0069
Uba52	0.0000	4.1900	0.0003
Ubap1l	0.0000	2.5489	0.0371
Ubash3b	0.0000	-2.2488	0.0125
Ube2c	0.0000	1.4584	0.0073
Ube2i	0.0000	1.0260	0.0098
Ube2ql1	0.0000	-1.3939	0.0153
Ube2t	0.0000	1.2203	0.0229
Ube2u	0.0000	3.9892	0.0406
Ubn2	0.0000	-1.0320	0.0100
Ubxn10	0.0000	3.3512	0.0135
Ubxn2a	0.0000	1.2998	0.0108
Ubxn8	0.0000	1.1179	0.0235
Uchl3	0.0000	1.9115	0.0043
Uchl5	0.0000	1.6348	0.0052
Ucp2	0.0000	1.4769	0.0050
Uhrf1	0.0000	1.7599	0.0054
Ulk2	0.0000	-1.0797	0.0129
Umad1	0.0000	-1.1617	0.0237
Umps	0.0000	1.4623	0.0080
Unc13c	0.0004	-2.0095	0.0496
Unc13d	0.0000	3.4752	0.0187
Unc5a	0.0000	-2.5573	0.0092
Unc5c	0.0000	1.1517	0.0250
Unc5d	0.0000	-1.7760	0.0177
Unc79	0.0000	-1.2307	0.0246
Ung	0.0000	2.3300	0.0021
Upp1	0.0000	3.7472	0.0006
Upp2	0.0000	-1.7261	0.0445
Uqcrb	0.0000	1.5045	0.0076
Urb1	0.0000	1.0906	0.0168
Usp1	0.0000	1.1022	0.0077
Usp13	0.0000	-1.0419	0.0298
Usp18	0.0000	2.2371	0.0333
Usp26	0.0000	3.9122	0.0282
Usp39	0.0000	1.2008	0.0110
Usp43	0.0000	1.5927	0.0422
Usp50	0.0000	-4.4552	0.0155
Usp9x	0.0000	1.6804	0.0013

Usp9y	0.0000	4.3733	0.0397
Ust	0.0000	-1.1667	0.0324
Utp20	0.0000	1.4136	0.0045
Utp4	0.0000	1.8274	0.0028
Utrn	0.0000	1.2054	0.0061
Uxs1	0.0000	1.5754	0.0085
Vamp1	0.0000	-1.0045	0.0415
Vamp8	0.0000	1.6355	0.0189
Vash1	0.0000	-1.3267	0.0108
Vash2	0.0000	-2.0560	0.0038
Vat1l	0.0000	1.6577	0.0047
Vav3	0.0000	-1.7125	0.0114
Vcam1	0.0000	1.1560	0.0156
Vcan	0.0000	-1.9919	0.0011
Vcp	0.0000	1.0009	0.0063
Vdac3	0.0000	1.6458	0.0020
Vegfc	0.0000	1.7242	0.0251
Vezt	0.0000	-1.5286	0.0066
Vgf	0.0000	1.5390	0.0280
Vil1	0.0000	3.2448	0.0402
Vill	0.0000	1.0998	0.0397
Vit	0.0000	-3.8927	0.0062
Vopp1	0.0000	-1.7568	0.0022
Vps37d	0.0000	-1.6345	0.0119
Vrk2	0.0000	1.1577	0.0316
Vsir	0.0000	-1.9336	0.0320
Vsnl1	0.0000	-3.0024	0.0156
Vstm2a	0.0000	-2.0447	0.0239
Vstm2b	0.0000	-1.4875	0.0244
Vstm2l	0.0000	-1.7339	0.0116
Vstm4	0.0000	-3.4838	0.0103
Vsx1	0.0000	-4.2339	0.0366
Vwa3a	0.0000	2.5031	0.0334
Vwa3b	0.0000	4.6786	0.0052
Vwa5b1	0.0000	1.8180	0.0299
Vwa8	0.0000	1.0242	0.0223
Vwf	0.0000	2.8703	0.0239
Was	0.0000	3.6925	0.0350
Wbp1	0.0000	-1.3493	0.0068
Wbp2nl	0.0000	4.1719	0.0416
Wdr12	0.0000	1.3654	0.0075
Wdr31	0.0000	2.8025	0.0058
Wdr35	0.0000	1.2832	0.0064
Wdr36	0.0000	1.0038	0.0103
Wdr47	0.0000	-1.2357	0.0086
Wdr6	0.0000	-1.1082	0.0046
Wdr62	0.0000	1.4911	0.0152
Wdr63	0.0000	3.6697	0.0263
Wdr75	0.0000	1.4116	0.0055

Wdr76	0.0000	1.1583	0.0203
Wdr86	0.0000	1.2093	0.0398
Wdr89	0.0000	-3.3858	0.0004
Wee1	0.0000	1.2334	0.0111
Wfdc1	0.0001	3.1476	0.0465
Wfdc2	0.0000	4.0973	0.0083
Wfikkn2	0.0003	1.0281	0.0493
Wls	0.0000	1.1781	0.0098
Wnk2	0.0000	-1.2754	0.0076
Wnk4	0.0000	-1.4382	0.0315
Wnt10b	0.0000	5.0116	0.0167
Wnt11	0.0000	-2.3308	0.0171
Wnt2b	0.0000	2.3010	0.0196
Wnt3	0.0000	2.8278	0.0067
Wnt4	0.0000	-2.7368	0.0072
Wnt5a	0.0000	-2.1326	0.0039
Wnt6	0.0000	-2.0728	0.0178
Wnt7a	0.0000	-4.5783	0.0023
Wnt7b	0.0000	-2.2317	0.0269
Wnt8a	0.0002	2.7946	0.0486
Wnt9a	0.0000	2.6222	0.0028
Wrn	0.0000	1.0208	0.0260
Wscd2	0.0000	-2.5236	0.0044
Wtip	0.0000	1.2423	0.0212
Xaf1	0.0000	3.4164	0.0086
Xk	0.0000	1.5375	0.0254
Xkr4	0.0000	-1.0677	0.0284
Xkr7	0.0000	-1.2091	0.0318
Xpo1	0.0000	1.0017	0.0062
Xpot	0.0000	1.1187	0.0069
Xrcc5	0.0000	2.2471	0.0009
Xylb	0.0000	1.3671	0.0309
Ybx3	0.0000	1.3840	0.0036
Yif1b	0.0000	1.0585	0.0151
Yjefn3	0.0000	1.2601	0.0418
Ypel4	0.0000	-2.3537	0.0170
Ypel5	0.0000	-1.1296	0.0117
Zadh2	0.0000	-1.6964	0.0089
Zap70	0.0000	4.9451	0.0229
Zbtb16	0.0000	-1.9758	0.0171
Zbtb20	0.0000	-1.0348	0.0169
Zbtb32	0.0000	4.7906	0.0217
Zbtb44	0.0000	1.0773	0.0116
Zbtb46	0.0000	-1.9270	0.0091
Zbtb8os	0.0000	1.2170	0.0219
Zc3h12a	0.0001	1.4209	0.0469
Zc3h12b	0.0000	-1.3276	0.0451
Zc3h8	0.0000	1.0596	0.0377
Zc3hav1	0.0000	2.5712	0.0024

Zc3hav1l	0.0000	-1.0347	0.0150
Zc4h2	0.0000	-1.7879	0.0050
Zcchc10	0.0000	1.1474	0.0242
Zcchc12	0.0000	1.1240	0.0093
Zcchc24	0.0000	-2.1759	0.0045
Zcwpw1	0.0000	1.0999	0.0279
Zdhhc14	0.0000	-1.7548	0.0327
Zdhhc23	0.0000	1.3253	0.0425
Zeb1	0.0000	-1.6312	0.0030
Zfhx3	0.0000	-1.6270	0.0043
Zfp14	0.0000	-1.5825	0.0240
Zfp28	0.0000	-1.0039	0.0326
Zfp30	0.0000	-1.2354	0.0175
Zfp41	0.0000	-1.3939	0.0056
Zfpm2	0.0001	-1.1370	0.0470
Zfr2	0.0000	-1.5789	0.0176
Zhx2	0.0000	-1.4637	0.0132
Zic2	0.0000	1.7540	0.0069
Zic3	0.0000	3.1720	0.0006
Zic5	0.0000	2.7603	0.0017
Zmat4	0.0000	1.3363	0.0218
Zmiz1	0.0000	-1.5357	0.0035
Zmym1	0.0000	1.1233	0.0140
Zmym6	0.0000	-1.1308	0.0129
Zmynd10	0.0000	1.8986	0.0407
Zranb3	0.0000	1.5107	0.0080
Zswim5	0.0000	-1.6411	0.0060
Zswim7	0.0000	1.1673	0.0322
Zwilch	0.0000	1.6279	0.0062

Supplementary Table 3. Disease ontology (DisGeNet) analyses of genes with significantly altered transcript levels in untreated *Tet/Tet* neurons

Disorder/Disease	Overlap	Bejamini-Hochberg Adjusted P-value
Alzheimer's Disease	493/1982	9.00E-23
Schizophrenia	468/1923	1.61E-19
Glioma	515/2211	1.19E-17
Neuroblastoma	415/1698	1.67E-17
Central neuroblastoma	401/1655	3.45E-16
Breast Carcinoma	987/4963	8.47E-14
Neoplasm Metastasis	805/3920	8.47E-14
Glioblastoma	443/1937	1.43E-13
Malignant neoplasm of breast	999/5054	2.29E-13
Colorectal Cancer	691/3298	2.37E-13
Mammary Neoplasms	525/2387	2.72E-13
Prostatic Neoplasms	367/1554	4.21E-13
Endometriosis	205/749	6.13E-13
Bipolar Disorder	222/837	1.41E-12
Colorectal Neoplasms	270/1073	1.41E-12
Carcinogenesis	809/4065	8.99E-11
Liver Cirrhosis, Experimental	208/801	9.53E-11
Depressive disorder	195/741	1.40E-10
Mental Depression	159/575	3.18E-10
Intellectual Disability	528/2503	3.36E-10
Autistic Disorder	180/677	3.86E-10
Malignant neoplasm of ovary	437/2026	1.58E-09
Adenocarcinoma	377/1712	3.49E-09
Tumor Progression	446/2090	4.42E-09
Colorectal Carcinoma	597/2931	4.71E-09
Brain Neoplasms	169/646	6.55E-09
Primary malignant neoplasm of lung	477/2268	6.74E-09
Secondary malignant neoplasm of lymph node	292/1271	7.02E-09
Adenoma	239/1000	9.62E-09
Alcoholic Intoxication, Chronic	121/424	1.33E-08
Medulloblastoma	156/590	1.41E-08
Malignant neoplasm of prostate	647/3239	1.60E-08
Epilepsy	272/1177	1.60E-08
Malignant tumor of colon	425/2001	2.07E-08
Malignant neoplasm of lung	505/2449	2.82E-08
Autism Spectrum Disorders	151/572	2.82E-08
Renal Cell Carcinoma	303/1348	2.93E-08
Carcinoma of lung	509/2476	3.60E-08
Unipolar Depression	139/517	3.76E-08
Astrocytoma	184/741	5.91E-08
Small cell carcinoma of lung	169/673	1.28E-07
Major Depressive Disorder	136/513	1.51E-07
Ovarian Carcinoma	455/2203	1.93E-07
Lung Neoplasms	266/1177	2.12E-07
Coronary heart disease	214/913	4.18E-07
Attention deficit hyperactivity disorder	113/412	4.81E-07
Carcinoma, Spindle-Cell	63/188	5.13E-07
Carcinomatosis	60/176	5.67E-07
Mammary Neoplasms, Human	126/476	6.05E-07
Colon Carcinoma	431/2091	6.57E-07
Carcinoma	69/216	8.30E-07
Anaplastic carcinoma	66/204	9.88E-07
Prostate carcinoma	615/3145	1.27E-06
Osteosarcoma of bone	236/1042	1.31E-06
Congenital Abnormality	151/607	1.56E-06
Malignant neoplasm of pancreas	384/1846	1.61E-06
Visual seizure	66/207	1.73E-06
Seizures	260/1174	1.84E-06
Malignant neoplasm of stomach	482/2398	1.97E-06
Amyotrophic Lateral Sclerosis	161/660	1.97E-06

Stomach Carcinoma	478/2378	2.24E-06
Benign Prostatic Hyperplasia	112/420	2.29E-06
Adenomatous Polyposis Coli	106/392	2.47E-06
Obesity	403/1961	2.60E-06
Generalized seizures	46/126	2.60E-06
ovarian neoplasm	214/938	2.99E-06
Undifferentiated carcinoma	72/237	3.13E-06
melanoma	490/2454	3.18E-06
Degenerative polyarthritis	221/976	3.20E-06
Solid Neoplasm	194/840	5.07E-06
Parkinson Disease	236/1064	6.46E-06
Pancreatic carcinoma	382/1869	1.07E-05
Amyloidosis	191/833	1.07E-05
Osteosarcoma	242/1103	1.07E-05
Malignant neoplasm of esophagus	160/673	1.07E-05
Mental Retardation	252/1158	1.17E-05
Hypertensive disease	280/1310	1.22E-05
Impaired cognition	159/671	1.41E-05
Cognition Disorders	70/237	1.41E-05
Seizures, Focal	45/129	1.43E-05
Esophageal carcinoma	161/685	1.96E-05
Status Epilepticus	66/221	1.96E-05
Mammary Neoplasms, Experimental	52/160	2.08E-05
Non-Small Cell Lung Carcinoma	446/2243	2.08E-05
Petit mal status	31/76	2.30E-05
Diabetes Mellitus	314/1507	2.30E-05
Epileptic drop attack	41/115	2.36E-05
Mental disorders	89/329	2.36E-05
Esophageal Neoplasms	151/637	2.49E-05
nervous system disorder	111/436	2.49E-05
Pancreatic Neoplasm	156/665	3.10E-05
Alzheimer Disease, Late Onset	72/253	3.98E-05
Presenile dementia	95/362	4.06E-05
Congenital Heart Defects	77/277	4.37E-05
Heart failure	184/815	4.37E-05
Glioblastoma Multiforme	191/854	5.16E-05
Stomach Neoplasms	187/835	6.11E-05
Endometrioma	66/229	6.81E-05
Mammary Carcinoma, Animal	47/145	7.10E-05
Animal Mammary Neoplasms	47/145	7.10E-05
Fanconi Anemia	79/290	7.10E-05
Malignant neoplasm of kidney	106/421	7.19E-05
Abnormal behavior	96/372	7.23E-05
Cleft Palate	39/112	7.76E-05
Lewy Body Disease	44/133	8.06E-05
Cardiomyopathy, Familial Idiopathic	99/388	8.31E-05
Meningioma	109/438	8.81E-05
Cardiomyopathy, Dilated	105/419	9.67E-05
Tonic - clonic seizures	53/173	9.75E-05
Anoxia	78/288	9.77E-05
leukemia	386/1941	1.17E-04
Ewings sarcoma	91/352	1.17E-04
Absence Seizures	41/123	1.39E-04
Sarcoma	151/658	1.48E-04
Myocardial Infarction	209/966	1.52E-04
Pancreatic Ductal Adenocarcinoma	159/701	1.62E-04
Acute Promyelocytic Leukemia	115/475	1.75E-04
Diabetes	264/1268	1.76E-04
Epithelioma	79/298	1.90E-04
Liver Cirrhosis	155/683	2.02E-04
Tonic Seizures	36/104	2.02E-04
Conventional (Clear Cell) Renal Cell Carcinoma	145/631	2.04E-04
Fibrosis	51/169	2.22E-04

Myocardial Ischemia	109/448	2.35E-04
Status Epilepticus, Subclinical	27/69	2.36E-04
Complex Partial Status Epilepticus	27/69	2.36E-04
Grand Mal Status Epilepticus	27/69	2.36E-04
Non-Convulsive Status Epilepticus	27/69	2.36E-04
Simple Partial Status Epilepticus	27/69	2.36E-04
Anxiety	112/464	2.46E-04
Malignant neoplasm of thyroid	120/505	2.52E-04
Pilocytic Astrocytoma	40/122	2.52E-04
Malignant neoplasm of urinary bladder	240/1144	2.58E-04
Drug Dependence	52/175	2.69E-04
Noninfiltrating Intraductal Carcinoma	76/287	2.69E-04
Colonic Neoplasms	172/778	2.77E-04
Carcinoma of bladder	243/1162	2.77E-04
Bladder Neoplasm	253/1217	2.77E-04
Atrial Premature Complexes	36/106	2.91E-04
Epithelial ovarian cancer	273/1329	2.99E-04
Thyroid carcinoma	161/721	2.99E-04
Gastrointestinal Stromal Tumors	83/323	3.37E-04
Myxoid cyst	59/209	3.60E-04
Sporadic Breast Carcinoma	48/159	3.61E-04
Hyperactive behavior	158/709	4.02E-04
Acute lymphocytic leukemia	195/907	4.03E-04
Neuroendocrine Tumors	69/257	4.06E-04
Movement Disorders	50/169	4.25E-04
Adenocarcinoma of lung (disorder)	246/1187	4.39E-04
Thyroid Neoplasm	113/476	4.39E-04
Myoclonic Seizures	47/156	4.54E-04
Mood Disorders	85/336	4.69E-04
Coronary Arteriosclerosis	167/760	4.91E-04
Short palpebral fissure	26/68	4.91E-04
Congestive heart failure	190/884	5.07E-04
Kidney Diseases	144/639	5.17E-04
Adenoid Cystic Carcinoma	58/208	5.89E-04
Endometrial Carcinoma	181/840	6.93E-04
Huntington Disease	141/627	7.01E-04
Parkinsonian Disorders	59/214	7.10E-04
Calcification of coronary artery	92/375	7.23E-04
Hereditary Nonpolyposis Colorectal Cancer	40/128	7.57E-04
Diabetic Nephropathy	128/561	8.26E-04
Paroxysmal atrial fibrillation	25/66	8.26E-04
Nonorganic psychosis	60/220	8.37E-04
Psychotic Disorders	80/317	8.58E-04
Recurrent tumor	86/347	8.74E-04
Epileptic Seizures	34/103	8.74E-04
Cleft palate, isolated	40/129	8.90E-04
Ischemic cardiomyopathy	37/116	8.97E-04
Plaque, Amyloid	63/235	8.99E-04
Aortic Aneurysm, Thoracic	33/99	8.99E-04
Brugada Syndrome (disorder)	22/55	9.21E-04
Pain	113/485	9.21E-04
Uterine Corpus Cancer	131/579	9.43E-04
idiopathic epilepsy	12/21	0.00102443
Malignant neoplasm of brain	48/166	0.001047147
Cleft Lip with or without Cleft Palate	16/34	0.001127464
Body mass index	90/370	0.001133603
Finding of body mass index	90/370	0.001133603
Cerebrovascular accident	166/768	0.001155696
Vascular Diseases	96/401	0.001199869
Senile Plaques	76/301	0.001224764
Coronary Artery Disease	205/981	0.001224764
Ehlers-Danlos Syndrome	34/105	0.001224764
Bethlem myopathy	9/13	0.001224764

Malignant Neoplasms	287/1438	0.001224764
Hypertension, Portal	33/101	0.001224764
Seizures, Auditory	33/101	0.001224764
Seizures, Clonic	33/101	0.001224764
Seizures, Sensory	33/101	0.001224764
Seizures, Somatosensory	33/101	0.001224764
Atonic Absence Seizures	33/101	0.001224764
Generalized Absence Seizures	33/101	0.001224764
Gustatory seizure	33/101	0.001224764
Jacksonian Seizure	33/101	0.001224764
Non-epileptic convulsion	33/101	0.001224764
Nonepileptic Seizures	33/101	0.001224764
Olfactory seizure	33/101	0.001224764
Single Seizure	33/101	0.001224764
Vertiginous seizure	33/101	0.001224764
Liver carcinoma	659/3593	0.001252473
Tumor Initiation	62/234	0.001297315
Diabetes Mellitus, Non-Insulin-Dependent	328/1672	0.001354495
Myopia	69/269	0.001479464
Adenocarcinoma of pancreas	86/354	0.001562184
Malignant neoplasm of endometrium	129/577	0.001652334
Drug habituation	41/138	0.001745035
Epileptic encephalopathy	108/468	0.001774153
Anxiety Disorders	94/396	0.001777231
Complex partial seizures	36/116	0.001801992
Addictive Behavior	56/208	0.001813895
Neurodevelopmental Disorders	56/208	0.001813895
Triple Negative Breast Neoplasms	139/632	0.001877802
Leukemia, Myelocytic, Acute	332/1703	0.001913369
Diabetic Retinopathy	64/247	0.001913369
Urothelial Carcinoma	66/257	0.001966785
Aortic Valve Stenosis	39/130	0.001999011
Memory impairment	68/267	0.001999079
Dysarthria	71/282	0.00204468
Middle Cerebral Artery Occlusion	40/135	0.002166851
Adenoma of large intestine	66/258	0.002184095
Malignant neoplasm of mouth	112/492	0.002221482
Drug abuse	60/229	0.002222695
Secondary malignant neoplasm of liver	115/508	0.002257624
Atrial Fibrillation	72/288	0.002257624
Cervix carcinoma	225/1105	0.002312806
Precancerous Conditions	103/446	0.002340604
FANCONI ANEMIA, COMPLEMENTATION GROUP A (disorder)	64/249	0.002340604
Osteoporosis	102/441	0.002362721
Convulsive Seizures	34/109	0.002362721
Myoclonus	44/154	0.002362721
Epilepsy, Generalized	21/55	0.002389918
Atherosclerosis	230/1134	0.00240336
Uranostaphyloschisis	69/274	0.00240336
Convulsions	36/118	0.00240336
High density lipoprotein measurement	79/324	0.002420503
Infantile Severe Myoclonic Epilepsy	15/33	0.002514578
Neurodegenerative Disorders	159/745	0.002553476
Secondary malignant neoplasm of bone	80/330	0.002648179
Cerebral Ischemia	69/275	0.002648179
Migraine Disorders	72/290	0.002666506
Substance abuse problem	44/155	0.002666506
Adenocarcinoma of lung, stage I	11/20	0.002666506
Rheumatoid Arthritis	353/1833	0.002680032
Tumor Angiogenesis	115/511	0.002682342
Bronchopulmonary Dysplasia	51/188	0.002714464
Ependymoma	38/128	0.002793518
Developmental delay (disorder)	52/193	0.002793518

Precursor Cell Lymphoblastic Leukemia Lymphoma	135/618	0.00285298
Age related macular degeneration	90/382	0.002854967
Childhood Medulloblastoma	30/93	0.002894458
Diaphragmatic Hernia	23/64	0.003026536
Gastric Adenocarcinoma	90/383	0.003116112
Poor school performance	202/985	0.003215039
Hypoplastic Left Heart Syndrome	17/41	0.003220233
Microcalcification	17/41	0.003220233
Malignant neoplasm of gastrointestinal tract	66/263	0.003488513
Idiopathic osteoarthritis	15/34	0.003523201
Other specified types of schizophrenia, unspecified	20/53	0.00384658
Nephroblastoma	87/370	0.00384658
Brain Ischemia	66/264	0.003872945
Depression, Bipolar	22/61	0.003875852
Generalized Epilepsy with Febrile Seizures Plus	8/12	0.003961122
Squamous cell carcinoma of the head and neck	192/934	0.004008108
Glaucoma	89/381	0.004050175
Diastolic blood pressure	35/117	0.004131239
Brain Diseases	63/250	0.004196305
Substance Dependence	40/140	0.004295074
Papillary thyroid carcinoma	125/571	0.004340543
Refractive Errors	18/46	0.004685289
Congenital contractural arachnodactyly	55/212	0.004822952
Mental deficiency	209/1032	0.004832732
Idiopathic generalized epilepsy	19/50	0.004832732
Red Blood Cell Count measurement	35/118	0.004832732
Congenital aneurysm of ascending aorta	21/58	0.004900016
SCHIZOPHRENIA 1 (disorder)	21/58	0.004900016
Focal glomerulosclerosis	43/155	0.004927011
Platelet hematocrit measurement	43/155	0.004927011
Lip and Oral Cavity Carcinoma	106/473	0.00523191
Ataxia	54/208	0.00523191
Liver neoplasms	260/1321	0.005391894
Squamous cell carcinoma	357/1876	0.005527171
Hemangioma	31/101	0.005527171
Drug Use Disorders	35/119	0.005592162
Borderline Personality Disorder	33/110	0.005592162
Squamous cell carcinoma of lung	69/283	0.005610739
Dull intelligence	193/947	0.00573884
Low intelligence	193/947	0.00573884
Retinal Diseases	83/355	0.005964871
Down Syndrome	106/475	0.005964871
Epilepsy, Cryptogenic	22/63	0.00600299
Desmoplastic	26/80	0.006287456
Luminal B Breast Carcinoma	26/80	0.006287456
Endometrial adenocarcinoma	37/129	0.006305238
Substance Use Disorders	40/143	0.006400697
Epilepsy, Temporal Lobe	48/181	0.006493705
Hereditary Diffuse Gastric Cancer	72/300	0.006600362
Ovarian Mucinous Adenocarcinoma	15/36	0.006650282
Neoplasms	244/1236	0.006655737
polyps	69/285	0.006666531
Organic Mental Disorders, Substance-Induced	34/116	0.006949231
Prescription Drug Abuse	34/116	0.006949231
Hyalinosis, Segmental Glomerular	13/29	0.00711518
Keratosis Follicularis	13/29	0.00711518
Intestinal Neoplasms	43/158	0.007126195
Congenital pes cavus	37/130	0.007176865
Dementia	98/436	0.00734981
Craniofacial Abnormalities	49/187	0.00734981
Skin Neoplasms	63/256	0.007372802
Tumoral calcinosis	17/44	0.00743512
Primary Focal Segmental Glomerulosclerosis	8/13	0.007483201

Renal carcinoma	110/500	0.007559496
Manic Disorder	18/48	0.007594471
Congenital absence of kidney	19/52	0.00762839
Hypotonic seizures	9/16	0.00762839
Sudden loss of muscle tone	9/16	0.00762839
Microphthalmos	56/222	0.00790551
Cardiomegaly	43/159	0.007935285
Nerve Degeneration	40/145	0.008124337
Malignant neoplasm of gallbladder	52/203	0.008516225
Congenital diaphragmatic hernia	29/95	0.008578735
Squamous cell carcinoma of esophagus	205/1023	0.008578735
Prostatic Intraepithelial Neoplasias	46/174	0.008578735
Cerebral Infarction	68/283	0.008578735
Lymphoid leukemia	54/213	0.008578735
Rhabdomyosarcoma	90/397	0.008981825
Multiple Myeloma	256/1312	0.009039571
Aura	21/61	0.009202936
Awakening Epilepsy	21/61	0.009202936
Pituitary Adenoma	70/294	0.009202936
Diaphragmatic paresis	6/8	0.009475308
Diminished diaphragmatic motion	6/8	0.009475308
gallbladder neoplasm	17/45	0.009475308
Substance-Related Disorders	35/123	0.009573697
Fibroid Tumor	73/310	0.009672871
familial atrial fibrillation	13/30	0.009672871
Persistent atrial fibrillation	13/30	0.009672871
Primary malignant neoplasm	206/1032	0.00982731
Vascular inflammations	39/142	0.009940775
Muscular Dystrophy, Duchenne	49/190	0.009940775
Glaucoma, Primary Open Angle	40/147	0.010324976
Muscle Rigidity	34/119	0.010371356
Malignant Pleural Mesothelioma	63/260	0.010396577
Ductal Carcinoma	57/230	0.010489518
Cytopenia	52/206	0.011523739
Calcinosis	18/50	0.012281014
Cirrhosis	96/433	0.012281014
Focal Clonic Seizures	7/11	0.012590723
Scleroatonic muscular dystrophy	9/17	0.012590723
Metastatic malignant neoplasm to brain	43/163	0.012770244
Neural Tube Defects	43/163	0.012770244
Secondary malignant neoplasm of lung	142/683	0.013615704
Afebrile seizure	5/6	0.013835251
MUNGAN SYNDROME	5/6	0.013835251
Acute Myeloid Leukemia (AML-M2)	34/121	0.01394351
Hydrocephalus	61/253	0.014050175
Congenital anemia	52/208	0.014209856
Heart Diseases	75/325	0.014322673
Postural instability	11/24	0.014364546
Lymphoma	253/1307	0.014451179
Radial club hand	20/59	0.014463233
Juvenile Myoclonic Epilepsy	14/35	0.014538689
Kidney Failure, Acute	72/310	0.014846104
Aplasia Cutis Congenita	50/199	0.015564298
Amyotrophic Lateral Sclerosis, Sporadic	37/136	0.015651283
Leukemogenesis	150/730	0.015781381
Gastrointestinal Neoplasms	34/122	0.015858611
Hip circumference	27/90	0.016089797
Diabetes Mellitus, Experimental	32/113	0.01648617
Alloxan Diabetes	32/113	0.01648617
Streptozotocin Diabetes	32/113	0.01648617
cervical cancer	192/965	0.01648617
Ciliary Motility Disorders	21/64	0.016688135
Cortical Dysplasia	21/64	0.016688135

Febrile Convulsions	21/64	0.016688135
High-Grade Prostatic Intraepithelial Neoplasia	24/77	0.016688135
Non-small cell lung cancer stage I	24/77	0.016688135
Duane Retraction Syndrome	10/21	0.016688135
response to risperidone	10/21	0.016688135
Sciatic Neuropathy	28/95	0.017082084
Hematologic Neoplasms	118/557	0.017507443
Meningioma, benign, no ICD-O subtype	58/241	0.018481987
Malignant tumor of cervix	177/884	0.018884518
Pre-Eclampsia	40/152	0.018897896
Kallmann Syndrome	22/69	0.01908077
Malignant neoplasm of salivary gland	22/69	0.01908077
Central Nervous System Embryonal Tumor, Not Otherwise Specified	9/18	0.019424271
Diastrophic dysplasia	9/18	0.019424271
Islet Cell Tumor	15/40	0.019424271
Motor symptoms	15/40	0.019424271
Chromosome Breakage	16/44	0.019472709
Dyskinetic syndrome	24/78	0.019749334
PARKINSON DISEASE 2, AUTOSOMAL RECESSIVE JUVENILE	28/96	0.019749334
MAJOR AFFECTIVE DISORDER 2	28/96	0.019749334
Hepatic Form of Wilson Disease	11/25	0.019904139
Spondyloepimetaphyseal disorder	11/25	0.019904139
Nephrotic Syndrome	46/182	0.020039426
Motor delay	56/232	0.020039426
Abortion, Tubal	31/110	0.020142834
Hematopoietic Neoplasms	48/192	0.020171133
Renal fibrosis	50/202	0.020232772
Hemiplegic migraine	6/9	0.020257985
Saddle nose	6/9	0.020257985
Adenocarcinoma Of Esophagus	69/299	0.02050775
Pervasive Development Disorder	35/129	0.02050775
Carcinoma, Transitional Cell	88/399	0.020858515
response to ACE inhibitor	8/15	0.021122037
Triglycerides measurement	76/336	0.021317802
Platelet distribution width measurement	33/120	0.021724313
Platelet distribution width result	33/120	0.021724313
Malignant neoplasm of colon stage IV	19/57	0.021771683
Nasopharyngeal carcinoma	158/782	0.021834861
Adult Brain Neoplasm	7/12	0.021834861
Familial Alzheimer Disease (FAD)	47/188	0.021834861
Big calvaria	52/213	0.021834861
Increased size of skull	52/213	0.021834861
Congenital clubfoot	49/198	0.021854283
Abnormality of brain morphology	22/70	0.021856177
Peripheral Arterial Diseases	34/125	0.022173835
Conduction disorder of the heart	24/79	0.022515545
Arteriosclerosis	212/1087	0.022952237
Familial (FPAH)	32/116	0.023572991
No development of motor milestones	55/229	0.023575059
Hereditary pyropoikilocytosis	14/37	0.02381597
Intelligence	52/214	0.023905421
Joint laxity	27/93	0.024150769
Hereditary Malignant Neoplasm	20/62	0.024832706
B-Cell Lymphomas	148/730	0.026013354
Increased head circumference	52/215	0.026450055
Cerebral Palsy	26/89	0.026467524
Neurofibromatosis 1	51/210	0.026467524
Global developmental delay	214/1102	0.026554265
drug response	11/26	0.027012845
Kidney Neoplasm	58/246	0.027454583
Pulmonary Hypertension	39/151	0.028131339
Mechanical Allodynia	30/108	0.028424754
Trichohepatoenteric Syndrome	62/267	0.028424754

Essential Hypertension	64/278	0.030157583
Parkinson Disease, Familial, Type 1	13/34	0.030580452
Left ventricular noncompaction cardiomyopathy	13/34	0.030580452
Vascular Neoplasms	15/42	0.030681031
Aneurysm	39/152	0.031515278
Orbital separation excessive	81/368	0.031515278
Narcolepsy	60/258	0.031823306
Alobar Holoprosencephaly	8/16	0.033074575
Familial benign neonatal epilepsy	8/16	0.033074575
psychological distress	8/16	0.033074575
Malattia Leventinese	5/7	0.033115767
Nocturnal hypoventilation	5/7	0.033115767
Endometrial Neoplasms	56/238	0.033269976
Adenomatous polyp of colon	21/68	0.03330621
Precocious Puberty	21/68	0.03330621
Immunologic Deficiency Syndromes	131/641	0.033441747
Ganglioneuroma	18/55	0.033550181
Infarction, Middle Cerebral Artery	32/119	0.034523674
Hyperglycemia	103/488	0.034581979
Familial lichen amyloidosis	39/153	0.034751626
Acute Cerebrovascular Accidents	26/91	0.035309653
Uterine Cervical Neoplasm	33/124	0.035309653
Agenesis of corpus callosum	71/317	0.035610703
tumor vasculature	34/129	0.036098102
Salivary Gland Neoplasms	24/82	0.036098102
Aplasia/Hypoplasia of the mandible	86/397	0.036098102
Uterine Fibroids	77/349	0.036098102
Bone neoplasms	22/73	0.036098102
Ectopia Lentis	11/27	0.036098102
Prelingual sensorineural hearing impairment	7/13	0.036098102
Aggressive reaction	30/110	0.036098102
Bone Density	30/110	0.036098102
ATRICHIA WITH PAPULAR LESIONS	16/47	0.036098102
Carcinoma in situ of uterine cervix	16/47	0.036098102
Chronic pain	16/47	0.036098102
Differentiated Thyroid Gland Carcinoma	36/139	0.036690012
Median cleft lip and palate	6/10	0.037134704
Motor Disorders	6/10	0.037134704
Non-small cell lung cancer metastatic	15/43	0.037198032
Ventricular arrhythmia	19/60	0.037198032
Multiple Sclerosis	213/1106	0.037373652
Disorder of macula of retina	14/39	0.03797863
Asthma	249/1313	0.038038912
Intracranial Hypertension	13/35	0.038042391
Marinesco-Sjogren syndrome	23/78	0.038242659
Holoprosencephaly	18/56	0.039508715
Carcinoma of ampulla of Vater	9/20	0.039508715
Soft skin	9/20	0.039508715
Velvety skin	9/20	0.039508715
Hirschsprung Disease	35/135	0.039892653
Neurilemmoma	35/135	0.039892653
Patent ductus arteriosus	58/251	0.039892653
Mesothelioma	83/383	0.039991411
Low set ears	66/293	0.04071942
Abnormally small eyeball	37/145	0.040722908
Decreased size of eyeball	37/145	0.040722908
Pancytopenia	31/116	0.041294331
Anaplasia	64/283	0.042181038
Micrognathism	86/400	0.042224528
Tremor	53/226	0.042448021
Chronic Obstructive Airway Disease	135/669	0.04263409
Brain Tumor, Primary	25/88	0.042748294
Nodule	33/126	0.042748294

Decreased projection of lower jaw	85/395	0.042748294
Decreased projection of mandible	85/395	0.042748294
Hypoplastic mandible condyle	85/395	0.042748294
Retrusion of lower jaw	85/395	0.042748294
Epilepsies, Partial	16/48	0.043092271
Psychotic symptom	16/48	0.043092271
Bile duct carcinoma	19/61	0.043216528
Bradykinesia	19/61	0.043216528
Glioblastoma, IDH-Wildtype	23/79	0.043459648
Polycystic Ovary Syndrome	109/526	0.043673447
Manic	26/93	0.04425949
Malignant neoplasm of liver	159/805	0.045116842
Malignant neoplasm of large intestine	107/516	0.045772651
Decreased projection of maxilla	24/84	0.045772651
Deficiency of upper jaw bones	24/84	0.045772651
Hypoplasia of the maxilla	24/84	0.045772651
Hypotrophic maxilla	24/84	0.045772651
Maxillary retrognathia	24/84	0.045772651
Retrusion of upper jaw bones	24/84	0.045772651
Hereditary non-polyposis colorectal cancer syndrome	11/28	0.045858943
Semilobar Holoprosencephaly	8/17	0.046113718
Breast Fibrocystic Disease	22/75	0.046650009
Malignant Fibrous Histiocytoma	22/75	0.046650009
Shy-Drager Syndrome	22/75	0.046650009
Giant Cell Fibroblastoma	12/32	0.046650009
Lobar Holoprosencephaly	12/32	0.046650009
Diffuse Astrocytoma	20/66	0.046704283
Reflex, Deep Tendon, Absent	35/137	0.048012331
Absent reflex	35/137	0.048012331
Pituitary Neoplasms	66/296	0.048881765
Microdontia (disorder)	23/80	0.049743284

Supplementary Table 4. KEGG Pathway analyses of genes with significantly altered transcript levels in untreated *Tet/Tet* neurons

KEGG Pathway	Overlap	Bejamini-Hochberg Adjusted P-value
Pathways in cancer	140/531	9.62E-08
Axon guidance	63/182	9.62E-08
ECM-receptor interaction	36/88	2.28E-06
Calcium signaling pathway	72/240	3.25E-06
Glutamatergic synapse	41/114	1.10E-05
Wnt signaling pathway	53/166	1.47E-05
Protein digestion and absorption	37/103	2.93E-05
Dopaminergic synapse	44/132	2.93E-05
PI3K-Akt signaling pathway	92/354	3.51E-05
DNA replication	18/36	7.23E-05
Hepatocellular carcinoma	50/168	1.67E-04
Cell adhesion molecules	45/148	2.37E-04
Melanogenesis	34/101	2.37E-04
Circadian entrainment	33/97	2.37E-04
GABAergic synapse	31/89	2.37E-04
Proteoglycans in cancer	57/205	2.63E-04
Morphine addiction	31/91	3.20E-04
Signaling pathways regulating pluripotency of stem cells	43/143	3.20E-04
MAPK signaling pathway	75/294	3.20E-04
Cholinergic synapse	36/113	3.55E-04
Focal adhesion	55/201	4.61E-04
Hypertrophic cardiomyopathy	30/90	5.83E-04
Insulin secretion	29/86	5.84E-04
Rap1 signaling pathway	56/210	7.51E-04
Dilated cardiomyopathy	31/96	7.66E-04
Basal cell carcinoma	23/63	7.78E-04
GnRH secretion	23/64	9.93E-04
Arrhythmogenic right ventricular cardiomyopathy	26/77	0.001141077
Amphetamine addiction	24/69	0.001186593
Ribosome	44/158	0.001230722
Hippo signaling pathway	45/163	0.001251669
Cushing syndrome	43/155	0.001430836
Coronavirus disease	59/232	0.001430836
Ras signaling pathway	59/232	0.001430836
Breast cancer	41/147	0.001703645
Human papillomavirus infection	78/331	0.001993509
Adrenergic signaling in cardiomyocytes	41/150	0.002572007
Ferroptosis	16/41	0.002809936
Glutathione metabolism	20/57	0.002812978
Apelin signaling pathway	38/137	0.0028269
Aldosterone synthesis and secretion	29/98	0.004197718
Pathways of neurodegeneration	103/475	0.00515825
Oxytocin signaling pathway	40/154	0.007783475
Long-term potentiation	21/67	0.009751812
Retrograde endocannabinoid signaling	38/148	0.012229604
Cell cycle	33/124	0.012362765
Relaxin signaling pathway	34/129	0.012427179
Synaptic vesicle cycle	23/78	0.013473367
One carbon pool by folate	9/20	0.013748267
Oocyte meiosis	33/129	0.021841385
AGE-RAGE signaling pathway in diabetic complications	27/100	0.021841385
Tight junction	41/169	0.021841385
Base excision repair	12/33	0.021841385
Amoebiasis	27/102	0.028163948
Gap junction	24/88	0.029115222

Inflammatory mediator regulation of TRP channels	26/98	0.030545374
Serotonergic synapse	29/113	0.03181565
Phospholipase D signaling pathway	36/148	0.031915979
cAMP signaling pathway	49/216	0.034633125
Mismatch repair	9/23	0.034633125
Gastric acid secretion	21/76	0.036794212
Glycosaminoglycan biosynthesis	16/53	0.036794212
Glycine, serine and threonine metabolism	13/40	0.038009171
cGMP-PKG signaling pathway	39/167	0.042335676
Glycosphingolipid biosynthesis	14/45	0.042335676
Cardiac muscle contraction	23/87	0.042728418
Notch signaling pathway	17/59	0.044658838

Supplementary Table 5. Gene ontology terms identified using genes showing altered transcript levels in untreated *Tet/Tet* neurons

Gene Ontology: Biological Processes	Overlap	Bejamini-Hochberg Adjusted P-value
Nervous System Development (GO:0007399)	147/433	9.85E-17
Axonogenesis (GO:0007409)	78/188	1.60E-13
Axon Guidance (GO:0007411)	63/149	3.50E-11
Cell-Cell Adhesion Via Plasma-Membrane Adhesion Molecules (GO:0098742)	69/172	3.65E-11
Chemical Synaptic Transmission (GO:0007268)	94/273	5.72E-11
Synapse Organization (GO:0050808)	56/131	2.77E-10
Axon Development (GO:0061564)	45/99	3.51E-09
Neuron Projection Guidance (GO:0097485)	52/124	3.51E-09
Regulation Of Cell Migration (GO:0030334)	122/434	5.85E-08
Anterograde Trans-Synaptic Signaling (GO:0098916)	68/199	1.22E-07
Transmembrane Receptor Protein Tyrosine Kinase Signaling Pathway (GO:0007169)	86/284	5.64E-07
Regulation Of Synapse Assembly (GO:0051963)	27/51	5.65E-07
Neuron Projection Morphogenesis (GO:0048812)	53/146	7.74E-07
Neurotransmitter Secretion (GO:0007269)	24/43	9.67E-07
Generation Of Neurons (GO:0048699)	59/172	1.03E-06
Signal Release From Synapse (GO:0099643)	22/38	1.53E-06
Transport Across Blood-Brain Barrier (GO:0150104)	36/85	2.10E-06
Modulation Of Chemical Synaptic Transmission (GO:0050804)	46/123	2.16E-06
Vascular Transport (GO:0010232)	35/83	3.47E-06
Cell Morphogenesis Involved In Neuron Differentiation (GO:0048667)	33/77	5.26E-06
Central Nervous System Development (GO:0007417)	82/283	6.52E-06
Regulation Of Neuron Projection Development (GO:0010975)	56/174	2.17E-05
Positive Regulation Of Cell Differentiation (GO:0045597)	80/283	2.78E-05
External Encapsulating Structure Organization (GO:0045229)	40/110	3.61E-05
Canonical Wnt Signaling Pathway (GO:0060070)	28/65	4.25E-05
Cell Junction Organization (GO:0034330)	27/62	5.13E-05
Regulation Of Axonogenesis (GO:0050770)	21/42	6.08E-05
Neuron Development (GO:0048666)	49/150	6.12E-05
Extracellular Matrix Organization (GO:0030198)	55/176	6.12E-05
Negative Regulation Of Cell Differentiation (GO:0045596)	62/207	6.12E-05
Positive Regulation Of Nervous System Development (GO:0051962)	23/49	6.25E-05
Extracellular Structure Organization (GO:0043062)	39/109	6.25E-05
Neuron Differentiation (GO:0030182)	54/173	7.30E-05
Homophilic Cell Adhesion Via Plasma Membrane Adhesion Molecules (GO:0007156)	26/60	7.30E-05
Synapse Assembly (GO:0007416)	29/71	7.30E-05
Regulation Of Anatomical Structure Morphogenesis (GO:0022603)	43/127	8.41E-05
Regulation Of ERK1 And ERK2 Cascade (GO:0070372)	69/244	1.19E-04
Regulation Of Endothelial Cell Migration (GO:0010594)	33/88	1.19E-04
Cellular Response To Salt (GO:1902075)	38/109	1.51E-04
Glutamate Receptor Signaling Pathway (GO:0007215)	18/35	1.67E-04
Neurotransmitter Transport (GO:0006836)	23/52	1.73E-04
Positive Regulation Of Synapse Assembly (GO:0051965)	17/32	1.73E-04
Negative Regulation Of Protein Phosphorylation (GO:0001933)	47/149	2.12E-04
Regulation Of Canonical Wnt Signaling Pathway (GO:0060828)	60/207	2.12E-04
Positive Regulation Of Macromolecule Metabolic Process (GO:0010604)	93/364	2.13E-04
Cell Junction Assembly (GO:0034329)	34/95	2.33E-04
Response To Cytokine (GO:0034097)	41/125	2.87E-04
Regulation Of Cell Migration Involved In Sprouting Angiogenesis (GO:0090049)	16/30	2.87E-04
Positive Regulation Of Supramolecular Fiber Organization (GO:1902905)	34/96	2.87E-04
Neuron Projection Development (GO:0031175)	56/192	3.28E-04
Regulation Of Monoatomic Ion Transmembrane Transporter Activity (GO:0032412)	27/69	3.28E-04
Positive Regulation Of MAPK Cascade (GO:0043410)	81/310	3.30E-04
Regulation Of Bone Mineralization (GO:0030500)	24/58	3.45E-04
Regulation Of Ossification (GO:0030278)	18/37	3.49E-04
Positive Regulation Of Nucleic Acid-Templated Transcription (GO:1903508)	130/557	3.53E-04
Negative Regulation Of Canonical Wnt Signaling Pathway (GO:0090090)	38/114	3.64E-04
Negative Regulation Of Wnt Signaling Pathway (GO:0030178)	44/140	3.82E-04

Response To Calcium Ion (GO:0051592)	36/106	3.84E-04
Cellular Response To Oxygen-Containing Compound (GO:1901701)	100/406	3.89E-04
Positive Regulation Of Angiogenesis (GO:0045766)	39/119	4.06E-04
Positive Regulation Of Ossification (GO:0045778)	19/41	4.15E-04
Regulation Of Calcium Ion-Dependent Exocytosis (GO:0017158)	15/28	4.28E-04
Positive Regulation Of DNA-templated Transcription (GO:0045893)	256/1243	6.03E-04
Wnt Signaling Pathway (GO:0016055)	28/76	6.82E-04
Cellular Response To Metal Ion (GO:0071248)	42/135	7.19E-04
Synaptic Membrane Adhesion (GO:0099560)	9/12	7.19E-04
Metal Ion Transport (GO:0030001)	50/171	7.41E-04
Positive Regulation Of Cell Projection Organization (GO:0031346)	38/118	7.46E-04
Sodium Ion Transport (GO:0006814)	31/89	8.11E-04
Regulation Of Osteoblast Differentiation (GO:0045667)	30/85	8.17E-04
Ephrin Receptor Signaling Pathway (GO:0048013)	19/43	8.30E-04
Positive Regulation Of Biomineral Tissue Development (GO:0070169)	19/43	8.30E-04
Peptidyl-Tyrosine Modification (GO:0018212)	22/54	8.50E-04
Regulation Of Angiogenesis (GO:0045765)	57/205	8.95E-04
Negative Regulation Of Signal Transduction (GO:0009968)	70/267	9.03E-04
Regulation Of Wound Healing (GO:0061041)	20/47	9.29E-04
Regulation Of Apoptotic Process (GO:0042981)	155/705	0.001092005
Cellular Response To Retinoic Acid (GO:0071300)	18/41	0.001423563
Synaptic Vesicle Exocytosis (GO:0016079)	18/41	0.001423563
Negative Regulation Of Protein Kinase Activity (GO:0006469)	30/88	0.001548697
Supramolecular Fiber Organization (GO:0097435)	79/316	0.001556437
Central Nervous System Neuron Differentiation (GO:0021953)	15/31	0.001603689
Cell-Cell Junction Organization (GO:0045216)	25/68	0.00163157
Cytoplasmic Translation (GO:0002181)	31/93	0.001799133
Collagen Fibril Organization (GO:0030199)	18/42	0.001933708
Positive Regulation Of Neuron Differentiation (GO:0045666)	18/42	0.001933708
Regulation Of Presynapse Assembly (GO:1905606)	10/16	0.001933708
Regulation Of Presynapse Organization (GO:0099174)	10/16	0.001933708
Regulation Of Chondrocyte Differentiation (GO:0032330)	13/25	0.001933708
Positive Regulation Of Programmed Cell Death (GO:0043068)	64/245	0.001933708
Positive Regulation Of Cell Motility (GO:2000147)	59/221	0.001933708
Cellular Response To Calcium Ion (GO:0071277)	25/69	0.001958834
Positive Regulation Of Transcription By RNA Polymerase II (GO:0045944)	196/938	0.002023306
Positive Regulation Of Cell Population Proliferation (GO:0008284)	111/483	0.002057626
Cellular Response To Organic Cyclic Compound (GO:0071407)	44/152	0.002254842
Protein Phosphorylation (GO:0006468)	114/500	0.002281827
Memory (GO:0007613)	22/58	0.002395252
Cellular Response To Lipid (GO:0071396)	60/228	0.002439632
Cardiac Muscle Cell Action Potential (GO:0086001)	14/29	0.002533958
Regulation Of GTPase Activity (GO:0043087)	57/214	0.002533958
Regulation Of Cell Adhesion (GO:0030155)	42/144	0.002575024
Positive Regulation Of Vasculature Development (GO:1904018)	33/104	0.002575024
Regulation Of Trans-Synaptic Signaling (GO:0099177)	16/36	0.002576475
Negative Regulation Of Macromolecule Metabolic Process (GO:0010605)	51/186	0.002626238
Regulation Of Neuron Differentiation (GO:0045664)	27/79	0.002796877
Neuron Migration (GO:0001764)	21/55	0.002863669
Regulation Of Synaptic Transmission, Glutamatergic (GO:0051966)	21/55	0.002863669
Aortic Valve Development (GO:0003176)	17/40	0.002881296
Peptidyl-Tyrosine Phosphorylation (GO:0018108)	24/67	0.002881296
Neuron Cell-Cell Adhesion (GO:0007158)	9/14	0.002881296
Positive Regulation Of Neuron Projection Development (GO:0010976)	30/92	0.002888652
Regulation Of Cell Motility (GO:2000145)	37/123	0.00305995
Circulatory System Development (GO:0072359)	39/132	0.00305995
Positive Regulation Of Apoptotic Process (GO:0043065)	68/270	0.00305995
Negative Regulation Of Protein Autophosphorylation (GO:0031953)	7/9	0.00305995
Aorta Morphogenesis (GO:0035909)	10/17	0.00305995
Regulation Of Calcium Ion Transmembrane Transport (GO:1903169)	11/20	0.00305995
Aortic Valve Morphogenesis (GO:0003180)	16/37	0.003374041

Negative Regulation Of Cell Adhesion (GO:0007162)	25/72	0.003405519
Negative Regulation Of Cell Motility (GO:2000146)	39/133	0.003471189
Response To Retinoic Acid (GO:0032526)	22/60	0.003488479
Regulation Of MAPK Cascade (GO:0043408)	54/204	0.003848905
Amino Acid Import Across Plasma Membrane (GO:0089718)	13/27	0.003907712
Regulation Of Catecholamine Secretion (GO:0050433)	13/27	0.003907712
Cellular Response To Hydrogen Peroxide (GO:0070301)	15/34	0.00393483
Glial Cell Differentiation (GO:0010001)	15/34	0.00393483
Pyrimidine Nucleobase Catabolic Process (GO:0006208)	6/7	0.00393483
Thyroid Hormone Transport (GO:0070327)	6/7	0.00393483
Negative Regulation Of Cellular Process (GO:0048523)	119/537	0.004233427
Positive Regulation Of Cell Junction Assembly (GO:1901890)	22/61	0.004314668
Regulation Of AMPA Receptor Activity (GO:2000311)	12/24	0.004336478
Positive Regulation Of Regulated Secretory Pathway (GO:1903307)	8/12	0.00433821
Calcium-Dependent Cell-Cell Adhesion Via Plasma Membrane Cell Adhesion Molecules (GO:0016339)	16/38	0.00433821
Positive Regulation Of Axonogenesis (GO:0050772)	16/38	0.00433821
Positive Regulation Of Bone Mineralization (GO:0030501)	16/38	0.00433821
Positive Regulation Of Gene Expression (GO:0010628)	108/480	0.00439296
Regulation Of Cell Population Proliferation (GO:0042127)	161/766	0.004916885
Cellular Response To BMP Stimulus (GO:0071773)	23/66	0.005232264
Negative Regulation Of Neuron Projection Development (GO:0010977)	20/54	0.005334786
Regulation Of Neuron Death (GO:1901214)	21/58	0.005334786
Positive Regulation Of Cell Migration (GO:0030335)	67/272	0.005634717
Negative Regulation Of Cell Population Proliferation (GO:0008285)	88/379	0.005656023
Positive Regulation Of Cytoskeleton Organization (GO:0051495)	26/79	0.005656023
Positive Regulation Of Neurogenesis (GO:0050769)	25/75	0.005932324
Positive Regulation Of Cell Adhesion (GO:0045785)	28/88	0.006126213
Regulation Of Hydrolase Activity (GO:0051336)	24/71	0.006140813
Learning (GO:0007612)	17/43	0.006237872
Enzyme-Linked Receptor Protein Signaling Pathway (GO:0007167)	36/124	0.006430912
Epithelial To Mesenchymal Transition (GO:0001837)	18/47	0.006467088
Positive Regulation Of Transferase Activity (GO:0051347)	40/143	0.006950171
Protein Autophosphorylation (GO:0046777)	44/162	0.007115918
Regulation Of Neural Precursor Cell Proliferation (GO:2000177)	11/22	0.007230824
Cardiac Epithelial To Mesenchymal Transition (GO:0060317)	10/19	0.008040789
Glycosphingolipid Biosynthetic Process (GO:0006688)	10/19	0.008040789
Negative Regulation Of Cell Migration (GO:0030336)	44/163	0.008040789
Negative Regulation Of ERK1 And ERK2 Cascade (GO:0070373)	18/48	0.008368792
Regulation Of Cation Channel Activity (GO:2001257)	20/56	0.008368792
Sprouting Angiogenesis (GO:0002040)	19/52	0.008368792
Positive Regulation Of Cell Migration Involved In Sprouting Angiogenesis (GO:0090050)	9/16	0.008368792
Regulation Of Intracellular Signal Transduction (GO:1902531)	71/297	0.008423598
Positive Regulation Of Synaptic Transmission (GO:0050806)	25/77	0.008442025
DNA Replication (GO:0006260)	24/73	0.008922261
Regulation Of Signal Transduction (GO:0009966)	50/193	0.008946338
Cell-Cell Adhesion Mediated By Cadherin (GO:0044331)	12/26	0.009019799
Brain Development (GO:0007420)	45/169	0.009019799
Cellular Response To Growth Factor Stimulus (GO:0071363)	42/155	0.009376809
Regulation Of Sodium Ion Transmembrane Transporter Activity (GO:2000649)	15/37	0.009393507
Regulation Of Heart Rate By Cardiac Conduction (GO:0086091)	16/41	0.009988871
Epithelial To Mesenchymal Transition Involved In Endocardial Cushion Formation (GO:0003198)	6/8	0.01028744
Positive Regulation Of Epithelial Cell Proliferation (GO:0050679)	35/123	0.010381099
Positive Regulation Of Cell Communication (GO:0010647)	13/30	0.010414404
Protein Localization To Cell Junction (GO:1902414)	11/23	0.010414404
Response To BMP (GO:0071772)	11/23	0.010414404
Regulation Of Protein Phosphorylation (GO:0001932)	64/265	0.011245494
Cellular Component Assembly (GO:0022607)	63/260	0.011245494
Regulation Of Neuronal Synaptic Plasticity (GO:0048168)	14/34	0.011601944
Positive Regulation Of Endocytosis (GO:0045807)	25/79	0.011925114
Negative Regulation Of B Cell Activation (GO:0050869)	10/20	0.011987978
Negative Regulation Of Kinase Activity (GO:0033673)	21/62	0.012007335

Import Into Cell (GO:0098657)	15/38	0.012276903
Ventricular Septum Morphogenesis (GO:0060412)	12/27	0.012582082
Regulation Of Smooth Muscle Cell Proliferation (GO:0048660)	19/54	0.012623275
Regulation Of Neurotransmitter Receptor Activity (GO:0099601)	16/42	0.012623275
Amino Acid Transport (GO:0006865)	17/46	0.012717372
Response To Hydrogen Peroxide (GO:0042542)	17/46	0.012717372
Regulation Of Interleukin-1 Beta Production (GO:0032651)	26/84	0.012762415
Negative Regulation Of Bone Remodeling (GO:0046851)	7/11	0.012762415
Pyrimidine Nucleobase Metabolic Process (GO:0006206)	7/11	0.012762415
Regulation Of Actin Filament Depolymerization (GO:0030834)	7/11	0.012762415
Extracellular Matrix Assembly (GO:0085029)	9/17	0.012762415
Serine Family Amino Acid Metabolic Process (GO:0009069)	8/14	0.013342523
Synaptonemal Complex Assembly (GO:0007130)	8/14	0.013342523
Synaptonemal Complex Organization (GO:0070193)	8/14	0.013342523
Regulation Of Extrinsic Apoptotic Signaling Pathway Via Death Domain Receptors (GO:1902041)	13/31	0.013637408
Elastic Fiber Assembly (GO:0048251)	5/6	0.013788819
Nucleobase Catabolic Process (GO:0046113)	5/6	0.013788819
Negative Regulation Of Ossification (GO:0030279)	11/24	0.014444281
Action Potential (GO:0001508)	14/35	0.014666769
Positive Regulation Of Endothelial Cell Migration (GO:0010595)	26/85	0.014706963
Regulation Of Kinase Activity (GO:0043549)	26/85	0.014706963
Regulation Of Release Of Sequestered Calcium Ion Into Cytosol (GO:0051279)	18/51	0.015374791
Positive Regulation Of Smooth Muscle Cell Proliferation (GO:0048661)	17/47	0.015543976
Regulation Of Sodium Ion Transmembrane Transport (GO:1902305)	12/28	0.016780635
Plasma Membrane Bounded Cell Projection Organization (GO:0120036)	36/132	0.017269128
Regulation Of Small GTPase Mediated Signal Transduction (GO:0051056)	33/118	0.017450371
Positive Regulation Of Cysteine-Type Endopeptidase Activity Involved In Apoptotic Process (GO:0043280)	31/109	0.018203355
Positive Regulation Of Secretion By Cell (GO:1903532)	23/73	0.018203355
Muscle Cell Development (GO:0055001)	13/32	0.018296315
Sodium Ion Transmembrane Transport (GO:0035725)	25/82	0.018694775
Positive Regulation Of Phosphorylation (GO:0042327)	56/231	0.018694775
Positive Regulation Of MAP Kinase Activity (GO:0043406)	27/91	0.018694775
DNA-templated DNA Replication (GO:0006261)	22/69	0.019166322
Cardiac Muscle Cell Contraction (GO:0086003)	9/18	0.019437965
Regulation Of Neuron Apoptotic Process (GO:0043523)	30/105	0.019437965
L-alpha-amino Acid Transmembrane Transport (GO:1902475)	15/40	0.019437965
Positive Regulation Of Osteoblast Differentiation (GO:0045669)	16/44	0.019445829
Negative Regulation Of Protein Modification Process (GO:0031400)	24/78	0.019689336
Regulation Of Biomineral Tissue Development (GO:0070167)	11/25	0.019838988
Regulation Of Glycogen Biosynthetic Process (GO:0005979)	11/25	0.019838988
Positive Regulation Of Protein Phosphorylation (GO:0001934)	84/377	0.019838988
Regulation Of JNK Cascade (GO:0046328)	31/110	0.019838988
B Cell Homeostasis (GO:0001782)	6/9	0.019838988
Hormone Transport (GO:0009914)	6/9	0.019838988
Negative Regulation Of Bone Resorption (GO:0045779)	6/9	0.019838988
Postsynapse Assembly (GO:0099068)	6/9	0.019838988
Serine Family Amino Acid Catabolic Process (GO:0009071)	6/9	0.019838988
Smooth Muscle Tissue Development (GO:0048745)	6/9	0.019838988
Peptide Biosynthetic Process (GO:0043043)	41/158	0.020552638
Calcium Ion Transmembrane Import Into Cytosol (GO:0097553)	25/83	0.020793565
Positive Regulation Of Neural Precursor Cell Proliferation (GO:2000179)	12/29	0.021443127
Macromolecule Biosynthetic Process (GO:0009059)	46/183	0.021443127
Axonal Fasciculation (GO:0007413)	7/12	0.021443127
Ionotropic Glutamate Receptor Signaling Pathway (GO:0035235)	7/12	0.021443127
Ras Protein Signal Transduction (GO:0007265)	38/144	0.021443127
Positive Regulation Of Protein Polymerization (GO:0032273)	22/70	0.021443127
Regulation Of Mitotic Cell Cycle (GO:0007346)	34/125	0.021714302
Negative Regulation Of Blood Vessel Endothelial Cell Migration (GO:0043537)	13/33	0.022633624
Acetylcholine Receptor Signaling Pathway (GO:0095500)	10/22	0.022934983
Positive Regulation Of Excitatory Postsynaptic Potential (GO:2000463)	10/22	0.022934983
Positive Regulation Of Signaling (GO:0023056)	14/37	0.022947224

Ventricular Septum Development (GO:0003281)	14/37	0.022947224
Positive Regulation Of Intracellular Signal Transduction (GO:1902533)	111/525	0.022947224
Positive Regulation Of ERK1 And ERK2 Cascade (GO:0070374)	45/179	0.022947224
Non-Canonical Wnt Signaling Pathway (GO:0035567)	15/41	0.022947224
Regulation Of Dendrite Development (GO:0050773)	15/41	0.022947224
Protein-Containing Complex Assembly (GO:0065003)	74/328	0.024273723
Negative Regulation Of Nucleic Acid-Templated Transcription (GO:1903507)	98/456	0.024784633
L-amino Acid Transport (GO:0015807)	11/26	0.026012143
Activation Of Cysteine-Type Endopeptidase Activity Involved In Apoptotic Process (GO:0006919)	21/67	0.027082218
Cardiac Ventricle Development (GO:0003231)	9/19	0.027133214
Homologous Chromosome Pairing At Meiosis (GO:0007129)	12/30	0.028098624
Regulation Of Potassium Ion Transport (GO:0043266)	12/30	0.028098624
Cardiac Conduction (GO:0061337)	16/46	0.028718696
Negative Regulation Of Interleukin-6 Production (GO:0032715)	13/34	0.029101145
Semaphorin-Plexin Signaling Pathway (GO:0071526)	13/34	0.029101145
Cardiac Ventricle Morphogenesis (GO:0003208)	15/42	0.029148755
G2/M Transition Of Mitotic Cell Cycle (GO:0000086)	14/38	0.029297602
Negative Regulation Of Gene Expression (GO:0010629)	75/336	0.029306526
Negative Regulation Of Developmental Process (GO:0051093)	30/109	0.030483359
Positive Regulation Of Wnt Signaling Pathway (GO:0030177)	30/109	0.030483359
Cell Surface Receptor Signaling Pathway Involved In Heart Development (GO:0061311)	8/16	0.030483359
Membrane Depolarization During Cardiac Muscle Cell Action Potential (GO:0086012)	8/16	0.030483359
Positive Regulation Of Cardiac Muscle Tissue Growth (GO:0055023)	8/16	0.030483359
Aromatic Amino Acid Transport (GO:0015801)	5/7	0.030483359
Lens Fiber Cell Development (GO:0070307)	5/7	0.030483359
Negative Regulation Of cAMP-dependent Protein Kinase Activity (GO:2000480)	5/7	0.030483359
Regulation Of Leukocyte Degranulation (GO:0043300)	5/7	0.030483359
Regulation Of Mesenchymal Stem Cell Differentiation (GO:2000739)	5/7	0.030483359
Regulation Of Myeloid Leukocyte Differentiation (GO:0002761)	5/7	0.030483359
Axon Extension (GO:0048675)	10/23	0.030671818
Regulation Of Extracellular Matrix Organization (GO:1903053)	10/23	0.030671818
Regulated Exocytosis (GO:0045055)	17/51	0.032734524
Cellular Response To Organonitrogen Compound (GO:0071417)	29/105	0.032734524
Negative Regulation Of Cytokine Production (GO:0001818)	44/178	0.032734524
Response To Metal Ion (GO:0010038)	20/64	0.032734524
Cellular Response To Organic Substance (GO:0071310)	24/82	0.032734524
Cellular Response To Decreased Oxygen Levels (GO:0036294)	22/73	0.032734524
Microtubule Polymerization Or Depolymerization (GO:0031109)	11/27	0.032734524
Synaptic Transmission, Glutamatergic (GO:0035249)	11/27	0.032734524
Negative Regulation Of Cartilage Development (GO:0061037)	7/13	0.032734524
Negative Regulation Of Cell Migration Involved In Sprouting Angiogenesis (GO:0090051)	7/13	0.032734524
Negative Regulation Of Myoblast Differentiation (GO:0045662)	7/13	0.032734524
Protein Localization To Synapse (GO:0035418)	7/13	0.032734524
Regulation Of Short-Term Neuronal Synaptic Plasticity (GO:0048172)	7/13	0.032734524
Secondary Palate Development (GO:0062009)	7/13	0.032734524
Monoatomic Cation Transmembrane Transport (GO:0098655)	64/281	0.033083188
Notch Signaling Involved In Heart Development (GO:0061314)	6/10	0.033083188
Basement Membrane Organization (GO:0071711)	6/10	0.033083188
Hematopoietic Stem Cell Proliferation (GO:0071425)	6/10	0.033083188
Mitotic DNA Replication (GO:1902969)	6/10	0.033083188
Neuron Projection Fasciculation (GO:0106030)	6/10	0.033083188
Positive Regulation Of Chondrocyte Differentiation (GO:0032332)	6/10	0.033083188
Positive Regulation Of Non-Canonical Wnt Signaling Pathway (GO:2000052)	6/10	0.033083188
Positive Regulation Of Protein Depolymerization (GO:1901881)	6/10	0.033083188
Modulation Of Excitatory Postsynaptic Potential (GO:0098815)	12/31	0.033498435
Odontogenesis (GO:0042476)	14/39	0.033745154
Adherens Junction Organization (GO:0034332)	13/35	0.033745154
Neutral Amino Acid Transport (GO:0015804)	13/35	0.033745154
Regulation Of Regulated Secretory Pathway (GO:1903305)	13/35	0.033745154
Regulation Of Heart Contraction (GO:0008016)	21/69	0.033855617
DNA Unwinding Involved In DNA Replication (GO:0006268)	9/20	0.034941939

Pulmonary Valve Development (GO:0003177)	9/20	0.034941939
Regulation Of Membrane Depolarization (GO:0003254)	9/20	0.034941939
Mitotic Cell Cycle Phase Transition (GO:0044772)	30/111	0.035787898
Positive Regulation Of Reactive Oxygen Species Metabolic Process (GO:2000379)	16/48	0.039416246
Regulation Of Pathway-Restricted SMAD Protein Phosphorylation (GO:0060393)	19/61	0.039556148
Regulation Of Actin Filament Polymerization (GO:0030833)	21/70	0.040097161
Sulfur Compound Biosynthetic Process (GO:0044272)	21/70	0.040097161
Peptidyl-Serine Modification (GO:0018209)	41/166	0.041402344
Negative Regulation Of Mitotic Metaphase/Anaphase Transition (GO:0045841)	11/28	0.04165252
Regulation Of Bone Resorption (GO:0045124)	11/28	0.04165252
Regulation Of Cardiac Muscle Cell Action Potential (GO:0098901)	11/28	0.04165252
Notch Signaling Pathway (GO:0007219)	14/40	0.04165252
Cell Cycle G2/M Phase Transition (GO:0044839)	14/40	0.04165252
Synaptic Vesicle Endocytosis (GO:0048488)	14/40	0.04165252
G Protein-Coupled Acetylcholine Receptor Signaling Pathway (GO:0007213)	8/17	0.04165252
Positive Regulation Of Cartilage Development (GO:0061036)	8/17	0.04165252
Postsynaptic Membrane Organization (GO:0001941)	8/17	0.04165252
Negative Regulation Of Phosphorylation (GO:0042326)	22/75	0.042274661
Negative Regulation Of Fat Cell Differentiation (GO:0045599)	12/32	0.042274661
Regulation Of Tumor Necrosis Factor Production (GO:0032680)	33/127	0.042330584
L-aspartate Import Across Plasma Membrane (GO:0140009)	4/5	0.042998288
L-aspartate Transmembrane Transport (GO:0070778)	4/5	0.042998288
T-helper 1 Cell Differentiation (GO:0045063)	4/5	0.042998288
Cellular Response To Iron Ion (GO:0071281)	4/5	0.042998288
Endothelin Receptor Signaling Pathway (GO:0086100)	4/5	0.042998288
Hexose Import Across Plasma Membrane (GO:0140271)	4/5	0.042998288
Presynaptic Active Zone Organization (GO:1990709)	4/5	0.042998288
Glycoprotein Metabolic Process (GO:0009100)	17/53	0.042998288
Regulation Of Alternative mRNA Splicing, Via Spliceosome (GO:0000381)	17/53	0.042998288
Negative Regulation Of Protein Serine/Threonine Kinase Activity (GO:0071901)	23/80	0.04399323
Muscle Contraction (GO:0006936)	26/94	0.044130938
Negative Regulation Of Cellular Response To Growth Factor Stimulus (GO:0090288)	21/71	0.044830888
Positive Regulation Of Pathway-Restricted SMAD Protein Phosphorylation (GO:0010862)	16/49	0.045599666
Integrin-Mediated Signaling Pathway (GO:0007229)	24/85	0.046324448
Regulation Of Cell Differentiation (GO:0045595)	46/193	0.046324448
Positive Regulation Of Kinase Activity (GO:0033674)	28/104	0.046754645
Aorta Development (GO:0035904)	7/14	0.046754645
Contractile Actin Filament Bundle Assembly (GO:0030038)	7/14	0.046754645
Mammary Gland Epithelium Development (GO:0061180)	7/14	0.046754645
Negative Regulation Of Androgen Receptor Signaling Pathway (GO:0060766)	7/14	0.046754645
Odontogenesis Of Dentin-Containing Tooth (GO:0042475)	7/14	0.046754645
Regulation Of Mast Cell Degranulation (GO:0043304)	7/14	0.046754645
Stress Fiber Assembly (GO:0043149)	7/14	0.046754645
Regulation Of MAP Kinase Activity (GO:0043405)	30/114	0.04860414
Regulation Of Interleukin-6 Production (GO:0032675)	30/114	0.04860414
Positive Regulation Of Epithelial Cell Migration (GO:0010634)	26/95	0.049372196
Regulation Of Protein Kinase Activity (GO:0045859)	31/119	0.049372196
Mismatch Repair (GO:0006298)	10/25	0.049948926
Endocytosis (GO:0006897)	45/189	0.049948926

Gene Ontology: Cellular Component	Overlap	Adjusted P-value
Neuron Projection (GO:0043005)	179/557	5.19E-19
Collagen-Containing Extracellular Matrix (GO:0062023)	125/373	9.73E-15
Axon (GO:0030424)	76/205	2.80E-11
Postsynaptic Density (GO:0014069)	58/151	2.42E-09
Dendrite (GO:0030425)	87/270	2.42E-09
Actin Cytoskeleton (GO:0015629)	92/327	1.35E-06
Asymmetric Synapse (GO:0032279)	47/133	2.53E-06
Glutamatergic Synapse (GO:0098978)	32/78	6.20E-06
Cell-Cell Junction (GO:0005911)	82/299	1.69E-05
Cytoskeleton (GO:0005856)	142/599	2.33E-05

Cell-Substrate Junction (GO:0030055)	100/395	4.78E-05
Focal Adhesion (GO:0005925)	97/387	1.00E-04
GABA-ergic Synapse (GO:0098982)	12/19	1.50E-04
Adherens Junction (GO:0005912)	46/150	1.67E-04
Postsynaptic Specialization Membrane (GO:0099634)	18/38	1.70E-04
Cytosolic Large Ribosomal Subunit (GO:0022625)	21/52	5.61E-04
Large Ribosomal Subunit (GO:0015934)	21/52	5.61E-04
Specific Granule Membrane (GO:0035579)	30/90	9.37E-04
Ionotropic Glutamate Receptor Complex (GO:0008328)	15/33	0.001451474
Potassium Channel Complex (GO:0034705)	27/81	0.001911388
Postsynaptic Density Membrane (GO:0098839)	15/34	0.001911388
Extrinsic Component Of Cytoplasmic Side Of Plasma Membrane (GO:0031234)	26/77	0.001911388
Secretory Granule Membrane (GO:0030667)	69/279	0.001965415
Endoplasmic Reticulum Lumen (GO:0005788)	70/284	0.001965415
Catenin Complex (GO:0016342)	13/28	0.002585609
Cytoplasmic Side Of Plasma Membrane (GO:0009898)	36/124	0.003065588
Polymeric Cytoskeletal Fiber (GO:0099513)	65/265	0.003473802
Glial Cell Projection (GO:0097386)	8/13	0.003842418
Endocytic Vesicle Membrane (GO:0030666)	43/159	0.003888236
Specific Granule (GO:0042581)	43/159	0.003888236
Voltage-Gated Potassium Channel Complex (GO:0008076)	24/73	0.003904641
Basolateral Plasma Membrane (GO:0016323)	45/171	0.005058421
Vesicle (GO:0031982)	58/235	0.005058421
Basement Membrane (GO:0005604)	17/46	0.005794862
Cell-Cell Contact Zone (GO:0044291)	17/47	0.00751274
Plasma Membrane Raft (GO:0044853)	25/82	0.009109713
Sarcolemma (GO:0042383)	18/52	0.009109713
Membrane Raft (GO:0045121)	43/169	0.012105998
Clathrin-Sculpted Gamma-Aminobutyric Acid Transport Vesicle (GO:0061200)	5/7	0.017167537
Clathrin-Sculpted Gamma-Aminobutyric Acid Transport Vesicle Membrane (GO:0061202)	5/7	0.017167537
Extracellular Vesicle (GO:1903561)	19/60	0.019703806
Intercalated Disc (GO:0014704)	12/31	0.019703806
Extracellular Membrane-Bounded Organelle (GO:0065010)	18/57	0.025586868
Platelet Alpha Granule (GO:0031091)	25/89	0.026485016
Azurophil Granule Membrane (GO:0035577)	18/58	0.030218171
Cytoplasmic Vesicle Membrane (GO:0030659)	83/389	0.030472403
Cytosolic Small Ribosomal Subunit (GO:0022627)	14/41	0.030472403
Inhibitory Synapse (GO:0060077)	5/8	0.032419384
Perineuronal Net (GO:0072534)	5/8	0.032419384
Sodium Channel Complex (GO:0034706)	8/18	0.034357852
Small Ribosomal Subunit (GO:0015935)	14/42	0.03599713
Secretory Vesicle (GO:0099503)	7/15	0.041232414
Tight Junction (GO:0070160)	25/93	0.041232414

Gene Ontology: Molecular Function	Overlap	Adjusted P-value
Metal Ion Binding (GO:0046872)	145/521	4.17E-09
Calcium Ion Binding (GO:0005509)	101/346	2.27E-07
Transmembrane Receptor Protein Tyrosine Kinase Activity (GO:0004714)	23/50	2.07E-04
Protein Tyrosine Kinase Activity (GO:0004713)	34/99	0.001544971
Ionotropic Glutamate Receptor Activity (GO:0004970)	10/16	0.006938192
Sialyltransferase Activity (GO:0008373)	11/20	0.011079408
Amino Acid Transmembrane Transporter Activity (GO:0015171)	21/56	0.011079408
alpha-N-acetylneuraminate Alpha-2,8-Sialyltransferase Activity (GO:0003828)	5/5	0.011079408
Dihydropyrimidinase Activity (GO:0004157)	5/5	0.011079408
Cadherin Binding (GO:0045296)	77/319	0.011079408
Protein Homodimerization Activity (GO:0042803)	142/662	0.011515805
GTPase Regulator Activity (GO:0030695)	97/424	0.011515805
Pentosyltransferase Activity (GO:0016763)	16/39	0.012253123
Sequence-Specific Double-Stranded DNA Binding (GO:1990837)	151/715	0.012253123
Transcription Regulatory Region Nucleic Acid Binding (GO:0001067)	57/224	0.012910211
Glutamate Receptor Activity (GO:0008066)	9/16	0.016745636

NAD Binding (GO:0051287)	12/26	0.01753627
Sodium Channel Activity (GO:0005272)	15/37	0.017548236
High Voltage-Gated Calcium Channel Activity (GO:0008331)	11/23	0.019093637
Sodium Channel Regulator Activity (GO:0017080)	14/34	0.019578791
Transmitter-Gated Monoatomic Ion Channel Activity Involved In Regulation Of Postsynaptic Membrane Potenti	14/34	0.019578791
L-amino Acid Transmembrane Transporter Activity (GO:0015179)	21/63	0.025093401
Protein Kinase Binding (GO:0019901)	110/511	0.025289207
Magnesium Ion Binding (GO:0000287)	41/155	0.025289207
Voltage-Gated Calcium Channel Activity (GO:0005245)	17/47	0.025289207
Protein Serine/Threonine Kinase Inhibitor Activity (GO:0030291)	13/32	0.029472389
Aromatic Amino Acid Transmembrane Transporter Activity (GO:0015173)	6/9	0.031446656
Phosphatase Activity (GO:0016791)	25/83	0.031446656
Actin Binding (GO:0003779)	46/183	0.031446656
NAD+ Binding (GO:0070403)	7/12	0.031446656
Hydrolase Activity, Acting On Carbon-Nitrogen (But Not Peptide) Bonds, In Cyclic Amides (GO:0016812)	7/12	0.031446656
Protein Kinase A Catalytic Subunit Binding (GO:0034236)	7/12	0.031446656
Amyloid-Beta Binding (GO:0001540)	24/79	0.031825857
Tubulin Binding (GO:0015631)	73/322	0.033063552
NAD+ ADP-ribosyltransferase Activity (GO:0003950)	11/26	0.037112611
Voltage-Gated Sodium Channel Activity (GO:0005248)	9/19	0.037940275
Syntaxin-1 Binding (GO:0017075)	8/16	0.044044019
Ligand-Gated Monoatomic Ion Channel Activity (GO:0015276)	10/23	0.044044019
cAMP-dependent Protein Kinase Regulator Activity (GO:0008603)	6/10	0.047376064
Inositol 1,4,5 Trisphosphate Binding (GO:0070679)	6/10	0.047376064
Secondary Active Transmembrane Transporter Activity (GO:0015291)	19/60	0.047376064
Kinesin Binding (GO:0019894)	12/31	0.047376064

Supplementary table 6: Comparison of genes showing common patterns of dysregulation in ASD, EPD, SZ and BPD with those in untreated and Doxycycline treated *Tet/Tet* neurons (Red: Down regulation, Green: Up regulation, Black: No change)

**SZ+ ASD+ BPD+ EPD**	**Untreated *Tet/Tet* Neurons**	**Doxycycline treated *Tet/Tet* Neurons**
Abcc12	Abcc12	Abcc12
Abcc9	Abcc9	Abcc9
Abcd4	Abcd4	Abcd4
Abhd3	Abhd3	Abhd3
Ablim2	Ablim2	Ablim2
Acat2	Acat2	Acat2
Aco1	Aco1	Aco1
Acot7	Acot7	Acot7
Adam11	Adam11	Adam11
Adamts9	Adamts9	Adamts9
Aff4	Aff4	Aff4
Ahcyl1	Ahcyl1	Ahcyl1
Aldh2	Aldh2	Aldh2
Arap2	Arap2	Arap2
Arhgap20	Arhgap20	Arhgap20
Arhgap6	Arhgap6	Arhgap6
Arhgef2	Arhgef2	Arhgef2
Arhgef28	Arhgef28	Arhgef28
Arrdc3	Arrdc3	Arrdc3
Asap2	Asap2	Asap2
Asap3	Asap3	Asap3
Atp2b4	Atp2b4	Atp2b4
Atp6v1c1	Atp6v1c1	Atp6v1c1
B3galt2	B3galt2	B3galt2
Bcap29	Bcap29	Bcap29
Bcl11a	Bcl11a	Bcl11a
Blvrb	Blvrb	Blvrb
Btg3	Btg3	Btg3
C2cd2	C2cd2	C2cd2
Cbfa2t2	Cbfa2t2	Cbfa2t2
Cbln4	Cbln4	Cbln4
Cd83	Cd83	Cd83
Cdc14a	Cdc14a	Cdc14a
Cdk2	Cdk2	Cdk2
Cdkn1a	Cdkn1a	Cdkn1a
Chchd4	Chchd4	Chchd4

Chp1	Chp1	Chp1
Chpt1	Chpt1	Chpt1
Chrdl1	Chrdl1	Chrdl1
Chrnb2	Chrnb2	Chrnb2
Clcn3	Clcn3	Clcn3
Clk4	Clk4	Clk4
Clu	Clu	Clu
Cngb1	Cngb1	Cngb1
Cnn3	Cnn3	Cnn3
Cog6	Cog6	Cog6
Col4a1	Col4a1	Col4a1
Col4a2	Col4a2	Col4a2
Cpne3	Cpne3	Cpne3
Cpne6	Cpne6	Cpne6
Crispld2	Crispld2	Crispld2
Cyp26b1	Cyp26b1	Cyp26b1
Dach2	Dach2	Dach2
Dhx58	Dhx58	Dhx58
Dnajc28	Dnajc28	Dnajc28
Dpy19l3	Dpy19l3	Dpy19l3
Drp2	Drp2	Drp2
Dsc2	Dsc2	Dsc2
Ecm1	Ecm1	Ecm1
Edn1	Edn1	Edn1
Efemp1	Efemp1	Efemp1
Elmod1	Elmod1	Elmod1
Eml3	Eml3	Eml3
Epc2	Epc2	Epc2
Epha8	Epha8	Epha8
Erbb2	Erbb2	Erbb2
Erlin2	Erlin2	Erlin2
Ezr	Ezr	Ezr
F3	F3	F3
Fah	Fah	Fah
Fam110c	Fam110c	Fam110c
Fam189a2	Fam189a2	Fam189a2
Fam192a	Fam192a	Fam192a
Fam98c	Fam98c	Fam98c
Fbln7	Fbln7	Fbln7
Fbxo9	Fbxo9	Fbxo9
Fermt2	Fermt2	Fermt2
Fgd4	Fgd4	Fgd4
Fhl1	Fhl1	Fhl1

Fhl2	Fhl2	Fhl2
Fkbp14	Fkbp14	Fkbp14
Fzd5	Fzd5	Fzd5
G3bp1	G3bp1	G3bp1
Gabrb1	Gabrb1	Gabrb1
Gabre	Gabre	Gabre
Gadd45g	Gadd45g	Gadd45g
Gas7	Gas7	Gas7
Gdpd1	Gdpd1	Gdpd1
Gfpt2	Gfpt2	Gfpt2
Ghr	Ghr	Ghr
Glis3	Glis3	Glis3
Glud1	Glud1	Glud1
Gna13	Gna13	Gna13
Gng12	Gng12	Gng12
Gng7	Gng7	Gng7
Gpr85	Gpr85	Gpr85
Grm1	Grm1	Grm1
Gstp1	Gstp1	Gstp1
Gtdc1	Gtdc1	Gtdc1
Hapln1	Hapln1	Hapln1
Hcrtr1	Hcrtr1	Hcrtr1
Hgf	Hgf	Hgf
Hibch	Hibch	Hibch
Hilpda	Hilpda	Hilpda
Hmgcs1	Hmgcs1	Hmgcs1
Hr	Hr	Hr
Hs2st1	Hs2st1	Hs2st1
Hs3st1	Hs3st1	Hs3st1
Hs3st5	Hs3st5	Hs3st5
Hsd17b14	Hsd17b14	Hsd17b14
Hsdl2	Hsdl2	Hsdl2
Hspb8	Hspb8	Hspb8
Ifitm3	Ifitm3	Ifitm3
Igdcc4	Igdcc4	Igdcc4
Igfbp6	Igfbp6	Igfbp6
Igfbp7	Igfbp7	Igfbp7
Il17rb	Il17rb	Il17rb
Inf2	Inf2	Inf2
Inip	Inip	Inip
Irak2	Irak2	Irak2
Isca1	Isca1	Isca1
Itgb4	Itgb4	Itgb4

Itm2a	Itm2a	Itm2a
Itpkb	Itpkb	Itpkb
Itpr2	Itpr2	Itpr2
Jun	Jun	Jun
Kcnc3	Kcnc3	Kcnc3
Kcne4	Kcne4	Kcne4
Kcnip3	Kcnip3	Kcnip3
Kcnk13	Kcnk13	Kcnk13
Kcnn3	Kcnn3	Kcnn3
Kcns2	Kcns2	Kcns2
Kcns3	Kcns3	Kcns3
Klf5	Klf5	Klf5
Lats2	Lats2	Lats2
Lgals3	Lgals3	Lgals3
Lgi2	Lgi2	Lgi2
Limk2	Limk2	Limk2
Lmna	Lmna	Lmna
Lonrf3	Lonrf3	Lonrf3
Lpin1	Lpin1	Lpin1
Lpp	Lpp	Lpp
Lrat	Lrat	Lrat
Lrp10	Lrp10	Lrp10
Lrrc4	Lrrc4	Lrrc4
Lrtm2	Lrtm2	Lrtm2
Mad2l2	Mad2l2	Mad2l2
Mamld1	Mamld1	Mamld1
Maoa	Maoa	Maoa
Mapk9	Mapk9	Mapk9
Masp1	Masp1	Masp1
Mbnl1	Mbnl1	Mbnl1
Mc4r	Mc4r	Mc4r
Mcl1	Mcl1	Mcl1
Mdk	Mdk	Mdk
Mgst1	Mgst1	Mgst1
Mid1	Mid1	Mid1
Miip	Miip	Miip
Mink1	Mink1	Mink1
Mknk1	Mknk1	Mknk1
Mob3b	Mob3b	Mob3b
Mr1	Mr1	Mr1
Mtmr11	Mtmr11	Mtmr11
Nabp1	Nabp1	Nabp1
Napa	Napa	Napa

Ndrg4	Ndrg4	Ndrg4
Necap2	Necap2	Necap2
Neto2	Neto2	Neto2
Neurod6	Neurod6	Neurod6
Nid1	Nid1	Nid1
Notch2	Notch2	Notch2
Nptx2	Nptx2	Nptx2
Nrep	Nrep	Nrep
Nxnl2	Nxnl2	Nxnl2
Opa3	Opa3	Opa3
Osbpl11	Osbpl11	Osbpl11
Osbpl6	Osbpl6	Osbpl6
Osmr	Osmr	Osmr
Pafah1b3	Pafah1b3	Pafah1b3
Pamr1	Pamr1	Pamr1
Pard3	Pard3	Pard3
Pde4dip	Pde4dip	Pde4dip
Pdk4	Pdk4	Pdk4
Pdlim4	Pdlim4	Pdlim4
Pdlim5	Pdlim5	Pdlim5
Peg10	Peg10	Peg10
Phf21b	Phf21b	Phf21b
Pias2	Pias2	Pias2
Piga	Piga	Piga
Pip5k1a	Pip5k1a	Pip5k1a
Pitpna	Pitpna	Pitpna
Plcb1	Plcb1	Plcb1
Plscr4	Plscr4	Plscr4
Pltp	Pltp	Pltp
Prkab2	Prkab2	Prkab2
Prss35	Prss35	Prss35
Pxn	Pxn	Pxn
Rab11fip1	Rab11fip1	Rab11fip1
Rab15	Rab15	Rab15
Rab34	Rab34	Rab34
Rae1	Rae1	Rae1
Rfx4	Rfx4	Rfx4
Rhobtb1	Rhobtb1	Rhobtb1
Rhoc	Rhoc	Rhoc
Rhpn2	Rhpn2	Rhpn2
Rimkla	Rimkla	Rimkla
Riok1	Riok1	Riok1
Ripk1	Ripk1	Ripk1

Rnf122	Rnf122	Rnf122
Rock2	Rock2	Rock2
Rwdd1	Rwdd1	Rwdd1
Ryr3	Ryr3	Ryr3
Sbf2	Sbf2	Sbf2
Scrn3	Scrn3	Scrn3
Sdc2	Sdc2	Sdc2
Sdc4	Sdc4	Sdc4
Sertad4	Sertad4	Sertad4
Slc12a2	Slc12a2	Slc12a2
Slc16a10	Slc16a10	Slc16a10
Slc16a2	Slc16a2	Slc16a2
Slc16a9	Slc16a9	Slc16a9
Slc1a3	Slc1a3	Slc1a3
Slc1a4	Slc1a4	Slc1a4
Slc25a28	Slc25a28	Slc25a28
Slc26a11	Slc26a11	Slc26a11
Slc35f2	Slc35f2	Slc35f2
Slc35f5	Slc35f5	Slc35f5
Slc36a1	Slc36a1	Slc36a1
Slc44a3	Slc44a3	Slc44a3
Slc4a11	Slc4a11	Slc4a11
Slc7a2	Slc7a2	Slc7a2
Slc8b1	Slc8b1	Slc8b1
Slco2a1	Slco2a1	Slco2a1
Smad1	Smad1	Smad1
Smarcd3	Smarcd3	Smarcd3
Smg1	Smg1	Smg1
Smg6	Smg6	Smg6
Smyd2	Smyd2	Smyd2
Sox2	Sox2	Sox2
Sox9	Sox9	Sox9
Srcin1	Srcin1	Srcin1
Stat3	Stat3	Stat3
Stom	Stom	Stom
Ston2	Ston2	Ston2
Strn	Strn	Strn
Sv2c	Sv2c	Sv2c
Tatdn2	Tatdn2	Tatdn2
Tead1	Tead1	Tead1
Tgfb2	Tgfb2	Tgfb2
Tgfbr3	Tgfbr3	Tgfbr3
Thra	Thra	Thra

Timp1	Timp1	Timp1
Tiparp	Tiparp	Tiparp
Tmbim6	Tmbim6	Tmbim6
Tmed10	Tmed10	Tmed10
Tmem169	Tmem169	Tmem169
Tmem176a	Tmem176a	Tmem176a
Tmem176b	Tmem176b	Tmem176b
Tmem255a	Tmem255a	Tmem255a
Tmem38a	Tmem38a	Tmem38a
Tnfrsf10b	Tnfrsf10b	Tnfrsf10b
Tnfrsf1a	Tnfrsf1a	Tnfrsf1a
Tob1	Tob1	Tob1
Traf3ip2	Traf3ip2	Traf3ip2
Trib2	Trib2	Trib2
Trpc3	Trpc3	Trpc3
Trpc4	Trpc4	Trpc4
Tspan6	Tspan6	Tspan6
Ttc22	Ttc22	Ttc22
Ttl	Ttl	Ttl
Ube2l6	Ube2l6	Ube2l6
Ung	Ung	Ung
Upp2	Upp2	Upp2
Usp3	Usp3	Usp3
Wdr1	Wdr1	Wdr1
Wfs1	Wfs1	Wfs1
Wnt7a	Wnt7a	Wnt7a
Yap1	Yap1	Yap1
Zc3hav1	Zc3hav1	Zc3hav1
Zhx2	Zhx2	Zhx2
Zscan18	Zscan18	Zscan18

Supplementary Table 7. List of genes with significantly altered transcript levels in doxycycline-treated *Tet/Tet* neurons

Gene	*p*- value	Log_2 Ratio	Q-value (Benjamini-Hochberg)
A2m	0.0000	1.6475	0.0177
A4galt	0.0000	3.1343	0.0056
Aaas	0.0000	1.1041	0.0171
Aak1	0.0000	-1.2296	0.0081
Aard	0.0000	3.9240	0.0139
Aars	0.0000	1.5920	0.0017
Aass	0.0000	4.1261	0.0282
Aatk	0.0000	-2.1719	0.0141
Abcb9	0.0000	-1.6898	0.0343
Abcc4	0.0000	3.3658	0.0009
Abcc8	0.0000	-2.5375	0.0449
Abcc9	0.0000	4.4195	0.0233
Abcd2	0.0000	-1.7776	0.0177
Abcg1	0.0000	1.0789	0.0293
Abcg4	0.0000	-1.3275	0.0416
Abhd14a	0.0000	1.0984	0.0295
Abhd18	0.0000	-1.4786	0.0213
Abi3	0.0000	4.1545	0.0407
Abi3bp	0.0000	1.7212	0.0413
Ablim2	0.0000	1.3529	0.0371
Ablim3	0.0000	-1.1415	0.0352
Acacb	0.0000	2.1693	0.0115
Acad10	0.0000	1.6500	0.0202
Acad11	0.0000	1.0373	0.0240
Acadl	0.0000	1.1236	0.0151
Acap1	0.0000	5.3658	0.0213
Acap3	0.0000	-1.7974	0.0057
Acat2	0.0000	-1.5093	0.0031
Acbd4	0.0000	1.9324	0.0153
Accs	0.0000	-1.5345	0.0326
Accsl	0.0000	5.4716	0.0224
Ace	0.0000	1.3856	0.0360
Ache	0.0000	-2.3388	0.0083
Ackr3	0.0000	-1.6276	0.0161
Acot13	0.0000	1.3616	0.0151
Acot2	0.0000	1.0422	0.0286
Acot9	0.0000	1.2366	0.0176
Acoxl	0.0000	4.2242	0.0367
Acp5	0.0000	3.5973	0.0182
Acp6	0.0000	2.0126	0.0036
Acpp	0.0000	1.8332	0.0458
Acsl1	0.0000	1.4184	0.0112
Acsl4	0.0000	-1.1203	0.0136
Acsl6	0.0000	-1.4575	0.0143
Acss1	0.0000	1.3548	0.0148
Acss3	0.0000	3.5808	0.0040
Actc1	0.0000	1.2982	0.0418
Actl6b	0.0000	-3.1595	0.0037
Actn1	0.0000	1.2962	0.0053

Actr1a	0.0000	-1.1687	0.0063
Acvr2a	0.0000	-1.8096	0.0041
Acvr2b	0.0000	-1.2675	0.0135
Acy3	0.0000	1.7537	0.0430
Adad2	0.0000	4.7267	0.0380
Adam11	0.0000	-2.1638	0.0093
Adam23	0.0000	1.3629	0.0059
Adamts15	0.0000	-1.1464	0.0237
Adamts16	0.0000	-2.9053	0.0106
Adamts18	0.0000	-2.8561	0.0279
Adamts19	0.0001	-1.8184	0.0483
Adamts4	0.0000	2.5640	0.0112
Adamts6	0.0000	-1.6194	0.0302
Adamts7	0.0000	-1.7961	0.0096
Adamtsl4	0.0000	2.0251	0.0454
Adap2	0.0000	3.4725	0.0423
Adarb2	0.0000	-2.5150	0.0152
Adcy2	0.0000	-3.1516	0.0044
Adcy3	0.0000	1.7153	0.0178
Adcy5	0.0000	-1.5364	0.0102
Adcy8	0.0000	-2.1277	0.0221
Adcyap1r1	0.0000	-2.3456	0.0029
Add2	0.0000	-3.0928	0.0006
Adgra1	0.0000	-1.5696	0.0238
Adgrb1	0.0000	-1.3717	0.0134
Adgrb2	0.0000	-1.2492	0.0076
Adgrb3	0.0000	-1.3948	0.0192
Adgre5	0.0000	3.5406	0.0010
Adgrg1	0.0000	-1.5567	0.0065
Adgrg6	0.0000	-1.0274	0.0362
Adgrl2	0.0000	-1.0394	0.0128
Adgrl3	0.0000	-2.1505	0.0040
Adgrv1	0.0000	-1.8902	0.0045
Adhfe1	0.0000	2.2561	0.0307
Adi1	0.0000	1.7129	0.0061
Adnp	0.0000	-1.4035	0.0099
Adpgk	0.0000	1.0465	0.0141
Adra1d	0.0000	-2.2378	0.0390
Adra2a	0.0000	-1.9861	0.0160
Aebp1	0.0000	1.5505	0.0100
Afap1	0.0000	-1.9601	0.0027
Afap1l2	0.0000	1.1833	0.0432
Aff1	0.0000	1.7583	0.0044
Aff2	0.0000	-3.6133	0.0066
Aff3	0.0000	-1.1379	0.0168
Aga	0.0000	2.5112	0.0045
Agap2	0.0000	-1.8698	0.0080
Ago1	0.0000	-1.7382	0.0028
Ago4	0.0000	-2.4148	0.0081
Agpat2	0.0000	2.1829	0.0131
Agt	0.0000	3.7105	0.0431
Agtpbp1	0.0000	-1.2054	0.0166

Agtrap	0.0000	2.0157	0.0030
Ahnak2	0.0000	3.0045	0.0048
Ahrr	0.0000	4.2500	0.0415
Ahsa1	0.0000	1.3928	0.0066
Aif1l	0.0000	-1.1026	0.0227
Aifm3	0.0000	1.9244	0.0369
Aim2	0.0000	4.3310	0.0027
Aire	0.0000	4.7077	0.0120
Ajap1	0.0000	-2.4873	0.0130
Ajuba	0.0000	1.4863	0.0053
Ak7	0.0000	1.3671	0.0456
Akap11	0.0000	-1.3257	0.0057
Akap14	0.0000	2.5278	0.0367
Akap6	0.0000	-2.8418	0.0004
Akap9	0.0000	-1.9042	0.0018
Akip1	0.0000	1.5203	0.0229
Akt3	0.0000	-1.5197	0.0040
Alcam	0.0000	-1.6308	0.0310
Aldh1a2	0.0000	-2.7140	0.0073
Aldh1b1	0.0000	1.9528	0.0090
Aldh1l1	0.0000	2.4592	0.0083
Aldh2	0.0000	1.6867	0.0031
Aldh9a1	0.0000	1.1328	0.0093
Aldoa	0.0000	1.4292	0.0019
Alg13	0.0000	2.8575	0.0008
Alg2	0.0000	-1.0113	0.0118
Alk	0.0000	-2.6822	0.0310
Alkbh2	0.0000	2.1825	0.0162
Alkbh8	0.0000	-1.0728	0.0119
Alox5	0.0001	2.1499	0.0479
Alpk3	0.0000	3.8468	0.0044
Alpl	0.0000	3.0318	0.0008
Als2cl	0.0000	2.7198	0.0159
Amdhd2	0.0000	1.5860	0.0139
Amer2	0.0000	-1.4298	0.0088
Amer3	0.0000	-2.5583	0.0084
Amigo1	0.0000	-1.6698	0.0124
Amn1	0.0000	-1.2046	0.0239
Amotl2	0.0000	-1.0238	0.0100
Amt	0.0000	1.4865	0.0077
Angptl2	0.0000	-1.5133	0.0185
Angptl4	0.0000	4.6738	0.0115
Ank1	0.0000	-1.1548	0.0422
Ank2	0.0000	-2.0611	0.0015
Ank3	0.0000	-1.9286	0.0012
Ankrd13b	0.0000	-2.0369	0.0049
Ankrd35	0.0000	4.4954	0.0088
Ankrd37	0.0000	-1.0385	0.0448
Ankrd42	0.0000	-1.5510	0.0255
Ankrd45	0.0000	3.3022	0.0105
Ankrd50	0.0000	-1.2468	0.0073
Ankrd6	0.0000	-1.0472	0.0183

Ankrd9	0.0000	1.5993	0.0293
Anks1b	0.0000	-1.9146	0.0184
Anks6	0.0000	-1.1379	0.0363
Anln	0.0000	1.8047	0.0027
Ano1	0.0000	1.0830	0.0366
Ano10	0.0000	-1.2693	0.0121
Anp32b	0.0000	1.1209	0.0101
Anxa1	0.0000	1.6995	0.0133
Anxa11	0.0000	3.2099	0.0011
Anxa3	0.0000	1.2429	0.0286
Anxa4	0.0000	2.0676	0.0062
Anxa7	0.0000	1.9089	0.0045
Aoah	0.0000	5.4742	0.0304
Ap1g2	0.0000	1.4578	0.0340
Ap1m2	0.0000	2.1911	0.0356
Ap3b2	0.0000	-1.4570	0.0069
Ap3s2	0.0000	-1.0201	0.0098
Ap4s1	0.0000	-1.0599	0.0355
Apba1	0.0000	-2.0597	0.0070
Apba2	0.0000	-2.8545	0.0015
Apbb1	0.0000	-1.4569	0.0056
Apbb1ip	0.0000	4.6598	0.0319
Apc	0.0000	-1.4044	0.0037
Apc2	0.0000	-3.0121	0.0003
Aph1b	0.0000	-1.3735	0.0151
Aplp1	0.0000	-1.3805	0.0035
Apobec2	0.0000	4.1830	0.0107
Apobr	0.0000	2.6225	0.0337
Apoc1	0.0000	4.7598	0.0078
Apod	0.0000	2.8361	0.0022
Apoe	0.0000	4.2302	0.0002
Aptx	0.0000	-1.1262	0.0160
Aqp3	0.0000	4.2158	0.0026
Aqp4	0.0000	2.1048	0.0312
Arc	0.0000	4.0484	0.0048
Arel1	0.0000	-1.0815	0.0126
Arg2	0.0000	-2.6619	0.0337
Arhgap20	0.0000	-2.1204	0.0138
Arhgap22	0.0000	1.5616	0.0395
Arhgap27	0.0000	1.9920	0.0340
Arhgap28	0.0000	-1.0137	0.0252
Arhgap30	0.0000	3.3452	0.0132
Arhgap33	0.0000	-2.3782	0.0016
Arhgap35	0.0000	-1.0193	0.0081
Arhgap36	0.0000	-4.1039	0.0091
Arhgap4	0.0000	1.5139	0.0448
Arhgap6	0.0000	-2.3425	0.0359
Arhgap8	0.0001	1.7664	0.0471
Arhgap9	0.0000	4.1537	0.0216
Arhgdib	0.0000	2.5204	0.0156
Arhgef10l	0.0000	-1.9517	0.0054
Arhgef15	0.0000	2.5138	0.0276

Arhgef16	0.0000	1.4036	0.0397
Arhgef18	0.0000	1.2779	0.0056
Arhgef19	0.0000	1.4136	0.0412
Arhgef26	0.0000	-1.5073	0.0167
Arhgef3	0.0000	1.8818	0.0150
Arhgef4	0.0000	-1.1217	0.0213
Arid1b	0.0000	-1.0301	0.0113
Arid2	0.0000	-1.0082	0.0104
Arid3b	0.0000	1.5242	0.0091
Arid4b	0.0000	-1.2082	0.0082
Arid5a	0.0000	2.2845	0.0224
Arid5b	0.0000	1.0860	0.0142
Arl10	0.0000	-1.1748	0.0231
Arl16	0.0000	-1.3405	0.0267
Arl4d	0.0000	-1.7087	0.0144
Arl5c	0.0000	2.0143	0.0366
Armc3	0.0000	3.2328	0.0360
Armcx3	0.0000	-1.0842	0.0112
Armcx4	0.0000	-1.4145	0.0045
Arntl	0.0000	1.1429	0.0330
Arpc1b	0.0000	2.0888	0.0041
Arrb1	0.0000	-1.7892	0.0036
Asah1	0.0000	1.4575	0.0050
Asb13	0.0000	-1.5201	0.0166
Ascl1	0.0000	-1.4982	0.0086
Ascl2	0.0000	2.1674	0.0397
Asf1b	0.0000	1.1947	0.0234
Ash1l	0.0000	-1.0077	0.0144
Ash2l	0.0000	1.8453	0.0014
Asic1	0.0000	-2.4562	0.0012
Asic4	0.0000	-2.8071	0.0024
Asl	0.0000	1.0499	0.0257
Asna1	0.0000	1.7026	0.0049
Asns	0.0000	2.5217	0.0004
Aspa	0.0000	3.1759	0.0456
Asphd1	0.0000	-2.3724	0.0264
Ass1	0.0000	3.9807	0.0003
Astn1	0.0000	-2.0955	0.0029
Astn2	0.0000	-1.5730	0.0145
Asxl3	0.0000	-3.3444	0.0015
Atad2	0.0000	1.0234	0.0136
Atat1	0.0000	-1.9218	0.0031
Atcay	0.0000	-2.4665	0.0020
Atf1	0.0000	1.6662	0.0067
Atf4	0.0000	1.0527	0.0088
Atf5	0.0000	2.6907	0.0003
Atf7ip2	0.0000	3.6352	0.0157
Atic	0.0000	1.5295	0.0046
Atmin	0.0000	1.1163	0.0122
Atp10a	0.0000	1.4721	0.0286
Atp10d	0.0000	1.6854	0.0120
Atp13a2	0.0000	-1.2015	0.0088

Atp1a3	0.0000	-1.0485	0.0148
Atp1b1	0.0000	1.3764	0.0060
Atp2a3	0.0000	2.5781	0.0099
Atp2b2	0.0000	-1.3149	0.0229
Atp2c2	0.0000	2.5940	0.0425
Atp6v0a4	0.0000	3.6574	0.0329
Atp6v1g2	0.0000	-1.5001	0.0275
Atp7a	0.0000	-1.0287	0.0187
Atp8a1	0.0000	-1.3120	0.0125
Atp8a2	0.0000	-2.8747	0.0048
Atrn	0.0000	-1.2194	0.0097
Atrnl1	0.0000	-1.6327	0.0051
Atxn7l3b	0.0000	-1.2555	0.0036
Aunip	0.0000	2.5836	0.0111
Aurka	0.0000	1.3797	0.0117
Aurkb	0.0000	1.0613	0.0217
Auts2	0.0000	-1.9154	0.0026
Avl9	0.0000	-1.4369	0.0063
Avpi1	0.0000	2.8698	0.0025
B2m	0.0000	1.7387	0.0078
B3galt1	0.0000	-1.5385	0.0221
B3galt2	0.0001	-1.0951	0.0480
B3galt4	0.0002	-1.0091	0.0497
B3galt5	0.0000	-2.9937	0.0298
B3gat1	0.0000	-1.8134	0.0104
B3gnt2	0.0000	3.6135	0.0021
B3gnt7	0.0000	4.1940	0.0004
B4galnt3	0.0000	2.5857	0.0167
B4galt1	0.0000	1.2825	0.0131
B4galt2	0.0000	-1.6723	0.0073
Bace1	0.0000	-1.1963	0.0101
Bach2	0.0000	-2.7211	0.0018
Bag3	0.0000	1.2341	0.0177
Bag4	0.0000	1.0998	0.0125
Bambi	0.0000	1.2240	0.0289
Bard1	0.0000	2.1515	0.0044
Basp1	0.0000	-1.5987	0.0053
Bbs1	0.0000	-1.0352	0.0267
Bcam	0.0000	1.8091	0.0071
Bcar3	0.0000	1.9094	0.0072
Bcas1	0.0000	2.9089	0.0218
Bcat1	0.0000	1.9449	0.0041
Bcat2	0.0000	2.0368	0.0058
Bckdhb	0.0000	1.5147	0.0112
Bcl11a	0.0000	-2.1182	0.0048
Bcl11b	0.0000	-1.4047	0.0258
Bcl3	0.0000	3.4306	0.0138
Bcl7a	0.0000	-2.0102	0.0019
Bcl9	0.0000	-1.3339	0.0067
Bcr	0.0000	-1.0815	0.0144
Bdh2	0.0000	1.9472	0.0322
Bean1	0.0000	-2.7736	0.0209

Begain	0.0000	-1.8960	0.0148
Bend5	0.0000	-1.7555	0.0130
Bend7	0.0000	-1.2041	0.0373
Bex1	0.0000	1.6043	0.0069
Bex4	0.0000	1.1620	0.0119
Bgn	0.0000	2.5599	0.0020
Bhlhe22	0.0000	-2.8907	0.0022
Bhlhe40	0.0000	1.4187	0.0063
Bhlhe41	0.0000	1.2327	0.0257
Bicd1	0.0000	-1.5065	0.0111
Bicd2	0.0000	-1.4546	0.0063
Bid	0.0000	1.3541	0.0160
Bin2	0.0000	5.2882	0.0045
Birc3	0.0000	1.7402	0.0340
Birc5	0.0000	1.1086	0.0180
Blcap	0.0000	-1.3792	0.0056
Blnk	0.0000	4.3487	0.0404
Blvrb	0.0000	1.4978	0.0162
Bmp1	0.0000	-1.3984	0.0070
Bmpr1b	0.0000	-1.2526	0.0264
Bok	0.0000	1.6256	0.0228
Brca1	0.0000	1.1847	0.0134
Brinp1	0.0000	-3.0208	0.0091
Brip1	0.0000	1.1056	0.0278
Brsk1	0.0000	-1.8536	0.0033
Brsk2	0.0000	-2.8475	0.0010
Bsn	0.0000	-3.6199	0.0007
Bst2	0.0000	2.4854	0.0043
Btbd19	0.0000	1.7903	0.0196
Btg3	0.0000	1.0557	0.0222
Bub1	0.0000	1.4891	0.0056
Bub1b	0.0000	1.3785	0.0072
Bves	0.0001	-1.3550	0.0485
Bzw2	0.0000	-1.1097	0.0073
C1ql3	0.0000	-3.4313	0.0214
C1qtnf1	0.0000	1.9953	0.0433
C1qtnf6	0.0000	1.5803	0.0136
C1rl	0.0000	2.3039	0.0400
C2	0.0000	3.8299	0.0123
C2cd2l	0.0000	-1.5908	0.0178
C3	0.0000	6.2696	0.0109
Cables2	0.0000	-1.0490	0.0222
Cacna1b	0.0000	-2.4130	0.0036
Cacna1c	0.0000	-2.0284	0.0088
Cacna1d	0.0000	-1.4918	0.0326
Cacna1e	0.0000	-1.6995	0.0228
Cacna1g	0.0000	-1.4449	0.0100
Cacna1h	0.0000	-2.1162	0.0028
Cacna1i	0.0000	-1.6927	0.0385
Cacna2d2	0.0000	-2.0858	0.0044
Cacna2d3	0.0000	-3.2583	0.0036
Cacnb1	0.0000	-2.0168	0.0071

Cacnb3	0.0000	-1.0034	0.0138
Cacnb4	0.0000	-2.2014	0.0183
Cacng2	0.0000	-2.4694	0.0095
Cacng4	0.0000	-2.2011	0.0021
Cacng6	0.0000	4.3766	0.0181
Cacng7	0.0000	-1.0472	0.0136
Cadm1	0.0000	-1.4804	0.0045
Cadm2	0.0000	-2.8341	0.0081
Cadm3	0.0000	-2.2237	0.0029
Cadm4	0.0000	-2.2060	0.0016
Cadps	0.0000	-2.9023	0.0017
Cadps2	0.0000	1.4558	0.0300
Calb1	0.0000	-1.9965	0.0446
Calcoco2	0.0000	4.2680	0.0019
Calhm2	0.0000	3.4285	0.0287
Calml4	0.0000	2.5241	0.0191
Camk1	0.0000	1.3775	0.0213
Camk1d	0.0000	1.4126	0.0136
Camk1g	0.0000	-1.6156	0.0225
Camk2a	0.0000	-1.6907	0.0196
Camk2g	0.0000	-1.0443	0.0139
Camk2n2	0.0000	-1.1330	0.0328
Camk4	0.0000	-1.3226	0.0374
Camsap2	0.0000	-1.1995	0.0064
Camta1	0.0000	-1.7475	0.0047
Capg	0.0000	4.7844	0.0075
Capn1	0.0000	2.2724	0.0048
Capn12	0.0001	3.2921	0.0481
Capn3	0.0000	3.5715	0.0281
Capsl	0.0000	2.9876	0.0169
Card6	0.0000	1.2838	0.0458
Carhsp1	0.0000	-1.6068	0.0027
Carnmt1	0.0000	1.5432	0.0088
Cars	0.0000	1.4891	0.0047
Cartpt	0.0000	-2.3048	0.0193
Casd1	0.0000	-1.2119	0.0136
Caskin1	0.0000	-1.5641	0.0135
Casp12	0.0000	1.7580	0.0448
Casp8	0.0000	2.4343	0.0219
Casq1	0.0000	2.4563	0.0447
Catip	0.0000	3.3088	0.0346
Catsperd	0.0000	2.7923	0.0272
Cav2	0.0000	2.7234	0.0354
Cavin2	0.0000	1.7424	0.0217
Cbarp	0.0000	-1.2094	0.0130
Cblb	0.0000	-1.6485	0.0065
Cbln1	0.0000	-1.7804	0.0050
Cbln2	0.0000	-4.4498	0.0073
Cbln4	0.0000	-1.7937	0.0270
Cbr3	0.0000	3.7696	0.0132
Cbs	0.0000	2.1427	0.0158
Cbx7	0.0000	3.6829	0.0007

Cc2d1b	0.0000	1.1597	0.0186
Ccar2	0.0000	-1.3640	0.0050
Ccbe1	0.0000	-2.4621	0.0255
Ccdc102a	0.0000	1.2049	0.0256
Ccdc113	0.0000	4.9417	0.0250
Ccdc114	0.0000	2.7511	0.0133
Ccdc125	0.0000	3.4932	0.0173
Ccdc136	0.0000	-1.6239	0.0102
Ccdc14	0.0000	1.0318	0.0260
Ccdc141	0.0000	2.8473	0.0129
Ccdc142	0.0000	-2.6872	0.0439
Ccdc160	0.0000	1.3545	0.0254
Ccdc170	0.0000	3.2309	0.0412
Ccdc18	0.0000	1.1457	0.0289
Ccdc184	0.0000	-2.7897	0.0080
Ccdc24	0.0000	-1.7893	0.0418
Ccdc28b	0.0000	-2.0916	0.0069
Ccdc36	0.0000	4.0969	0.0248
Ccdc40	0.0000	-1.0299	0.0335
Ccdc43	0.0000	1.0648	0.0146
Ccdc50	0.0000	-1.3799	0.0049
Ccdc68	0.0000	4.9532	0.0202
Ccdc80	0.0000	2.0659	0.0404
Ccdc82	0.0000	-1.1109	0.0271
Ccdc85c	0.0000	-1.2496	0.0074
Ccdc86	0.0000	1.4114	0.0126
Ccdc88a	0.0000	-1.1146	0.0094
Cck	0.0000	4.7026	0.0235
Cckbr	0.0000	2.9049	0.0457
Ccl2	0.0000	5.8522	0.0162
Ccna1	0.0000	2.2158	0.0450
Ccnb1	0.0000	1.2376	0.0126
Ccnb1ip1	0.0000	3.8530	0.0114
Ccnb2	0.0000	1.6088	0.0084
Ccnd3	0.0000	1.1562	0.0079
Ccnf	0.0000	1.7698	0.0052
Ccng2	0.0000	-1.3350	0.0067
Ccnjl	0.0000	-2.1616	0.0117
Ccser1	0.0000	-2.7876	0.0085
Cd2ap	0.0000	1.2835	0.0089
Cd34	0.0000	2.0183	0.0246
Cd38	0.0000	3.9224	0.0107
Cd44	0.0000	1.7536	0.0127
Cd55	0.0000	2.2402	0.0101
Cd68	0.0000	4.1546	0.0110
Cd74	0.0000	3.2768	0.0438
Cd83	0.0000	-1.6524	0.0424
Cd84	0.0000	6.4706	0.0332
Cd9	0.0000	2.7697	0.0007
Cd99l2	0.0000	-1.0382	0.0121
Cdc14b	0.0000	1.2626	0.0182
Cdc20	0.0000	1.2720	0.0090

Cdc25b	0.0000	-1.1490	0.0136
Cdc42ep2	0.0000	-1.3771	0.0375
Cdc42ep4	0.0000	-1.1815	0.0128
Cdca2	0.0000	1.3391	0.0097
Cdca3	0.0000	1.2664	0.0188
Cdca5	0.0000	1.6749	0.0096
Cdca7	0.0000	1.1539	0.0136
Cdcp1	0.0000	2.7755	0.0307
Cdh1	0.0000	3.5969	0.0001
Cdh10	0.0000	-2.5021	0.0092
Cdh12	0.0000	-3.3675	0.0314
Cdh13	0.0000	-1.3008	0.0171
Cdh18	0.0000	-2.2633	0.0311
Cdh2	0.0000	-2.1915	0.0005
Cdh22	0.0000	-1.8172	0.0110
Cdh3	0.0000	2.8214	0.0067
Cdh4	0.0000	-1.9955	0.0036
Cdh6	0.0000	-1.7931	0.0053
Cdh7	0.0000	-3.5950	0.0199
Cdh8	0.0000	-4.2293	0.0069
Cdhr1	0.0000	3.3477	0.0114
Cdk1	0.0000	1.3253	0.0106
Cdk14	0.0000	-1.2262	0.0107
Cdk17	0.0000	-1.1846	0.0131
Cdk18	0.0000	2.2443	0.0306
Cdk5r1	0.0000	-2.4778	0.0028
Cdk5rap1	0.0000	1.2219	0.0243
Cdkn1c	0.0000	-1.3585	0.0055
Cdkn2a	0.0000	3.4181	0.0016
Cdkn3	0.0000	1.3358	0.0348
Cds1	0.0000	1.8270	0.0144
Cdx1	0.0000	2.5665	0.0432
Cdyl	0.0000	1.1060	0.0133
Cdyl2	0.0000	2.9330	0.0060
Ceacam1	0.0000	4.5507	0.0179
Cebpb	0.0000	2.8258	0.0065
Cebpd	0.0000	2.4085	0.0140
Celf2	0.0000	-1.6066	0.0050
Celf3	0.0000	-3.3617	0.0009
Celf4	0.0000	-2.0685	0.0020
Celf5	0.0000	-1.4517	0.0087
Celf6	0.0000	-1.8693	0.0188
Celsr2	0.0000	-2.2265	0.0024
Celsr3	0.0000	-2.9532	0.0008
Cend1	0.0000	-2.5531	0.0087
Cenpn	0.0000	1.4821	0.0141
Cenpw	0.0000	1.9108	0.0105
Cep112	0.0000	1.8002	0.0175
Cep170	0.0000	-1.7543	0.0024
Cep55	0.0000	1.1826	0.0145
Cerk	0.0000	-1.3745	0.0119
Cers4	0.0000	1.1781	0.0155

Cers6	0.0000	-1.2698	0.0086
Cfap161	0.0000	4.3172	0.0460
Cfap52	0.0000	2.6845	0.0257
Cfap70	0.0000	6.1864	0.0173
Cfh	0.0000	1.1316	0.0226
Cfl2	0.0000	-1.0124	0.0100
Cgn	0.0000	1.8764	0.0109
Ch25h	0.0000	3.1645	0.0435
Chac1	0.0000	2.4459	0.0090
Chaf1b	0.0000	1.2721	0.0210
Chchd4	0.0000	1.2140	0.0127
Chd3	0.0000	-1.4122	0.0105
Chd7	0.0000	-1.5127	0.0075
Chek1	0.0000	1.2598	0.0153
Chek2	0.0000	1.3909	0.0211
Chga	0.0000	-1.2097	0.0154
Chl1	0.0000	-2.5794	0.0047
Chmp3	0.0000	1.0624	0.0132
Chn1	0.0000	-1.5843	0.0050
Chrdl1	0.0000	-1.1603	0.0418
Chrm3	0.0001	-1.7184	0.0480
Chrna3	0.0000	-3.7641	0.0076
Chrna4	0.0000	-2.5446	0.0018
Chrna5	0.0000	-3.1443	0.0101
Chrnb2	0.0000	-2.8408	0.0011
Chrnb4	0.0000	-1.9700	0.0265
Chst11	0.0000	-1.3975	0.0142
Chsy3	0.0000	-1.6908	0.0292
Chtf18	0.0000	1.7487	0.0093
Cib2	0.0000	-1.3482	0.0336
Cilp2	0.0000	1.6848	0.0282
Cirbp	0.0000	-1.2728	0.0107
Ckb	0.0000	-1.1049	0.0089
Cks2	0.0000	1.2202	0.0142
Clasp1	0.0000	-1.1020	0.0068
Clasp2	0.0000	-1.2183	0.0076
Clcn4	0.0000	-1.7856	0.0027
Cldn11	0.0000	2.4290	0.0104
Cldn19	0.0000	3.5801	0.0398
Cldn2	0.0000	2.3781	0.0432
Cldn4	0.0000	4.4738	0.0004
Clec11a	0.0000	2.5391	0.0202
Clec1a	0.0000	2.5772	0.0449
Clec2l	0.0000	-1.4601	0.0459
Clgn	0.0000	1.3863	0.0259
Clic5	0.0000	2.5954	0.0386
Clip1	0.0000	-1.0338	0.0134
Clip2	0.0000	-2.1232	0.0016
Clip3	0.0000	-1.6831	0.0041
Clmp	0.0000	-1.4052	0.0137
Cln5	0.0000	1.7847	0.0042
Clstn2	0.0000	-2.0466	0.0189

Clstn3	0.0000	-1.0463	0.0194
Clu	0.0000	2.5704	0.0025
Clvs1	0.0000	-2.2169	0.0063
Cmklr1	0.0000	1.0373	0.0433
Cmpk2	0.0000	-1.2306	0.0406
Cmtm6	0.0000	1.7912	0.0041
Cmtm7	0.0000	1.5021	0.0183
Cmtm8	0.0000	-1.5011	0.0220
Cnksr2	0.0000	-1.8242	0.0202
Cnot6	0.0000	-1.1892	0.0075
Cnpy3	0.0000	1.1073	0.0134
Cnr1	0.0000	-2.8234	0.0025
Cntd1	0.0000	3.7057	0.0114
Cntfr	0.0000	-1.2757	0.0086
Cntn2	0.0000	-1.8763	0.0020
Cntn4	0.0000	-3.7659	0.0288
Cntn6	0.0000	-4.0081	0.0377
Cntnap2	0.0000	-1.8762	0.0097
Cobl	0.0000	3.1760	0.0003
Col10a1	0.0000	6.0336	0.0386
Col11a1	0.0000	-2.0333	0.0062
Col1a1	0.0000	1.1914	0.0306
Col1a2	0.0000	1.5852	0.0298
Col23a1	0.0000	-1.7639	0.0190
Col25a1	0.0000	1.1573	0.0431
Col5a2	0.0000	3.7647	0.0005
Col5a3	0.0000	4.5969	0.0137
Col6a2	0.0000	1.1502	0.0195
Col6a5	0.0000	6.9188	0.0044
Col8a2	0.0000	1.5172	0.0421
Col9a1	0.0000	-1.8487	0.0221
Col9a2	0.0000	1.5766	0.0233
Col9a3	0.0000	1.6625	0.0344
Colgalt1	0.0000	1.1593	0.0067
Coq3	0.0000	1.4740	0.0153
Corin	0.0000	2.3140	0.0056
Coro1a	0.0000	-1.9920	0.0153
Coro6	0.0000	3.4418	0.0345
Cox17	0.0000	1.1968	0.0160
Cox19	0.0000	-1.7322	0.0058
Cox7a1	0.0000	2.5614	0.0265
Cp	0.0000	1.7976	0.0083
Cpa2	0.0001	1.8065	0.0477
Cpeb1	0.0000	1.7181	0.0139
Cpeb2	0.0000	-1.1871	0.0148
Cpeb3	0.0000	-1.2260	0.0334
Cpeb4	0.0000	-1.8899	0.0043
Cped1	0.0000	1.1330	0.0341
Cplx1	0.0000	-1.6917	0.0187
Cplx2	0.0000	-1.9446	0.0029
Cpm	0.0000	-1.0975	0.0362
Cpn1	0.0000	5.0332	0.0239

Cpne9	0.0000	3.7791	0.0309
Cpq	0.0000	3.4876	0.0099
Cpt1a	0.0000	2.9296	0.0010
Crabp1	0.0000	-2.1134	0.0043
Crabp2	0.0000	-2.2552	0.0233
Cramp1l	0.0000	-1.1237	0.0165
Crb1	0.0000	-2.5025	0.0062
Crb2	0.0000	-1.7491	0.0088
Creb5	0.0000	-1.1547	0.0374
Crebbp	0.0000	-1.0590	0.0180
Creg1	0.0000	1.4347	0.0184
Crhbp	0.0002	-1.1595	0.0499
Crim1	0.0000	-1.3971	0.0104
Crispld1	0.0000	3.1152	0.0026
Crlf3	0.0000	1.8372	0.0061
Crmp1	0.0000	-3.2096	0.0002
Crybg3	0.0000	-1.4321	0.0134
Crym	0.0000	1.7976	0.0297
Csdc2	0.0000	-1.5952	0.0100
Csf1	0.0000	1.1553	0.0182
Csf1r	0.0000	2.8626	0.0435
Csmd2	0.0000	-2.8811	0.0065
Cspg4	0.0000	2.3283	0.0271
Cspg5	0.0000	-1.1173	0.0175
Csrnp1	0.0000	1.9517	0.0161
Csrnp2	0.0000	-1.5109	0.0085
Csrnp3	0.0000	-2.4505	0.0019
Csrp2	0.0000	1.6063	0.0038
Cstb	0.0000	1.2578	0.0126
Ctbp1	0.0000	-1.0941	0.0074
Ctbs	0.0000	1.3695	0.0204
Cth	0.0000	4.5167	0.0004
Ctif	0.0000	-1.7531	0.0053
Ctnna2	0.0000	-2.7321	0.0012
Ctnnbip1	0.0000	-1.3095	0.0126
Ctnnd2	0.0000	-1.5477	0.0082
Ctsa	0.0000	1.4556	0.0053
Ctsc	0.0000	3.5320	0.0040
Ctsf	0.0000	-1.4314	0.0161
Ctsz	0.0000	1.5807	0.0078
Cttnbp2	0.0000	-3.0694	0.0065
Cuedc1	0.0000	-1.0793	0.0147
Cul4b	0.0000	2.2268	0.0008
Cux1	0.0000	-1.2296	0.0078
Cux2	0.0000	-2.4139	0.0042
Cx3cl1	0.0000	1.4569	0.0159
Cxadr	0.0000	-1.4403	0.0051
Cxcl1	0.0000	7.7112	0.0166
Cxcl12	0.0000	-1.4991	0.0108
Cxcl16	0.0000	1.7290	0.0358
Cxxc4	0.0000	-2.5967	0.0012
Cyb561d1	0.0000	-1.2062	0.0330

Cyb5d1	0.0000	-1.0999	0.0371
Cyb5r1	0.0000	2.7046	0.0009
Cyba	0.0000	3.7430	0.0033
Cybrd1	0.0000	1.7590	0.0312
Cyfip2	0.0000	-1.7857	0.0023
Cyp27a1	0.0000	4.5482	0.0234
Cyp2s1	0.0000	1.0127	0.0449
Cyp46a1	0.0000	-1.4884	0.0317
Cyth1	0.0000	-1.9104	0.0073
Cyyr1	0.0000	1.7385	0.0361
Daam1	0.0000	-1.3064	0.0065
Dab1	0.0000	-1.7229	0.0074
Dab2	0.0000	-1.0896	0.0274
Dach1	0.0000	-2.7281	0.0021
Dach2	0.0000	-3.1071	0.0097
Dact2	0.0000	4.1496	0.0018
Daglb	0.0000	-1.0444	0.0368
Dapk2	0.0001	2.3910	0.0480
Dapp1	0.0000	3.4216	0.0229
Dbf4	0.0000	1.2745	0.0094
Dbn1	0.0000	-1.9882	0.0011
Dbndd1	0.0000	-2.3064	0.0091
Dbndd2	0.0000	-2.2186	0.0175
Dbp	0.0000	1.1480	0.0249
Dbx2	0.0000	-2.8351	0.0315
Dcaf7	0.0000	-1.1745	0.0051
Dcc	0.0000	-2.5508	0.0009
Dchs1	0.0000	-1.5215	0.0042
Dchs2	0.0000	-3.0792	0.0062
Dclk1	0.0000	-2.0819	0.0026
Dctd	0.0000	2.7425	0.0037
Dctpp1	0.0000	1.2312	0.0159
Dcun1d4	0.0000	-1.4599	0.0069
Dcx	0.0000	-3.9595	0.0002
Dcxr	0.0000	1.2898	0.0222
Ddah2	0.0000	-1.1128	0.0114
Ddit3	0.0000	2.2349	0.0053
Ddn	0.0000	2.2369	0.0266
Ddx25	0.0000	-1.1729	0.0216
Ddx58	0.0000	1.5578	0.0166
Deaf1	0.0000	-1.3871	0.0135
Degs1	0.0000	1.3510	0.0077
Dennd2c	0.0000	3.2552	0.0011
Dennd2d	0.0001	2.9923	0.0467
Dennd3	0.0001	1.3039	0.0473
Dennd5b	0.0000	-1.9625	0.0042
Depdc7	0.0000	1.8981	0.0217
Deptor	0.0000	3.9135	0.0022
Dgka	0.0000	2.1973	0.0097
Dgkb	0.0000	-2.3501	0.0189
Dgke	0.0000	-1.0183	0.0257
Dgki	0.0000	-2.2077	0.0324

Dhdh	0.0000	3.2359	0.0049
Dhodh	0.0000	1.1434	0.0189
Dhtkd1	0.0000	1.8089	0.0100
Diaph1	0.0000	1.4146	0.0047
Dio2	0.0000	3.0748	0.0379
Dio3	0.0000	1.6916	0.0365
Dip2b	0.0000	-1.2061	0.0095
Diras1	0.0000	-2.5357	0.0069
Diras2	0.0000	-1.3392	0.0196
Disp2	0.0000	-1.4053	0.0125
Dixdc1	0.0000	-1.0594	0.0198
Dkc1	0.0000	1.8997	0.0021
Dkk3	0.0000	1.8115	0.0057
Dlc1	0.0000	2.4676	0.0011
Dleu2	0.0000	1.0685	0.0429
Dleu7	0.0000	1.7768	0.0459
Dlg2	0.0000	-2.3902	0.0190
Dlg3	0.0000	-1.1544	0.0128
Dlg4	0.0000	-1.6991	0.0032
Dlgap1	0.0000	-1.0761	0.0358
Dlgap5	0.0000	1.7171	0.0047
Dll1	0.0000	-1.9811	0.0054
Dll3	0.0000	-1.2785	0.0238
Dll4	0.0000	-2.3559	0.0270
Dmbx1	0.0000	2.2920	0.0236
Dmc1	0.0000	3.6622	0.0185
Dmd	0.0000	1.9123	0.0050
Dmgdh	0.0000	2.3285	0.0461
Dmrta2	0.0000	2.6734	0.0082
Dmtn	0.0000	1.6224	0.0090
Dmxl2	0.0000	-1.3568	0.0092
Dnah5	0.0000	-2.3401	0.0451
Dnah6	0.0000	3.1937	0.0349
Dnah8	0.0000	3.4094	0.0032
Dnah9	0.0000	-1.7955	0.0230
Dnajb5	0.0000	-1.1720	0.0136
Dnajc12	0.0000	-1.0893	0.0298
Dnajc21	0.0000	1.5136	0.0066
Dnajc22	0.0000	2.4009	0.0325
Dnajc28	0.0000	-1.7617	0.0276
Dnajc5	0.0000	-1.2361	0.0059
Dner	0.0000	-1.9436	0.0039
Dnm1	0.0000	-1.5848	0.0104
Dnm3	0.0000	-2.9319	0.0055
Dnmt1	0.0000	-5.8368	0.0002
Dnmt3b	0.0000	1.1720	0.0182
Dnmt3l	0.0000	3.5679	0.0018
Dnph1	0.0000	1.5157	0.0128
Doc2b	0.0000	-2.3444	0.0165
Dock10	0.0000	1.0245	0.0435
Dock2	0.0001	2.9001	0.0477
Dock3	0.0000	-2.1842	0.0088

Dock4	0.0000	-1.9894	0.0050
Dock5	0.0000	1.2428	0.0155
Dock6	0.0000	1.5202	0.0055
Dock7	0.0000	-2.2543	0.0012
Dock9	0.0000	1.2150	0.0191
Dohh	0.0000	1.0015	0.0203
Dok1	0.0000	1.3165	0.0301
Dok4	0.0000	-1.2820	0.0204
Dok5	0.0000	-2.0523	0.0129
Dok6	0.0000	-2.7916	0.0137
Dpf1	0.0000	-1.8358	0.0070
Dpf3	0.0000	-2.6048	0.0108
Dph6	0.0000	1.1318	0.0205
Dpp10	0.0000	-1.4086	0.0304
Dpp4	0.0000	1.2827	0.0385
Dpp8	0.0000	-1.4000	0.0072
Dppa2	0.0000	3.0829	0.0062
Dppa4	0.0000	4.8634	0.0011
Dpys	0.0000	5.3588	0.0165
Dpysl2	0.0000	-1.9513	0.0030
Dpysl3	0.0000	-2.4334	0.0003
Dpysl4	0.0000	-3.5663	0.0004
Dpysl5	0.0000	-3.0449	0.0002
Draxin	0.0000	-2.5397	0.0032
Drc1	0.0000	3.3312	0.0162
Dscam	0.0000	-1.8690	0.0130
Dse	0.0000	1.4877	0.0095
Dsg2	0.0000	3.6110	0.0015
Dsp	0.0000	2.4512	0.0035
Dtl	0.0000	1.1876	0.0113
Dtwd1	0.0000	1.1067	0.0310
Dtwd2	0.0000	1.3108	0.0338
Dtx3	0.0000	-1.0997	0.0107
Dtx3l	0.0000	1.6684	0.0288
Dtx4	0.0000	-2.3628	0.0021
Dtymk	0.0000	1.4180	0.0051
Dusp10	0.0000	-1.2500	0.0280
Dusp27	0.0000	2.8490	0.0034
Dusp4	0.0000	-1.4942	0.0064
Dusp5	0.0000	1.3571	0.0330
Dusp8	0.0000	-2.1057	0.0019
Dusp9	0.0000	2.7638	0.0016
Dvl3	0.0000	-1.4637	0.0059
Dync1i2	0.0000	-1.0368	0.0090
Dync2h1	0.0000	-1.4695	0.0114
Dyrk2	0.0000	-1.0655	0.0125
Dyrk3	0.0000	1.5142	0.0108
E2f2	0.0000	-1.5539	0.0211
Ebf1	0.0000	-2.0242	0.0032
Ebf3	0.0000	-3.2069	0.0004
Ebf4	0.0000	-1.1693	0.0347
Ebp	0.0000	1.6143	0.0098

Echdc2	0.0000	1.4490	0.0220
Echdc3	0.0001	2.5608	0.0471
Ecm2	0.0000	1.9842	0.0314
Ect2	0.0000	1.2583	0.0117
Edil3	0.0000	-1.8608	0.0075
Ednrb	0.0000	-1.4018	0.0085
Eed	0.0000	1.1609	0.0096
Eef1a2	0.0000	-1.1157	0.0303
Eef2k	0.0000	-1.0657	0.0177
Efcab10	0.0000	3.5609	0.0457
Efcab12	0.0000	2.1629	0.0463
Efcc1	0.0000	-2.5100	0.0192
Efhc1	0.0000	2.5234	0.0160
Efhc2	0.0000	3.2145	0.0194
Efhd1	0.0000	2.4982	0.0236
Efna5	0.0000	-1.4333	0.0159
Efr3b	0.0000	-1.7240	0.0056
Egfl7	0.0000	2.0903	0.0099
Egr1	0.0000	-2.0125	0.0364
Ehbp1	0.0000	-1.0420	0.0147
Ehd3	0.0000	-1.2124	0.0253
Ehd4	0.0000	1.3203	0.0139
Eid1	0.0000	-1.1894	0.0065
Eif1b	0.0000	-1.0121	0.0134
Eif2d	0.0000	1.1887	0.0116
Eif2s2	0.0000	1.3112	0.0034
Eif4e3	0.0000	-1.1403	0.0344
Eif4ebp1	0.0000	3.6064	0.0001
Eif4g3	0.0000	-1.1021	0.0078
Elavl2	0.0000	-2.1649	0.0021
Elavl3	0.0000	-2.6410	0.0006
Elavl4	0.0000	-2.7766	0.0004
Elf4	0.0000	1.6276	0.0215
Elfn1	0.0000	-1.6893	0.0258
Elfn2	0.0000	-1.5499	0.0206
Elk1	0.0000	1.1556	0.0127
Ell2	0.0000	2.3123	0.0040
Ell3	0.0000	3.2264	0.0198
Elmod1	0.0000	-3.2938	0.0086
Eln	0.0000	3.8346	0.0009
Elovl2	0.0000	-1.0738	0.0244
Elovl4	0.0000	-1.1142	0.0194
Elovl7	0.0000	4.4652	0.0041
Emb	0.0000	1.8977	0.0028
Eml1	0.0000	-1.8677	0.0047
Emp1	0.0000	1.5791	0.0074
Emp2	0.0000	2.5467	0.0088
Emp3	0.0000	1.3651	0.0344
Enc1	0.0000	-1.4307	0.0047
Endov	0.0000	-1.7953	0.0061
Eng	0.0000	4.6401	0.0021
Enho	0.0000	-2.0396	0.0057

Enkd1	0.0000	1.0085	0.0322
Enkur	0.0001	1.6052	0.0472
Enoph1	0.0000	1.0497	0.0174
Enox2	0.0000	-1.2586	0.0203
Eomes	0.0000	3.5890	0.0116
Epb41	0.0000	-1.8150	0.0037
Epc1	0.0000	-1.1569	0.0118
Epha10	0.0000	-1.7115	0.0173
Epha2	0.0000	2.2224	0.0025
Epha3	0.0000	-2.9159	0.0016
Epha5	0.0000	-4.3128	0.0009
Epha7	0.0000	-1.8716	0.0041
Epha8	0.0000	-3.5267	0.0075
Ephb1	0.0000	-2.9565	0.0013
Ephb2	0.0000	-2.2004	0.0012
Ephx1	0.0000	2.1429	0.0093
Ephx2	0.0000	4.5421	0.0005
Epm2aip1	0.0000	-1.2383	0.0114
Eprs	0.0000	1.0603	0.0062
Eps8	0.0000	1.1413	0.0280
Erbb3	0.0000	2.0456	0.0184
Erbb4	0.0000	-2.9965	0.0031
Erc2	0.0000	-1.9766	0.0060
Erich3	0.0000	1.7339	0.0372
Errfi1	0.0000	-1.2180	0.0148
Espl1	0.0000	1.3263	0.0097
Esrrb	0.0000	4.4183	0.0001
Esrrg	0.0000	-4.2594	0.0019
Esyt3	0.0000	3.1416	0.0208
Etv4	0.0000	2.9342	0.0055
Etv5	0.0000	3.4113	0.0001
Eva1a	0.0002	1.1766	0.0498
Evc	0.0000	2.1384	0.0079
Evc2	0.0000	2.2425	0.0073
Evl	0.0000	-1.4629	0.0046
Exoc5	0.0000	-1.0337	0.0128
Exoc7	0.0000	-1.0544	0.0156
Exog	0.0000	-1.0554	0.0235
Exosc5	0.0000	1.5597	0.0094
Extl1	0.0000	2.1046	0.0353
Eya2	0.0000	-1.1597	0.0363
Eya4	0.0000	-2.0136	0.0255
Ezr	0.0000	1.0043	0.0111
F2rl1	0.0000	4.5261	0.0039
Fabp3	0.0000	3.8461	0.0003
Fabp5	0.0000	-1.4381	0.0057
Fabp7	0.0000	-1.5515	0.0042
Fads6	0.0000	4.9714	0.0060
Fah	0.0000	2.9021	0.0080
Faim2	0.0000	-2.1744	0.0267
Fam102b	0.0000	-1.2377	0.0149
Fam107a	0.0000	2.4373	0.0386

Fam110a	0.0000	-1.2986	0.0195
Fam111a	0.0000	1.0014	0.0241
Fam117a	0.0000	1.7352	0.0124
Fam122b	0.0000	1.5263	0.0087
Fam124a	0.0000	-1.4857	0.0249
Fam129a	0.0000	2.7088	0.0099
Fam131a	0.0000	-1.3097	0.0221
Fam131b	0.0000	-2.4417	0.0052
Fam135b	0.0000	-3.9117	0.0177
Fam13b	0.0000	-1.1138	0.0216
Fam149a	0.0000	1.2865	0.0315
Fam155a	0.0000	-1.9154	0.0125
Fam160a1	0.0001	1.3328	0.0488
Fam161a	0.0000	1.9453	0.0192
Fam169b	0.0000	3.6516	0.0209
Fam171a1	0.0000	-1.0118	0.0142
Fam171a2	0.0000	-1.6058	0.0052
Fam171b	0.0000	-1.9286	0.0024
Fam178b	0.0000	6.1881	0.0050
Fam181a	0.0000	-2.2395	0.0167
Fam181b	0.0000	-1.6431	0.0053
Fam189a1	0.0000	-1.8612	0.0143
Fam189b	0.0000	-1.0318	0.0319
Fam20c	0.0000	-1.5418	0.0106
Fam214a	0.0000	-1.2416	0.0190
Fam214b	0.0000	-1.1231	0.0164
Fam219a	0.0000	-1.7991	0.0051
Fam227a	0.0000	-1.5431	0.0357
Fam57b	0.0000	-2.9198	0.0030
Fam72a	0.0000	1.4514	0.0333
Fam78a	0.0000	2.6263	0.0192
Fam78b	0.0000	-2.8321	0.0044
Fam81a	0.0000	2.5354	0.0056
Fam83d	0.0000	1.3328	0.0161
Fam83g	0.0000	2.0807	0.0260
Fam89b	0.0000	-1.9199	0.0133
Fanca	0.0000	1.6858	0.0115
Fancd2	0.0000	1.6042	0.0110
Fanci	0.0000	1.8722	0.0084
Fancl	0.0000	1.2521	0.0185
Farp2	0.0000	-1.1892	0.0450
Farsb	0.0000	1.2892	0.0080
Fas	0.0000	1.4584	0.0446
Fastkd2	0.0000	1.1304	0.0163
Fat3	0.0000	-2.4300	0.0017
Fat4	0.0000	-3.1237	0.0009
Faxc	0.0000	-2.6992	0.0010
Fbf1	0.0000	-1.0127	0.0170
Fbll1	0.0000	-2.0119	0.0200
Fbln2	0.0000	1.5920	0.0159
Fbln7	0.0000	2.0683	0.0393
Fbxl16	0.0000	-1.5492	0.0089

Fbxl17	0.0000	-1.0236	0.0223
Fbxl7	0.0000	-1.1096	0.0309
Fbxo16	0.0000	-1.1407	0.0313
Fbxo17	0.0002	1.4686	0.0494
Fbxo2	0.0000	1.1469	0.0357
Fbxo21	0.0000	-1.1316	0.0077
Fbxo27	0.0000	3.2549	0.0137
Fbxo32	0.0000	-1.8805	0.0143
Fbxo41	0.0000	-1.9425	0.0166
Fbxo44	0.0000	-1.0490	0.0407
Fbxo5	0.0000	1.5403	0.0084
Fbxo6	0.0000	1.6178	0.0151
Fcgrt	0.0000	1.3236	0.0250
Fen1	0.0000	1.0501	0.0176
Fermt3	0.0000	1.7528	0.0456
Fes	0.0001	2.1761	0.0478
Fez1	0.0000	-1.9495	0.0017
Fgd3	0.0000	-1.4814	0.0298
Fgd4	0.0000	-1.1375	0.0176
Fgf1	0.0000	3.3854	0.0084
Fgf12	0.0000	-3.2551	0.0084
Fgf13	0.0000	-1.8987	0.0088
Fgf14	0.0000	-1.3291	0.0377
Fgf17	0.0000	5.0891	0.0129
Fgf18	0.0000	-1.7574	0.0442
Fgf7	0.0000	3.5160	0.0163
Fgf9	0.0000	-2.1327	0.0378
Fgfr3	0.0000	-2.7961	0.0019
Fgfr4	0.0000	1.6458	0.0427
Fgl2	0.0000	1.2847	0.0337
Fhdc1	0.0000	-1.4594	0.0299
Fhod1	0.0000	1.2097	0.0206
Fibcd1	0.0000	-2.4187	0.0133
Fign	0.0000	-1.8967	0.0076
Fignl1	0.0000	1.4688	0.0117
Filip1	0.0000	-2.6901	0.0092
Fkbp4	0.0000	1.0298	0.0105
Fkbp9	0.0000	1.3258	0.0060
Flrt1	0.0000	-1.9259	0.0225
Flrt2	0.0000	-1.9161	0.0100
Flt1	0.0000	-1.4593	0.0348
Flt4	0.0000	2.2751	0.0272
Flvcr2	0.0000	1.3999	0.0339
Flywch1	0.0000	-1.5225	0.0052
Fmn1	0.0000	2.2454	0.0266
Fmn2	0.0000	-2.0265	0.0050
Fmnl1	0.0000	2.3251	0.0245
Fmnl2	0.0000	-1.1510	0.0114
Fnbp1l	0.0000	-1.3645	0.0042
Fndc5	0.0000	-2.4008	0.0153
Folr1	0.0000	3.4541	0.0040
Foxc2	0.0000	-1.1742	0.0379

Foxh1	0.0000	4.9125	0.0020
Foxj1	0.0000	1.5973	0.0110
Foxk1	0.0000	-1.0213	0.0137
Foxl2	0.0000	-1.9127	0.0299
Fpgs	0.0000	1.3725	0.0145
Fras1	0.0000	-1.3151	0.0185
Frat1	0.0000	-1.8875	0.0219
Frk	0.0000	3.1612	0.0208
Frmd3	0.0001	-1.4530	0.0480
Frmd4a	0.0000	-1.7710	0.0025
Frmd4b	0.0000	-2.3456	0.0012
Frmd5	0.0000	-1.6061	0.0094
Frmpd3	0.0000	-2.9018	0.0073
Frrs1	0.0000	4.7283	0.0004
Frrs1l	0.0000	-2.0334	0.0162
Fry	0.0000	-1.3788	0.0172
Frzb	0.0000	-2.5122	0.0074
Fsd1	0.0000	-1.2919	0.0173
Fsd1l	0.0000	-1.4978	0.0074
Fstl4	0.0000	-2.5187	0.0309
Fstl5	0.0000	-4.5232	0.0040
Fth1	0.0000	1.9347	0.0014
Ftsj3	0.0000	1.1601	0.0112
Fxr1	0.0000	1.0872	0.0061
Fxyd2	0.0000	4.3864	0.0390
Fyn	0.0000	-1.5067	0.0048
Fzd3	0.0000	-2.1199	0.0015
Fzd5	0.0000	3.8080	0.0007
G0s2	0.0000	3.1948	0.0062
Gab1	0.0000	1.2402	0.0085
Gab2	0.0000	-1.9748	0.0043
Gabbr1	0.0000	-1.9904	0.0024
Gabbr2	0.0000	-1.4144	0.0344
Gabra1	0.0000	-1.7141	0.0452
Gabra2	0.0000	-2.3195	0.0212
Gabra3	0.0000	-1.5571	0.0325
Gabrb2	0.0000	-3.4117	0.0069
Gabrb3	0.0000	-1.5818	0.0115
Gabrg1	0.0000	-2.5824	0.0391
Gabrg2	0.0000	-1.6386	0.0235
Gad1	0.0000	-1.3964	0.0297
Gadd45a	0.0000	1.5632	0.0099
Gadd45b	0.0000	1.3818	0.0191
Gadd45g	0.0000	-2.5245	0.0030
Gadd45gip1	0.0000	1.1257	0.0154
Galm	0.0000	2.6019	0.0085
Galnt12	0.0000	1.3724	0.0343
Galnt13	0.0000	-2.0862	0.0207
Galnt14	0.0000	-2.9572	0.0222
Galnt16	0.0000	-1.0519	0.0165
Galnt18	0.0000	-2.4749	0.0129
Galnt3	0.0000	1.7847	0.0425

Galnt6	0.0000	3.4842	0.0094
Galnt7	0.0000	1.1053	0.0133
Galt	0.0000	1.6946	0.0196
Gap43	0.0000	-2.3979	0.0005
Gart	0.0000	1.4878	0.0028
Gas6	0.0000	1.7918	0.0086
Gata2	0.0000	-1.9030	0.0200
Gata3	0.0000	-2.1383	0.0125
Gatad2a	0.0000	1.0273	0.0086
Gatm	0.0000	2.0778	0.0307
Gba2	0.0000	-1.3028	0.0121
Gbp2	0.0000	1.1729	0.0466
Gbp3	0.0000	3.3190	0.0332
Gca	0.0000	1.4622	0.0249
Gcat	0.0000	1.6844	0.0070
Gcdh	0.0000	1.3097	0.0180
Gclm	0.0000	1.6807	0.0072
Gcnt1	0.0000	1.4789	0.0296
Gcnt2	0.0000	1.6538	0.0109
Gdap1	0.0000	-2.2516	0.0040
Gdap1l1	0.0000	-2.7936	0.0025
Gdf11	0.0000	-1.3759	0.0092
Gdi1	0.0000	-1.3128	0.0048
Gdpd1	0.0000	-1.3766	0.0087
Gdpd5	0.0000	-2.1610	0.0043
Gem	0.0000	1.6112	0.0336
Gfap	0.0000	8.1019	0.0098
Gfod2	0.0000	-1.8884	0.0051
Gfpt2	0.0000	2.7802	0.0038
Gfra1	0.0000	-2.0432	0.0025
Gga2	0.0000	1.0443	0.0083
Gipc1	0.0000	1.0247	0.0123
Gipc3	0.0001	2.9532	0.0488
Gipr	0.0000	-1.1167	0.0441
Gjb5	0.0000	4.2314	0.0041
Gjb6	0.0000	1.6669	0.0448
Gjd2	0.0000	-2.0113	0.0239
Gk	0.0000	1.5024	0.0160
Glb1	0.0000	1.1316	0.0190
Glcci1	0.0000	-1.8547	0.0393
Gli2	0.0000	1.1674	0.0124
Gli3	0.0000	-1.2450	0.0112
Glis3	0.0000	1.0317	0.0306
Glra1	0.0000	-4.7368	0.0176
Glra2	0.0000	-3.3027	0.0182
Glrx	0.0000	1.5120	0.0172
Gls2	0.0000	2.9675	0.0089
Glt1d1	0.0000	2.0092	0.0123
Gmip	0.0000	-1.1672	0.0337
Gmnn	0.0000	1.2610	0.0165
Gna14	0.0002	1.9084	0.0496
Gna15	0.0000	5.6818	0.0119

Gnal	0.0000	-1.9741	0.0080
Gnaq	0.0000	-1.5094	0.0046
Gnaz	0.0000	-1.2812	0.0215
Gnb5	0.0000	-1.5178	0.0113
Gng2	0.0000	-1.7710	0.0020
Gng7	0.0000	-1.5964	0.0228
Gnl3	0.0000	1.7890	0.0023
Gnpda1	0.0000	2.5934	0.0011
Gnpnat1	0.0000	1.7014	0.0074
Golga1	0.0000	-1.4793	0.0134
Golga7b	0.0000	-2.6994	0.0106
Got1	0.0000	1.8866	0.0025
Gpat2	0.0000	4.1714	0.0030
Gpc2	0.0000	-2.7006	0.0006
Gpc5	0.0002	-2.0322	0.0495
Gphn	0.0000	-1.2343	0.0134
Gpm6a	0.0000	-1.0241	0.0131
Gpm6b	0.0000	-1.2550	0.0090
Gpr137c	0.0000	-1.4279	0.0205
Gpr139	0.0000	-1.5615	0.0416
Gpr153	0.0000	-1.1965	0.0199
Gpr155	0.0000	-2.0232	0.0260
Gpr156	0.0000	-1.6941	0.0444
Gpr160	0.0000	4.0345	0.0260
Gpr161	0.0000	-1.7690	0.0055
Gpr162	0.0000	-1.8822	0.0101
Gpr173	0.0000	-2.0578	0.0070
Gpr22	0.0000	-3.2985	0.0087
Gpr37l1	0.0000	4.7490	0.0396
Gpr61	0.0001	-1.6256	0.0477
Gpr68	0.0001	2.5477	0.0482
Gpr83	0.0002	2.3280	0.0494
Gprasp1	0.0000	-1.5873	0.0036
Gprc5a	0.0000	2.5261	0.0122
Gprin1	0.0000	-2.9390	0.0014
Gpsm1	0.0000	-1.8401	0.0031
Gpsm3	0.0000	2.4568	0.0345
Gpt2	0.0000	1.2267	0.0076
Gpx1	0.0000	1.5413	0.0027
Gpx3	0.0000	1.2282	0.0181
Gpx4	0.0000	1.7226	0.0042
Gpx7	0.0000	3.2923	0.0055
Gramd1a	0.0000	-1.0322	0.0180
Gramd1c	0.0000	1.9555	0.0277
Grasp	0.0000	1.2175	0.0249
Grb10	0.0000	-2.9398	0.0005
Grb7	0.0000	3.5788	0.0075
Greb1	0.0000	-1.6766	0.0263
Greb1l	0.0000	-1.1144	0.0324
Grem2	0.0000	-2.3883	0.0112
Grhl1	0.0000	1.9127	0.0408
Grhl3	0.0000	3.2752	0.0066

Gria1	0.0000	-2.4998	0.0135
Gria2	0.0000	-2.3442	0.0071
Gria4	0.0000	-1.2534	0.0235
Grid1	0.0000	-1.2908	0.0281
Grid2	0.0000	-1.7866	0.0314
Grik1	0.0000	-2.5622	0.0288
Grik2	0.0000	-1.9967	0.0202
Grik3	0.0000	-1.6963	0.0035
Grik4	0.0000	-1.6316	0.0273
Grik5	0.0000	-1.4795	0.0082
Grin2a	0.0000	-1.4408	0.0435
Grin2b	0.0000	-2.5220	0.0111
Grin2c	0.0000	1.7537	0.0412
Grin3a	0.0000	-3.0036	0.0139
Grip1	0.0000	-1.0815	0.0156
Grip2	0.0000	-1.6838	0.0370
Grk5	0.0000	-1.4534	0.0165
Grm2	0.0000	-3.4767	0.0181
Grm3	0.0001	-1.7326	0.0472
Grn	0.0000	1.5013	0.0052
Grp	0.0000	-3.7587	0.0453
Grtp1	0.0000	2.3104	0.0078
Gsap	0.0001	2.2635	0.0488
Gsdmd	0.0000	4.4282	0.0184
Gsg1l	0.0000	-3.2740	0.0053
Gsk3b	0.0000	-1.8631	0.0015
Gsr	0.0000	2.4861	0.0009
Gss	0.0000	1.7580	0.0084
Gsta4	0.0000	2.1675	0.0014
Gstm1	0.0000	2.2364	0.0035
Gstm2	0.0000	2.3301	0.0102
Gstm3	0.0000	4.3130	0.0207
Gstm4	0.0000	2.2630	0.0138
Gstp1	0.0000	2.1692	0.0018
Gstt1	0.0000	3.0021	0.0286
Gstt2	0.0000	3.7771	0.0074
Gsx1	0.0000	-2.0029	0.0326
Gsx2	0.0000	2.1045	0.0423
Gtf2ird2	0.0000	-1.3621	0.0266
Gusb	0.0000	1.4347	0.0135
Gypc	0.0000	1.6316	0.0320
H2ac18	0.0000	1.4446	0.0291
Hacd2	0.0000	1.4107	0.0141
Hapln1	0.0000	-2.0576	0.0252
Hapln4	0.0000	1.5828	0.0418
Haspin	0.0000	1.4346	0.0193
Hat1	0.0000	1.7863	0.0021
Haus4	0.0000	1.7382	0.0132
Hck	0.0000	3.4406	0.0046
Hcn3	0.0000	-2.0667	0.0149
Hcn4	0.0000	-1.8793	0.0085
Hdac9	0.0000	-1.1470	0.0257

Hddc3	0.0000	-1.2318	0.0208
Hdgfl3	0.0000	-1.6877	0.0030
Hdhd3	0.0000	-1.1380	0.0400
Hdx	0.0000	-1.2224	0.0297
Hebp1	0.0000	1.5358	0.0094
Hebp2	0.0002	1.3497	0.0498
Hectd4	0.0000	-2.1432	0.0023
Hecw1	0.0000	-2.1518	0.0157
Heg1	0.0000	-1.5186	0.0097
Helb	0.0000	1.7716	0.0097
Hells	0.0000	1.6930	0.0040
Helz2	0.0000	2.3691	0.0242
Hepacam	0.0000	4.1276	0.0098
Herc1	0.0000	-1.6193	0.0026
Herc2	0.0000	-1.3498	0.0044
Herpud1	0.0000	1.1941	0.0125
Hes1	0.0000	1.1989	0.0121
Hexa	0.0000	1.7595	0.0024
Hexb	0.0000	2.4700	0.0011
Hey2	0.0000	-2.4352	0.0229
Heyl	0.0001	-1.0865	0.0482
Hhat	0.0000	2.1681	0.0294
Hhex	0.0000	3.8008	0.0162
Hhipl1	0.0000	-1.3967	0.0282
Hid1	0.0000	-1.1646	0.0239
Hip1	0.0000	-1.4526	0.0045
Hipk2	0.0000	-1.9317	0.0031
Hirip3	0.0000	1.2287	0.0160
Hivep1	0.0000	-1.1933	0.0110
Hivep2	0.0000	-1.1978	0.0182
Hivep3	0.0000	-1.8001	0.0421
Hjurp	0.0000	1.0126	0.0149
Hmces	0.0000	1.3649	0.0144
Hmcn2	0.0000	3.1253	0.0243
Hmga1	0.0000	1.2750	0.0087
Hmga2	0.0000	-1.7008	0.0077
Hmgb2	0.0000	1.8964	0.0034
Hmgb3	0.0000	-1.7153	0.0020
Hmgcr	0.0000	-1.1250	0.0063
Hmgcs1	0.0000	-1.4901	0.0017
Hmox1	0.0000	3.0047	0.0014
Hook1	0.0000	1.3545	0.0079
Hook2	0.0000	1.4438	0.0172
Hopx	0.0000	-1.2419	0.0410
Hormad1	0.0000	4.3367	0.0148
Hp1bp3	0.0000	-1.5055	0.0025
Hpca	0.0000	-2.4703	0.0120
Hpcal4	0.0000	-1.3235	0.0420
Hpgd	0.0000	1.3556	0.0462
Hpgds	0.0000	-2.2115	0.0417
Hpn	0.0000	2.3132	0.0442
Hr	0.0000	1.7834	0.0465

Hrc	0.0000	1.1444	0.0350
Hs1bp3	0.0000	1.3378	0.0103
Hs3st1	0.0000	-2.1516	0.0317
Hs3st2	0.0000	-2.9941	0.0266
Hs3st3a1	0.0000	-2.3048	0.0305
Hs3st3b1	0.0000	-1.0930	0.0295
Hs3st5	0.0000	-3.6262	0.0159
Hs6st3	0.0000	-2.3941	0.0459
Hscb	0.0000	1.1330	0.0286
Hsd17b11	0.0000	2.2633	0.0048
Hsd17b14	0.0000	5.2922	0.0071
Hsd3b7	0.0000	1.6249	0.0281
Hsdl2	0.0000	-1.0789	0.0174
Hsp90aa1	0.0000	2.0758	0.0002
Hspa12a	0.0000	-2.2980	0.0016
Hspa14	0.0000	1.0907	0.0101
Hspa1a	0.0000	1.6490	0.0278
Hspa1b	0.0000	2.4715	0.0077
Hspa1l	0.0000	-1.6378	0.0451
Hspa4l	0.0000	-1.3048	0.0101
Hspa9	0.0000	1.4134	0.0031
Hspb1	0.0000	3.0354	0.0008
Hspb2	0.0000	2.5739	0.0351
Hspb6	0.0000	1.3799	0.0334
Hspb8	0.0000	2.9162	0.0013
Hspbap1	0.0000	1.9906	0.0074
Hspd1	0.0000	1.2798	0.0033
Hspe1	0.0000	1.8458	0.0012
Hvcn1	0.0000	1.4324	0.0261
Hydin	0.0000	3.7089	0.0154
Iars	0.0000	1.1847	0.0055
Icam1	0.0000	3.4210	0.0012
Id2	0.0000	-1.3086	0.0089
Id4	0.0000	-1.8625	0.0034
Ide	0.0000	1.0561	0.0099
Idh2	0.0000	1.2007	0.0059
Idnk	0.0000	-1.0585	0.0338
Ids	0.0000	-1.6381	0.0067
Ier2	0.0000	1.0774	0.0145
Ifi30	0.0000	3.4193	0.0009
Ifi35	0.0000	2.1080	0.0305
Ifi44	0.0000	2.9148	0.0317
Ifih1	0.0000	1.7513	0.0450
Ifit1	0.0000	3.8093	0.0267
Ifit3	0.0000	2.4894	0.0278
Ifitm1	0.0000	3.8895	0.0005
Ifitm2	0.0000	2.5715	0.0007
Ifitm3	0.0000	4.2755	0.0006
Ifnlr1	0.0000	1.9461	0.0403
Igdcc3	0.0002	-1.7223	0.0491
Igf1r	0.0000	-1.4249	0.0043
Igf2r	0.0000	-1.3551	0.0069

Igfbp2	0.0000	1.1842	0.0093
Igfbp5	0.0000	-1.0049	0.0367
Igfbp6	0.0000	3.0569	0.0253
Igip	0.0003	1.0032	0.0500
Iglon5	0.0000	-2.6108	0.0024
Igsf10	0.0000	1.5524	0.0149
Igsf11	0.0000	-2.3409	0.0097
Igsf21	0.0000	-1.5092	0.0188
Igsf23	0.0000	3.0163	0.0346
Il17rc	0.0000	2.8374	0.0243
Il1r1	0.0000	3.4174	0.0187
Il1rapl2	0.0000	-4.5203	0.0371
Il1rl2	0.0000	3.8145	0.0342
Il23a	0.0000	3.7647	0.0354
Il27ra	0.0001	5.1012	0.0469
Il33	0.0000	3.4237	0.0284
Il34	0.0000	2.4502	0.0411
Il3ra	0.0000	2.1676	0.0220
Il4i1	0.0000	3.2490	0.0412
Immp2l	0.0001	1.0970	0.0478
Impa2	0.0000	3.1960	0.0010
Impact	0.0000	1.8647	0.0015
Ina	0.0000	-3.1583	0.0002
Inafm2	0.0000	-1.2283	0.0173
Inf2	0.0000	1.5601	0.0165
Ing5	0.0000	1.9178	0.0042
Inpp4a	0.0000	-1.3803	0.0096
Inpp5d	0.0000	3.8552	0.0024
Inpp5f	0.0000	-1.5835	0.0046
Inpp5j	0.0000	-2.1473	0.0464
Insm2	0.0000	-4.9664	0.0387
Ip6k2	0.0000	-1.0108	0.0133
Ipcef1	0.0002	2.5941	0.0491
Iqcg	0.0000	1.0032	0.0285
Iqgap1	0.0000	1.8850	0.0014
Iqgap3	0.0000	1.0166	0.0249
Iqsec3	0.0000	-2.7497	0.0042
Iqub	0.0000	3.6152	0.0286
Irak2	0.0000	1.2044	0.0339
Irak3	0.0000	3.2823	0.0100
Irf1	0.0000	1.8957	0.0124
Irf6	0.0000	3.7104	0.0080
Irf7	0.0000	3.0552	0.0376
Irgq	0.0000	-1.5700	0.0064
Isg15	0.0000	4.2267	0.0164
Islr2	0.0000	-1.9073	0.0028
Ism2	0.0000	3.9198	0.0106
Itga5	0.0000	1.4062	0.0087
Itga6	0.0000	1.3870	0.0066
Itga8	0.0000	1.1721	0.0236
Itgae	0.0000	3.6236	0.0426
Itgal	0.0000	3.0308	0.0448

Itgam	0.0000	3.3833	0.0283
Itgb3	0.0000	1.0908	0.0376
Itgb4	0.0000	1.5983	0.0358
Itgb5	0.0000	1.1280	0.0162
Itgb7	0.0000	4.2505	0.0201
Itgb8	0.0000	-1.9475	0.0058
Itpk1	0.0000	1.1625	0.0145
Itpka	0.0000	3.1510	0.0134
Itprip	0.0000	-1.5258	0.0157
Itsn1	0.0000	-1.4752	0.0035
Itsn2	0.0000	1.0415	0.0186
Jade1	0.0000	2.5682	0.0006
Jade2	0.0000	3.2110	0.0058
Jade3	0.0000	1.7487	0.0039
Jag1	0.0000	-1.0799	0.0172
Jak3	0.0000	2.4463	0.0049
Jakmip1	0.0000	2.4042	0.0079
Jakmip2	0.0000	-2.7637	0.0017
Jazf1	0.0000	-1.6106	0.0268
Jdp2	0.0000	1.5271	0.0142
Jph1	0.0000	2.4217	0.0111
Jph3	0.0000	-1.1972	0.0196
Jph4	0.0000	-1.0817	0.0283
Jun	0.0000	-1.3860	0.0061
Junb	0.0000	1.6091	0.0200
Kalrn	0.0000	-2.1135	0.0024
Kank2	0.0000	1.8901	0.0032
Kank3	0.0000	2.7468	0.0033
Kank4	0.0000	2.0626	0.0350
Kat2b	0.0000	1.3696	0.0093
Katnal1	0.0000	-1.2003	0.0132
Kcmf1	0.0000	1.0050	0.0131
Kcna1	0.0000	-6.8945	0.0313
Kcna5	0.0000	-2.2387	0.0201
Kcnab2	0.0000	-1.6906	0.0170
Kcnb1	0.0000	-1.6521	0.0147
Kcnb2	0.0000	-2.1243	0.0246
Kcnc1	0.0000	-2.6785	0.0045
Kcnd2	0.0000	-3.2094	0.0206
Kcnd3	0.0000	-2.0318	0.0071
Kcne4	0.0000	1.1489	0.0450
Kcnf1	0.0000	-1.5332	0.0411
Kcnh2	0.0000	-1.4713	0.0166
Kcnh3	0.0000	3.3507	0.0201
Kcnh4	0.0000	-2.2098	0.0315
Kcnh7	0.0000	-1.9900	0.0274
Kcnh8	0.0000	-3.0707	0.0266
Kcnip1	0.0000	-1.7396	0.0297
Kcnip2	0.0000	1.8305	0.0292
Kcnip4	0.0000	-3.3945	0.0208
Kcnj10	0.0000	2.5060	0.0236
Kcnj11	0.0000	-2.3548	0.0296

Kcnj16	0.0000	3.6497	0.0385
Kcnj4	0.0000	2.3158	0.0218
Kcnj5	0.0000	-3.3409	0.0439
Kcnk1	0.0000	1.9893	0.0132
Kcnk10	0.0000	-2.8238	0.0086
Kcnk2	0.0000	-1.4182	0.0200
Kcnk3	0.0000	-1.9584	0.0306
Kcnk9	0.0000	-1.6650	0.0243
Kcnma1	0.0000	-2.0617	0.0174
Kcnmb2	0.0000	-2.3941	0.0276
Kcnmb4	0.0000	-1.8628	0.0215
Kcnn1	0.0000	-1.1242	0.0212
Kcnn3	0.0000	-1.3261	0.0281
Kcnq2	0.0000	-2.3180	0.0027
Kcnq3	0.0000	-2.3516	0.0102
Kcns3	0.0000	2.6996	0.0192
Kctd1	0.0000	-1.4324	0.0167
Kctd21	0.0000	-1.1339	0.0371
Kctd4	0.0000	2.2799	0.0325
Kctd6	0.0000	1.5354	0.0166
Kctd7	0.0000	-1.1566	0.0299
Kctd8	0.0000	-4.0830	0.0225
Kdelr3	0.0000	1.3257	0.0163
Kdf1	0.0000	2.9227	0.0294
Kdm1b	0.0000	-1.1362	0.0180
Kdm4b	0.0000	-1.2532	0.0075
Kdm6a	0.0000	1.0319	0.0130
Kdsr	0.0000	1.3263	0.0136
Khdrbs1	0.0000	-1.1110	0.0065
Khdrbs2	0.0000	-2.3457	0.0183
Khdrbs3	0.0000	-1.2210	0.0101
Kidins220	0.0000	-1.8166	0.0020
Kif14	0.0000	1.2790	0.0143
Kif18b	0.0000	1.5103	0.0131
Kif1a	0.0000	-1.2655	0.0046
Kif1b	0.0000	-2.0489	0.0005
Kif20a	0.0000	1.2881	0.0089
Kif21a	0.0000	-1.9891	0.0038
Kif21b	0.0000	-1.7295	0.0020
Kif22	0.0000	1.1355	0.0193
Kif26b	0.0000	-1.9829	0.0066
Kif2c	0.0000	1.2884	0.0154
Kif3a	0.0000	-1.4336	0.0080
Kif3c	0.0000	-1.5426	0.0033
Kif5a	0.0000	-2.1470	0.0020
Kif5c	0.0000	-2.8957	0.0002
Kifc1	0.0000	1.2775	0.0100
Kirrel3	0.0000	-2.5154	0.0081
Klc1	0.0000	-1.8808	0.0017
Klc3	0.0000	2.3857	0.0295
Klf15	0.0000	2.9387	0.0057
Klf2	0.0000	5.1046	0.0001

Klf4	0.0000	2.9969	0.0023
Klf5	0.0000	2.5402	0.0159
Klf7	0.0000	-1.1937	0.0123
Klf8	0.0000	1.4854	0.0217
Klf9	0.0000	3.6339	0.0003
Klhdc8b	0.0000	-1.0810	0.0176
Klhl1	0.0000	-3.8603	0.0140
Klhl14	0.0000	-3.6326	0.0146
Klhl23	0.0000	-1.4468	0.0097
Klhl29	0.0000	-1.5471	0.0155
Klhl3	0.0000	-2.1776	0.0127
Klhl32	0.0000	-1.9674	0.0166
Klhl5	0.0000	-1.0609	0.0200
Klhl7	0.0000	-1.1711	0.0083
Kmt2a	0.0000	-1.3633	0.0062
Kmt2e	0.0000	-1.7084	0.0034
Kndc1	0.0000	-1.0997	0.0306
Krt18	0.0000	1.2248	0.0440
Ksr2	0.0000	-2.9395	0.0053
Kxd1	0.0000	1.2873	0.0120
L1cam	0.0000	-2.8213	0.0008
L2hgdh	0.0000	1.4757	0.0106
L3mbtl3	0.0000	-1.3212	0.0139
Lactb2	0.0000	3.0476	0.0003
Lair1	0.0001	-2.5975	0.0486
Lama1	0.0000	-1.8338	0.0049
Lama3	0.0000	3.1407	0.0184
Lama4	0.0000	2.7123	0.0024
Lama5	0.0000	1.3697	0.0119
Lamc3	0.0000	-2.2834	0.0214
Lamp1	0.0000	1.7241	0.0018
Lamp2	0.0000	1.4637	0.0043
Lamp5	0.0000	-2.9121	0.0073
Lap3	0.0000	1.9779	0.0024
Laptm5	0.0000	5.4627	0.0012
Larp1b	0.0000	1.1194	0.0310
Layn	0.0000	2.2903	0.0420
Lbh	0.0000	-1.9141	0.0016
Lbr	0.0000	1.0188	0.0151
Lbx1	0.0000	-3.6666	0.0186
Lcor	0.0000	-1.9752	0.0042
Ldb3	0.0000	1.9386	0.0324
Ldhd	0.0000	5.7876	0.0268
Ldlrap1	0.0000	1.0273	0.0310
Lefty1	0.0000	4.3768	0.0002
Letmd1	0.0000	-1.3488	0.0177
Lfng	0.0000	-1.2131	0.0226
Lgals3	0.0000	3.3651	0.0067
Lgals3bp	0.0000	2.9246	0.0088
Lgals9	0.0000	1.6299	0.0230
Lgi1	0.0000	2.4586	0.0205
Lgi3	0.0001	1.0055	0.0478

Lgr4	0.0000	-1.1307	0.0102
Lgr5	0.0000	-1.3646	0.0407
Lhfpl3	0.0000	-2.3060	0.0371
Lhfpl4	0.0000	-2.1838	0.0035
Lhx9	0.0001	1.2838	0.0484
Lif	0.0000	1.6105	0.0309
Lig1	0.0000	1.0000	0.0126
Lima1	0.0000	2.4163	0.0006
Limch1	0.0000	-1.4853	0.0092
Limk1	0.0000	-1.3880	0.0085
Lin7a	0.0000	-4.5719	0.0026
Lingo2	0.0000	-2.5839	0.0274
Lipc	0.0000	5.7420	0.0326
Liph	0.0000	2.4249	0.0204
Llgl2	0.0000	2.7678	0.0028
Lmo1	0.0000	-1.9276	0.0177
Lmo3	0.0000	-1.8569	0.0195
Lmo4	0.0000	-1.0048	0.0176
Lmo7	0.0000	2.7669	0.0047
Lnx1	0.0000	2.2148	0.0362
Lonrf2	0.0000	-1.5445	0.0100
Lonrf3	0.0000	1.6095	0.0262
Lpar5	0.0000	3.9482	0.0437
Lpar6	0.0000	-1.1662	0.0246
Lpin3	0.0000	2.9047	0.0198
Lpl	0.0000	2.4557	0.0034
Lrch2	0.0000	-1.5745	0.0062
Lrfn2	0.0000	-1.5100	0.0426
Lrfn3	0.0000	-1.9284	0.0145
Lrfn5	0.0000	-2.0229	0.0300
Lrmp	0.0000	5.0107	0.0121
Lrp11	0.0000	1.3449	0.0109
Lrp12	0.0000	-1.7044	0.0051
Lrp3	0.0000	-1.7578	0.0063
Lrp5	0.0000	1.0562	0.0144
Lrp8	0.0000	-1.4497	0.0072
Lrrc10b	0.0000	1.2810	0.0448
Lrrc17	0.0000	3.1748	0.0113
Lrrc2	0.0000	5.6681	0.0047
Lrrc20	0.0000	-1.7015	0.0109
Lrrc34	0.0000	5.0350	0.0025
Lrrc4	0.0000	-1.8419	0.0128
Lrrc49	0.0000	-1.8654	0.0061
Lrrc4b	0.0000	-1.8733	0.0033
Lrrc4c	0.0000	-3.3463	0.0132
Lrrc7	0.0000	-2.6364	0.0454
Lrrc73	0.0000	-1.4994	0.0454
Lrrc75b	0.0000	-1.8242	0.0164
Lrrn1	0.0000	-2.7227	0.0008
Lrrn3	0.0000	-2.1117	0.0045
Lrrn4	0.0000	3.0065	0.0226
Lrrtm2	0.0000	-2.2568	0.0141

Lrrtm3	0.0000	-3.3650	0.0084
Lrsam1	0.0000	-1.1488	0.0248
Lsamp	0.0000	-1.8455	0.0059
Lsm11	0.0000	-1.1328	0.0152
Lsm14b	0.0000	-1.0663	0.0104
Lsm4	0.0000	1.2274	0.0137
Lsr	0.0000	1.3423	0.0186
Lss	0.0000	-1.0902	0.0093
Ltb	0.0000	5.8026	0.0396
Ltbp4	0.0000	1.7524	0.0263
Ltbr	0.0000	1.3150	0.0317
Luzp2	0.0000	1.5182	0.0246
Ly6e	0.0000	1.0517	0.0224
Ly6h	0.0000	-2.1345	0.0029
Ly75	0.0000	4.3514	0.0002
Ly96	0.0001	2.2179	0.0485
Lyn	0.0000	2.0047	0.0225
Lynx1	0.0000	-1.4746	0.0462
Lypla1	0.0000	1.7181	0.0038
Lysmd1	0.0000	-1.5892	0.0161
Lztr1	0.0000	-1.0334	0.0147
Lzts1	0.0000	-2.3966	0.0047
M1ap	0.0000	4.6185	0.0156
Macrod2	0.0000	-1.2227	0.0241
Madcam1	0.0000	5.4848	0.0308
Madd	0.0000	-2.1834	0.0026
Mael	0.0000	4.4540	0.0138
Maf	0.0000	-1.0039	0.0258
Magee1	0.0000	-2.2584	0.0021
Magef1	0.0000	2.2700	0.0215
Mak	0.0000	1.6046	0.0453
Mak16	0.0000	1.1639	0.0091
Mall	0.0000	4.8613	0.0308
Malt1	0.0000	1.6521	0.0277
Mamdc2	0.0000	2.9568	0.0292
Maml3	0.0000	-1.7449	0.0061
Mamld1	0.0000	-1.3364	0.0387
Man2a2	0.0000	-1.2512	0.0106
Man2b1	0.0000	1.5790	0.0077
Manba	0.0000	2.7809	0.0022
Mansc1	0.0000	2.8417	0.0375
Maob	0.0000	3.7909	0.0290
Map10	0.0000	2.4264	0.0080
Map1a	0.0000	-1.3558	0.0065
Map1b	0.0000	-2.3211	0.0002
Map2	0.0000	-2.1868	0.0004
Map2k3	0.0000	1.0635	0.0219
Map3k10	0.0000	-1.2445	0.0120
Map3k13	0.0000	-2.3150	0.0063
Map3k15	0.0000	2.6613	0.0103
Map3k19	0.0000	4.6271	0.0278
Map3k5	0.0000	1.6085	0.0272

Map7d3	0.0000	2.7735	0.0137
Mapk10	0.0000	-2.3881	0.0019
Mapk11	0.0000	-1.9560	0.0102
Mapk13	0.0000	2.6841	0.0286
Mapk8	0.0000	-1.4502	0.0055
Mapk8ip1	0.0000	-1.7446	0.0023
Mapk8ip2	0.0000	-2.0747	0.0021
Mapkapk3	0.0000	1.9151	0.0072
Mapkbp1	0.0000	-1.2048	0.0137
Mapre2	0.0000	-1.6444	0.0037
Mapt	0.0000	-2.2975	0.0022
Marcks	0.0000	-1.1444	0.0073
Marcksl1	0.0000	-1.0092	0.0064
Mark1	0.0000	-1.4310	0.0088
Marveld2	0.0000	1.7178	0.0353
Mast1	0.0000	-2.1769	0.0047
Mast4	0.0000	-1.5252	0.0107
Mastl	0.0000	1.1891	0.0230
Matn3	0.0000	2.3295	0.0301
Mavs	0.0000	1.3487	0.0169
Mb21d2	0.0000	-1.6935	0.0071
Mbd5	0.0000	-1.4366	0.0133
Mbnl2	0.0000	1.2087	0.0163
Mboat1	0.0000	2.4146	0.0198
Mboat2	0.0000	-1.1179	0.0150
Mcam	0.0000	1.4054	0.0144
Mccc1	0.0000	1.2114	0.0199
Mcf2	0.0000	4.6260	0.0013
Mcf2l	0.0000	-1.2414	0.0112
Mcm4	0.0000	1.0200	0.0125
Mcm5	0.0000	2.0626	0.0027
Mcm6	0.0000	1.6157	0.0037
Mcm7	0.0000	1.1454	0.0120
Mcmdc2	0.0000	5.2722	0.0367
Mcoln1	0.0000	1.1158	0.0163
Mcph1	0.0000	1.7921	0.0064
Mctp2	0.0000	1.8925	0.0412
Mdfic	0.0000	1.4166	0.0171
Mdga2	0.0000	-2.4023	0.0197
Me1	0.0000	1.7796	0.0090
Mecom	0.0000	-2.3031	0.0102
Mecp2	0.0000	-1.3610	0.0063
Med12l	0.0000	-1.1251	0.0237
Med26	0.0000	1.0057	0.0224
Mef2b	0.0000	2.7471	0.0151
Meg3	0.0000	-1.0424	0.0164
Megf11	0.0000	-1.5011	0.0180
Megf6	0.0000	-1.5322	0.0092
Megf9	0.0000	-1.4279	0.0056
Meiob	0.0000	4.6080	0.0279
Meis1	0.0000	-3.1924	0.0007
Meis2	0.0000	-2.7296	0.0005

Meis3	0.0000	-2.5013	0.0038
Meox1	0.0000	2.7933	0.0234
Metrn	0.0000	-1.1493	0.0149
Metrnl	0.0000	-1.0709	0.0322
Mettl5	0.0000	1.0836	0.0256
Mex3a	0.0000	-1.4417	0.0033
Mex3b	0.0000	-1.5296	0.0058
Mfge8	0.0000	1.5369	0.0068
Mfn2	0.0000	-1.1456	0.0123
Mfng	0.0000	-1.1357	0.0304
Mfsd10	0.0000	1.3316	0.0182
Mfsd13a	0.0000	1.0086	0.0324
Mfsd2a	0.0000	-3.1570	0.0099
Mgarp	0.0000	2.8212	0.0080
Mgat3	0.0000	-1.3264	0.0093
Mgat4c	0.0000	-3.8781	0.0130
Mgat5	0.0000	-1.0732	0.0122
Mgat5b	0.0000	-1.9673	0.0037
Mgll	0.0001	-1.0622	0.0487
Mgme1	0.0000	1.6114	0.0114
Mia	0.0000	4.9743	0.0125
Miat	0.0000	-2.1645	0.0359
Mib1	0.0000	-1.4722	0.0033
Mical2	0.0000	-1.1476	0.0340
Mid2	0.0000	-1.8710	0.0141
Midn	0.0000	-1.1556	0.0071
Mif4gd	0.0000	2.4095	0.0050
Mipol1	0.0000	1.3170	0.0209
Mir124-2hg	0.0000	-3.2694	0.0010
Mir17hg	0.0000	2.1435	0.0288
Mis18bp1	0.0000	1.2294	0.0133
Mitf	0.0000	1.7939	0.0222
Mks1	0.0000	1.0370	0.0343
Mlh3	0.0000	1.5465	0.0098
Mllt1	0.0000	-1.0605	0.0105
Mllt11	0.0000	-2.1575	0.0012
Mllt3	0.0000	-1.5423	0.0065
Mmab	0.0000	-1.0478	0.0218
Mmd	0.0000	-1.5793	0.0062
Mme	0.0000	3.0095	0.0021
Mmgt1	0.0000	-1.0111	0.0139
Mmp14	0.0000	1.3916	0.0121
Mmp16	0.0000	-1.3264	0.0230
Mmp19	0.0002	1.7088	0.0493
Mmp2	0.0000	1.2881	0.0124
Mmp24	0.0000	-2.3816	0.0040
Mmp9	0.0000	3.7106	0.0161
Mmrn2	0.0000	4.0303	0.0039
Mnd1	0.0000	3.1176	0.0133
Moap1	0.0000	-1.6595	0.0199
Mob3c	0.0000	1.2297	0.0418
Mobp	0.0000	3.4723	0.0186

Mov10	0.0000	3.0092	0.0005
Mpdz	0.0000	-1.5343	0.0059
Mpeg1	0.0000	-2.2278	0.0368
Mphosph6	0.0000	1.3072	0.0116
Mpp2	0.0000	-2.2868	0.0036
Mpp3	0.0000	-2.9585	0.0077
Mpped1	0.0000	-2.2580	0.0245
Mpped2	0.0000	-1.5792	0.0214
Mpzl2	0.0000	2.5247	0.0060
Mr1	0.0000	2.3504	0.0167
Mras	0.0000	-1.4556	0.0115
Mreg	0.0000	3.7418	0.0003
Mrgbp	0.0000	-1.0908	0.0217
Mri1	0.0000	1.4739	0.0163
Mrpl15	0.0000	1.7983	0.0028
Mrpl17	0.0000	1.1472	0.0124
Mrpl35	0.0000	1.0884	0.0239
Mrps28	0.0000	1.4101	0.0175
Mrps31	0.0000	1.3675	0.0096
Mrps9	0.0000	1.0366	0.0129
Msc	0.0000	2.7736	0.0214
Msh2	0.0000	1.1466	0.0074
Msln	0.0000	4.2026	0.0340
Msrb2	0.0000	1.6313	0.0231
Msto1	0.0000	1.0518	0.0174
Mt3	0.0000	1.9738	0.0155
Mta3	0.0000	1.9193	0.0027
Mtfp1	0.0000	1.7191	0.0287
Mthfd1	0.0000	2.2220	0.0016
Mthfd1l	0.0000	1.7197	0.0045
Mthfd2	0.0000	2.5449	0.0008
Mthfs	0.0001	1.2502	0.0470
Mtmr1	0.0000	-1.2316	0.0191
Mtmr12	0.0000	1.0242	0.0213
Mtmr4	0.0000	-1.6442	0.0034
Mtmr6	0.0000	-1.0795	0.0133
Mtrr	0.0000	1.1807	0.0174
Mturn	0.0000	-2.0316	0.0021
Mtus2	0.0000	-2.1878	0.0057
Mtx3	0.0000	-1.2387	0.0202
Mutyh	0.0000	1.3417	0.0319
Mvb12a	0.0000	1.1097	0.0190
Mvb12b	0.0000	-1.5102	0.0053
Mvp	0.0000	1.5829	0.0164
Mxd1	0.0000	-1.5915	0.0182
Mxra7	0.0000	-1.6324	0.0081
Myb	0.0000	2.7698	0.0083
Mybbp1a	0.0000	1.1839	0.0071
Mybl2	0.0000	4.1161	0.0001
Mybpc2	0.0000	4.8675	0.0301
Mycbp2	0.0000	-1.3615	0.0046
Mycl	0.0000	-1.9640	0.0032

Myh13	0.0000	3.6202	0.0131
Myh14	0.0000	2.1549	0.0156
Myh7b	0.0000	2.5607	0.0146
Myh9	0.0000	1.0581	0.0104
Myl6b	0.0000	-1.6396	0.0299
Myo10	0.0000	1.2143	0.0059
Myo15b	0.0000	3.1119	0.0406
Myo16	0.0000	-1.9901	0.0069
Myo1f	0.0000	4.4922	0.0210
Myo5a	0.0000	-1.7645	0.0020
Myo9a	0.0000	-1.1585	0.0100
Myof	0.0000	1.7387	0.0124
Myom2	0.0000	2.0298	0.0416
Mypn	0.0000	2.7169	0.0449
Myrf	0.0000	2.3449	0.0068
Myt1	0.0000	-3.7521	0.0004
Myt1l	0.0000	-2.5627	0.0068
N4bp3	0.0000	2.1749	0.0073
Naaa	0.0000	1.0205	0.0308
Naalad2	0.0000	4.5295	0.0218
Nabp1	0.0000	2.1366	0.0038
Nacad	0.0000	-2.9409	0.0022
Nacc1	0.0000	1.3056	0.0044
Naf1	0.0000	1.2981	0.0101
Nagpa	0.0000	1.4438	0.0279
Nalcn	0.0000	-1.3724	0.0207
Nanog	0.0000	5.1275	0.0000
Nap1l2	0.0000	-1.9934	0.0081
Nap1l3	0.0000	-1.5175	0.0228
Napb	0.0000	-2.2752	0.0035
Narf	0.0000	-1.2215	0.0071
Nars	0.0000	1.1545	0.0066
Nat10	0.0000	1.1877	0.0142
Nat8l	0.0000	-1.4863	0.0110
Nav2	0.0000	-1.9482	0.0038
Nav3	0.0000	-2.4785	0.0070
Nbea	0.0000	-2.2731	0.0018
Ncald	0.0000	-1.2201	0.0139
Ncam1	0.0000	-1.5700	0.0036
Ncam2	0.0000	-2.1428	0.0167
Ncan	0.0000	-1.1062	0.0142
Nceh1	0.0000	1.5354	0.0145
Ncf2	0.0000	3.2837	0.0240
Nckap1l	0.0000	3.1357	0.0246
Ncl	0.0000	1.4554	0.0044
Ncoa5	0.0000	-1.1322	0.0101
Ncs1	0.0000	-1.8739	0.0047
Ndn	0.0000	-1.2258	0.0063
Ndnf	0.0000	-2.2573	0.0059
Ndrg1	0.0000	1.1348	0.0252
Ndrg2	0.0000	1.0684	0.0282
Ndrg3	0.0000	-1.2273	0.0117

Ndrg4	0.0000	-1.4430	0.0068
Ndst1	0.0000	-1.2999	0.0060
Ndst3	0.0000	-1.4818	0.0323
Ndst4	0.0000	-3.5857	0.0175
Ndufa13	0.0000	1.0307	0.0220
Ndufa4l2	0.0000	4.8802	0.0026
Ndufaf6	0.0000	1.1705	0.0324
Ndufb11	0.0000	1.0924	0.0085
Ndufb4	0.0000	1.1805	0.0212
Neat1	0.0000	6.1365	0.0006
Nebl	0.0000	-1.2181	0.0318
Necab1	0.0000	1.9556	0.0316
Nedd4l	0.0000	-1.3293	0.0125
Nefh	0.0000	2.5486	0.0064
Nefl	0.0000	-3.1050	0.0003
Nefm	0.0000	-3.2777	0.0067
Negr1	0.0000	-1.6627	0.0083
Neil2	0.0000	4.2094	0.0085
Nek10	0.0000	3.6536	0.0418
Nek2	0.0000	1.7390	0.0050
Nek7	0.0000	1.0201	0.0157
Nell1	0.0000	-1.6608	0.0254
Nell2	0.0000	-3.6651	0.0001
Nes	0.0000	-2.2163	0.0019
Neto1	0.0000	-2.1935	0.0393
Neto2	0.0000	-1.9444	0.0057
Neu3	0.0000	1.3651	0.0346
Neurl3	0.0000	3.7933	0.0283
Neurl4	0.0000	-1.0673	0.0109
Neurod6	0.0000	-2.0609	0.0375
Nexn	0.0000	-1.1977	0.0332
Nf1	0.0000	-1.3224	0.0089
Nfasc	0.0000	-1.9065	0.0029
Nfatc2	0.0001	1.2763	0.0478
Nfatc2ip	0.0000	1.3143	0.0102
Nfe2l2	0.0000	1.7160	0.0049
Nfe2l3	0.0000	-1.5033	0.0423
Nfia	0.0000	-1.3894	0.0155
Nfkb2	0.0000	1.5001	0.0180
Nfkbia	0.0000	2.0850	0.0062
Nfkbiz	0.0000	1.1383	0.0400
Ngfr	0.0000	-1.8231	0.0045
Nhlh2	0.0000	-2.7625	0.0131
Nhp2	0.0000	1.5527	0.0089
Nhs	0.0000	-1.0115	0.0273
Nicn1	0.0000	-1.1573	0.0126
Nid2	0.0000	1.3880	0.0054
Nin	0.0000	-1.3649	0.0119
Nip7	0.0000	1.1276	0.0119
Nipa1	0.0000	-1.5461	0.0135
Nkain1	0.0000	-2.0893	0.0051
Nkain2	0.0000	-1.4910	0.0443

Nkain3	0.0000	-2.9503	0.0152
Nkain4	0.0000	-1.0656	0.0257
Nkx2-2	0.0000	-1.9925	0.0220
Nkx6-1	0.0000	-2.9094	0.0017
Nle1	0.0000	1.0886	0.0262
Nlgn1	0.0000	-2.1238	0.0192
Nlgn2	0.0000	-1.4548	0.0058
Nlrp12	0.0000	5.4554	0.0142
Nlrp14	0.0000	5.0159	0.0315
Nlrx1	0.0000	4.7314	0.0328
Nme7	0.0000	1.0113	0.0246
Nmi	0.0000	2.4981	0.0430
Nmnat2	0.0000	-1.0879	0.0155
Nmrk1	0.0000	1.3882	0.0380
Nnat	0.0000	-1.5793	0.0026
Noct	0.0000	2.3604	0.0009
Nodal	0.0000	4.5913	0.0008
Nol4	0.0000	-2.2154	0.0179
Nol4l	0.0000	-1.8254	0.0046
Nop2	0.0000	1.0198	0.0137
Nop58	0.0000	1.2779	0.0037
Nos1	0.0000	1.5509	0.0224
Notch1	0.0000	-1.4426	0.0091
Notum	0.0000	3.4809	0.0347
Nova1	0.0000	-3.2851	0.0009
Nova2	0.0000	-2.6433	0.0010
Npas3	0.0000	-1.9283	0.0085
Npdc1	0.0000	-1.2290	0.0097
Npepl1	0.0000	3.4122	0.0063
Nphs1	0.0000	1.1186	0.0122
Npl	0.0000	1.0109	0.0442
Npm3	0.0000	1.3666	0.0117
Npr2	0.0000	-1.1348	0.0205
Nptx2	0.0000	2.3604	0.0071
Nqo1	0.0000	4.1689	0.0015
Nr0b1	0.0000	5.9387	0.0003
Nr2c1	0.0000	-1.6637	0.0173
Nr2c2	0.0000	-1.1281	0.0113
Nr2f1	0.0000	-2.3908	0.0010
Nr2f2	0.0000	-1.7538	0.0063
Nr3c2	0.0000	1.4930	0.0385
Nr4a1	0.0000	1.6230	0.0187
Nr4a2	0.0000	-1.9848	0.0102
Nrbp2	0.0000	-1.0373	0.0252
Nrcam	0.0000	-1.6525	0.0028
Nrep	0.0000	-2.2660	0.0007
Nrg1	0.0000	-1.5506	0.0207
Nrg3	0.0000	-3.0706	0.0298
Nrg4	0.0000	2.6267	0.0416
Nrgn	0.0000	-1.3259	0.0327
Nrip1	0.0000	-1.0635	0.0223
Nrxn1	0.0000	-1.8216	0.0074

Nrxn2	0.0000	-1.9126	0.0046
Nrxn3	0.0000	-2.7586	0.0027
Nsd2	0.0000	-1.4552	0.0034
Nsdhl	0.0000	-1.1822	0.0103
Nsg1	0.0000	-2.0237	0.0020
Nsl1	0.0000	1.2062	0.0216
Nsmf	0.0000	-1.3438	0.0121
Nt5c2	0.0000	-1.0430	0.0146
Nt5dc1	0.0000	1.7786	0.0201
Nt5m	0.0000	-1.3381	0.0242
Nthl1	0.0000	1.3702	0.0208
Ntn1	0.0000	-1.4005	0.0030
Ntn4	0.0000	-2.1941	0.0093
Ntng1	0.0000	-3.4548	0.0076
Ntng2	0.0000	-2.1065	0.0184
Ntpcr	0.0000	1.0971	0.0318
Ntrk1	0.0000	-1.3242	0.0426
Ntrk2	0.0000	-1.2806	0.0183
Ntrk3	0.0000	-3.0129	0.0013
Nts	0.0000	-3.9985	0.0209
Nuak1	0.0000	-1.2293	0.0141
Nudc	0.0000	1.1226	0.0168
Nudt10	0.0000	-1.3107	0.0227
Nudt17	0.0000	1.9027	0.0368
Nup133	0.0000	1.0153	0.0152
Nup62cl	0.0000	2.4914	0.0113
Nup93	0.0000	1.3377	0.0049
Nupr1	0.0000	4.1393	0.0011
Nwd1	0.0000	1.5940	0.0340
Nwd2	0.0000	-4.9367	0.0064
Nxn	0.0000	-1.4837	0.0064
Nxph1	0.0000	-5.0493	0.0090
Nyap1	0.0000	-1.7470	0.0048
Nyap2	0.0000	-1.6890	0.0245
Oas2	0.0000	3.8533	0.0350
Oas3	0.0000	4.8740	0.0251
Ocln	0.0000	2.4929	0.0205
Ogdhl	0.0000	1.6272	0.0033
Oip5	0.0000	1.2687	0.0273
Olfm1	0.0000	-2.5082	0.0017
Olfm2	0.0000	-2.3275	0.0030
Olfm3	0.0000	-2.1588	0.0284
Olig2	0.0000	-3.1519	0.0189
Oma1	0.0000	1.0441	0.0328
Onecut2	0.0000	-2.5303	0.0048
Onecut3	0.0000	-2.7588	0.0124
Ophn1	0.0000	-1.2268	0.0209
Opn3	0.0000	-1.0052	0.0460
Oprl1	0.0000	-2.3641	0.0091
Optn	0.0000	1.4628	0.0130
Orai2	0.0000	-1.1000	0.0270
Orai3	0.0000	1.3327	0.0169

Orc1	0.0000	2.5028	0.0044
Orc2	0.0000	1.6028	0.0040
Orc5	0.0000	1.0045	0.0197
Osbp2	0.0000	-1.4777	0.0370
Osbpl6	0.0000	-1.5901	0.0143
Osbpl8	0.0000	-1.1131	0.0145
Osmr	0.0000	4.5830	0.0198
Osr1	0.0000	-1.8181	0.0239
Ostf1	0.0000	2.1849	0.0045
Otud1	0.0000	2.2983	0.0120
Otud7a	0.0000	-1.2408	0.0358
Otx2	0.0000	4.3640	0.0006
P2rx7	0.0000	2.8200	0.0091
Padi2	0.0000	2.7113	0.0198
Padi4	0.0000	4.5021	0.0461
Pafah1b2	0.0000	-1.5350	0.0029
Pafah1b3	0.0000	-1.5417	0.0066
Pag1	0.0000	-1.3007	0.0168
Pak1	0.0000	-1.3824	0.0054
Pak3	0.0000	-2.0744	0.0035
Pak4	0.0000	1.2619	0.0102
Pak7	0.0000	-2.6251	0.0041
Palb2	0.0000	1.5122	0.0203
Palm3	0.0000	2.7858	0.0044
Pamr1	0.0000	1.8863	0.0325
Pank3	0.0000	-1.2388	0.0080
Panx1	0.0000	-1.7239	0.0042
Papss1	0.0000	-1.0978	0.0105
Paqr4	0.0000	-1.6934	0.0125
Paqr9	0.0000	-1.8156	0.0384
Pard6g	0.0000	-1.0962	0.0154
Parm1	0.0000	-1.2064	0.0159
Parp10	0.0000	1.4860	0.0366
Parp11	0.0000	-1.3052	0.0119
Parp12	0.0000	1.6981	0.0194
Parp14	0.0000	2.7995	0.0319
Parp3	0.0000	2.2340	0.0227
Parp4	0.0000	1.8607	0.0208
Parp6	0.0000	-2.4784	0.0028
Parp9	0.0000	1.5962	0.0277
Pask	0.0000	1.3203	0.0134
Patl2	0.0000	6.8376	0.0100
Pawr	0.0000	1.7644	0.0075
Pax2	0.0000	-4.2004	0.0020
Pax6	0.0000	-1.9695	0.0071
Pax7	0.0000	-2.9079	0.0111
Pax8	0.0000	-3.4475	0.0076
Pbx1	0.0000	-1.1700	0.0080
Pbx3	0.0000	-2.1646	0.0025
Pcbd1	0.0002	1.3175	0.0490
Pcbp4	0.0000	-1.0912	0.0118
Pcdh1	0.0000	-1.2370	0.0185

Pcdh10	0.0000	-3.0189	0.0039
Pcdh15	0.0000	-2.4853	0.0379
Pcdh17	0.0000	-2.6928	0.0052
Pcdh19	0.0000	-1.5426	0.0222
Pcdh7	0.0000	-1.3985	0.0429
Pcdh8	0.0000	-1.6377	0.0109
Pcdh9	0.0000	-3.6167	0.0024
Pcdha1	0.0000	-1.1329	0.0458
Pcdha3	0.0000	-2.3102	0.0078
Pcdha9	0.0000	-1.5660	0.0285
Pcdhb10	0.0000	-2.1242	0.0119
Pcdhb11	0.0000	-1.1461	0.0193
Pcdhb18	0.0000	-1.0473	0.0301
Pcdhb2	0.0000	-1.6301	0.0138
Pcdhb3	0.0000	-1.3176	0.0237
Pcdhb5	0.0000	-2.3653	0.0116
Pcdhb6	0.0000	-1.0833	0.0334
Pcdhb7	0.0000	-2.2655	0.0144
Pcdhb8	0.0000	-2.0292	0.0239
Pcdhga4	0.0000	-1.3396	0.0254
Pcdhgb6	0.0000	-1.6432	0.0159
Pcdhgb7	0.0000	-1.1502	0.0350
Pced1b	0.0000	3.1238	0.0043
Pck2	0.0000	2.0467	0.0029
Pclo	0.0000	-3.1133	0.0023
Pcolce2	0.0000	3.8431	0.0083
Pcp4	0.0000	-3.2507	0.0047
Pcp4l1	0.0000	2.7164	0.0037
Pcsk1	0.0000	5.8650	0.0086
Pcsk2	0.0000	-1.7707	0.0155
Pcsk5	0.0000	-1.6557	0.0179
Pcsk6	0.0000	2.6616	0.0017
Pdcl2	0.0000	6.4071	0.0170
Pde10a	0.0000	-2.3295	0.0051
Pde1b	0.0000	-1.4309	0.0105
Pde2a	0.0000	-1.1799	0.0299
Pde4c	0.0000	3.2375	0.0282
Pde4dip	0.0000	-1.0447	0.0155
Pde7b	0.0000	2.2658	0.0426
Pde8a	0.0000	5.0838	0.0030
Pdgfb	0.0002	1.3008	0.0498
Pdgfd	0.0000	3.1641	0.0280
Pdgfrl	0.0000	2.2615	0.0176
Pdk4	0.0000	4.9581	0.0135
Pdlim1	0.0000	2.0304	0.0084
Pdlim2	0.0000	1.1290	0.0419
Pdp1	0.0000	-1.3642	0.0114
Pdyn	0.0000	2.5277	0.0240
Pdzrn3	0.0000	-1.4523	0.0096
Pdzrn4	0.0000	-2.2004	0.0076
Pecam1	0.0000	4.6843	0.0003
Pecr	0.0000	1.8723	0.0110

Peli1	0.0000	-1.3594	0.0072
Peli2	0.0000	-1.7090	0.0088
Peli3	0.0000	-1.5044	0.0267
Perp	0.0000	1.5819	0.0147
Pex2	0.0000	-1.1981	0.0141
Pex5l	0.0000	-2.0276	0.0242
Pfkfb1	0.0000	1.6183	0.0327
Pfkfb3	0.0000	-1.5603	0.0058
Pfkfb4	0.0000	-2.2901	0.0153
Pfkp	0.0000	2.8514	0.0010
Pfn2	0.0000	-1.4664	0.0037
Pgam2	0.0000	1.9974	0.0374
Pgghg	0.0000	1.0626	0.0243
Pgm2l1	0.0000	-3.1445	0.0004
Phactr1	0.0000	-2.1053	0.0117
Phc1	0.0000	1.5613	0.0027
Phc2	0.0000	-1.3173	0.0055
Phf14	0.0000	-1.1282	0.0081
Phf19	0.0000	2.8930	0.0076
Phf2	0.0000	-1.4116	0.0063
Phf20l1	0.0000	-1.3041	0.0101
Phf21b	0.0000	-2.9182	0.0012
Phf24	0.0000	-2.7296	0.0064
Phf6	0.0000	-1.3142	0.0082
Phkg1	0.0000	1.7956	0.0438
Phldb1	0.0000	-1.1087	0.0133
Phyh	0.0000	1.5009	0.0116
Phyhd1	0.0000	2.1968	0.0366
Phyhipl	0.0000	-2.2161	0.0033
Pi15	0.0000	1.3599	0.0401
Piezo1	0.0000	1.5381	0.0063
Piezo2	0.0000	-2.1411	0.0382
Pif1	0.0000	1.2322	0.0237
Pigl	0.0000	1.0090	0.0299
Pik3c2b	0.0000	-1.2824	0.0155
Pik3ca	0.0000	-1.1532	0.0114
Pik3cd	0.0000	-1.2648	0.0283
Pik3r1	0.0000	-1.3075	0.0140
Pik3r3	0.0000	-2.3040	0.0004
Pim1	0.0000	1.6334	0.0067
Pim2	0.0000	2.5297	0.0038
Pim3	0.0000	1.6702	0.0069
Pink1	0.0000	-1.3211	0.0083
Pip4k2b	0.0000	-1.0401	0.0111
Pip4k2c	0.0000	1.0896	0.0155
Pir	0.0000	2.2938	0.0164
Pirt	0.0000	5.7270	0.0179
Pitpnm3	0.0000	-1.4269	0.0447
Piwil2	0.0000	4.7998	0.0069
Pja2	0.0000	-1.2470	0.0060
Pkdcc	0.0000	-1.1965	0.0122
Pkia	0.0000	-2.5047	0.0014

Pkn3	0.0000	1.7560	0.0212
Pknox2	0.0000	-1.0018	0.0219
Pkp2	0.0000	1.4052	0.0149
Pla1a	0.0000	5.1102	0.0132
Pla2g10	0.0000	4.0301	0.0236
Pla2g12a	0.0000	1.2947	0.0211
Pla2g4a	0.0000	1.9929	0.0229
Pla2g5	0.0000	4.9846	0.0168
Pla2g7	0.0000	1.2995	0.0299
Pla2r1	0.0000	4.4640	0.0408
Plagl2	0.0000	-1.1327	0.0170
Plaur	0.0000	1.6629	0.0367
Plbd1	0.0000	2.3156	0.0390
Plcb1	0.0000	-2.2958	0.0076
Plcd3	0.0000	1.7769	0.0176
Plcd4	0.0000	2.7425	0.0157
Plcg2	0.0000	3.3000	0.0048
Plch1	0.0000	-1.6286	0.0089
Plch2	0.0000	-1.7761	0.0321
Plcl1	0.0000	-1.7696	0.0146
Plcxd2	0.0000	-1.8652	0.0090
Plcxd3	0.0000	-2.1752	0.0399
Pld6	0.0000	3.8112	0.0150
Plek2	0.0000	4.8255	0.0360
Plekha4	0.0001	1.4200	0.0467
Plekha6	0.0000	-1.9457	0.0032
Plekha7	0.0000	1.6057	0.0115
Plekhf1	0.0000	2.0363	0.0143
Plekhf2	0.0000	1.9660	0.0027
Plekhg3	0.0000	2.3521	0.0050
Plekhh1	0.0000	1.2683	0.0150
Plekho1	0.0000	-1.4921	0.0116
Plet1	0.0000	2.9046	0.0275
Plin2	0.0000	1.5359	0.0088
Plin3	0.0000	1.6372	0.0047
Plk1	0.0000	1.3007	0.0107
Plk3	0.0000	-1.0789	0.0310
Plk5	0.0000	2.4925	0.0333
Pllp	0.0000	2.3678	0.0447
Plp2	0.0000	3.6906	0.0001
Plppr2	0.0000	-1.8007	0.0055
Plppr4	0.0000	1.3625	0.0430
Plscr1	0.0000	1.1097	0.0268
Plxdc1	0.0000	4.9520	0.0075
Plxna1	0.0000	-1.0796	0.0084
Plxna2	0.0000	-2.2606	0.0008
Plxna3	0.0000	-2.2933	0.0030
Plxna4	0.0000	-3.3358	0.0016
Plxnb1	0.0000	-1.8053	0.0029
Plxnc1	0.0000	-1.5631	0.0118
Pmaip1	0.0000	2.1357	0.0076
Pmel	0.0000	2.6607	0.0328

Pmf1	0.0000	1.7009	0.0086
Pml	0.0000	2.4405	0.0011
Pmm1	0.0000	1.4416	0.0058
Pms1	0.0000	1.3948	0.0142
Pnldc1	0.0000	4.1536	0.0171
Pnma3	0.0000	4.8610	0.0279
Pnma5	0.0000	4.5201	0.0047
Pnmal1	0.0000	-1.8030	0.0116
Pnmal2	0.0000	-1.5016	0.0072
Pnoc	0.0000	-1.5233	0.0303
Pnpla7	0.0000	1.3750	0.0277
Pnpt1	0.0000	1.5584	0.0068
Podxl2	0.0000	-1.6004	0.0064
Pogk	0.0000	-1.6288	0.0034
Pola1	0.0000	1.2134	0.0145
Pold1	0.0000	1.8602	0.0041
Pole	0.0000	1.5534	0.0076
Pole2	0.0000	1.7400	0.0156
Polr1a	0.0000	1.4714	0.0056
Polr2e	0.0000	1.4923	0.0050
Polr3g	0.0000	1.6624	0.0079
Polr3k	0.0000	-1.1591	0.0096
Pon3	0.0000	3.3004	0.0397
Pou2f2	0.0000	-2.5959	0.0158
Pou2f3	0.0000	4.1319	0.0211
Pou3f1	0.0000	-1.6089	0.0167
Pou3f2	0.0000	-2.6569	0.0022
Pou3f3	0.0000	-1.7228	0.0042
Pou4f1	0.0000	-2.6315	0.0054
Pou6f1	0.0000	-2.0854	0.0050
Ppan	0.0000	1.6552	0.0062
Ppargc1b	0.0000	1.6701	0.0317
Ppat	0.0000	1.3051	0.0109
Ppfia2	0.0000	-2.1999	0.0081
Ppfia3	0.0000	-1.8651	0.0089
Pphln1	0.0000	-1.0764	0.0169
Ppm1e	0.0000	-2.2933	0.0024
Ppm1h	0.0000	-1.1509	0.0197
Ppm1j	0.0000	4.1498	0.0105
Ppm1l	0.0000	-1.7929	0.0040
Ppm1m	0.0000	1.4170	0.0193
Ppm1n	0.0000	4.0473	0.0239
Ppme1	0.0000	-1.1407	0.0094
Ppp1r14a	0.0000	1.4951	0.0366
Ppp1r14c	0.0000	-1.7136	0.0213
Ppp1r15a	0.0000	1.0608	0.0247
Ppp1r15b	0.0000	1.1317	0.0070
Ppp1r17	0.0000	-3.0432	0.0242
Ppp1r26	0.0000	-1.1261	0.0196
Ppp1r3b	0.0000	1.6530	0.0163
Ppp1r3c	0.0000	-1.2497	0.0199
Ppp1r3d	0.0001	-1.1516	0.0483

Ppp1r3e	0.0000	-1.7855	0.0384
Ppp1r9a	0.0000	-1.3417	0.0082
Ppp1r9b	0.0000	-1.0602	0.0134
Ppp2r2b	0.0000	-1.7335	0.0120
Ppp2r3a	0.0000	-2.1398	0.0041
Ppp2r5a	0.0000	1.1806	0.0103
Ppp2r5b	0.0000	-1.5797	0.0063
Ppp6r2	0.0000	-1.0528	0.0147
Pqlc3	0.0000	1.8239	0.0118
Prdm1	0.0000	4.4275	0.0039
Prdm2	0.0000	-1.0267	0.0119
Prdm5	0.0000	1.9952	0.0146
Prdm8	0.0000	-3.0876	0.0029
Prdx1	0.0000	1.6989	0.0020
Prelid2	0.0000	3.1106	0.0118
Prelp	0.0000	2.0611	0.0116
Prepl	0.0000	-1.2016	0.0087
Prex1	0.0000	-1.3648	0.0094
Prickle1	0.0000	-1.3619	0.0148
Prickle3	0.0000	5.1682	0.0168
Prima1	0.0002	-1.9934	0.0497
Prkar2a	0.0000	-1.1541	0.0070
Prkar2b	0.0000	-1.7268	0.0045
Prkca	0.0000	-1.1271	0.0295
Prkce	0.0000	-1.6114	0.0106
Prkcg	0.0000	1.9630	0.0334
Prkch	0.0000	-1.4934	0.0291
Prkci	0.0000	1.1414	0.0102
Prkg1	0.0000	-1.3511	0.0338
Prkra	0.0000	-1.1391	0.0162
Prmt2	0.0000	-1.5087	0.0062
Prmt7	0.0000	1.3896	0.0056
Prmt8	0.0000	-2.5428	0.0031
Prodh	0.0000	1.2732	0.0338
Prox1	0.0000	-2.3710	0.0030
Prr16	0.0000	-3.1002	0.0397
Prr18	0.0000	-1.5371	0.0308
Prr36	0.0000	-1.7419	0.0081
Prrx2	0.0000	1.3799	0.0404
Prss16	0.0000	3.4223	0.0382
Prss35	0.0000	2.1421	0.0186
Prss50	0.0000	4.5961	0.0357
Prtn3	0.0000	-2.6141	0.0236
Prune2	0.0000	2.4577	0.0044
Prx	0.0000	1.2980	0.0233
Psat1	0.0000	1.0885	0.0135
Psd	0.0000	-1.7965	0.0104
Psd4	0.0000	3.8642	0.0156
Psmb9	0.0000	2.1707	0.0435
Psrc1	0.0000	1.1519	0.0147
Ptbp1	0.0000	1.2358	0.0053
Ptbp2	0.0000	-1.7065	0.0029

Ptchd4	0.0003	-3.8454	0.0500
Ptgds	0.0002	1.7342	0.0492
Ptger3	0.0001	-1.2035	0.0483
Ptgr1	0.0000	1.7453	0.0100
Ptgs2	0.0000	4.1117	0.0155
Pthlh	0.0000	1.2134	0.0463
Ptk2b	0.0000	2.5521	0.0197
Ptpn18	0.0001	4.3382	0.0473
Ptpn3	0.0000	1.4099	0.0182
Ptpn5	0.0000	-1.9660	0.0166
Ptpn6	0.0000	3.1854	0.0066
Ptprd	0.0000	-2.5066	0.0039
Ptprh	0.0000	3.7921	0.0466
Ptprm	0.0000	-1.1597	0.0276
Ptprn2	0.0000	-2.0081	0.0072
Ptpro	0.0000	-2.6839	0.0027
Ptprq	0.0000	2.7718	0.0428
Ptprr	0.0000	-2.1006	0.0345
Ptpru	0.0000	-1.2576	0.0195
Ptprz1	0.0000	-1.8686	0.0033
Pttg1	0.0000	1.4689	0.0147
Ptx3	0.0000	1.9056	0.0209
Pvt1	0.0000	2.9484	0.0216
Pxdn	0.0000	-1.4930	0.0046
Pxylp1	0.0000	-1.5883	0.0111
Pycard	0.0000	5.3674	0.0049
Pygl	0.0000	4.1384	0.0052
Pygo1	0.0000	-2.2005	0.0036
Pyroxd2	0.0000	2.7855	0.0409
R3hdm4	0.0000	-1.0327	0.0116
Rab11fip1	0.0000	1.5499	0.0126
Rab17	0.0000	3.4076	0.0433
Rab20	0.0000	1.8416	0.0453
Rab26	0.0000	2.4184	0.0445
Rab27a	0.0000	3.4651	0.0039
Rab30	0.0000	-2.2645	0.0059
Rab39b	0.0000	-2.1081	0.0061
Rab3a	0.0000	-1.2708	0.0139
Rab3c	0.0000	-3.0192	0.0010
Rab6b	0.0000	-2.5426	0.0010
Rab8b	0.0000	-1.2353	0.0106
Rabgap1l	0.0000	-1.2276	0.0136
Rac3	0.0000	-1.2468	0.0173
Rad18	0.0000	1.4582	0.0113
Rad50	0.0000	1.4427	0.0075
Rad51b	0.0000	1.1205	0.0466
Rad51c	0.0000	1.3064	0.0189
Rad54b	0.0000	1.7698	0.0110
Rad9a	0.0000	1.0652	0.0233
Rae1	0.0000	1.0582	0.0109
Rai1	0.0000	-1.0687	0.0115
Ralgapa1	0.0000	-1.3515	0.0080

Ralgds	0.0000	-1.4982	0.0078
Ralgps1	0.0000	-1.5778	0.0105
Ramp2	0.0000	1.0605	0.0368
Ranbp17	0.0000	1.9470	0.0068
Ranbp6	0.0000	-1.5752	0.0079
Rangrf	0.0000	1.6887	0.0151
Rapgef5	0.0000	-1.8478	0.0082
Rapgef6	0.0000	-1.2866	0.0086
Rapgefl1	0.0000	-1.6331	0.0258
Rarb	0.0000	-1.2084	0.0304
Rarg	0.0000	1.5634	0.0166
Rasal1	0.0000	3.3888	0.0367
Rasal2	0.0000	-1.0267	0.0158
Rasd2	0.0000	3.1977	0.0164
Rasgef1a	0.0000	2.5833	0.0391
Rasgef1b	0.0000	-1.0500	0.0375
Rasgef1c	0.0000	-3.4618	0.0265
Rasgrf2	0.0000	-3.0900	0.0220
Rasgrp1	0.0000	2.8690	0.0178
Rasgrp2	0.0000	3.0239	0.0066
Rasgrp4	0.0000	3.3306	0.0140
Rasl10b	0.0000	-2.2597	0.0034
Rasl11a	0.0000	-1.6119	0.0443
Rassf2	0.0000	-1.3635	0.0093
Rassf3	0.0000	1.6061	0.0095
Rassf4	0.0000	-1.0355	0.0304
Rassf9	0.0000	3.6844	0.0382
Rbfox1	0.0000	-2.5787	0.0083
Rbfox2	0.0000	-1.0827	0.0079
Rbfox3	0.0000	-2.9028	0.0035
Rbm43	0.0000	2.2964	0.0292
Rbm47	0.0000	2.6793	0.0140
Rbm4b	0.0000	-1.4263	0.0090
Rbm5	0.0000	-1.0233	0.0099
Rbms3	0.0000	-1.0412	0.0246
Rbp1	0.0000	-1.8752	0.0068
Rbpms	0.0000	2.9347	0.0007
Rbpms2	0.0000	4.0547	0.0002
Rc3h2	0.0000	-1.2125	0.0139
Rcan2	0.0000	-2.4463	0.0015
Rcl1	0.0000	1.2461	0.0164
Rcn2	0.0000	-1.0079	0.0080
Rcor2	0.0000	-1.1204	0.0137
Rdm1	0.0000	2.4838	0.0049
Rec8	0.0000	4.3578	0.0032
Reep1	0.0000	-1.7854	0.0049
Reep2	0.0000	-1.4275	0.0190
Reep3	0.0000	-1.2679	0.0102
Reep4	0.0000	1.0518	0.0251
Reep6	0.0000	1.3791	0.0246
Relb	0.0000	1.9934	0.0150
Rell2	0.0000	-1.2543	0.0336

Reln	0.0000	-4.4298	0.0007
Rem2	0.0000	-2.8364	0.0053
Renbp	0.0000	2.2882	0.0030
Reps2	0.0000	-1.2122	0.0325
Rerg	0.0000	1.2813	0.0386
Resp18	0.0000	-1.6404	0.0283
Rest	0.0000	1.8707	0.0044
Ret	0.0000	-2.2037	0.0119
Retsat	0.0000	2.9282	0.0070
Rev3l	0.0000	-1.7375	0.0054
Rfc5	0.0000	1.7104	0.0040
Rfx2	0.0000	1.2772	0.0199
Rfx3	0.0000	-1.1742	0.0163
Rfx4	0.0000	-1.9836	0.0030
Rgma	0.0000	-1.1675	0.0125
Rgs12	0.0000	-1.0979	0.0129
Rgs17	0.0000	-1.8561	0.0242
Rgs5	0.0000	2.1966	0.0311
Rgs7	0.0000	-2.1679	0.0194
Rgs7bp	0.0000	-1.3696	0.0316
Rgs8	0.0000	-2.0657	0.0073
Rhbdd1	0.0000	1.2171	0.0174
Rhbdf2	0.0000	1.1517	0.0273
Rhbdl3	0.0000	-2.7118	0.0037
Rhob	0.0000	-1.2238	0.0069
Rhobtb1	0.0000	-1.2305	0.0172
Rhpn1	0.0000	-1.6601	0.0211
Ribc1	0.0000	1.2324	0.0407
Rimbp2	0.0000	-1.3758	0.0263
Rimklb	0.0000	-1.2504	0.0073
Rims2	0.0000	-1.1912	0.0141
Rims4	0.0000	-1.0252	0.0333
Rin1	0.0000	2.2565	0.0200
Ripk2	0.0000	1.2035	0.0264
Ripk3	0.0000	3.4379	0.0313
Rit2	0.0000	1.6991	0.0459
Rmi2	0.0000	1.5208	0.0259
Rmnd5a	0.0000	1.0205	0.0101
Rnaseh2a	0.0000	1.0207	0.0185
Rnasel	0.0000	-1.5209	0.0148
Rnd2	0.0000	-2.4271	0.0038
Rnd3	0.0000	-1.7395	0.0015
Rnf112	0.0000	-2.0880	0.0310
Rnf122	0.0000	-1.4394	0.0124
Rnf125	0.0000	4.0566	0.0196
Rnf128	0.0000	1.8623	0.0210
Rnf130	0.0000	-1.1284	0.0092
Rnf135	0.0000	2.6056	0.0252
Rnf150	0.0000	-1.1220	0.0212
Rnf157	0.0000	-1.3427	0.0093
Rnf165	0.0000	-1.8595	0.0055
Rnf180	0.0000	-1.0254	0.0389

Rnf181	0.0000	1.3589	0.0079
Rnf187	0.0000	-1.0500	0.0066
Rnf217	0.0000	-1.2673	0.0229
Rnf219	0.0000	-1.0823	0.0191
Rnf24	0.0000	-1.5488	0.0093
Rnf38	0.0000	-1.8254	0.0026
Rnft2	0.0000	-1.2686	0.0148
Rnh1	0.0000	1.1174	0.0173
Rnls	0.0000	2.1750	0.0374
Robo1	0.0000	-1.8326	0.0032
Robo2	0.0000	-2.4763	0.0023
Robo3	0.0000	-2.9230	0.0032
Ror1	0.0000	1.3334	0.0270
Rorb	0.0000	-2.3321	0.0271
Rpa1	0.0000	1.1940	0.0065
Rpa3	0.0000	1.0010	0.0250
Rpgrip1l	0.0000	-1.1460	0.0204
Rph3a	0.0000	-1.9940	0.0275
Rpl12	0.0000	1.0991	0.0127
Rpl13	0.0000	1.0608	0.0095
Rpl18a	0.0000	1.2979	0.0068
Rpl28	0.0000	1.0039	0.0192
Rpl39l	0.0000	4.5041	0.0052
Rpl3l	0.0000	4.9408	0.0406
Rpl8	0.0000	1.0197	0.0084
Rpp25	0.0000	3.1190	0.0020
Rpp40	0.0000	1.3869	0.0253
Rps2	0.0000	1.3050	0.0081
Rps6ka1	0.0000	1.9272	0.0038
Rrad	0.0000	1.9291	0.0336
Rras	0.0000	1.0965	0.0244
Rrnad1	0.0000	-1.0455	0.0312
Rrp15	0.0000	1.4468	0.0083
Rrp1b	0.0000	1.2491	0.0103
Rrs1	0.0000	1.4669	0.0064
Rsbn1	0.0000	-1.1242	0.0108
Rspo2	0.0000	3.4631	0.0022
Rsrp1	0.0000	1.1820	0.0093
Rtn1	0.0000	-1.7253	0.0035
Rtn2	0.0000	-1.8658	0.0063
Rtn3	0.0000	-1.0655	0.0060
Rtn4r	0.0000	-2.0029	0.0202
Rtn4rl1	0.0000	1.5817	0.0237
Rtp4	0.0000	4.7167	0.0270
Rufy3	0.0000	-1.9209	0.0012
Rundc3b	0.0000	-1.8139	0.0099
Runx1	0.0001	-1.1194	0.0470
Runx1t1	0.0000	-1.6742	0.0152
Rusc1	0.0000	-1.8927	0.0070
S100a1	0.0000	4.7581	0.0057
S100a10	0.0000	1.6334	0.0094
S100a6	0.0000	2.9143	0.0145

Sall2	0.0000	-1.4806	0.0060
Sall3	0.0000	-2.1600	0.0034
Sall4	0.0000	2.7841	0.0023
Samd1	0.0000	1.1919	0.0121
Samd10	0.0000	-1.8541	0.0081
Samd12	0.0000	-1.2738	0.0228
Samd14	0.0000	-2.3067	0.0018
Samd9l	0.0000	2.6830	0.0268
Sap25	0.0002	2.2163	0.0490
Sarm1	0.0000	-1.9950	0.0082
Sat1	0.0000	1.4871	0.0108
Sat2	0.0000	1.1113	0.0434
Satb1	0.0000	-1.5457	0.0084
Sbk1	0.0000	-2.2669	0.0006
Scai	0.0000	-1.1326	0.0208
Scara3	0.0000	3.8105	0.0090
Scara5	0.0000	2.3408	0.0119
Scarb1	0.0000	1.2680	0.0125
Scarf1	0.0000	4.2890	0.0199
Scg3	0.0000	-2.7229	0.0009
Scg5	0.0000	-2.2001	0.0111
Scn1a	0.0000	-2.8013	0.0287
Scn1b	0.0000	2.1542	0.0134
Scn2a	0.0000	-2.6376	0.0056
Scn2b	0.0000	-1.1753	0.0372
Scn3a	0.0000	-2.2635	0.0035
Scn3b	0.0000	-1.7807	0.0045
Scn5a	0.0000	-2.3768	0.0442
Scn8a	0.0000	-1.2036	0.0221
Scn9a	0.0000	-4.2530	0.0108
Scpep1	0.0000	1.7730	0.0022
Scrn1	0.0000	-1.2472	0.0061
Scrn2	0.0000	-1.0659	0.0294
Scrn3	0.0000	1.1989	0.0222
Scrt1	0.0000	-2.7490	0.0014
Scrt2	0.0000	-3.6624	0.0007
Scube1	0.0000	-2.0670	0.0040
Scube2	0.0000	-1.9613	0.0292
Sdc3	0.0000	-1.3681	0.0068
Sdc4	0.0000	2.5713	0.0010
Sdk2	0.0000	-1.9824	0.0038
Sdr39u1	0.0000	4.2807	0.0108
Sec14l4	0.0000	5.0314	0.0332
Sec14l5	0.0000	-1.8972	0.0297
Secisbp2l	0.0000	-1.1012	0.0101
Selenbp1	0.0000	1.2110	0.0396
Sema3a	0.0000	-1.3569	0.0233
Sema3d	0.0001	-1.1141	0.0487
Sema4c	0.0000	-1.2350	0.0115
Sema5a	0.0000	-1.3092	0.0266
Sema6a	0.0000	-1.3115	0.0108
Sema6b	0.0000	-1.6522	0.0151

Sema6c	0.0000	-1.8295	0.0048
Sema6d	0.0000	-2.5243	0.0029
Sema7a	0.0000	-1.9205	0.0129
Senp7	0.0000	-1.7937	0.0053
Sephs2	0.0000	1.3366	0.0062
Serinc1	0.0000	-1.0415	0.0086
Serp2	0.0000	-1.1186	0.0458
Serpinb8	0.0000	3.5030	0.0394
Serpine1	0.0000	1.9759	0.0146
Serpinf1	0.0000	1.0708	0.0271
Serpini1	0.0000	-3.0121	0.0013
Sestd1	0.0000	-1.9584	0.0036
Setbp1	0.0000	-1.9543	0.0028
Setd4	0.0000	1.0208	0.0457
Setd6	0.0000	1.9361	0.0101
Sez6	0.0000	-2.4004	0.0037
Sez6l	0.0000	-2.4899	0.0037
Sez6l2	0.0000	-2.2663	0.0035
Sfn	0.0000	1.3454	0.0397
Sfpq	0.0000	-1.1525	0.0075
Sfrp4	0.0000	2.6230	0.0237
Sfrp5	0.0002	2.2712	0.0489
Sgip1	0.0000	-2.9162	0.0027
Sgk1	0.0000	1.8291	0.0061
Sgk2	0.0000	4.1274	0.0421
Sgk3	0.0000	4.3361	0.0023
Sgms2	0.0000	1.2337	0.0357
Sgtb	0.0000	-1.6540	0.0090
Sh2b2	0.0000	-2.0527	0.0120
Sh2d3c	0.0000	-1.9481	0.0075
Sh2d4a	0.0000	6.4517	0.0170
Sh2d5	0.0000	-1.4606	0.0360
Sh3bgrl	0.0000	-1.2815	0.0064
Sh3bgrl2	0.0000	2.1276	0.0095
Sh3bp1	0.0000	2.0161	0.0077
Sh3bp5	0.0000	-1.6426	0.0098
Sh3d19	0.0000	1.4668	0.0090
Sh3gl3	0.0000	-1.3288	0.0202
Sh3kbp1	0.0000	-1.2082	0.0195
Sh3pxd2b	0.0000	-1.3519	0.0064
Sh3tc1	0.0000	4.3986	0.0051
Sh3yl1	0.0000	-1.8316	0.0127
Shank1	0.0000	-2.3326	0.0048
Shank2	0.0000	-1.0966	0.0306
Shcbp1	0.0000	1.1720	0.0154
Shd	0.0000	-2.3176	0.0071
She	0.0000	3.1388	0.0124
Shf	0.0000	-1.6427	0.0118
Shisa7	0.0000	-1.8539	0.0121
Shisa8	0.0000	1.8691	0.0378
Shisa9	0.0000	-1.6411	0.0283
Shmt1	0.0000	2.4124	0.0026

Shmt2	0.0000	1.5111	0.0040
Siah3	0.0000	-3.1211	0.0158
Sigirr	0.0000	4.6606	0.0111
Sim1	0.0000	-5.4441	0.0104
Simc1	0.0000	-1.1440	0.0243
Sirt7	0.0000	-1.1622	0.0312
Siva1	0.0000	1.1362	0.0164
Six4	0.0000	1.8957	0.0104
Ska3	0.0000	1.3481	0.0164
Skida1	0.0000	-1.1918	0.0169
Skor1	0.0000	-2.8766	0.0178
Slc10a4	0.0000	-2.5787	0.0294
Slc12a4	0.0000	1.5073	0.0088
Slc12a5	0.0000	-2.5429	0.0038
Slc12a8	0.0000	4.8955	0.0077
Slc13a5	0.0000	3.8296	0.0175
Slc15a1	0.0000	5.9964	0.0135
Slc15a2	0.0000	-1.2667	0.0419
Slc16a1	0.0000	1.5412	0.0067
Slc16a10	0.0000	1.0614	0.0341
Slc16a12	0.0000	-1.8210	0.0311
Slc16a13	0.0000	-1.0959	0.0298
Slc16a14	0.0000	1.0919	0.0303
Slc16a6	0.0000	1.8351	0.0054
Slc16a7	0.0002	-1.0207	0.0498
Slc17a6	0.0000	-1.3217	0.0211
Slc18a3	0.0000	-3.5930	0.0112
Slc19a3	0.0000	4.1660	0.0206
Slc1a2	0.0000	-3.0507	0.0008
Slc1a5	0.0000	2.9478	0.0013
Slc20a1	0.0000	1.4174	0.0051
Slc20a2	0.0000	1.7616	0.0042
Slc22a15	0.0000	-1.9884	0.0200
Slc22a17	0.0000	-1.3552	0.0070
Slc22a23	0.0000	-1.6403	0.0056
Slc22a6	0.0000	-2.9283	0.0361
Slc22a7	0.0000	3.6843	0.0447
Slc24a3	0.0000	-1.5925	0.0206
Slc25a12	0.0000	-1.0721	0.0159
Slc25a13	0.0000	2.7739	0.0018
Slc25a15	0.0000	1.0726	0.0179
Slc25a27	0.0000	-1.9576	0.0134
Slc25a31	0.0000	6.2535	0.0098
Slc25a33	0.0000	1.3873	0.0201
Slc25a35	0.0001	1.3068	0.0475
Slc25a39	0.0000	1.5407	0.0052
Slc25a40	0.0000	1.0723	0.0312
Slc25a42	0.0000	1.2318	0.0321
Slc25a43	0.0000	4.8004	0.0360
Slc25a5	0.0000	1.7494	0.0019
Slc27a2	0.0000	2.6949	0.0218
Slc27a4	0.0000	-1.1492	0.0113

Slc29a1	0.0000	2.5529	0.0023
Slc29a4	0.0000	-1.5183	0.0109
Slc2a1	0.0000	1.1630	0.0055
Slc2a13	0.0000	-1.1931	0.0318
Slc2a3	0.0000	2.8454	0.0001
Slc2a5	0.0000	3.7777	0.0391
Slc32a1	0.0000	-2.4204	0.0138
Slc35d2	0.0000	2.6254	0.0326
Slc35f1	0.0000	-1.4637	0.0081
Slc35f2	0.0000	3.8363	0.0015
Slc35f4	0.0000	-2.2087	0.0383
Slc35g1	0.0000	1.3911	0.0261
Slc36a4	0.0000	-1.3237	0.0138
Slc37a2	0.0000	2.8932	0.0108
Slc37a4	0.0000	-1.3994	0.0170
Slc38a4	0.0000	1.7890	0.0110
Slc38a6	0.0000	4.4200	0.0000
Slc38a7	0.0000	1.4615	0.0091
Slc39a4	0.0000	4.9398	0.0023
Slc39a6	0.0000	-1.5782	0.0036
Slc3a2	0.0000	1.8743	0.0013
Slc40a1	0.0000	1.9459	0.0107
Slc43a3	0.0000	2.9753	0.0201
Slc44a1	0.0000	2.1765	0.0188
Slc44a3	0.0000	2.8303	0.0411
Slc44a5	0.0000	-2.6596	0.0042
Slc45a1	0.0002	-1.1442	0.0495
Slc45a4	0.0000	1.1642	0.0118
Slc46a3	0.0002	1.0297	0.0499
Slc47a1	0.0000	7.6419	0.0068
Slc4a10	0.0000	1.4046	0.0370
Slc4a11	0.0000	3.9995	0.0089
Slc4a5	0.0000	4.8991	0.0087
Slc4a8	0.0000	-1.6785	0.0081
Slc50a1	0.0000	3.6609	0.0094
Slc52a3	0.0000	3.7388	0.0337
Slc5a1	0.0000	3.1436	0.0406
Slc5a11	0.0000	3.7314	0.0203
Slc5a3	0.0000	-1.0651	0.0213
Slc6a1	0.0000	-1.5738	0.0084
Slc6a11	0.0000	-1.7416	0.0276
Slc6a13	0.0000	-2.9682	0.0153
Slc6a5	0.0000	-4.4813	0.0286
Slc7a1	0.0000	1.3541	0.0070
Slc7a14	0.0000	-2.0658	0.0160
Slc7a3	0.0000	3.3616	0.0006
Slc7a5	0.0000	2.1624	0.0008
Slc7a7	0.0000	2.5588	0.0087
Slc8a1	0.0000	-2.4422	0.0030
Slc8b1	0.0000	1.4729	0.0212
Slco1c1	0.0000	6.1344	0.0283
Slco3a1	0.0000	-1.5046	0.0171

Slco4a1	0.0000	1.7142	0.0263
Slco4c1	0.0000	4.6305	0.0086
Slco5a1	0.0000	-3.0831	0.0011
Slfn5	0.0000	6.0164	0.0384
Slit1	0.0000	-2.0918	0.0013
Slitrk1	0.0000	-3.7768	0.0072
Slitrk2	0.0000	-2.6180	0.0184
Slitrk3	0.0000	-3.5422	0.0151
Slitrk4	0.0000	-2.7863	0.0270
Slitrk5	0.0000	-2.1372	0.0060
Slx4ip	0.0000	-1.1928	0.0199
Smagp	0.0000	4.3085	0.0065
Smarcc2	0.0000	-1.9113	0.0026
Smarcd2	0.0000	1.1902	0.0143
Smc1b	0.0000	4.9111	0.0024
Smc2	0.0000	1.1401	0.0087
Smim18	0.0000	-3.4642	0.0230
Smim3	0.0000	1.7133	0.0345
Smoc2	0.0000	-1.2873	0.0354
Smpd3	0.0000	-1.9241	0.0041
Smtn	0.0000	1.1283	0.0183
Smtnl2	0.0000	2.5960	0.0032
Smyd1	0.0000	2.0642	0.0364
Snai3	0.0000	4.3040	0.0449
Snap25	0.0000	-2.2350	0.0040
Snap91	0.0000	-1.9742	0.0070
Snca	0.0000	-1.4895	0.0206
Sncaip	0.0000	-1.8685	0.0099
Sncb	0.0000	-1.7334	0.0319
Sncg	0.0000	-3.7191	0.0018
Snhg3	0.0000	1.4324	0.0415
Snn	0.0000	-1.8649	0.0026
Snph	0.0000	-2.0043	0.0229
Sntb2	0.0000	1.0223	0.0171
Snx20	0.0000	3.9760	0.0219
Snx32	0.0000	-1.5582	0.0329
Soat2	0.0000	3.3463	0.0372
Sobp	0.0000	-1.7351	0.0067
Socs7	0.0000	-1.0368	0.0114
Sod1	0.0000	1.1409	0.0067
Soga1	0.0000	-1.3375	0.0061
Soga3	0.0000	-2.8460	0.0007
Sorbs1	0.0000	-1.2170	0.0206
Sorbs2	0.0000	-1.2384	0.0120
Sorbs3	0.0000	1.1246	0.0352
Sorcs3	0.0000	-2.7996	0.0075
Sort1	0.0000	-1.1158	0.0071
Sowahb	0.0000	3.2656	0.0229
Sox11	0.0000	-2.7923	0.0003
Sox3	0.0000	-1.9785	0.0117
Sox4	0.0000	-1.7053	0.0025
Sox5	0.0000	-1.8408	0.0172

Sox6	0.0000	-1.6836	0.0191
Sox8	0.0000	-1.1824	0.0437
Sox9	0.0000	-1.1833	0.0164
Sp5	0.0000	1.2960	0.0408
Sp8	0.0000	-2.2151	0.0120
Sp9	0.0000	-2.6003	0.0322
Spag1	0.0000	1.0696	0.0424
Spag16	0.0000	4.1457	0.0172
Spag9	0.0000	-2.1806	0.0005
Spast	0.0000	-1.3082	0.0055
Spata5	0.0000	1.1087	0.0154
Spc24	0.0000	2.0508	0.0046
Spcs3	0.0000	1.0706	0.0086
Spef1	0.0000	-1.5226	0.0222
Spef2	0.0000	1.5547	0.0436
Sphkap	0.0000	-2.5053	0.0229
Spidr	0.0000	1.2475	0.0118
Spint2	0.0000	2.6654	0.0016
Spire1	0.0000	-1.2687	0.0096
Spn	0.0000	2.7339	0.0301
Spns2	0.0000	-2.3659	0.0129
Spock1	0.0000	-2.9734	0.0020
Spock2	0.0000	-1.3651	0.0051
Spock3	0.0000	-2.3880	0.0078
Spon1	0.0000	-1.9853	0.0010
Spp1	0.0000	5.5215	0.0001
Spr	0.0000	1.2557	0.0137
Spry4	0.0000	1.6513	0.0232
Spsb4	0.0000	-3.2478	0.0022
Sptbn2	0.0000	-2.9681	0.0003
Sptssa	0.0000	-1.0002	0.0151
Srcin1	0.0000	-2.0754	0.0044
Srd5a1	0.0000	-1.8906	0.0176
Srfbp1	0.0000	1.0072	0.0188
Srgap1	0.0000	-1.5137	0.0069
Srgap3	0.0000	-2.1428	0.0010
Srgn	0.0000	1.1223	0.0425
Srpx	0.0000	2.9373	0.0185
Srpx2	0.0000	2.2706	0.0403
Srr	0.0000	-1.5246	0.0103
Srrm3	0.0000	-2.8382	0.0052
Srrm4	0.0000	-3.6379	0.0008
Srxn1	0.0000	1.0383	0.0215
Ss18l1	0.0000	-2.2738	0.0029
Ssbp3	0.0000	-1.2713	0.0046
Ssbp4	0.0000	1.3964	0.0078
Ssh2	0.0000	-1.6774	0.0059
Sspn	0.0000	2.0242	0.0170
Sst	0.0000	-2.8552	0.0060
Sstr1	0.0000	-2.8995	0.0373
Sstr2	0.0000	-3.4054	0.0091
Sstr3	0.0000	-3.8072	0.0371

Sstr4	0.0000	-2.2973	0.0457
St14	0.0000	3.7159	0.0011
St18	0.0000	-2.7145	0.0032
St3gal1	0.0000	-1.8334	0.0117
St6gal1	0.0000	-1.0607	0.0152
St6gal2	0.0000	-2.2477	0.0105
St6galnac5	0.0000	-3.6342	0.0025
St7	0.0000	-1.6252	0.0118
St8sia1	0.0000	-1.7618	0.0074
St8sia2	0.0000	-1.9150	0.0023
St8sia3	0.0000	-1.8498	0.0121
St8sia4	0.0000	-2.8963	0.0028
St8sia5	0.0000	-2.0083	0.0453
St8sia6	0.0000	4.2679	0.0142
Stag3	0.0000	1.7367	0.0082
Stambpl1	0.0001	1.0720	0.0471
Stard8	0.0000	1.5514	0.0309
Stard9	0.0000	-1.0007	0.0334
Stat1	0.0000	1.0389	0.0232
Stat4	0.0000	4.5593	0.0169
Stc2	0.0000	1.5087	0.0109
Steap1	0.0000	3.4092	0.0130
Steap2	0.0000	1.5335	0.0133
Steap3	0.0000	3.9175	0.0083
Steap4	0.0000	7.5923	0.0120
Stk17b	0.0000	2.3463	0.0032
Stk26	0.0000	1.6488	0.0232
Stk32b	0.0000	-2.5600	0.0127
Stmn1	0.0000	-1.2846	0.0032
Stmn2	0.0000	-1.4992	0.0021
Stmn3	0.0000	-2.2604	0.0014
Stra6	0.0000	-2.2544	0.0179
Stx1a	0.0000	-1.4083	0.0230
Stx1b	0.0000	-2.2047	0.0048
Stx3	0.0000	2.3160	0.0034
Stxbp1	0.0000	-2.0252	0.0017
Stxbp2	0.0000	2.9299	0.0026
Stxbp4	0.0000	-1.4137	0.0094
Stxbp5l	0.0000	-4.1369	0.0252
Suclg2	0.0000	1.4280	0.0071
Susd1	0.0001	1.1363	0.0470
Sv2a	0.0000	-1.5132	0.0091
Sv2c	0.0000	-1.7443	0.0167
Svop	0.0000	-2.2646	0.0042
Swap70	0.0000	1.3652	0.0099
Syce1	0.0000	4.4146	0.0150
Sycp1	0.0000	1.7744	0.0359
Sycp2	0.0000	3.8765	0.0328
Sycp3	0.0000	4.6898	0.0017
Syk	0.0000	3.1180	0.0023
Syn2	0.0000	-1.2616	0.0273
Syn3	0.0000	-2.5492	0.0196

Syndig1l	0.0000	-2.0023	0.0440
Syne2	0.0000	-1.9348	0.0029
Syngr2	0.0000	1.2844	0.0195
Syngr3	0.0000	-1.3641	0.0236
Synj1	0.0000	-1.3936	0.0089
Synpo2	0.0000	-1.2542	0.0312
Synpr	0.0000	-2.1565	0.0156
Synrg	0.0000	-1.3501	0.0072
Syt1	0.0000	-2.7108	0.0015
Syt11	0.0000	-1.8565	0.0016
Syt13	0.0000	-1.0832	0.0320
Syt16	0.0000	-1.6110	0.0327
Syt17	0.0000	-1.0539	0.0389
Syt2	0.0000	-2.8308	0.0137
Syt4	0.0000	-2.3778	0.0015
Syt6	0.0000	-2.3084	0.0265
Syt9	0.0000	3.1899	0.0036
Tacr1	0.0000	-1.1447	0.0285
Tada2a	0.0000	1.0251	0.0185
Taf4b	0.0000	2.9859	0.0076
Taf5l	0.0000	1.5986	0.0044
Taf7	0.0000	1.1783	0.0086
Taf7l	0.0000	6.0808	0.0054
Tafa2	0.0000	-1.9877	0.0289
Tagln2	0.0000	2.6895	0.0007
Tagln3	0.0000	-2.7832	0.0005
Tal1	0.0000	-3.1496	0.0052
Tanc1	0.0000	1.2674	0.0082
Tanc2	0.0000	-2.0295	0.0030
Tango2	0.0000	-1.0562	0.0309
Tango6	0.0000	1.8309	0.0079
Taok1	0.0000	-1.0155	0.0117
Taok2	0.0000	-1.0038	0.0114
Tap2	0.0000	1.4262	0.0280
Tars	0.0000	1.0396	0.0096
Tbc1d16	0.0000	-1.8737	0.0014
Tbc1d2	0.0000	1.4634	0.0312
Tbc1d30	0.0000	-2.9649	0.0052
Tbc1d8	0.0000	1.3092	0.0246
Tbc1d9	0.0000	-1.4331	0.0058
Tbrg1	0.0000	1.5288	0.0031
Tbx3	0.0000	3.0245	0.0014
Tcaf1	0.0000	-2.2052	0.0004
Tcea3	0.0000	3.5325	0.0068
Tcerg1l	0.0000	-2.0160	0.0241
Tcf12	0.0000	-1.1679	0.0085
Tcf15	0.0000	1.7846	0.0213
Tcf24	0.0000	4.5844	0.0104
Tcf4	0.0000	-1.1738	0.0082
Tcf7	0.0000	2.6495	0.0031
Tcf7l1	0.0000	1.3646	0.0103
Tcirg1	0.0000	1.7029	0.0186

Tcof1	0.0000	1.2634	0.0061
Tcp11l2	0.0000	-1.5801	0.0250
Tdrd1	0.0000	2.8468	0.0387
Tdrd12	0.0000	2.6459	0.0105
Tdrd5	0.0000	2.7699	0.0363
Tdrkh	0.0000	-1.6815	0.0064
Tdrp	0.0000	3.7374	0.0012
Tead4	0.0000	3.2906	0.0058
Tek	0.0000	1.3114	0.0451
Tekt1	0.0000	5.6277	0.0241
Tenm1	0.0000	-3.1115	0.0143
Tenm2	0.0000	-2.0122	0.0056
Tent5a	0.0000	-1.4415	0.0272
Tep1	0.0000	1.1327	0.0277
Tesk1	0.0000	-1.0531	0.0174
Tesk2	0.0000	1.7071	0.0198
Tet3	0.0000	-2.1658	0.0014
Tex14	0.0000	5.1293	0.0014
Tfap4	0.0000	1.1192	0.0225
Tfcp2	0.0000	-1.1255	0.0149
Tfcp2l1	0.0000	5.5192	0.0001
Tfe3	0.0000	1.3438	0.0068
Tfrc	0.0000	-1.4106	0.0037
Tgfa	0.0000	-1.7169	0.0291
Tgfb1	0.0000	1.6965	0.0254
Tgfbi	0.0000	2.8090	0.0201
Tgfbr1	0.0000	-1.5783	0.0033
Tgif1	0.0000	1.9350	0.0031
Tgm1	0.0000	3.0303	0.0259
Tgm3	0.0000	1.9088	0.0455
Thap2	0.0000	-1.1687	0.0153
Thap4	0.0000	1.1672	0.0128
Thbd	0.0000	2.0150	0.0183
Thbs2	0.0000	1.3668	0.0442
Thbs4	0.0000	3.7705	0.0426
Them6	0.0000	2.0717	0.0194
Thnsl2	0.0000	1.6440	0.0355
Thra	0.0000	-1.0822	0.0131
Thsd7a	0.0000	-2.6425	0.0015
Thsd7b	0.0000	-1.8695	0.0379
Thy1	0.0000	2.8154	0.0107
Tia1	0.0000	-1.2854	0.0065
Tie1	0.0000	2.1582	0.0457
Tigd3	0.0000	-1.7068	0.0335
Timp1	0.0000	5.2514	0.0017
Timp4	0.0002	1.6335	0.0497
Tinf2	0.0000	1.0319	0.0218
Tjp3	0.0000	1.8563	0.0165
Tktl1	0.0001	4.6408	0.0468
Tlcd2	0.0000	2.5752	0.0443
Tle1	0.0000	-1.0473	0.0130
Tle6	0.0001	1.6752	0.0467

Tll2	0.0000	-4.1343	0.0201
Tlr2	0.0000	1.5360	0.0333
Tlr3	0.0000	3.6561	0.0376
Tlr4	0.0000	4.4656	0.0355
Tm4sf1	0.0000	4.2152	0.0047
Tm7sf3	0.0000	1.2976	0.0067
Tma16	0.0000	1.0816	0.0169
Tmbim1	0.0000	1.8925	0.0077
Tmc6	0.0000	2.8553	0.0138
Tmc8	0.0000	4.0969	0.0355
Tmco4	0.0000	5.1950	0.0079
Tmeff1	0.0000	-1.0127	0.0084
Tmeff2	0.0000	-2.4596	0.0082
Tmem106a	0.0000	2.1374	0.0349
Tmem107	0.0000	1.0651	0.0285
Tmem108	0.0000	-1.4193	0.0260
Tmem117	0.0000	1.7257	0.0283
Tmem132b	0.0000	-1.2062	0.0314
Tmem132c	0.0000	-1.0586	0.0259
Tmem132e	0.0000	-2.8055	0.0070
Tmem141	0.0002	-1.1279	0.0495
Tmem144	0.0000	2.9008	0.0208
Tmem14a	0.0000	1.2993	0.0289
Tmem150c	0.0000	-1.6165	0.0294
Tmem151b	0.0000	-2.0714	0.0044
Tmem154	0.0000	4.1324	0.0290
Tmem169	0.0000	-2.3720	0.0059
Tmem170b	0.0000	-1.5080	0.0086
Tmem176a	0.0000	1.5321	0.0155
Tmem178b	0.0000	-1.8616	0.0082
Tmem182	0.0000	3.6152	0.0349
Tmem192	0.0000	2.0502	0.0061
Tmem198	0.0000	-1.9649	0.0143
Tmem200a	0.0000	-1.0244	0.0283
Tmem200b	0.0000	1.8104	0.0450
Tmem229b	0.0000	-1.3872	0.0136
Tmem243	0.0000	1.2393	0.0354
Tmem25	0.0000	-1.5275	0.0428
Tmem255a	0.0000	-2.6091	0.0296
Tmem266	0.0000	-1.2221	0.0421
Tmem30a	0.0000	-1.0782	0.0098
Tmem33	0.0000	-1.0309	0.0099
Tmem37	0.0000	2.0628	0.0241
Tmem39a	0.0000	1.1768	0.0114
Tmem44	0.0000	-2.5894	0.0031
Tmem51	0.0000	2.8322	0.0115
Tmem59l	0.0000	1.3863	0.0407
Tmem62	0.0000	-2.0280	0.0312
Tmem63a	0.0000	1.2936	0.0351
Tmem63c	0.0001	-2.0936	0.0471
Tmem65	0.0000	-1.0776	0.0123
Tmem74	0.0000	-1.7109	0.0405

Tmem74b	0.0000	-1.4877	0.0411
Tmem86b	0.0001	-1.5775	0.0474
Tmem88	0.0000	2.0582	0.0297
Tmem8b	0.0000	-1.0980	0.0139
Tmod2	0.0000	-1.7485	0.0053
Tmtc2	0.0000	-2.3916	0.0034
Tmtc4	0.0000	-1.0558	0.0180
Tmub1	0.0000	-1.1466	0.0311
Tmub2	0.0000	-1.0471	0.0162
Tmx4	0.0000	-1.3087	0.0060
Tnfaip2	0.0000	2.0724	0.0246
Tnfrsf10b	0.0000	1.4351	0.0102
Tnfrsf12a	0.0000	1.2304	0.0290
Tnfrsf13b	0.0000	5.5151	0.0418
Tnfrsf1b	0.0000	5.0051	0.0058
Tnfrsf8	0.0000	3.9343	0.0429
Tnfsf13	0.0000	3.1159	0.0439
Tnik	0.0000	-1.4238	0.0098
Tnip1	0.0000	1.9950	0.0046
Tnmd	0.0000	2.3791	0.0375
Tnnt1	0.0000	2.2274	0.0251
Tnnt2	0.0000	2.0640	0.0297
Tnr	0.0000	-2.1700	0.0216
Tnrc6b	0.0000	-1.0114	0.0123
Tnxb	0.0000	1.8922	0.0365
Tomm20	0.0000	1.0585	0.0057
Tomm40	0.0000	1.0246	0.0124
Top2b	0.0000	-1.6526	0.0016
Tor3a	0.0000	2.5052	0.0092
Tor4a	0.0000	1.6536	0.0318
Tox	0.0000	-2.0582	0.0087
Tox2	0.0000	-2.0743	0.0072
Tox3	0.0000	-1.3774	0.0108
Tpd52	0.0000	1.6261	0.0038
Tpd52l1	0.0000	1.7940	0.0128
Tpgs2	0.0000	-1.0087	0.0194
Tph2	0.0000	1.2783	0.0446
Tpmt	0.0000	-1.6965	0.0129
Tppp	0.0000	-1.7487	0.0241
Traf1	0.0000	4.6336	0.0294
Traf3ip2	0.0000	2.1627	0.0165
Trafd1	0.0000	-1.3520	0.0106
Traip	0.0000	1.2271	0.0200
Tram1	0.0000	1.2391	0.0081
Trap1	0.0000	1.1964	0.0083
Trhde	0.0000	-5.3392	0.0185
Trib3	0.0000	4.2817	0.0013
Trim13	0.0000	1.4437	0.0132
Trim2	0.0000	-1.8632	0.0021
Trim25	0.0000	3.0917	0.0018
Trim36	0.0000	-3.1769	0.0018
Trim46	0.0000	-2.3230	0.0078

Trim47	0.0000	3.3563	0.0113
Trim52	0.0000	5.9440	0.0261
Trim54	0.0000	3.6607	0.0329
Trim6	0.0000	3.6735	0.0004
Trim62	0.0000	-1.6797	0.0127
Trim66	0.0000	3.9842	0.0039
Trim67	0.0000	-2.1104	0.0039
Trim7	0.0000	1.5279	0.0352
Trim71	0.0000	2.1805	0.0247
Trim9	0.0000	-2.4872	0.0026
Triml2	0.0000	5.6311	0.0006
Trio	0.0000	-2.0837	0.0021
Trip10	0.0000	1.2734	0.0144
Trip13	0.0000	1.4135	0.0111
Tro	0.0000	-1.4701	0.0046
Troap	0.0000	1.2706	0.0233
Trpm2	0.0000	2.5927	0.0285
Trpm3	0.0000	1.8648	0.0206
Trpv4	0.0000	3.1508	0.0451
Trrap	0.0000	-1.1410	0.0065
Tsen15	0.0000	1.4205	0.0209
Tshr	0.0000	-1.9530	0.0450
Tshz1	0.0000	-3.1451	0.0013
Tshz2	0.0000	-2.5168	0.0198
Tshz3	0.0000	-2.4508	0.0054
Tspan11	0.0000	-1.7350	0.0170
Tspan17	0.0000	-1.3728	0.0231
Tspan18	0.0000	-1.5127	0.0103
Tspan3	0.0000	-1.0796	0.0068
Tspo	0.0000	3.7695	0.0079
Tspyl4	0.0000	-1.9834	0.0019
Tst	0.0000	1.4178	0.0304
Ttbk2	0.0000	-1.8254	0.0032
Ttc28	0.0000	-1.8136	0.0038
Ttc39b	0.0000	2.9448	0.0022
Ttc39c	0.0000	-1.2550	0.0248
Ttc9b	0.0000	-2.2202	0.0162
Ttf2	0.0000	1.0579	0.0204
Ttll1	0.0000	-1.4373	0.0121
Ttll11	0.0001	-1.1344	0.0484
Ttll6	0.0000	4.7799	0.0197
Ttll7	0.0000	-2.3765	0.0161
Ttn	0.0000	1.6138	0.0395
Ttpa	0.0000	4.6404	0.0061
Ttpal	0.0000	-1.2552	0.0175
Ttr	0.0000	-1.8387	0.0396
Ttyh1	0.0000	-1.1558	0.0096
Ttyh3	0.0000	-1.2197	0.0042
Tub	0.0000	-2.1643	0.0027
Tuba1a	0.0000	-2.4323	0.0002
Tuba4a	0.0000	4.2395	0.0009
Tubb2b	0.0000	-1.9297	0.0006

Tubb3	0.0000	-2.3097	0.0003
Tubb6	0.0000	1.1764	0.0094
Tulp2	0.0000	4.5462	0.0191
Tulp3	0.0000	1.1337	0.0142
Tulp4	0.0000	-2.0595	0.0018
Twf2	0.0000	1.0986	0.0168
Tyms	0.0000	1.3029	0.0112
Uap1l1	0.0000	3.1126	0.0009
Uba52	0.0000	1.2314	0.0095
Ubap1l	0.0000	5.0770	0.0150
Ubash3b	0.0000	-1.0208	0.0365
Ube2c	0.0000	1.3839	0.0096
Ube2d1	0.0000	-1.4640	0.0067
Ube2e3	0.0000	-1.0703	0.0106
Ube2ql1	0.0000	-2.7717	0.0036
Ube2t	0.0000	2.5540	0.0053
Ube4a	0.0000	-1.0373	0.0155
Ubn2	0.0000	-1.6679	0.0034
Ubxn10	0.0000	3.0731	0.0176
Ubxn8	0.0000	1.0121	0.0282
Uchl1	0.0000	-1.4391	0.0037
Ucp2	0.0000	2.2916	0.0013
Uhrf1	0.0000	1.6554	0.0075
Ulk2	0.0000	-1.7481	0.0048
Umad1	0.0000	-1.4910	0.0176
Umps	0.0000	1.3374	0.0111
Unc13a	0.0000	-1.2039	0.0109
Unc13d	0.0000	2.6635	0.0293
Unc5a	0.0000	-2.5914	0.0095
Unc5c	0.0000	-2.1903	0.0100
Unc5d	0.0000	-2.3786	0.0116
Unc79	0.0000	-3.1214	0.0053
Unc80	0.0000	-1.0883	0.0437
Unc93b1	0.0000	2.5118	0.0284
Ung	0.0000	2.8287	0.0011
Upp1	0.0000	3.6322	0.0005
Uros	0.0000	-1.2633	0.0294
Ushbp1	0.0000	3.9126	0.0323
Usp20	0.0000	-1.2063	0.0175
Usp22	0.0000	-1.0347	0.0081
Usp26	0.0000	2.9123	0.0402
Usp3	0.0000	-1.2899	0.0112
Usp43	0.0000	1.9976	0.0351
Usp44	0.0000	1.4495	0.0290
Usp47	0.0000	-1.0048	0.0084
Usp9x	0.0000	1.1434	0.0051
Usp9y	0.0000	4.0163	0.0441
Utp4	0.0000	1.5881	0.0052
Uxs1	0.0000	1.2981	0.0144
Vamp8	0.0000	1.7687	0.0181
Vapb	0.0000	-1.0118	0.0122
Vash1	0.0000	-2.0744	0.0043

Vash2	0.0000	-2.3926	0.0028
Vasp	0.0000	1.2263	0.0072
Vat1l	0.0000	-1.8287	0.0052
Vav2	0.0000	-1.2564	0.0096
Vav3	0.0000	-3.1850	0.0034
Vcan	0.0000	-1.3458	0.0042
Vdr	0.0001	2.0300	0.0471
Vegfc	0.0000	1.9159	0.0230
Vezf1	0.0000	-1.3836	0.0054
Vezt	0.0000	-1.2678	0.0113
Vgf	0.0000	2.4961	0.0137
Vgll3	0.0000	-1.1119	0.0323
Vil1	0.0000	3.9656	0.0342
Vill	0.0000	1.7744	0.0241
Vit	0.0000	-2.0055	0.0196
Vldlr	0.0000	-1.1209	0.0161
Vopp1	0.0000	-1.7977	0.0023
Vps37b	0.0000	-1.2205	0.0152
Vps37d	0.0000	-1.6373	0.0130
Vps53	0.0000	-1.0618	0.0176
Vrk2	0.0000	1.1462	0.0332
Vsnl1	0.0000	-1.2600	0.0399
Vstm2a	0.0000	-1.9001	0.0270
Vstm2b	0.0000	-1.7379	0.0209
Vstm2l	0.0000	-1.5810	0.0150
Vsx1	0.0000	-4.7104	0.0346
Vta1	0.0000	-1.0709	0.0114
Vwa3a	0.0000	2.2480	0.0386
Vwa3b	0.0000	5.0978	0.0048
Vwa5b2	0.0000	-2.0255	0.0410
Vwf	0.0000	3.7575	0.0168
Was	0.0000	3.3747	0.0396
Wasf1	0.0000	-1.0698	0.0136
Wasf2	0.0000	1.2137	0.0089
Wbp1	0.0000	-1.1433	0.0112
Wbp2nl	0.0000	4.2233	0.0425
Wdfy3	0.0000	-1.5982	0.0031
Wdpcp	0.0002	-1.0500	0.0489
Wdr12	0.0000	1.3009	0.0096
Wdr17	0.0001	-1.1893	0.0481
Wdr31	0.0000	2.0703	0.0138
Wdr37	0.0000	-1.2676	0.0069
Wdr47	0.0000	-2.0586	0.0025
Wdr6	0.0000	-1.3814	0.0028
Wdr62	0.0000	1.3862	0.0188
Wdr63	0.0000	2.5987	0.0399
Wdr7	0.0000	-1.5660	0.0057
Wdr75	0.0000	1.3127	0.0079
Wdr76	0.0000	1.4913	0.0144
Wdr86	0.0000	1.1253	0.0436
Wee1	0.0000	1.1353	0.0149
Wfdc2	0.0000	5.6308	0.0040

Wnk2	0.0000	-1.8157	0.0035
Wnk3	0.0000	-1.5427	0.0085
Wnt10b	0.0000	2.7191	0.0395
Wnt11	0.0000	-1.3198	0.0350
Wnt2b	0.0000	-1.3477	0.0439
Wnt4	0.0000	-2.7440	0.0078
Wnt5a	0.0000	-1.1215	0.0178
Wnt7a	0.0000	-2.9466	0.0049
Wnt7b	0.0000	-2.2016	0.0286
Wsb1	0.0000	-1.5289	0.0030
Wscd1	0.0000	-1.1339	0.0168
Wscd2	0.0000	-1.9258	0.0089
Wtip	0.0000	1.2415	0.0226
Wwc2	0.0000	1.1619	0.0081
Xaf1	0.0000	3.9536	0.0068
Xk	0.0000	2.0001	0.0181
Xkr4	0.0000	-2.2975	0.0090
Xkr6	0.0000	-1.3133	0.0277
Xkr7	0.0000	-2.7968	0.0106
Xpr1	0.0000	-1.6323	0.0035
Xrcc5	0.0000	2.5817	0.0004
Xylb	0.0000	1.3243	0.0333
Ybx3	0.0000	1.1826	0.0066
Yif1b	0.0000	1.2884	0.0112
Ypel1	0.0000	-1.7214	0.0077
Ypel4	0.0000	-2.5336	0.0160
Zap70	0.0000	5.5298	0.0209
Zbtb16	0.0000	-2.3758	0.0135
Zbtb18	0.0000	-1.0166	0.0177
Zbtb32	0.0000	4.0195	0.0293
Zbtb38	0.0000	1.1230	0.0202
Zbtb39	0.0000	-1.0402	0.0206
Zbtb44	0.0000	1.5917	0.0051
Zbtb46	0.0000	-1.6563	0.0133
Zbtb5	0.0000	-1.4471	0.0064
Zbtb8a	0.0000	1.2298	0.0256
Zc3h12a	0.0000	2.0910	0.0338
Zc3h12b	0.0001	-1.2156	0.0482
Zc3h12c	0.0000	-1.7120	0.0131
Zc3h8	0.0000	1.3319	0.0308
Zc3hav1	0.0000	2.9540	0.0017
Zc3hav1l	0.0000	-1.2976	0.0107
Zc4h2	0.0000	-2.2336	0.0031
Zcchc10	0.0000	1.2891	0.0219
Zcchc18	0.0000	-1.1387	0.0140
Zcchc2	0.0000	1.0501	0.0183
Zcwpw1	0.0000	1.3575	0.0220
Zdbf2	0.0000	-1.0359	0.0104
Zdhhc14	0.0001	-1.1286	0.0475
Zdhhc17	0.0000	-1.2372	0.0121
Zdhhc9	0.0000	-1.0241	0.0201
Zeb1	0.0000	-1.0067	0.0115

Zeb2	0.0000	-1.3178	0.0095
Zfhx2	0.0000	-1.1641	0.0099
Zfhx3	0.0000	-1.9624	0.0029
Zfhx4	0.0000	-3.2228	0.0005
Zfp14	0.0000	-1.5472	0.0257
Zfp2	0.0000	-1.1462	0.0220
Zfp37	0.0000	-1.1516	0.0167
Zfp41	0.0000	-2.1483	0.0018
Zfp64	0.0000	1.0965	0.0146
Zfpm2	0.0000	-1.4505	0.0397
Zfr2	0.0000	-1.0533	0.0311
Zhx2	0.0000	-1.1593	0.0205
Zic3	0.0000	2.6276	0.0009
Zic5	0.0000	2.3477	0.0033
Zik1	0.0000	-1.5243	0.0092
Zkscan1	0.0000	-1.5644	0.0058
Zkscan3	0.0000	-1.0635	0.0129
Zmat4	0.0000	1.5771	0.0180
Zmiz1	0.0000	-1.9784	0.0019
Zmym6	0.0000	-1.6277	0.0066
Zmynd10	0.0000	1.8070	0.0437
Znrf1	0.0000	-1.1307	0.0124
Zranb3	0.0000	1.2080	0.0147
Zscan21	0.0000	-1.1609	0.0095
Zswim4	0.0000	-1.0352	0.0137
Zswim5	0.0000	-2.6366	0.0019
Zswim7	0.0000	1.8152	0.0187
Zswim8	0.0000	-1.1267	0.0088
Zwilch	0.0000	1.7091	0.0065
Zyg11b	0.0000	-1.2355	0.0071
Zzef1	0.0000	-1.0884	0.0180

Supplementary Table 8. Disease ontology (DisGeNet) analyses of genes with significantly altered transcript levels in *doxycycline-treated Tet/Tet* neurons

Term	Overlap	Adjusted P-value
Schizophrenia	488/1923	1.78E-20
Bipolar Disorder	251/837	8.23E-19
Colorectal Cancer	740/3298	3.98E-17
Neuroblastoma	427/1698	5.24E-17
Epilepsy	317/1177	1.55E-16
Central neuroblastoma	414/1655	4.24E-16
Autistic Disorder	205/677	7.40E-16
Intellectual Disability	580/2503	7.40E-16
Glioma	519/2211	5.05E-15
Glioblastoma	465/1937	5.05E-15
Carcinogenesis	861/4065	8.96E-14
Breast Carcinoma	1020/4963	3.09E-13
Colorectal Carcinoma	647/2931	3.09E-13
Prostatic Neoplasms	380/1554	3.33E-13
Liver Cirrhosis, Experimental	222/801	9.56E-13
Malignant neoplasm of ovary	470/2026	1.41E-12
Mammary Neoplasms	539/2387	1.77E-12
Colorectal Neoplasms	277/1073	4.11E-12
Malignant neoplasm of breast	1025/5054	9.05E-12
Autism Spectrum Disorders	168/572	9.88E-12
Mental Depression	168/575	1.57E-11
Neoplasm Metastasis	818/3920	1.80E-11
Depressive disorder	203/741	3.78E-11
Tumor Progression	474/2090	4.05E-11
Ovarian Carcinoma	494/2203	7.90E-11
Small cell carcinoma of lung	187/673	8.08E-11
Alcoholic Intoxication, Chronic	131/424	1.04E-10
Unipolar Depression	151/517	2.46E-10
Major Depressive Disorder	150/513	2.57E-10
Mental Retardation	285/1158	4.77E-10
Malignant tumor of colon	450/2001	5.93E-10
Epilepsy, Temporal Lobe	69/181	1.22E-09
Senile Plaques	99/301	1.22E-09
Seizures	286/1174	1.34E-09
Stomach Carcinoma	518/2378	2.21E-09
Malignant neoplasm of stomach	520/2398	4.06E-09
Secondary malignant neoplasm of lymph node	303/1271	4.06E-09
Status Epilepticus	78/221	4.10E-09
Amyloidosis	214/833	4.24E-09
Adenoma	248/1000	4.92E-09
Adenocarcinoma	388/1712	6.60E-09
Amyotrophic Lateral Sclerosis	176/660	1.09E-08
Medulloblastoma	161/590	1.09E-08
Generalized seizures	52/126	1.26E-08
Malignant neoplasm of prostate	671/3239	1.72E-08
Neurodevelopmental Disorders	73/208	2.02E-08
Prostate carcinoma	653/3145	2.13E-08
Colon Carcinoma	456/2091	3.16E-08
Astrocytoma	191/741	3.18E-08
Obesity	431/1961	3.51E-08
Esophageal carcinoma	179/685	3.63E-08
Mental disorders	101/329	4.20E-08
ovarian neoplasm	230/938	6.37E-08
Visual seizure	71/207	9.75E-08
Pancreatic Neoplasm	173/665	9.75E-08
Brain Neoplasms	169/646	9.75E-08
melanoma	520/2454	1.11E-07
Cognition Disorders	78/237	1.23E-07
Lung Neoplasms	276/1177	1.33E-07
Psychotic Disorders	96/317	2.23E-07
Impaired cognition	172/671	3.29E-07
Epileptic drop attack	46/115	3.76E-07
Tonic - clonic seizures	61/173	4.01E-07
Plaque, Amyloid	76/235	4.42E-07
Attention deficit hyperactivity disorder	116/412	5.28E-07
Epileptic encephalopathy	128/468	5.87E-07

Bladder Neoplasm	280/1217	6.52E-07
Seizures, Focal	49/129	8.16E-07
Coronary heart disease	219/913	9.68E-07
Solid Neoplasm	204/840	1.12E-06
nervous system disorder	120/436	1.12E-06
Acute Promyelocytic Leukemia	128/475	1.42E-06
Leukemia, Myelocytic, Acute	371/1703	1.54E-06
Diabetes Mellitus, Non-Insulin-Dependent	365/1672	1.59E-06
Congenital Heart Defects	84/277	1.73E-06
Esophageal Neoplasms	161/637	2.44E-06
Neurodegenerative Disorders	183/745	2.53E-06
Cerebral Ischemia	83/275	2.57E-06
Pilocytic Astrocytoma	46/122	2.64E-06
Carcinoma of lung	513/2476	2.86E-06
Hypotonic seizures	13/16	2.93E-06
Sudden loss of muscle tone	13/16	2.93E-06
Nonorganic psychosis	70/220	2.93E-06
Ewings sarcoma	100/352	2.93E-06
Transient Ischemic Attack	63/191	3.14E-06
Squamous cell carcinoma	401/1876	3.28E-06
Abnormal behavior	104/372	3.71E-06
Cerebral Infarction	84/283	4.30E-06
Primary malignant neoplasm of lung	473/2268	4.30E-06
Parkinson Disease	245/1064	4.37E-06
Malignant neoplasm of lung	506/2449	4.69E-06
Non-Small Cell Lung Carcinoma	468/2243	4.69E-06
Huntington Disease	157/627	5.74E-06
Mental deficiency	238/1032	5.75E-06
Brain Diseases	76/250	5.84E-06
leukemia	411/1941	6.02E-06
Renal Cell Carcinoma	299/1348	6.02E-06
Carcinoma of bladder	263/1162	6.35E-06
Malignant neoplasm of esophagus	166/673	6.68E-06
Glioblastoma Multiforme	202/854	8.01E-06
Lymphoma	290/1307	8.98E-06
Alzheimer Disease, Late Onset	76/253	9.40E-06
Anxiety	122/464	9.66E-06
Acute lymphocytic leukemia	212/907	9.69E-06
Narcolepsy	77/258	1.02E-05
Myoclonic Seizures	53/156	1.04E-05
Presenile dementia	100/362	1.04E-05
Infantile Severe Myoclonic Epilepsy	19/33	1.08E-05
Cerebrovascular accident	184/768	1.09E-05
Thyroid Neoplasm	124/476	1.24E-05
Mood Disorders	94/336	1.26E-05
Focal Clonic Seizures	10/11	1.29E-05
Dull intelligence	219/947	1.30E-05
Low intelligence	219/947	1.30E-05
Poor school performance	226/985	1.57E-05
Speech Delay	66/213	1.58E-05
Tremor	69/226	1.58E-05
Dementia	115/436	1.63E-05
Coronary Artery Disease	225/981	1.66E-05
Endometriosis	179/749	1.76E-05
Liver carcinoma	707/3593	1.84E-05
Other specified transient cerebral ischemias	52/155	1.86E-05
Absence Seizures	44/123	1.99E-05
Thyroid carcinoma	173/721	2.06E-05
Manic	36/93	2.56E-05
Squamous cell carcinoma of esophagus	232/1023	2.57E-05
Ataxia, Truncal	22/44	2.71E-05
Anxiety Disorders	105/396	3.80E-05
Adenomatous Polyposis Coli	104/392	4.14E-05
Malignant neoplasm of urinary bladder	254/1144	4.29E-05
Brugada Syndrome (disorder)	25/55	4.34E-05
Language Delay	63/206	4.34E-05
Pancreatic Ductal Adenocarcinoma	167/701	4.98E-05
Delayed speech and language development	66/220	5.04E-05

Mammary Neoplasms, Human	121/476	5.66E-05
Ataxia	63/208	6.10E-05
Pervasive Development Disorder	44/129	8.10E-05
Colonic Neoplasms	181/778	8.62E-05
Body mass index	98/370	8.63E-05
Finding of body mass index	98/370	8.63E-05
Cortical Dysplasia	27/64	9.03E-05
B-Cell Lymphomas	171/730	1.05E-04
Diabetes Mellitus	320/1507	1.05E-04
Malignant Neoplasms	307/1438	1.06E-04
Degenerative polyarthritis	219/976	1.07E-04
Depression, Bipolar	26/61	1.08E-04
Myocardial Infarction	217/966	1.09E-04
Petit mal status	30/76	1.23E-04
Congenital Abnormality	146/607	1.25E-04
Sarcoma	156/658	1.34E-04
Status Epilepticus, Subclinical	28/69	1.34E-04
Complex Partial Status Epilepticus	28/69	1.34E-04
Grand Mal Status Epilepticus	28/69	1.34E-04
Non-Convulsive Status Epilepticus	28/69	1.34E-04
Simple Partial Status Epilepticus	28/69	1.34E-04
Diabetes	274/1268	1.36E-04
Epilepsy, Generalized	24/55	1.50E-04
Tonic Seizures	37/104	1.55E-04
Meningioma	111/438	1.57E-04
Carcinoma, Spindle-Cell	57/188	1.61E-04
Brain Ischemia	74/264	1.61E-04
Drug Dependence	54/175	1.61E-04
Undifferentiated carcinoma	68/237	1.64E-04
Myoclonus	49/154	1.72E-04
Neoplasms	267/1236	1.78E-04
Precursor Cell Lymphoblastic Leukemia Lymphoma	147/618	1.99E-04
Convulsions	40/118	2.36E-04
Obtundation status	8/9	2.51E-04
Pschomotor retardation	8/9	2.51E-04
Endometrial Carcinoma	190/840	2.64E-04
Speech impairment	59/200	2.68E-04
Multiple Myeloma	280/1312	2.70E-04
Squamous cell carcinoma of the head and neck	208/934	2.79E-04
Idiopathic generalized epilepsy	22/50	2.97E-04
Coronary Arteriosclerosis	174/760	3.02E-04
Epileptic Seizures	36/103	3.02E-04
Age related macular degeneration	98/382	3.04E-04
Uterine Corpus Cancer	138/579	3.28E-04
Severe mental retardation (I.Q. 20-34)	64/224	3.35E-04
Mesothelioma	98/383	3.37E-04
Complex partial seizures	39/116	3.57E-04
Refractory anemias	73/266	3.76E-04
Carcinoma	62/216	3.86E-04
Hodgkin Disease	140/592	4.18E-04
Malignant neoplasm of endometrium	137/577	4.18E-04
Memory impairment	73/267	4.25E-04
Benign Rolandic Epilepsy	13/22	4.27E-04
Diastolic blood pressure	39/117	4.33E-04
Convulsive Seizures	37/109	4.53E-04
Chronic Obstructive Airway Disease	155/669	4.54E-04
Benign Prostatic Hyperplasia	105/420	4.54E-04
Microcalcification	19/41	4.63E-04
Nasopharyngeal carcinoma	177/782	4.69E-04
Manic Disorder	21/48	4.81E-04
Pancreatic carcinoma	381/1869	4.81E-04
Malignant neoplasm of kidney	105/421	4.94E-04
Dyskinetic syndrome	29/78	5.46E-04
Anoxia	77/288	5.53E-04
Generalized Epilepsy with Febrile Seizures Plus	9/12	6.30E-04
Calcification of coronary artery	95/375	6.42E-04
Cardiomyopathy, Dilated	104/419	6.58E-04
Epilepsy, Rolandic	16/32	6.66E-04

Liver neoplasms	278/1321	7.52E-04
Malignant neoplasm of pancreas	375/1846	7.52E-04
Encephalomyelitis	89/348	7.81E-04
Renal carcinoma	120/500	7.92E-04
Malignant neoplasm of thyroid	121/505	7.92E-04
Lewy Body Disease	42/133	8.15E-04
Adenoid Cystic Carcinoma	59/208	8.17E-04
Anaplastic carcinoma	58/204	8.83E-04
Hyperactive behavior	161/709	8.90E-04
Myocardial Ischemia	109/448	9.93E-04
Seizures, Auditory	34/101	0.001048435
Seizures, Clonic	34/101	0.001048435
Seizures, Sensory	34/101	0.001048435
Seizures, Somatosensory	34/101	0.001048435
Atonic Absence Seizures	34/101	0.001048435
Generalized Absence Seizures	34/101	0.001048435
Gustatory seizure	34/101	0.001048435
Jacksonian Seizure	34/101	0.001048435
Non-epileptic convulsion	34/101	0.001048435
Nonepileptic Seizures	34/101	0.001048435
Olfactory seizure	34/101	0.001048435
Single Seizure	34/101	0.001048435
Vertiginous seizure	34/101	0.001048435
Ovarian Serous Adenocarcinoma	33/97	0.001067734
Vascular lesions	29/81	0.001086357
Cervix carcinoma	236/1105	0.001086357
Middle Cerebral Artery Occlusion	42/135	0.001103859
Heart valve disease	23/58	0.001105886
Cerebellar Ataxia	132/565	0.00110614
Conventional (Clear Cell) Renal Cell Carcinoma	145/631	0.00110614
Multiple Sclerosis	236/1106	0.001131682
Atrial Premature Complexes	35/106	0.001225017
Atherosclerosis	241/1134	0.001225017
Tumor Angiogenesis	121/511	0.001225193
Heartburn	48/162	0.001240553
Kidney Failure, Acute	80/310	0.001244243
Keloid	50/171	0.001255919
Tumoral calcinosis	19/44	0.001279819
Dysarthria	74/282	0.001315611
Ganglioneuroma	22/55	0.001331511
Ependymoma	40/128	0.001424344
Metastatic malignant neoplasm to brain	48/163	0.00143352
Cleft Lip with or without Cleft Palate	16/34	0.001437873
Adenocarcinoma of lung (disorder)	250/1187	0.001579166
Sciatic Neuropathy	32/95	0.001605899
Myxoid cyst	58/209	0.001608034
Liver Cirrhosis	154/683	0.001656859
Pain	115/485	0.001687333
Skin Neoplasms	68/256	0.001706074
Rhabdomyosarcoma	97/397	0.001927282
Restenosis	59/215	0.001933086
Burkitt Lymphoma	99/407	0.001938765
Endometrioma	62/229	0.001941316
High density lipoprotein measurement	82/324	0.001941316
Febrile Convulsions	24/64	0.00196443
Alcohol Use Disorder	30/88	0.002039704
Global developmental delay	233/1102	0.002114032
Secondary malignant neoplasm of liver	119/508	0.002114032
Neurodevelopmental delay	8/11	0.00216927
Immunologic Deficiency Syndromes	145/641	0.002204935
Classical Hodgkin's Lymphoma	48/166	0.00220614
Epithelial ovarian cancer	275/1329	0.002268752
Malignant Glioma	118/504	0.00227661
Hypercholesterolemia	64/240	0.002332325
Leukemogenesis	162/730	0.002363893
Atrial Fibrillation	74/288	0.002484747
Intestinal Neoplasms	46/158	0.002518856
Carcinomatosis	50/176	0.002518856

Calcinosis	20/50	0.002662329
Gastrointestinal Stromal Tumors	81/323	0.002812882
Pituitary Adenoma	75/294	0.002812882
Sporadic Breast Carcinoma	46/159	0.002932102
Parkinsonian Disorders	58/214	0.002979017
Substance abuse problem	45/155	0.00315254
Cardiomyopathy, Familial Idiopathic	94/388	0.003163871
Neuroendocrine Tumors	67/257	0.003217697
MYELODYSPLASTIC SYNDROME	152/683	0.003242664
Tracheoesophageal Fistula	19/47	0.003261434
Lymphoma, Follicular	88/359	0.003315149
Movement Disorders	48/169	0.003340042
Drug habituation	41/138	0.003521171
Nephroblastoma	90/370	0.003690258
Malignant neoplasm of large intestine	119/516	0.003870524
Developmental delay (disorder)	53/193	0.003902002
cervical cancer	205/965	0.003976097
Squamous cell carcinoma of lung	72/283	0.003978687
Congenital pes cavus	39/130	0.003978687
Epilepsy, Cryptogenic	23/63	0.003978687
Epileptic Syndromes	6/7	0.004081692
Anaplastic astrocytoma	47/166	0.004107807
Malignant neoplasm of gallbladder	55/203	0.004225334
Meningioma, benign, no ICD-O subtype	63/241	0.004437665
Familial lichen amyloidosis	44/153	0.004447759
Stomach Neoplasms	180/835	0.004487672
Gastroesophageal reflux disease	64/246	0.004531997
Hippocampal sclerosis	20/52	0.004606054
Ataxias, Hereditary	47/167	0.00468658
Acid reflux	41/140	0.00468658
Substance Dependence	41/140	0.00468658
Childhood Absence Epilepsy	8/12	0.004779349
Generalized tonic seizures	8/12	0.004779349
Dyslexia	27/80	0.004812114
Absence Epilepsy	22/60	0.004890916
Carcinoma, Transitional Cell	95/399	0.005155577
Involuntary jerking movements	37/123	0.005172026
Spasmodic movement	37/123	0.005172026
Urothelial Carcinoma	66/257	0.005202513
Breast adenocarcinoma	47/168	0.005271302
Down Syndrome	110/475	0.005298638
Hyperreflexia	75/301	0.005455179
Drug Use Disorders	36/119	0.005458583
idiopathic epilepsy	11/21	0.00560571
Osteosarcoma of bone	218/1042	0.005671458
Triglycerides measurement	82/336	0.005792041
Ankle clonus	10/18	0.005792041
Malignant neoplasm of gastrointestinal tract	67/263	0.005828537
Hematopoietic Neoplasms	52/192	0.005869921
Aura	22/61	0.006128657
Awakening Epilepsy	22/61	0.006128657
Vascular inflammations	41/142	0.00613069
Prostatic Intraepithelial Neoplasias	48/174	0.006246616
Malignant tumor of cervix	188/884	0.006246616
Aortic Aneurysm, Abdominal	72/288	0.006252318
Bronchopulmonary Dysplasia	51/188	0.006293551
Juvenile Myoclonic Epilepsy	15/35	0.006900262
Absent speech	27/82	0.007071834
Aggressive behavior	34/112	0.007176393
Addictive Behavior	55/208	0.007381468
Hyperkyphosis	44/157	0.007464763
Strabismus	95/404	0.007562558
Physical aggression	33/108	0.007585367
Carcinoma, Small Cell	35/117	0.007913208
Arteriosclerosis	225/1087	0.00803594
Encephalitis	41/144	0.008129598
Hirschsprung Disease	39/135	0.008129598
Intelligence	56/214	0.008491799

Familial Epilepsies	8/13	0.009443722
Infrequent generalized seizures	8/13	0.009443722
Amyotrophic Lateral Sclerosis, Sporadic	39/136	0.009455779
Stereotypic Movement Disorder	29/92	0.009455779
Nephrotic Syndrome	49/182	0.009495689
Drug Resistant Epilepsy	21/59	0.009709184
Familial benign neonatal epilepsy	9/16	0.009888646
Diabetic Nephropathy	125/561	0.009888646
Childhood Acute Lymphoblastic Leukemia	102/443	0.009919957
Sleep Apnea, Obstructive	38/132	0.010075581
Epithelioma	73/298	0.010095721
Aggressive reaction	33/110	0.010463263
Hematologic Neoplasms	124/557	0.01064385
Renal fibrosis	53/202	0.010832323
Nonverbal	25/76	0.01105636
Platyspondyly	25/76	0.01105636
Varicosity	25/76	0.01105636
Fetal Growth Retardation	74/304	0.011060214
Myeloid Leukemia	113/501	0.011104733
Malignant Pleural Mesothelioma	65/260	0.01111885
Malignant neoplasm of liver	171/805	0.01127867
Congenital contractural arachnodactyly	55/212	0.01127867
Seizure, Febrile, Simple	6/8	0.011284936
Metabolic Syndrome X	122/548	0.0114207
Epilepsies, Partial	18/48	0.011438111
Psychotic symptom	18/48	0.011438111
Short palpebral fissure	23/68	0.011481724
AURAL ATRESIA, CONGENITAL	21/60	0.011795554
Dry Eye Syndromes	21/60	0.011795554
Congenital hypoplasia of kidney	20/56	0.011795554
Acute leukemia	111/492	0.01188364
Malignant mesothelioma	80/335	0.012030189
Mental and motor retardation	211/1021	0.012110976
Primary malignant neoplasm	213/1032	0.012216037
Acute myelomonocytic leukemia	26/81	0.01252367
Osteosarcoma	226/1103	0.012609904
Organic Mental Disorders, Substance-Induced	34/116	0.012867102
Prescription Drug Abuse	34/116	0.012867102
Attention Deficit Disorder	24/73	0.013555601
Dwarfism	68/277	0.013564635
Malignant neoplasm of salivary gland	23/69	0.013986298
gallbladder neoplasm	17/45	0.014115966
Myxoid/Round Cell Liposarcoma	17/45	0.014115966
Ganglioglioma	21/61	0.014605329
Orbital separation excessive	86/368	0.015189861
Apraxia of Phonation	7/11	0.015189861
Carcinoid tumor no ICD-O subtype	31/104	0.015783306
Neointima	5/6	0.015783306
Neointima Formation	5/6	0.015783306
Bicornuate uterus	9/17	0.015857366
Kyphosis deformity of spine	45/168	0.015857366
Diabetes Mellitus, Experimental	33/113	0.015857366
Alloxan Diabetes	33/113	0.015857366
Streptozotocin Diabetes	33/113	0.015857366
Congenital heart disease	35/122	0.015878139
Mammary Carcinoma, Animal	40/145	0.016279073
Animal Mammary Neoplasms	40/145	0.016279073
Adenocarcinoma Of Esophagus	72/299	0.01629302
Spastic gait	15/38	0.016405406
Hyperalgesia, Thermal	32/109	0.016903689
Paroxysmal atrial fibrillation	22/66	0.017318453
Gait Ataxia	26/83	0.017679539
Refractive Errors	17/46	0.01798545
Substance-Related Disorders	35/123	0.018216229
Muscular fasciculation	18/50	0.018216229
leiomyosarcoma	33/114	0.018222203
Thyroid associated opthalmopathies	28/92	0.018656195
Cognitive changes	11/24	0.018785539

TARSAL-CARPAL COALITION SYNDROME	44/165	0.019213837
Hemangioma	30/101	0.019242731
IGA Glomerulonephritis	59/237	0.019398762
Abortion, Tubal	32/110	0.01946481
Colorectal cancer metastatic	53/208	0.01946481
Lymphoid leukemia	54/213	0.019831702
Acquired scoliosis	95/418	0.020260994
Recurrent tumor	81/347	0.020549366
Congenital Hypothyroidism	22/67	0.020916917
Rheumatoid Arthritis	356/1833	0.021174074
Growth Disorders	15/39	0.021480127
MIXED LINEAGE LEUKEMIA	48/185	0.021480127
Trisomy	53/209	0.021480127
Duane Retraction Syndrome	10/21	0.02155782
Sinus Node Dysfunction (disorder)	10/21	0.02155782
Urgency frequency syndrome	10/21	0.02155782
Hydrocephalus	62/253	0.021605668
Adenoma of large intestine	63/258	0.02173224
Squamous cell carcinoma of mouth	20/59	0.021863956
AMYOTROPHIC LATERAL SCLEROSIS 1, AUTOSOMAL RECESSIVE	12/28	0.021873762
Adult Acute Myeloblastic Leukemia	30/102	0.021933538
Substance Use Disorders	39/143	0.022030388
Charcot-Marie-Tooth Disease	71/298	0.022173393
Cerebral Palsy	27/89	0.022745449
Ischemic cardiomyopathy	33/116	0.023711669
Peripheral Neuropathy	73/309	0.023935716
Congenital deafness	69/289	0.023935716
Epilepsy, Absence, Atypical	6/9	0.023935716
Seizure, Febrile, Complex	6/9	0.023935716
Akinetic Petit Mal	6/9	0.023935716
eyelids (symptom)	6/9	0.023935716
Juvenile Absence Epilepsy	6/9	0.023935716
AMYOTROPHIC LATERAL SCLEROSIS 1	28/94	0.02488651
Autoimmune Diseases	215/1060	0.02488651
Cleft Palate	32/112	0.025238601
Cytogenetically normal acute myeloid leukemia	21/64	0.025572551
Neurilemmoma	37/135	0.025572551
Heart Diseases	76/325	0.02559205
Fanconi Anemia	69/290	0.025862625
Spondyloepimetaphyseal disorder	11/25	0.025886993
Urgency of micturition	11/25	0.025886993
Maternal hypertension	8/15	0.026035525
response to ACE inhibitor	8/15	0.026035525
Hypercholesterolemia, Familial	41/154	0.026399053
Amphetamine Addiction	24/77	0.026416193
High-Grade Prostatic Intraepithelial Neoplasia	24/77	0.026416193
Aortic Aneurysm, Thoracic	29/99	0.02661761
Tumor Promotion	31/108	0.02661761
Islet Cell Tumor	15/40	0.02661761
Motor symptoms	15/40	0.02661761
Infantile Spasm	17/48	0.027377479
oligodendroglioma	47/183	0.027666094
Gastrointestinal Neoplasms	34/122	0.027898988
Curvature of spine	92/408	0.028004904
Familial Alzheimer Disease (FAD)	48/188	0.028065898
Malignant Peripheral Nerve Sheath Tumor	48/188	0.028065898
Short stature	157/750	0.028065898
Small head	123/570	0.02894392
Hyalinosis, Segmental Glomerular	12/29	0.028954327
Keratosis Follicularis	12/29	0.028954327
Platelet hematocrit measurement	41/155	0.029038017
Salivary Gland Neoplasms	25/82	0.029038017
Red Blood Cell Count measurement	33/118	0.029852607
Other specified manifestations of hyperkinetic syndrome	10/22	0.030140089
Recurrent respiratory infections	53/213	0.030140089
Precancerous Conditions	99/446	0.031456204
Endometrial Neoplasms	58/238	0.03157677
Secondary malignant neoplasm of lung	144/683	0.031591725

Malignant Childhood Neoplasm	26/87	0.032053446
Ataxia, Appendicular	19/57	0.032320058
M5b Acute differentiated monocytic leukemia	30/105	0.032560222
Other specified types of schizophrenia, unspecified	18/53	0.033199632
Muscle Rigidity	33/119	0.03396821
Drug abuse	56/229	0.03396821
Ascites	16/45	0.03396821
Glaucoma, Primary Open Angle	39/147	0.03399681
Monosomy	39/147	0.03399681
Epstein-Barr Virus Infections	57/234	0.03399681
FANCONI ANEMIA, COMPLEMENTATION GROUP A (disorder)	60/249	0.034470835
Dejerine-Sottas Disease (disorder)	27/92	0.034633514
Hypertension, Portal	29/101	0.034723836
Lupus Erythematosus, Systemic	223/1113	0.034858324
Overactive bladder syndrome	11/26	0.034858324
Spontaneous abortion	37/138	0.035320547
Anaplastic thyroid carcinoma	45/176	0.035320547
Cognitive delay	196/966	0.035320547
Neurofibromatosis 1	52/210	0.035345399
Diffuse Astrocytoma	21/66	0.035345399
Skeletal dysplasia	21/66	0.035345399
Secondary malignant neoplasm of bone	76/330	0.035551759
insulinoma	46/181	0.035730015
EPILEPSY, CHILDHOOD ABSENCE, 1	5/7	0.036892555
Atypical absence seizure	5/7	0.036892555
Multiple polyps	20/62	0.036892555
Malignant Head and Neck Neoplasm	103/470	0.037337556
Profound Mental Retardation	49/196	0.037337556
Liver and Intrahepatic Biliary Tract Carcinoma	129/607	0.037428008
Cardiac Arrest	50/201	0.037658668
Amyotrophic Lateral Sclerosis With Dementia	12/30	0.037658668
familial atrial fibrillation	12/30	0.037658668
Persistent atrial fibrillation	12/30	0.037658668
Hepatosplenomegaly	19/58	0.038064326
Hyperlipoproteinemia Type IIa	40/153	0.038146696
Hydronephrosis	31/111	0.038296625
Carcinoma of larynx	45/177	0.038310797
Congenital chromosomal disease	108/497	0.038733691
Cortical cataract	8/16	0.038746987
Tetralogy of Fallot	34/125	0.038746987
Childhood Medulloblastoma	27/93	0.038746987
Joint laxity	27/93	0.038746987
Pituitary Neoplasms	69/296	0.038808783
Action Tremor	18/54	0.038858636
Essential Tremor	22/71	0.038924132
Amyotrophic Lateral Sclerosis, Guam Form	13/34	0.039184467
Parkinson Disease, Familial, Type 1	13/34	0.039184467
Acute Myeloid Leukemia, M1	32/116	0.03976746
Common acute lymphoblastic leukemia	14/38	0.040401118
Depressed nasal ridge	24/80	0.040541798
AMYOTROPHIC LATERAL SCLEROSIS 6 (disorder)	10/23	0.04062175
Childhood Non-Hodgkin Lymphoma	10/23	0.04062175
Abnormality of cardiovascular system morphology	21/67	0.040837801
Serum total cholesterol measurement	71/307	0.041061003
Acute Myeloid Leukemia (AML-M2)	33/121	0.041338238
Adenocarcinoma of large intestine	69/297	0.041338238
Hyperglycemia	106/488	0.041338238
Noninfiltrating Intraductal Carcinoma	67/287	0.041627388
Apraxia, Verbal	6/10	0.042046894
Lennox-Gastaut syndrome	6/10	0.042046894
Paraneoplastic Opsoclonus-Myoclonus Ataxia	6/10	0.042046894
Cardiovascular Diseases	162/787	0.042440806
Amphetamine-Related Disorders	23/76	0.042510131
Amphetamine Abuse	23/76	0.042510131
Lip and Oral Cavity Carcinoma	103/473	0.042794376
Asthma	258/1313	0.043211681
Fibrosis	43/169	0.043996263
Malignant lymphoma, lymphocytic, intermediate differentiation, diffuse	79/349	0.044116472

Fibrosarcoma	69/298	0.044116472
Angina Pectoris	19/59	0.044237331
Generalized myoclonic seizures	19/59	0.044237331
Chronic Kidney Diseases	85/380	0.044246229
Decreased number of peripheral myelinated nerve fibers	11/27	0.044705529
Learning Disabilities	22/72	0.04480974
Gallbladder Carcinoma	56/233	0.04480974
Renal Insufficiency	62/263	0.044834684
Mechanical Allodynia	30/108	0.045224642
Focal glomerulosclerosis	40/155	0.045283848
Follicular adenoma	36/136	0.045283848
Mammary Neoplasms, Experimental	41/160	0.046313977
Motor neuron atrophy	26/90	0.04637638
Kidney Diseases	134/639	0.046755667
Aplasia Cutis Congenita	49/199	0.046854948
Cirrhosis	95/433	0.046854948
Gingival Hyperplasia	17/51	0.046854948
Hypsarrhythmia	17/51	0.046854948
Lafora Disease	17/51	0.046854948
Carcinoma of ampulla of Vater	9/20	0.046854948
Pseudoachondroplasia	9/20	0.046854948
Soft skin	9/20	0.046854948
Velvety skin	9/20	0.046854948
Tumor Initiation	56/234	0.047992199
Acute pancreatitis	29/104	0.048126333
Leiomyosarcoma of uterus	13/35	0.048518952
Congenital diaphragmatic hernia	27/95	0.048774278
Fragile X Syndrome	27/95	0.048774278
Membranous glomerulonephritis	27/95	0.048774278
Alcohol consumption	14/39	0.048774278
Low Grade Lymphoma (neoplasm)	14/39	0.048774278

Supplementary Table 9. KEGG Pathway analyses of genes with significantly altered transcript levels in doxycycline-treated *Tet/Tet* neurons

KEGG Pathway	Overlap	Bejamini-Hochberg Adjusted P-value
Axon guidance	71/182	1.91E-10
MAPK signaling pathway	96/294	2.45E-09
Pathways in cancer	142/531	3.36E-07
Hepatocellular carcinoma	54/168	5.65E-05
Calcium signaling pathway	70/240	7.10E-05
Insulin secretion	33/86	7.10E-05
Aldosterone synthesis and secretion	36/98	7.10E-05
Arrhythmogenic right ventricular cardiomyopathy	30/77	1.12E-04
Cell adhesion molecules	47/148	1.67E-04
Focal adhesion	59/201	1.67E-04
Rap1 signaling pathway	61/210	1.67E-04
Nicotine addiction	19/40	1.67E-04
ECM-receptor interaction	32/88	1.82E-04
Fluid shear stress and atherosclerosis	44/139	2.57E-04
TNF signaling pathway	37/112	4.04E-04
Ras signaling pathway	64/232	4.49E-04
Breast cancer	44/147	9.38E-04
Dilated cardiomyopathy	32/96	9.38E-04
Signaling pathways regulating pluripotency of stem cells	43/143	9.38E-04
GABAergic synapse	30/89	0.001181
One carbon pool by folate	11/20	0.001663
Wnt signaling pathway	47/166	0.001918
Cholinergic synapse	35/113	0.001918
Regulation of actin cytoskeleton	58/218	0.002005
Glutamatergic synapse	35/114	0.002005
GnRH secretion	23/64	0.002005
PI3K-Akt signaling pathway	86/354	0.002005
Proteoglycans in cancer	55/205	0.002005
Dopaminergic synapse	39/132	0.002005
Hypertrophic cardiomyopathy	29/90	0.002509
Morphine addiction	29/91	0.002999
AGE-RAGE signaling pathway in diabetic complications	31/100	0.003173
Hippo signaling pathway	45/163	0.003273
Notch signaling pathway	21/59	0.003461
Adrenergic signaling in cardiomyocytes	42/150	0.003461
Phosphatidylinositol signaling system	30/97	0.003651
ErbB signaling pathway	27/85	0.004195
Inflammatory mediator regulation of TRP channels	30/98	0.004195
Human papillomavirus infection	79/331	0.004434
Amphetamine addiction	23/69	0.004682
Cortisol synthesis and secretion	22/65	0.004705
Cushing syndrome	42/155	0.006014
Leukocyte transendothelial migration	33/114	0.006047
Circadian entrainment	29/97	0.00675
Cell cycle	35/124	0.00675
Fc epsilon RI signaling pathway	22/68	0.008313
DNA replication	14/36	0.008677
C-type lectin receptor signaling pathway	30/104	0.00969
Progesterone-mediated oocyte maturation	29/100	0.010273
Synaptic vesicle cycle	24/78	0.010273
Glutathione metabolism	19/57	0.010651
Glycosaminoglycan biosynthesis	18/53	0.010683
Ferroptosis	15/41	0.010683
Glycosphingolipid biosynthesis	16/45	0.010683
Fc gamma R-mediated phagocytosis	28/97	0.011705
Retrograde endocannabinoid signaling	39/148	0.011705

Glioma	23/75	0.011705
cAMP signaling pathway	53/216	0.01197
Basal cell carcinoma	20/63	0.013662
Colorectal cancer	25/86	0.016463
Inositol phosphate metabolism	22/73	0.0169
p53 signaling pathway	22/73	0.0169
Neurotrophin signaling pathway	32/119	0.017949
Transcriptional misregulation in cancer	47/192	0.01949
Amoebiasis	28/102	0.021983
Cocaine addiction	16/49	0.023256
Choline metabolism in cancer	27/98	0.023416
Protein digestion and absorption	28/103	0.024138
Alanine, aspartate and glutamate metabolism	13/37	0.024138
Aldosterone-regulated sodium reabsorption	13/37	0.024138
Lipid and atherosclerosis	51/215	0.024138
Other glycan degradation	8/18	0.02428
Gastric acid secretion	22/76	0.024508
Apoptosis	36/142	0.025492
Hepatitis B	40/162	0.026718
Growth hormone synthesis, secretion and action	31/119	0.028101
Relaxin signaling pathway	33/129	0.029257
Gastric cancer	37/149	0.03056
Melanogenesis	27/101	0.03056
Oxytocin signaling pathway	38/154	0.03056
Other types of O-glycan biosynthesis	15/47	0.031003
Thyroid hormone signaling pathway	31/121	0.033471
Chagas disease	27/102	0.033471
GnRH signaling pathway	25/93	0.035039
Cellular senescence	38/156	0.035713
Osteoclast differentiation	32/127	0.036813
Thyroid hormone synthesis	21/75	0.036813
Lysosome	32/128	0.040473
Phospholipase D signaling pathway	36/148	0.041657
Platelet activation	31/124	0.043786
Melanoma	20/72	0.044757
Non-small cell lung cancer	20/72	0.044757
Cysteine and methionine metabolism	15/50	0.049367

Supplementary Table 10. Gene ontology terms identified using genes showing altered transcript levels in doxycycline-treated *Tet/Tet* neurons

Gene Ontology: Biological Processes	Overlap	Bejamini-Hochberg Adjusted P-value
Axonogenesis (GO:0007409)	97/188	2.02E-24
Nervous System Development (GO:0007399)	166/433	4.53E-24
Axon Guidance (GO:0007411)	79/149	6.23E-21
Chemical Synaptic Transmission (GO:0007268)	109/273	6.28E-17
Neuron Projection Guidance (GO:0097485)	65/124	8.90E-17
Cell-Cell Adhesion Via Plasma-Membrane Adhesion Molecules (GO:0098742)	78/172	1.31E-15
Regulation Of Neuron Projection Development (GO:0010975)	77/174	1.06E-14
Axon Development (GO:0061564)	51/99	1.27E-12
Synapse Organization (GO:0050808)	60/131	4.37E-12
Synapse Assembly (GO:0007416)	40/71	2.10E-11
Neuron Projection Morphogenesis (GO:0048812)	63/146	2.40E-11
Anterograde Trans-Synaptic Signaling (GO:0098916)	75/199	4.34E-10
Modulation Of Chemical Synaptic Transmission (GO:0050804)	54/123	5.46E-10
Cell Morphogenesis Involved In Neuron Differentiation (GO:0048667)	39/77	2.83E-09
Regulation Of Intracellular Signal Transduction (GO:1902531)	97/297	3.78E-09
Positive Regulation Of Cell Projection Organization (GO:0031346)	51/118	3.78E-09
Homophilic Cell Adhesion Via Plasma Membrane Adhesion Molecules (GO:0007156)	33/60	5.05E-09
Central Nervous System Development (GO:0007417)	93/283	5.90E-09
Cell Junction Assembly (GO:0034329)	43/95	2.07E-08
Regulation Of Synapse Assembly (GO:0051963)	29/51	2.42E-08
Regulation Of Anatomical Structure Morphogenesis (GO:0022603)	51/127	7.30E-08
Neuron Development (GO:0048666)	57/150	8.24E-08
Positive Regulation Of Neuron Projection Development (GO:0010976)	40/92	3.37E-07
Regulation Of Dendrite Development (GO:0050773)	24/41	3.72E-07
Neuron Projection Development (GO:0031175)	66/192	4.27E-07
Regulation Of Trans-Synaptic Signaling (GO:0099177)	22/36	5.25E-07
Regulation Of Cell Migration (GO:0030334)	121/434	6.21E-07
Regulation Of Axonogenesis (GO:0050770)	24/42	6.25E-07
Central Nervous System Neuron Differentiation (GO:0021953)	20/31	6.38E-07
Transmembrane Receptor Protein Tyrosine Kinase Signaling Pathway (GO:0007169)	87/284	7.82E-07
Neurotransmitter Secretion (GO:0007269)	24/43	1.07E-06
Generation Of Neurons (GO:0048699)	59/172	2.54E-06
Extracellular Structure Organization (GO:0043062)	42/109	6.65E-06
Regulation Of GTPase Activity (GO:0043087)	68/214	6.65E-06
Protein Phosphorylation (GO:0006468)	131/500	6.76E-06
External Encapsulating Structure Organization (GO:0045229)	42/110	8.51E-06
Signal Release From Synapse (GO:0099643)	21/38	1.00E-05
Positive Regulation Of Excitatory Postsynaptic Potential (GO:2000463)	15/22	1.53E-05
Regulation Of Neuron Apoptotic Process (GO:0043523)	40/105	1.72E-05
Regulation Of Monoatomic Ion Transmembrane Transporter Activity (GO:0032412)	30/69	2.09E-05
Modulation Of Excitatory Postsynaptic Potential (GO:0098815)	18/31	2.83E-05
Cardiac Muscle Cell Action Potential (GO:0086001)	17/29	4.72E-05
Calcium-Dependent Cell-Cell Adhesion Via Plasma Membrane Cell Adhesion Molecules (GO:0016339)	20/38	4.72E-05
Positive Regulation Of Axonogenesis (GO:0050772)	20/38	4.72E-05
Positive Regulation Of Cell Junction Assembly (GO:1901890)	27/61	4.72E-05
Positive Regulation Of Synapse Assembly (GO:0051965)	18/32	4.72E-05
Glutamate Receptor Signaling Pathway (GO:0007215)	19/35	4.72E-05
Regulation Of Axon Extension (GO:0030516)	19/35	4.72E-05
Regulated Exocytosis (GO:0045055)	24/51	4.88E-05
Positive Regulation Of DNA-templated Transcription (GO:0045893)	273/1243	6.05E-05
Regulation Of Synaptic Transmission, Glutamatergic (GO:0051966)	25/55	6.12E-05
Regulation Of Microtubule Depolymerization (GO:0031114)	15/24	6.12E-05
Cell Junction Organization (GO:0034330)	27/62	6.20E-05
Regulation Of Neurotransmitter Receptor Activity (GO:0099601)	21/42	6.46E-05
Synaptic Transmission, Glutamatergic (GO:0035249)	16/27	6.82E-05
Protein Autophosphorylation (GO:0046777)	52/162	1.05E-04
Regulation Of Cell Differentiation (GO:0045595)	59/193	1.26E-04
Negative Regulation Of Microtubule Polymerization Or Depolymerization (GO:0031111)	17/31	1.31E-04
Cell-Cell Junction Organization (GO:0045216)	28/68	1.36E-04
Regulation Of Cytoskeleton Organization (GO:0051493)	40/114	1.36E-04
Positive Regulation Of Macromolecule Metabolic Process (GO:0010604)	96/364	1.70E-04
Mitotic Cell Cycle Phase Transition (GO:0044772)	39/111	1.70E-04
Positive Regulation Of Phosphorylation (GO:0042327)	67/231	1.83E-04
Negative Regulation Of Protein Depolymerization (GO:1901880)	14/23	1.87E-04
Circulatory System Development (GO:0072359)	44/132	1.90E-04
Neuron Migration (GO:0001764)	24/55	1.96E-04
Positive Regulation Of Supramolecular Fiber Organization (GO:1902905)	35/96	1.96E-04
Cell-Cell Adhesion Mediated By Cadherin (GO:0044331)	15/26	1.97E-04
Inhibitory Synapse Assembly (GO:1904862)	10/13	2.17E-04

Cellular Component Assembly (GO:0022607)	73/260	2.21E-04
Transport Across Blood-Brain Barrier (GO:0150104)	32/85	2.21E-04
Cellular Response To Salt (GO:1902075)	38/109	2.43E-04
Regulation Of Small GTPase Mediated Signal Transduction (GO:0051056)	40/118	2.98E-04
Neuron Differentiation (GO:0030182)	53/173	2.98E-04
Regulation Of Hydrolase Activity (GO:0051336)	28/71	2.98E-04
Brain Development (GO:0007420)	52/169	3.13E-04
Regulation Of Cell Motility (GO:2000145)	41/123	3.45E-04
Vascular Transport (GO:0010232)	31/83	3.45E-04
Positive Regulation Of Developmental Growth (GO:0048639)	22/50	3.61E-04
Cell Cycle G2/M Phase Transition (GO:0044839)	19/40	3.89E-04
Regulation Of Apoptotic Process (GO:0042981)	163/705	3.95E-04
Peptidyl-Tyrosine Modification (GO:0018212)	23/54	4.17E-04
Metal Ion Transport (GO:0030001)	52/171	4.17E-04
Negative Regulation Of Cell Population Proliferation (GO:0008285)	97/379	4.34E-04
Negative Regulation Of Neuron Death (GO:1901215)	32/88	4.34E-04
Positive Regulation Of Axon Extension (GO:0045773)	12/19	4.36E-04
Regulation Of Neuronal Synaptic Plasticity (GO:0048168)	17/34	4.56E-04
Semaphorin-Plexin Signaling Pathway (GO:0071526)	17/34	4.56E-04
Phosphorylation (GO:0016310)	107/429	4.92E-04
Positive Regulation Of Synaptic Transmission (GO:0050806)	29/77	4.92E-04
Regulation Of Calcium Ion-Dependent Exocytosis (GO:0017158)	15/28	5.09E-04
Synaptic Vesicle Exocytosis (GO:0016079)	19/41	5.32E-04
Regulation Of MAPK Cascade (GO:0043408)	59/204	5.32E-04
Positive Regulation Of Protein Phosphorylation (GO:0001934)	96/377	5.43E-04
Potassium Ion Transport (GO:0006813)	40/122	5.71E-04
G2/M Transition Of Mitotic Cell Cycle (GO:0000086)	18/38	5.96E-04
Neurotransmitter Transport (GO:0006836)	22/52	6.36E-04
Protein Polymerization (GO:0051258)	25/63	6.41E-04
Positive Regulation Of Dendrite Extension (GO:1903861)	11/17	6.58E-04
Action Potential (GO:0001508)	17/35	6.58E-04
Synaptic Membrane Adhesion (GO:0099560)	9/12	6.84E-04
Negative Regulation Of Supramolecular Fiber Organization (GO:1902904)	27/71	7.12E-04
Positive Regulation Of Transcription By RNA Polymerase II (GO:0045944)	206/938	7.32E-04
Positive Regulation Of Nervous System Development (GO:0051962)	21/49	7.32E-04
Negative Regulation Of Microtubule Depolymerization (GO:0007026)	12/20	7.57E-04
Extracellular Matrix Organization (GO:0030198)	52/176	8.00E-04
Potassium Ion Transmembrane Transport (GO:0071805)	43/137	8.26E-04
Regulation Of Monoatomic Cation Transmembrane Transport (GO:1904062)	18/39	8.30E-04
Negative Regulation Of Cell Adhesion (GO:0007162)	27/72	8.96E-04
Response To Cytokine (GO:0034097)	40/125	9.39E-04
Regulation Of Endothelial Cell Migration (GO:0010594)	31/88	9.50E-04
Regulation Of Cell Population Proliferation (GO:0042127)	172/766	9.50E-04
SA Node Cell Action Potential (GO:0086015)	6/6	9.64E-04
Ephrin Receptor Signaling Pathway (GO:0048013)	19/43	9.90E-04
Learning (GO:0007612)	19/43	9.90E-04
Negative Regulation Of Neuron Projection Development (GO:0010977)	22/54	0.001088115
Positive Regulation Of JNK Cascade (GO:0046330)	28/77	0.001144931
Regulation Of Microtubule Cytoskeleton Organization (GO:0070507)	18/40	0.00116531
Cellular Response To Metal Ion (GO:0071248)	42/135	0.001168657
Cardiac Muscle Cell Contraction (GO:0086003)	11/18	0.001181726
Regulation Of Dendrite Extension (GO:1903859)	11/18	0.001181726
Regulation Of Microtubule Polymerization Or Depolymerization (GO:0031110)	14/27	0.001231364
Regulation Of JNK Cascade (GO:0046328)	36/110	0.001240691
Regulation Of Sodium Ion Transmembrane Transporter Activity (GO:2000649)	17/37	0.001325313
Positive Regulation Of Nucleic Acid-Templated Transcription (GO:1903508)	130/557	0.001355626
Negative Regulation Of Neuron Apoptotic Process (GO:0043524)	27/74	0.00136195
Positive Regulation Of Angiogenesis (GO:0045766)	38/119	0.00136195
Membrane Depolarization During Action Potential (GO:0086010)	9/13	0.001479014
Negative Regulation Of Myoblast Differentiation (GO:0045662)	9/13	0.001479014
Glial Cell Differentiation (GO:0010001)	16/34	0.001490407
Positive Regulation Of Cell Differentiation (GO:0045597)	74/283	0.001510998
Inorganic Cation Import Across Plasma Membrane (GO:0098659)	34/103	0.001543488
Regulation Of Heart Rate By Cardiac Conduction (GO:0086091)	18/41	0.001543488
Regulation Of Microtubule-Based Process (GO:0032886)	18/41	0.001543488
Peptidyl-Tyrosine Phosphorylation (GO:0018108)	25/67	0.001599307
Regulation Of Dendrite Morphogenesis (GO:0048814)	15/31	0.00165047
Regulation Of Stress Fiber Assembly (GO:0051492)	27/75	0.001658378
Negative Regulation Of Nucleic Acid-Templated Transcription (GO:1903507)	109/456	0.001844775
Protein Modification Process (GO:0036211)	159/711	0.001931532
Monoatomic Cation Transmembrane Transport (GO:0098655)	73/281	0.001982791
Negative Regulation Of Apoptotic Process (GO:0043066)	114/482	0.001982791

Cardiac Ventricle Development (GO:0003231)	11/19	0.002028073
Negative Regulation Of Axonogenesis (GO:0050771)	12/22	0.002032562
Positive Regulation Of Synaptic Transmission, Glutamatergic (GO:0051968)	12/22	0.002032562
Positive Regulation Of Protein Modification Process (GO:0031401)	61/225	0.002057186
Regulation Of Regulated Secretory Pathway (GO:1903305)	16/35	0.002065043
Cell Differentiation In Hindbrain (GO:0021533)	8/11	0.002117825
Peptidyl-Serine Modification (GO:0018209)	48/166	0.002155599
Cardiac Conduction (GO:0061337)	19/46	0.002363798
Positive Regulation Of Programmed Cell Death (GO:0043068)	65/245	0.002363798
Regulation Of Endothelial Cell Proliferation (GO:0001936)	31/93	0.002363798
Skeletal System Development (GO:0001501)	44/149	0.002418666
Positive Regulation Of Endothelial Cell Migration (GO:0010595)	29/85	0.002453845
Response To Calcium Ion (GO:0051592)	34/106	0.00256027
Plasma Membrane Bounded Cell Projection Organization (GO:0120036)	40/132	0.002643176
Positive Regulation Of Apoptotic Process (GO:0043065)	70/270	0.002688569
Neuron Cell-Cell Adhesion (GO:0007158)	9/14	0.002875387
Synaptic Transmission, GABAergic (GO:0051932)	9/14	0.002875387
Enzyme-Linked Receptor Protein Signaling Pathway (GO:0007167)	38/124	0.002943077
Positive Regulation Of MAPK Cascade (GO:0043410)	78/310	0.003141321
Positive Regulation Of GTPase Activity (GO:0043547)	62/234	0.003323856
Regulation Of Microtubule Polymerization (GO:0031113)	17/40	0.003396132
Positive Regulation Of Nitrogen Compound Metabolic Process (GO:0051173)	15/33	0.003396132
Positive Regulation Of Epithelial Cell Migration (GO:0010634)	31/95	0.00340958
Regulation Of ERK1 And ERK2 Cascade (GO:0070372)	64/244	0.003481116
Positive Regulation Of Gene Expression (GO:0010628)	112/480	0.003481116
Regulation Of Actin Cytoskeleton Organization (GO:0032956)	30/91	0.003481116
Positive Regulation Of Vasculature Development (GO:1904018)	33/104	0.003745768
Positive Regulation Of Neurogenesis (GO:0050769)	26/75	0.003799724
Positive Regulation Of Vascular Associated Smooth Muscle Cell Migration (GO:1904754)	6/7	0.00381063
Pyrimidine Nucleobase Catabolic Process (GO:0006208)	6/7	0.00381063
Cardiac Muscle Cell Action Potential Involved In Contraction (GO:0086002)	14/30	0.00381063
Negative Regulation Of Axon Extension (GO:0030517)	14/30	0.00381063
Calcium Ion Import Across Plasma Membrane (GO:0098703)	16/37	0.003923171
Negative Regulation Of Protein Kinase Activity (GO:0006469)	29/88	0.004313528
Regulation Of Catecholamine Secretion (GO:0050433)	13/27	0.004379882
Ionotropic Glutamate Receptor Signaling Pathway (GO:0035235)	8/12	0.004424019
Positive Regulation Of Regulated Secretory Pathway (GO:1903307)	8/12	0.004424019
Semaphorin-Plexin Signaling Pathway Involved In Neuron Projection Guidance (GO:1902285)	8/12	0.004424019
Positive Regulation Of Ossification (GO:0045778)	17/41	0.004424019
Positive Regulation Of Neuron Death (GO:1901216)	15/34	0.004627827
Regulation Of AMPA Receptor Activity (GO:2000311)	12/24	0.004829938
Sympathetic Nervous System Development (GO:0048485)	9/15	0.005252241
Heterophilic Cell-Cell Adhesion Via Plasma Membrane Cell Adhesion Molecules (GO:0007157)	19/49	0.005252241
Negative Chemotaxis (GO:0050919)	14/31	0.005495131
Regulation Of Alternative mRNA Splicing, Via Spliceosome (GO:0000381)	20/53	0.005495131
Negative Regulation Of Cell Motility (GO:2000146)	39/133	0.005745778
Cellular Response To Calcium Ion (GO:0071277)	24/69	0.005757065
Canonical Wnt Signaling Pathway (GO:0060070)	23/65	0.005757065
Dendrite Morphogenesis (GO:0048813)	15/35	0.006490806
Regulation Of Sodium Ion Transport (GO:0002028)	15/35	0.006490806
Regulation Of Protein Phosphorylation (GO:0001932)	67/265	0.006692351
Positive Regulation Of Tumor Necrosis Factor Production (GO:0032760)	26/78	0.006839393
Memory (GO:0007613)	21/58	0.007184536
Regulation Of Neuron Death (GO:1901214)	21/58	0.007184536
Positive Regulation Of Hydrolase Activity (GO:0051345)	48/176	0.007520907
Positive Regulation Of Kinase Activity (GO:0033674)	32/104	0.007520907
Calcium Ion Transmembrane Import Into Cytosol (GO:0097553)	27/83	0.008033015
Regulation Of Neuron Differentiation (GO:0045664)	26/79	0.0083275
Negative Regulation Of DNA-templated Transcription (GO:0045892)	213/1025	0.008530121
Protein Localization To Synapse (GO:0035418)	8/13	0.00876226
Positive Regulation Of Actin Filament Bundle Assembly (GO:0032233)	19/51	0.00876226
Positive Regulation Of Stress-Activated MAPK Cascade (GO:0032874)	32/105	0.00883164
Positive Regulation Of Microtubule Polymerization (GO:0031116)	13/29	0.008949793
Response To Interferon-Beta (GO:0035456)	13/29	0.008949793
Autonomic Nervous System Development (GO:0048483)	9/16	0.008949793
Membrane Depolarization During Cardiac Muscle Cell Action Potential (GO:0086012)	9/16	0.008949793
Regulation Of NMDA Receptor Activity (GO:2000310)	9/16	0.008949793
Regulation Of Presynapse Assembly (GO:1905606)	9/16	0.008949793
Regulation Of Presynapse Organization (GO:0099174)	9/16	0.008949793
Synaptic Vesicle Endocytosis (GO:0048488)	16/40	0.009538412
Inorganic Cation Transmembrane Transport (GO:0098662)	70/284	0.009859204
Wnt Signaling Pathway (GO:0016055)	25/76	0.010122205

Tetrahydrofolate Interconversion (GO:0035999)	6/8	0.010448827
Positive Regulation Of Ion Transmembrane Transporter Activity (GO:0032414)	14/33	0.010448827
Calcium-Ion Regulated Exocytosis (GO:0017156)	12/26	0.010448827
Positive Regulation Of Reactive Oxygen Species Metabolic Process (GO:2000379)	18/48	0.01053853
Synaptic Vesicle Cycle (GO:0099504)	15/37	0.011680831
Ventricular Septum Development (GO:0003281)	15/37	0.011680831
Cellular Response To Oxygen-Containing Compound (GO:1901701)	94/406	0.011718132
Positive Regulation Of Transferase Activity (GO:0051347)	40/143	0.011827729
Protein Localization To Cell Junction (GO:1902414)	11/23	0.0120334
Positive Regulation Of Cell Communication (GO:0010647)	13/30	0.012491677
Regulation Of Potassium Ion Transport (GO:0043266)	13/30	0.012491677
Ras Protein Signal Transduction (GO:0007265)	40/144	0.013436804
Regulation Of Cell Adhesion (GO:0030155)	40/144	0.013436804
Regulation Of Angiogenesis (GO:0045765)	53/205	0.013436804
Negative Regulation Of Viral Process (GO:0048525)	21/61	0.013436804
Gamma-Aminobutyric Acid Signaling Pathway (GO:0007214)	10/20	0.013436804
Actin Polymerization Or Depolymerization (GO:0008154)	19/53	0.013455291
Peptidyl-Serine Phosphorylation (GO:0018105)	43/158	0.013455291
Positive Regulation Of Tumor Necrosis Factor Superfamily Cytokine Production (GO:1903557)	26/82	0.013642786
Pyrimidine Nucleobase Metabolic Process (GO:0006206)	7/11	0.013689615
Tetrahydrofolate Metabolic Process (GO:0046653)	7/11	0.013689615
Negative Regulation Of Cell Migration (GO:0030336)	44/163	0.013887408
Nucleobase Catabolic Process (GO:0046113)	5/6	0.014094359
Positive Regulation Of Hormone Metabolic Process (GO:0032352)	5/6	0.014094359
Vascular Associated Smooth Muscle Cell Development (GO:0097084)	5/6	0.014094359
Postsynaptic Membrane Organization (GO:0001941)	9/17	0.014180336
Positive Regulation Of Cellular Component Organization (GO:0051130)	33/113	0.014180336
Microtubule Polymerization Or Depolymerization (GO:0031109)	12/27	0.014180336
Regulation Of Insulin Secretion Involved In Cellular Response To Glucose Stimulus (GO:0061178)	12/27	0.014180336
Ventricular Septum Morphogenesis (GO:0060412)	12/27	0.014180336
Regulation Of Mast Cell Degranulation (GO:0043304)	8/14	0.014269032
Regulation Of Neuron Projection Arborization (GO:0150011)	8/14	0.014269032
Serine Family Amino Acid Metabolic Process (GO:0009069)	8/14	0.014269032
Positive Regulation Of Protein Tyrosine Kinase Activity (GO:0061098)	15/38	0.014479904
Protein Localization To Membrane (GO:0072657)	50/192	0.014490685
Positive Regulation Of Protein Polymerization (GO:0032273)	23/70	0.014625039
Regulation Of Actin Filament-Based Process (GO:0032970)	23/70	0.014625039
Regulation Of Tumor Necrosis Factor Production (GO:0032680)	36/127	0.014736366
Regulation Of Canonical Wnt Signaling Pathway (GO:0060828)	53/207	0.015470669
Negative Regulation Of Cell Projection Organization (GO:0031345)	18/50	0.015830644
Regulation Of Exocytosis (GO:0017157)	19/54	0.015830644
Actin Filament Polymerization (GO:0030041)	13/31	0.015830644
Negative Regulation Of Chemotaxis (GO:0050922)	13/31	0.015830644
Regulation Of Cell Morphogenesis (GO:0022604)	13/31	0.015830644
Regulation Of Protein Autophosphorylation (GO:0031952)	13/31	0.015830644
Positive Regulation Of Cytoskeleton Organization (GO:0051495)	25/79	0.015884321
Gliogenesis (GO:0042063)	11/24	0.01610784
Cell-Cell Junction Assembly (GO:0007043)	23/71	0.01738941
Adherens Junction Organization (GO:0034332)	14/35	0.01738941
Regulation Of Postsynaptic Membrane Potential (GO:0060078)	14/35	0.01738941
Positive Regulation Of Cell Migration (GO:0030335)	66/272	0.017640729
Positive Regulation Of Cell Growth (GO:0030307)	31/106	0.017928796
Anterograde Axonal Transport (GO:0008089)	15/39	0.018423878
Regulation Of Protein Tyrosine Kinase Activity (GO:0061097)	15/39	0.018423878
Inorganic Anion Transmembrane Transport (GO:0098661)	21/63	0.01842666
Cellular Response To Organic Cyclic Compound (GO:0071407)	41/152	0.01842666
Negative Regulation Of Actin Filament Bundle Assembly (GO:0032232)	12/28	0.018816794
Regulation Of Plasma Membrane Bounded Cell Projection Organization (GO:0120035)	12/28	0.018816794
Positive Regulation Of Biomineral Tissue Development (GO:0070169)	16/43	0.018816794
Positive Regulation Of Stress Fiber Assembly (GO:0051496)	16/43	0.018816794
Insulin Receptor Signaling Pathway (GO:0008286)	17/47	0.019144989
Sodium Ion Transport (GO:0006814)	27/89	0.019526827
Regulation Of Cellular Component Organization (GO:0051128)	41/153	0.020463493
Calcium Ion Transport Into Cytosol (GO:0060402)	6/9	0.020463493
Negative Regulation Of Protein Autophosphorylation (GO:0031953)	6/9	0.020463493
Positive Regulation Of Sodium Ion Transmembrane Transport (GO:1902307)	6/9	0.020463493
Postsynapse Assembly (GO:0099068)	6/9	0.020463493
Associative Learning (GO:0008306)	9/18	0.020463493
Neuron Fate Commitment (GO:0048663)	9/18	0.020463493
Vesicle-Mediated Transport In Synapse (GO:0099003)	9/18	0.020463493
Skin Development (GO:0043588)	22/68	0.020933025
Actin Filament Organization (GO:0007015)	39/144	0.02094694

Positive Regulation Of Interferon-Beta Production (GO:0032728)	14/36	0.02213453
Negative Regulation Of Stress Fiber Assembly (GO:0051497)	11/25	0.02213453
Dendritic Spine Morphogenesis (GO:0060997)	8/15	0.022206167
Retrograde Axonal Transport (GO:0008090)	8/15	0.022206167
Axonal Fasciculation (GO:0007413)	7/12	0.022284429
Dendrite Self-Avoidance (GO:0070593)	7/12	0.022284429
Positive Regulation Of Sodium Ion Transmembrane Transporter Activity (GO:2000651)	7/12	0.022284429
Positive Regulation Of Sodium Ion Transport (GO:0010765)	7/12	0.022284429
Regulation Of Vascular Associated Smooth Muscle Cell Migration (GO:1904752)	7/12	0.022284429
Spinal Cord Development (GO:0021510)	7/12	0.022284429
Cellular Response To Insulin Stimulus (GO:0032869)	29/99	0.022359585
Notch Signaling Pathway (GO:0007219)	15/40	0.022359585
Regulation Of Cation Channel Activity (GO:2001257)	19/56	0.022359585
Regulation Of Cell-Substrate Adhesion (GO:0010810)	16/44	0.022690368
Response To Unfolded Protein (GO:0006986)	16/44	0.022690368
Synaptic Vesicle Recycling (GO:0036465)	16/44	0.022690368
Cytoplasmic Microtubule Organization (GO:0031122)	17/48	0.022700371
Supramolecular Fiber Organization (GO:0097435)	74/316	0.022700371
Positive Regulation Of Secretion By Cell (GO:1903532)	23/73	0.022826167
Regulation Of Rho Protein Signal Transduction (GO:0035023)	23/73	0.022826167
Alpha-Amino Acid Metabolic Process (GO:1901605)	12/29	0.024047627
Regulation Of Developmental Growth (GO:0048638)	12/29	0.024047627
Regulation Of Neuron Migration (GO:2001222)	12/29	0.024047627
Cellular Response To Growth Factor Stimulus (GO:0071363)	41/155	0.02412611
Regulation Of Dopamine Secretion (GO:0014059)	10/22	0.025127092
Positive Regulation Of Developmental Process (GO:0051094)	57/233	0.025955921
Lung Development (GO:0030324)	13/33	0.025955921
Positive Regulation Of Interleukin-12 Production (GO:0032735)	13/33	0.025955921
Regulation Of Cell Adhesion Mediated By Integrin (GO:0033628)	13/33	0.025955921
Positive Regulation Of Epithelial Cell Proliferation (GO:0050679)	34/123	0.025955921
Regulation Of Insulin Secretion (GO:0050796)	26/87	0.026447655
Gland Development (GO:0048732)	23/74	0.026809006
Cellular Response To Organonitrogen Compound (GO:0071417)	30/105	0.026809006
Negative Regulation Of Endothelial Cell Proliferation (GO:0001937)	14/37	0.027052381
Blood Vessel Morphogenesis (GO:0048514)	18/53	0.027052381
Glycoprotein Metabolic Process (GO:0009100)	18/53	0.027052381
Negative Regulation Of Viral Genome Replication (GO:0045071)	18/53	0.027052381
Heart Contraction (GO:0060047)	15/41	0.027544986
Negative Regulation Of Epithelial Cell Proliferation (GO:0050680)	22/70	0.027891949
Sensory Organ Development (GO:0007423)	22/70	0.027891949
Heart Development (GO:0007507)	44/171	0.028406946
Negative Regulation Of Endothelial Cell Apoptotic Process (GO:2000352)	11/26	0.028406946
Positive Regulation Of Microtubule Polymerization Or Depolymerization (GO:0031112)	11/26	0.028406946
Embryonic Digestive Tract Development (GO:0048566)	9/19	0.028406946
Ganglioside Metabolic Process (GO:0001573)	9/19	0.028406946
Negative Regulation Of Cellular Process (GO:0048523)	116/537	0.028709507
Positive Regulation Of Endocytosis (GO:0045807)	24/79	0.029139749
Foam Cell Differentiation (GO:0090077)	5/7	0.029261254
Lens Fiber Cell Development (GO:0070307)	5/7	0.029261254
Negative Regulation Of Glial Cell Apoptotic Process (GO:0034351)	5/7	0.029261254
Positive Regulation Of Cardiac Epithelial To Mesenchymal Transition (GO:0062043)	5/7	0.029261254
Positive Regulation Of Melanin Biosynthetic Process (GO:0048023)	5/7	0.029261254
Positive Regulation Of Microtubule Nucleation (GO:0090063)	5/7	0.029261254
Positive Regulation Of Secondary Metabolite Biosynthetic Process (GO:1900378)	5/7	0.029261254
Postsynaptic Density Organization (GO:0097106)	5/7	0.029261254
Regulation Of Chaperone-Mediated Autophagy (GO:1904714)	5/7	0.029261254
Regulation Of Leukocyte Degranulation (GO:0043300)	5/7	0.029261254
Regulation Of Mesenchymal Stem Cell Differentiation (GO:2000739)	5/7	0.029261254
Ventricular Trabecula Myocardium Morphogenesis (GO:0003222)	5/7	0.029261254
Negative Regulation Of Kinase Activity (GO:0033673)	20/62	0.029261254
Phosphatidylinositol-Mediated Signaling (GO:0048015)	20/62	0.029261254
Cellular Response To Vascular Endothelial Growth Factor Stimulus (GO:0035924)	13/34	0.032288477
Positive Regulation Of Bone Mineralization (GO:0030501)	14/38	0.032846841
Regulation Of Neurotransmitter Secretion (GO:0046928)	14/38	0.032846841
T Cell Differentiation (GO:0030217)	16/46	0.032846841
Regulation Of Nervous System Development (GO:0051960)	16/46	0.032846841
Negative Regulation Of Protein Serine/Threonine Kinase Activity (GO:0071901)	24/80	0.032846841
Monoatomic Cation Transport (GO:0006812)	15/42	0.032846841
Potassium Ion Import Across Plasma Membrane (GO:1990573)	15/42	0.032846841
Axon Extension (GO:0048675)	10/23	0.032846841
Heparan Sulfate Proteoglycan Biosynthetic Process (GO:0015012)	10/23	0.032846841
Regulation Of Axon Extension Involved In Axon Guidance (GO:0048841)	10/23	0.032846841

Negative Regulation Of Cell Differentiation (GO:0045596)	51/207	0.032899887
Regulation Of Neurogenesis (GO:0050767)	21/67	0.032899887
Membrane Depolarization (GO:0051899)	7/13	0.03336488
Positive Regulation Of Superoxide Anion Generation (GO:0032930)	7/13	0.03336488
Regulation Of Short-Term Neuronal Synaptic Plasticity (GO:0048172)	7/13	0.03336488
Positive Regulation Of Cell Population Proliferation (GO:0008284)	105/483	0.03336488
Hematopoietic Stem Cell Proliferation (GO:0071425)	6/10	0.03336488
Heparan Sulfate Proteoglycan Biosynthetic Process, Enzymatic Modification (GO:0015015)	6/10	0.03336488
Neuron Projection Fasciculation (GO:0106030)	6/10	0.03336488
Positive Regulation Of Chondrocyte Differentiation (GO:0032332)	6/10	0.03336488
Positive Regulation Of Macrophage Differentiation (GO:0045651)	6/10	0.03336488
Positive Regulation Of Protein Depolymerization (GO:1901881)	6/10	0.03336488
Regulation Of Microtubule Nucleation (GO:0010968)	6/10	0.03336488
Calcium Ion Transport (GO:0006816)	31/112	0.033557539
Small GTPase Mediated Signal Transduction (GO:0007264)	31/112	0.033557539
Integrin-Mediated Signaling Pathway (GO:0007229)	25/85	0.034526814
Positive Regulation Of Protein Localization (GO:1903829)	25/85	0.034526814
Negative Regulation Of Neurogenesis (GO:0050768)	11/27	0.035689999
Positive Regulation Of Mitotic Cell Cycle (GO:0045931)	11/27	0.035689999
Regulation Of Peptidyl-Tyrosine Phosphorylation (GO:0050730)	26/90	0.037464753
Negative Regulation Of Programmed Cell Death (GO:0043069)	85/381	0.037633722
Presynaptic Endocytosis (GO:0140238)	12/31	0.037633722
Regulation Of Extrinsic Apoptotic Signaling Pathway Via Death Domain Receptors (GO:1902041)	12/31	0.037633722
Regulation Of Long-Term Synaptic Potentiation (GO:1900271)	12/31	0.037633722
Fc-gamma Receptor Signaling Pathway Involved In Phagocytosis (GO:0038096)	9/20	0.037633722
Peripheral Nervous System Development (GO:0007422)	9/20	0.037633722
Positive Regulation Of Vascular Associated Smooth Muscle Cell Proliferation (GO:1904707)	9/20	0.037633722
Regulation Of Calcium Ion Transmembrane Transport (GO:1903169)	9/20	0.037633722
Regulation Of Membrane Depolarization (GO:0003254)	9/20	0.037633722
Regulation Of Cold-Induced Thermogenesis (GO:0120161)	38/146	0.037994261
Positive Regulation Of Smooth Muscle Cell Proliferation (GO:0048661)	16/47	0.038403961
Regulation Of Wound Healing (GO:0061041)	16/47	0.038403961
Embryonic Organ Development (GO:0048568)	23/77	0.038730115
Regulation Of Secretion By Cell (GO:1903530)	20/64	0.039388012
Regulation Of Interleukin-6 Production (GO:0032675)	31/114	0.042169224
Negative Regulation Of Lipoprotein Lipase Activity (GO:0051005)	4/5	0.042169224
Neurotransmitter-Gated Ion Channel Clustering (GO:0072578)	4/5	0.042169224
Regulation Of Blood Vessel Remodeling (GO:0060312)	4/5	0.042169224
Regulation Of Glomerular Filtration (GO:0003093)	4/5	0.042169224
Regulation Of Voltage-Gated Sodium Channel Activity (GO:1905150)	4/5	0.042169224
Retinal Ganglion Cell Axon Guidance (GO:0031290)	4/5	0.042169224
Regulation Of Supramolecular Fiber Organization (GO:1902903)	10/24	0.042884277
Regulation Of Protein Kinase Activity (GO:0045859)	32/119	0.043742378
Central Nervous System Neuron Development (GO:0021954)	8/17	0.043742378
Filopodium Assembly (GO:0046847)	8/17	0.043742378
Neuron Maturation (GO:0042551)	8/17	0.043742378
Regulation Of Cartilage Development (GO:0061035)	8/17	0.043742378
Positive Regulation Of Cell Motility (GO:2000147)	53/221	0.044564902
Negative Regulation Of Cytoskeleton Organization (GO:0051494)	11/28	0.046172397
Negative Regulation Of Mitotic Metaphase/Anaphase Transition (GO:0045841)	11/28	0.046172397
Cellular Response To Mechanical Stimulus (GO:0071260)	16/48	0.046279396
Chloride Transmembrane Transport (GO:1902476)	16/48	0.046279396
Oligosaccharide Metabolic Process (GO:0009311)	16/48	0.046279396
Neural Crest Cell Development (GO:0014032)	15/44	0.047697056
Mesenchymal Cell Migration (GO:0090497)	12/32	0.047771503
Neural Tube Closure (GO:0001843)	12/32	0.047771503
Aortic Valve Development (GO:0003176)	14/40	0.048321522
Neural Crest Cell Migration (GO:0001755)	13/36	0.048400681
2-Oxoglutarate Metabolic Process (GO:0006103)	7/14	0.049550844
Calcium Ion-Regulated Exocytosis Of Neurotransmitter (GO:0048791)	7/14	0.049550844
Mammary Gland Epithelium Development (GO:0061180)	7/14	0.049550844
Protein Homotrimerization (GO:0070207)	7/14	0.049550844
Protein Trimerization (GO:0070206)	7/14	0.049550844
Regulation Of Superoxide Anion Generation (GO:0032928)	7/14	0.049550844

Gene Ontology: Cellular Component	Overlap	Adjusted P-value
Neuron Projection (GO:0043005)	220/557	7.45E-36
Axon (GO:0030424)	92/205	6.54E-19
Dendrite (GO:0030425)	107/270	2.19E-17
Postsynaptic Density (GO:0014069)	73/151	2.19E-17
Glutamatergic Synapse (GO:0098978)	42/78	6.60E-12
Asymmetric Synapse (GO:0032279)	58/133	2.04E-11

Cell-Cell Junction (GO:0005911)	99/299	1.69E-10
Postsynaptic Specialization Membrane (GO:0099634)	25/38	1.07E-09
Postsynaptic Density Membrane (GO:0098839)	22/34	2.50E-08
Adherens Junction (GO:0005912)	56/150	4.52E-08
Ionotropic Glutamate Receptor Complex (GO:0008328)	21/33	7.97E-08
Potassium Channel Complex (GO:0034705)	36/81	1.65E-07
Cytoskeleton (GO:0005856)	150/599	3.60E-06
Polymeric Cytoskeletal Fiber (GO:0099513)	78/265	5.34E-06
Collagen-Containing Extracellular Matrix (GO:0062023)	100/373	1.54E-05
Microtubule (GO:0005874)	58/185	1.78E-05
Intercalated Disc (GO:0014704)	17/31	3.41E-05
Synaptic Membrane (GO:0097060)	17/31	3.41E-05
Axonal Growth Cone (GO:0044295)	11/15	4.29E-05
Voltage-Gated Potassium Channel Complex (GO:0008076)	29/73	5.15E-05
Basolateral Plasma Membrane (GO:0016323)	53/171	5.86E-05
GABA-ergic Synapse (GO:0098982)	12/19	1.36E-04
Actin Cytoskeleton (GO:0015629)	86/327	1.36E-04
Catenin Complex (GO:0016342)	15/28	1.56E-04
Voltage-Gated Calcium Channel Complex (GO:0005891)	21/49	2.46E-04
Cell-Cell Contact Zone (GO:0044291)	20/47	4.26E-04
Dendrite Membrane (GO:0032590)	14/28	7.61E-04
Extrinsic Component Of Cytoplasmic Side Of Plasma Membrane (GO:0031234)	27/77	0.001110019
Microtubule Cytoskeleton (GO:0015630)	85/342	0.001117671
Astrocyte Projection (GO:0097449)	6/7	0.001759785
Sodium Channel Complex (GO:0034706)	10/18	0.002582891
Exocytic Vesicle Membrane (GO:0099501)	20/53	0.002582891
Synaptic Vesicle Membrane (GO:0030672)	20/54	0.003356214
Glial Cell Projection (GO:0097386)	8/13	0.00418177
AMPA Glutamate Receptor Complex (GO:0032281)	10/19	0.00418177
Cytoplasmic Side Of Plasma Membrane (GO:0009898)	36/124	0.005082207
Filopodium (GO:0030175)	21/60	0.005082207
Cell-Substrate Junction (GO:0030055)	92/395	0.005082207
Focal Adhesion (GO:0005925)	90/387	0.005963997
Excitatory Synapse (GO:0060076)	11/24	0.008466731
Actin Filament (GO:0005884)	23/71	0.008995913
Apical Junction Complex (GO:0043296)	31/106	0.009147719
Tight Junction (GO:0070160)	28/93	0.009376556
Calcium Channel Complex (GO:0034704)	20/59	0.009376556
Specific Granule Membrane (GO:0035579)	27/90	0.011615707
Lysosomal Lumen (GO:0043202)	26/86	0.012220641
Membrane Raft (GO:0045121)	44/169	0.012470236
NMDA Selective Glutamate Receptor Complex (GO:0017146)	5/7	0.016682313
Voltage-Gated Sodium Channel Complex (GO:0001518)	5/7	0.016682313
Dense Core Granule (GO:0031045)	8/16	0.017718879
Bicellular Tight Junction (GO:0005923)	24/81	0.02216378
Vacuolar Lumen (GO:0005775)	41/161	0.024707863
Specific Granule (GO:0042581)	40/159	0.033863513
Inhibitory Synapse (GO:0060077)	5/8	0.034149803
Perineuronal Net (GO:0072534)	5/8	0.034149803
Cortical Cytoskeleton (GO:0030863)	19/63	0.042851901
G Protein-Coupled Receptor Dimeric Complex (GO:0038037)	4/6	0.061766738
Apical Dendrite (GO:0097440)	5/9	0.061766738
Perisynaptic Extracellular Matrix (GO:0098966)	5/9	0.061766738
Endocytic Vesicle Membrane (GO:0030666)	38/159	0.08672085
Plasma Membrane Raft (GO:0044853)	22/82	0.093992712
Secretory Granule Membrane (GO:0030667)	61/279	0.099844327
Neuron To Neuron Synapse (GO:0098984)	5/10	0.099844327
Clathrin-Sculpted Gamma-Aminobutyric Acid Transport Vesicle (GO:0061200)	4/7	0.106636349
Clathrin-Sculpted Gamma-Aminobutyric Acid Transport Vesicle Membrane (GO:0061202)	4/7	0.106636349
Cytoskeleton Of Presynaptic Active Zone (GO:0048788)	4/7	0.106636349
Dendritic Spine Membrane (GO:0032591)	4/7	0.106636349
Actin-Based Cell Projection (GO:0098858)	22/84	0.111178863
Vesicle (GO:0031982)	52/235	0.111189187
Platelet Alpha Granule (GO:0031091)	23/89	0.111189187
Cytoplasmic Vesicle Membrane (GO:0030659)	81/389	0.114595519
Spindle (GO:0005819)	47/210	0.115678614
Preribosome, Large Subunit Precursor (GO:0030687)	7/18	0.115678614
Azurophil Granule (GO:0042582)	36/155	0.12457155
Bounding Membrane Of Organelle (GO:0098588)	159/819	0.12457155
Intracellular Vesicle (GO:0097708)	5/11	0.131064086
Dendrite Cytoplasm (GO:0032839)	4/8	0.161182369
Mitotic Spindle (GO:0072686)	33/143	0.161281709

Vesicle Membrane (GO:0012506)	18/69	0.165892524
Basement Membrane (GO:0005604)	13/46	0.172396392
Activin Receptor Complex (GO:0048179)	3/5	0.172396392
Extrinsic Component Of Endoplasmic Reticulum Membrane (GO:0042406)	3/5	0.172396392
Neuronal Dense Core Vesicle (GO:0098992)	3/5	0.172396392
P Granule (GO:0043186)	8/24	0.172812906
Platelet Alpha Granule Membrane (GO:0031092)	6/16	0.181081028
Golgi Lumen (GO:0005796)	24/100	0.185016151
SWI/SNF Complex (GO:0016514)	9/29	0.194799036
Sarcolemma (GO:0042383)	14/52	0.19769335
Extracellular Membrane-Bounded Organelle (GO:0065010)	15/57	0.202851721
Caveola (GO:0005901)	16/62	0.206298227
bBAF Complex (GO:0140092)	4/9	0.211135833
Pi-Body (GO:0071546)	4/9	0.211135833

Gene Ontology: Molecular Function	Overlap	Adjusted P-value
Tubulin Binding (GO:0015631)	97/322	1.69E-06
Microtubule Binding (GO:0008017)	75/239	5.55E-06
High Voltage-Gated Calcium Channel Activity (GO:0008331)	16/23	5.55E-06
Transmitter-Gated Monoatomic Ion Channel Activity Involved In Regulation Of Postsynaptic Membrane Potential (GO:1904315)	20/34	5.55E-06
Transmitter-Gated Monoatomic Ion Channel Activity (GO:0022824)	22/40	5.55E-06
Voltage-Gated Monoatomic Cation Channel Activity (GO:0022843)	33/76	5.55E-06
Neurotransmitter Receptor Activity Involved In Regulation Of Postsynaptic Membrane Potential (GO:0099529)	15/21	5.55E-06
Calcium Ion Binding (GO:0005509)	98/346	5.55E-06
Metal Ion Binding (GO:0046872)	135/521	6.76E-06
GTPase Regulator Activity (GO:0030695)	114/424	8.18E-06
Voltage-Gated Calcium Channel Activity (GO:0005245)	23/47	3.13E-05
Ionotropic Glutamate Receptor Activity (GO:0004970)	12/16	3.76E-05
Potassium Channel Activity (GO:0005267)	30/79	3.99E-04
Voltage-Gated Potassium Channel Activity (GO:0005249)	30/80	4.74E-04
Sodium Channel Activity (GO:0005272)	18/37	4.74E-04
Voltage-Gated Sodium Channel Activity (GO:0005248)	12/19	4.74E-04
Transmembrane Receptor Protein Tyrosine Kinase Activity (GO:0004714)	21/50	0.001313639
Protein Serine/Threonine Kinase Activity (GO:0004674)	87/342	0.001512139
Chemorepellent Activity (GO:0045499)	13/24	0.0016719
Sequence-Specific Double-Stranded DNA Binding (GO:1990837)	161/715	0.0016719
Guanyl-Nucleotide Exchange Factor Activity (GO:0005085)	57/203	0.001701973
Protein Tyrosine Kinase Activity (GO:0004713)	33/99	0.001925978
Protein Homodimerization Activity (GO:0042803)	150/662	0.001925978
Cell Adhesion Mediator Activity (GO:0098631)	14/28	0.002138296
GTPase Activator Activity (GO:0005096)	58/211	0.002375149
Transcription Regulatory Region Nucleic Acid Binding (GO:0001067)	60/224	0.003833742
Ligand-Gated Monoatomic Ion Channel Activity (GO:0015276)	12/23	0.003878938
Actin Binding (GO:0003779)	51/183	0.003930713
alpha-N-acetylneuraminate Alpha-2,8-Sialyltransferase Activity (GO:0003828)	5/5	0.004253243
Dihydropyrimidinase Activity (GO:0004157)	5/5	0.004253243
Neurotrophin Binding (GO:0043121)	6/7	0.004253243
Semaphorin Receptor Activity (GO:0017154)	7/10	0.008602062
Semaphorin Receptor Binding (GO:0030215)	11/22	0.010047789
Heparan Sulfate Sulfotransferase Activity (GO:0034483)	8/13	0.010477976
SH3 Domain Binding (GO:0017124)	21/59	0.010587478
Protein Tyrosine Phosphatase Activity (GO:0004725)	25/76	0.01209535
Sialyltransferase Activity (GO:0008373)	10/20	0.016795616
Sodium Channel Regulator Activity (GO:0017080)	14/34	0.01721948
Amyloid-Beta Binding (GO:0001540)	25/79	0.021304433
Sequence-Specific DNA Binding (GO:0043565)	152/717	0.024231185
Phosphotyrosine Residue Binding (GO:0001784)	15/39	0.024231185
Cell-Cell Adhesion Mediator Activity (GO:0098632)	16/43	0.024637885
Calcium-Dependent Phospholipid Binding (GO:0005544)	17/47	0.024637885
Glutamate Receptor Binding (GO:0035254)	9/18	0.025990814
Ligand-Gated Monoatomic Anion Channel Activity (GO:0099095)	9/18	0.025990814
Insulin-Like Growth Factor Binding (GO:0005520)	8/15	0.02817603
Protein Kinase A Catalytic Subunit Binding (GO:0034236)	7/12	0.02824827
Double-Stranded DNA Binding (GO:0003690)	138/650	0.032399824
Potassium Channel Regulator Activity (GO:0015459)	16/45	0.035965522
Cadherin Binding (GO:0045296)	74/319	0.035965522
Adenyl Ribonucleotide Binding (GO:0032559)	72/309	0.035965522
Inward Rectifier Potassium Channel Activity (GO:0005242)	11/26	0.036337748
NMDA Glutamate Receptor Activity (GO:0004972)	5/7	0.037560835
PDZ Domain Binding (GO:0030165)	20/62	0.037560835
Ligand-Gated Monoatomic Cation Channel Activity (GO:0099094)	23/75	0.037560835
Glutamate Receptor Activity (GO:0008066)	8/16	0.037560835

Transmembrane Receptor Protein Phosphatase Activity (GO:0019198)	8/16	0.037560835
Transmembrane Receptor Protein Tyrosine Phosphatase Activity (GO:0005001)	8/16	0.037560835
Phosphatidylinositol Bisphosphate Binding (GO:1902936)	27/93	0.037560835
Ligand-Gated Monoatomic Ion Channel Activity Involved In Regulation Of Presynaptic Membrane Potential (GO:0099507)	6/10	0.041553368
Voltage-Gated Monoatomic Ion Channel Activity (GO:0005244)	11/27	0.044429429
Kinesin Binding (GO:0019894)	12/31	0.046319835
Pyridoxal Phosphate Binding (GO:0030170)	9/20	0.046319835
ATP Binding (GO:0005524)	65/279	0.046319835
Tau Protein Binding (GO:0048156)	14/39	0.047648382
Secondary Active Transmembrane Transporter Activity (GO:0015291)	19/60	0.050083014
C3HC4-type RING Finger Domain Binding (GO:0055131)	4/5	0.050590628
Transmembrane-Ephrin Receptor Activity (GO:0005005)	7/14	0.061957007
GABA Receptor Activity (GO:0016917)	9/21	0.063442869
Cuprous Ion Binding (GO:1903136)	5/8	0.064502856
Tropomyosin Binding (GO:0005523)	5/8	0.064502856
UDP-galactose:beta-N-acetylglucosamine Beta-1,3-Galactosyltransferase Activity (GO:0008499)	6/11	0.064502856
Ionotropic Glutamate Receptor Binding (GO:0035255)	6/11	0.064502856
Protein Phosphorylated Amino Acid Binding (GO:0045309)	15/45	0.068014454
Transmembrane Receptor Protein Kinase Activity (GO:0019199)	13/37	0.070781714
Protein Kinase Binding (GO:0019901)	108/511	0.071361611
L-amino Acid Transmembrane Transporter Activity (GO:0015179)	19/63	0.077833847
Kinase Binding (GO:0019900)	98/460	0.079146451
Ephrin Receptor Activity (GO:0005003)	7/15	0.083806859
Sodium-Independent Organic Anion Transmembrane Transporter Activity (GO:0015347)	7/15	0.083806859
NAD Binding (GO:0051287)	10/26	0.083806859
Phosphatidylinositol-4,5-Bisphosphate Binding (GO:0005546)	21/73	0.088473824
E-box Binding (GO:0070888)	16/51	0.089301729
GABA-gated Chloride Ion Channel Activity (GO:0022851)	6/12	0.093142452
Hydrolase Activity, Acting On Carbon-Nitrogen (But Not Peptide) Bonds, In Cyclic Amides (GO:0016812)	6/12	0.093142452
Insulin-Like Growth Factor I Binding (GO:0031994)	6/12	0.093142452
Microtubule Plus-End Binding (GO:0051010)	8/19	0.093142452
1-Alkyl-2-Acetylglycerophosphocholine Esterase Activity (GO:0003847)	4/6	0.093828775
Minor Groove Of Adenine-Thymine-Rich DNA Binding (GO:0003680)	4/6	0.093828775
Inorganic Cation Transmembrane Transporter Activity (GO:0022890)	12/35	0.093828775
DNA Binding (GO:0003677)	168/846	0.093828775
MAP Kinase Tyrosine Phosphatase Activity (GO:0033550)	5/9	0.093828775
MAP Kinase Tyrosine/Serine/Threonine Phosphatase Activity (GO:0017017)	5/9	0.093828775
Aromatic Amino Acid Transmembrane Transporter Activity (GO:0015173)	5/9	0.093828775
Glycine Binding (GO:0016594)	5/9	0.093828775
Phospholipase Inhibitor Activity (GO:0004859)	5/9	0.093828775
Protein Tyrosine/Threonine Phosphatase Activity (GO:0008330)	5/9	0.093828775
Manganese Ion Binding (GO:0030145)	15/48	0.099248493
Acetylglucosaminyltransferase Activity (GO:0008375)	13/40	0.109856112
Monoatomic Ion Gated Channel Activity (GO:0022839)	13/40	0.109856112
Purine Ribonucleoside Triphosphate Binding (GO:0035639)	99/476	0.116187133
Monocarboxylic Acid Transmembrane Transporter Activity (GO:0008028)	11/32	0.117214268
Inhibitory Extracellular Ligand-Gated Monoatomic Ion Channel Activity (GO:0005237)	6/13	0.124241351
Monoatomic Cation Channel Activity (GO:0005261)	27/105	0.124241351
Calcium Channel Activity (GO:0005262)	24/91	0.128283641
Transcription Cis-Regulatory Region Binding (GO:0000976)	98/474	0.133917141
Calcium Channel Regulator Activity (GO:0005246)	12/37	0.134426037
Monoatomic Cation Transmembrane Transporter Activity (GO:0008324)	12/37	0.134426037
DNA Secondary Structure Binding (GO:0000217)	11/33	0.140126822
L-arginine Transmembrane Transporter Activity (GO:0061459)	5/10	0.140264261
Dipeptidyl-Peptidase Activity (GO:0008239)	5/10	0.140264261
Filamin Binding (GO:0031005)	5/10	0.140264261
Glycogen Binding (GO:2001069)	4/7	0.156522337
Hydroxymethyl-, Formyl- And Related Transferase Activity (GO:0016742)	4/7	0.156522337
Alpha-Tubulin Binding (GO:0043014)	12/38	0.157013084
Amino Acid Transmembrane Transporter Activity (GO:0015171)	16/56	0.162552718
C4-dicarboxylate Transmembrane Transporter Activity (GO:0015556)	6/14	0.163365134
Beta-1,3-Galactosyltransferase Activity (GO:0048531)	6/14	0.163365134
Transaminase Activity (GO:0008483)	6/14	0.163365134
Ankyrin Binding (GO:0030506)	7/18	0.170197712
Calcium Activated Cation Channel Activity (GO:0005227)	7/18	0.170197712
Hydrolase Activity, Hydrolyzing N-glycosyl Compounds (GO:0016799)	7/18	0.170197712
NAD+ ADP-ribosyltransferase Activity (GO:0003950)	9/26	0.170197712
Protein Serine/Threonine/Tyrosine Kinase Activity (GO:0004712)	9/26	0.170197712
Protein Heterodimerization Activity (GO:0046982)	43/190	0.175036607
Pentosyltransferase Activity (GO:0016763)	12/39	0.17620913
Calcium-Activated Potassium Channel Activity (GO:0015269)	5/11	0.195882068
Adenyl Nucleotide Binding (GO:0030554)	10/31	0.198208263

Potassium Ion Transmembrane Transporter Activity (GO:0015079)	10/31	0.198208263
Alpha-Actinin Binding (GO:0051393)	8/23	0.213658846
Calmodulin-Dependent Protein Kinase Activity (GO:0004683)	8/23	0.213658846
mRNA 3'-UTR AU-rich Region Binding (GO:0035925)	8/23	0.213658846
UDP-galactosyltransferase Activity (GO:0035250)	7/19	0.21553058
Amino Acid Binding (GO:0016597)	11/36	0.217463283
Non-Membrane Spanning Protein Tyrosine Kinase Activity (GO:0004715)	11/36	0.217463283
Small GTPase Binding (GO:0031267)	44/199	0.219204841
Carboxylic Acid Transmembrane Transporter Activity (GO:0046943)	16/59	0.219831667
L-aspartate Transmembrane Transporter Activity (GO:0015183)	4/8	0.219831667
Chloride:Monoatomic Cation Symporter Activity (GO:0015377)	4/8	0.219831667
Leucine Zipper Domain Binding (GO:0043522)	4/8	0.219831667
Type II Transforming Growth Factor Beta Receptor Binding (GO:0005114)	4/8	0.219831667
RNA Endonuclease Activity (GO:0004521)	12/41	0.229079352
Glutathione Transferase Activity (GO:0004364)	9/28	0.238543455
Monoatomic Ion Channel Activity (GO:0005216)	11/37	0.239839717
ATP-activated Inward Rectifier Potassium Channel Activity (GO:0015272)	3/5	0.239839717
[Heparan Sulfate]-Glucosamine 3-Sulfotransferase 1 Activity (GO:0008467)	3/5	0.239839717
arachidonate-CoA Ligase Activity (GO:0047676)	3/5	0.239839717
Axon Guidance Receptor Activity (GO:0008046)	3/5	0.239839717
Lysophosphatidic Acid Phosphatase Activity (GO:0052642)	3/5	0.239839717
Magnesium Ion Binding (GO:0000287)	35/155	0.240966952
LRR Domain Binding (GO:0030275)	5/12	0.240966952
Ligand-Gated Channel Activity (GO:0022834)	5/12	0.240966952
Platelet-Derived Growth Factor Receptor Binding (GO:0005161)	5/12	0.240966952
Tau-Protein Kinase Activity (GO:0050321)	7/20	0.245985473
Syntaxin-1 Binding (GO:0017075)	6/16	0.248005484
Protein Phosphatase Binding (GO:0019903)	27/115	0.253131773
Carboxypeptidase Activity (GO:0004180)	11/38	0.27259719
Kinase Activity (GO:0016301)	25/106	0.278382606
1-Phosphatidylinositol-3-Kinase Activity (GO:0016303)	4/9	0.292242107
JUN Kinase Binding (GO:0008432)	4/9	0.292242107
Benzodiazepine Receptor Activity (GO:0008503)	4/9	0.292242107
Calcium:Sodium Antiporter Activity (GO:0005432)	4/9	0.292242107
Glutathione Binding (GO:0043295)	4/9	0.292242107
Hydrolase Activity, Acting On Carbon-Nitrogen (But Not Peptide) Bonds, In Linear Amidines (GO:0016813)	4/9	0.292242107
Phosphatidylinositol-3,5-Bisphosphate 3-Phosphatase Activity (GO:0052629)	4/9	0.292242107
Protein Binding Involved In Heterotypic Cell-Cell Adhesion (GO:0086080)	4/9	0.292242107
Lipid Kinase Activity (GO:0001727)	7/21	0.292460546

Supplementary Table 11. Different categories of genes identified through comparisons of their transcript levels before and after doxycycline treatment of the *Tet/Tet* neurons

Genes of category I

	Untreated Tet/Tet Neurons			Doxycycline-treated *Tet/Tet* Neurons		
Gene	*p* -value	Log_2 Fold change	Q-value (Benjamini-Hochberg)	*p* -value	Log_2 Fold change	Q-value (Benjamini-Hochberg)
Abcg5	0.0000	4.7788	0.0283	0.5950	-0.4919	0.0894
Abhd2	0.0000	3.8934	0.0001	0.0000	0.6358	0.0219
Ablim1	0.0000	-1.2731	0.0104	0.0000	-0.8899	0.0212
Acot1	0.0000	-2.1048	0.0049	0.0376	0.2410	0.0661
Acot11	0.0000	-1.1380	0.0389	0.3718	-0.1964	0.0829
Acsbg1	0.0000	-1.4204	0.0363	0.0020	-0.8054	0.0553
Acsl5	0.0000	1.2545	0.0265	0.0021	0.5538	0.0555
Actn4	0.0000	1.0082	0.0081	0.0000	0.8298	0.0141
Actr3	0.0000	1.1875	0.0035	0.0000	0.5089	0.0242
Adam12	0.0000	-2.0421	0.0154	0.0052	-0.5316	0.0582
Adamts1	0.0003	-1.2310	0.0487	0.0294	-0.7309	0.0649
Adamts10	0.0000	-1.0327	0.0256	0.0000	-0.6096	0.0442
Adamts12	0.0000	-2.0213	0.0073	0.3088	-0.1383	0.0808
Adamts17	0.0000	-1.0990	0.0453	0.0027	-0.7820	0.0562
Adamts2	0.0000	-2.4977	0.0067	0.8184	0.0348	0.0953
Adamts3	0.0000	2.8846	0.0028	0.0000	-0.7780	0.0418
Adamts5	0.0000	-1.5086	0.0298	0.0015	-0.7327	0.0544
Adcy1	0.0000	-1.0967	0.0192	0.4970	0.0824	0.0867
Adcyap1	0.0000	1.6583	0.0388	0.4234	0.2804	0.0845
Adk	0.0000	1.3144	0.0098	0.0000	0.8300	0.0245
Adora1	0.0000	-1.4942	0.0340	0.0091	-0.6609	0.0600
Adora2b	0.0000	-2.0291	0.0416	0.7068	0.1455	0.0926
Adra1a	0.0000	1.4333	0.0299	0.0004	-0.8771	0.0508
Adrm1	0.0000	1.5156	0.0046	0.0007	0.3158	0.0524
Agk	0.0000	2.0440	0.0059	0.0001	0.5298	0.0488
Ahcy	0.0000	5.5363	0.0001	0.0000	0.6992	0.0450
Ak9	0.0000	2.6233	0.0422	0.4360	0.4889	0.0848
Aldh1a3	0.0000	-4.3570	0.0177	0.0083	0.6958	0.0597
Alg8	0.0000	1.1072	0.0373	0.0729	0.3946	0.0697
Alpk1	0.0000	-1.7510	0.0405	0.9580	-0.0176	0.0990
Amd1	0.0000	1.6486	0.0029	0.0000	0.7039	0.0229
Amph	0.0000	-1.2492	0.0150	0.0000	-0.5155	0.0443
Anapc10	0.0000	1.0215	0.0232	0.0000	0.5846	0.0440
Angpt1	0.0000	-3.7219	0.0035	0.0000	-0.9806	0.0346
Angpt2	0.0000	-1.1273	0.0454	0.9962	0.0013	0.0999
Angpt4	0.0003	-2.9736	0.0496	0.4471	-0.4946	0.0852
Angptl1	0.0000	-3.6334	0.0212	0.0242	-0.7448	0.0640
Ankrd12	0.0000	-1.0355	0.0144	0.0000	-0.9816	0.0173
Ankrd34a	0.0003	-1.6110	0.0489	0.7282	0.1370	0.0931
Ankrd46	0.0000	-1.0953	0.0096	0.0000	-0.7802	0.0198
Anpep	0.0000	-1.9006	0.0121	0.3710	-0.1388	0.0828
Ap5s1	0.0000	-1.2506	0.0304	0.0000	-0.8406	0.0443
Apold1	0.0000	4.1074	0.0144	0.3959	0.3775	0.0837
Apoo	0.0000	2.7070	0.0075	0.0309	-0.4752	0.0652
Aqp1	0.0000	-2.2606	0.0227	0.0007	0.8635	0.0524
Arhgap18	0.0000	1.5487	0.0122	0.0093	0.3711	0.0601
Arhgap24	0.0000	-1.1488	0.0383	0.0482	-0.4426	0.0673
Arhgap29	0.0000	-1.7355	0.0054	0.0000	-0.9619	0.0200
Arhgap31	0.0000	-1.5281	0.0194	0.0001	-0.6554	0.0480
Arhgdig	0.0000	-1.0464	0.0397	0.0005	-0.7422	0.0515
Arhgef25	0.0000	-1.0123	0.0150	0.0000	-0.8891	0.0201
Arx	0.0000	-5.9717	0.0030	0.0000	-0.9064	0.0314
Asah2	0.0000	-1.9332	0.0305	0.4136	0.2172	0.0842
Asap3	0.0000	-1.2353	0.0294	0.3573	-0.1666	0.0824
Asic2	0.0000	-1.2244	0.0325	0.0000	-0.9880	0.0403
Aspm	0.0000	1.3283	0.0093	0.0000	0.5117	0.0404
Atp1a2	0.0000	-3.6448	0.0008	0.0000	0.4264	0.0445
Atp5e	0.0000	-1.1454	0.0153	0.0274	-0.2426	0.0646
Atp5g1	0.0000	2.1484	0.0031	0.0004	0.4129	0.0511
Atp6v0e2	0.0000	-1.2733	0.0091	0.0000	-0.9492	0.0175
Atxn1	0.0000	-1.4318	0.0281	0.0000	-0.8446	0.0463
Aven	0.0000	1.0221	0.0301	0.0000	0.7553	0.0421
Axl	0.0000	-1.3694	0.0125	0.0000	-0.7403	0.0327
B3gat2	0.0000	-1.0214	0.0411	0.0000	-0.9547	0.0442
B3gnt8	0.0003	-1.8108	0.0491	0.1962	0.5477	0.0767
B4galt4	0.0000	-1.3224	0.0168	0.0009	-0.4416	0.0529
B4galt7	0.0000	-1.1928	0.0169	0.0000	-0.8634	0.0276
Baalc	0.0000	-2.0051	0.0437	0.3121	-0.4224	0.0810
Bace2	0.0000	-3.9639	0.0161	0.0942	-0.5025	0.0713
Bcan	0.0000	-3.7566	0.0342	0.2527	0.7125	0.0789
Bicc1	0.0000	-1.4428	0.0202	0.0817	0.2860	0.0704
Bloc1s6	0.0000	-1.0781	0.0146	0.0000	-0.7269	0.0277

Bmf	0.0000	-1.1096	0.0166	0.1281	-0.1694	0.0734
Bmp2	0.0003	-1.3673	0.0488	0.6377	-0.1653	0.0906
Bmp6	0.0000	2.3652	0.0070	0.0000	-0.8365	0.0428
Bmper	0.0001	-1.1603	0.0460	0.0006	-0.9739	0.0521
Brca2	0.0000	1.0950	0.0145	0.0001	0.4344	0.0468
Brix1	0.0000	1.0213	0.0104	0.0000	0.9903	0.0127
Bsdc1	0.0000	-1.0367	0.0159	0.0000	-0.4692	0.0428
Btf3l4	0.0000	1.4667	0.0046	0.0000	-0.6596	0.0272
C1ql1	0.0000	1.3736	0.0246	0.0000	0.9390	0.0393
Cab39l	0.0000	1.0681	0.0158	0.0000	0.6777	0.0322
Cabp1	0.0000	-1.0481	0.0284	0.5588	-0.0887	0.0883
Caly	0.0000	-1.5171	0.0291	0.0001	-0.8946	0.0470
Camk2d	0.0000	-1.0091	0.0236	0.0094	-0.3341	0.0602
Camk2n1	0.0000	-1.3609	0.0054	0.0000	-0.7063	0.0223
Capza1	0.0000	2.1913	0.0014	0.1348	-0.1363	0.0738
Cars2	0.0000	1.3980	0.0147	0.0000	0.9849	0.0272
Cast	0.0000	1.3903	0.0111	0.0000	0.9264	0.0248
Cc2d1a	0.0000	1.0436	0.0204	0.0000	0.9004	0.0266
Cc2d2a	0.0000	1.2379	0.0181	0.0000	0.6535	0.0413
Ccdc22	0.0000	1.0294	0.0226	0.0000	0.6609	0.0392
Ccdc3	0.0000	-1.0886	0.0183	0.0588	0.2187	0.0684
Ccdc39	0.0000	1.6946	0.0229	0.0001	0.8513	0.0485
Ccdc60	0.0000	1.4846	0.0438	0.1490	0.5142	0.0745
Ccdc65	0.0003	1.4125	0.0492	0.4159	0.3233	0.0843
Cchcr1	0.0000	1.0280	0.0309	0.0000	0.9685	0.0342
Ccne1	0.0000	1.2840	0.0123	0.0000	0.8948	0.0249
Ccno	0.0000	4.0226	0.0211	0.5697	0.3126	0.0886
Cd109	0.0000	-1.1355	0.0391	0.8352	-0.0457	0.0957
Cd248	0.0000	-3.6734	0.0028	0.0000	-0.6907	0.0418
Cd36	0.0000	-3.8466	0.0194	0.0951	0.4666	0.0714
Cd47	0.0000	-1.8041	0.0034	0.0000	-0.6079	0.0304
Cd93	0.0001	-1.5006	0.0470	0.2286	0.4202	0.0780
Cdc42ep1	0.0000	-1.3333	0.0325	0.6678	0.0893	0.0915
Cdc42ep3	0.0000	-1.4030	0.0198	0.7665	-0.0459	0.0941
Cdc5l	0.0000	1.0363	0.0083	0.0000	0.8899	0.0132
Cdc6	0.0000	1.5301	0.0134	0.0000	0.9550	0.0298
Cdc73	0.0000	1.1137	0.0067	0.0000	0.5410	0.0280
Cdh11	0.0000	-1.5006	0.0248	0.3803	-0.1796	0.0832
Cdh20	0.0000	1.1696	0.0390	0.0026	-0.7685	0.0561
Cdh24	0.0000	-1.1535	0.0292	0.0000	-0.8550	0.0398
Cdh5	0.0000	-3.9483	0.0037	0.0000	-0.8954	0.0403
Cdkl2	0.0003	1.2776	0.0491	0.4330	0.2811	0.0847
Cdon	0.0000	2.3116	0.0193	0.5504	-0.1597	0.0881
Cenpp	0.0000	1.6312	0.0201	0.0000	0.9318	0.0408
Cep135	0.0000	1.1003	0.0207	0.0003	0.4834	0.0503
Cep57l1	0.0000	1.0039	0.0283	0.0000	0.9302	0.0323
Cep89	0.0000	1.3230	0.0122	0.0000	0.8081	0.0292
Cfap206	0.0000	2.8772	0.0323	0.0665	0.9378	0.0691
Cfap53	0.0000	2.8872	0.0430	0.2100	0.8800	0.0772
Cfap69	0.0000	1.1351	0.0432	0.6608	0.1181	0.0913
Chd5	0.0000	-1.0675	0.0290	0.9000	-0.0198	0.0974
Chgb	0.0000	-1.7034	0.0053	0.3281	0.1040	0.0814
Chodl	0.0000	-1.9246	0.0397	0.3058	-0.3741	0.0807
Chrd	0.0000	-1.6093	0.0261	0.0000	-0.9117	0.0449
Chst2	0.0000	-1.5003	0.0205	0.1955	-0.2223	0.0767
Chst3	0.0000	-2.1340	0.0092	0.0000	-0.7787	0.0384
Cit	0.0000	1.2009	0.0151	0.0000	0.8757	0.0266
Cldn1	0.0000	2.2324	0.0091	0.4009	-0.1545	0.0838
Clec2d	0.0001	-2.0533	0.0458	0.3854	0.3691	0.0834
Clip4	0.0000	-1.0583	0.0323	0.0018	0.5149	0.0549
Clmn	0.0000	1.6081	0.0101	0.0000	0.8793	0.0298
Clstn1	0.0000	-1.0120	0.0070	0.0000	-0.5563	0.0243
Cmss1	0.0000	1.0048	0.0295	0.0000	0.8132	0.0380
Cnga4	0.0000	3.4230	0.0437	0.4117	0.7132	0.0841
Cnn1	0.0000	1.5222	0.0395	0.0174	0.7657	0.0626
Cnnm1	0.0000	-2.1930	0.0294	0.0021	-0.9408	0.0554
Cnot1	0.0000	1.2777	0.0041	0.0000	0.4870	0.0301
Cnpy1	0.0000	1.5674	0.0170	0.0012	0.5507	0.0537
Cnrip1	0.0000	1.3884	0.0445	0.0030	0.9868	0.0565
Cntnap3	0.0001	1.6090	0.0468	0.1902	0.5534	0.0764
Coa4	0.0000	-1.4998	0.0226	0.9737	0.0057	0.0994
Coch	0.0000	2.3958	0.0243	0.0029	0.9886	0.0564
Col13a1	0.0000	-2.4912	0.0213	0.8656	-0.0454	0.0965
Col16a1	0.0000	-1.9002	0.0185	0.0000	0.9929	0.0379
Col4a1	0.0000	-1.7735	0.0039	0.0076	-0.2667	0.0594
Col4a2	0.0000	-1.4586	0.0055	0.0020	-0.2875	0.0553
Col6a1	0.0000	-1.9247	0.0047	0.0000	0.6468	0.0342

Col6a3	0.0000	-5.5626	0.0005	0.1620	0.1326	0.0752
Col6a6	0.0000	-3.5015	0.0245	0.4418	-0.2655	0.0851
Col8a1	0.0000	-2.0516	0.0077	0.0000	-0.6624	0.0427
Colec12	0.0000	2.1997	0.0237	0.0487	0.5765	0.0674
Colgalt2	0.0000	-1.7584	0.0259	0.0000	0.8632	0.0465
Coro2b	0.0000	-1.4522	0.0079	0.0001	-0.3938	0.0485
Cox7a2	0.0000	-1.4500	0.0076	0.0025	-0.3024	0.0560
Cox7c	0.0000	1.9499	0.0015	0.0000	0.5417	0.0299
Crispld2	0.0000	-2.0186	0.0165	0.0000	-0.9122	0.0415
Crtac1	0.0000	-2.0478	0.0074	0.0111	-0.3445	0.0608
Csgalnact1	0.0000	-2.0077	0.0268	0.0046	-0.7534	0.0579
Csnk1e	0.0000	-1.4581	0.0035	0.0000	-0.7919	0.0165
Cspp1	0.0000	1.0488	0.0152	0.0000	0.7084	0.0292
Ctdspl	0.0000	-1.5946	0.0195	0.0052	-0.4783	0.0582
Cthrc1	0.0000	-2.5731	0.0056	0.0000	0.9517	0.0284
Ctnnal1	0.0000	1.3994	0.0106	0.0000	0.9402	0.0237
Cwc22	0.0000	1.1320	0.0107	0.0102	0.2483	0.0604
Cxcl14	0.0000	-2.2335	0.0244	0.9808	-0.0063	0.0996
Cycs	0.0000	1.2398	0.0075	0.3772	0.0808	0.0831
Cygb	0.0000	-3.3068	0.0122	0.2036	-0.2966	0.0770
Cyp1b1	0.0000	-5.6026	0.0011	0.0719	0.2064	0.0697
Cys1	0.0000	-4.0294	0.0373	0.7206	0.1734	0.0929
Daam2	0.0000	-2.4114	0.0098	0.0009	-0.5886	0.0530
Dact3	0.0000	-1.8202	0.0056	0.0000	-0.7458	0.0294
Dap	0.0000	-1.7660	0.0107	0.0885	0.2290	0.0709
Dcn	0.0000	-5.0053	0.0005	0.0017	-0.3673	0.0549
Ddb1	0.0000	1.0323	0.0060	0.0000	0.7637	0.0141
Ddhd1	0.0000	-1.0731	0.0187	0.0000	-0.7237	0.0325
Ddr2	0.0000	-1.4358	0.0382	0.2337	0.3436	0.0782
Ddx11	0.0000	1.3457	0.0166	0.0000	0.6846	0.0408
Dennd1b	0.0000	1.0991	0.0147	0.0031	0.3238	0.0566
Dexi	0.0000	-1.1997	0.0282	0.0000	-0.9336	0.0372
Dhrs3	0.0000	-3.4427	0.0259	0.1753	-0.6323	0.0757
Dkk1	0.0000	2.0539	0.0402	0.5006	0.3086	0.0868
Dkk2	0.0001	1.7491	0.0476	0.1167	0.7235	0.0728
Dlx1	0.0001	-3.3211	0.0459	0.1774	0.7399	0.0758
Dnaaf3	0.0000	1.3167	0.0406	0.0007	0.9575	0.0525
Dnah1	0.0000	2.0350	0.0276	0.2445	0.3653	0.0786
Dnajc19	0.0000	1.2197	0.0211	0.0050	0.4212	0.0581
Dpy19l1	0.0000	1.2716	0.0081	0.0000	0.6409	0.0293
Dpy19l3	0.0000	1.2891	0.0121	0.0031	-0.3528	0.0566
Dpy30	0.0000	1.0488	0.0128	0.0000	0.9686	0.0166
Drc3	0.0000	1.0103	0.0443	0.0002	0.8907	0.0495
Drd1	0.0000	-4.6274	0.0223	0.2363	-0.4099	0.0783
Dscaml1	0.0001	-1.2575	0.0458	0.8875	0.0415	0.0971
Dtx1	0.0000	-1.2511	0.0143	0.0000	0.5026	0.0441
Dusp14	0.0000	2.0244	0.0138	0.0000	0.8785	0.0434
Dusp26	0.0000	1.5737	0.0177	0.0000	0.8442	0.0403
Dusp6	0.0000	1.0609	0.0159	0.0000	0.6520	0.0333
Dzip1	0.0000	-1.2542	0.0088	0.0014	-0.3016	0.0540
Ecm1	0.0000	-1.0668	0.0441	0.0004	0.8092	0.0509
Eda	0.0000	-1.5220	0.0223	0.0053	-0.4983	0.0583
Eda2r	0.0000	-1.0469	0.0163	0.0000	-0.6588	0.0326
Edn1	0.0001	1.6700	0.0470	0.6231	-0.2223	0.0902
Ednra	0.0000	-3.5312	0.0040	0.0000	0.9288	0.0320
Eef1g	0.0000	1.6999	0.0017	0.0000	0.3786	0.0377
Efcab11	0.0000	1.6710	0.0238	0.0129	0.5638	0.0614
Efemp1	0.0000	-1.7343	0.0250	0.0000	0.8471	0.0466
Efemp2	0.0000	-2.4776	0.0053	0.3706	-0.1231	0.0828
Efhd2	0.0000	-1.6624	0.0065	0.0001	-0.4304	0.0473
Efna3	0.0000	-1.5251	0.0128	0.0000	-0.5623	0.0453
Efnb1	0.0000	-1.2829	0.0081	0.0000	-0.7842	0.0223
Efnb3	0.0000	-1.8472	0.0020	0.0000	-0.4051	0.0388
Efr3a	0.0000	1.0287	0.0181	0.0000	0.9450	0.0222
Egflam	0.0000	-3.3667	0.0034	0.0000	-0.9280	0.0319
Egfr	0.0000	-2.2220	0.0085	0.8817	-0.0233	0.0970
Ehd2	0.0000	-1.8693	0.0163	0.5686	0.0997	0.0885
Elk3	0.0000	-1.0780	0.0291	0.0000	0.6959	0.0432
Emc10	0.0000	-1.5603	0.0039	0.2514	0.0949	0.0789
Emilin1	0.0000	-1.2862	0.0146	0.0000	0.6399	0.0375
Emilin3	0.0000	-2.1563	0.0290	0.1107	-0.4682	0.0724
Eno1	0.0000	3.5792	0.0002	0.0000	0.8726	0.0140
Eno2	0.0000	-1.2088	0.0132	0.0000	-0.4564	0.0451
Enpep	0.0000	-4.5619	0.0111	0.3542	-0.2413	0.0823
Enpp1	0.0000	-4.1724	0.0031	0.0000	-0.9442	0.0332
Entpd1	0.0000	-2.5704	0.0255	0.5871	-0.1606	0.0891
Epb41l1	0.0000	-1.0900	0.0107	0.0000	-0.8557	0.0185

Epm2a	0.0000	-1.3040	0.0253	0.0000	-0.7795	0.0431
Epn3	0.0000	1.9772	0.0423	0.0731	0.8221	0.0697
Etfa	0.0000	1.0645	0.0108	0.0000	0.9002	0.0169
Ets1	0.0000	-2.1700	0.0100	0.0004	-0.5748	0.0508
Etv1	0.0000	1.6906	0.0109	0.0595	0.2774	0.0685
Eva1c	0.0000	-3.0900	0.0319	0.8049	0.0963	0.0949
Exosc8	0.0000	1.0146	0.0184	0.0000	0.8653	0.0250
F2r	0.0000	-1.4620	0.0041	0.0000	-0.8698	0.0153
F3	0.0000	1.1829	0.0297	0.0484	0.3710	0.0674
Fam107b	0.0000	-1.0273	0.0145	0.4271	-0.0765	0.0845
Fam131c	0.0000	-1.8002	0.0362	0.0437	-0.6278	0.0669
Fam163a	0.0000	2.8576	0.0075	0.8360	0.0461	0.0958
Fam169a	0.0000	1.0071	0.0155	0.0000	0.8721	0.0214
Fam189a2	0.0000	-1.4661	0.0302	0.0799	-0.3815	0.0703
Fam20a	0.0000	-4.5407	0.0403	0.4240	0.4130	0.0845
Fam210b	0.0000	-2.9199	0.0019	0.0000	-0.7250	0.0303
Fam217b	0.0000	-1.5394	0.0254	0.0000	-0.9335	0.0426
Fam220a	0.0000	-1.1105	0.0145	0.0000	-0.8491	0.0234
Fam222a	0.0000	-1.1391	0.0301	0.0000	-0.9138	0.0380
Fam234b	0.0000	-1.8330	0.0049	0.0000	-0.4787	0.0426
Fam32a	0.0000	-1.1572	0.0095	0.1784	-0.1211	0.0759
Fancm	0.0000	1.1337	0.0137	0.0097	0.2816	0.0603
Fbln1	0.0000	-1.9768	0.0022	0.0000	-0.5601	0.0314
Fbln5	0.0000	-1.9567	0.0230	0.0002	0.8633	0.0493
Fbn2	0.0000	-1.8564	0.0244	0.0005	-0.8722	0.0517
Fbxl18	0.0000	-1.3470	0.0088	0.0000	-0.9764	0.0180
Fbxl20	0.0000	-1.0804	0.0102	0.0000	-0.6575	0.0256
Fcf1	0.0000	1.7333	0.0072	0.0000	0.6159	0.0401
Fgd6	0.0000	-1.6689	0.0164	0.0096	-0.4156	0.0602
Fgf11	0.0000	-1.4041	0.0151	0.0003	-0.4816	0.0503
Fgf2	0.0000	-4.3795	0.0227	0.8991	0.0420	0.0973
Fgfrl1	0.0000	-1.7151	0.0086	0.0855	-0.2058	0.0707
Fhl1	0.0000	-1.7526	0.0031	0.0000	-0.7196	0.0230
Fhl2	0.0000	-2.1753	0.0177	0.0003	0.7194	0.0502
Fibin	0.0000	-3.0076	0.0163	0.0123	0.6251	0.0612
Filip1l	0.0000	-1.8687	0.0180	0.0000	-0.9180	0.0414
Fkbp10	0.0000	-1.3936	0.0144	0.0000	0.7922	0.0320
Fkrp	0.0000	-1.2762	0.0138	0.0000	-0.9758	0.0226
Fli1	0.0000	-3.0329	0.0401	0.2408	-0.6292	0.0785
Fndc4	0.0000	-1.1869	0.0152	0.0000	-0.9219	0.0238
Focad	0.0000	1.9999	0.0031	0.0000	0.5738	0.0371
Foxf2	0.0000	-4.9690	0.0121	0.0006	0.7113	0.0519
Foxn3	0.0000	-1.4923	0.0073	0.0000	-0.8946	0.0214
Foxo6	0.0000	-1.3742	0.0343	0.1630	-0.3241	0.0752
Foxp1	0.0000	-1.6533	0.0081	0.0000	-0.6258	0.0378
Foxp2	0.0000	-2.4034	0.0242	0.0046	-0.9055	0.0579
Frem1	0.0000	-1.9272	0.0124	0.3599	-0.1418	0.0825
Frs3	0.0000	-1.2984	0.0250	0.0030	-0.5020	0.0565
Fxyd1	0.0000	-1.5666	0.0450	0.0831	0.5741	0.0705
Fxyd6	0.0000	-1.3209	0.0051	0.0018	-0.2505	0.0550
Fyb	0.0000	-4.8237	0.0314	0.1686	0.5280	0.0754
Fzd10	0.0000	2.3190	0.0013	0.4286	-0.0758	0.0846
Fzd2	0.0000	-1.7168	0.0026	0.0000	-0.5054	0.0321
Fzd6	0.0000	1.1982	0.0274	0.0005	0.6122	0.0518
Gabarapl1	0.0000	-1.2495	0.0060	0.0000	-0.4198	0.0384
Gad2	0.0000	-2.5570	0.0061	0.0118	-0.3861	0.0610
Galnt4	0.0000	1.4547	0.0210	0.0001	0.7103	0.0471
Galnt9	0.0000	-1.0095	0.0377	0.0015	-0.6148	0.0544
Gars	0.0000	1.1683	0.0053	0.0000	0.8313	0.0137
Gas1	0.0000	1.3950	0.0061	0.0000	-0.4800	0.0396
Gas5	0.0000	-2.6810	0.0129	0.0010	0.6597	0.0533
Gas7	0.0000	-1.9178	0.0127	0.0000	-0.6617	0.0463
Gbp4	0.0000	-2.0039	0.0354	0.1333	0.4862	0.0737
Gch1	0.0000	-1.1676	0.0411	0.0000	0.9407	0.0457
Gdf10	0.0000	-3.0326	0.0280	0.2933	-0.4654	0.0802
Gdf7	0.0000	7.9776	0.0074	0.4637	0.5287	0.0857
Gdpd2	0.0000	-1.7312	0.0159	0.3113	-0.1649	0.0809
Gfm1	0.0000	1.0572	0.0142	0.0000	0.6803	0.0298
Gfod1	0.0000	-1.4063	0.0264	0.0035	-0.5502	0.0570
Ggt5	0.0000	-3.2208	0.0266	0.1490	0.4630	0.0745
Ghr	0.0000	-1.3658	0.0167	0.0000	-0.6541	0.0416
Gins1	0.0000	1.2429	0.0182	0.0000	0.9608	0.0279
Gldc	0.0000	1.0020	0.0224	0.0022	0.3905	0.0556
Glrx3	0.0000	1.4126	0.0037	0.0520	0.1548	0.0678
Glt8d2	0.0000	-2.9661	0.0327	0.6793	0.1602	0.0918
Gmnc	0.0000	7.8309	0.0142	0.6328	0.4590	0.0905
Gng10	0.0000	-1.8809	0.0071	0.0021	-0.3807	0.0554

Gng11	0.0000	-2.0188	0.0272	0.0108	0.6184	0.0607
Gng3	0.0000	-1.3945	0.0091	0.0000	-0.9715	0.0193
Gng4	0.0000	-1.5568	0.0326	0.8976	0.0310	0.0973
Gpn2	0.0000	-1.2188	0.0295	0.0094	0.4531	0.0601
Gpr137	0.0000	-1.0316	0.0257	0.0000	-0.7797	0.0356
Gpr37	0.0000	-4.5565	0.0077	0.6963	0.0746	0.0922
Gps1	0.0000	1.2271	0.0084	0.0055	-0.2634	0.0584
Gramd4	0.0000	-1.5882	0.0086	0.0000	-0.9132	0.0239
Grin2d	0.0000	-1.7548	0.0256	0.0280	-0.4896	0.0647
Grm4	0.0000	-1.0418	0.0369	0.6316	-0.0946	0.0905
Grm5	0.0001	-1.4580	0.0458	0.0040	-0.9992	0.0574
Habp4	0.0000	-1.3988	0.0130	0.0000	-0.8952	0.0271
Hadhb	0.0000	2.2311	0.0040	0.7970	0.0345	0.0947
Haghl	0.0000	-1.5865	0.0138	0.0000	-0.8313	0.0346
Has2	0.0000	-2.3197	0.0168	0.0000	-0.8879	0.0465
Haus2	0.0000	-1.2519	0.0112	0.6276	-0.0495	0.0904
Haus5	0.0000	1.4780	0.0185	0.0000	0.8299	0.0396
Hax1	0.0000	1.2791	0.0112	0.0000	0.7671	0.0282
Hdac1	0.0000	2.7591	0.0009	0.0000	0.6161	0.0321
Hdac4	0.0000	1.1412	0.0142	0.0000	0.8945	0.0232
Hdac5	0.0000	-1.1307	0.0083	0.0000	-0.8692	0.0159
Hectd2	0.0000	-1.0685	0.0299	0.0000	-0.8188	0.0395
Herc4	0.0000	1.1873	0.0082	0.0000	0.9123	0.0162
Hhip	0.0000	-1.5899	0.0171	0.0000	0.9303	0.0325
Hic1	0.0000	-5.4542	0.0026	0.0013	-0.5390	0.0540
Hlf	0.0000	-1.5865	0.0211	0.0001	-0.7200	0.0472
Hlx	0.0002	-2.0492	0.0483	0.1965	0.5871	0.0768
Hmcn1	0.0000	-2.6918	0.0070	0.0001	-0.6766	0.0478
Hmgn2	0.0000	2.0236	0.0020	0.0000	-0.9193	0.0174
Hnrnpa1	0.0000	1.4019	0.0032	0.0001	-0.2877	0.0484
Hnrnpa3	0.0000	1.9962	0.0007	0.0032	-0.1855	0.0567
Hpcal1	0.0000	-1.3167	0.0153	0.0004	-0.4478	0.0511
Hsd11b2	0.0000	1.4691	0.0244	0.0469	-0.4179	0.0672
Hspa12b	0.0000	-1.4483	0.0330	0.0179	-0.5647	0.0627
Htr1b	0.0001	1.4546	0.0461	0.0342	-0.8460	0.0656
Hyi	0.0000	-1.7044	0.0290	0.1249	-0.3713	0.0732
Hypk	0.0000	-4.3955	0.0017	0.0070	0.3737	0.0592
Ier5l	0.0000	-1.3021	0.0104	0.0000	-0.8197	0.0245
Igdcc4	0.0000	-1.2088	0.0091	0.8784	0.0139	0.0969
Igf1	0.0000	-2.1353	0.0422	0.3711	0.4280	0.0828
Igf2	0.0000	-2.8593	0.0009	0.0987	-0.1682	0.0716
Igfbp3	0.0000	-2.0356	0.0107	0.0000	0.7238	0.0432
Igfbp4	0.0000	-2.8697	0.0008	0.0000	0.6268	0.0276
Igfbp7	0.0000	-3.1150	0.0082	0.0024	0.5617	0.0558
Il1rap	0.0000	1.5506	0.0167	0.7549	-0.0521	0.0938
Inava	0.0000	2.0219	0.0104	0.0000	0.7338	0.0451
Inha	0.0000	-1.3964	0.0395	0.0011	-0.9078	0.0535
Inhba	0.0000	-3.7188	0.0268	0.0589	-0.7758	0.0684
Iqcc	0.0000	-1.4804	0.0186	0.2971	0.1579	0.0804
Irf2bp1	0.0000	-1.0702	0.0118	0.0000	-0.8105	0.0209
Islr	0.0000	-6.5190	0.0006	0.0033	-0.3538	0.0569
Ism1	0.0000	-1.3954	0.0436	0.5725	-0.1690	0.0887
Isyna1	0.0000	1.0949	0.0101	0.0000	0.5462	0.0329
Itga11	0.0000	-2.3697	0.0287	0.0005	0.9944	0.0518
Itga9	0.0000	-2.1500	0.0110	0.0002	-0.6278	0.0499
Itgb1	0.0000	1.0249	0.0116	0.0000	0.6073	0.0294
Itih2	0.0000	-6.6105	0.0137	0.1227	-0.3503	0.0732
Itih5	0.0000	-3.8762	0.0013	0.0000	-0.9616	0.0253
Itm2a	0.0000	-1.7527	0.0073	0.0067	0.3305	0.0590
Itpa	0.0000	1.3557	0.0086	0.0000	-0.5100	0.0415
Itpr1	0.0000	1.3440	0.0086	0.0000	0.7408	0.0270
Itpr2	0.0000	-2.1525	0.0167	0.0050	-0.5713	0.0581
Jmjd8	0.0000	-1.0624	0.0238	0.0000	-0.6404	0.0419
Jrk	0.0000	-1.0752	0.0231	0.0023	-0.4054	0.0557
Kazald1	0.0000	-2.4709	0.0251	0.0167	0.6505	0.0624
Kbtbd11	0.0000	-2.1793	0.0014	0.0000	-0.5136	0.0315
Kcnj3	0.0002	1.0964	0.0481	0.0441	0.5918	0.0670
Kcnj8	0.0000	-5.3820	0.0240	0.3609	-0.3062	0.0825
Kcp	0.0000	-2.4318	0.0287	0.4990	-0.2096	0.0867
Kctd13	0.0000	-1.0522	0.0211	0.0000	-0.8892	0.0275
Khk	0.0001	-1.2171	0.0461	0.1391	-0.4347	0.0740
Kif11	0.0000	1.1649	0.0123	0.0000	0.5331	0.0392
Kif15	0.0000	1.0708	0.0139	0.0005	0.3597	0.0515
Kif9	0.0000	1.6700	0.0354	0.5874	0.1747	0.0892
Kifc2	0.0000	-1.2606	0.0306	0.0000	-0.9247	0.0419
Kirrel2	0.0000	2.5386	0.0073	0.0325	0.4060	0.0654
Klhdc8a	0.0000	-1.4182	0.0303	0.0947	-0.3533	0.0713

Knop1	0.0000	-1.0620	0.0076	0.0002	-0.2840	0.0491
Kpna2	0.0000	8.3761	0.0002	0.0159	-0.6278	0.0621
Kras	0.0000	-1.1452	0.0068	0.0000	-0.6709	0.0217
L3hypdh	0.0000	-1.1104	0.0418	0.7655	0.0680	0.0940
Lama2	0.0000	-1.8752	0.0119	0.0095	-0.3949	0.0602
Lamb1	0.0000	-1.2437	0.0068	0.3879	-0.0737	0.0834
Lars	0.0000	1.0374	0.0082	0.0000	0.8599	0.0140
Ldb2	0.0000	2.1809	0.0151	0.0065	0.6142	0.0589
Lef1	0.0000	3.1030	0.0052	0.0001	0.8181	0.0471
Lepr	0.0000	-3.8278	0.0177	0.0161	-0.9778	0.0622
Lgals1	0.0000	-1.3792	0.0088	0.0000	0.8770	0.0213
Lgi2	0.0000	-1.2340	0.0222	0.2102	0.1837	0.0772
Lhfpl2	0.0000	-1.7624	0.0103	0.0014	-0.4558	0.0541
Lhpp	0.0000	1.0551	0.0221	0.5800	0.0746	0.0889
Lhx2	0.0000	1.2559	0.0336	0.2656	0.2527	0.0793
Lipe	0.0000	-1.1109	0.0350	0.0878	-0.3304	0.0709
Lix1l	0.0000	-1.7352	0.0022	0.0000	-0.9936	0.0105
Lmcd1	0.0000	-2.5484	0.0216	0.0020	-0.8559	0.0553
Lox	0.0000	-5.2620	0.0040	0.0716	0.4931	0.0696
Loxl2	0.0000	-1.1068	0.0143	0.0001	-0.4240	0.0473
Lpar1	0.0000	-1.7013	0.0182	0.1283	0.2708	0.0734
Lpcat2	0.0001	1.2555	0.0455	0.2650	0.3524	0.0793
Lpin1	0.0000	-1.1645	0.0151	0.0723	-0.2002	0.0697
Lrfn4	0.0000	-1.5125	0.0066	0.0000	-0.8693	0.0213
Lrp1b	0.0000	2.6775	0.0111	0.1130	0.3858	0.0726
Lrp2	0.0000	2.4496	0.0042	0.3443	0.1380	0.0820
Lrp4	0.0000	1.9260	0.0032	0.0287	0.2290	0.0648
Lrrc32	0.0000	-3.6211	0.0122	0.0000	0.9240	0.0437
Lrrc8c	0.0000	1.2423	0.0134	0.2593	-0.1349	0.0791
Lrriq1	0.0000	1.8395	0.0352	0.0700	-0.6751	0.0695
Lrrk1	0.0000	-1.7219	0.0179	0.0000	-0.9474	0.0375
Lrtm2	0.0000	-1.2511	0.0430	0.9746	0.0085	0.0995
Lsm5	0.0000	3.7507	0.0027	0.0107	0.5235	0.0606
Lsp1	0.0000	-1.0989	0.0402	0.9179	0.0226	0.0979
Ltv1	0.0000	1.0914	0.0127	0.0000	0.8413	0.0219
Lum	0.0000	-7.4595	0.0008	0.5376	-0.0577	0.0877
Lypd1	0.0000	-1.5330	0.0201	0.0007	-0.5752	0.0523
Lypd6	0.0000	-1.7379	0.0282	0.0120	-0.6105	0.0611
Lysmd2	0.0000	-1.7829	0.0281	0.9107	0.0265	0.0976
Lysmd4	0.0000	-1.3117	0.0163	0.0000	-0.9555	0.0268
Lzts3	0.0000	-1.5633	0.0169	0.0000	-0.8791	0.0360
Magee2	0.0000	1.8029	0.0414	0.0152	0.9760	0.0620
Man2b2	0.0000	1.0219	0.0201	0.0000	0.6551	0.0366
Map7d2	0.0000	-1.0601	0.0226	0.0000	-0.8795	0.0299
Mat2a	0.0000	1.0574	0.0080	0.0000	0.4327	0.0363
Matk	0.0003	-1.4130	0.0491	0.0207	-0.8515	0.0634
Matn2	0.0000	-1.2194	0.0252	0.0065	-0.4412	0.0589
Mblac2	0.0000	-1.1334	0.0324	0.0000	-0.9602	0.0387
Mbnl3	0.0000	1.5161	0.0275	0.1363	0.3412	0.0739
Mcoln3	0.0000	2.5646	0.0324	0.4338	0.3639	0.0848
Mdfi	0.0000	-1.1212	0.0201	0.1176	-0.1973	0.0728
Mdn1	0.0000	1.0558	0.0090	0.1032	0.1353	0.0719
Med14	0.0000	1.1903	0.0095	0.0244	0.2156	0.0641
Mef2c	0.0000	-1.9426	0.0315	0.3092	0.2928	0.0808
Megf10	0.0000	-1.0440	0.0223	0.0000	-0.9651	0.0263
Melk	0.0000	1.3674	0.0114	0.0000	0.8596	0.0269
Meox2	0.0000	-2.4221	0.0328	0.2533	0.3840	0.0789
Mest	0.0000	1.4854	0.0032	0.0000	-0.9348	0.0126
Met	0.0000	-1.8724	0.0183	0.0000	-0.9970	0.0383
Mfap4	0.0000	-3.2032	0.0014	0.2779	-0.1179	0.0798
Mfhas1	0.0000	-1.3130	0.0109	0.0000	-0.7008	0.0304
Mgst1	0.0000	-1.0814	0.0309	0.2341	-0.2031	0.0782
Micall1	0.0000	-1.1206	0.0142	0.0000	-0.9309	0.0207
Mkrn3	0.0000	-1.0111	0.0266	0.0000	-0.6855	0.0400
Mlc1	0.0000	1.7990	0.0258	0.0230	-0.6175	0.0638
Mms22l	0.0000	1.4191	0.0116	0.0000	0.9249	0.0262
Morf4l1	0.0000	1.8972	0.0014	0.0000	-0.5455	0.0280
Morn5	0.0000	2.8324	0.0453	0.3969	0.6246	0.0837
Moxd1	0.0000	-2.5306	0.0051	0.0000	-0.7582	0.0377
Mrc2	0.0000	-1.6109	0.0067	0.7807	-0.0298	0.0943
Mrpl1	0.0000	1.0401	0.0190	0.0000	0.9444	0.0234
Mrpl21	0.0000	1.0212	0.0194	0.0000	0.6011	0.0386
Mrpl22	0.0000	1.2805	0.0186	0.0136	0.3607	0.0616
Mrpl23	0.0000	4.7701	0.0035	0.0208	0.6734	0.0634
Mrpl33	0.0000	-1.0975	0.0212	0.3647	0.1135	0.0827
Mrpl42	0.0000	1.0238	0.0164	0.0000	0.9918	0.0188
Mrpl48	0.0000	1.3540	0.0147	0.8738	-0.0218	0.0968

Mrps36	0.0000	1.3064	0.0219	0.0022	0.5056	0.0555
Mrto4	0.0000	1.4547	0.0078	0.0000	0.7895	0.0265
Mrvi1	0.0000	-3.2092	0.0409	0.8739	-0.0831	0.0968
Mtbp	0.0000	1.0195	0.0211	0.0000	0.8019	0.0305
Mxra8	0.0000	-1.1917	0.0220	0.0000	0.6504	0.0412
Mybl1	0.0000	-1.0936	0.0288	0.0027	-0.4962	0.0562
Mybpc1	0.0000	-1.9968	0.0337	0.0822	-0.5482	0.0704
Mycbpap	0.0001	2.0046	0.0458	0.4665	0.3799	0.0857
Myh7	0.0000	-1.9064	0.0305	0.0207	0.6352	0.0634
Myh8	0.0000	-2.9615	0.0387	0.5420	0.3494	0.0878
Myl3	0.0001	-2.5640	0.0471	0.5559	0.3154	0.0882
Mylk	0.0000	-1.5249	0.0253	0.0871	-0.3382	0.0708
Myo5c	0.0000	1.0164	0.0415	0.0004	0.7935	0.0510
Myo6	0.0000	-1.1846	0.0115	0.9553	-0.0055	0.0989
Nab1	0.0000	1.1022	0.0190	0.0000	0.7848	0.0317
Naca	0.0000	1.8589	0.0010	0.0000	0.3767	0.0358
Nacc2	0.0000	-1.1014	0.0267	0.0086	-0.4027	0.0598
Nae1	0.0000	1.0786	0.0110	0.0000	0.5473	0.0336
Nanos1	0.0000	-1.1378	0.0307	0.0000	-0.7980	0.0436
Nap1l1	0.0000	1.3484	0.0041	0.0139	-0.1919	0.0617
Nbl1	0.0000	-3.4940	0.0023	0.0045	-0.3959	0.0578
Ncapg	0.0000	1.3167	0.0088	0.0000	0.9982	0.0175
Ncoa4	0.0000	1.5575	0.0042	0.0683	0.1684	0.0694
Ndufa1	0.0000	1.0916	0.0151	0.0000	0.5906	0.0368
Ndufab1	0.0000	1.3456	0.0072	0.0000	0.6326	0.0303
Neurl1b	0.0000	-1.0577	0.0403	0.2748	0.2282	0.0796
Neurod1	0.0000	-1.0540	0.0332	0.0001	-0.7031	0.0479
Nfib	0.0000	-1.5746	0.0360	0.4697	-0.2143	0.0859
Ngef	0.0000	1.6174	0.0309	0.0007	0.9081	0.0523
Nid1	0.0000	-1.6385	0.0025	0.0702	0.1410	0.0695
Nifk	0.0000	1.1095	0.0089	0.0000	0.7876	0.0195
Nkd1	0.0000	1.3215	0.0101	0.2231	-0.1352	0.0777
Nkiras1	0.0000	-1.2769	0.0211	0.0000	-0.6361	0.0446
Nlrc4	0.0000	3.4826	0.0311	0.1697	0.8419	0.0755
Nmd3	0.0000	1.2187	0.0087	0.0000	0.8419	0.0201
Nol3	0.0000	1.1515	0.0320	0.0002	0.7369	0.0489
Nr4a3	0.0000	1.4098	0.0222	0.0000	-0.9981	0.0383
Nrn1	0.0000	1.8932	0.0136	0.9784	-0.0051	0.0996
Nrp1	0.0000	-3.1097	0.0015	0.0000	-0.6477	0.0350
Nrp2	0.0000	-1.0071	0.0170	0.0000	-0.5856	0.0363
Nudt16	0.0000	-1.3708	0.0265	0.6170	-0.0899	0.0900
Nudt3	0.0000	-1.0050	0.0101	0.0000	-0.8650	0.0154
Nudt7	0.0002	1.1140	0.0485	0.6990	-0.1202	0.0923
Nup160	0.0000	1.1684	0.0090	0.0000	0.8026	0.0206
Nup188	0.0000	1.0904	0.0102	0.0000	0.9331	0.0159
Nup205	0.0000	1.0640	0.0081	0.0000	0.5575	0.0278
Nup35	0.0000	1.6066	0.0106	0.0215	0.3172	0.0636
Nup54	0.0000	1.0065	0.0143	0.0000	0.6947	0.0274
Nup85	0.0000	1.1453	0.0121	0.0000	0.6426	0.0321
Nutf2	0.0000	5.3239	0.0001	0.0000	0.7470	0.0379
Odc1	0.0000	1.5035	0.0023	0.0000	0.8309	0.0127
Ogn	0.0000	-1.8509	0.0407	0.1331	0.5978	0.0736
Olfml1	0.0000	-2.2265	0.0217	0.2168	-0.3018	0.0775
Olfml2a	0.0000	1.2874	0.0281	0.0000	0.9895	0.0385
Olfml2b	0.0000	-1.9515	0.0200	0.4673	0.1485	0.0858
Olfml3	0.0000	-1.8964	0.0123	0.0000	0.6999	0.0420
Opa3	0.0000	-1.5454	0.0065	0.0000	-0.5031	0.0395
Ormdl2	0.0000	1.1798	0.0227	0.0000	0.9648	0.0310
Otud4	0.0000	1.3835	0.0050	0.0000	0.9841	0.0132
Oxa1l	0.0000	-1.0160	0.0140	0.1702	0.1285	0.0755
P2ry1	0.0000	-1.2518	0.0453	0.0058	-0.8092	0.0586
P2ry14	0.0000	-3.2098	0.0358	0.0969	0.6827	0.0715
P3h2	0.0000	1.2963	0.0209	0.0008	-0.5481	0.0528
P3h4	0.0000	-1.4814	0.0137	0.0613	-0.2485	0.0686
P4ha3	0.0000	-2.4036	0.0088	0.0770	-0.2917	0.0701
P4htm	0.0000	-2.1168	0.0184	0.0055	-0.5805	0.0584
Pafah2	0.0000	-1.0978	0.0323	0.0065	-0.4843	0.0589
Palmd	0.0000	5.8556	0.0013	0.0003	0.9247	0.0505
Pam16	0.0000	4.3479	0.0068	0.7228	0.1259	0.0930
Papss2	0.0000	-2.0794	0.0361	0.6529	-0.1550	0.0910
Parvb	0.0000	-1.2317	0.0265	0.2005	0.2156	0.0769
Pcdh18	0.0000	-1.2946	0.0085	0.0000	-0.9798	0.0164
Pcdhga10	0.0000	-1.0138	0.0323	0.7072	-0.0613	0.0926
Pced1a	0.0000	-1.3877	0.0154	0.0003	-0.4807	0.0503
Pcna	0.0000	1.5943	0.0032	0.0000	0.6032	0.0275
Pcnt	0.0000	1.3043	0.0065	0.0000	0.8160	0.0198
Pcolce	0.0000	-1.7378	0.0093	0.0000	0.6060	0.0421

Pdcd10	0.0000	1.4200	0.0092	0.5077	0.0757	0.0870
Pdcd11	0.0000	1.0393	0.0120	0.0000	0.5960	0.0311
Pde1a	0.0000	-1.8668	0.0344	0.5009	-0.2077	0.0868
Pde5a	0.0000	-1.0779	0.0157	0.0001	-0.4182	0.0474
Pde8b	0.0000	-1.4145	0.0353	0.0025	-0.7486	0.0559
Pdgfra	0.0000	-2.3092	0.0048	0.0000	-0.6576	0.0400
Pdgfrb	0.0000	-3.2949	0.0021	0.0857	-0.2115	0.0707
Pdia6	0.0000	1.0776	0.0079	0.0000	0.5936	0.0259
Pdik1l	0.0000	-1.0311	0.0234	0.0000	-0.8376	0.0313
Pdk2	0.0000	-1.0652	0.0302	0.0005	-0.5706	0.0515
Pdlim3	0.0000	-2.5355	0.0221	0.0006	-0.9933	0.0520
Pdxp	0.0000	-1.0400	0.0167	0.0000	-0.8121	0.0254
Pdzd9	0.0000	-3.3172	0.0444	0.6507	0.2556	0.0909
Per3	0.0000	-2.0908	0.0114	0.3817	-0.1407	0.0833
Pgf	0.0000	-4.1708	0.0158	0.0015	0.7925	0.0544
Pgk1	0.0000	1.8448	0.0010	0.2176	-0.0824	0.0775
Pgm5	0.0000	2.2196	0.0174	0.0003	0.8907	0.0503
Phb	0.0000	1.7529	0.0031	0.0056	0.2580	0.0585
Phf1	0.0000	-1.1207	0.0261	0.0000	-0.8082	0.0374
Phgdh	0.0000	1.3020	0.0058	0.0000	0.5129	0.0333
Phlda1	0.0000	-1.9304	0.0039	0.0000	-0.7742	0.0253
Phldb2	0.0000	-1.2393	0.0114	0.0000	-0.6826	0.0307
Pid1	0.0000	-1.3434	0.0102	0.0000	-0.8221	0.0256
Pik3ip1	0.0000	-1.6008	0.0109	0.0035	-0.3690	0.0570
Pik3r5	0.0000	-2.1209	0.0442	0.0408	-0.9272	0.0665
Pin4	0.0000	3.0853	0.0048	0.0734	-0.3806	0.0698
Pitpnc1	0.0000	-1.0553	0.0109	0.0003	-0.3154	0.0504
Pitpnm1	0.0000	-1.1955	0.0251	0.0000	-0.7724	0.0400
Pkig	0.0000	-1.2489	0.0270	0.8443	0.0328	0.0960
Plcd1	0.0000	-1.9655	0.0091	0.0000	-0.7627	0.0369
Pld1	0.0000	-1.1555	0.0286	0.2501	0.1870	0.0788
Plekhb1	0.0004	1.0524	0.0498	0.0698	0.5367	0.0695
Plekhh2	0.0000	-1.0808	0.0282	0.0079	-0.4154	0.0596
Plpp3	0.0000	-1.5723	0.0056	0.5419	-0.0595	0.0878
Plppr1	0.0000	-1.4081	0.0126	0.0000	-0.9265	0.0262
Plppr5	0.0000	-2.1797	0.0442	0.0372	-0.9724	0.0660
Pltp	0.0000	-1.0326	0.0086	0.0000	0.3639	0.0409
Plxnd1	0.0000	-2.3018	0.0075	0.9091	0.0162	0.0976
Pmp22	0.0000	-1.2622	0.0164	0.3583	-0.1152	0.0824
Pnma2	0.0000	-1.7594	0.0121	0.0081	-0.3843	0.0596
Pnpla6	0.0000	1.1821	0.0101	0.0000	0.9490	0.0174
Pola2	0.0000	1.2268	0.0127	0.0000	0.9928	0.0205
Polr1b	0.0000	1.4962	0.0082	0.0000	0.9405	0.0221
Polr3gl	0.0000	-1.0265	0.0300	0.2144	-0.1933	0.0774
Postn	0.0000	-4.0023	0.0069	0.0020	0.8585	0.0553
Pou6f2	0.0000	2.2585	0.0113	0.0040	-0.6142	0.0574
Ppia	0.0000	2.4850	0.0004	0.0000	0.5637	0.0217
Ppid	0.0000	1.1725	0.0061	0.0000	0.7257	0.0193
Ppih	0.0000	3.7368	0.0029	0.4100	0.1749	0.0840
Ppil1	0.0000	-1.0128	0.0202	0.0000	0.7092	0.0320
Ppm1k	0.0000	-1.2085	0.0271	0.0421	-0.3355	0.0667
Ppp1r37	0.0000	-1.0018	0.0140	0.0000	-0.8012	0.0217
Ppp2r2c	0.0000	-1.2961	0.0204	0.4128	0.1218	0.0842
Ppp3ca	0.0000	-1.4183	0.0054	0.0000	-0.8669	0.0176
Prcp	0.0000	1.3432	0.0107	0.7101	0.0430	0.0927
Prdm16	0.0000	1.4031	0.0189	0.0018	0.5012	0.0550
Prim1	0.0000	1.2147	0.0158	0.0000	0.8456	0.0290
Prkag2	0.0000	-1.0607	0.0176	0.0000	-0.9256	0.0229
Prkar1b	0.0000	-1.0740	0.0220	0.0000	-0.7497	0.0347
Prkcb	0.0000	-1.1158	0.0250	0.0000	-0.9427	0.0314
Prom1	0.0000	-1.2913	0.0058	0.0000	-0.5507	0.0297
Prpf19	0.0000	1.0887	0.0061	0.0000	0.3800	0.0381
Prph	0.0000	-2.1122	0.0135	0.0265	0.3884	0.0644
Prrg1	0.0000	-1.1043	0.0352	0.0565	-0.3699	0.0682
Prrt4	0.0000	-1.7526	0.0376	0.2599	-0.3531	0.0791
Prrx1	0.0000	-1.9656	0.0405	0.3262	0.4124	0.0813
Psma5	0.0000	1.6115	0.0040	0.0088	0.2461	0.0599
Psme2	0.0000	2.4324	0.0029	0.0054	-0.3803	0.0583
Ptch1	0.0000	-2.0568	0.0017	0.0000	-0.9311	0.0144
Ptges3	0.0000	1.5863	0.0020	0.0000	0.9925	0.0087
Ptgs1	0.0000	-1.3896	0.0369	0.0500	-0.4954	0.0676
Ptprt	0.0000	1.2673	0.0180	0.7894	-0.0379	0.0945
Pwwp2b	0.0000	-1.5005	0.0247	0.6981	-0.0729	0.0923
Pxmp4	0.0000	-1.3695	0.0160	0.0000	-0.7801	0.0343
Qpct	0.0000	-2.4747	0.0148	0.5126	0.1313	0.0870
Qpctl	0.0000	-1.7641	0.0166	0.0036	-0.4959	0.0570
Qsox1	0.0000	-1.1777	0.0104	0.0438	-0.1896	0.0669

Qtrt1	0.0000	1.0964	0.0235	0.0000	0.9953	0.0281
Rab11fip3	0.0000	-1.0169	0.0125	0.0000	-0.8861	0.0177
Rab3il1	0.0000	-1.2951	0.0191	0.0000	0.6375	0.0413
Ralyl	0.0000	-1.8610	0.0218	0.0005	-0.7370	0.0517
Ramp3	0.0000	-1.5860	0.0380	0.5318	-0.1818	0.0875
Rara	0.0000	-1.1185	0.0128	0.0002	-0.3657	0.0497
Rassf10	0.0000	-2.0110	0.0277	0.3846	-0.2309	0.0833
Rassf8	0.0000	-1.2875	0.0170	0.0264	-0.2944	0.0644
Rbbp5	0.0000	1.1086	0.0101	0.0000	0.7553	0.0224
Rbbp9	0.0000	-1.5310	0.0074	0.0001	-0.4088	0.0476
Rbm10	0.0000	1.6062	0.0030	0.0000	0.3553	0.0455
Rbm24	0.0000	-2.4658	0.0202	0.0185	-0.5923	0.0628
Rbm3	0.0000	1.5495	0.0025	0.0000	0.8635	0.0133
Rccd1	0.0000	-1.4974	0.0206	0.0001	-0.6835	0.0467
Rcn3	0.0000	-1.4369	0.0101	0.8844	0.0168	0.0970
Rcsd1	0.0000	-2.0225	0.0123	0.5033	-0.1104	0.0868
Recql4	0.0000	1.1855	0.0349	0.0000	0.9386	0.0444
Rftn1	0.0000	1.2817	0.0277	0.0001	0.7434	0.0480
Rgs20	0.0000	-1.0172	0.0401	0.0000	-0.9701	0.0425
Rhobtb3	0.0000	-1.0357	0.0123	0.0000	-0.9675	0.0157
Rhoj	0.0000	-3.5616	0.0056	0.0011	-0.5962	0.0536
Rif1	0.0000	1.1060	0.0059	0.0000	0.9445	0.0101
Rin3	0.0001	-1.2380	0.0471	0.9738	0.0097	0.0994
Rnf144a	0.0000	-1.6285	0.0058	0.0000	-0.9674	0.0188
Rnf19a	0.0000	-1.0069	0.0137	0.0000	-0.8184	0.0210
Rnf213	0.0000	1.0330	0.0243	0.0000	0.8023	0.0344
Rora	0.0000	-1.2615	0.0279	0.1084	-0.2882	0.0723
Rpgr	0.0000	1.0101	0.0127	0.0000	0.5006	0.0369
Rpl14	0.0000	2.1213	0.0012	0.0000	0.7316	0.0204
Rpl15	0.0000	1.1867	0.0031	0.0000	0.2561	0.0464
Rpl17	0.0000	5.0805	0.0001	0.0000	0.7440	0.0252
Rpl18	0.0000	1.7376	0.0016	0.0000	0.8130	0.0138
Rpl19	0.0000	2.6082	0.0008	0.0000	0.4729	0.0371
Rpl23	0.0000	2.3286	0.0007	0.0000	0.4171	0.0362
Rpl24	0.0000	4.3456	0.0002	0.0585	-0.1768	0.0684
Rpl27a	0.0000	3.4602	0.0001	0.0003	0.2787	0.0507
Rpl29	0.0000	4.5979	0.0022	0.0038	0.6951	0.0572
Rpl3	0.0000	4.7606	0.0002	0.7000	-0.0363	0.0924
Rpl35	0.0000	2.3968	0.0011	0.0000	0.3782	0.0458
Rpl36	0.0000	1.8572	0.0020	0.0000	0.5603	0.0298
Rpl36a	0.0000	3.7047	0.0003	0.0000	0.7446	0.0242
Rpl5	0.0000	4.9308	0.0002	0.5969	-0.0477	0.0895
Rpl6	0.0000	1.8811	0.0017	0.0000	0.5188	0.0307
Rpl7a	0.0000	1.9619	0.0018	0.6189	-0.0426	0.0901
Rps10	0.0000	3.4464	0.0002	0.0000	0.5277	0.0323
Rps12	0.0000	4.4377	0.0001	0.0000	0.6233	0.0245
Rps14	0.0000	1.0752	0.0132	0.0000	0.6486	0.0306
Rps15a	0.0000	1.3419	0.0055	0.0906	-0.1477	0.0710
Rps17	0.0000	1.4403	0.0027	0.0000	0.8217	0.0133
Rps20	0.0000	1.2247	0.0079	0.0000	0.8619	0.0183
Rps23	0.0000	3.0145	0.0002	0.0009	0.2528	0.0530
Rps24	0.0000	1.0805	0.0049	0.0000	0.7235	0.0145
Rps27a	0.0000	3.8594	0.0002	0.0012	0.2612	0.0537
Rps27l	0.0000	1.0510	0.0093	0.0000	0.8266	0.0170
Rps6	0.0000	3.6331	0.0004	0.0000	0.7129	0.0194
Rps8	0.0000	1.9168	0.0012	0.0000	0.4669	0.0315
Rpsa	0.0000	1.2609	0.0048	0.0000	0.7766	0.0167
Rrp12	0.0000	1.0543	0.0139	0.0000	0.6177	0.0327
Rsph9	0.0000	1.4222	0.0383	0.0318	0.6262	0.0653
Rspo4	0.0000	3.3732	0.0413	0.8138	0.1942	0.0951
Rttn	0.0000	1.0860	0.0241	0.0000	0.6219	0.0450
Rusc2	0.0000	-1.3907	0.0086	0.0000	-0.8393	0.0228
Ruvbl1	0.0000	1.6956	0.0045	0.0000	0.5506	0.0370
Ruvbl2	0.0000	1.0467	0.0108	0.0000	0.9598	0.0147
Rxra	0.0000	-1.0214	0.0141	0.0000	-0.6334	0.0301
S1pr1	0.0000	-2.1237	0.0120	0.1338	-0.2483	0.0737
S1pr3	0.0000	1.1592	0.0239	0.0001	0.6093	0.0478
Sap18	0.0000	2.8724	0.0008	0.2427	-0.1209	0.0786
Sc5d	0.0000	-1.7897	0.0038	0.0000	-0.8303	0.0214
Scg2	0.0000	-1.0961	0.0099	0.0066	0.2321	0.0590
Scml2	0.0000	1.2182	0.0435	0.1034	0.4687	0.0720
Scn7a	0.0004	2.0994	0.0496	0.1362	-0.9641	0.0739
Sdc2	0.0000	-1.1014	0.0153	0.5695	0.0611	0.0886
Sdf2l1	0.0000	1.2375	0.0150	0.0000	0.8138	0.0300
Sdsl	0.0001	1.8977	0.0475	0.1774	0.6878	0.0758
Sec61g	0.0000	3.2328	0.0012	0.1586	0.1897	0.0750
Serping1	0.0000	-1.9002	0.0160	0.0119	-0.4628	0.0611

Sertm1	0.0001	-1.1841	0.0456	0.0191	-0.6578	0.0630
Sf3b3	0.0000	1.4517	0.0025	0.0000	0.7072	0.0173
Sfrp1	0.0000	-1.5837	0.0021	0.0000	0.7482	0.0154
Sft2d1	0.0000	1.4115	0.0188	0.9137	-0.0178	0.0978
Sgsm1	0.0000	-1.0087	0.0253	0.0000	-0.6213	0.0426
Sh3pxd2a	0.0000	-1.0762	0.0376	0.0210	-0.4937	0.0634
Sh3rf3	0.0000	-1.2919	0.0318	0.0003	-0.7507	0.0504
Sh3tc2	0.0001	1.7211	0.0466	0.0262	0.9887	0.0643
Shc2	0.0000	-1.2909	0.0115	0.0000	-0.7997	0.0267
Shroom3	0.0000	1.1474	0.0112	0.0544	0.1926	0.0681
Shroom4	0.0000	1.2029	0.0180	0.0000	0.9411	0.0273
Sidt1	0.0002	-2.5450	0.0482	0.2193	-0.7122	0.0776
Sirpa	0.0000	-1.2048	0.0116	0.9900	0.0013	0.0998
Sirt1	0.0000	1.1980	0.0110	0.0000	0.8901	0.0210
Sirt2	0.0000	-1.0018	0.0176	0.0000	-0.7206	0.0292
Sirt5	0.0000	1.2219	0.0312	0.6921	0.0814	0.0921
Ska1	0.0000	1.0252	0.0342	0.0000	0.9825	0.0369
Skap2	0.0000	-1.1322	0.0158	0.0000	-0.6492	0.0347
Slc11a2	0.0000	-1.1735	0.0156	0.0000	-0.5990	0.0377
Slc16a2	0.0000	1.9416	0.0055	0.9652	-0.0056	0.0992
Slc16a4	0.0000	2.0407	0.0391	0.0381	0.8953	0.0661
Slc18b1	0.0000	2.0542	0.0138	0.0001	0.7784	0.0482
Slc1a3	0.0000	-1.4340	0.0055	0.0008	-0.3023	0.0527
Slc1a4	0.0000	1.0095	0.0137	0.0000	0.8943	0.0187
Slc22a3	0.0000	-2.7602	0.0422	0.4006	0.4055	0.0838
Slc24a4	0.0000	-3.0886	0.0407	0.1502	-0.7677	0.0746
Slc2a10	0.0000	-2.3224	0.0359	0.8274	-0.0800	0.0955
Slc2a12	0.0000	1.2661	0.0281	0.9829	-0.0042	0.0997
Slc30a10	0.0000	-1.7058	0.0361	0.5505	-0.1737	0.0881
Slc30a3	0.0000	-2.1024	0.0250	0.0911	-0.4315	0.0711
Slc35e4	0.0000	-1.0501	0.0294	0.0000	-0.9876	0.0324
Slc38a3	0.0000	-2.5840	0.0164	0.0073	-0.6176	0.0593
Slc39a12	0.0000	3.4723	0.0096	0.1944	0.3967	0.0766
Slc45a3	0.0000	1.5494	0.0296	0.0973	0.4120	0.0716
Slc4a4	0.0000	-1.0147	0.0320	0.0000	-0.7581	0.0431
Slc6a17	0.0000	-1.6023	0.0285	0.0013	-0.7381	0.0539
Slc7a2	0.0000	-2.9068	0.0045	0.0000	0.8134	0.0328
Slc7a8	0.0000	1.3853	0.0334	0.3105	0.2532	0.0809
Slc8a3	0.0000	-1.1260	0.0322	0.0000	-0.9470	0.0387
Slc9a3r1	0.0000	1.2511	0.0109	0.0000	0.7559	0.0276
Slco2a1	0.0003	-1.2393	0.0490	0.1696	-0.4456	0.0755
Slit2	0.0000	-1.3235	0.0048	0.0000	-0.7250	0.0197
Slit3	0.0000	-1.0089	0.0246	0.0014	-0.4278	0.0541
Smad9	0.0001	-1.8751	0.0464	0.0517	0.8047	0.0678
Smap2	0.0000	-1.0590	0.0138	0.0000	-0.8814	0.0204
Smarca5	0.0000	1.1686	0.0037	0.0005	0.2256	0.0518
Smc4	0.0000	1.0104	0.0092	0.0000	0.7948	0.0169
Smpd4	0.0000	1.0684	0.0148	0.0000	0.7208	0.0288
Smyd4	0.0000	1.2378	0.0235	0.0000	0.8272	0.0388
Snai1	0.0000	-1.9322	0.0234	0.0247	-0.5122	0.0641
Snai2	0.0000	-2.2485	0.0289	0.2752	0.3303	0.0796
Sned1	0.0000	-2.4845	0.0079	0.0000	-0.9436	0.0365
Snhg16	0.0000	-1.2671	0.0386	0.0104	0.5979	0.0605
Snhg18	0.0000	-1.9601	0.0408	0.1766	0.4837	0.0758
Snhg5	0.0000	-1.2524	0.0159	0.0000	-0.7644	0.0325
Snrpd1	0.0000	1.0516	0.0098	0.0059	0.2362	0.0586
Snrpf	0.0000	1.1281	0.0123	0.0954	0.1725	0.0714
Snrpg	0.0000	2.7801	0.0023	0.0000	-0.6259	0.0444
Sntb1	0.0000	1.0676	0.0401	0.5887	0.1239	0.0892
Snx2	0.0000	1.0917	0.0097	0.0000	0.7669	0.0209
Snx21	0.0000	-1.4506	0.0281	0.0012	-0.6678	0.0537
Snx22	0.0000	-3.8141	0.0091	0.7952	-0.0567	0.0946
Sod3	0.0000	-3.6000	0.0230	0.1954	0.3957	0.0767
Sorcs2	0.0000	-1.8074	0.0056	0.0000	-0.9420	0.0221
Sost	0.0000	4.6917	0.0128	0.4518	0.3775	0.0853
Sparcl1	0.0000	-2.0158	0.0197	0.5220	0.1495	0.0873
Spata6	0.0000	1.0658	0.0240	0.0000	0.8731	0.0325
Speg	0.0000	-1.3599	0.0122	0.0000	-0.6643	0.0351
Spesp1	0.0000	2.5883	0.0401	0.1810	0.7697	0.0760
Spon2	0.0000	-2.2056	0.0182	0.0073	0.5730	0.0593
Spopl	0.0000	-1.0168	0.0167	0.6521	-0.0462	0.0910
Spry2	0.0000	1.2834	0.0078	0.0000	0.9690	0.0161
Spsb1	0.0000	-1.4823	0.0210	0.0374	0.3412	0.0660
Ssbp1	0.0000	1.3135	0.0120	0.0138	0.2924	0.0617
Ssc5d	0.0000	-1.5946	0.0150	0.0000	-0.7545	0.0388
St6galnac3	0.0000	-1.5196	0.0113	0.0000	-0.5574	0.0432
Stac2	0.0000	-1.2707	0.0347	0.0001	0.8086	0.0482

Stag2	0.0000	1.0596	0.0063	0.0000	0.6072	0.0221
Stxbp3	0.0000	1.1293	0.0233	0.0410	0.3051	0.0666
Stxbp6	0.0000	-1.6504	0.0095	0.0000	-0.8255	0.0297
Styk1	0.0000	3.4533	0.0278	0.7844	0.1562	0.0944
Sulf2	0.0000	-1.3347	0.0042	0.0000	-0.4795	0.0322
Sun2	0.0000	-1.0675	0.0139	0.0000	-0.5513	0.0357
Susd5	0.0000	-3.1072	0.0116	0.6502	0.0921	0.0909
Svep1	0.0000	-3.4481	0.0082	0.7400	-0.0716	0.0935
Svil	0.0000	-1.0951	0.0202	0.0189	-0.2982	0.0629
Syne1	0.0000	1.4719	0.0106	0.5646	0.0730	0.0884
Synm	0.0000	-1.3960	0.0153	0.1104	-0.2103	0.0724
Synpo	0.0000	-2.3436	0.0068	0.2962	0.1463	0.0803
Sytl2	0.0000	2.3931	0.0167	0.0017	-0.9057	0.0547
Tarbp1	0.0000	1.3566	0.0170	0.0000	0.8236	0.0350
Tars2	0.0000	1.0575	0.0201	0.0000	0.8283	0.0296
Tbc1d25	0.0000	1.3053	0.0177	0.0000	0.8643	0.0329
Tbkbp1	0.0000	-1.2722	0.0181	0.0000	-0.6994	0.0381
Tbx15	0.0000	-1.9078	0.0187	0.0037	-0.5832	0.0571
Tbx18	0.0000	-3.1735	0.0348	0.9090	0.0497	0.0976
Tbx2	0.0000	-2.9939	0.0215	0.0008	0.8939	0.0528
Tcea1	0.0000	1.4357	0.0038	0.0000	0.8752	0.0145
Tceal1	0.0000	-1.0471	0.0373	0.0000	-0.8980	0.0432
Tcta	0.0000	-1.3672	0.0172	0.0000	-0.9768	0.0281
Tdg	0.0000	2.1568	0.0019	0.0000	-0.5408	0.0370
Tead3	0.0000	-1.0257	0.0292	0.0000	-0.8871	0.0348
Tefm	0.0000	1.0061	0.0256	0.0000	0.7580	0.0365
Tent5c	0.0000	2.4608	0.0206	0.0012	0.9845	0.0537
Tex15	0.0000	2.9355	0.0170	0.0083	0.8313	0.0597
Tex30	0.0000	1.0686	0.0268	0.0000	0.9058	0.0335
Tfap2a	0.0000	1.2914	0.0245	0.0002	-0.6955	0.0489
Tfdp1	0.0000	1.2232	0.0058	0.0000	0.4562	0.0354
Tgfb1i1	0.0000	-1.9693	0.0180	0.0389	-0.4001	0.0662
Tgfbr2	0.0000	-1.8984	0.0115	0.0000	0.7744	0.0385
Tgm2	0.0000	-2.8074	0.0031	0.0002	0.5004	0.0498
Th	0.0002	1.0365	0.0479	0.0104	0.7038	0.0605
Tifa	0.0000	-3.6829	0.0182	0.8342	0.0583	0.0957
Timm23	0.0000	1.9431	0.0028	0.0000	0.8233	0.0227
Timp2	0.0000	-1.6534	0.0085	0.7455	-0.0397	0.0936
Timp3	0.0000	-1.2988	0.0411	0.0995	0.4673	0.0717
Tipin	0.0000	1.1594	0.0112	0.0000	0.4247	0.0453
Tjp2	0.0000	1.0722	0.0089	0.0000	0.8589	0.0159
Tk1	0.0000	1.1405	0.0248	0.0000	0.9151	0.0338
Tle2	0.0000	-3.1860	0.0243	0.1180	-0.5304	0.0729
Tmc7	0.0000	-1.8875	0.0169	0.1084	-0.2948	0.0723
Tmcc1	0.0000	-3.3038	0.0006	0.0025	0.2824	0.0560
Tmed3	0.0000	-1.0637	0.0237	0.2953	0.1384	0.0803
Tmed5	0.0000	-1.1948	0.0131	0.6139	-0.0530	0.0899
Tmem100	0.0000	-3.8848	0.0231	0.0125	-0.8946	0.0612
Tmem119	0.0000	-2.8173	0.0089	0.0086	-0.4874	0.0598
Tmem121	0.0000	-1.0005	0.0319	0.0002	-0.6020	0.0496
Tmem130	0.0000	-1.5638	0.0119	0.0000	-0.6925	0.0382
Tmem179	0.0000	-1.5930	0.0293	0.0012	-0.7489	0.0538
Tmem185l	0.0000	1.0746	0.0201	0.0000	0.7358	0.0342
Tmem204	0.0000	-2.6375	0.0449	0.3037	0.5022	0.0806
Tmem231	0.0000	1.0468	0.0160	0.0000	0.9494	0.0205
Tmem246	0.0000	-1.0810	0.0214	0.0000	-0.9968	0.0250
Tmem26	0.0001	1.5512	0.0471	0.2522	0.4700	0.0789
Tmem86a	0.0000	-1.4968	0.0260	0.4363	-0.1520	0.0849
Tmem98	0.0000	-1.1193	0.0275	0.0013	-0.5094	0.0540
Tmtc1	0.0000	-1.9376	0.0136	0.2075	-0.2203	0.0771
Tnfaip3	0.0000	-1.1228	0.0354	0.9491	-0.0126	0.0988
Tnfaip6	0.0002	-1.2865	0.0484	0.5267	0.2007	0.0874
Tnfrsf21	0.0000	-1.2753	0.0124	0.0182	-0.2619	0.0628
Tnfsf15	0.0001	-4.3266	0.0462	0.3847	-0.6423	0.0833
Tns1	0.0000	-1.3881	0.0074	0.0597	-0.1792	0.0685
Tom1	0.0000	2.1934	0.0056	0.0000	0.8174	0.0354
Tom1l2	0.0000	-1.3126	0.0100	0.0000	-0.9554	0.0196
Top2a	0.0000	1.1345	0.0090	0.0000	0.6418	0.0269
Tpbg	0.0000	1.0204	0.0188	0.0000	-0.8098	0.0292
Tpm3	0.0000	1.1164	0.0055	0.0006	0.2490	0.0520
Tppp3	0.0000	-1.7707	0.0049	0.0000	-0.8158	0.0242
Tpr	0.0000	1.2239	0.0039	0.0355	0.1461	0.0658
Tpt1	0.0000	1.3007	0.0023	0.0000	0.3003	0.0407
Trappc6a	0.0000	-1.1120	0.0242	0.0132	-0.3520	0.0615
Trdmt1	0.0000	1.1379	0.0353	0.0000	0.9055	0.0446
Trib2	0.0000	-1.2979	0.0084	0.0000	-0.9373	0.0177
Tril	0.0000	-1.3614	0.0156	0.2803	-0.1392	0.0798

Trmt11	0.0000	1.2191	0.0235	0.0000	0.8168	0.0388
Trnp1	0.0000	-1.5711	0.0399	0.0899	0.4880	0.0710
Trpc1	0.0000	-1.1480	0.0338	0.0000	-0.9797	0.0398
Tspan2	0.0000	-1.7345	0.0145	0.0403	-0.3168	0.0664
Tsr1	0.0000	1.1396	0.0074	0.0000	0.8099	0.0173
Ttc6	0.0000	-2.8595	0.0308	0.0465	0.6779	0.0672
Ttl	0.0000	-1.1541	0.0155	0.0000	-0.9792	0.0215
Tufm	0.0000	1.1568	0.0113	0.1063	-0.1654	0.0721
Tuft1	0.0000	1.6971	0.0210	0.0000	0.9477	0.0427
Twist2	0.0000	-5.0390	0.0173	0.8031	0.0797	0.0949
Txlnb	0.0000	1.3179	0.0253	0.6803	-0.0776	0.0918
Txndc16	0.0000	-1.0363	0.0163	0.0000	-0.8108	0.0250
U2af1	0.0000	3.0763	0.0007	0.0000	0.7921	0.0250
Uba2	0.0000	1.0015	0.0069	0.0000	0.4244	0.0332
Ube2i	0.0000	1.0260	0.0098	0.1697	-0.1155	0.0755
Ubxn2a	0.0000	1.2998	0.0108	0.0000	0.6494	0.0340
Uchl3	0.0000	1.9115	0.0043	0.0000	0.8327	0.0268
Uchl5	0.0000	1.6348	0.0052	0.0000	0.8327	0.0235
Unc13c	0.0004	-2.0095	0.0496	0.3079	0.4827	0.0807
Uqcrb	0.0000	1.5045	0.0076	0.0000	0.6101	0.0362
Urb1	0.0000	1.0906	0.0168	0.0000	0.4737	0.0464
Usp1	0.0000	1.1022	0.0077	0.0000	0.8147	0.0167
Usp13	0.0000	-1.0419	0.0298	0.0818	-0.2724	0.0704
Usp39	0.0000	1.2008	0.0110	0.0000	0.9482	0.0191
Usp50	0.0000	-4.4552	0.0155	0.6353	-0.1268	0.0906
Ust	0.0000	-1.1667	0.0324	0.0002	-0.7199	0.0492
Utp20	0.0000	1.4136	0.0045	0.0000	0.9053	0.0150
Utrn	0.0000	1.2054	0.0061	0.0000	0.9416	0.0128
Vamp1	0.0000	-1.0045	0.0415	0.0010	-0.7100	0.0532
Vcam1	0.0000	1.1560	0.0156	0.0000	0.9454	0.0234
Vcp	0.0000	1.0009	0.0063	0.0006	-0.2350	0.0521
Vdac3	0.0000	1.6458	0.0020	0.0028	0.2282	0.0563
Vsir	0.0000	-1.9336	0.0320	0.0098	-0.7623	0.0603
Vstm4	0.0000	-3.4838	0.0103	0.0130	-0.5726	0.0614
Vwa5b1	0.0000	1.8180	0.0299	0.1581	0.4208	0.0750
Vwa8	0.0000	1.0242	0.0223	0.0000	0.5450	0.0455
Wdr35	0.0000	1.2832	0.0064	0.0000	0.9679	0.0141
Wdr36	0.0000	1.0038	0.0103	0.0000	0.6537	0.0243
Wdr89	0.0000	-3.3858	0.0004	0.0000	0.6968	0.0199
Wfikkn2	0.0003	1.0281	0.0493	0.7683	-0.0851	0.0941
Wls	0.0000	1.1781	0.0098	0.0010	-0.3168	0.0533
Wnk4	0.0000	-1.4382	0.0315	0.0012	-0.7281	0.0539
Wnt3	0.0000	2.8278	0.0067	0.0143	0.5090	0.0618
Wnt6	0.0000	-2.0728	0.0178	0.7268	-0.0733	0.0931
Wnt8a	0.0002	2.7946	0.0486	0.2971	0.8211	0.0804
Wnt9a	0.0000	2.6222	0.0028	0.3968	0.1186	0.0837
Wrn	0.0000	1.0208	0.0260	0.0000	0.7439	0.0380
Xpo1	0.0000	1.0017	0.0062	0.0000	0.6652	0.0173
Xpot	0.0000	1.1187	0.0069	0.0000	0.6958	0.0206
Yjefn3	0.0000	1.2601	0.0418	0.1064	0.4576	0.0722
Ypel5	0.0000	-1.1296	0.0117	0.0000	-0.5310	0.0362
Zadh2	0.0000	-1.6964	0.0089	0.0000	-0.7988	0.0311
Zbtb20	0.0000	-1.0348	0.0169	0.0000	-0.9373	0.0209
Zbtb8os	0.0000	1.2170	0.0219	0.2008	-0.2007	0.0770
Zcchc12	0.0000	1.1240	0.0093	0.8670	0.0151	0.0966
Zcchc24	0.0000	-2.1759	0.0045	0.3904	0.1054	0.0835
Zdhhc23	0.0000	1.3253	0.0425	0.0017	0.9462	0.0547
Zfp28	0.0000	-1.0039	0.0326	0.0000	-0.9674	0.0347
Zfp30	0.0000	-1.2354	0.0175	0.2248	-0.1524	0.0778
Zic2	0.0000	1.7540	0.0069	0.0000	0.6608	0.0373
Zmym1	0.0000	1.1233	0.0140	0.0000	0.8738	0.0231

Genes of category 2

	Untreated *Tet/Tet* Neurons			Doxycycline-treated *Tet/Tet* Neurons		
Gene	*p* -value	Log_2 Fold change	Q-value (Benjamini-Hochberg)	*p* -value	Log_2 Fold change	Q-value (Benjamini-Hochberg)
Actc1	0.0000	-2.8334	0.0213	0.0000	1.2982	0.0418
Adcy8	0.0000	3.2214	0.0048	0.0000	-2.1277	0.0221
Aqp4	0.0001	-1.6662	0.0467	0.0000	2.1048	0.0312
Bgn	0.0000	-4.9638	0.0015	0.0000	2.5599	0.0020
Calb1	0.0003	1.4610	0.0489	0.0000	-1.9965	0.0446
Casp12	0.0000	-3.6714	0.0391	0.0000	1.7580	0.0448
Cfh	0.0000	-2.0521	0.0091	0.0000	1.1316	0.0226
Cldn11	0.0000	-2.8307	0.0141	0.0000	2.4290	0.0104
Cntnap2	0.0000	1.6792	0.0091	0.0000	-1.8762	0.0097
Col1a1	0.0000	-3.1526	0.0043	0.0000	1.1914	0.0306
Col1a2	0.0000	-1.9636	0.0212	0.0000	1.5852	0.0298

Col25a1	0.0001	-1.0812	0.0474	0.0000	1.1573	0.0431
Col6a2	0.0000	-1.5077	0.0115	0.0000	1.1502	0.0195
Corin	0.0000	-2.0664	0.0104	0.0000	2.3140	0.0056
Cped1	0.0000	-4.1030	0.0112	0.0000	1.1330	0.0341
Crym	0.0000	-1.9254	0.0345	0.0000	1.7976	0.0297
Dkk3	0.0000	-1.2450	0.0150	0.0000	1.8115	0.0057
Dnmt1	0.0000	1.8732	0.0021	0.0000	-5.8368	0.0002
Efna5	0.0000	1.4515	0.0124	0.0000	-1.4333	0.0159
Emp1	0.0000	-1.5325	0.0081	0.0000	1.5791	0.0074
Fbln2	0.0000	-1.1793	0.0276	0.0000	1.5920	0.0159
Fcgrt	0.0000	-1.2262	0.0302	0.0000	1.3236	0.0250
Fgl2	0.0000	-1.7785	0.0282	0.0000	1.2847	0.0337
Gabrg1	0.0000	1.6451	0.0450	0.0000	-2.5824	0.0391
Grasp	0.0000	-1.1483	0.0294	0.0000	1.2175	0.0249
Gsx2	0.0000	-3.2187	0.0450	0.0000	2.1045	0.0423
Hpgds	0.0000	1.8764	0.0370	0.0000	-2.2115	0.0417
Hrc	0.0000	-2.6796	0.0142	0.0000	1.1444	0.0350
Itga8	0.0000	-3.3615	0.0053	0.0000	1.1721	0.0236
Kcne4	0.0000	-4.1098	0.0176	0.0000	1.1489	0.0450
Khdrbs2	0.0000	1.0556	0.0375	0.0000	-2.3457	0.0183
Lgi3	0.0000	-4.8741	0.0182	0.0001	1.0055	0.0478
Lingo2	0.0000	2.5357	0.0162	0.0000	-2.5839	0.0274
Meox1	0.0000	-2.8451	0.0405	0.0000	2.7933	0.0234
Nphs1	0.0000	-2.1440	0.0025	0.0000	1.1186	0.0122
Onecut3	0.0000	1.1263	0.0317	0.0000	-2.7588	0.0124
Pi15	0.0000	-5.6550	0.0174	0.0000	1.3599	0.0401
Prrx2	0.0000	-1.5585	0.0402	0.0000	1.3799	0.0404
Ramp2	0.0000	-2.2108	0.0183	0.0000	1.0605	0.0368
Srgn	0.0001	-1.0607	0.0469	0.0000	1.1223	0.0425
Srpx2	0.0001	-3.0114	0.0476	0.0000	2.2706	0.0403
Tgfbi	0.0001	-1.2266	0.0474	0.0000	2.8090	0.0201
Thbd	0.0000	-1.6091	0.0312	0.0000	2.0150	0.0183
Tll2	0.0000	1.2506	0.0435	0.0000	-4.1343	0.0201
Tmem200b	0.0001	-2.3805	0.0462	0.0000	1.8104	0.0450
Tnnt2	0.0000	-2.6345	0.0308	0.0000	2.0640	0.0297
Tnxb	0.0000	-3.1928	0.0351	0.0000	1.8922	0.0365
Ttr	0.0000	7.7546	0.0003	0.0000	-1.8387	0.0396
Unc5c	0.0000	1.1517	0.0250	0.0000	-2.1903	0.0100
Vat1l	0.0000	1.6577	0.0047	0.0000	-1.8287	0.0052
Wnt2b	0.0000	2.3010	0.0196	0.0000	-1.3477	0.0439

Genes of category 3

	Untreated Tet/Tet Neurons			Doxycycline-treated *Tet/Tet* Neurons		
Gene	*p* -value	Log_2 Fold change	Q-value (Benjamini-Hochberg)	*p* -value	Log_2 Fold change	Q-value (Benjamini-Hochberg)
A2m	0.0000	1.3834	0.0218	0.0000	1.6475	0.0177
A4galt	0.0000	2.0868	0.0133	0.0000	3.1343	0.0056
Aaas	0.0000	1.1296	0.0149	0.0000	1.1041	0.0171
Aard	0.0000	2.3622	0.0280	0.0000	3.9240	0.0139
Aars	0.0000	1.2328	0.0033	0.0000	1.5920	0.0017
Aass	0.0000	4.2593	0.0258	0.0000	4.1261	0.0282
Aatk	0.0000	-2.4783	0.0102	0.0000	-2.1719	0.0141
Abcb9	0.0000	-1.7035	0.0333	0.0000	-1.6898	0.0343
Abcc4	0.0000	2.1755	0.0039	0.0000	3.3658	0.0009
Abhd18	0.0000	-1.0096	0.0323	0.0000	-1.4786	0.0213
Abi3	0.0002	3.2770	0.0482	0.0000	4.1545	0.0407
Acacb	0.0000	1.4842	0.0215	0.0000	2.1693	0.0115
Acap1	0.0000	5.9884	0.0163	0.0000	5.3658	0.0213
Acap3	0.0000	-1.3965	0.0091	0.0000	-1.7974	0.0057
Acat2	0.0000	-1.3678	0.0036	0.0000	-1.5093	0.0031
Acbd4	0.0000	1.0617	0.0339	0.0000	1.9324	0.0153
Accsl	0.0000	5.8054	0.0190	0.0000	5.4716	0.0224
Ace	0.0000	2.4789	0.0149	0.0000	1.3856	0.0360
Ache	0.0000	-1.9486	0.0109	0.0000	-2.3388	0.0083
Ackr3	0.0000	-1.7244	0.0132	0.0000	-1.6276	0.0161
Acot9	0.0000	1.3470	0.0137	0.0000	1.2366	0.0176
Acp5	0.0004	1.4338	0.0498	0.0000	3.5973	0.0182
Acp6	0.0000	1.9232	0.0036	0.0000	2.0126	0.0036
Acpp	0.0000	5.6302	0.0076	0.0000	1.8332	0.0458
Acsl1	0.0000	1.2906	0.0121	0.0000	1.4184	0.0112
Acsl6	0.0000	-1.1138	0.0207	0.0000	-1.4575	0.0143
Acss3	0.0000	4.7799	0.0014	0.0000	3.5808	0.0040
Actl6b	0.0000	-2.2584	0.0070	0.0000	-3.1595	0.0037
Actn1	0.0000	1.0264	0.0083	0.0000	1.2962	0.0053
Actr1a	0.0000	-1.2143	0.0048	0.0000	-1.1687	0.0063
Acvr2a	0.0000	-1.0982	0.0120	0.0000	-1.8096	0.0041
Adad2	0.0000	3.8305	0.0447	0.0000	4.7267	0.0380

Adam11	0.0000	-1.7743		0.0125	0.0000	-2.1638	0.0093
Adam23	0.0000	1.1776		0.0072	0.0000	1.3629	0.0059
Adamts16	0.0000	-2.9759		0.0095	0.0000	-2.9053	0.0106
Adamts18	0.0000	-4.8402		0.0192	0.0000	-2.8561	0.0279
Adamts6	0.0000	-1.2040		0.0388	0.0000	-1.6194	0.0302
Adamts7	0.0000	-1.5712		0.0113	0.0000	-1.7961	0.0096
Adap2	0.0001	3.0790		0.0455	0.0000	3.4725	0.0423
Adarb2	0.0000	-1.0799		0.0393	0.0000	-2.5150	0.0152
Adcy2	0.0000	-2.3451		0.0077	0.0000	-3.1516	0.0044
Adcy5	0.0000	-1.9614		0.0053	0.0000	-1.5364	0.0102
Adcyap1r1	0.0000	-2.6213		0.0019	0.0000	-2.3456	0.0029
Add2	0.0000	-1.9703		0.0028	0.0000	-3.0928	0.0006
Adgrb1	0.0000	-1.6708		0.0080	0.0000	-1.3717	0.0134
Adgrb2	0.0000	-1.2301		0.0067	0.0000	-1.2492	0.0076
Adgre5	0.0000	1.4061		0.0147	0.0000	3.5406	0.0010
Adgrg6	0.0000	-1.5091		0.0227	0.0000	-1.0274	0.0362
Adgrl3	0.0000	-1.0797		0.0164	0.0000	-2.1505	0.0040
Adgrv1	0.0000	-1.4658		0.0075	0.0000	-1.8902	0.0045
Adra1d	0.0000	-3.9821		0.0250	0.0000	-2.2378	0.0390
Adra2a	0.0000	-3.2609		0.0063	0.0000	-1.9861	0.0160
Afap1	0.0000	-1.4232		0.0057	0.0000	-1.9601	0.0027
Afap1l2	0.0000	1.1448		0.0433	0.0000	1.1833	0.0432
Aff1	0.0000	1.7957		0.0035	0.0000	1.7583	0.0044
Aff2	0.0000	-2.3594		0.0125	0.0000	-3.6133	0.0066
Aga	0.0000	2.0814		0.0064	0.0000	2.5112	0.0045
Agap2	0.0000	-1.4314		0.0124	0.0000	-1.8698	0.0080
Ago4	0.0000	-1.1809		0.0246	0.0000	-2.4148	0.0081
Agpat2	0.0000	1.8304		0.0168	0.0000	2.1829	0.0131
Ahnak2	0.0000	1.1141		0.0310	0.0000	3.0045	0.0048
Ahrr	0.0000	4.6302		0.0368	0.0000	4.2500	0.0415
Ahsa1	0.0000	1.1832		0.0085	0.0000	1.3928	0.0066
Aifm3	0.0000	3.8754		0.0119	0.0000	1.9244	0.0369
Aim2	0.0000	3.8881		0.0033	0.0000	4.3310	0.0027
Aire	0.0000	4.8170		0.0099	0.0000	4.7077	0.0120
Ajap1	0.0000	-1.1031		0.0353	0.0000	-2.4873	0.0130
Ajuba	0.0000	1.2121		0.0078	0.0000	1.4863	0.0053
Ak7	0.0000	2.7320		0.0198	0.0000	1.3671	0.0456
Akap6	0.0000	-1.6809		0.0028	0.0000	-2.8418	0.0004
Akip1	0.0000	1.0087		0.0360	0.0000	1.5203	0.0229
Alcam	0.0000	-2.2460		0.0191	0.0000	-1.6308	0.0310
Aldh1a2	0.0000	-3.9431		0.0030	0.0000	-2.7140	0.0073
Aldh1l1	0.0000	1.5215		0.0199	0.0000	2.4592	0.0083
Aldh2	0.0000	1.3550		0.0051	0.0000	1.6867	0.0031
Alg13	0.0000	2.8457		0.0009	0.0000	2.8575	0.0008
Alk	0.0001	-1.5579		0.0468	0.0000	-2.6822	0.0310
Alkbh2	0.0000	1.8007		0.0208	0.0000	2.1825	0.0162
Alpk3	0.0000	1.2933		0.0342	0.0000	3.8468	0.0044
Alpl	0.0000	2.4354		0.0018	0.0000	3.0318	0.0008
Als2cl	0.0000	1.7899		0.0284	0.0000	2.7198	0.0159
Amer3	0.0000	-1.4874		0.0199	0.0000	-2.5583	0.0084
Amigo1	0.0000	-1.1941		0.0202	0.0000	-1.6698	0.0124
Angptl4	0.0000	4.2708		0.0123	0.0000	4.6738	0.0115
Ank1	0.0000	-1.5483		0.0319	0.0000	-1.1548	0.0422
Ank2	0.0000	-1.4325		0.0041	0.0000	-2.0611	0.0015
Ank3	0.0000	-1.2627		0.0044	0.0000	-1.9286	0.0012
Ankrd13b	0.0000	-1.3179		0.0117	0.0000	-2.0369	0.0049
Ankrd35	0.0000	2.5398		0.0239	0.0000	4.4954	0.0088
Ankrd45	0.0000	2.5591		0.0162	0.0000	3.3022	0.0105
Ankrd50	0.0000	-1.3569		0.0050	0.0000	-1.2468	0.0073
Ankrd6	0.0000	-1.0955		0.0157	0.0000	-1.0472	0.0183
Ankrd9	0.0000	1.0117		0.0446	0.0000	1.5993	0.0293
Anks1b	0.0000	-1.6137		0.0221	0.0000	-1.9146	0.0184
Anln	0.0000	1.8509		0.0021	0.0000	1.8047	0.0027
Ano1	0.0000	3.5247		0.0033	0.0000	1.0830	0.0366
Anp32b	0.0000	1.0144		0.0110	0.0000	1.1209	0.0101
Anxa1	0.0000	1.2318		0.0214	0.0000	1.6995	0.0133
Anxa11	0.0000	2.9313		0.0016	0.0000	3.2099	0.0011
Anxa3	0.0000	1.4541		0.0219	0.0000	1.2429	0.0286
Anxa4	0.0000	2.5631		0.0027	0.0000	2.0676	0.0062
Anxa7	0.0000	2.1774		0.0025	0.0000	1.9089	0.0045
Aoah	0.0000	4.6244		0.0349	0.0000	5.4742	0.0304
Ap1g2	0.0000	1.3003		0.0368	0.0000	1.4578	0.0340
Ap1m2	0.0000	2.7442		0.0264	0.0000	2.1911	0.0356
Apba1	0.0000	-1.7331		0.0092	0.0000	-2.0597	0.0070
Apba2	0.0000	-1.3977		0.0092	0.0000	-2.8545	0.0015
Apbb1	0.0000	-1.2390		0.0072	0.0000	-1.4569	0.0056
Apbb1ip	0.0000	3.8416		0.0373	0.0000	4.6598	0.0319

Apc2	0.0000	-1.5983	0.0034	0.0000	-3.0121	0.0003
Aph1b	0.0000	-1.4865	0.0118	0.0000	-1.3735	0.0151
Apobec2	0.0000	2.4227	0.0264	0.0000	4.1830	0.0107
Apobr	0.0000	2.0221	0.0421	0.0000	2.6225	0.0337
Apoc1	0.0000	3.8916	0.0109	0.0000	4.7598	0.0078
Apoe	0.0000	1.8470	0.0035	0.0000	4.2302	0.0002
Aqp3	0.0000	2.0632	0.0153	0.0000	4.2158	0.0026
Arc	0.0000	3.3207	0.0071	0.0000	4.0484	0.0048
Arg2	0.0001	-1.7163	0.0464	0.0000	-2.6619	0.0337
Arhgap20	0.0000	-2.1183	0.0125	0.0000	-2.1204	0.0138
Arhgap27	0.0002	1.3155	0.0479	0.0000	1.9920	0.0340
Arhgap28	0.0000	-1.9305	0.0073	0.0000	-1.0137	0.0252
Arhgap30	0.0000	3.7533	0.0090	0.0000	3.3452	0.0132
Arhgap33	0.0000	-1.3506	0.0071	0.0000	-2.3782	0.0016
Arhgap36	0.0000	-3.8720	0.0097	0.0000	-4.1039	0.0091
Arhgap4	0.0001	1.3650	0.0477	0.0000	1.5139	0.0448
Arhgap6	0.0000	-2.0583	0.0392	0.0000	-2.3425	0.0359
Arhgap9	0.0000	3.0695	0.0307	0.0000	4.1537	0.0216
Arhgdib	0.0000	2.4240	0.0152	0.0000	2.5204	0.0156
Arhgef10l	0.0000	-1.2391	0.0130	0.0000	-1.9517	0.0054
Arhgef15	0.0000	1.7457	0.0396	0.0000	2.5138	0.0276
Arhgef16	0.0000	1.6909	0.0317	0.0000	1.4036	0.0397
Arhgef18	0.0000	2.2721	0.0008	0.0000	1.2779	0.0056
Arhgef3	0.0000	2.0184	0.0116	0.0000	1.8818	0.0150
Arhgef4	0.0000	-2.0528	0.0058	0.0000	-1.1217	0.0213
Arid3b	0.0000	1.1712	0.0142	0.0000	1.5242	0.0091
Arid5a	0.0000	1.3679	0.0393	0.0000	2.2845	0.0224
Arl4d	0.0000	-3.1589	0.0041	0.0000	-1.7087	0.0144
Armc3	0.0000	3.8458	0.0283	0.0000	3.2328	0.0360
Armcx4	0.0000	-1.1627	0.0063	0.0000	-1.4145	0.0045
Arntl	0.0000	1.3605	0.0257	0.0000	1.1429	0.0330
Arpc1b	0.0000	1.1348	0.0158	0.0000	2.0888	0.0041
Arrb1	0.0000	-1.4811	0.0053	0.0000	-1.7892	0.0036
Asb13	0.0000	-1.6647	0.0133	0.0000	-1.5201	0.0166
Ascl2	0.0000	2.9413	0.0274	0.0000	2.1674	0.0397
Asf1b	0.0000	1.3911	0.0172	0.0000	1.1947	0.0234
Ash2l	0.0000	1.5663	0.0022	0.0000	1.8453	0.0014
Asic1	0.0000	-2.2512	0.0017	0.0000	-2.4562	0.0012
Asic4	0.0000	-2.0473	0.0049	0.0000	-2.8071	0.0024
Asna1	0.0000	1.3533	0.0077	0.0000	1.7026	0.0049
Asns	0.0000	2.4300	0.0007	0.0000	2.5217	0.0004
Aspa	0.0003	2.7669	0.0495	0.0000	3.1759	0.0456
Asphd1	0.0000	-2.1348	0.0289	0.0000	-2.3724	0.0264
Ass1	0.0000	4.0842	0.0005	0.0000	3.9807	0.0003
Astn1	0.0000	-1.0207	0.0144	0.0000	-2.0955	0.0029
Astn2	0.0000	-2.1253	0.0075	0.0000	-1.5730	0.0145
Asxl3	0.0000	-1.6481	0.0083	0.0000	-3.3444	0.0015
Atad2	0.0000	1.5520	0.0042	0.0000	1.0234	0.0136
Atat1	0.0000	-1.3300	0.0071	0.0000	-1.9218	0.0031
Atcay	0.0000	-2.1841	0.0026	0.0000	-2.4665	0.0020
Atf1	0.0000	1.4939	0.0076	0.0000	1.6662	0.0067
Atf5	0.0000	2.5569	0.0007	0.0000	2.6907	0.0003
Atf7ip2	0.0000	3.2686	0.0175	0.0000	3.6352	0.0157
Atic	0.0000	1.7421	0.0026	0.0000	1.5295	0.0046
Atmin	0.0000	1.0578	0.0120	0.0000	1.1163	0.0122
Atp10a	0.0000	1.1791	0.0353	0.0000	1.4721	0.0286
Atp10d	0.0000	2.7916	0.0026	0.0000	1.6854	0.0120
Atp13a2	0.0000	-1.1516	0.0086	0.0000	-1.2015	0.0088
Atp1a3	0.0000	-1.1616	0.0106	0.0000	-1.0485	0.0148
Atp2a3	0.0000	1.2405	0.0311	0.0000	2.5781	0.0099
Atp2b2	0.0000	-1.5945	0.0162	0.0000	-1.3149	0.0229
Atp6v0a4	0.0000	3.6554	0.0313	0.0000	3.6574	0.0329
Atp6v1g2	0.0000	-2.2432	0.0155	0.0000	-1.5001	0.0275
Atp8a1	0.0000	-1.1768	0.0139	0.0000	-1.3120	0.0125
Atp8a2	0.0000	-1.6321	0.0140	0.0000	-2.8747	0.0048
Aurka	0.0000	1.3112	0.0114	0.0000	1.3797	0.0117
Auts2	0.0000	-1.5520	0.0041	0.0000	-1.9154	0.0026
Avl9	0.0000	-1.2678	0.0073	0.0000	-1.4369	0.0063
Avpi1	0.0000	1.5041	0.0127	0.0000	2.8698	0.0025
B3galt2	0.0000	-1.6259	0.0343	0.0001	-1.0951	0.0480
B3galt4	0.0000	-1.3099	0.0407	0.0002	-1.0091	0.0497
B3galt5	0.0000	-3.1325	0.0289	0.0000	-2.9937	0.0298
B3gat1	0.0000	-1.5325	0.0133	0.0000	-1.8134	0.0104
B3gnt2	0.0000	2.8891	0.0037	0.0000	3.6135	0.0021
B3gnt7	0.0000	3.3354	0.0013	0.0000	4.1940	0.0004
B4galnt3	0.0000	2.5185	0.0160	0.0000	2.5857	0.0167
B4galt1	0.0000	1.0982	0.0158	0.0000	1.2825	0.0131

B4galt2	0.0000	-1.9227	0.0044	0.0000	-1.6723	0.0073
Bace1	0.0000	-1.5078	0.0052	0.0000	-1.1963	0.0101
Bach2	0.0000	-2.0741	0.0036	0.0000	-2.7211	0.0018
Bambi	0.0000	3.5861	0.0024	0.0000	1.2240	0.0289
Bard1	0.0000	1.9504	0.0050	0.0000	2.1515	0.0044
Basp1	0.0000	-1.5163	0.0051	0.0000	-1.5987	0.0053
Bcam	0.0000	1.1676	0.0165	0.0000	1.8091	0.0071
Bcar3	0.0000	1.2873	0.0152	0.0000	1.9094	0.0072
Bcas1	0.0000	1.9532	0.0345	0.0000	2.9089	0.0218
Bcat1	0.0000	1.8167	0.0042	0.0000	1.9449	0.0041
Bcat2	0.0000	1.6912	0.0082	0.0000	2.0368	0.0058
Bckdhb	0.0000	1.2502	0.0151	0.0000	1.5147	0.0112
Bcl11a	0.0000	-1.6892	0.0074	0.0000	-2.1182	0.0048
Bcl11b	0.0000	-2.1848	0.0123	0.0000	-1.4047	0.0258
Bcl3	0.0000	1.8321	0.0334	0.0000	3.4306	0.0138
Bcl7a	0.0000	-1.4495	0.0043	0.0000	-2.0102	0.0019
Bdh2	0.0002	1.2166	0.0480	0.0000	1.9472	0.0322
Bean1	0.0000	-2.0998	0.0278	0.0000	-2.7736	0.0209
Begain	0.0000	-1.7234	0.0161	0.0000	-1.8960	0.0148
Bend5	0.0000	-1.6457	0.0135	0.0000	-1.7555	0.0130
Bend7	0.0000	-2.5484	0.0156	0.0000	-1.2041	0.0373
Bex1	0.0000	1.0849	0.0143	0.0000	1.6043	0.0069
Bex4	0.0000	1.1859	0.0100	0.0000	1.1620	0.0119
Bhlhe22	0.0000	-4.9065	0.0008	0.0000	-2.8907	0.0022
Bicd1	0.0000	-1.3310	0.0130	0.0000	-1.5065	0.0111
Bicd2	0.0000	-1.0005	0.0128	0.0000	-1.4546	0.0063
Bin2	0.0000	4.3355	0.0066	0.0000	5.2882	0.0045
Blcap	0.0000	-1.3454	0.0051	0.0000	-1.3792	0.0056
Blnk	0.0000	4.8076	0.0353	0.0000	4.3487	0.0404
Bmp1	0.0000	-2.0890	0.0020	0.0000	-1.3984	0.0070
Bok	0.0000	1.4426	0.0256	0.0000	1.6256	0.0228
Brca1	0.0000	1.4212	0.0079	0.0000	1.1847	0.0134
Brinp1	0.0000	-2.2549	0.0140	0.0000	-3.0208	0.0091
Brip1	0.0000	1.3655	0.0196	0.0000	1.1056	0.0278
Brsk1	0.0000	-1.3902	0.0061	0.0000	-1.8536	0.0033
Brsk2	0.0000	-1.3969	0.0072	0.0000	-2.8475	0.0010
Bsn	0.0000	-2.3848	0.0024	0.0000	-3.6199	0.0007
Bst2	0.0000	2.4028	0.0041	0.0000	2.4854	0.0043
Btbd19	0.0000	1.4056	0.0266	0.0000	1.7903	0.0196
Btg3	0.0000	1.7380	0.0074	0.0000	1.0557	0.0222
Bub1	0.0000	1.6678	0.0033	0.0000	1.4891	0.0056
Bub1b	0.0000	1.5068	0.0047	0.0000	1.3785	0.0072
C1ql3	0.0000	-2.1177	0.0332	0.0000	-3.4313	0.0214
C2	0.0000	2.1294	0.0300	0.0000	3.8299	0.0123
Cacna1c	0.0000	-1.7397	0.0108	0.0000	-2.0284	0.0088
Cacna1g	0.0000	-1.0207	0.0176	0.0000	-1.4449	0.0100
Cacna1h	0.0000	-2.0122	0.0029	0.0000	-2.1162	0.0028
Cacna2d2	0.0000	-2.0788	0.0040	0.0000	-2.0858	0.0044
Cacna2d3	0.0000	-2.5162	0.0059	0.0000	-3.2583	0.0036
Cacnb1	0.0000	-1.2855	0.0158	0.0000	-2.0168	0.0071
Cacnb4	0.0000	-1.1757	0.0360	0.0000	-2.2014	0.0183
Cacng2	0.0000	-1.9545	0.0136	0.0000	-2.4694	0.0095
Cacng4	0.0000	-1.0006	0.0136	0.0000	-2.2011	0.0021
Cacng6	0.0000	2.9742	0.0297	0.0000	4.3766	0.0181
Cadm2	0.0000	-2.2821	0.0113	0.0000	-2.8341	0.0081
Cadm3	0.0000	-2.0541	0.0032	0.0000	-2.2237	0.0029
Cadm4	0.0000	-1.9833	0.0020	0.0000	-2.2060	0.0016
Cadps	0.0000	-1.6798	0.0066	0.0000	-2.9023	0.0017
Cadps2	0.0000	2.6007	0.0103	0.0000	1.4558	0.0300
Calcoco2	0.0000	2.8959	0.0057	0.0000	4.2680	0.0019
Calhm2	0.0000	2.5615	0.0379	0.0000	3.4285	0.0287
Calml4	0.0000	3.5106	0.0087	0.0000	2.5241	0.0191
Camk1d	0.0000	1.3881	0.0125	0.0000	1.4126	0.0136
Camk2a	0.0000	-1.6329	0.0196	0.0000	-1.6907	0.0196
Camk2n2	0.0000	-1.7948	0.0178	0.0000	-1.1330	0.0328
Camk4	0.0000	-1.4508	0.0337	0.0000	-1.3226	0.0374
Camta1	0.0000	-1.5891	0.0052	0.0000	-1.7475	0.0047
Capg	0.0000	4.1306	0.0093	0.0000	4.7844	0.0075
Capn1	0.0000	1.7159	0.0088	0.0000	2.2724	0.0048
Capn3	0.0000	3.6965	0.0256	0.0000	3.5715	0.0281
Capsl	0.0000	2.6825	0.0188	0.0000	2.9876	0.0169
Carnmt1	0.0000	2.1286	0.0030	0.0000	1.5432	0.0088
Cars	0.0000	1.4595	0.0042	0.0000	1.4891	0.0047
Casp8	0.0000	2.2160	0.0237	0.0000	2.4343	0.0219
Catip	0.0000	4.0335	0.0263	0.0000	3.3088	0.0346
Catsperd	0.0000	3.4157	0.0190	0.0000	2.7923	0.0272
Cav2	0.0000	2.1194	0.0435	0.0000	2.7234	0.0354

Cbarp	0.0000	-1.3059	0.0100	0.0000	-1.2094	0.0130
Cbln2	0.0000	-4.5380	0.0073	0.0000	-4.4498	0.0073
Cbln4	0.0000	-2.7194	0.0158	0.0000	-1.7937	0.0270
Cbr3	0.0000	1.8401	0.0360	0.0000	3.7696	0.0132
Cbs	0.0000	1.9796	0.0166	0.0000	2.1427	0.0158
Cbx7	0.0000	1.0745	0.0214	0.0000	3.6829	0.0007
Cc2d1b	0.0000	1.1354	0.0177	0.0000	1.1597	0.0186
Ccbe1	0.0000	-1.5630	0.0384	0.0000	-2.4621	0.0255
Ccdc113	0.0000	5.2344	0.0214	0.0000	4.9417	0.0250
Ccdc114	0.0000	2.4060	0.0157	0.0000	2.7511	0.0133
Ccdc125	0.0000	2.3538	0.0292	0.0000	3.4932	0.0173
Ccdc136	0.0000	-1.3600	0.0132	0.0000	-1.6239	0.0102
Ccdc141	0.0000	2.6791	0.0130	0.0000	2.8473	0.0129
Ccdc170	0.0000	2.8146	0.0451	0.0000	3.2309	0.0412
Ccdc18	0.0000	1.3479	0.0220	0.0000	1.1457	0.0289
Ccdc184	0.0000	-1.1127	0.0306	0.0000	-2.7897	0.0080
Ccdc24	0.0000	-2.2644	0.0340	0.0000	-1.7893	0.0418
Ccdc28b	0.0000	-2.4767	0.0043	0.0000	-2.0916	0.0069
Ccdc36	0.0000	3.9757	0.0242	0.0000	4.0969	0.0248
Ccdc68	0.0000	3.4614	0.0310	0.0000	4.9532	0.0202
Ccdc86	0.0000	1.4919	0.0099	0.0000	1.4114	0.0126
Cck	0.0000	6.1805	0.0134	0.0000	4.7026	0.0235
Ccnb1	0.0000	1.8756	0.0038	0.0000	1.2376	0.0126
Ccnb1ip1	0.0000	3.4726	0.0126	0.0000	3.8530	0.0114
Ccnb2	0.0000	1.5594	0.0078	0.0000	1.6088	0.0084
Ccnf	0.0000	1.4239	0.0078	0.0000	1.7698	0.0052
Ccng2	0.0000	-1.2308	0.0071	0.0000	-1.3350	0.0067
Ccnjl	0.0000	-1.0193	0.0320	0.0000	-2.1616	0.0117
Ccser1	0.0000	-1.3077	0.0261	0.0000	-2.7876	0.0085
Cd2ap	0.0000	1.3412	0.0069	0.0000	1.2835	0.0089
Cd38	0.0000	3.2838	0.0139	0.0000	3.9224	0.0107
Cd44	0.0000	1.8398	0.0100	0.0000	1.7536	0.0127
Cd55	0.0000	2.2441	0.0089	0.0000	2.2402	0.0101
Cd68	0.0000	2.5250	0.0248	0.0000	4.1546	0.0110
Cd74	0.0000	4.2692	0.0327	0.0000	3.2768	0.0438
Cd83	0.0000	-1.7922	0.0392	0.0000	-1.6524	0.0424
Cd84	0.0000	7.0224	0.0287	0.0000	6.4706	0.0332
Cd9	0.0000	1.7080	0.0039	0.0000	2.7697	0.0007
Cdc14b	0.0000	1.2120	0.0180	0.0000	1.2626	0.0182
Cdc20	0.0000	1.4245	0.0057	0.0000	1.2720	0.0090
Cdc42ep2	0.0000	-1.6905	0.0305	0.0000	-1.3771	0.0375
Cdc42ep4	0.0000	-1.2897	0.0094	0.0000	-1.1815	0.0128
Cdca2	0.0000	1.4247	0.0073	0.0000	1.3391	0.0097
Cdca3	0.0000	1.0737	0.0222	0.0000	1.2664	0.0188
Cdca5	0.0000	1.5663	0.0098	0.0000	1.6749	0.0096
Cdca7	0.0000	1.1064	0.0131	0.0000	1.1539	0.0136
Cdcp1	0.0000	3.0754	0.0258	0.0000	2.7755	0.0307
Cdh1	0.0000	3.6049	0.0004	0.0000	3.5969	0.0001
Cdh10	0.0000	-2.0802	0.0119	0.0000	-2.5021	0.0092
Cdh13	0.0000	-1.6054	0.0105	0.0000	-1.3008	0.0171
Cdh18	0.0001	-1.3479	0.0464	0.0000	-2.2633	0.0311
Cdh3	0.0000	2.9376	0.0051	0.0000	2.8214	0.0067
Cdh6	0.0000	-1.9608	0.0037	0.0000	-1.7931	0.0053
Cdh7	0.0000	-2.7510	0.0256	0.0000	-3.5950	0.0199
Cdh8	0.0000	-2.6868	0.0125	0.0000	-4.2293	0.0069
Cdhr1	0.0000	2.4048	0.0198	0.0000	3.3477	0.0114
Cdk1	0.0000	1.2934	0.0098	0.0000	1.3253	0.0106
Cdk17	0.0000	-1.0045	0.0161	0.0000	-1.1846	0.0131
Cdk18	0.0000	1.8247	0.0368	0.0000	2.2443	0.0306
Cdk5r1	0.0000	-1.9383	0.0048	0.0000	-2.4778	0.0028
Cdk5rap1	0.0000	1.2285	0.0227	0.0000	1.2219	0.0243
Cdkn1c	0.0000	-1.6195	0.0027	0.0000	-1.3585	0.0055
Cdkn2a	0.0000	2.0049	0.0073	0.0000	3.4181	0.0016
Cds1	0.0000	1.2381	0.0256	0.0000	1.8270	0.0144
Cdx1	0.0000	5.5544	0.0148	0.0000	2.5665	0.0432
Cdyl2	0.0000	2.6260	0.0068	0.0000	2.9330	0.0060
Ceacam1	0.0000	4.0625	0.0199	0.0000	4.5507	0.0179
Cebpb	0.0000	1.1113	0.0324	0.0000	2.8258	0.0065
Celf3	0.0000	-1.8419	0.0051	0.0000	-3.3617	0.0009
Celf4	0.0000	-1.7524	0.0029	0.0000	-2.0685	0.0020
Celf5	0.0000	-1.2628	0.0104	0.0000	-1.4517	0.0087
Celf6	0.0000	-1.5035	0.0239	0.0000	-1.8693	0.0188
Celsr2	0.0000	-1.2471	0.0099	0.0000	-2.2265	0.0024
Celsr3	0.0000	-1.5318	0.0057	0.0000	-2.9532	0.0008
Cend1	0.0000	-2.1486	0.0111	0.0000	-2.5531	0.0087
Cenpn	0.0000	1.7666	0.0085	0.0000	1.4821	0.0141
Cenpw	0.0000	2.7491	0.0034	0.0000	1.9108	0.0105

Cep112	0.0000	3.0120	0.0045	0.0000	1.8002	0.0175
Cep170	0.0000	-1.1654	0.0065	0.0000	-1.7543	0.0024
Cerk	0.0000	-1.8173	0.0057	0.0000	-1.3745	0.0119
Cfap161	0.0000	4.5953	0.0425	0.0000	4.3172	0.0460
Cfap52	0.0000	3.4973	0.0156	0.0000	2.6845	0.0257
Cfap70	0.0000	6.9072	0.0123	0.0000	6.1864	0.0173
Cfl2	0.0000	-1.0106	0.0090	0.0000	-1.0124	0.0100
Cgn	0.0000	1.9434	0.0089	0.0000	1.8764	0.0109
Chac1	0.0000	2.3942	0.0083	0.0000	2.4459	0.0090
Chaf1b	0.0000	1.3688	0.0172	0.0000	1.2721	0.0210
Chd3	0.0000	-1.0851	0.0158	0.0000	-1.4122	0.0105
Chek1	0.0000	1.5296	0.0089	0.0000	1.2598	0.0153
Chek2	0.0000	1.6146	0.0150	0.0000	1.3909	0.0211
Chga	0.0000	-1.2123	0.0140	0.0000	-1.2097	0.0154
Chl1	0.0000	-2.3251	0.0053	0.0000	-2.5794	0.0047
Chn1	0.0000	-1.1172	0.0102	0.0000	-1.5843	0.0050
Chrna3	0.0000	-1.1539	0.0363	0.0000	-3.7641	0.0076
Chrna4	0.0000	-1.7225	0.0049	0.0000	-2.5446	0.0018
Chrna5	0.0000	-2.8386	0.0114	0.0000	-3.1443	0.0101
Chrnb2	0.0000	-1.7206	0.0046	0.0000	-2.8408	0.0011
Chrnb4	0.0000	-2.0486	0.0244	0.0000	-1.9700	0.0265
Chst11	0.0000	-2.0676	0.0056	0.0000	-1.3975	0.0142
Chsy3	0.0000	-1.7142	0.0281	0.0000	-1.6908	0.0292
Chtf18	0.0000	1.5307	0.0111	0.0000	1.7487	0.0093
Cilp2	0.0000	1.5396	0.0303	0.0000	1.6848	0.0282
Cirbp	0.0000	-1.2967	0.0092	0.0000	-1.2728	0.0107
Ckb	0.0000	-1.1381	0.0070	0.0000	-1.1049	0.0089
Cks2	0.0000	1.6719	0.0059	0.0000	1.2202	0.0142
Cldn19	0.0000	4.4673	0.0304	0.0000	3.5801	0.0398
Cldn2	0.0000	4.0881	0.0222	0.0000	2.3781	0.0432
Cldn4	0.0000	2.3874	0.0044	0.0000	4.4738	0.0004
Clec2l	0.0000	-1.8161	0.0387	0.0000	-1.4601	0.0459
Clip2	0.0000	-1.5492	0.0038	0.0000	-2.1232	0.0016
Clip3	0.0000	-1.3401	0.0063	0.0000	-1.6831	0.0041
Clmp	0.0000	-1.7185	0.0080	0.0000	-1.4052	0.0137
Cln5	0.0000	1.1712	0.0109	0.0000	1.7847	0.0042
Clstn2	0.0000	-2.4728	0.0132	0.0000	-2.0466	0.0189
Clu	0.0000	2.8184	0.0017	0.0000	2.5704	0.0025
Cmtm6	0.0000	1.1072	0.0119	0.0000	1.7912	0.0041
Cmtm7	0.0000	1.7791	0.0118	0.0000	1.5021	0.0183
Cmtm8	0.0000	-2.5286	0.0092	0.0000	-1.5011	0.0220
Cnksr2	0.0000	-1.5011	0.0249	0.0000	-1.8242	0.0202
Cnr1	0.0000	-1.7135	0.0080	0.0000	-2.8234	0.0025
Cntd1	0.0000	2.3011	0.0247	0.0000	3.7057	0.0114
Cntfr	0.0000	-1.2388	0.0080	0.0000	-1.2757	0.0086
Cntn4	0.0000	-2.1504	0.0425	0.0000	-3.7659	0.0288
Cobl	0.0000	2.6311	0.0010	0.0000	3.1760	0.0003
Col11a1	0.0000	-2.4090	0.0033	0.0000	-2.0333	0.0062
Col23a1	0.0000	-2.6482	0.0085	0.0000	-1.7639	0.0190
Col5a2	0.0000	2.8355	0.0017	0.0000	3.7647	0.0005
Col5a3	0.0000	4.0710	0.0156	0.0000	4.5969	0.0137
Col6a5	0.0000	6.8866	0.0037	0.0000	6.9188	0.0044
Col9a1	0.0000	-5.4337	0.0044	0.0000	-1.8487	0.0221
Col9a3	0.0001	1.1475	0.0467	0.0000	1.6625	0.0344
Coq3	0.0000	1.0579	0.0242	0.0000	1.4740	0.0153
Coro6	0.0000	2.7983	0.0409	0.0000	3.4418	0.0345
Cox19	0.0000	-1.1591	0.0125	0.0000	-1.7322	0.0058
Cox7a1	0.0000	2.4368	0.0271	0.0000	2.5614	0.0265
Cp	0.0000	1.3341	0.0141	0.0000	1.7976	0.0083
Cpa2	0.0000	2.3073	0.0375	0.0001	1.8065	0.0477
Cpeb1	0.0000	1.4234	0.0180	0.0000	1.7181	0.0139
Cpeb3	0.0000	-1.3195	0.0300	0.0000	-1.2260	0.0334
Cpeb4	0.0000	-1.4015	0.0080	0.0000	-1.8899	0.0043
Cplx1	0.0000	-2.1840	0.0112	0.0000	-1.6917	0.0187
Cplx2	0.0000	-2.1810	0.0019	0.0000	-1.9446	0.0029
Cpm	0.0000	-2.1171	0.0159	0.0000	-1.0975	0.0362
Cpn1	0.0000	3.6356	0.0339	0.0000	5.0332	0.0239
Cpne9	0.0000	3.3353	0.0340	0.0000	3.7791	0.0309
Cpq	0.0000	3.8276	0.0067	0.0000	3.4876	0.0099
Cpt1a	0.0000	2.0622	0.0033	0.0000	2.9296	0.0010
Crabp1	0.0000	-1.6672	0.0068	0.0000	-2.1134	0.0043
Crabp2	0.0000	-1.3601	0.0387	0.0000	-2.2552	0.0233
Crb1	0.0000	-1.4771	0.0158	0.0000	-2.5025	0.0062
Creb5	0.0000	-1.0044	0.0412	0.0000	-1.1547	0.0374
Crim1	0.0000	-2.0220	0.0037	0.0000	-1.3971	0.0104
Crlf3	0.0000	1.8184	0.0053	0.0000	1.8372	0.0061
Crmp1	0.0000	-1.9489	0.0011	0.0000	-3.2096	0.0002

Csdc2	0.0000	-1.9254	0.0059	0.0000	-1.5952	0.0100
Csf1r	0.0002	2.4649	0.0478	0.0000	2.8626	0.0435
Cspg5	0.0000	-1.7120	0.0066	0.0000	-1.1173	0.0175
Csrnp1	0.0000	1.8423	0.0163	0.0000	1.9517	0.0161
Csrnp3	0.0000	-1.1127	0.0126	0.0000	-2.4505	0.0019
Cth	0.0000	3.7691	0.0010	0.0000	4.5167	0.0004
Ctif	0.0000	-1.3842	0.0083	0.0000	-1.7531	0.0053
Ctnnbip1	0.0000	-1.2825	0.0117	0.0000	-1.3095	0.0126
Ctsc	0.0000	3.9589	0.0024	0.0000	3.5320	0.0040
Ctsf	0.0000	-1.5285	0.0133	0.0000	-1.4314	0.0161
Cttnbp2	0.0000	-1.4044	0.0226	0.0000	-3.0694	0.0065
Cuedc1	0.0000	-1.6399	0.0052	0.0000	-1.0793	0.0147
Cul4b	0.0000	2.4817	0.0007	0.0000	2.2268	0.0008
Cux2	0.0000	-1.6170	0.0096	0.0000	-2.4139	0.0042
Cx3cl1	0.0000	3.8763	0.0009	0.0000	1.4569	0.0159
Cxcl12	0.0000	-5.6669	0.0005	0.0000	-1.4991	0.0108
Cxxc4	0.0000	-1.9514	0.0027	0.0000	-2.5967	0.0012
Cyb561d1	0.0000	-1.0480	0.0366	0.0000	-1.2062	0.0330
Cyb5r1	0.0000	2.1636	0.0021	0.0000	2.7046	0.0009
Cyba	0.0000	2.0827	0.0135	0.0000	3.7430	0.0033
Cyfip2	0.0000	-1.4080	0.0040	0.0000	-1.7857	0.0023
Cyp27a1	0.0000	3.6828	0.0293	0.0000	4.5482	0.0234
Cyth1	0.0000	-1.4054	0.0123	0.0000	-1.9104	0.0073
Daam1	0.0000	-1.2229	0.0065	0.0000	-1.3064	0.0065
Dab2	0.0000	-2.5913	0.0051	0.0000	-1.0896	0.0274
Dach2	0.0000	-1.8481	0.0206	0.0000	-3.1071	0.0097
Dact2	0.0000	3.1414	0.0040	0.0000	4.1496	0.0018
Dapp1	0.0000	3.1351	0.0242	0.0000	3.4216	0.0229
Dbf4	0.0000	1.2265	0.0091	0.0000	1.2745	0.0094
Dbndd1	0.0000	-1.4601	0.0189	0.0000	-2.3064	0.0091
Dbndd2	0.0000	-1.2814	0.0326	0.0000	-2.2186	0.0175
Dbx2	0.0000	-3.2917	0.0280	0.0000	-2.8351	0.0315
Dchs1	0.0000	-1.3191	0.0054	0.0000	-1.5215	0.0042
Dchs2	0.0000	-4.8852	0.0035	0.0000	-3.0792	0.0062
Dclk1	0.0000	-1.5262	0.0054	0.0000	-2.0819	0.0026
Dctd	0.0000	1.1229	0.0239	0.0000	2.7425	0.0037
Dcx	0.0000	-2.3637	0.0010	0.0000	-3.9595	0.0002
Ddn	0.0000	6.3330	0.0020	0.0000	2.2369	0.0266
Ddx58	0.0000	2.6130	0.0042	0.0000	1.5578	0.0166
Dennd2c	0.0000	2.5582	0.0024	0.0000	3.2552	0.0011
Dennd2d	0.0001	2.9249	0.0465	0.0001	2.9923	0.0467
Dennd5b	0.0000	-1.3527	0.0094	0.0000	-1.9625	0.0042
Depdc7	0.0000	1.8957	0.0205	0.0000	1.8981	0.0217
Deptor	0.0000	2.2020	0.0100	0.0000	3.9135	0.0022
Dgka	0.0000	1.8832	0.0121	0.0000	2.1973	0.0097
Dgkb	0.0000	-2.9559	0.0134	0.0000	-2.3501	0.0189
Dgke	0.0000	-1.2321	0.0188	0.0000	-1.0183	0.0257
Dhdh	0.0000	1.7711	0.0179	0.0000	3.2359	0.0049
Diaph1	0.0000	1.3774	0.0043	0.0000	1.4146	0.0047
Diras1	0.0000	-1.9664	0.0105	0.0000	-2.5357	0.0069
Disp2	0.0000	-1.3304	0.0125	0.0000	-1.4053	0.0125
Dixdc1	0.0000	-1.4418	0.0103	0.0000	-1.0594	0.0198
Dkc1	0.0000	1.7457	0.0024	0.0000	1.8997	0.0021
Dlc1	0.0000	1.7521	0.0033	0.0000	2.4676	0.0011
Dlg2	0.0000	-2.0477	0.0222	0.0000	-2.3902	0.0190
Dlg3	0.0000	-1.3100	0.0089	0.0000	-1.1544	0.0128
Dlgap5	0.0000	1.3544	0.0076	0.0000	1.7171	0.0047
Dll1	0.0000	-1.0533	0.0183	0.0000	-1.9811	0.0054
Dll4	0.0000	-3.1227	0.0201	0.0000	-2.3559	0.0270
Dmbx1	0.0000	4.6799	0.0043	0.0000	2.2920	0.0236
Dmc1	0.0000	2.7904	0.0263	0.0000	3.6622	0.0185
Dmd	0.0000	1.0840	0.0165	0.0000	1.9123	0.0050
Dmgdh	0.0000	5.3332	0.0153	0.0000	2.3285	0.0461
Dmrta2	0.0000	1.2248	0.0304	0.0000	2.6734	0.0082
Dnah6	0.0000	4.8767	0.0187	0.0000	3.1937	0.0349
Dnah8	0.0000	4.7606	0.0009	0.0000	3.4094	0.0032
Dnah9	0.0000	-1.3992	0.0297	0.0000	-1.7955	0.0230
Dnajb5	0.0000	-1.3710	0.0088	0.0000	-1.1720	0.0136
Dnajc12	0.0000	-1.4051	0.0212	0.0000	-1.0893	0.0298
Dnajc21	0.0000	1.3343	0.0078	0.0000	1.5136	0.0066
Dnajc22	0.0000	1.6453	0.0452	0.0000	2.4009	0.0325
Dnajc28	0.0000	-1.5828	0.0303	0.0000	-1.7617	0.0276
Dner	0.0000	-1.3105	0.0091	0.0000	-1.9436	0.0039
Dnm1	0.0000	-2.1102	0.0047	0.0000	-1.5848	0.0104
Dnm3	0.0000	-2.3057	0.0085	0.0000	-2.9319	0.0055
Dnmt3b	0.0000	2.2820	0.0032	0.0000	1.1720	0.0182
Dnmt3l	0.0000	4.1683	0.0010	0.0000	3.5679	0.0018

Doc2b	0.0000	-1.2098	0.0347	0.0000	-2.3444	0.0165
Dock10	0.0000	2.4252	0.0129	0.0000	1.0245	0.0435
Dock2	0.0000	3.5981	0.0386	0.0001	2.9001	0.0477
Dock4	0.0000	-1.3754	0.0103	0.0000	-1.9894	0.0050
Dock6	0.0000	1.1693	0.0093	0.0000	1.5202	0.0055
Dock9	0.0000	1.7530	0.0081	0.0000	1.2150	0.0191
Dok5	0.0000	-2.1640	0.0108	0.0000	-2.0523	0.0129
Dok6	0.0000	-1.3768	0.0322	0.0000	-2.7916	0.0137
Dpf1	0.0000	-1.5008	0.0097	0.0000	-1.8358	0.0070
Dpf3	0.0000	-1.5622	0.0225	0.0000	-2.6048	0.0108
Dph6	0.0000	1.0478	0.0216	0.0000	1.1318	0.0205
Dpp10	0.0000	-1.6057	0.0257	0.0000	-1.4086	0.0304
Dpp4	0.0000	1.2785	0.0375	0.0000	1.2827	0.0385
Dppa2	0.0000	3.5155	0.0036	0.0000	3.0829	0.0062
Dppa4	0.0000	4.8702	0.0011	0.0000	4.8634	0.0011
Dpys	0.0000	5.2196	0.0156	0.0000	5.3588	0.0165
Dpysl2	0.0000	-1.2684	0.0083	0.0000	-1.9513	0.0030
Dpysl3	0.0000	-1.3933	0.0026	0.0000	-2.4334	0.0003
Dpysl4	0.0000	-3.4644	0.0007	0.0000	-3.5663	0.0004
Dpysl5	0.0000	-1.5974	0.0022	0.0000	-3.0449	0.0002
Draxin	0.0000	-1.6279	0.0085	0.0000	-2.5397	0.0032
Drc1	0.0000	2.5969	0.0225	0.0000	3.3312	0.0162
Dsg2	0.0000	3.2979	0.0019	0.0000	3.6110	0.0015
Dsp	0.0000	1.1198	0.0195	0.0000	2.4512	0.0035
Dtl	0.0000	1.2655	0.0087	0.0000	1.1876	0.0113
Dtwd2	0.0000	1.1489	0.0372	0.0000	1.3108	0.0338
Dtx3	0.0000	-1.2676	0.0067	0.0000	-1.0997	0.0107
Dtx3l	0.0000	2.5611	0.0135	0.0000	1.6684	0.0288
Dtymk	0.0000	1.1273	0.0080	0.0000	1.4180	0.0051
Dusp10	0.0000	-1.6039	0.0198	0.0000	-1.2500	0.0280
Dusp27	0.0000	2.4006	0.0047	0.0000	2.8490	0.0034
Dusp4	0.0000	-1.5762	0.0047	0.0000	-1.4942	0.0064
Dusp5	0.0000	1.6130	0.0257	0.0000	1.3571	0.0330
Dusp8	0.0000	-1.7464	0.0028	0.0000	-2.1057	0.0019
Dyrk2	0.0000	-1.0326	0.0118	0.0000	-1.0655	0.0125
Dyrk3	0.0000	1.7502	0.0066	0.0000	1.5142	0.0108
E2f2	0.0000	-1.1491	0.0295	0.0000	-1.5539	0.0211
Ebf1	0.0000	-1.0792	0.0129	0.0000	-2.0242	0.0032
Ebf3	0.0000	-1.1643	0.0090	0.0000	-3.2069	0.0004
Ebf4	0.0000	-1.6962	0.0224	0.0000	-1.1693	0.0347
Ebp	0.0000	1.7255	0.0073	0.0000	1.6143	0.0098
Echdc2	0.0000	2.1981	0.0089	0.0000	1.4490	0.0220
Echdc3	0.0000	3.0457	0.0396	0.0001	2.5608	0.0471
Ecm2	0.0000	4.2318	0.0072	0.0000	1.9842	0.0314
Ect2	0.0000	1.4631	0.0071	0.0000	1.2583	0.0117
Ednrb	0.0000	-1.3486	0.0080	0.0000	-1.4018	0.0085
Eed	0.0000	1.4188	0.0049	0.0000	1.1609	0.0096
Eef1a2	0.0000	-1.7946	0.0150	0.0000	-1.1157	0.0303
Eef2k	0.0000	-1.4044	0.0097	0.0000	-1.0657	0.0177
Efcab10	0.0000	4.3771	0.0367	0.0000	3.5609	0.0457
Efcab12	0.0000	3.0058	0.0330	0.0000	2.1629	0.0463
Efcc1	0.0000	-1.2868	0.0378	0.0000	-2.5100	0.0192
Efhc1	0.0000	1.9313	0.0230	0.0000	2.5234	0.0160
Efhc2	0.0000	4.1510	0.0104	0.0000	3.2145	0.0194
Efr3b	0.0000	-1.3549	0.0087	0.0000	-1.7240	0.0056
Egfl7	0.0000	1.0667	0.0293	0.0000	2.0903	0.0099
Ehd3	0.0000	-1.0246	0.0296	0.0000	-1.2124	0.0253
Ehd4	0.0000	1.0322	0.0198	0.0000	1.3203	0.0139
Eif1b	0.0000	-1.0180	0.0118	0.0000	-1.0121	0.0134
Eif2d	0.0000	1.5360	0.0054	0.0000	1.1887	0.0116
Eif2s2	0.0000	1.6527	0.0014	0.0000	1.3112	0.0034
Eif4ebp1	0.0000	2.7938	0.0007	0.0000	3.6064	0.0001
Elavl2	0.0000	-1.5332	0.0049	0.0000	-2.1649	0.0021
Elavl3	0.0000	-1.9396	0.0019	0.0000	-2.6410	0.0006
Elavl4	0.0000	-1.9116	0.0018	0.0000	-2.7766	0.0004
Elf4	0.0000	2.0214	0.0136	0.0000	1.6276	0.0215
Elfn1	0.0000	-1.1649	0.0365	0.0000	-1.6893	0.0258
Elfn2	0.0000	-1.9601	0.0137	0.0000	-1.5499	0.0206
Ell2	0.0000	1.4199	0.0121	0.0000	2.3123	0.0040
Ell3	0.0000	2.9959	0.0207	0.0000	3.2264	0.0198
Elmod1	0.0000	-3.7585	0.0064	0.0000	-3.2938	0.0086
Elovl4	0.0000	-1.4411	0.0114	0.0000	-1.1142	0.0194
Elovl7	0.0000	3.1299	0.0094	0.0000	4.4652	0.0041
Emb	0.0000	2.5696	0.0009	0.0000	1.8977	0.0028
Eml1	0.0000	-1.0483	0.0152	0.0000	-1.8677	0.0047
Endov	0.0000	-1.2282	0.0123	0.0000	-1.7953	0.0061
Eng	0.0000	3.6725	0.0039	0.0000	4.6401	0.0021

Enho	0.0000	-1.6104	0.0088	0.0000	-2.0396	0.0057
Enkd1	0.0000	1.0849	0.0283	0.0000	1.0085	0.0322
Enkur	0.0000	2.1917	0.0347	0.0001	1.6052	0.0472
Eomes	0.0000	3.6046	0.0101	0.0000	3.5890	0.0116
Epha10	0.0000	-1.5307	0.0194	0.0000	-1.7115	0.0173
Epha2	0.0000	2.0575	0.0027	0.0000	2.2224	0.0025
Epha3	0.0000	-1.4350	0.0090	0.0000	-2.9159	0.0016
Epha5	0.0000	-2.9362	0.0025	0.0000	-4.3128	0.0009
Epha8	0.0000	-3.4288	0.0075	0.0000	-3.5267	0.0075
Ephb1	0.0000	-2.4005	0.0024	0.0000	-2.9565	0.0013
Ephb2	0.0000	-1.6076	0.0032	0.0000	-2.2004	0.0012
Ephx2	0.0000	4.6874	0.0006	0.0000	4.5421	0.0005
Epm2aip1	0.0000	-1.4164	0.0074	0.0000	-1.2383	0.0114
Erbb3	0.0000	2.3807	0.0123	0.0000	2.0456	0.0184
Erbb4	0.0000	-1.6071	0.0117	0.0000	-2.9965	0.0031
Erc2	0.0000	-1.2114	0.0150	0.0000	-1.9766	0.0060
Errfi1	0.0000	-1.1858	0.0140	0.0000	-1.2180	0.0148
Espl1	0.0000	1.4504	0.0067	0.0000	1.3263	0.0097
Esrrb	0.0000	3.4713	0.0005	0.0000	4.4183	0.0001
Esrrg	0.0000	-2.4006	0.0065	0.0000	-4.2594	0.0019
Esyt3	0.0000	2.4762	0.0276	0.0000	3.1416	0.0208
Etv4	0.0000	3.5939	0.0025	0.0000	2.9342	0.0055
Etv5	0.0000	3.3722	0.0004	0.0000	3.4113	0.0001
Evc2	0.0000	1.2821	0.0209	0.0000	2.2425	0.0073
Evl	0.0000	-1.0229	0.0097	0.0000	-1.4629	0.0046
Exosc5	0.0000	1.5152	0.0088	0.0000	1.5597	0.0094
Eya2	0.0000	-1.3384	0.0307	0.0000	-1.1597	0.0363
Ezr	0.0000	2.0862	0.0012	0.0000	1.0043	0.0111
F2rl1	0.0000	2.2958	0.0181	0.0000	4.5261	0.0039
Fabp3	0.0000	1.7239	0.0067	0.0000	3.8461	0.0003
Fads6	0.0000	2.0825	0.0298	0.0000	4.9714	0.0060
Fah	0.0000	1.9840	0.0165	0.0000	2.9021	0.0080
Faim2	0.0000	-2.1383	0.0268	0.0000	-2.1744	0.0267
Fam102b	0.0000	-1.1509	0.0156	0.0000	-1.2377	0.0149
Fam111a	0.0000	1.4338	0.0117	0.0000	1.0014	0.0241
Fam117a	0.0000	1.2320	0.0213	0.0000	1.7352	0.0124
Fam124a	0.0000	-1.4622	0.0242	0.0000	-1.4857	0.0249
Fam129a	0.0000	1.1379	0.0359	0.0000	2.7088	0.0099
Fam131a	0.0000	-1.3540	0.0202	0.0000	-1.3097	0.0221
Fam131b	0.0000	-1.3363	0.0162	0.0000	-2.4417	0.0052
Fam135b	0.0000	-1.7712	0.0369	0.0000	-3.9117	0.0177
Fam149a	0.0000	1.2852	0.0303	0.0000	1.2865	0.0315
Fam155a	0.0000	-1.6967	0.0142	0.0000	-1.9154	0.0125
Fam160a1	0.0000	1.7337	0.0383	0.0001	1.3328	0.0488
Fam161a	0.0000	1.3131	0.0311	0.0000	1.9453	0.0192
Fam169b	0.0000	3.8634	0.0176	0.0000	3.6516	0.0209
Fam171a1	0.0000	-1.2959	0.0075	0.0000	-1.0118	0.0142
Fam171a2	0.0000	-1.2764	0.0081	0.0000	-1.6058	0.0052
Fam171b	0.0000	-1.3965	0.0052	0.0000	-1.9286	0.0024
Fam178b	0.0000	5.0438	0.0074	0.0000	6.1881	0.0050
Fam189a1	0.0000	-1.4897	0.0193	0.0000	-1.8612	0.0143
Fam20c	0.0000	-1.7616	0.0072	0.0000	-1.5418	0.0106
Fam219a	0.0000	-1.5117	0.0068	0.0000	-1.7991	0.0051
Fam227a	0.0001	-1.1196	0.0455	0.0000	-1.5431	0.0357
Fam57b	0.0000	-2.6133	0.0036	0.0000	-2.9198	0.0030
Fam72a	0.0000	1.3613	0.0343	0.0000	1.4514	0.0333
Fam78a	0.0000	1.7844	0.0310	0.0000	2.6263	0.0192
Fam78b	0.0000	-1.2563	0.0199	0.0000	-2.8321	0.0044
Fam81a	0.0000	2.4134	0.0055	0.0000	2.5354	0.0056
Fam83d	0.0000	1.2704	0.0159	0.0000	1.3328	0.0161
Fam83g	0.0000	1.8016	0.0297	0.0000	2.0807	0.0260
Fam89b	0.0000	-1.6794	0.0154	0.0000	-1.9199	0.0133
Fanca	0.0000	2.1526	0.0055	0.0000	1.6858	0.0115
Fancd2	0.0000	1.9273	0.0060	0.0000	1.6042	0.0110
Fanci	0.0000	2.1192	0.0052	0.0000	1.8722	0.0084
Fancl	0.0000	1.6350	0.0098	0.0000	1.2521	0.0185
Farsb	0.0000	1.3332	0.0062	0.0000	1.2892	0.0080
Fastkd2	0.0000	1.2813	0.0113	0.0000	1.1304	0.0163
Fat3	0.0000	-1.4340	0.0067	0.0000	-2.4300	0.0017
Fat4	0.0000	-2.7577	0.0014	0.0000	-3.1237	0.0009
Faxc	0.0000	-1.6148	0.0046	0.0000	-2.6992	0.0010
Fbll1	0.0000	-1.6500	0.0246	0.0000	-2.0119	0.0200
Fbln7	0.0000	2.1724	0.0362	0.0000	2.0683	0.0393
Fbxl16	0.0000	-1.2364	0.0126	0.0000	-1.5492	0.0089
Fbxl7	0.0000	-2.1626	0.0110	0.0000	-1.1096	0.0309
Fbxo21	0.0000	-1.1312	0.0066	0.0000	-1.1316	0.0077
Fbxo27	0.0000	1.8389	0.0311	0.0000	3.2549	0.0137

Fbxo32	0.0000	-1.6526	0.0167	0.0000	-1.8805	0.0143
Fbxo41	0.0000	-1.2442	0.0285	0.0000	-1.9425	0.0166
Fbxo44	0.0000	-1.6918	0.0246	0.0000	-1.0490	0.0407
Fbxo5	0.0000	1.4604	0.0083	0.0000	1.5403	0.0084
Fen1	0.0000	1.2405	0.0114	0.0000	1.0501	0.0176
Fes	0.0001	2.2144	0.0461	0.0001	2.1761	0.0478
Fez1	0.0000	-1.8896	0.0018	0.0000	-1.9495	0.0017
Fgd3	0.0000	-1.7553	0.0237	0.0000	-1.4814	0.0298
Fgf1	0.0000	1.2557	0.0384	0.0000	3.3854	0.0084
Fgf12	0.0000	-2.3679	0.0134	0.0000	-3.2551	0.0084
Fgf13	0.0000	-1.9268	0.0076	0.0000	-1.8987	0.0088
Fgf17	0.0000	3.9374	0.0189	0.0000	5.0891	0.0129
Fgf9	0.0001	-1.5989	0.0463	0.0000	-2.1327	0.0378
Fgfr3	0.0000	-3.1390	0.0013	0.0000	-2.7961	0.0019
Fhod1	0.0000	1.1591	0.0206	0.0000	1.2097	0.0206
Fibcd1	0.0000	-2.0300	0.0166	0.0000	-2.4187	0.0133
Fign	0.0000	-1.3375	0.0139	0.0000	-1.8967	0.0076
Fignl1	0.0000	1.1225	0.0176	0.0000	1.4688	0.0117
Filip1	0.0000	-1.1245	0.0309	0.0000	-2.6901	0.0092
Flrt1	0.0000	-2.1926	0.0181	0.0000	-1.9259	0.0225
Flrt2	0.0000	-3.3435	0.0024	0.0000	-1.9161	0.0100
Flt4	0.0000	2.2108	0.0271	0.0000	2.2751	0.0272
Flywch1	0.0000	-1.2291	0.0077	0.0000	-1.5225	0.0052
Fmn1	0.0000	2.5248	0.0212	0.0000	2.2454	0.0266
Fmn2	0.0000	-1.4860	0.0092	0.0000	-2.0265	0.0050
Fndc5	0.0000	-4.2946	0.0055	0.0000	-2.4008	0.0153
Folr1	0.0000	4.0827	0.0020	0.0000	3.4541	0.0040
Foxc2	0.0000	-5.1903	0.0065	0.0000	-1.1742	0.0379
Foxh1	0.0000	3.7413	0.0041	0.0000	4.9125	0.0020
Foxj1	0.0000	1.3368	0.0143	0.0000	1.5973	0.0110
Foxl2	0.0000	-1.9718	0.0285	0.0000	-1.9127	0.0299
Fpgs	0.0000	1.5462	0.0101	0.0000	1.3725	0.0145
Frat1	0.0000	-1.8555	0.0216	0.0000	-1.8875	0.0219
Frk	0.0000	4.2649	0.0105	0.0000	3.1612	0.0208
Frmd4b	0.0000	-2.1904	0.0016	0.0000	-2.3456	0.0012
Frmpd3	0.0000	-2.1478	0.0117	0.0000	-2.9018	0.0073
Frrs1	0.0000	3.9720	0.0011	0.0000	4.7283	0.0004
Frrs1l	0.0000	-2.4272	0.0115	0.0000	-2.0334	0.0162
Frzb	0.0000	-2.8505	0.0053	0.0000	-2.5122	0.0074
Fstl5	0.0000	-3.0701	0.0072	0.0000	-4.5232	0.0040
Ftsj3	0.0000	1.4118	0.0060	0.0000	1.1601	0.0112
Fyn	0.0000	-1.2325	0.0069	0.0000	-1.5067	0.0048
Fzd5	0.0000	2.6479	0.0025	0.0000	3.8080	0.0007
G0s2	0.0000	1.4913	0.0261	0.0000	3.1948	0.0062
Gab2	0.0000	-1.9701	0.0040	0.0000	-1.9748	0.0043
Gabbr1	0.0000	-1.4848	0.0047	0.0000	-1.9904	0.0024
Gabbr2	0.0000	-1.3362	0.0354	0.0000	-1.4144	0.0344
Gabrb2	0.0000	-1.6276	0.0213	0.0000	-3.4117	0.0069
Gabrb3	0.0000	-1.1849	0.0179	0.0000	-1.5818	0.0115
Gad1	0.0000	-1.5644	0.0253	0.0000	-1.3964	0.0297
Gadd45g	0.0000	-1.1744	0.0155	0.0000	-2.5245	0.0030
Galm	0.0000	2.0741	0.0125	0.0000	2.6019	0.0085
Galnt12	0.0000	4.0990	0.0036	0.0000	1.3724	0.0343
Galnt16	0.0000	-1.1526	0.0127	0.0000	-1.0519	0.0165
Galnt18	0.0000	-2.7953	0.0099	0.0000	-2.4749	0.0129
Galnt3	0.0000	2.9301	0.0232	0.0000	1.7847	0.0425
Galnt6	0.0000	1.9300	0.0260	0.0000	3.4842	0.0094
Galt	0.0000	1.4189	0.0238	0.0000	1.6946	0.0196
Gap43	0.0000	-1.7344	0.0019	0.0000	-2.3979	0.0005
Gart	0.0000	1.6362	0.0018	0.0000	1.4878	0.0028
Gata2	0.0000	-2.1882	0.0152	0.0000	-1.9030	0.0200
Gata3	0.0000	-3.4002	0.0051	0.0000	-2.1383	0.0125
Gatm	0.0000	1.9394	0.0318	0.0000	2.0778	0.0307
Gba2	0.0000	-1.0100	0.0175	0.0000	-1.3028	0.0121
Gbp3	0.0000	2.8822	0.0370	0.0000	3.3190	0.0332
Gca	0.0000	1.2922	0.0278	0.0000	1.4622	0.0249
Gcat	0.0000	1.7102	0.0056	0.0000	1.6844	0.0070
Gclm	0.0000	1.0444	0.0175	0.0000	1.6807	0.0072
Gcnt1	0.0000	1.0460	0.0408	0.0000	1.4789	0.0296
Gdap1	0.0000	-1.4010	0.0108	0.0000	-2.2516	0.0040
Gdap1l1	0.0000	-2.6826	0.0026	0.0000	-2.7936	0.0025
Gdi1	0.0000	-1.3175	0.0041	0.0000	-1.3128	0.0048
Gdpd5	0.0000	-1.5739	0.0083	0.0000	-2.1610	0.0043
Gem	0.0000	1.1660	0.0444	0.0000	1.6112	0.0336
Gfod2	0.0000	-1.0359	0.0165	0.0000	-1.8884	0.0051
Gfpt2	0.0000	1.6870	0.0120	0.0000	2.7802	0.0038
Gfra1	0.0000	-1.8488	0.0029	0.0000	-2.0432	0.0025

Gipr	0.0000	-1.8779	0.0270	0.0000	-1.1167	0.0441
Gjb5	0.0000	3.0813	0.0086	0.0000	4.2314	0.0041
Gjd2	0.0000	-1.2782	0.0369	0.0000	-2.0113	0.0239
Gk	0.0000	1.4423	0.0157	0.0000	1.5024	0.0160
Glis3	0.0000	3.7940	0.0014	0.0000	1.0317	0.0306
Glra1	0.0000	-3.5295	0.0213	0.0000	-4.7368	0.0176
Glra2	0.0000	-2.6443	0.0229	0.0000	-3.3027	0.0182
Gls2	0.0000	2.6973	0.0098	0.0000	2.9675	0.0089
Glt1d1	0.0000	1.9779	0.0113	0.0000	2.0092	0.0123
Gmnn	0.0000	1.5758	0.0094	0.0000	1.2610	0.0165
Gna15	0.0000	5.0791	0.0133	0.0000	5.6818	0.0119
Gnal	0.0000	-1.7713	0.0092	0.0000	-1.9741	0.0080
Gnaq	0.0000	-1.2663	0.0061	0.0000	-1.5094	0.0046
Gnaz	0.0000	-1.1046	0.0250	0.0000	-1.2812	0.0215
Gnb5	0.0000	-1.2082	0.0159	0.0000	-1.5178	0.0113
Gng2	0.0000	-1.5061	0.0028	0.0000	-1.7710	0.0020
Gng7	0.0000	-1.9528	0.0165	0.0000	-1.5964	0.0228
Gnl3	0.0000	1.7125	0.0023	0.0000	1.7890	0.0023
Gnpda1	0.0000	1.7240	0.0042	0.0000	2.5934	0.0011
Gnpnat1	0.0000	1.5180	0.0086	0.0000	1.7014	0.0074
Golga7b	0.0000	-2.9056	0.0088	0.0000	-2.6994	0.0106
Gpat2	0.0000	4.0671	0.0028	0.0000	4.1714	0.0030
Gpc2	0.0000	-1.7199	0.0027	0.0000	-2.7006	0.0006
Gpr137c	0.0000	-1.5043	0.0181	0.0000	-1.4279	0.0205
Gpr153	0.0000	-2.4515	0.0044	0.0000	-1.1965	0.0199
Gpr155	0.0000	-1.2254	0.0408	0.0000	-2.0232	0.0260
Gpr160	0.0000	4.2224	0.0228	0.0000	4.0345	0.0260
Gpr161	0.0000	-1.0427	0.0154	0.0000	-1.7690	0.0055
Gpr162	0.0000	-1.4494	0.0152	0.0000	-1.8822	0.0101
Gpr173	0.0000	-1.4380	0.0132	0.0000	-2.0578	0.0070
Gpr22	0.0000	-1.3731	0.0291	0.0000	-3.2985	0.0087
Gpr83	0.0000	3.1924	0.0366	0.0002	2.3280	0.0494
Gprin1	0.0000	-1.5404	0.0080	0.0000	-2.9390	0.0014
Gpsm1	0.0000	-1.6300	0.0038	0.0000	-1.8401	0.0031
Gpsm3	0.0001	1.7489	0.0459	0.0000	2.4568	0.0345
Gpx4	0.0000	2.4214	0.0013	0.0000	1.7226	0.0042
Gpx7	0.0000	1.8319	0.0186	0.0000	3.2923	0.0055
Gramd1a	0.0000	-1.6253	0.0062	0.0000	-1.0322	0.0180
Gramd1c	0.0000	2.5058	0.0181	0.0000	1.9555	0.0277
Grb10	0.0000	-3.1926	0.0006	0.0000	-2.9398	0.0005
Grb7	0.0000	3.2393	0.0083	0.0000	3.5788	0.0075
Greb1	0.0000	-1.0284	0.0412	0.0000	-1.6766	0.0263
Grhl1	0.0002	1.4940	0.0487	0.0000	1.9127	0.0408
Grhl3	0.0000	3.2283	0.0057	0.0000	3.2752	0.0066
Gria2	0.0000	-2.2239	0.0070	0.0000	-2.3442	0.0071
Gria4	0.0000	-1.4979	0.0172	0.0000	-1.2534	0.0235
Grik2	0.0000	-1.7629	0.0228	0.0000	-1.9967	0.0202
Grik3	0.0000	-1.5463	0.0039	0.0000	-1.6963	0.0035
Grik4	0.0000	-2.5092	0.0150	0.0000	-1.6316	0.0273
Grik5	0.0000	-2.1812	0.0027	0.0000	-1.4795	0.0082
Grin2b	0.0000	-2.6959	0.0091	0.0000	-2.5220	0.0111
Grin3a	0.0000	-2.4048	0.0182	0.0000	-3.0036	0.0139
Grip2	0.0000	-2.0157	0.0310	0.0000	-1.6838	0.0370
Grk5	0.0000	-1.2561	0.0195	0.0000	-1.4534	0.0165
Grm3	0.0000	-2.2316	0.0393	0.0001	-1.7326	0.0472
Grtp1	0.0000	2.0467	0.0091	0.0000	2.3104	0.0078
Gsdmd	0.0000	3.2068	0.0278	0.0000	4.4282	0.0184
Gsg1l	0.0000	-1.0195	0.0325	0.0000	-3.2740	0.0053
Gsk3b	0.0000	-1.1916	0.0051	0.0000	-1.8631	0.0015
Gss	0.0000	1.1518	0.0181	0.0000	1.7580	0.0084
Gsta4	0.0000	1.6565	0.0031	0.0000	2.1675	0.0014
Gstm1	0.0000	1.8903	0.0050	0.0000	2.2364	0.0035
Gstm2	0.0000	2.3178	0.0092	0.0000	2.3301	0.0102
Gstm3	0.0000	4.9940	0.0146	0.0000	4.3130	0.0207
Gstm4	0.0000	1.5252	0.0248	0.0000	2.2630	0.0138
Gstp1	0.0000	1.7061	0.0034	0.0000	2.1692	0.0018
Gstt1	0.0000	2.7971	0.0298	0.0000	3.0021	0.0286
Gstt2	0.0000	2.2251	0.0202	0.0000	3.7771	0.0074
Gsx1	0.0000	-1.3921	0.0434	0.0000	-2.0029	0.0326
Gtf2ird2	0.0000	-1.0025	0.0355	0.0000	-1.3621	0.0266
Hacd2	0.0000	1.1302	0.0193	0.0000	1.4107	0.0141
Hapln1	0.0000	-3.4111	0.0137	0.0000	-2.0576	0.0252
Haspin	0.0000	1.3589	0.0196	0.0000	1.4346	0.0193
Hat1	0.0000	1.7587	0.0019	0.0000	1.7863	0.0021
Haus4	0.0000	1.2707	0.0213	0.0000	1.7382	0.0132
Hck	0.0000	1.5342	0.0240	0.0000	3.4406	0.0046
Hcn3	0.0000	-1.6616	0.0197	0.0000	-2.0667	0.0149

Hcn4	0.0000	-1.0056	0.0233	0.0000	-1.8793	0.0085
Hdac9	0.0000	-1.0380	0.0279	0.0000	-1.1470	0.0257
Hddc3	0.0000	-1.5777	0.0132	0.0000	-1.2318	0.0208
Hdgfl3	0.0000	-1.2518	0.0058	0.0000	-1.6877	0.0030
Heg1	0.0000	-1.2716	0.0124	0.0000	-1.5186	0.0097
Helb	0.0000	1.4919	0.0125	0.0000	1.7716	0.0097
Hells	0.0000	2.0563	0.0019	0.0000	1.6930	0.0040
Helz2	0.0000	2.7737	0.0176	0.0000	2.3691	0.0242
Hepacam	0.0003	1.2425	0.0495	0.0000	4.1276	0.0098
Herpud1	0.0000	1.2403	0.0102	0.0000	1.1941	0.0125
Hexb	0.0000	1.8142	0.0030	0.0000	2.4700	0.0011
Hey2	0.0000	-2.8514	0.0188	0.0000	-2.4352	0.0229
Heyl	0.0000	-5.1153	0.0141	0.0001	-1.0865	0.0482
Hhat	0.0000	1.5614	0.0400	0.0000	2.1681	0.0294
Hhex	0.0000	2.1173	0.0349	0.0000	3.8008	0.0162
Hip1	0.0000	-1.0042	0.0098	0.0000	-1.4526	0.0045
Hivep2	0.0000	-1.4758	0.0116	0.0000	-1.1978	0.0182
Hivep3	0.0000	-2.1756	0.0359	0.0000	-1.8001	0.0421
Hmces	0.0000	1.9937	0.0051	0.0000	1.3649	0.0144
Hmcn2	0.0000	2.1270	0.0367	0.0000	3.1253	0.0243
Hmga1	0.0000	3.1521	0.0005	0.0000	1.2750	0.0087
Hmgb2	0.0000	2.7085	0.0009	0.0000	1.8964	0.0034
Hmgcs1	0.0000	-1.5595	0.0013	0.0000	-1.4901	0.0017
Hmox1	0.0000	2.4926	0.0024	0.0000	3.0047	0.0014
Hook1	0.0000	1.5852	0.0043	0.0000	1.3545	0.0079
Hook2	0.0000	1.6131	0.0124	0.0000	1.4438	0.0172
Hopx	0.0000	-2.0191	0.0240	0.0000	-1.2419	0.0410
Hormad1	0.0000	4.7809	0.0105	0.0000	4.3367	0.0148
Hpca	0.0000	-4.0406	0.0052	0.0000	-2.4703	0.0120
Hpcal4	0.0000	-1.6567	0.0342	0.0000	-1.3235	0.0420
Hpgd	0.0001	1.3489	0.0455	0.0000	1.3556	0.0462
Hs3st3a1	0.0000	-2.7099	0.0259	0.0000	-2.3048	0.0305
Hs3st3b1	0.0000	-1.4935	0.0191	0.0000	-1.0930	0.0295
Hs3st5	0.0000	-1.7850	0.0328	0.0000	-3.6262	0.0159
Hsd17b11	0.0000	1.3245	0.0151	0.0000	2.2633	0.0048
Hsd17b14	0.0000	4.7238	0.0081	0.0000	5.2922	0.0071
Hsp90aa1	0.0000	2.0702	0.0005	0.0000	2.0758	0.0002
Hspa12a	0.0000	-1.7825	0.0031	0.0000	-2.2980	0.0016
Hspa14	0.0000	1.3380	0.0052	0.0000	1.0907	0.0101
Hspa1b	0.0000	1.7185	0.0153	0.0000	2.4715	0.0077
Hspa9	0.0000	1.1535	0.0047	0.0000	1.4134	0.0031
Hspb1	0.0000	3.5402	0.0006	0.0000	3.0354	0.0008
Hspb8	0.0000	2.3146	0.0027	0.0000	2.9162	0.0013
Hspbap1	0.0000	2.0696	0.0056	0.0000	1.9906	0.0074
Hspd1	0.0000	1.7449	0.0011	0.0000	1.2798	0.0033
Hspe1	0.0000	1.9849	0.0009	0.0000	1.8458	0.0012
Hydin	0.0000	4.0451	0.0114	0.0000	3.7089	0.0154
Iars	0.0000	1.4823	0.0023	0.0000	1.1847	0.0055
Id4	0.0000	-3.0051	0.0008	0.0000	-1.8625	0.0034
Idnk	0.0000	-1.3016	0.0263	0.0000	-1.0585	0.0338
Ids	0.0000	-1.2273	0.0113	0.0000	-1.6381	0.0067
Ifi30	0.0000	1.5832	0.0097	0.0000	3.4193	0.0009
Ifi35	0.0000	2.3871	0.0246	0.0000	2.1080	0.0305
Ifi44	0.0000	3.2231	0.0269	0.0000	2.9148	0.0317
Ifih1	0.0000	3.3083	0.0210	0.0000	1.7513	0.0450
Ifit1	0.0000	3.4914	0.0284	0.0000	3.8093	0.0267
Ifit3	0.0000	1.5864	0.0429	0.0000	2.4894	0.0278
Ifitm1	0.0000	3.1206	0.0014	0.0000	3.8895	0.0005
Ifitm3	0.0000	2.3701	0.0046	0.0000	4.2755	0.0006
Ifnlr1	0.0000	1.8786	0.0405	0.0000	1.9461	0.0403
Igf1r	0.0000	-1.5050	0.0032	0.0000	-1.4249	0.0043
Igf2r	0.0000	-1.0116	0.0115	0.0000	-1.3551	0.0069
Igfbp5	0.0000	-2.2596	0.0101	0.0000	-1.0049	0.0367
Iglon5	0.0000	-2.0391	0.0041	0.0000	-2.6108	0.0024
Igsf11	0.0000	-4.2223	0.0031	0.0000	-2.3409	0.0097
Igsf21	0.0000	-1.1203	0.0269	0.0000	-1.5092	0.0188
Igsf23	0.0000	3.3545	0.0294	0.0000	3.0163	0.0346
Il17rc	0.0000	2.4142	0.0286	0.0000	2.8374	0.0243
Il1r1	0.0000	2.7232	0.0248	0.0000	3.4174	0.0187
Il1rapl2	0.0000	-4.6420	0.0383	0.0000	-4.5203	0.0371
Il34	0.0001	2.0177	0.0471	0.0000	2.4502	0.0411
Impa2	0.0000	2.8578	0.0016	0.0000	3.1960	0.0010
Impact	0.0000	1.0699	0.0070	0.0000	1.8647	0.0015
Ina	0.0000	-1.7110	0.0028	0.0000	-3.1583	0.0002
Inafm2	0.0000	-1.8761	0.0069	0.0000	-1.2283	0.0173
Ing5	0.0000	1.5277	0.0067	0.0000	1.9178	0.0042
Inpp5d	0.0000	3.2050	0.0037	0.0000	3.8552	0.0024

Inpp5j	0.0000	-3.6836	0.0339	0.0000	-2.1473	0.0464
Insm2	0.0000	-3.8680	0.0422	0.0000	-4.9664	0.0387
Iqcg	0.0000	1.1854	0.0216	0.0000	1.0032	0.0285
Iqgap1	0.0000	1.6273	0.0022	0.0000	1.8850	0.0014
Iqsec3	0.0000	-2.9077	0.0035	0.0000	-2.7497	0.0042
Iqub	0.0000	4.5103	0.0196	0.0000	3.6152	0.0286
Irak2	0.0000	1.1692	0.0336	0.0000	1.2044	0.0339
Irak3	0.0000	2.6731	0.0141	0.0000	3.2823	0.0100
Irf1	0.0000	2.0421	0.0094	0.0000	1.8957	0.0124
Irf6	0.0000	3.1031	0.0107	0.0000	3.7104	0.0080
Irf7	0.0000	3.5858	0.0306	0.0000	3.0552	0.0376
Irgq	0.0000	-1.1004	0.0122	0.0000	-1.5700	0.0064
Isg15	0.0000	3.9420	0.0169	0.0000	4.2267	0.0164
Ism2	0.0000	2.8168	0.0187	0.0000	3.9198	0.0106
Itgae	0.0000	3.4399	0.0431	0.0000	3.6236	0.0426
Itgal	0.0000	3.6321	0.0368	0.0000	3.0308	0.0448
Itgam	0.0000	3.4563	0.0264	0.0000	3.3833	0.0283
Itgb7	0.0000	4.2686	0.0184	0.0000	4.2505	0.0201
Itgb8	0.0000	-1.8264	0.0057	0.0000	-1.9475	0.0058
Itpk1	0.0000	1.0560	0.0158	0.0000	1.1625	0.0145
Itpka	0.0000	2.4811	0.0191	0.0000	3.1510	0.0134
Itsn1	0.0000	-1.2196	0.0050	0.0000	-1.4752	0.0035
Itsn2	0.0000	1.1557	0.0141	0.0000	1.0415	0.0186
Jade1	0.0000	2.1210	0.0014	0.0000	2.5682	0.0006
Jade2	0.0000	2.5418	0.0093	0.0000	3.2110	0.0058
Jade3	0.0000	1.8854	0.0026	0.0000	1.7487	0.0039
Jag1	0.0000	-1.1580	0.0138	0.0000	-1.0799	0.0172
Jak3	0.0000	2.1872	0.0057	0.0000	2.4463	0.0049
Jakmip1	0.0000	2.1242	0.0094	0.0000	2.4042	0.0079
Jazf1	0.0000	-1.2155	0.0349	0.0000	-1.6106	0.0268
Jph1	0.0000	1.7110	0.0199	0.0000	2.4217	0.0111
Jph3	0.0000	-1.9121	0.0074	0.0000	-1.1972	0.0196
Jph4	0.0000	-1.6340	0.0148	0.0000	-1.0817	0.0283
Jun	0.0000	-1.7062	0.0029	0.0000	-1.3860	0.0061
Kalrn	0.0000	-1.0219	0.0127	0.0000	-2.1135	0.0024
Kank3	0.0000	2.3308	0.0046	0.0000	2.7468	0.0033
Kat2b	0.0000	1.2030	0.0108	0.0000	1.3696	0.0093
Katnal1	0.0000	-1.1615	0.0127	0.0000	-1.2003	0.0132
Kcnab2	0.0000	-2.0820	0.0111	0.0000	-1.6906	0.0170
Kcnb1	0.0000	-1.4914	0.0163	0.0000	-1.6521	0.0147
Kcnb2	0.0000	-1.4423	0.0355	0.0000	-2.1243	0.0246
Kcnc1	0.0000	-2.7780	0.0038	0.0000	-2.6785	0.0045
Kcnd2	0.0000	-2.6406	0.0249	0.0000	-3.2094	0.0206
Kcnd3	0.0000	-1.0007	0.0233	0.0000	-2.0318	0.0071
Kcnh3	0.0000	2.4468	0.0295	0.0000	3.3507	0.0201
Kcnh7	0.0000	-1.7259	0.0310	0.0000	-1.9900	0.0274
Kcnip1	0.0000	-1.5970	0.0317	0.0000	-1.7396	0.0297
Kcnip2	0.0004	1.0030	0.0497	0.0000	1.8305	0.0292
Kcnip4	0.0000	-3.4687	0.0203	0.0000	-3.3945	0.0208
Kcnj11	0.0000	-2.5646	0.0272	0.0000	-2.3548	0.0296
Kcnj5	0.0000	-4.1323	0.0411	0.0000	-3.3409	0.0439
Kcnk1	0.0000	1.9780	0.0118	0.0000	1.9893	0.0132
Kcnk10	0.0000	-1.2442	0.0285	0.0000	-2.8238	0.0086
Kcnk3	0.0000	-2.6283	0.0221	0.0000	-1.9584	0.0306
Kcnma1	0.0000	-1.2228	0.0320	0.0000	-2.0617	0.0174
Kcnmb2	0.0000	-1.8246	0.0350	0.0000	-2.3941	0.0276
Kcnmb4	0.0000	-1.5989	0.0251	0.0000	-1.8628	0.0215
Kcnn1	0.0000	-1.5903	0.0109	0.0000	-1.1242	0.0212
Kcnn3	0.0000	-1.1075	0.0329	0.0000	-1.3261	0.0281
Kcnq2	0.0000	-1.1868	0.0123	0.0000	-2.3180	0.0027
Kcnq3	0.0000	-1.1014	0.0303	0.0000	-2.3516	0.0102
Kcns3	0.0000	1.7232	0.0330	0.0000	2.6996	0.0192
Kctd1	0.0000	-1.0607	0.0242	0.0000	-1.4324	0.0167
Kctd4	0.0000	1.6170	0.0439	0.0000	2.2799	0.0325
Kctd7	0.0000	-1.3906	0.0232	0.0000	-1.1566	0.0299
Kdf1	0.0000	2.3229	0.0363	0.0000	2.9227	0.0294
Kdm6a	0.0000	1.4524	0.0049	0.0000	1.0319	0.0130
Kidins220	0.0000	-1.0556	0.0080	0.0000	-1.8166	0.0020
Kif14	0.0000	1.5037	0.0090	0.0000	1.2790	0.0143
Kif18b	0.0000	1.1477	0.0196	0.0000	1.5103	0.0131
Kif1b	0.0000	-1.1715	0.0038	0.0000	-2.0489	0.0005
Kif20a	0.0000	1.3782	0.0063	0.0000	1.2881	0.0089
Kif22	0.0000	1.1687	0.0167	0.0000	1.1355	0.0193
Kif2c	0.0000	1.2593	0.0143	0.0000	1.2884	0.0154
Kif3c	0.0000	-1.2781	0.0048	0.0000	-1.5426	0.0033
Kif5a	0.0000	-1.5733	0.0043	0.0000	-2.1470	0.0020
Kif5c	0.0000	-2.0231	0.0014	0.0000	-2.8957	0.0002

Kifc1	0.0000	1.2082	0.0100	0.0000	1.2775	0.0100
Kirrel3	0.0000	-1.8555	0.0134	0.0000	-2.5154	0.0081
Klc1	0.0000	-1.3631	0.0041	0.0000	-1.8808	0.0017
Klc3	0.0000	2.2502	0.0303	0.0000	2.3857	0.0295
Klf15	0.0000	1.4517	0.0226	0.0000	2.9387	0.0057
Klf2	0.0000	4.4947	0.0004	0.0000	5.1046	0.0001
Klf4	0.0000	1.6448	0.0106	0.0000	2.9969	0.0023
Klf5	0.0000	1.8021	0.0247	0.0000	2.5402	0.0159
Klf8	0.0000	2.1434	0.0098	0.0000	1.4854	0.0217
Klf9	0.0000	2.3122	0.0021	0.0000	3.6339	0.0003
Klhl1	0.0000	-1.5644	0.0354	0.0000	-3.8603	0.0140
Klhl14	0.0000	-2.3469	0.0234	0.0000	-3.6326	0.0146
Klhl3	0.0000	-1.4327	0.0226	0.0000	-2.1776	0.0127
Klhl32	0.0000	-1.2146	0.0296	0.0000	-1.9674	0.0166
Klhl5	0.0000	-1.3701	0.0118	0.0000	-1.0609	0.0200
Kmt2e	0.0000	-1.1544	0.0082	0.0000	-1.7084	0.0034
Kndc1	0.0000	-1.2122	0.0267	0.0000	-1.0997	0.0306
Krt18	0.0003	1.0013	0.0496	0.0000	1.2248	0.0440
Ksr2	0.0000	-1.2413	0.0234	0.0000	-2.9395	0.0053
L1cam	0.0000	-1.4974	0.0052	0.0000	-2.8213	0.0008
L2hgdh	0.0000	1.0571	0.0186	0.0000	1.4757	0.0106
Lactb2	0.0000	2.3906	0.0013	0.0000	3.0476	0.0003
Lair1	0.0001	-2.9208	0.0460	0.0001	-2.5975	0.0486
Lama1	0.0000	-1.9106	0.0038	0.0000	-1.8338	0.0049
Lama3	0.0000	3.0102	0.0182	0.0000	3.1407	0.0184
Lama5	0.0000	1.9742	0.0040	0.0000	1.3697	0.0119
Lamc3	0.0000	-5.3556	0.0107	0.0000	-2.2834	0.0214
Lamp1	0.0000	1.1479	0.0054	0.0000	1.7241	0.0018
Lamp2	0.0000	1.3272	0.0048	0.0000	1.4637	0.0043
Lamp5	0.0000	-3.0733	0.0059	0.0000	-2.9121	0.0073
Lap3	0.0000	1.7949	0.0027	0.0000	1.9779	0.0024
Laptm5	0.0000	4.1865	0.0027	0.0000	5.4627	0.0012
Lbh	0.0000	-1.8506	0.0017	0.0000	-1.9141	0.0016
Lbr	0.0000	1.3498	0.0069	0.0000	1.0188	0.0151
Lbx1	0.0000	-3.3600	0.0197	0.0000	-3.6666	0.0186
Ldhd	0.0000	5.5973	0.0267	0.0000	5.7876	0.0268
Lefty1	0.0000	3.2741	0.0012	0.0000	4.3768	0.0002
Letmd1	0.0000	-1.2435	0.0189	0.0000	-1.3488	0.0177
Lgals3	0.0000	1.9167	0.0202	0.0000	3.3651	0.0067
Lgals3bp	0.0000	2.3849	0.0122	0.0000	2.9246	0.0088
Lgi1	0.0000	3.1889	0.0113	0.0000	2.4586	0.0205
Lgr4	0.0000	-1.0421	0.0108	0.0000	-1.1307	0.0102
Lgr5	0.0000	-2.3316	0.0241	0.0000	-1.3646	0.0407
Lhfpl3	0.0000	-1.9954	0.0410	0.0000	-2.3060	0.0371
Lhfpl4	0.0000	-1.6128	0.0068	0.0000	-2.1838	0.0035
Lhx9	0.0000	6.3124	0.0022	0.0001	1.2838	0.0484
Lig1	0.0000	1.2213	0.0068	0.0000	1.0000	0.0126
Lima1	0.0000	1.2769	0.0053	0.0000	2.4163	0.0006
Limch1	0.0000	-1.8739	0.0047	0.0000	-1.4853	0.0092
Limk1	0.0000	-1.0521	0.0135	0.0000	-1.3880	0.0085
Lin7a	0.0000	-3.6566	0.0036	0.0000	-4.5719	0.0026
Lipc	0.0000	5.1477	0.0348	0.0000	5.7420	0.0326
Liph	0.0000	2.6187	0.0165	0.0000	2.4249	0.0204
Llgl2	0.0000	2.4312	0.0036	0.0000	2.7678	0.0028
Lmo1	0.0000	-1.2070	0.0306	0.0000	-1.9276	0.0177
Lmo3	0.0000	-1.1432	0.0333	0.0000	-1.8569	0.0195
Lmo4	0.0000	-1.2315	0.0108	0.0000	-1.0048	0.0176
Lmo7	0.0000	1.9552	0.0101	0.0000	2.7669	0.0047
Lnx1	0.0000	2.7891	0.0268	0.0000	2.2148	0.0362
Lonrf3	0.0000	2.6301	0.0099	0.0000	1.6095	0.0262
Lpar5	0.0001	3.4981	0.0470	0.0000	3.9482	0.0437
Lpin3	0.0000	2.8294	0.0191	0.0000	2.9047	0.0198
Lrch2	0.0000	-1.1915	0.0102	0.0000	-1.5745	0.0062
Lrfn2	0.0000	-1.7105	0.0380	0.0000	-1.5100	0.0426
Lrfn3	0.0000	-1.8361	0.0147	0.0000	-1.9284	0.0145
Lrfn5	0.0000	-1.9577	0.0303	0.0000	-2.0229	0.0300
Lrmp	0.0000	4.0858	0.0162	0.0000	5.0107	0.0121
Lrp12	0.0000	-1.2817	0.0089	0.0000	-1.7044	0.0051
Lrp3	0.0000	-1.9146	0.0043	0.0000	-1.7578	0.0063
Lrp8	0.0000	-1.4893	0.0057	0.0000	-1.4497	0.0072
Lrrc10b	0.0000	1.5280	0.0371	0.0000	1.2810	0.0448
Lrrc2	0.0000	3.3923	0.0146	0.0000	5.6681	0.0047
Lrrc20	0.0000	-1.4172	0.0142	0.0000	-1.7015	0.0109
Lrrc34	0.0000	4.7065	0.0027	0.0000	5.0350	0.0025
Lrrc4	0.0000	-1.9014	0.0111	0.0000	-1.8419	0.0128
Lrrc49	0.0000	-1.1126	0.0162	0.0000	-1.8654	0.0061
Lrrc4b	0.0000	-1.7762	0.0033	0.0000	-1.8733	0.0033

Lrrc4c	0.0000	-2.5138	0.0186	0.0000	-3.3463	0.0132
Lrrc75b	0.0000	-2.0898	0.0121	0.0000	-1.8242	0.0164
Lrrn1	0.0000	-2.1819	0.0018	0.0000	-2.7227	0.0008
Lrrn3	0.0000	-1.9545	0.0047	0.0000	-2.1117	0.0045
Lrrn4	0.0000	2.0482	0.0349	0.0000	3.0065	0.0226
Lrrtm2	0.0000	-2.0846	0.0151	0.0000	-2.2568	0.0141
Lrrtm3	0.0000	-1.2617	0.0310	0.0000	-3.3650	0.0084
Lsamp	0.0000	-1.8690	0.0050	0.0000	-1.8455	0.0059
Lsm11	0.0000	-1.0423	0.0162	0.0000	-1.1328	0.0152
Lsr	0.0000	1.4667	0.0145	0.0000	1.3423	0.0186
Lss	0.0000	-1.1272	0.0075	0.0000	-1.0902	0.0093
Luzp2	0.0000	3.0923	0.0049	0.0000	1.5182	0.0246
Ly6h	0.0000	-1.1682	0.0113	0.0000	-2.1345	0.0029
Ly75	0.0000	3.9759	0.0006	0.0000	4.3514	0.0002
Lyn	0.0000	1.6629	0.0276	0.0000	2.0047	0.0225
Lynx1	0.0000	-3.2120	0.0250	0.0000	-1.4746	0.0462
Lypla1	0.0000	1.3613	0.0061	0.0000	1.7181	0.0038
Lysmd1	0.0000	-1.2380	0.0220	0.0000	-1.5892	0.0161
M1ap	0.0000	5.2519	0.0105	0.0000	4.6185	0.0156
Madcam1	0.0000	3.8189	0.0425	0.0000	5.4848	0.0308
Madd	0.0000	-1.1168	0.0120	0.0000	-2.1834	0.0026
Mael	0.0000	3.9988	0.0152	0.0000	4.4540	0.0138
Magee1	0.0000	-1.8729	0.0032	0.0000	-2.2584	0.0021
Magef1	0.0000	2.1119	0.0225	0.0000	2.2700	0.0215
Mak	0.0000	2.7517	0.0244	0.0000	1.6046	0.0453
Mall	0.0000	3.1985	0.0449	0.0000	4.8613	0.0308
Malt1	0.0000	1.0573	0.0423	0.0000	1.6521	0.0277
Mamdc2	0.0000	3.6676	0.0203	0.0000	2.9568	0.0292
Maml3	0.0000	-1.3397	0.0099	0.0000	-1.7449	0.0061
Man2a2	0.0000	-1.8510	0.0035	0.0000	-1.2512	0.0106
Man2b1	0.0000	1.1309	0.0140	0.0000	1.5790	0.0077
Manba	0.0000	3.3570	0.0010	0.0000	2.7809	0.0022
Maob	0.0000	4.1310	0.0243	0.0000	3.7909	0.0290
Map10	0.0000	2.7234	0.0050	0.0000	2.4264	0.0080
Map1b	0.0000	-1.0348	0.0045	0.0000	-2.3211	0.0002
Map3k10	0.0000	-1.5630	0.0063	0.0000	-1.2445	0.0120
Map3k13	0.0000	-1.2109	0.0199	0.0000	-2.3150	0.0063
Map3k15	0.0000	2.8177	0.0079	0.0000	2.6613	0.0103
Map3k19	0.0000	4.9598	0.0238	0.0000	4.6271	0.0278
Map3k5	0.0000	1.0570	0.0411	0.0000	1.6085	0.0272
Map7d3	0.0000	1.7136	0.0279	0.0000	2.7735	0.0137
Mapk11	0.0000	-1.2683	0.0202	0.0000	-1.9560	0.0102
Mapk13	0.0000	2.4988	0.0299	0.0000	2.6841	0.0286
Mapk8	0.0000	-1.4965	0.0043	0.0000	-1.4502	0.0055
Mapk8ip2	0.0000	-1.2543	0.0073	0.0000	-2.0747	0.0021
Mapkapk3	0.0000	1.1206	0.0195	0.0000	1.9151	0.0072
Mapkbp1	0.0000	-1.0230	0.0169	0.0000	-1.2048	0.0137
Mapt	0.0000	-2.2349	0.0021	0.0000	-2.2975	0.0022
Marcks	0.0000	-1.1102	0.0065	0.0000	-1.1444	0.0073
Mark1	0.0000	-1.6210	0.0057	0.0000	-1.4310	0.0088
Marveld2	0.0000	2.8550	0.0165	0.0000	1.7178	0.0353
Mast1	0.0000	-1.0531	0.0188	0.0000	-2.1769	0.0047
Mast4	0.0000	-1.1550	0.0165	0.0000	-1.5252	0.0107
Mastl	0.0000	1.1286	0.0232	0.0000	1.1891	0.0230
Mb21d2	0.0000	-1.3725	0.0100	0.0000	-1.6935	0.0071
Mboat1	0.0000	2.0918	0.0229	0.0000	2.4146	0.0198
Mboat2	0.0000	-1.0417	0.0155	0.0000	-1.1179	0.0150
Mcf2	0.0000	4.8194	0.0011	0.0000	4.6260	0.0013
Mcm4	0.0000	1.2780	0.0063	0.0000	1.0200	0.0125
Mcm5	0.0000	2.3917	0.0015	0.0000	2.0626	0.0027
Mcm6	0.0000	2.0689	0.0015	0.0000	1.6157	0.0037
Mcm7	0.0000	1.3750	0.0066	0.0000	1.1454	0.0120
Mcmdc2	0.0000	4.6228	0.0401	0.0000	5.2722	0.0367
Mcph1	0.0000	1.5844	0.0076	0.0000	1.7921	0.0064
Mdga2	0.0000	-2.2519	0.0206	0.0000	-2.4023	0.0197
Me1	0.0000	1.0439	0.0223	0.0000	1.7796	0.0090
Mecom	0.0000	-2.1386	0.0109	0.0000	-2.3031	0.0102
Meg3	0.0000	-2.4071	0.0019	0.0000	-1.0424	0.0164
Megf9	0.0000	-1.4357	0.0048	0.0000	-1.4279	0.0056
Meiob	0.0000	3.5556	0.0359	0.0000	4.6080	0.0279
Meis1	0.0000	-1.7731	0.0042	0.0000	-3.1924	0.0007
Meis2	0.0000	-1.8090	0.0023	0.0000	-2.7296	0.0005
Meis3	0.0000	-1.7330	0.0082	0.0000	-2.5013	0.0038
Metrnl	0.0000	-1.0251	0.0326	0.0000	-1.0709	0.0322
Mex3b	0.0000	-1.1089	0.0107	0.0000	-1.5296	0.0058
Mfng	0.0000	-1.2037	0.0276	0.0000	-1.1357	0.0304
Mfsd10	0.0000	1.1746	0.0207	0.0000	1.3316	0.0182

Mgarp	0.0000	1.2310	0.0319	0.0000	2.8212	0.0080
Mgat3	0.0000	-2.4983	0.0016	0.0000	-1.3264	0.0093
Mgat4c	0.0000	-2.4912	0.0211	0.0000	-3.8781	0.0130
Mgat5b	0.0000	-1.7213	0.0046	0.0000	-1.9673	0.0037
Mgll	0.0000	-1.2312	0.0433	0.0001	-1.0622	0.0487
Mia	0.0000	4.8286	0.0116	0.0000	4.9743	0.0125
Miat	0.0000	-1.6378	0.0441	0.0000	-2.1645	0.0359
Mib1	0.0000	-1.0130	0.0077	0.0000	-1.4722	0.0033
Mical2	0.0000	-3.0994	0.0077	0.0000	-1.1476	0.0340
Mif4gd	0.0000	1.8156	0.0093	0.0000	2.4095	0.0050
Mir124-2h	0.0000	-2.6871	0.0020	0.0000	-3.2694	0.0010
Mis18bp1	0.0000	1.4089	0.0087	0.0000	1.2294	0.0133
Mitf	0.0000	1.7145	0.0224	0.0000	1.7939	0.0222
Mks1	0.0000	1.0966	0.0310	0.0000	1.0370	0.0343
Mlh3	0.0000	1.8605	0.0053	0.0000	1.5465	0.0098
Mllt11	0.0000	-1.6773	0.0026	0.0000	-2.1575	0.0012
Mmab	0.0000	-1.1388	0.0184	0.0000	-1.0478	0.0218
Mme	0.0000	2.1257	0.0053	0.0000	3.0095	0.0021
Mmp24	0.0000	-2.1671	0.0045	0.0000	-2.3816	0.0040
Mmrn2	0.0000	1.7019	0.0242	0.0000	4.0303	0.0039
Mnd1	0.0000	4.7767	0.0038	0.0000	3.1176	0.0133
Moap1	0.0000	-1.1568	0.0299	0.0000	-1.6595	0.0199
Mobp	0.0000	2.6023	0.0271	0.0000	3.4723	0.0186
Mov10	0.0000	2.5488	0.0012	0.0000	3.0092	0.0005
Mphosph6	0.0000	1.4650	0.0079	0.0000	1.3072	0.0116
Mpp2	0.0000	-2.1993	0.0036	0.0000	-2.2868	0.0036
Mpp3	0.0000	-1.5559	0.0210	0.0000	-2.9585	0.0077
Mpped1	0.0000	-1.5207	0.0354	0.0000	-2.2580	0.0245
Mpped2	0.0000	-1.0692	0.0326	0.0000	-1.5792	0.0214
Mras	0.0000	-2.1130	0.0044	0.0000	-1.4556	0.0115
Mreg	0.0000	1.8362	0.0051	0.0000	3.7418	0.0003
Mrpl15	0.0000	1.5143	0.0040	0.0000	1.7983	0.0028
Mrpl35	0.0000	1.0990	0.0221	0.0000	1.0884	0.0239
Mrps31	0.0000	1.3007	0.0095	0.0000	1.3675	0.0096
Mrps9	0.0000	1.0739	0.0105	0.0000	1.0366	0.0129
Msh2	0.0000	1.3619	0.0039	0.0000	1.1466	0.0074
Msrb2	0.0000	1.4294	0.0265	0.0000	1.6313	0.0231
Msto1	0.0000	1.1140	0.0142	0.0000	1.0518	0.0174
Mta3	0.0000	1.4085	0.0058	0.0000	1.9193	0.0027
Mtfp1	0.0000	1.2247	0.0398	0.0000	1.7191	0.0287
Mthfd1	0.0000	1.9342	0.0023	0.0000	2.2220	0.0016
Mthfd1l	0.0000	1.8624	0.0030	0.0000	1.7197	0.0045
Mthfd2	0.0000	2.1969	0.0015	0.0000	2.5449	0.0008
Mturn	0.0000	-1.4757	0.0046	0.0000	-2.0316	0.0021
Mtus2	0.0000	-1.7174	0.0088	0.0000	-2.1878	0.0057
Mutyh	0.0000	1.6348	0.0235	0.0000	1.3417	0.0319
Mvb12b	0.0000	-1.2593	0.0072	0.0000	-1.5102	0.0053
Mvp	0.0000	1.3963	0.0187	0.0000	1.5829	0.0164
Mxd1	0.0000	-1.3914	0.0210	0.0000	-1.5915	0.0182
Mxra7	0.0000	-1.6389	0.0070	0.0000	-1.6324	0.0081
Myb	0.0000	2.6427	0.0082	0.0000	2.7698	0.0083
Mybbp1a	0.0000	1.2056	0.0056	0.0000	1.1839	0.0071
Mybl2	0.0000	3.4050	0.0005	0.0000	4.1161	0.0001
Mybpc2	0.0000	3.4834	0.0409	0.0000	4.8675	0.0301
Mycl	0.0000	-1.6470	0.0044	0.0000	-1.9640	0.0032
Myh13	0.0000	4.9162	0.0054	0.0000	3.6202	0.0131
Myh14	0.0000	1.6610	0.0222	0.0000	2.1549	0.0156
Myh7b	0.0000	2.2133	0.0174	0.0000	2.5607	0.0146
Myh9	0.0000	1.1359	0.0077	0.0000	1.0581	0.0104
Myl6b	0.0000	-1.0599	0.0431	0.0000	-1.6396	0.0299
Myo15b	0.0000	2.7831	0.0435	0.0000	3.1119	0.0406
Myo1f	0.0000	2.4097	0.0421	0.0000	4.4922	0.0210
Myo5a	0.0000	-1.2655	0.0046	0.0000	-1.7645	0.0020
Myof	0.0000	1.4718	0.0154	0.0000	1.7387	0.0124
Myrf	0.0000	2.0542	0.0082	0.0000	2.3449	0.0068
Myt1	0.0000	-2.1140	0.0029	0.0000	-3.7521	0.0004
Myt1l	0.0000	-1.7968	0.0127	0.0000	-2.5627	0.0068
N4bp3	0.0000	2.0228	0.0076	0.0000	2.1749	0.0073
Nabp1	0.0000	1.5345	0.0083	0.0000	2.1366	0.0038
Nacad	0.0000	-1.7249	0.0076	0.0000	-2.9409	0.0022
Naf1	0.0000	1.3793	0.0076	0.0000	1.2981	0.0101
Nanog	0.0000	4.0511	0.0003	0.0000	5.1275	0.0000
Nap1l2	0.0000	-1.4534	0.0139	0.0000	-1.9934	0.0081
Nap1l3	0.0000	-1.7078	0.0184	0.0000	-1.5175	0.0228
Napb	0.0000	-1.4628	0.0093	0.0000	-2.2752	0.0035
Nat10	0.0000	1.1595	0.0134	0.0000	1.1877	0.0142
Nat8l	0.0000	-1.1889	0.0154	0.0000	-1.4863	0.0110

Nav2	0.0000	-1.2048	0.0107	0.0000	-1.9482	0.0038
Nav3	0.0000	-1.1178	0.0258	0.0000	-2.4785	0.0070
Ncam1	0.0000	-1.5165	0.0034	0.0000	-1.5700	0.0036
Ncam2	0.0000	-2.1582	0.0155	0.0000	-2.1428	0.0167
Ncan	0.0000	-1.0053	0.0152	0.0000	-1.1062	0.0142
Ncf2	0.0000	3.0386	0.0252	0.0000	3.2837	0.0240
Nckap1l	0.0000	2.8973	0.0260	0.0000	3.1357	0.0246
Ncl	0.0000	1.5745	0.0029	0.0000	1.4554	0.0044
Ncs1	0.0000	-1.5900	0.0062	0.0000	-1.8739	0.0047
Ndnf	0.0000	-2.6964	0.0034	0.0000	-2.2573	0.0059
Ndrg1	0.0000	1.2507	0.0205	0.0000	1.1348	0.0252
Ndrg3	0.0000	-1.1330	0.0123	0.0000	-1.2273	0.0117
Ndrg4	0.0000	-1.3342	0.0071	0.0000	-1.4430	0.0068
Ndufa4l2	0.0000	3.6051	0.0055	0.0000	4.8802	0.0026
Ndufaf6	0.0000	1.3117	0.0272	0.0000	1.1705	0.0324
Ndufb4	0.0000	2.7998	0.0023	0.0000	1.1805	0.0212
Neat1	0.0000	2.9734	0.0068	0.0000	6.1365	0.0006
Necab1	0.0001	1.2868	0.0458	0.0000	1.9556	0.0316
Nefh	0.0000	1.3629	0.0215	0.0000	2.5486	0.0064
Nefl	0.0000	-1.7647	0.0027	0.0000	-3.1050	0.0003
Nefm	0.0000	-1.1241	0.0358	0.0000	-3.2777	0.0067
Negr1	0.0000	-1.7727	0.0062	0.0000	-1.6627	0.0083
Neil2	0.0000	2.3423	0.0240	0.0000	4.2094	0.0085
Nek10	0.0000	4.4084	0.0332	0.0000	3.6536	0.0418
Nek2	0.0000	1.4672	0.0066	0.0000	1.7390	0.0050
Nell2	0.0000	-2.5498	0.0008	0.0000	-3.6651	0.0001
Nes	0.0000	-1.0605	0.0117	0.0000	-2.2163	0.0019
Neurod6	0.0000	-4.9777	0.0178	0.0000	-2.0609	0.0375
Nexn	0.0000	-1.7356	0.0212	0.0000	-1.1977	0.0332
Nfatc2ip	0.0000	1.5190	0.0061	0.0000	1.3143	0.0102
Nfia	0.0000	-1.7803	0.0084	0.0000	-1.3894	0.0155
Nfkbia	0.0000	1.3367	0.0151	0.0000	2.0850	0.0062
Ngfr	0.0000	-1.9746	0.0032	0.0000	-1.8231	0.0045
Nhp2	0.0000	1.0267	0.0181	0.0000	1.5527	0.0089
Nhs	0.0000	-1.2570	0.0195	0.0000	-1.0115	0.0273
Nicn1	0.0000	-1.1524	0.0114	0.0000	-1.1573	0.0126
Nip7	0.0000	1.0475	0.0121	0.0000	1.1276	0.0119
Nipa1	0.0000	-1.3926	0.0151	0.0000	-1.5461	0.0135
Nkain1	0.0000	-1.0400	0.0196	0.0000	-2.0893	0.0051
Nkain3	0.0000	-1.1448	0.0410	0.0000	-2.9503	0.0152
Nkx2-2	0.0000	-5.1620	0.0083	0.0000	-1.9925	0.0220
Nkx6-1	0.0000	-6.1467	0.0008	0.0000	-2.9094	0.0017
Nlgn1	0.0000	-1.8764	0.0216	0.0000	-2.1238	0.0192
Nlgn2	0.0000	-1.5043	0.0045	0.0000	-1.4548	0.0058
Nlrp12	0.0001	2.0685	0.0473	0.0000	5.4554	0.0142
Nlrp14	0.0000	3.5251	0.0429	0.0000	5.0159	0.0315
Nlrx1	0.0003	2.9475	0.0488	0.0000	4.7314	0.0328
Nme7	0.0000	1.2637	0.0160	0.0000	1.0113	0.0246
Nmi	0.0000	3.5427	0.0288	0.0000	2.4981	0.0430
Nmrk1	0.0002	1.0086	0.0487	0.0000	1.3882	0.0380
Noct	0.0000	3.6954	0.0003	0.0000	2.3604	0.0009
Nodal	0.0000	3.4171	0.0023	0.0000	4.5913	0.0008
Nol4	0.0000	-1.2326	0.0344	0.0000	-2.2154	0.0179
Nol4l	0.0000	-1.3798	0.0082	0.0000	-1.8254	0.0046
Nop2	0.0000	1.4596	0.0050	0.0000	1.0198	0.0137
Nop58	0.0000	1.4622	0.0021	0.0000	1.2779	0.0037
Nos1	0.0000	1.3825	0.0251	0.0000	1.5509	0.0224
Notch1	0.0000	-1.0055	0.0168	0.0000	-1.4426	0.0091
Notum	0.0000	5.0669	0.0204	0.0000	3.4809	0.0347
Nova1	0.0000	-2.7093	0.0019	0.0000	-3.2851	0.0009
Nova2	0.0000	-1.7990	0.0034	0.0000	-2.6433	0.0010
Npas3	0.0000	-1.8782	0.0081	0.0000	-1.9283	0.0085
Npdc1	0.0000	-1.3608	0.0068	0.0000	-1.2290	0.0097
Npepl1	0.0000	2.5051	0.0116	0.0000	3.4122	0.0063
Npm3	0.0000	1.3617	0.0103	0.0000	1.3666	0.0117
Npr2	0.0000	-1.4205	0.0133	0.0000	-1.1348	0.0205
Nptx2	0.0000	1.1976	0.0245	0.0000	2.3604	0.0071
Nqo1	0.0000	2.8355	0.0047	0.0000	4.1689	0.0015
Nr0b1	0.0000	4.4266	0.0013	0.0000	5.9387	0.0003
Nr2c1	0.0000	-1.3434	0.0224	0.0000	-1.6637	0.0173
Nr2f1	0.0000	-2.0178	0.0018	0.0000	-2.3908	0.0010
Nr2f2	0.0000	-2.1503	0.0030	0.0000	-1.7538	0.0063
Nr3c2	0.0000	2.1105	0.0251	0.0000	1.4930	0.0385
Nr4a2	0.0000	-1.7978	0.0115	0.0000	-1.9848	0.0102
Nrbp2	0.0000	-1.7000	0.0103	0.0000	-1.0373	0.0252
Nrcam	0.0000	-1.4434	0.0036	0.0000	-1.6525	0.0028
Nrep	0.0000	-1.9536	0.0012	0.0000	-2.2660	0.0007

Nrg3	0.0000	-1.8925	0.0430	0.0000	-3.0706	0.0298
Nrgn	0.0000	-1.4955	0.0282	0.0000	-1.3259	0.0327
Nrxn1	0.0000	-1.1756	0.0160	0.0000	-1.8216	0.0074
Nrxn2	0.0000	-1.5567	0.0066	0.0000	-1.9126	0.0046
Nrxn3	0.0000	-1.9487	0.0058	0.0000	-2.7586	0.0027
Nsg1	0.0000	-1.7050	0.0030	0.0000	-2.0237	0.0020
Nsmf	0.0000	-1.3424	0.0109	0.0000	-1.3438	0.0121
Nt5dc1	0.0000	1.9265	0.0162	0.0000	1.7786	0.0201
Nt5m	0.0000	-1.0494	0.0309	0.0000	-1.3381	0.0242
Ntn1	0.0000	-3.4235	0.0003	0.0000	-1.4005	0.0030
Ntn4	0.0000	-2.5197	0.0062	0.0000	-2.1941	0.0093
Ntng1	0.0000	-1.4308	0.0274	0.0000	-3.4548	0.0076
Ntng2	0.0000	-1.9765	0.0193	0.0000	-2.1065	0.0184
Ntrk2	0.0000	-1.7956	0.0084	0.0000	-1.2806	0.0183
Ntrk3	0.0000	-1.6347	0.0065	0.0000	-3.0129	0.0013
Nts	0.0000	-2.3825	0.0317	0.0000	-3.9985	0.0209
Nudc	0.0000	1.3794	0.0098	0.0000	1.1226	0.0168
Nup133	0.0000	1.2169	0.0091	0.0000	1.0153	0.0152
Nup62cl	0.0000	1.5778	0.0237	0.0000	2.4914	0.0113
Nup93	0.0000	1.5713	0.0025	0.0000	1.3377	0.0049
Nwd2	0.0000	-2.7019	0.0126	0.0000	-4.9367	0.0064
Nxph1	0.0000	-6.9555	0.0131	0.0000	-5.0493	0.0090
Nyap1	0.0000	-1.5225	0.0059	0.0000	-1.7470	0.0048
Oas2	0.0000	3.5527	0.0364	0.0000	3.8533	0.0350
Oas3	0.0000	3.3798	0.0368	0.0000	4.8740	0.0251
Ocln	0.0000	3.6762	0.0084	0.0000	2.4929	0.0205
Ogdhl	0.0000	1.0259	0.0099	0.0000	1.6272	0.0033
Oip5	0.0000	1.1749	0.0286	0.0000	1.2687	0.0273
Olfm1	0.0000	-1.6256	0.0053	0.0000	-2.5082	0.0017
Olfm2	0.0000	-1.8210	0.0051	0.0000	-2.3275	0.0030
Olfm3	0.0000	-1.4928	0.0392	0.0000	-2.1588	0.0284
Olig2	0.0000	-1.7820	0.0337	0.0000	-3.1519	0.0189
Oma1	0.0000	1.1448	0.0283	0.0000	1.0441	0.0328
Ophn1	0.0000	-1.0113	0.0259	0.0000	-1.2268	0.0209
Oprl1	0.0000	-1.1958	0.0255	0.0000	-2.3641	0.0091
Optn	0.0000	1.3407	0.0139	0.0000	1.4628	0.0130
Orc1	0.0000	2.7174	0.0029	0.0000	2.5028	0.0044
Orc2	0.0000	1.5659	0.0037	0.0000	1.6028	0.0040
Osbpl6	0.0000	-1.6388	0.0122	0.0000	-1.5901	0.0143
Osmr	0.0003	1.9359	0.0496	0.0000	4.5830	0.0198
Osr1	0.0000	-2.4887	0.0145	0.0000	-1.8181	0.0239
Ostf1	0.0000	1.3522	0.0130	0.0000	2.1849	0.0045
Otud1	0.0000	1.7789	0.0180	0.0000	2.2983	0.0120
Otud7a	0.0000	-1.1451	0.0375	0.0000	-1.2408	0.0358
Otx2	0.0000	4.4568	0.0007	0.0000	4.3640	0.0006
P2rx7	0.0000	1.6606	0.0229	0.0000	2.8200	0.0091
Pafah1b2	0.0000	-1.0165	0.0078	0.0000	-1.5350	0.0029
Pak3	0.0000	-2.4097	0.0020	0.0000	-2.0744	0.0035
Pak7	0.0000	-1.9053	0.0078	0.0000	-2.6251	0.0041
Palb2	0.0000	1.4744	0.0198	0.0000	1.5122	0.0203
Palm3	0.0000	1.7315	0.0127	0.0000	2.7858	0.0044
Pank3	0.0000	-1.0398	0.0103	0.0000	-1.2388	0.0080
Paqr4	0.0000	-1.1635	0.0216	0.0000	-1.6934	0.0125
Paqr9	0.0000	-2.0964	0.0336	0.0000	-1.8156	0.0384
Pard6g	0.0000	-1.0886	0.0143	0.0000	-1.0962	0.0154
Parm1	0.0000	-2.6321	0.0024	0.0000	-1.2064	0.0159
Parp10	0.0000	1.7333	0.0299	0.0000	1.4860	0.0366
Parp12	0.0000	2.3311	0.0093	0.0000	1.6981	0.0194
Parp14	0.0000	3.3007	0.0244	0.0000	2.7995	0.0319
Parp3	0.0000	1.6169	0.0325	0.0000	2.2340	0.0227
Parp4	0.0000	2.0805	0.0159	0.0000	1.8607	0.0208
Parp6	0.0000	-1.2640	0.0126	0.0000	-2.4784	0.0028
Parp9	0.0000	2.2648	0.0150	0.0000	1.5962	0.0277
Pask	0.0000	1.3576	0.0112	0.0000	1.3203	0.0134
Patl2	0.0000	3.8150	0.0266	0.0000	6.8376	0.0100
Pawr	0.0000	1.1634	0.0165	0.0000	1.7644	0.0075
Pax2	0.0000	-3.7348	0.0025	0.0000	-4.2004	0.0020
Pax6	0.0000	-1.4562	0.0119	0.0000	-1.9695	0.0071
Pax8	0.0000	-3.3540	0.0076	0.0000	-3.4475	0.0076
Pbx3	0.0000	-1.4497	0.0065	0.0000	-2.1646	0.0025
Pcdh10	0.0000	-2.3509	0.0062	0.0000	-3.0189	0.0039
Pcdh17	0.0000	-3.1651	0.0034	0.0000	-2.6928	0.0052
Pcdh19	0.0000	-1.7906	0.0171	0.0000	-1.5426	0.0222
Pcdh9	0.0000	-1.9585	0.0090	0.0000	-3.6167	0.0024
Pcdha3	0.0000	-1.6440	0.0138	0.0000	-2.3102	0.0078
Pcdhb10	0.0000	-1.2761	0.0243	0.0000	-2.1242	0.0119
Pcdhb3	0.0000	-1.5977	0.0173	0.0000	-1.3176	0.0237

Pcdhb5	0.0000	-5.8539	0.0049	0.0000	-2.3653	0.0116
Pcdhb7	0.0000	-1.5481	0.0235	0.0000	-2.2655	0.0144
Pcdhb8	0.0000	-2.3463	0.0196	0.0000	-2.0292	0.0239
Pcdhga4	0.0000	-1.0316	0.0327	0.0000	-1.3396	0.0254
Pcdhgb6	0.0000	-2.3443	0.0077	0.0000	-1.6432	0.0159
Pcdhgb7	0.0000	-1.4562	0.0269	0.0000	-1.1502	0.0350
Pced1b	0.0000	2.2179	0.0095	0.0000	3.1238	0.0043
Pclo	0.0000	-1.5566	0.0107	0.0000	-3.1133	0.0023
Pcolce2	0.0000	3.3219	0.0103	0.0000	3.8431	0.0083
Pcp4	0.0000	-5.3758	0.0027	0.0000	-3.2507	0.0047
Pcp4l1	0.0000	3.4020	0.0016	0.0000	2.7164	0.0037
Pcsk1	0.0000	5.4942	0.0087	0.0000	5.8650	0.0086
Pcsk6	0.0000	1.7263	0.0057	0.0000	2.6616	0.0017
Pdcl2	0.0000	5.3138	0.0212	0.0000	6.4071	0.0170
Pde10a	0.0000	-1.8408	0.0079	0.0000	-2.3295	0.0051
Pde1b	0.0000	-1.1985	0.0136	0.0000	-1.4309	0.0105
Pde8a	0.0000	3.7584	0.0062	0.0000	5.0838	0.0030
Pdgfd	0.0001	1.8583	0.0463	0.0000	3.1641	0.0280
Pdk4	0.0000	3.5951	0.0219	0.0000	4.9581	0.0135
Pdlim1	0.0000	1.7778	0.0100	0.0000	2.0304	0.0084
Pdzrn3	0.0000	-1.5416	0.0074	0.0000	-1.4523	0.0096
Pdzrn4	0.0000	-2.5048	0.0052	0.0000	-2.2004	0.0076
Pecam1	0.0000	3.2486	0.0019	0.0000	4.6843	0.0003
Pecr	0.0000	1.7686	0.0110	0.0000	1.8723	0.0110
Peli2	0.0000	-1.7860	0.0070	0.0000	-1.7090	0.0088
Peli3	0.0000	-1.4626	0.0271	0.0000	-1.5044	0.0267
Pfkfb1	0.0000	1.1318	0.0446	0.0000	1.6183	0.0327
Pfkfb3	0.0000	-1.4796	0.0057	0.0000	-1.5603	0.0058
Pfkfb4	0.0000	-1.0014	0.0387	0.0000	-2.2901	0.0153
Pfkp	0.0000	2.9829	0.0009	0.0000	2.8514	0.0010
Pgam2	0.0000	1.6276	0.0439	0.0000	1.9974	0.0374
Pgm2l1	0.0000	-1.9077	0.0026	0.0000	-3.1445	0.0004
Phactr1	0.0000	-1.3028	0.0232	0.0000	-2.1053	0.0117
Phf21b	0.0000	-1.9669	0.0039	0.0000	-2.9182	0.0012
Phf24	0.0000	-2.4715	0.0071	0.0000	-2.7296	0.0064
Phyh	0.0000	1.3352	0.0133	0.0000	1.5009	0.0116
Phyhd1	0.0000	2.9942	0.0239	0.0000	2.1968	0.0366
Phyhipl	0.0000	-2.0368	0.0037	0.0000	-2.2161	0.0033
Piezo1	0.0000	1.0281	0.0138	0.0000	1.5381	0.0063
Piezo2	0.0000	-3.3334	0.0221	0.0000	-2.1411	0.0382
Pim2	0.0000	2.1988	0.0049	0.0000	2.5297	0.0038
Pink1	0.0000	-1.6585	0.0042	0.0000	-1.3211	0.0083
Pip4k2b	0.0000	-1.0558	0.0095	0.0000	-1.0401	0.0111
Pir	0.0000	2.2693	0.0152	0.0000	2.2938	0.0164
Pirt	0.0000	4.6631	0.0228	0.0000	5.7270	0.0179
Piwil2	0.0000	4.5080	0.0068	0.0000	4.7998	0.0069
Pkdcc	0.0000	-1.9556	0.0034	0.0000	-1.1965	0.0122
Pkia	0.0000	-2.0219	0.0026	0.0000	-2.5047	0.0014
Pkn3	0.0000	1.4294	0.0271	0.0000	1.7560	0.0212
Pknox2	0.0000	-1.0168	0.0203	0.0000	-1.0018	0.0219
Pkp2	0.0000	2.1408	0.0047	0.0000	1.4052	0.0149
Pla1a	0.0000	4.6307	0.0143	0.0000	5.1102	0.0132
Pla2g10	0.0000	3.5032	0.0271	0.0000	4.0301	0.0236
Pla2g5	0.0000	3.9842	0.0224	0.0000	4.9846	0.0168
Pla2g7	0.0000	2.7382	0.0069	0.0000	1.2995	0.0299
Pla2r1	0.0000	3.9369	0.0442	0.0000	4.4640	0.0408
Plbd1	0.0000	2.8943	0.0297	0.0000	2.3156	0.0390
Plcb1	0.0000	-1.8372	0.0107	0.0000	-2.2958	0.0076
Plcd3	0.0000	1.2857	0.0270	0.0000	1.7769	0.0176
Plcd4	0.0000	2.6663	0.0149	0.0000	2.7425	0.0157
Plcg2	0.0000	3.4688	0.0035	0.0000	3.3000	0.0048
Plcl1	0.0000	-2.0905	0.0101	0.0000	-1.7696	0.0146
Pld6	0.0000	2.5063	0.0274	0.0000	3.8112	0.0150
Plek2	0.0000	4.3241	0.0385	0.0000	4.8255	0.0360
Plekhf2	0.0000	1.0888	0.0116	0.0000	1.9660	0.0027
Plekhg3	0.0000	1.9357	0.0074	0.0000	2.3521	0.0050
Plekhh1	0.0000	1.2059	0.0148	0.0000	1.2683	0.0150
Plekho1	0.0000	-1.1481	0.0173	0.0000	-1.4921	0.0116
Plet1	0.0000	2.1906	0.0364	0.0000	2.9046	0.0275
Plin2	0.0000	1.5423	0.0075	0.0000	1.5359	0.0088
Plin3	0.0000	1.1964	0.0092	0.0000	1.6372	0.0047
Plk1	0.0000	1.2736	0.0098	0.0000	1.3007	0.0107
Plp2	0.0000	3.7253	0.0004	0.0000	3.6906	0.0001
Plppr2	0.0000	-1.7201	0.0054	0.0000	-1.8007	0.0055
Plppr4	0.0000	2.2278	0.0240	0.0000	1.3625	0.0430
Plscr1	0.0000	1.2010	0.0229	0.0000	1.1097	0.0268
Plxdc1	0.0000	3.0211	0.0192	0.0000	4.9520	0.0075

Plxna2	0.0000	-1.1466	0.0061	0.0000	-2.2606	0.0008
Plxna3	0.0000	-1.1359	0.0141	0.0000	-2.2933	0.0030
Plxna4	0.0000	-1.1258	0.0186	0.0000	-3.3358	0.0016
Pmaip1	0.0000	4.1928	0.0008	0.0000	2.1357	0.0076
Pmf1	0.0000	1.1529	0.0174	0.0000	1.7009	0.0086
Pml	0.0000	1.7540	0.0033	0.0000	2.4405	0.0011
Pms1	0.0000	1.5129	0.0107	0.0000	1.3948	0.0142
Pnldc1	0.0000	4.0631	0.0161	0.0000	4.1536	0.0171
Pnma3	0.0000	5.3737	0.0229	0.0000	4.8610	0.0279
Pnma5	0.0000	4.1972	0.0049	0.0000	4.5201	0.0047
Pnmal1	0.0000	-1.7996	0.0106	0.0000	-1.8030	0.0116
Pnmal2	0.0000	-1.7008	0.0044	0.0000	-1.5016	0.0072
Pnoc	0.0000	-2.1720	0.0189	0.0000	-1.5233	0.0303
Pnpla7	0.0000	1.1732	0.0320	0.0000	1.3750	0.0277
Pnpt1	0.0000	2.5398	0.0013	0.0000	1.5584	0.0068
Podxl2	0.0000	-1.5702	0.0056	0.0000	-1.6004	0.0064
Pola1	0.0000	1.8187	0.0048	0.0000	1.2134	0.0145
Pold1	0.0000	1.5248	0.0062	0.0000	1.8602	0.0041
Pole	0.0000	1.9287	0.0035	0.0000	1.5534	0.0076
Pole2	0.0000	2.0903	0.0094	0.0000	1.7400	0.0156
Polr1a	0.0000	1.7028	0.0030	0.0000	1.4714	0.0056
Polr3g	0.0000	1.6726	0.0065	0.0000	1.6624	0.0079
Pon3	0.0000	4.1122	0.0304	0.0000	3.3004	0.0397
Pou2f2	0.0000	-2.2798	0.0178	0.0000	-2.5959	0.0158
Pou2f3	0.0000	4.9090	0.0141	0.0000	4.1319	0.0211
Pou3f1	0.0000	-1.1758	0.0246	0.0000	-1.6089	0.0167
Pou3f2	0.0000	-1.9694	0.0045	0.0000	-2.6569	0.0022
Pou4f1	0.0000	-1.4966	0.0154	0.0000	-2.6315	0.0054
Pou6f1	0.0000	-2.5613	0.0026	0.0000	-2.0854	0.0050
Ppan	0.0000	1.4879	0.0069	0.0000	1.6552	0.0062
Ppat	0.0000	1.4046	0.0081	0.0000	1.3051	0.0109
Ppfia2	0.0000	-1.2412	0.0208	0.0000	-2.1999	0.0081
Ppfia3	0.0000	-1.4552	0.0133	0.0000	-1.8651	0.0089
Ppm1e	0.0000	-1.5641	0.0060	0.0000	-2.2933	0.0024
Ppm1h	0.0000	-1.4268	0.0124	0.0000	-1.1509	0.0197
Ppm1j	0.0000	3.1376	0.0171	0.0000	4.1498	0.0105
Ppm1l	0.0000	-1.8005	0.0035	0.0000	-1.7929	0.0040
Ppp1r14c	0.0000	-2.3916	0.0117	0.0000	-1.7136	0.0213
Ppp1r15b	0.0000	1.0922	0.0064	0.0000	1.1317	0.0070
Ppp1r17	0.0000	-2.0812	0.0340	0.0000	-3.0432	0.0242
Ppp1r3c	0.0000	-3.2973	0.0025	0.0000	-1.2497	0.0199
Ppp1r3d	0.0000	-1.4051	0.0415	0.0001	-1.1516	0.0483
Ppp1r3e	0.0000	-1.6000	0.0415	0.0000	-1.7855	0.0384
Ppp1r9b	0.0000	-1.0896	0.0112	0.0000	-1.0602	0.0134
Ppp2r2b	0.0000	-2.2130	0.0063	0.0000	-1.7335	0.0120
Ppp2r3a	0.0000	-1.8139	0.0055	0.0000	-2.1398	0.0041
Ppp2r5b	0.0000	-1.4870	0.0064	0.0000	-1.5797	0.0063
Pqlc3	0.0000	1.1071	0.0258	0.0000	1.8239	0.0118
Prdm1	0.0000	3.7418	0.0054	0.0000	4.4275	0.0039
Prdm5	0.0000	1.5790	0.0204	0.0000	1.9952	0.0146
Prdm8	0.0000	-3.9162	0.0019	0.0000	-3.0876	0.0029
Prdx1	0.0000	1.6917	0.0018	0.0000	1.6989	0.0020
Prelid2	0.0000	3.3439	0.0088	0.0000	3.1106	0.0118
Prickle1	0.0000	-2.0099	0.0059	0.0000	-1.3619	0.0148
Prickle3	0.0000	4.6105	0.0187	0.0000	5.1682	0.0168
Prkar2a	0.0000	-1.0163	0.0083	0.0000	-1.1541	0.0070
Prkar2b	0.0000	-1.4451	0.0061	0.0000	-1.7268	0.0045
Prkca	0.0000	-1.2060	0.0266	0.0000	-1.1271	0.0295
Prkce	0.0000	-1.7768	0.0076	0.0000	-1.6114	0.0106
Prkcg	0.0000	2.3542	0.0258	0.0000	1.9630	0.0334
Prmt7	0.0000	1.3311	0.0053	0.0000	1.3896	0.0056
Prmt8	0.0000	-1.6915	0.0079	0.0000	-2.5428	0.0031
Prodh	0.0000	1.0955	0.0380	0.0000	1.2732	0.0338
Prox1	0.0000	-2.8438	0.0018	0.0000	-2.3710	0.0030
Prr16	0.0000	-3.5405	0.0370	0.0000	-3.1002	0.0397
Prr18	0.0000	-1.6249	0.0285	0.0000	-1.5371	0.0308
Prtn3	0.0000	-1.7849	0.0336	0.0000	-2.6141	0.0236
Prune2	0.0000	1.2221	0.0197	0.0000	2.4577	0.0044
Psd	0.0000	-1.9575	0.0079	0.0000	-1.7965	0.0104
Psd4	0.0000	3.2579	0.0194	0.0000	3.8642	0.0156
Ptbp1	0.0000	1.3236	0.0036	0.0000	1.2358	0.0053
Ptger3	0.0000	-3.9215	0.0175	0.0001	-1.2035	0.0483
Ptgr1	0.0000	1.0369	0.0238	0.0000	1.7453	0.0100
Ptgs2	0.0000	2.0951	0.0372	0.0000	4.1117	0.0155
Pthlh	0.0000	4.5403	0.0046	0.0000	1.2134	0.0463
Ptk2b	0.0000	2.7459	0.0159	0.0000	2.5521	0.0197
Ptpn3	0.0000	1.7582	0.0106	0.0000	1.4099	0.0182

Ptpn5	0.0000	-1.2362	0.0292	0.0000	-1.9660	0.0166
Ptpn6	0.0000	3.9622	0.0028	0.0000	3.1854	0.0066
Ptprh	0.0000	4.4108	0.0399	0.0000	3.7921	0.0466
Ptprn2	0.0000	-1.4339	0.0130	0.0000	-2.0081	0.0072
Ptpro	0.0000	-1.5042	0.0100	0.0000	-2.6839	0.0027
Ptprq	0.0000	4.3919	0.0244	0.0000	2.7718	0.0428
Ptpru	0.0000	-1.0923	0.0226	0.0000	-1.2576	0.0195
Ptprz1	0.0000	-2.7366	0.0010	0.0000	-1.8686	0.0033
Pvt1	0.0000	2.7364	0.0226	0.0000	2.9484	0.0216
Pxdn	0.0000	-1.6882	0.0027	0.0000	-1.4930	0.0046
Pxylp1	0.0000	-2.3887	0.0041	0.0000	-1.5883	0.0111
Pycard	0.0000	4.3418	0.0074	0.0000	5.3674	0.0049
Pygl	0.0000	5.5198	0.0018	0.0000	4.1384	0.0052
Pygo1	0.0000	-1.8499	0.0050	0.0000	-2.2005	0.0036
Pyroxd2	0.0000	2.5220	0.0434	0.0000	2.7855	0.0409
Rab11fip1	0.0000	1.6654	0.0095	0.0000	1.5499	0.0126
Rab27a	0.0000	3.2233	0.0042	0.0000	3.4651	0.0039
Rab30	0.0000	-2.1350	0.0059	0.0000	-2.2645	0.0059
Rab39b	0.0000	-1.1188	0.0191	0.0000	-2.1081	0.0061
Rab3a	0.0000	-1.2614	0.0127	0.0000	-1.2708	0.0139
Rab3c	0.0000	-2.7088	0.0016	0.0000	-3.0192	0.0010
Rab6b	0.0000	-1.6949	0.0036	0.0000	-2.5426	0.0010
Rad50	0.0000	1.3800	0.0071	0.0000	1.4427	0.0075
Rad54b	0.0000	1.6574	0.0112	0.0000	1.7698	0.0110
Ralgds	0.0000	-1.1114	0.0130	0.0000	-1.4982	0.0078
Ralgps1	0.0000	-1.0784	0.0193	0.0000	-1.5778	0.0105
Ranbp17	0.0000	1.5973	0.0097	0.0000	1.9470	0.0068
Rangrf	0.0000	2.0733	0.0085	0.0000	1.6887	0.0151
Rapgef5	0.0000	-1.4642	0.0120	0.0000	-1.8478	0.0082
Rapgefl1	0.0000	-1.2273	0.0339	0.0000	-1.6331	0.0258
Rarb	0.0000	-5.4590	0.0034	0.0000	-1.2084	0.0304
Rasd2	0.0000	1.9232	0.0322	0.0000	3.1977	0.0164
Rasgef1a	0.0003	1.9175	0.0489	0.0000	2.5833	0.0391
Rasgef1c	0.0000	-3.2246	0.0281	0.0000	-3.4618	0.0265
Rasgrf2	0.0000	-2.9707	0.0226	0.0000	-3.0900	0.0220
Rasgrp1	0.0000	3.9423	0.0081	0.0000	2.8690	0.0178
Rasgrp2	0.0000	1.7744	0.0188	0.0000	3.0239	0.0066
Rasgrp4	0.0000	2.0444	0.0287	0.0000	3.3306	0.0140
Rasl10b	0.0000	-1.9405	0.0044	0.0000	-2.2597	0.0034
Rasl11a	0.0001	-1.4953	0.0462	0.0000	-1.6119	0.0443
Rassf2	0.0000	-1.1946	0.0109	0.0000	-1.3635	0.0093
Rassf3	0.0000	1.1286	0.0176	0.0000	1.6061	0.0095
Rbfox1	0.0000	-1.7628	0.0157	0.0000	-2.5787	0.0083
Rbfox2	0.0000	-1.0543	0.0071	0.0000	-1.0827	0.0079
Rbfox3	0.0000	-2.8186	0.0035	0.0000	-2.9028	0.0035
Rbm43	0.0000	2.2242	0.0290	0.0000	2.2964	0.0292
Rbm47	0.0000	3.6308	0.0058	0.0000	2.6793	0.0140
Rbms3	0.0000	-2.4535	0.0043	0.0000	-1.0412	0.0246
Rbp1	0.0000	-3.7807	0.0009	0.0000	-1.8752	0.0068
Rbpms	0.0000	2.3861	0.0017	0.0000	2.9347	0.0007
Rbpms2	0.0000	2.7192	0.0012	0.0000	4.0547	0.0002
Rcan2	0.0000	-2.8522	0.0010	0.0000	-2.4463	0.0015
Rcl1	0.0000	1.3781	0.0120	0.0000	1.2461	0.0164
Rdm1	0.0000	1.1631	0.0227	0.0000	2.4838	0.0049
Rec8	0.0000	4.1958	0.0031	0.0000	4.3578	0.0032
Reep1	0.0000	-1.0261	0.0151	0.0000	-1.7854	0.0049
Reep2	0.0000	-1.2892	0.0209	0.0000	-1.4275	0.0190
Relb	0.0000	1.7955	0.0166	0.0000	1.9934	0.0150
Rell2	0.0000	-1.4751	0.0278	0.0000	-1.2543	0.0336
Rem2	0.0000	-1.7551	0.0128	0.0000	-2.8364	0.0053
Renbp	0.0000	1.5305	0.0081	0.0000	2.2882	0.0030
Reps2	0.0000	-2.0463	0.0160	0.0000	-1.2122	0.0325
Resp18	0.0000	-1.1444	0.0391	0.0000	-1.6404	0.0283
Rest	0.0000	1.4558	0.0074	0.0000	1.8707	0.0044
Ret	0.0000	-1.5108	0.0206	0.0000	-2.2037	0.0119
Retsat	0.0000	2.6983	0.0074	0.0000	2.9282	0.0070
Rev3l	0.0000	-1.1039	0.0133	0.0000	-1.7375	0.0054
Rfc5	0.0000	1.7107	0.0034	0.0000	1.7104	0.0040
Rfx2	0.0000	1.1526	0.0218	0.0000	1.2772	0.0199
Rfx4	0.0000	-1.2445	0.0088	0.0000	-1.9836	0.0030
Rgma	0.0000	-2.6378	0.0012	0.0000	-1.1675	0.0125
Rgs17	0.0000	-1.2564	0.0355	0.0000	-1.8561	0.0242
Rgs7	0.0000	-2.0013	0.0207	0.0000	-2.1679	0.0194
Rgs7bp	0.0000	-2.0770	0.0185	0.0000	-1.3696	0.0316
Rgs8	0.0000	-1.1572	0.0197	0.0000	-2.0657	0.0073
Rhbdl3	0.0000	-3.5356	0.0018	0.0000	-2.7118	0.0037
Rhob	0.0000	-1.2890	0.0052	0.0000	-1.2238	0.0069

Ribc1	0.0000	1.1357	0.0425	0.0000	1.2324	0.0407
Rimklb	0.0000	-1.0012	0.0105	0.0000	-1.2504	0.0073
Rims4	0.0000	-1.6022	0.0185	0.0000	-1.0252	0.0333
Rin1	0.0000	1.8094	0.0261	0.0000	2.2565	0.0200
Ripk3	0.0000	3.2152	0.0323	0.0000	3.4379	0.0313
Rit2	0.0000	2.3516	0.0329	0.0000	1.6991	0.0459
Rmi2	0.0000	1.0625	0.0373	0.0000	1.5208	0.0259
Rnaseh2a	0.0000	1.1686	0.0130	0.0000	1.0207	0.0185
Rnd2	0.0000	-1.3618	0.0132	0.0000	-2.4271	0.0038
Rnd3	0.0000	-2.5418	0.0005	0.0000	-1.7395	0.0015
Rnf112	0.0000	-1.8209	0.0343	0.0000	-2.0880	0.0310
Rnf122	0.0000	-1.7183	0.0077	0.0000	-1.4394	0.0124
Rnf125	0.0000	4.0890	0.0177	0.0000	4.0566	0.0196
Rnf135	0.0000	2.4988	0.0252	0.0000	2.6056	0.0252
Rnf150	0.0000	-1.4166	0.0136	0.0000	-1.1220	0.0212
Rnf157	0.0000	-1.9393	0.0033	0.0000	-1.3427	0.0093
Rnf165	0.0000	-1.8247	0.0049	0.0000	-1.8595	0.0055
Rnf187	0.0000	-1.1204	0.0046	0.0000	-1.0500	0.0066
Rnf24	0.0000	-1.0855	0.0168	0.0000	-1.5488	0.0093
Rnft2	0.0000	-1.2258	0.0145	0.0000	-1.2686	0.0148
Rnls	0.0000	2.0223	0.0390	0.0000	2.1750	0.0374
Robo1	0.0000	-1.6573	0.0037	0.0000	-1.8326	0.0032
Robo2	0.0000	-1.2509	0.0115	0.0000	-2.4763	0.0023
Ror1	0.0000	1.8532	0.0150	0.0000	1.3334	0.0270
Rorb	0.0000	-1.2722	0.0451	0.0000	-2.3321	0.0271
Rpa1	0.0000	1.0881	0.0069	0.0000	1.1940	0.0065
Rpa3	0.0000	1.2252	0.0171	0.0000	1.0010	0.0250
Rph3a	0.0000	-2.3390	0.0224	0.0000	-1.9940	0.0275
Rpl12	0.0000	4.9194	0.0001	0.0000	1.0991	0.0127
Rpl13	0.0000	3.9938	0.0004	0.0000	1.0608	0.0095
Rpl18a	0.0000	1.9361	0.0018	0.0000	1.2979	0.0068
Rpl28	0.0000	2.0118	0.0033	0.0000	1.0039	0.0192
Rpl39l	0.0000	4.9830	0.0032	0.0000	4.5041	0.0052
Rpl3l	0.0003	3.7830	0.0491	0.0000	4.9408	0.0406
Rpp25	0.0000	1.5398	0.0124	0.0000	3.1190	0.0020
Rpp40	0.0000	1.1629	0.0300	0.0000	1.3869	0.0253
Rps2	0.0000	1.5239	0.0045	0.0000	1.3050	0.0081
Rps6ka1	0.0000	1.2895	0.0096	0.0000	1.9272	0.0038
Rrp15	0.0000	1.3965	0.0079	0.0000	1.4468	0.0083
Rrp1b	0.0000	1.3245	0.0080	0.0000	1.2491	0.0103
Rspo2	0.0000	5.4560	0.0005	0.0000	3.4631	0.0022
Rtn1	0.0000	-1.3411	0.0059	0.0000	-1.7253	0.0035
Rtn2	0.0000	-1.1686	0.0150	0.0000	-1.8658	0.0063
Rtn4rl1	0.0000	1.8152	0.0178	0.0000	1.5817	0.0237
Rtp4	0.0000	4.7218	0.0255	0.0000	4.7167	0.0270
Rufy3	0.0000	-1.1954	0.0047	0.0000	-1.9209	0.0012
Runx1	0.0000	-2.2766	0.0243	0.0001	-1.1194	0.0470
Runx1t1	0.0000	-2.0620	0.0090	0.0000	-1.6742	0.0152
Rusc1	0.0000	-1.2394	0.0149	0.0000	-1.8927	0.0070
S100a1	0.0000	1.4919	0.0400	0.0000	4.7581	0.0057
S100a6	0.0000	1.3272	0.0402	0.0000	2.9143	0.0145
Sall2	0.0000	-1.2461	0.0079	0.0000	-1.4806	0.0060
Sall3	0.0000	-1.3938	0.0088	0.0000	-2.1600	0.0034
Sall4	0.0000	2.4471	0.0030	0.0000	2.7841	0.0023
Samd10	0.0000	-1.6533	0.0095	0.0000	-1.8541	0.0081
Samd14	0.0000	-2.0890	0.0022	0.0000	-2.3067	0.0018
Samd9l	0.0000	2.2330	0.0318	0.0000	2.6830	0.0268
Sarm1	0.0000	-1.7803	0.0095	0.0000	-1.9950	0.0082
Sat1	0.0000	1.2750	0.0135	0.0000	1.4871	0.0108
Sbk1	0.0000	-1.9437	0.0012	0.0000	-2.2669	0.0006
Scarf1	0.0000	3.1221	0.0293	0.0000	4.2890	0.0199
Scg3	0.0000	-2.1127	0.0022	0.0000	-2.7229	0.0009
Scg5	0.0000	-1.6010	0.0180	0.0000	-2.2001	0.0111
Scn1a	0.0000	-2.1386	0.0356	0.0000	-2.8013	0.0287
Scn2a	0.0000	-1.5532	0.0148	0.0000	-2.6376	0.0056
Scn2b	0.0000	-1.7985	0.0232	0.0000	-1.1753	0.0372
Scn3a	0.0000	-1.1671	0.0145	0.0000	-2.2635	0.0035
Scn3b	0.0000	-2.1532	0.0023	0.0000	-1.7807	0.0045
Scn5a	0.0002	-2.0808	0.0478	0.0000	-2.3768	0.0442
Scn9a	0.0000	-1.4764	0.0349	0.0000	-4.2530	0.0108
Scrt1	0.0000	-1.7801	0.0048	0.0000	-2.7490	0.0014
Scrt2	0.0000	-2.4794	0.0026	0.0000	-3.6624	0.0007
Scube1	0.0000	-2.5900	0.0020	0.0000	-2.0670	0.0040
Scube2	0.0000	-1.8788	0.0296	0.0000	-1.9613	0.0292
Sdc3	0.0000	-1.6280	0.0036	0.0000	-1.3681	0.0068
Sdc4	0.0000	1.2834	0.0087	0.0000	2.5713	0.0010
Sdk2	0.0000	-1.7392	0.0047	0.0000	-1.9824	0.0038

Sdr39u1	0.0000	4.3318	0.0093	0.0000	4.2807	0.0108
Sec14l4	0.0000	5.3117	0.0297	0.0000	5.0314	0.0332
Sec14l5	0.0000	-1.6689	0.0329	0.0000	-1.8972	0.0297
Sema3a	0.0000	-1.8419	0.0133	0.0000	-1.3569	0.0233
Sema3d	0.0000	-1.6418	0.0351	0.0001	-1.1141	0.0487
Sema5a	0.0000	-1.8632	0.0147	0.0000	-1.3092	0.0266
Sema6b	0.0000	-2.0069	0.0098	0.0000	-1.6522	0.0151
Sema6c	0.0000	-1.4541	0.0075	0.0000	-1.8295	0.0048
Sema6d	0.0000	-1.0204	0.0191	0.0000	-2.5243	0.0029
Senp7	0.0000	-1.0193	0.0160	0.0000	-1.7937	0.0053
Sephs2	0.0000	1.1055	0.0087	0.0000	1.3366	0.0062
Serp2	0.0000	-1.1233	0.0451	0.0000	-1.1186	0.0458
Serpinb8	0.0000	3.2387	0.0410	0.0000	3.5030	0.0394
Serpine1	0.0000	1.2710	0.0275	0.0000	1.9759	0.0146
Serpini1	0.0000	-2.1786	0.0033	0.0000	-3.0121	0.0013
Sestd1	0.0000	-1.3826	0.0078	0.0000	-1.9584	0.0036
Setbp1	0.0000	-1.3590	0.0065	0.0000	-1.9543	0.0028
Setd6	0.0000	1.1365	0.0245	0.0000	1.9361	0.0101
Sez6	0.0000	-1.5714	0.0094	0.0000	-2.4004	0.0037
Sez6l	0.0000	-1.6662	0.0087	0.0000	-2.4899	0.0037
Sez6l2	0.0000	-1.4414	0.0094	0.0000	-2.2663	0.0035
Sfn	0.0000	1.7645	0.0288	0.0000	1.3454	0.0397
Sfrp4	0.0003	1.2587	0.0489	0.0000	2.6230	0.0237
Sgip1	0.0000	-1.7769	0.0083	0.0000	-2.9162	0.0027
Sgk1	0.0000	2.6510	0.0017	0.0000	1.8291	0.0061
Sgk3	0.0000	3.0529	0.0057	0.0000	4.3361	0.0023
Sgms2	0.0000	2.9236	0.0078	0.0000	1.2337	0.0357
Sgtb	0.0000	-1.3785	0.0117	0.0000	-1.6540	0.0090
Sh2b2	0.0000	-1.4917	0.0191	0.0000	-2.0527	0.0120
Sh2d3c	0.0000	-1.5911	0.0103	0.0000	-1.9481	0.0075
Sh2d4a	0.0000	4.7529	0.0253	0.0000	6.4517	0.0170
Sh2d5	0.0000	-2.1668	0.0236	0.0000	-1.4606	0.0360
Sh3bgrl	0.0000	-1.2765	0.0055	0.0000	-1.2815	0.0064
Sh3bgrl2	0.0000	1.6408	0.0148	0.0000	2.1276	0.0095
Sh3bp1	0.0000	1.2878	0.0178	0.0000	2.0161	0.0077
Sh3bp5	0.0000	-1.5738	0.0096	0.0000	-1.6426	0.0098
Sh3tc1	0.0000	3.3478	0.0091	0.0000	4.3986	0.0051
Sh3yl1	0.0000	-1.2461	0.0219	0.0000	-1.8316	0.0127
Shank1	0.0000	-1.5660	0.0106	0.0000	-2.3326	0.0048
Shcbp1	0.0000	1.4912	0.0081	0.0000	1.1720	0.0154
Shd	0.0000	-1.5383	0.0144	0.0000	-2.3176	0.0071
She	0.0000	1.5812	0.0333	0.0000	3.1388	0.0124
Shf	0.0000	-1.4095	0.0142	0.0000	-1.6427	0.0118
Shisa7	0.0000	-1.4948	0.0163	0.0000	-1.8539	0.0121
Shisa8	0.0000	2.1187	0.0321	0.0000	1.8691	0.0378
Shisa9	0.0000	-1.3451	0.0338	0.0000	-1.6411	0.0283
Shmt1	0.0000	2.4675	0.0020	0.0000	2.4124	0.0026
Shmt2	0.0000	1.2142	0.0063	0.0000	1.5111	0.0040
Siah3	0.0000	-2.4017	0.0214	0.0000	-3.1211	0.0158
Sigirr	0.0000	3.8823	0.0147	0.0000	4.6606	0.0111
Sim1	0.0000	-5.8958	0.0117	0.0000	-5.4441	0.0104
Ska3	0.0000	1.3897	0.0140	0.0000	1.3481	0.0164
Slc10a4	0.0000	-2.8716	0.0269	0.0000	-2.5787	0.0294
Slc12a5	0.0000	-2.8503	0.0026	0.0000	-2.5429	0.0038
Slc12a8	0.0000	3.9877	0.0108	0.0000	4.8955	0.0077
Slc13a5	0.0000	3.2936	0.0208	0.0000	3.8296	0.0175
Slc15a1	0.0000	4.3441	0.0218	0.0000	5.9964	0.0135
Slc16a10	0.0000	3.2892	0.0032	0.0000	1.0614	0.0341
Slc16a6	0.0000	1.5884	0.0068	0.0000	1.8351	0.0054
Slc18a3	0.0000	-3.2538	0.0124	0.0000	-3.5930	0.0112
Slc19a3	0.0000	2.6797	0.0348	0.0000	4.1660	0.0206
Slc1a2	0.0000	-1.4794	0.0065	0.0000	-3.0507	0.0008
Slc1a5	0.0000	1.6327	0.0078	0.0000	2.9478	0.0013
Slc20a2	0.0000	1.1990	0.0101	0.0000	1.7616	0.0042
Slc22a15	0.0000	-2.1234	0.0175	0.0000	-1.9884	0.0200
Slc22a17	0.0000	-1.1412	0.0092	0.0000	-1.3552	0.0070
Slc22a6	0.0000	-2.6330	0.0387	0.0000	-2.9283	0.0361
Slc24a3	0.0000	-1.6917	0.0179	0.0000	-1.5925	0.0206
Slc25a13	0.0000	2.3745	0.0026	0.0000	2.7739	0.0018
Slc25a15	0.0000	1.1276	0.0149	0.0000	1.0726	0.0179
Slc25a27	0.0000	-1.5011	0.0192	0.0000	-1.9576	0.0134
Slc25a31	0.0000	8.2256	0.0041	0.0000	6.2535	0.0098
Slc25a43	0.0000	4.5309	0.0364	0.0000	4.8004	0.0360
Slc25a5	0.0000	1.2380	0.0046	0.0000	1.7494	0.0019
Slc27a2	0.0000	2.7324	0.0200	0.0000	2.6949	0.0218
Slc29a1	0.0000	2.5127	0.0021	0.0000	2.5529	0.0023
Slc29a4	0.0000	-1.2910	0.0137	0.0000	-1.5183	0.0109

Slc2a1	0.0000	1.0132	0.0066	0.0000	1.1630	0.0055
Slc2a3	0.0000	1.9917	0.0008	0.0000	2.8454	0.0001
Slc2a5	0.0000	3.4727	0.0410	0.0000	3.7777	0.0391
Slc32a1	0.0000	-2.4330	0.0124	0.0000	-2.4204	0.0138
Slc35d2	0.0000	2.2878	0.0361	0.0000	2.6254	0.0326
Slc35f2	0.0000	3.1516	0.0026	0.0000	3.8363	0.0015
Slc35f4	0.0001	-1.6977	0.0460	0.0000	-2.2087	0.0383
Slc35g1	0.0000	1.0850	0.0332	0.0000	1.3911	0.0261
Slc36a4	0.0000	-1.0749	0.0182	0.0000	-1.3237	0.0138
Slc37a2	0.0000	1.6087	0.0278	0.0000	2.8932	0.0108
Slc37a4	0.0000	-1.4285	0.0153	0.0000	-1.3994	0.0170
Slc38a6	0.0000	13.0195	0.0000	0.0000	4.4200	0.0000
Slc38a7	0.0000	1.0394	0.0167	0.0000	1.4615	0.0091
Slc39a4	0.0000	3.4094	0.0060	0.0000	4.9398	0.0023
Slc3a2	0.0000	1.6557	0.0020	0.0000	1.8743	0.0013
Slc40a1	0.0000	1.9211	0.0097	0.0000	1.9459	0.0107
Slc43a3	0.0003	1.2817	0.0489	0.0000	2.9753	0.0201
Slc44a1	0.0000	1.8772	0.0217	0.0000	2.1765	0.0188
Slc47a1	0.0000	7.9322	0.0051	0.0000	7.6419	0.0068
Slc4a10	0.0000	2.7564	0.0130	0.0000	1.4046	0.0370
Slc4a11	0.0000	3.3899	0.0115	0.0000	3.9995	0.0089
Slc4a5	0.0000	7.2530	0.0024	0.0000	4.8991	0.0087
Slc4a8	0.0000	-1.3552	0.0115	0.0000	-1.6785	0.0081
Slc50a1	0.0000	2.9886	0.0132	0.0000	3.6609	0.0094
Slc52a3	0.0000	4.4502	0.0262	0.0000	3.7388	0.0337
Slc5a1	0.0000	3.7106	0.0331	0.0000	3.1436	0.0406
Slc5a11	0.0000	3.4454	0.0214	0.0000	3.7314	0.0203
Slc6a1	0.0000	-2.7412	0.0019	0.0000	-1.5738	0.0084
Slc6a11	0.0000	-4.7278	0.0072	0.0000	-1.7416	0.0276
Slc6a13	0.0000	-2.8430	0.0156	0.0000	-2.9682	0.0153
Slc6a5	0.0000	-4.8393	0.0252	0.0000	-4.4813	0.0286
Slc7a1	0.0000	1.6240	0.0036	0.0000	1.3541	0.0070
Slc7a14	0.0000	-2.1119	0.0145	0.0000	-2.0658	0.0160
Slc7a3	0.0000	3.6509	0.0006	0.0000	3.3616	0.0006
Slc7a5	0.0000	1.3752	0.0036	0.0000	2.1624	0.0008
Slc7a7	0.0000	3.1165	0.0043	0.0000	2.5588	0.0087
Slc8a1	0.0000	-1.2176	0.0140	0.0000	-2.4422	0.0030
Slco1c1	0.0000	6.8421	0.0230	0.0000	6.1344	0.0283
Slco4c1	0.0000	3.0724	0.0184	0.0000	4.6305	0.0086
Slco5a1	0.0000	-1.2572	0.0110	0.0000	-3.0831	0.0011
Slfn5	0.0004	4.2613	0.0498	0.0000	6.0164	0.0384
Slit1	0.0000	-2.4584	0.0008	0.0000	-2.0918	0.0013
Slitrk1	0.0000	-2.0802	0.0174	0.0000	-3.7768	0.0072
Slitrk2	0.0000	-2.3340	0.0205	0.0000	-2.6180	0.0184
Slitrk3	0.0000	-2.8692	0.0188	0.0000	-3.5422	0.0151
Slitrk4	0.0000	-3.2271	0.0236	0.0000	-2.7863	0.0270
Slitrk5	0.0000	-1.1214	0.0193	0.0000	-2.1372	0.0060
Smagp	0.0000	3.5461	0.0093	0.0000	4.3085	0.0065
Smarcc2	0.0000	-1.1134	0.0096	0.0000	-1.9113	0.0026
Smc1b	0.0000	2.8921	0.0096	0.0000	4.9111	0.0024
Smc2	0.0000	1.7471	0.0022	0.0000	1.1401	0.0087
Smim18	0.0000	-4.6399	0.0200	0.0000	-3.4642	0.0230
Smim3	0.0000	1.7050	0.0334	0.0000	1.7133	0.0345
Smoc2	0.0000	-4.3288	0.0055	0.0000	-1.2873	0.0354
Smtnl2	0.0000	2.4484	0.0033	0.0000	2.5960	0.0032
Smyd1	0.0001	1.5015	0.0470	0.0000	2.0642	0.0364
Snai3	0.0000	5.2461	0.0361	0.0000	4.3040	0.0449
Snap25	0.0000	-1.8402	0.0057	0.0000	-2.2350	0.0040
Snap91	0.0000	-1.7213	0.0085	0.0000	-1.9742	0.0070
Snca	0.0000	-1.9653	0.0124	0.0000	-1.4895	0.0206
Sncaip	0.0000	-2.1957	0.0063	0.0000	-1.8685	0.0099
Sncb	0.0000	-2.7277	0.0179	0.0000	-1.7334	0.0319
Sncg	0.0000	-1.8876	0.0082	0.0000	-3.7191	0.0018
Snn	0.0000	-1.5737	0.0037	0.0000	-1.8649	0.0026
Snph	0.0000	-1.6337	0.0282	0.0000	-2.0043	0.0229
Snx20	0.0000	2.8331	0.0324	0.0000	3.9760	0.0219
Snx32	0.0000	-1.4156	0.0352	0.0000	-1.5582	0.0329
Soat2	0.0000	2.7733	0.0429	0.0000	3.3463	0.0372
Sobp	0.0000	-1.6667	0.0064	0.0000	-1.7351	0.0067
Sod1	0.0000	1.3128	0.0039	0.0000	1.1409	0.0067
Soga3	0.0000	-1.2981	0.0070	0.0000	-2.8460	0.0007
Sorbs1	0.0000	-1.0742	0.0232	0.0000	-1.2170	0.0206
Sowahb	0.0000	2.0552	0.0381	0.0000	3.2656	0.0229
Sox11	0.0000	-1.8010	0.0022	0.0000	-2.7923	0.0003
Sox3	0.0000	-1.3149	0.0215	0.0000	-1.9785	0.0117
Sox4	0.0000	-1.9303	0.0015	0.0000	-1.7053	0.0025
Sox5	0.0000	-1.0586	0.0329	0.0000	-1.8408	0.0172

Sox8	0.0000	-2.4409	0.0208	0.0000	-1.1824	0.0437
Sox9	0.0000	-1.3114	0.0121	0.0000	-1.1833	0.0164
Sp5	0.0000	3.3100	0.0090	0.0000	1.2960	0.0408
Sp8	0.0000	-2.4723	0.0087	0.0000	-2.2151	0.0120
Sp9	0.0000	-4.5569	0.0228	0.0000	-2.6003	0.0322
Spag1	0.0000	1.3091	0.0339	0.0000	1.0696	0.0424
Spag16	0.0000	4.1490	0.0156	0.0000	4.1457	0.0172
Spag9	0.0000	-1.3579	0.0029	0.0000	-2.1806	0.0005
Spata5	0.0000	1.1341	0.0133	0.0000	1.1087	0.0154
Spc24	0.0000	1.2483	0.0135	0.0000	2.0508	0.0046
Spef2	0.0000	2.3632	0.0273	0.0000	1.5547	0.0436
Sphkap	0.0000	-3.0664	0.0180	0.0000	-2.5053	0.0229
Spidr	0.0000	1.3510	0.0087	0.0000	1.2475	0.0118
Spint2	0.0000	2.9655	0.0010	0.0000	2.6654	0.0016
Spire1	0.0000	-1.1411	0.0106	0.0000	-1.2687	0.0096
Spn	0.0000	2.5504	0.0311	0.0000	2.7339	0.0301
Spns2	0.0000	-1.6432	0.0214	0.0000	-2.3659	0.0129
Spock1	0.0000	-1.8258	0.0065	0.0000	-2.9734	0.0020
Spock2	0.0000	-2.0819	0.0013	0.0000	-1.3651	0.0051
Spon1	0.0000	-4.8117	0.0003	0.0000	-1.9853	0.0010
Spp1	0.0000	3.1807	0.0005	0.0000	5.5215	0.0001
Spry4	0.0000	1.9860	0.0158	0.0000	1.6513	0.0232
Spsb4	0.0000	-2.2773	0.0051	0.0000	-3.2478	0.0022
Sptbn2	0.0000	-1.7457	0.0026	0.0000	-2.9681	0.0003
Srcin1	0.0000	-1.4726	0.0089	0.0000	-2.0754	0.0044
Srd5a1	0.0000	-1.9532	0.0159	0.0000	-1.8906	0.0176
Srgap3	0.0000	-1.4438	0.0036	0.0000	-2.1428	0.0010
Srpx	0.0000	2.5159	0.0220	0.0000	2.9373	0.0185
Srr	0.0000	-1.2532	0.0139	0.0000	-1.5246	0.0103
Srrm3	0.0000	-1.2681	0.0216	0.0000	-2.8382	0.0052
Srrm4	0.0000	-1.8898	0.0054	0.0000	-3.6379	0.0008
Ss18l1	0.0000	-1.7890	0.0049	0.0000	-2.2738	0.0029
Ssbp4	0.0000	1.4296	0.0062	0.0000	1.3964	0.0078
Sst	0.0000	-1.6142	0.0166	0.0000	-2.8552	0.0060
Sstr1	0.0000	-4.2706	0.0301	0.0000	-2.8995	0.0373
Sstr2	0.0000	-1.8239	0.0217	0.0000	-3.4054	0.0091
Sstr3	0.0000	-4.7985	0.0362	0.0000	-3.8072	0.0371
Sstr4	0.0000	-4.5695	0.0321	0.0000	-2.2973	0.0457
St14	0.0000	2.5589	0.0037	0.0000	3.7159	0.0011
St18	0.0000	-1.8974	0.0069	0.0000	-2.7145	0.0032
St3gal1	0.0000	-2.3223	0.0064	0.0000	-1.8334	0.0117
St6gal2	0.0000	-1.8191	0.0143	0.0000	-2.2477	0.0105
St6galnac5	0.0000	-3.0959	0.0034	0.0000	-3.6342	0.0025
St8sia1	0.0000	-1.5288	0.0089	0.0000	-1.7618	0.0074
St8sia2	0.0000	-1.2467	0.0064	0.0000	-1.9150	0.0023
St8sia3	0.0000	-1.1545	0.0237	0.0000	-1.8498	0.0121
St8sia4	0.0000	-1.5972	0.0102	0.0000	-2.8963	0.0028
St8sia5	0.0000	-3.0335	0.0343	0.0000	-2.0083	0.0453
St8sia6	0.0000	4.5522	0.0110	0.0000	4.2679	0.0142
Stag3	0.0000	1.8685	0.0058	0.0000	1.7367	0.0082
Stard9	0.0000	-1.4645	0.0205	0.0000	-1.0007	0.0334
Stat1	0.0000	1.4730	0.0113	0.0000	1.0389	0.0232
Stat4	0.0000	4.7120	0.0142	0.0000	4.5593	0.0169
Stc2	0.0000	1.7093	0.0070	0.0000	1.5087	0.0109
Steap1	0.0000	2.9097	0.0161	0.0000	3.4092	0.0130
Steap3	0.0000	2.6421	0.0175	0.0000	3.9175	0.0083
Steap4	0.0000	4.6465	0.0254	0.0000	7.5923	0.0120
Stk17b	0.0000	1.9473	0.0048	0.0000	2.3463	0.0032
Stk32b	0.0000	-2.4148	0.0129	0.0000	-2.5600	0.0127
Stmn2	0.0000	-1.1042	0.0044	0.0000	-1.4992	0.0021
Stmn3	0.0000	-1.4421	0.0050	0.0000	-2.2604	0.0014
Stra6	0.0000	-2.4705	0.0145	0.0000	-2.2544	0.0179
Stx1a	0.0000	-1.2311	0.0261	0.0000	-1.4083	0.0230
Stx1b	0.0000	-1.7720	0.0072	0.0000	-2.2047	0.0048
Stx3	0.0000	2.2332	0.0033	0.0000	2.3160	0.0034
Stxbp1	0.0000	-2.0779	0.0015	0.0000	-2.0252	0.0017
Stxbp2	0.0000	2.9427	0.0022	0.0000	2.9299	0.0026
Stxbp5l	0.0000	-2.1740	0.0392	0.0000	-4.1369	0.0252
Suclg2	0.0000	1.4215	0.0060	0.0000	1.4280	0.0071
Sv2a	0.0000	-1.7088	0.0060	0.0000	-1.5132	0.0091
Sv2c	0.0000	-3.1628	0.0049	0.0000	-1.7443	0.0167
Svop	0.0000	-2.1710	0.0042	0.0000	-2.2646	0.0042
Swap70	0.0000	1.0698	0.0147	0.0000	1.3652	0.0099
Syce1	0.0000	5.1207	0.0096	0.0000	4.4146	0.0150
Sycp1	0.0000	2.7169	0.0197	0.0000	1.7744	0.0359
Sycp2	0.0000	4.9530	0.0225	0.0000	3.8765	0.0328
Sycp3	0.0000	2.6167	0.0090	0.0000	4.6898	0.0017

Syk	0.0000	3.5337	0.0014	0.0000	3.1180	0.0023
Syn2	0.0000	-1.2777	0.0261	0.0000	-1.2616	0.0273
Syn3	0.0000	-1.4587	0.0347	0.0000	-2.5492	0.0196
Syndig1l	0.0000	-2.5183	0.0372	0.0000	-2.0023	0.0440
Syngr3	0.0000	-1.3380	0.0230	0.0000	-1.3641	0.0236
Synpo2	0.0000	-2.3739	0.0119	0.0000	-1.2542	0.0312
Synpr	0.0000	-2.2909	0.0133	0.0000	-2.1565	0.0156
Syt1	0.0000	-2.0890	0.0030	0.0000	-2.7108	0.0015
Syt11	0.0000	-1.4541	0.0030	0.0000	-1.8565	0.0016
Syt16	0.0000	-1.6329	0.0313	0.0000	-1.6110	0.0327
Syt2	0.0000	-2.2197	0.0186	0.0000	-2.8308	0.0137
Syt4	0.0000	-1.4098	0.0060	0.0000	-2.3778	0.0015
Syt6	0.0000	-1.2720	0.0442	0.0000	-2.3084	0.0265
Syt9	0.0000	2.6469	0.0054	0.0000	3.1899	0.0036
Tacr1	0.0000	-2.8844	0.0059	0.0000	-1.1447	0.0285
Tada2a	0.0000	1.0146	0.0173	0.0000	1.0251	0.0185
Taf4b	0.0000	3.0531	0.0060	0.0000	2.9859	0.0076
Taf5l	0.0000	1.2787	0.0069	0.0000	1.5986	0.0044
Taf7	0.0000	1.0142	0.0105	0.0000	1.1783	0.0086
Taf7l	0.0000	5.5113	0.0058	0.0000	6.0808	0.0054
Tafa2	0.0000	-1.7840	0.0312	0.0000	-1.9877	0.0289
Tagln2	0.0000	1.5706	0.0042	0.0000	2.6895	0.0007
Tagln3	0.0000	-1.8800	0.0021	0.0000	-2.7832	0.0005
Tal1	0.0000	-3.7173	0.0038	0.0000	-3.1496	0.0052
Tanc1	0.0000	1.3290	0.0061	0.0000	1.2674	0.0082
Tanc2	0.0000	-1.6008	0.0050	0.0000	-2.0295	0.0030
Tango6	0.0000	1.3471	0.0139	0.0000	1.8309	0.0079
Tap2	0.0000	1.0859	0.0364	0.0000	1.4262	0.0280
Tars	0.0000	1.1990	0.0057	0.0000	1.0396	0.0096
Tbc1d16	0.0000	-1.5892	0.0023	0.0000	-1.8737	0.0014
Tbc1d2	0.0000	1.9157	0.0206	0.0000	1.4634	0.0312
Tbc1d30	0.0000	-1.2804	0.0221	0.0000	-2.9649	0.0052
Tbrg1	0.0000	1.1263	0.0063	0.0000	1.5288	0.0031
Tbx3	0.0000	1.8057	0.0064	0.0000	3.0245	0.0014
Tcaf1	0.0000	-1.3153	0.0031	0.0000	-2.2052	0.0004
Tcea3	0.0000	4.3812	0.0030	0.0000	3.5325	0.0068
Tcf15	0.0000	1.1970	0.0337	0.0000	1.7846	0.0213
Tcf24	0.0000	2.8471	0.0233	0.0000	4.5844	0.0104
Tcf4	0.0000	-1.3159	0.0054	0.0000	-1.1738	0.0082
Tcf7	0.0000	2.4603	0.0034	0.0000	2.6495	0.0031
Tcof1	0.0000	1.3424	0.0043	0.0000	1.2634	0.0061
Tcp11l2	0.0000	-2.5404	0.0121	0.0000	-1.5801	0.0250
Tdrd1	0.0000	2.8998	0.0368	0.0000	2.8468	0.0387
Tdrd12	0.0000	3.8989	0.0031	0.0000	2.6459	0.0105
Tdrd5	0.0000	3.6587	0.0249	0.0000	2.7699	0.0363
Tdrp	0.0000	3.6377	0.0013	0.0000	3.7374	0.0012
Tead4	0.0000	2.6108	0.0092	0.0000	3.2906	0.0058
Tekt1	0.0000	5.9899	0.0204	0.0000	5.6277	0.0241
Tenm1	0.0000	-2.4648	0.0189	0.0000	-3.1115	0.0143
Tent5a	0.0000	-2.2586	0.0132	0.0000	-1.4415	0.0272
Tep1	0.0000	1.0777	0.0283	0.0000	1.1327	0.0277
Tesk1	0.0000	-1.1388	0.0140	0.0000	-1.0531	0.0174
Tesk2	0.0000	1.7195	0.0181	0.0000	1.7071	0.0198
Tet3	0.0000	-1.3108	0.0056	0.0000	-2.1658	0.0014
Tex14	0.0000	4.9601	0.0016	0.0000	5.1293	0.0014
Tfcp2	0.0000	-1.0040	0.0167	0.0000	-1.1255	0.0149
Tfcp2l1	0.0000	5.2162	0.0004	0.0000	5.5192	0.0001
Tfe3	0.0000	1.3995	0.0052	0.0000	1.3438	0.0068
Tgfa	0.0000	-1.6458	0.0298	0.0000	-1.7169	0.0291
Tgif1	0.0000	1.3636	0.0072	0.0000	1.9350	0.0031
Tgm1	0.0000	3.8817	0.0163	0.0000	3.0303	0.0259
Tgm3	0.0000	3.3399	0.0238	0.0000	1.9088	0.0455
Thbs4	0.0000	4.7168	0.0328	0.0000	3.7705	0.0426
Thnsl2	0.0000	1.7353	0.0323	0.0000	1.6440	0.0355
Thra	0.0000	-1.5923	0.0045	0.0000	-1.0822	0.0131
Thsd7a	0.0000	-1.0980	0.0137	0.0000	-2.6425	0.0015
Timp1	0.0000	4.1007	0.0033	0.0000	5.2514	0.0017
Tjp3	0.0000	1.2306	0.0286	0.0000	1.8563	0.0165
Tktl1	0.0000	6.2700	0.0344	0.0001	4.6408	0.0468
Tle1	0.0000	-1.0464	0.0117	0.0000	-1.0473	0.0130
Tlr3	0.0000	4.7623	0.0271	0.0000	3.6561	0.0376
Tlr4	0.0003	2.9827	0.0491	0.0000	4.4656	0.0355
Tm4sf1	0.0000	3.1815	0.0087	0.0000	4.2152	0.0047
Tma16	0.0000	1.2240	0.0119	0.0000	1.0816	0.0169
Tmbim1	0.0000	1.1458	0.0195	0.0000	1.8925	0.0077
Tmc6	0.0000	2.9612	0.0113	0.0000	2.8553	0.0138
Tmc8	0.0000	3.7070	0.0377	0.0000	4.0969	0.0355

Tmco4	0.0000	4.2246	0.0112	0.0000	5.1950	0.0079
Tmeff1	0.0000	-1.0122	0.0072	0.0000	-1.0127	0.0084
Tmeff2	0.0000	-1.6754	0.0155	0.0000	-2.4596	0.0082
Tmem106a	0.0000	2.7177	0.0250	0.0000	2.1374	0.0349
Tmem132e	0.0000	-3.5201	0.0040	0.0000	-2.8055	0.0070
Tmem141	0.0000	-1.4210	0.0418	0.0002	-1.1279	0.0495
Tmem144	0.0000	2.1494	0.0298	0.0000	2.9008	0.0208
Tmem14a	0.0000	1.2884	0.0280	0.0000	1.2993	0.0289
Tmem151b	0.0000	-1.2324	0.0126	0.0000	-2.0714	0.0044
Tmem154	0.0000	2.7342	0.0428	0.0000	4.1324	0.0290
Tmem169	0.0000	-1.5037	0.0137	0.0000	-2.3720	0.0059
Tmem170b	0.0000	-1.5521	0.0072	0.0000	-1.5080	0.0086
Tmem178b	0.0000	-1.1530	0.0188	0.0000	-1.8616	0.0082
Tmem192	0.0000	1.2710	0.0161	0.0000	2.0502	0.0061
Tmem198	0.0000	-1.7272	0.0166	0.0000	-1.9649	0.0143
Tmem229b	0.0000	-1.5676	0.0097	0.0000	-1.3872	0.0136
Tmem25	0.0000	-1.7075	0.0387	0.0000	-1.5275	0.0428
Tmem266	0.0000	-1.4182	0.0365	0.0000	-1.2221	0.0421
Tmem30a	0.0000	-1.0601	0.0090	0.0000	-1.0782	0.0098
Tmem37	0.0000	2.1686	0.0211	0.0000	2.0628	0.0241
Tmem44	0.0000	-2.7231	0.0025	0.0000	-2.5894	0.0031
Tmem51	0.0000	1.1728	0.0397	0.0000	2.8322	0.0115
Tmem59l	0.0000	1.7063	0.0319	0.0000	1.3863	0.0407
Tmem63a	0.0000	1.2667	0.0345	0.0000	1.2936	0.0351
Tmem63c	0.0004	-1.8923	0.0500	0.0001	-2.0936	0.0471
Tmem65	0.0000	-1.0361	0.0119	0.0000	-1.0776	0.0123
Tmem74b	0.0000	-1.3668	0.0431	0.0000	-1.4877	0.0411
Tmem88	0.0000	1.7210	0.0349	0.0000	2.0582	0.0297
Tmem8b	0.0000	-1.2292	0.0100	0.0000	-1.0980	0.0139
Tmod2	0.0000	-2.1753	0.0024	0.0000	-1.7485	0.0053
Tmtc2	0.0000	-1.4774	0.0098	0.0000	-2.3916	0.0034
Tmtc4	0.0000	-1.2312	0.0122	0.0000	-1.0558	0.0180
Tnfrsf12a	0.0000	1.5209	0.0205	0.0000	1.2304	0.0290
Tnfrsf13b	0.0001	4.6180	0.0468	0.0000	5.5151	0.0418
Tnfrsf1b	0.0000	2.3968	0.0245	0.0000	5.0051	0.0058
Tnip1	0.0000	1.2513	0.0128	0.0000	1.9950	0.0046
Tnnt1	0.0000	1.8145	0.0308	0.0000	2.2274	0.0251
Tnr	0.0000	-1.1520	0.0404	0.0000	-2.1700	0.0216
Tomm20	0.0000	1.0436	0.0049	0.0000	1.0585	0.0057
Tor3a	0.0000	2.4330	0.0087	0.0000	2.5052	0.0092
Tor4a	0.0000	1.3775	0.0370	0.0000	1.6536	0.0318
Tox	0.0000	-1.2393	0.0200	0.0000	-2.0582	0.0087
Tox2	0.0000	-3.4174	0.0021	0.0000	-2.0743	0.0072
Tpd52	0.0000	1.0020	0.0112	0.0000	1.6261	0.0038
Tpd52l1	0.0000	1.9634	0.0093	0.0000	1.7940	0.0128
Tpmt	0.0000	-1.1861	0.0215	0.0000	-1.6965	0.0129
Tppp	0.0000	-1.0276	0.0401	0.0000	-1.7487	0.0241
Traf3ip2	0.0000	1.2605	0.0332	0.0000	2.1627	0.0165
Trafd1	0.0000	-1.1952	0.0122	0.0000	-1.3520	0.0106
Traip	0.0000	1.1601	0.0203	0.0000	1.2271	0.0200
Tram1	0.0000	1.3536	0.0055	0.0000	1.2391	0.0081
Trap1	0.0000	1.0942	0.0090	0.0000	1.1964	0.0083
Trhde	0.0000	-2.3930	0.0304	0.0000	-5.3392	0.0185
Trib3	0.0000	2.8893	0.0044	0.0000	4.2817	0.0013
Trim25	0.0000	2.6051	0.0027	0.0000	3.0917	0.0018
Trim36	0.0000	-1.6110	0.0086	0.0000	-3.1769	0.0018
Trim46	0.0000	-1.2516	0.0214	0.0000	-2.3230	0.0078
Trim47	0.0000	1.4255	0.0385	0.0000	3.3563	0.0113
Trim52	0.0000	5.5141	0.0272	0.0000	5.9440	0.0261
Trim6	0.0000	3.0952	0.0011	0.0000	3.6735	0.0004
Trim62	0.0000	-2.7187	0.0042	0.0000	-1.6797	0.0127
Trim66	0.0000	5.7376	0.0010	0.0000	3.9842	0.0039
Trim67	0.0000	-1.2851	0.0114	0.0000	-2.1104	0.0039
Trim71	0.0000	2.0622	0.0247	0.0000	2.1805	0.0247
Trim9	0.0000	-1.2608	0.0118	0.0000	-2.4872	0.0026
Triml2	0.0000	4.1175	0.0020	0.0000	5.6311	0.0006
Trio	0.0000	-1.1338	0.0096	0.0000	-2.0837	0.0021
Trip10	0.0000	1.6423	0.0071	0.0000	1.2734	0.0144
Trip13	0.0000	1.6346	0.0067	0.0000	1.4135	0.0111
Tro	0.0000	-1.0290	0.0096	0.0000	-1.4701	0.0046
Troap	0.0000	1.1889	0.0240	0.0000	1.2706	0.0233
Trpm2	0.0000	4.3949	0.0104	0.0000	2.5927	0.0285
Trpm3	0.0000	4.7998	0.0016	0.0000	1.8648	0.0206
Tsen15	0.0000	1.7772	0.0127	0.0000	1.4205	0.0209
Tshz1	0.0000	-3.0606	0.0015	0.0000	-3.1451	0.0013
Tshz2	0.0000	-4.1201	0.0066	0.0000	-2.5168	0.0198
Tshz3	0.0000	-3.0511	0.0029	0.0000	-2.4508	0.0054

Tspan11	0.0000	-2.7172	0.0070	0.0000	-1.7350	0.0170
Tspan17	0.0000	-1.7101	0.0159	0.0000	-1.3728	0.0231
Tspo	0.0000	2.6999	0.0149	0.0000	3.7695	0.0079
Tspyl4	0.0000	-1.4161	0.0044	0.0000	-1.9834	0.0019
Tst	0.0000	1.5767	0.0255	0.0000	1.4178	0.0304
Ttbk2	0.0000	-1.5401	0.0044	0.0000	-1.8254	0.0032
Ttc28	0.0000	-1.1850	0.0096	0.0000	-1.8136	0.0038
Ttc39b	0.0000	2.4045	0.0037	0.0000	2.9448	0.0022
Ttc9b	0.0000	-1.6776	0.0228	0.0000	-2.2202	0.0162
Ttll6	0.0000	2.6726	0.0388	0.0000	4.7799	0.0197
Ttll7	0.0000	-1.4742	0.0286	0.0000	-2.3765	0.0161
Ttn	0.0000	1.4902	0.0411	0.0000	1.6138	0.0395
Ttpa	0.0000	3.6197	0.0100	0.0000	4.6404	0.0061
Ttpal	0.0000	-1.4402	0.0126	0.0000	-1.2552	0.0175
Ttyh1	0.0000	-2.0057	0.0019	0.0000	-1.1558	0.0096
Tub	0.0000	-1.9674	0.0031	0.0000	-2.1643	0.0027
Tuba1a	0.0000	-1.4898	0.0021	0.0000	-2.4323	0.0002
Tuba4a	0.0000	3.6436	0.0017	0.0000	4.2395	0.0009
Tubb2b	0.0000	-1.5961	0.0013	0.0000	-1.9297	0.0006
Tulp2	0.0000	4.1625	0.0203	0.0000	4.5462	0.0191
Tulp4	0.0000	-1.3299	0.0056	0.0000	-2.0595	0.0018
Tyms	0.0000	1.8804	0.0038	0.0000	1.3029	0.0112
Uap1l1	0.0000	1.6740	0.0064	0.0000	3.1126	0.0009
Uba52	0.0000	4.1900	0.0003	0.0000	1.2314	0.0095
Ubap1l	0.0000	2.5489	0.0371	0.0000	5.0770	0.0150
Ubash3b	0.0000	-2.2488	0.0125	0.0000	-1.0208	0.0365
Ube2c	0.0000	1.4584	0.0073	0.0000	1.3839	0.0096
Ube2ql1	0.0000	-1.3939	0.0153	0.0000	-2.7717	0.0036
Ube2t	0.0000	1.2203	0.0229	0.0000	2.5540	0.0053
Ubn2	0.0000	-1.0320	0.0100	0.0000	-1.6679	0.0034
Ubxn10	0.0000	3.3512	0.0135	0.0000	3.0731	0.0176
Ubxn8	0.0000	1.1179	0.0235	0.0000	1.0121	0.0282
Ucp2	0.0000	1.4769	0.0050	0.0000	2.2916	0.0013
Uhrf1	0.0000	1.7599	0.0054	0.0000	1.6554	0.0075
Ulk2	0.0000	-1.0797	0.0129	0.0000	-1.7481	0.0048
Umad1	0.0000	-1.1617	0.0237	0.0000	-1.4910	0.0176
Umps	0.0000	1.4623	0.0080	0.0000	1.3374	0.0111
Unc13d	0.0000	3.4752	0.0187	0.0000	2.6635	0.0293
Unc5a	0.0000	-2.5573	0.0092	0.0000	-2.5914	0.0095
Unc5d	0.0000	-1.7760	0.0177	0.0000	-2.3786	0.0116
Unc79	0.0000	-1.2307	0.0246	0.0000	-3.1214	0.0053
Ung	0.0000	2.3300	0.0021	0.0000	2.8287	0.0011
Upp1	0.0000	3.7472	0.0006	0.0000	3.6322	0.0005
Usp26	0.0000	3.9122	0.0282	0.0000	2.9123	0.0402
Usp43	0.0000	1.5927	0.0422	0.0000	1.9976	0.0351
Usp9x	0.0000	1.6804	0.0013	0.0000	1.1434	0.0051
Usp9y	0.0000	4.3733	0.0397	0.0000	4.0163	0.0441
Utp4	0.0000	1.8274	0.0028	0.0000	1.5881	0.0052
Uxs1	0.0000	1.5754	0.0085	0.0000	1.2981	0.0144
Vamp8	0.0000	1.6355	0.0189	0.0000	1.7687	0.0181
Vash1	0.0000	-1.3267	0.0108	0.0000	-2.0744	0.0043
Vash2	0.0000	-2.0560	0.0038	0.0000	-2.3926	0.0028
Vav3	0.0000	-1.7125	0.0114	0.0000	-3.1850	0.0034
Vcan	0.0000	-1.9919	0.0011	0.0000	-1.3458	0.0042
Vegfc	0.0000	1.7242	0.0251	0.0000	1.9159	0.0230
Vezt	0.0000	-1.5286	0.0066	0.0000	-1.2678	0.0113
Vgf	0.0000	1.5390	0.0280	0.0000	2.4961	0.0137
Vil1	0.0000	3.2448	0.0402	0.0000	3.9656	0.0342
Vill	0.0000	1.0998	0.0397	0.0000	1.7744	0.0241
Vit	0.0000	-3.8927	0.0062	0.0000	-2.0055	0.0196
Vopp1	0.0000	-1.7568	0.0022	0.0000	-1.7977	0.0023
Vps37d	0.0000	-1.6345	0.0119	0.0000	-1.6373	0.0130
Vrk2	0.0000	1.1577	0.0316	0.0000	1.1462	0.0332
Vsnl1	0.0000	-3.0024	0.0156	0.0000	-1.2600	0.0399
Vstm2a	0.0000	-2.0447	0.0239	0.0000	-1.9001	0.0270
Vstm2b	0.0000	-1.4875	0.0244	0.0000	-1.7379	0.0209
Vstm2l	0.0000	-1.7339	0.0116	0.0000	-1.5810	0.0150
Vsx1	0.0000	-4.2339	0.0366	0.0000	-4.7104	0.0346
Vwa3a	0.0000	2.5031	0.0334	0.0000	2.2480	0.0386
Vwa3b	0.0000	4.6786	0.0052	0.0000	5.0978	0.0048
Vwf	0.0000	2.8703	0.0239	0.0000	3.7575	0.0168
Was	0.0000	3.6925	0.0350	0.0000	3.3747	0.0396
Wbp1	0.0000	-1.3493	0.0068	0.0000	-1.1433	0.0112
Wbp2nl	0.0000	4.1719	0.0416	0.0000	4.2233	0.0425
Wdr12	0.0000	1.3654	0.0075	0.0000	1.3009	0.0096
Wdr31	0.0000	2.8025	0.0058	0.0000	2.0703	0.0138
Wdr47	0.0000	-1.2357	0.0086	0.0000	-2.0586	0.0025

Wdr6	0.0000	-1.1082	0.0046	0.0000	-1.3814	0.0028
Wdr62	0.0000	1.4911	0.0152	0.0000	1.3862	0.0188
Wdr63	0.0000	3.6697	0.0263	0.0000	2.5987	0.0399
Wdr75	0.0000	1.4116	0.0055	0.0000	1.3127	0.0079
Wdr76	0.0000	1.1583	0.0203	0.0000	1.4913	0.0144
Wdr86	0.0000	1.2093	0.0398	0.0000	1.1253	0.0436
Wee1	0.0000	1.2334	0.0111	0.0000	1.1353	0.0149
Wfdc2	0.0000	4.0973	0.0083	0.0000	5.6308	0.0040
Wnk2	0.0000	-1.2754	0.0076	0.0000	-1.8157	0.0035
Wnt10b	0.0000	5.0116	0.0167	0.0000	2.7191	0.0395
Wnt11	0.0000	-2.3308	0.0171	0.0000	-1.3198	0.0350
Wnt4	0.0000	-2.7368	0.0072	0.0000	-2.7440	0.0078
Wnt5a	0.0000	-2.1326	0.0039	0.0000	-1.1215	0.0178
Wnt7a	0.0000	-4.5783	0.0023	0.0000	-2.9466	0.0049
Wnt7b	0.0000	-2.2317	0.0269	0.0000	-2.2016	0.0286
Wscd2	0.0000	-2.5236	0.0044	0.0000	-1.9258	0.0089
Wtip	0.0000	1.2423	0.0212	0.0000	1.2415	0.0226
Xaf1	0.0000	3.4164	0.0086	0.0000	3.9536	0.0068
Xk	0.0000	1.5375	0.0254	0.0000	2.0001	0.0181
Xkr4	0.0000	-1.0677	0.0284	0.0000	-2.2975	0.0090
Xkr7	0.0000	-1.2091	0.0318	0.0000	-2.7968	0.0106
Xrcc5	0.0000	2.2471	0.0009	0.0000	2.5817	0.0004
Xylb	0.0000	1.3671	0.0309	0.0000	1.3243	0.0333
Ybx3	0.0000	1.3840	0.0036	0.0000	1.1826	0.0066
Yif1b	0.0000	1.0585	0.0151	0.0000	1.2884	0.0112
Ypel4	0.0000	-2.3537	0.0170	0.0000	-2.5336	0.0160
Zap70	0.0000	4.9451	0.0229	0.0000	5.5298	0.0209
Zbtb16	0.0000	-1.9758	0.0171	0.0000	-2.3758	0.0135
Zbtb32	0.0000	4.7906	0.0217	0.0000	4.0195	0.0293
Zbtb44	0.0000	1.0773	0.0116	0.0000	1.5917	0.0051
Zbtb46	0.0000	-1.9270	0.0091	0.0000	-1.6563	0.0133
Zc3h12a	0.0001	1.4209	0.0469	0.0000	2.0910	0.0338
Zc3h12b	0.0000	-1.3276	0.0451	0.0001	-1.2156	0.0482
Zc3h8	0.0000	1.0596	0.0377	0.0000	1.3319	0.0308
Zc3hav1	0.0000	2.5712	0.0024	0.0000	2.9540	0.0017
Zc3hav1l	0.0000	-1.0347	0.0150	0.0000	-1.2976	0.0107
Zc4h2	0.0000	-1.7879	0.0050	0.0000	-2.2336	0.0031
Zcchc10	0.0000	1.1474	0.0242	0.0000	1.2891	0.0219
Zcwpw1	0.0000	1.0999	0.0279	0.0000	1.3575	0.0220
Zdhhc14	0.0000	-1.7548	0.0327	0.0001	-1.1286	0.0475
Zeb1	0.0000	-1.6312	0.0030	0.0000	-1.0067	0.0115
Zfhx3	0.0000	-1.6270	0.0043	0.0000	-1.9624	0.0029
Zfp14	0.0000	-1.5825	0.0240	0.0000	-1.5472	0.0257
Zfp41	0.0000	-1.3939	0.0056	0.0000	-2.1483	0.0018
Zfpm2	0.0001	-1.1370	0.0470	0.0000	-1.4505	0.0397
Zfr2	0.0000	-1.5789	0.0176	0.0000	-1.0533	0.0311
Zhx2	0.0000	-1.4637	0.0132	0.0000	-1.1593	0.0205
Zic3	0.0000	3.1720	0.0006	0.0000	2.6276	0.0009
Zic5	0.0000	2.7603	0.0017	0.0000	2.3477	0.0033
Zmat4	0.0000	1.3363	0.0218	0.0000	1.5771	0.0180
Zmiz1	0.0000	-1.5357	0.0035	0.0000	-1.9784	0.0019
Zmym6	0.0000	-1.1308	0.0129	0.0000	-1.6277	0.0066
Zmynd10	0.0000	1.8986	0.0407	0.0000	1.8070	0.0437
Zranb3	0.0000	1.5107	0.0080	0.0000	1.2080	0.0147
Zswim5	0.0000	-1.6411	0.0060	0.0000	-2.6366	0.0019
Zswim7	0.0000	1.1673	0.0322	0.0000	1.8152	0.0187
Zwilch	0.0000	1.6279	0.0062	0.0000	1.7091	0.0065

Genes of category 4

	Untreated Tet/Tet Neurons			Doxycycline-treated *Tet/Tet* Neurons		
Gene	*p* -value	Log_2 Fold change	Q-value (Benjamini-Hochberg)	*p* -value	Log_2 Fold change	Q-value (Benjamini-Hochberg)
Aak1	0.0000	-0.7644	0.0186	0.0000	-1.2296	0.0081
Abcd2	0.0001	-0.6976	0.0465	0.0000	-1.7776	0.0177
Abcg1	0.0000	0.8874	0.0349	0.0000	1.0789	0.0293
Abcg4	0.0582	-0.5239	0.0677	0.0000	-1.3275	0.0416
Abhd14a	0.0001	-0.7040	0.0466	0.0000	1.0984	0.0295
Ablim2	0.2305	-0.3297	0.0778	0.0000	1.3529	0.0371
Ablim3	0.0000	-0.8912	0.0425	0.0000	-1.1415	0.0352
Acad10	0.0002	0.7119	0.0484	0.0000	1.6500	0.0202
Acad11	0.0000	0.9993	0.0237	0.0000	1.0373	0.0240
Acadl	0.0000	0.9023	0.0201	0.0000	1.1236	0.0151
Accs	0.0001	-0.9436	0.0477	0.0000	-1.5345	0.0326
Acot13	0.0000	0.6856	0.0363	0.0000	1.3616	0.0151
Acot2	0.2786	0.1720	0.0795	0.0000	1.0422	0.0286
Acsl4	0.0000	-0.6942	0.0268	0.0000	-1.1203	0.0136
Acss1	0.1107	0.2059	0.0718	0.0000	1.3548	0.0148

Acvr2b	0.0000	-0.6841	0.0307	0.0000	-1.2675	0.0135
Adamts15	0.0443	-0.2864	0.0662	0.0000	-1.1464	0.0237
Adamts4	0.2415	0.2679	0.0782	0.0000	2.5640	0.0112
Adamtsl4	0.6254	-0.2651	0.0901	0.0000	2.0251	0.0454
Adcy3	0.0013	0.5992	0.0532	0.0000	1.7153	0.0178
Adgra1	0.0000	-0.9310	0.0396	0.0000	-1.5696	0.0238
Adgrb3	0.2313	-0.1748	0.0778	0.0000	-1.3948	0.0192
Adgrg1	0.0025	-0.2975	0.0551	0.0000	-1.5567	0.0065
Adgrl2	0.0000	-0.8386	0.0172	0.0000	-1.0394	0.0128
Adi1	0.1872	-0.1515	0.0761	0.0000	1.7129	0.0061
Adnp	0.0006	-0.3645	0.0513	0.0000	-1.4035	0.0099
Adpgk	0.0005	0.3418	0.0503	0.0000	1.0465	0.0141
Aebp1	0.0000	0.8868	0.0247	0.0000	1.5505	0.0100
Aff3	0.0000	-0.7661	0.0276	0.0000	-1.1379	0.0168
Ago1	0.0000	-0.7728	0.0173	0.0000	-1.7382	0.0028
Agtpbp1	0.0000	-0.9986	0.0208	0.0000	-1.2054	0.0166
Agtrap	0.0001	0.4047	0.0465	0.0000	2.0157	0.0030
Aif1l	0.0000	-0.8427	0.0302	0.0000	-1.1026	0.0227
Akap11	0.0000	-0.7983	0.0153	0.0000	-1.3257	0.0057
Akap14	0.0862	0.8577	0.0700	0.0000	2.5278	0.0367
Akap9	0.0000	-0.6315	0.0219	0.0000	-1.9042	0.0018
Akt3	0.0000	-0.9429	0.0113	0.0000	-1.5197	0.0040
Aldh1b1	0.0798	0.2686	0.0695	0.0000	1.9528	0.0090
Aldh9a1	0.0000	0.9215	0.0128	0.0000	1.1328	0.0093
Aldoa	0.0000	0.9613	0.0055	0.0000	1.4292	0.0019
Alg2	0.0000	-0.3477	0.0444	0.0000	-1.0113	0.0118
Alkbh8	0.0000	-0.5975	0.0278	0.0000	-1.0728	0.0119
Amdhd2	0.0179	0.3561	0.0620	0.0000	1.5860	0.0139
Amer2	0.0018	-0.3184	0.0540	0.0000	-1.4298	0.0088
Amn1	0.0000	-0.7633	0.0376	0.0000	-1.2046	0.0239
Amotl2	0.0000	-0.9024	0.0116	0.0000	-1.0238	0.0100
Amt	0.0000	0.9147	0.0185	0.0000	1.4865	0.0077
Angptl2	0.0000	-0.6502	0.0450	0.0000	-1.5133	0.0185
Ankrd37	0.5389	-0.1462	0.0876	0.0000	-1.0385	0.0448
Ankrd42	0.0069	-0.5391	0.0585	0.0000	-1.5510	0.0255
Anks6	0.3196	-0.2021	0.0809	0.0000	-1.1379	0.0363
Ano10	0.0000	-0.8509	0.0219	0.0000	-1.2693	0.0121
Ap3b2	0.0000	-0.5423	0.0329	0.0000	-1.4570	0.0069
Ap3s2	0.0000	-0.8832	0.0118	0.0000	-1.0201	0.0098
Ap4s1	0.0002	-0.7174	0.0479	0.0000	-1.0599	0.0355
Apc	0.0000	-0.7079	0.0160	0.0000	-1.4044	0.0037
Aplp1	0.0000	-0.7898	0.0122	0.0000	-1.3805	0.0035
Apod	0.0000	-0.6086	0.0450	0.0000	2.8361	0.0022
Aptx	0.0000	-0.6554	0.0314	0.0000	-1.1262	0.0160
Arel1	0.0000	-0.7118	0.0231	0.0000	-1.0815	0.0126
Arhgap22	0.4540	0.2474	0.0852	0.0000	1.5616	0.0395
Arhgap35	0.0000	-0.5879	0.0210	0.0000	-1.0193	0.0081
Arhgap8	0.7257	0.1663	0.0928	0.0001	1.7664	0.0471
Arhgef19	0.4695	0.2254	0.0857	0.0000	1.4136	0.0412
Arhgef26	0.0012	-0.4742	0.0529	0.0000	-1.5073	0.0167
Arid1b	0.0000	-0.7980	0.0167	0.0000	-1.0301	0.0113
Arid2	0.0000	-0.4469	0.0334	0.0000	-1.0082	0.0104
Arid4b	0.0000	-0.5087	0.0313	0.0000	-1.2082	0.0082
Arid5b	0.0000	0.7588	0.0234	0.0000	1.0860	0.0142
Arl10	0.0053	-0.3973	0.0576	0.0000	-1.1748	0.0231
Arl16	0.0000	-0.8648	0.0401	0.0000	-1.3405	0.0267
Arl5c	0.4106	-0.3458	0.0839	0.0000	2.0143	0.0366
Armcx3	0.0000	-0.4229	0.0390	0.0000	-1.0842	0.0112
Asah1	0.0000	0.5279	0.0308	0.0000	1.4575	0.0050
Ascl1	0.0000	-0.4918	0.0405	0.0000	-1.4982	0.0086
Ash1l	0.0000	-0.4556	0.0382	0.0000	-1.0077	0.0144
Asl	0.0000	0.9983	0.0263	0.0000	1.0499	0.0257
Atf4	0.0000	0.5237	0.0273	0.0000	1.0527	0.0088
Atp1b1	0.0000	0.8247	0.0163	0.0000	1.3764	0.0060
Atp7a	0.0043	-0.3109	0.0568	0.0000	-1.0287	0.0187
Atrn	0.0000	-0.4742	0.0367	0.0000	-1.2194	0.0097
Atrnl1	0.0000	-0.9362	0.0156	0.0000	-1.6327	0.0051
Atxn7l3b	0.0000	-0.7137	0.0126	0.0000	-1.2555	0.0036
Aunip	0.2000	0.2943	0.0767	0.0000	2.5836	0.0111
Aurkb	0.0000	0.5709	0.0414	0.0000	1.0613	0.0217
B2m	0.0000	0.5257	0.0432	0.0000	1.7387	0.0078
B3galt1	0.0000	-0.9961	0.0350	0.0000	-1.5385	0.0221
Bag3	0.0000	0.7794	0.0314	0.0000	1.2341	0.0177
Bag4	0.0000	0.8651	0.0177	0.0000	1.0998	0.0125
Bbs1	0.0042	-0.4052	0.0568	0.0000	-1.0352	0.0267
Bcl9	0.0000	-0.7667	0.0185	0.0000	-1.3339	0.0067
Bcr	0.0000	-0.6140	0.0305	0.0000	-1.0815	0.0144

Bhlhe40	0.0951	0.1547	0.0708	0.0000	1.4187	0.0063
Bhlhe41	0.0055	-0.4917	0.0577	0.0000	1.2327	0.0257
Bid	0.0000	0.9392	0.0264	0.0000	1.3541	0.0160
Birc3	0.3894	0.2732	0.0832	0.0000	1.7402	0.0340
Birc5	0.0000	0.9625	0.0209	0.0000	1.1086	0.0180
Blvrb	0.0001	0.5818	0.0475	0.0000	1.4978	0.0162
Bmpr1b	0.6403	0.0769	0.0904	0.0000	-1.2526	0.0264
Bzw2	0.0000	-0.6693	0.0182	0.0000	-1.1097	0.0073
C1qtnf1	0.1738	-0.7087	0.0754	0.0000	1.9953	0.0433
C1qtnf6	0.0000	0.6890	0.0398	0.0000	1.5803	0.0136
C2cd2l	0.0000	-0.8258	0.0371	0.0000	-1.5908	0.0178
C3	0.1126	0.9680	0.0719	0.0000	6.2696	0.0109
Cables2	0.0000	-0.9119	0.0257	0.0000	-1.0490	0.0222
Cacna1b	0.0000	-0.8514	0.0257	0.0000	-2.4130	0.0036
Cacna1d	0.3918	-0.2006	0.0833	0.0000	-1.4918	0.0326
Cacna1e	0.0896	-0.3366	0.0702	0.0000	-1.6995	0.0228
Cacnb3	0.0022	-0.2731	0.0547	0.0000	-1.0034	0.0138
Cacng7	0.0000	-0.5859	0.0299	0.0000	-1.0472	0.0136
Cadm1	0.0000	-0.5262	0.0295	0.0000	-1.4804	0.0045
Camk1	0.0964	-0.2889	0.0708	0.0000	1.3775	0.0213
Camk1g	0.0000	-0.8147	0.0437	0.0000	-1.6156	0.0225
Camk2g	0.0000	-0.8150	0.0195	0.0000	-1.0443	0.0139
Camsap2	0.0000	-0.6375	0.0204	0.0000	-1.1995	0.0064
Card6	0.0250	0.7173	0.0636	0.0000	1.2838	0.0458
Carhsp1	0.0000	-0.9712	0.0089	0.0000	-1.6068	0.0027
Cartpt	0.3897	-0.2016	0.0832	0.0000	-2.3048	0.0193
Casd1	0.0002	-0.3939	0.0483	0.0000	-1.2119	0.0136
Caskin1	0.0000	-0.9568	0.0267	0.0000	-1.5641	0.0135
Casq1	0.1079	0.9744	0.0716	0.0000	2.4563	0.0447
Cavin2	0.0230	0.4875	0.0632	0.0000	1.7424	0.0217
Cblb	0.0042	-0.2960	0.0567	0.0000	-1.6485	0.0065
Cbln1	0.0001	0.3870	0.0470	0.0000	-1.7804	0.0050
Ccar2	0.0000	-0.6069	0.0231	0.0000	-1.3640	0.0050
Ccdc102a	0.0000	0.9491	0.0324	0.0000	1.2049	0.0256
Ccdc14	0.0000	0.9607	0.0272	0.0000	1.0318	0.0260
Ccdc160	0.0087	0.4899	0.0592	0.0000	1.3545	0.0254
Ccdc40	0.0003	-0.6286	0.0492	0.0000	-1.0299	0.0335
Ccdc43	0.0000	0.7145	0.0257	0.0000	1.0648	0.0146
Ccdc50	0.0000	-0.7356	0.0174	0.0000	-1.3799	0.0049
Ccdc80	0.5654	-0.2464	0.0883	0.0000	2.0659	0.0404
Ccdc82	0.0009	-0.5107	0.0522	0.0000	-1.1109	0.0271
Ccdc85c	0.0000	-0.9619	0.0115	0.0000	-1.2496	0.0074
Ccdc88a	0.0000	-0.4981	0.0315	0.0000	-1.1146	0.0094
Ccnd3	0.0000	0.6255	0.0230	0.0000	1.1562	0.0079
Cd34	0.0945	-0.4926	0.0707	0.0000	2.0183	0.0246
Cd99l2	0.0000	-0.7614	0.0193	0.0000	-1.0382	0.0121
Cdc25b	0.0000	-0.7913	0.0230	0.0000	-1.1490	0.0136
Cdh2	0.0000	-0.4618	0.0279	0.0000	-2.1915	0.0005
Cdh22	0.0000	-0.9541	0.0281	0.0000	-1.8172	0.0110
Cdh4	0.0000	-0.8214	0.0214	0.0000	-1.9955	0.0036
Cdk14	0.0000	-0.8893	0.0178	0.0000	-1.2262	0.0107
Cdkn3	0.0001	0.9134	0.0474	0.0000	1.3358	0.0348
Cdyl	0.0000	0.9142	0.0173	0.0000	1.1060	0.0133
Cebpd	0.0113	-0.6281	0.0601	0.0000	2.4085	0.0140
Celf2	0.0000	-0.9241	0.0152	0.0000	-1.6066	0.0050
Cep55	0.0000	0.9568	0.0193	0.0000	1.1826	0.0145
Cers4	0.0000	0.6012	0.0362	0.0000	1.1781	0.0155
Cers6	0.0000	-0.9428	0.0141	0.0000	-1.2698	0.0086
Chchd4	0.0000	0.8334	0.0224	0.0000	1.2140	0.0127
Chd7	0.0000	-0.6101	0.0314	0.0000	-1.5127	0.0075
Chmp3	0.0000	0.9647	0.0143	0.0000	1.0624	0.0132
Chrdl1	0.0003	0.8542	0.0490	0.0000	-1.1603	0.0418
Cib2	0.0025	-0.6809	0.0551	0.0000	-1.3482	0.0336
Clasp1	0.0097	-0.1843	0.0596	0.0000	-1.1020	0.0068
Clasp2	0.0000	-0.6512	0.0223	0.0000	-1.2183	0.0076
Clcn4	0.0000	-0.8941	0.0132	0.0000	-1.7856	0.0027
Clec11a	0.8043	0.0792	0.0948	0.0000	2.5391	0.0202
Clgn	0.0274	0.4281	0.0640	0.0000	1.3863	0.0259
Clip1	0.0000	-0.6254	0.0272	0.0000	-1.0338	0.0134
Clstn3	0.0000	-0.5357	0.0401	0.0000	-1.0463	0.0194
Clvs1	0.0000	-0.7074	0.0361	0.0000	-2.2169	0.0063
Cmklr1	0.0036	-0.7404	0.0563	0.0000	1.0373	0.0433
Cmpk2	0.1448	-0.3637	0.0738	0.0000	-1.2306	0.0406
Cnot6	0.0368	-0.1670	0.0654	0.0000	-1.1892	0.0075
Cnpy3	0.5647	0.0584	0.0883	0.0000	1.1073	0.0134
Cntn2	0.0000	0.4247	0.0355	0.0000	-1.8763	0.0020
Col8a2	0.8852	0.0474	0.0968	0.0000	1.5172	0.0421

Col9a2	0.0043	-0.5992	0.0569	0.0000	1.5766	0.0233
Colgalt1	0.0000	0.4282	0.0338	0.0000	1.1593	0.0067
Coro1a	0.0000	-0.9431	0.0365	0.0000	-1.9920	0.0153
Cox17	0.0000	0.5480	0.0407	0.0000	1.1968	0.0160
Cpeb2	0.0000	-0.6190	0.0336	0.0000	-1.1871	0.0148
Cramp1l	0.0000	-0.6710	0.0311	0.0000	-1.1237	0.0165
Crb2	0.1495	-0.1768	0.0740	0.0000	-1.7491	0.0088
Crebbp	0.6133	-0.0550	0.0897	0.0000	-1.0590	0.0180
Creg1	0.3848	-0.1381	0.0830	0.0000	1.4347	0.0184
Crhbp	0.0894	0.5056	0.0702	0.0002	-1.1595	0.0499
Crispld1	0.0000	-0.9381	0.0362	0.0000	3.1152	0.0026
Crybg3	0.0000	-0.9195	0.0248	0.0000	-1.4321	0.0134
Csf1	0.6363	0.0593	0.0903	0.0000	1.1553	0.0182
Csmd2	0.0000	-0.9141	0.0346	0.0000	-2.8811	0.0065
Csrnp2	0.0000	-0.7944	0.0240	0.0000	-1.5109	0.0085
Csrp2	0.0000	0.7238	0.0207	0.0000	1.6063	0.0038
Cstb	0.0000	0.8025	0.0246	0.0000	1.2578	0.0126
Ctbp1	0.0000	-0.6202	0.0203	0.0000	-1.0941	0.0074
Ctbs	0.0003	0.5800	0.0489	0.0000	1.3695	0.0204
Ctnna2	0.9866	-0.0016	0.0996	0.0000	-2.7321	0.0012
Ctnnd2	0.0000	-0.5674	0.0358	0.0000	-1.5477	0.0082
Ctsa	0.0000	0.6248	0.0262	0.0000	1.4556	0.0053
Ctsz	0.0234	0.2565	0.0633	0.0000	1.5807	0.0078
Cux1	0.0000	-0.5496	0.0285	0.0000	-1.2296	0.0078
Cxadr	0.0000	-0.4949	0.0320	0.0000	-1.4403	0.0051
Cxcl1	0.7464	0.3398	0.0934	0.0000	7.7112	0.0166
Cxcl16	0.4915	0.2302	0.0863	0.0000	1.7290	0.0358
Cyb5d1	0.2354	-0.2396	0.0780	0.0000	-1.0999	0.0371
Cybrd1	0.6072	-0.1561	0.0895	0.0000	1.7590	0.0312
Cyp2s1	0.0001	0.9382	0.0467	0.0000	1.0127	0.0449
Cyp46a1	0.1054	0.3617	0.0714	0.0000	-1.4884	0.0317
Cyyr1	0.8828	0.0505	0.0967	0.0000	1.7385	0.0361
Dab1	0.0000	-0.5714	0.0372	0.0000	-1.7229	0.0074
Dach1	0.0000	-0.8698	0.0238	0.0000	-2.7281	0.0021
Daglb	0.0003	-0.7027	0.0493	0.0000	-1.0444	0.0368
Dbn1	0.0000	-0.8809	0.0106	0.0000	-1.9882	0.0011
Dbp	0.0239	-0.3608	0.0634	0.0000	1.1480	0.0249
Dcaf7	0.0000	-0.7174	0.0139	0.0000	-1.1745	0.0051
Dcc	0.0000	0.6895	0.0215	0.0000	-2.5508	0.0009
Dctpp1	0.0000	0.8446	0.0264	0.0000	1.2312	0.0159
Dcun1d4	0.0000	-0.7624	0.0218	0.0000	-1.4599	0.0069
Dcxr	0.1847	0.2142	0.0760	0.0000	1.2898	0.0222
Ddah2	0.0000	-0.6610	0.0249	0.0000	-1.1128	0.0114
Ddit3	0.0000	0.9294	0.0273	0.0000	2.2349	0.0053
Ddx25	0.0000	-0.6204	0.0415	0.0000	-1.1729	0.0216
Deaf1	0.0000	-0.9161	0.0240	0.0000	-1.3871	0.0135
Degs1	0.0000	0.8085	0.0195	0.0000	1.3510	0.0077
Dennd3	0.1922	0.4479	0.0764	0.0001	1.3039	0.0473
Dhodh	0.0000	0.9260	0.0241	0.0000	1.1434	0.0189
Dhtkd1	0.0000	0.7179	0.0381	0.0000	1.8089	0.0100
Dio3	0.0998	0.5362	0.0710	0.0000	1.6916	0.0365
Dip2b	0.0000	-0.5163	0.0330	0.0000	-1.2061	0.0095
Diras2	0.0000	0.7034	0.0370	0.0000	-1.3392	0.0196
Dleu2	0.0222	0.5554	0.0630	0.0000	1.0685	0.0429
Dleu7	0.5247	0.2918	0.0872	0.0000	1.7768	0.0459
Dlg4	0.0000	-0.9656	0.0115	0.0000	-1.6991	0.0032
Dlgap1	0.0006	-0.6714	0.0509	0.0000	-1.0761	0.0358
Dll3	0.0007	-0.5437	0.0514	0.0000	-1.2785	0.0238
Dmtn	0.0000	0.7403	0.0310	0.0000	1.6224	0.0090
Dmxl2	0.0000	-0.6641	0.0280	0.0000	-1.3568	0.0092
Dnah5	0.4619	0.3554	0.0854	0.0000	-2.3401	0.0451
Dnajc5	0.0000	-0.9599	0.0094	0.0000	-1.2361	0.0059
Dnph1	0.0000	0.9193	0.0270	0.0000	1.5157	0.0128
Dock3	0.0000	-0.9490	0.0301	0.0000	-2.1842	0.0088
Dock5	0.0000	0.8697	0.0255	0.0000	1.2428	0.0155
Dock7	0.0000	-0.7737	0.0175	0.0000	-2.2543	0.0012
Dohh	0.0000	0.6733	0.0321	0.0000	1.0015	0.0203
Dok1	0.0000	0.9641	0.0399	0.0000	1.3165	0.0301
Dok4	0.0000	-0.7832	0.0350	0.0000	-1.2820	0.0204
Dpp8	0.0000	-0.5784	0.0300	0.0000	-1.4000	0.0072
Dscam	0.0074	-0.4214	0.0587	0.0000	-1.8690	0.0130
Dse	0.0000	0.6585	0.0327	0.0000	1.4877	0.0095
Dtwd1	0.0000	0.7460	0.0440	0.0000	1.1067	0.0310
Dtx4	0.0000	-0.6810	0.0279	0.0000	-2.3628	0.0021
Dusp9	0.0323	0.2563	0.0648	0.0000	2.7638	0.0016
Dvl3	0.0000	-0.6070	0.0274	0.0000	-1.4637	0.0059
Dync1i2	0.0000	-0.5462	0.0254	0.0000	-1.0368	0.0090

Dync2h1	0.1631	-0.1620	0.0748	0.0000	-1.4695	0.0114
Edil3	0.0000	-0.7618	0.0306	0.0000	-1.8608	0.0075
Efhd1	0.1025	0.5513	0.0712	0.0000	2.4982	0.0236
Egr1	0.0123	-0.9212	0.0605	0.0000	-2.0125	0.0364
Ehbp1	0.0000	-0.9640	0.0155	0.0000	-1.0420	0.0147
Eid1	0.0000	-0.8424	0.0121	0.0000	-1.1894	0.0065
Eif4e3	0.0000	-0.8513	0.0434	0.0000	-1.1403	0.0344
Eif4g3	0.0000	-0.5006	0.0282	0.0000	-1.1021	0.0078
Elk1	0.0000	0.7918	0.0225	0.0000	1.1556	0.0127
Eln	0.0000	0.6698	0.0424	0.0000	3.8346	0.0009
Elovl2	0.0000	-0.9907	0.0257	0.0000	-1.0738	0.0244
Emp2	0.0015	0.6309	0.0535	0.0000	2.5467	0.0088
Emp3	0.9652	0.0109	0.0990	0.0000	1.3651	0.0344
Enc1	0.0000	-0.4909	0.0311	0.0000	-1.4307	0.0047
Enoph1	0.0000	0.6531	0.0316	0.0000	1.0497	0.0174
Enox2	0.0017	-0.4390	0.0539	0.0000	-1.2586	0.0203
Epb41	0.0000	-0.9143	0.0160	0.0000	-1.8150	0.0037
Epc1	0.0000	-0.4937	0.0365	0.0000	-1.1569	0.0118
Epha7	0.0001	0.3798	0.0475	0.0000	-1.8716	0.0041
Ephx1	0.0000	0.7865	0.0397	0.0000	2.1429	0.0093
Eprs	0.0000	0.8609	0.0088	0.0000	1.0603	0.0062
Eps8	0.0585	0.3192	0.0677	0.0000	1.1413	0.0280
Evc	0.0000	0.8173	0.0358	0.0000	2.1384	0.0079
Exoc5	0.0000	-0.5046	0.0334	0.0000	-1.0337	0.0128
Exoc7	0.0000	-0.6079	0.0313	0.0000	-1.0544	0.0156
Exog	0.0000	-0.6884	0.0363	0.0000	-1.0554	0.0235
Eya4	0.0127	-0.6352	0.0607	0.0000	-2.0136	0.0255
Fabp5	0.1514	-0.1233	0.0741	0.0000	-1.4381	0.0057
Fabp7	0.0145	-0.2083	0.0612	0.0000	-1.5515	0.0042
Fam110a	0.0000	-0.6506	0.0408	0.0000	-1.2986	0.0195
Fam122b	0.0000	0.4811	0.0440	0.0000	1.5263	0.0087
Fam13b	0.6121	0.0647	0.0896	0.0000	-1.1138	0.0216
Fam181a	0.0000	-0.9008	0.0439	0.0000	-2.2395	0.0167
Fam181b	0.3330	0.0906	0.0814	0.0000	-1.6431	0.0053
Fam189b	0.0000	-0.7376	0.0421	0.0000	-1.0318	0.0319
Fam214a	0.0020	-0.4062	0.0544	0.0000	-1.2416	0.0190
Fam214b	0.0000	-0.6867	0.0303	0.0000	-1.1231	0.0164
Farp2	0.2043	0.3394	0.0769	0.0000	-1.1892	0.0450
Fas	0.7781	-0.1030	0.0942	0.0000	1.4584	0.0446
Fbf1	0.0000	-0.8099	0.0223	0.0000	-1.0127	0.0170
Fbxl17	0.0000	-0.5598	0.0412	0.0000	-1.0236	0.0223
Fbxo16	0.0215	0.3942	0.0629	0.0000	-1.1407	0.0313
Fbxo17	0.0766	0.7260	0.0692	0.0002	1.4686	0.0494
Fbxo2	0.0001	-0.8840	0.0473	0.0000	1.1469	0.0357
Fbxo6	0.0067	0.4310	0.0584	0.0000	1.6178	0.0151
Fermt3	0.0941	0.7347	0.0706	0.0000	1.7528	0.0456
Fgd4	0.0164	-0.2769	0.0617	0.0000	-1.1375	0.0176
Fgf14	0.0044	-0.7177	0.0569	0.0000	-1.3291	0.0377
Fgf18	0.0545	-0.7527	0.0673	0.0000	-1.7574	0.0442
Fgfr4	0.4230	0.3051	0.0843	0.0000	1.6458	0.0427
Fhdc1	0.0290	-0.4721	0.0643	0.0000	-1.4594	0.0299
Fkbp4	0.0000	0.8169	0.0149	0.0000	1.0298	0.0105
Fkbp9	0.5846	0.0457	0.0889	0.0000	1.3258	0.0060
Flt1	0.0060	-0.6950	0.0579	0.0000	-1.4593	0.0348
Flvcr2	0.0001	0.9832	0.0457	0.0000	1.3999	0.0339
Fmnl1	0.0792	0.5614	0.0694	0.0000	2.3251	0.0245
Fmnl2	0.0000	-0.7223	0.0231	0.0000	-1.1510	0.0114
Fnbp1l	0.0000	-0.6505	0.0194	0.0000	-1.3645	0.0042
Foxk1	0.0000	-0.8389	0.0177	0.0000	-1.0213	0.0137
Fras1	0.2712	-0.1505	0.0793	0.0000	-1.3151	0.0185
Frmd3	0.1104	-0.5835	0.0717	0.0001	-1.4530	0.0480
Frmd4a	0.0000	-0.9799	0.0102	0.0000	-1.7710	0.0025
Frmd5	0.0000	-0.8256	0.0266	0.0000	-1.6061	0.0094
Fry	0.0003	-0.4976	0.0493	0.0000	-1.3788	0.0172
Fsd1	0.0000	-0.8836	0.0277	0.0000	-1.2919	0.0173
Fsd1l	0.3073	0.0995	0.0805	0.0000	-1.4978	0.0074
Fstl4	0.0120	-0.9213	0.0604	0.0000	-2.5187	0.0309
Fth1	0.0000	0.3883	0.0380	0.0000	1.9347	0.0014
Fxr1	0.0000	0.9301	0.0078	0.0000	1.0872	0.0061
Fzd3	0.0000	-0.6947	0.0211	0.0000	-2.1199	0.0015
Gab1	0.0000	0.6879	0.0230	0.0000	1.2402	0.0085
Gabra1	0.2035	-0.4936	0.0768	0.0000	-1.7141	0.0452
Gabra2	0.0249	-0.5614	0.0636	0.0000	-2.3195	0.0212
Gabra3	0.0028	-0.7421	0.0555	0.0000	-1.5571	0.0325
Gabrg2	0.0005	-0.7005	0.0503	0.0000	-1.6386	0.0235
Gadd45a	0.0000	0.8305	0.0273	0.0000	1.5632	0.0099
Gadd45b	0.3938	-0.1350	0.0834	0.0000	1.3818	0.0191

Gadd45gip	0.8556	-0.0203	0.0961	0.0000	1.1257	0.0154
Galnt13	0.2084	-0.2809	0.0770	0.0000	-2.0862	0.0207
Galnt14	0.0063	-0.8611	0.0582	0.0000	-2.9572	0.0222
Galnt7	0.0000	0.9082	0.0174	0.0000	1.1053	0.0133
Gas6	0.0000	0.8884	0.0273	0.0000	1.7918	0.0086
Gatad2a	0.0000	0.7063	0.0167	0.0000	1.0273	0.0086
Gbp2	0.0177	0.7046	0.0620	0.0000	1.1729	0.0466
Gcdh	0.0000	0.8047	0.0328	0.0000	1.3097	0.0180
Gcnt2	0.0000	0.7396	0.0349	0.0000	1.6538	0.0109
Gdf11	0.0000	-0.6236	0.0305	0.0000	-1.3759	0.0092
Gdpd1	0.0000	-0.8310	0.0204	0.0000	-1.3766	0.0087
Gfap	0.3398	0.7645	0.0816	0.0000	8.1019	0.0098
Gga2	0.0000	0.7721	0.0141	0.0000	1.0443	0.0083
Gipc1	0.0000	0.7134	0.0213	0.0000	1.0247	0.0123
Gjb6	0.0975	-0.7347	0.0709	0.0000	1.6669	0.0448
Glb1	0.1219	0.1952	0.0725	0.0000	1.1316	0.0190
Glcci1	0.0090	-0.9643	0.0593	0.0000	-1.8547	0.0393
Gli2	0.0000	0.6728	0.0279	0.0000	1.1674	0.0124
Gli3	0.0001	0.3712	0.0477	0.0000	-1.2450	0.0112
Glrx	0.0000	0.6808	0.0434	0.0000	1.5120	0.0172
Gmip	0.0000	-0.8139	0.0449	0.0000	-1.1672	0.0337
Golga1	0.0010	-0.4200	0.0523	0.0000	-1.4793	0.0134
Got1	0.0000	0.5832	0.0289	0.0000	1.8866	0.0025
Gphn	0.0000	-0.4751	0.0422	0.0000	-1.2343	0.0134
Gpm6a	0.9663	-0.0037	0.0990	0.0000	-1.0241	0.0131
Gpm6b	0.0000	-0.9174	0.0151	0.0000	-1.2550	0.0090
Gpr139	0.4829	0.2170	0.0860	0.0000	-1.5615	0.0416
Gpr156	0.6491	0.1653	0.0907	0.0000	-1.6941	0.0444
Gprasp1	0.0000	-0.9551	0.0112	0.0000	-1.5873	0.0036
Gprc5a	0.0055	0.6389	0.0577	0.0000	2.5261	0.0122
Gpt2	0.0000	0.8001	0.0166	0.0000	1.2267	0.0076
Gpx1	0.0000	0.9285	0.0094	0.0000	1.5413	0.0027
Gpx3	0.0082	0.3477	0.0591	0.0000	1.2282	0.0181
Greb1l	0.3747	-0.1587	0.0827	0.0000	-1.1144	0.0324
Grem2	0.0000	-0.7433	0.0451	0.0000	-2.3883	0.0112
Gria1	0.5182	0.1293	0.0870	0.0000	-2.4998	0.0135
Grid1	0.0001	-0.7150	0.0470	0.0000	-1.2908	0.0281
Grid2	0.0119	-0.6910	0.0604	0.0000	-1.7866	0.0314
Grin2a	0.0391	-0.6561	0.0657	0.0000	-1.4408	0.0435
Grin2c	0.5947	0.2109	0.0891	0.0000	1.7537	0.0412
Grip1	0.0000	-0.6515	0.0299	0.0000	-1.0815	0.0156
Grn	0.0000	0.6492	0.0255	0.0000	1.5013	0.0052
Gsr	0.0000	0.9555	0.0144	0.0000	2.4861	0.0009
Gusb	0.0393	0.2724	0.0657	0.0000	1.4347	0.0135
Gypc	0.4001	-0.2336	0.0836	0.0000	1.6316	0.0320
H2ac18	0.0001	0.8870	0.0455	0.0000	1.4446	0.0291
Hapln4	0.2230	-0.4562	0.0775	0.0000	1.5828	0.0418
Hdhd3	0.0002	-0.8790	0.0480	0.0000	-1.1380	0.0400
Hdx	0.0000	-0.9128	0.0383	0.0000	-1.2224	0.0297
Hebp1	0.0006	0.4058	0.0511	0.0000	1.5358	0.0094
Hectd4	0.0000	-0.8534	0.0183	0.0000	-2.1432	0.0023
Hecw1	0.0083	-0.5182	0.0591	0.0000	-2.1518	0.0157
Herc1	0.0000	-0.6099	0.0214	0.0000	-1.6193	0.0026
Herc2	0.0000	-0.3762	0.0373	0.0000	-1.3498	0.0044
Hes1	0.5810	0.0579	0.0888	0.0000	1.1989	0.0121
Hexa	0.0000	0.7173	0.0192	0.0000	1.7595	0.0024
Hhipl1	0.9350	-0.0157	0.0981	0.0000	-1.3967	0.0282
Hid1	0.0530	0.2727	0.0671	0.0000	-1.1646	0.0239
Hipk2	0.0000	-0.9547	0.0146	0.0000	-1.9317	0.0031
Hirip3	0.0000	0.7996	0.0281	0.0000	1.2287	0.0160
Hivep1	0.0000	-0.6861	0.0256	0.0000	-1.1933	0.0110
Hjurp	0.0000	0.6496	0.0272	0.0000	1.0126	0.0149
Hmga2	0.0000	-0.7888	0.0271	0.0000	-1.7008	0.0077
Hmgb3	0.0000	-0.5267	0.0257	0.0000	-1.7153	0.0020
Hmgcr	0.0000	-0.7398	0.0139	0.0000	-1.1250	0.0063
Hp1bp3	0.0000	-0.9033	0.0087	0.0000	-1.5055	0.0025
Hs1bp3	0.0000	0.7710	0.0254	0.0000	1.3378	0.0103
Hs3st1	0.1248	-0.4920	0.0726	0.0000	-2.1516	0.0317
Hscb	0.0203	0.3974	0.0626	0.0000	1.1330	0.0286
Hsd3b7	0.0017	0.7634	0.0539	0.0000	1.6249	0.0281
Hsdl2	0.0000	-0.7757	0.0265	0.0000	-1.0789	0.0174
Hspa1a	0.0006	0.8329	0.0512	0.0000	1.6490	0.0278
Hspa4l	0.1085	-0.1598	0.0716	0.0000	-1.3048	0.0101
Hspb2	0.1434	0.7174	0.0737	0.0000	2.5739	0.0351
Hspb6	0.4040	0.2048	0.0838	0.0000	1.3799	0.0334
Hvcn1	0.0000	0.9460	0.0394	0.0000	1.4324	0.0261
Icam1	0.0005	0.4973	0.0506	0.0000	3.4210	0.0012

Id2	0.0000	-0.4836	0.0369	0.0000	-1.3086	0.0089
Ide	0.0000	0.9786	0.0103	0.0000	1.0561	0.0099
Idh2	0.0058	0.2082	0.0579	0.0000	1.2007	0.0059
Ier2	0.0000	0.6588	0.0284	0.0000	1.0774	0.0145
Ifitm2	0.0000	0.9549	0.0130	0.0000	2.5715	0.0007
Igfbp2	0.0000	0.7145	0.0215	0.0000	1.1842	0.0093
Igfbp6	0.0421	0.8911	0.0660	0.0000	3.0569	0.0253
Igip	0.3422	0.2715	0.0817	0.0003	1.0032	0.0500
Igsf10	0.0010	0.4921	0.0525	0.0000	1.5524	0.0149
Il33	0.4136	0.4138	0.0841	0.0000	3.4237	0.0284
Il3ra	0.0944	0.4638	0.0707	0.0000	2.1676	0.0220
Il4i1	0.8138	0.1845	0.0950	0.0000	3.2490	0.0412
Immp2l	0.9887	-0.0042	0.0996	0.0001	1.0970	0.0478
Inf2	0.0000	-0.7010	0.0447	0.0000	1.5601	0.0165
Inpp4a	0.0000	-0.8033	0.0229	0.0000	-1.3803	0.0096
Inpp5f	0.0000	-0.6185	0.0267	0.0000	-1.5835	0.0046
Ip6k2	0.0000	-0.7961	0.0185	0.0000	-1.0108	0.0133
Iqgap3	0.0000	0.9660	0.0252	0.0000	1.0166	0.0249
Islr2	0.0000	-0.3899	0.0427	0.0000	-1.9073	0.0028
Itga5	0.0000	0.6719	0.0289	0.0000	1.4062	0.0087
Itga6	0.0000	0.9485	0.0136	0.0000	1.3870	0.0066
Itgb3	0.0005	-0.7971	0.0508	0.0000	1.0908	0.0376
Itgb4	0.0034	0.8734	0.0561	0.0000	1.5983	0.0358
Itgb5	0.1119	-0.1795	0.0718	0.0000	1.1280	0.0162
Itprip	0.0330	-0.3029	0.0649	0.0000	-1.5258	0.0157
Jakmip2	0.0000	-0.7739	0.0256	0.0000	-2.7637	0.0017
Jdp2	0.0270	-0.3253	0.0640	0.0000	1.5271	0.0142
Junb	0.0127	0.4588	0.0607	0.0000	1.6091	0.0200
Kank2	0.0000	0.5465	0.0335	0.0000	1.8901	0.0032
Kank4	0.9979	-0.0010	0.0999	0.0000	2.0626	0.0350
Kcmf1	0.0000	0.9070	0.0144	0.0000	1.0050	0.0131
Kcna1	0.1799	-0.5276	0.0757	0.0000	-6.8945	0.0313
Kcna5	0.0001	-0.9520	0.0461	0.0000	-2.2387	0.0201
Kcnf1	0.0037	-0.9247	0.0564	0.0000	-1.5332	0.0411
Kcnh2	0.0000	-0.9826	0.0276	0.0000	-1.4713	0.0166
Kcnh8	0.2267	-0.4276	0.0776	0.0000	-3.0707	0.0266
Kcnj4	0.0051	0.8154	0.0574	0.0000	2.3158	0.0218
Kcnk2	0.0000	-0.8819	0.0338	0.0000	-1.4182	0.0200
Kcnk9	0.8138	-0.0481	0.0950	0.0000	-1.6650	0.0243
Kctd21	0.0000	-0.9872	0.0410	0.0000	-1.1339	0.0371
Kctd6	0.0013	0.5121	0.0531	0.0000	1.5354	0.0166
Kctd8	0.0458	-0.7390	0.0664	0.0000	-4.0830	0.0225
Kdelr3	0.0000	-0.7602	0.0352	0.0000	1.3257	0.0163
Kdm1b	0.0000	-0.6034	0.0369	0.0000	-1.1362	0.0180
Kdm4b	0.0000	-0.7803	0.0175	0.0000	-1.2532	0.0075
Kdsr	0.0000	0.8007	0.0280	0.0000	1.3263	0.0136
Khdrbs1	0.0000	-0.5918	0.0205	0.0000	-1.1110	0.0065
Khdrbs3	0.0000	-0.6087	0.0289	0.0000	-1.2210	0.0101
Kif1a	0.0000	-0.4755	0.0278	0.0000	-1.2655	0.0046
Kif21a	0.0000	-0.6659	0.0293	0.0000	-1.9891	0.0038
Kif21b	0.0000	-0.6181	0.0209	0.0000	-1.7295	0.0020
Kif26b	0.0000	-0.7556	0.0310	0.0000	-1.9829	0.0066
Kif3a	0.0000	-0.8925	0.0184	0.0000	-1.4336	0.0080
Klf7	0.0006	-0.3409	0.0513	0.0000	-1.1937	0.0123
Klhdc8b	0.0078	-0.2927	0.0588	0.0000	-1.0810	0.0176
Klhl23	0.0000	-0.9337	0.0200	0.0000	-1.4468	0.0097
Klhl29	0.0035	-0.4194	0.0562	0.0000	-1.5471	0.0155
Klhl7	0.0000	-0.6506	0.0224	0.0000	-1.1711	0.0083
Kmt2a	0.0000	-0.5407	0.0295	0.0000	-1.3633	0.0062
Kxd1	0.0000	0.8559	0.0224	0.0000	1.2873	0.0120
L3mbtl3	0.0033	-0.3424	0.0560	0.0000	-1.3212	0.0139
Lama4	0.0000	-0.8272	0.0337	0.0000	2.7123	0.0024
Larp1b	0.0927	0.3031	0.0705	0.0000	1.1194	0.0310
Lcor	0.0000	-0.7748	0.0247	0.0000	-1.9752	0.0042
Ldb3	0.9554	-0.0193	0.0987	0.0000	1.9386	0.0324
Ldlrap1	0.1237	0.2553	0.0726	0.0000	1.0273	0.0310
Lfng	0.0001	-0.5743	0.0464	0.0000	-1.2131	0.0226
Lgals9	0.0001	0.8060	0.0468	0.0000	1.6299	0.0230
Lif	0.1705	0.3646	0.0753	0.0000	1.6105	0.0309
Lonrf2	0.0004	-0.4101	0.0502	0.0000	-1.5445	0.0100
Lpar6	0.1277	0.2210	0.0729	0.0000	-1.1662	0.0246
Lpl	0.0835	0.2287	0.0697	0.0000	2.4557	0.0034
Lrp11	0.0000	0.6286	0.0332	0.0000	1.3449	0.0109
Lrp5	0.0000	0.4234	0.0433	0.0000	1.0562	0.0144
Lrrc17	0.7473	-0.0941	0.0934	0.0000	3.1748	0.0113
Lrsam1	0.0000	-0.7233	0.0389	0.0000	-1.1488	0.0248
Lsm14b	0.0000	-0.9216	0.0125	0.0000	-1.0663	0.0104

Lsm4	0.0000	0.9007	0.0213	0.0000	1.2274	0.0137
Ltbp4	0.1446	0.3484	0.0738	0.0000	1.7524	0.0263
Ltbr	0.0001	-0.9250	0.0468	0.0000	1.3150	0.0317
Ly6e	0.4018	0.1089	0.0837	0.0000	1.0517	0.0224
Lztr1	0.0000	-0.5759	0.0313	0.0000	-1.0334	0.0147
Lzts1	0.4801	-0.0883	0.0860	0.0000	-2.3966	0.0047
Macrod2	0.0032	-0.4522	0.0559	0.0000	-1.2227	0.0241
Maf	0.9514	-0.0081	0.0986	0.0000	-1.0039	0.0258
Mak16	0.0000	0.9977	0.0113	0.0000	1.1639	0.0091
Mamld1	0.0661	-0.4721	0.0684	0.0000	-1.3364	0.0387
Map1a	0.0000	-0.9959	0.0114	0.0000	-1.3558	0.0065
Map2	0.0000	-0.8992	0.0083	0.0000	-2.1868	0.0004
Map2k3	0.0000	0.7701	0.0316	0.0000	1.0635	0.0219
Mapk10	0.0000	-0.8280	0.0203	0.0000	-2.3881	0.0019
Mapk8ip1	0.0000	-0.9806	0.0093	0.0000	-1.7446	0.0023
Mapre2	0.0156	-0.2056	0.0615	0.0000	-1.6444	0.0037
Marcksl1	0.0000	-0.9689	0.0059	0.0000	-1.0092	0.0064
Matn3	0.0516	0.7363	0.0671	0.0000	2.3295	0.0301
Mavs	0.0000	0.9689	0.0264	0.0000	1.3487	0.0169
Mbd5	0.0000	-0.8566	0.0273	0.0000	-1.4366	0.0133
Mbnl2	0.2764	0.1314	0.0794	0.0000	1.2087	0.0163
Mcam	0.0000	0.6183	0.0406	0.0000	1.4054	0.0144
Mccc1	0.0000	0.5702	0.0449	0.0000	1.2114	0.0199
Mcf2l	0.0000	-0.7519	0.0239	0.0000	-1.2414	0.0112
Mcoln1	0.0000	0.7717	0.0269	0.0000	1.1158	0.0163
Mdfic	0.0000	0.6122	0.0443	0.0000	1.4166	0.0171
Mecp2	0.0000	-0.9512	0.0122	0.0000	-1.3610	0.0063
Med12l	0.0080	-0.3699	0.0590	0.0000	-1.1251	0.0237
Med26	0.0000	0.8043	0.0288	0.0000	1.0057	0.0224
Mef2b	0.0008	0.9239	0.0517	0.0000	2.7471	0.0151
Megf11	0.5058	-0.1007	0.0866	0.0000	-1.5011	0.0180
Megf6	0.0000	-0.8599	0.0232	0.0000	-1.5322	0.0092
Metrn	0.0000	-0.5356	0.0377	0.0000	-1.1493	0.0149
Mettl5	0.0000	0.7907	0.0353	0.0000	1.0836	0.0256
Mex3a	0.0000	-0.9592	0.0084	0.0000	-1.4417	0.0033
Mfge8	0.0000	0.9950	0.0155	0.0000	1.5369	0.0068
Mfn2	0.0000	-0.8801	0.0180	0.0000	-1.1456	0.0123
Mfsd13a	0.0000	0.7164	0.0433	0.0000	1.0086	0.0324
Mfsd2a	0.0013	-0.7037	0.0531	0.0000	-3.1570	0.0099
Mgat5	0.0081	0.2354	0.0590	0.0000	-1.0732	0.0122
Mgme1	0.0199	0.3219	0.0625	0.0000	1.6114	0.0114
Mid2	0.0000	-0.8158	0.0382	0.0000	-1.8710	0.0141
Midn	0.0000	-0.7692	0.0149	0.0000	-1.1556	0.0071
Mipol1	0.0001	0.6193	0.0460	0.0000	1.3170	0.0209
Mir17hg	0.0528	0.6417	0.0671	0.0000	2.1435	0.0288
Mllt1	0.0001	-0.3202	0.0471	0.0000	-1.0605	0.0105
Mllt3	0.0055	-0.2682	0.0577	0.0000	-1.5423	0.0065
Mmd	0.0000	-0.7610	0.0228	0.0000	-1.5793	0.0062
Mmgt1	0.0000	-0.5599	0.0306	0.0000	-1.0111	0.0139
Mmp14	0.0004	0.4202	0.0500	0.0000	1.3916	0.0121
Mmp16	0.0001	-0.6538	0.0454	0.0000	-1.3264	0.0230
Mmp19	0.5970	0.2582	0.0892	0.0002	1.7088	0.0493
Mmp2	0.0000	-0.8235	0.0246	0.0000	1.2881	0.0124
Mmp9	0.0197	0.9326	0.0625	0.0000	3.7106	0.0161
Mob3c	0.0649	0.4990	0.0682	0.0000	1.2297	0.0418
Mpdz	0.0001	-0.3554	0.0472	0.0000	-1.5343	0.0059
Mpeg1	0.0722	-0.6928	0.0689	0.0000	-2.2278	0.0368
Mpzl2	0.0003	0.6020	0.0490	0.0000	2.5247	0.0060
Mr1	0.0001	0.9868	0.0456	0.0000	2.3504	0.0167
Mrgbp	0.0000	-0.7696	0.0318	0.0000	-1.0908	0.0217
Mri1	0.0000	0.7473	0.0377	0.0000	1.4739	0.0163
Mrpl17	0.0000	0.7025	0.0258	0.0000	1.1472	0.0124
Mrps28	0.0000	0.7423	0.0379	0.0000	1.4101	0.0175
Mt3	0.6184	0.1012	0.0898	0.0000	1.9738	0.0155
Mthfs	0.1917	0.4245	0.0763	0.0001	1.2502	0.0470
Mtmr1	0.0000	-0.6191	0.0401	0.0000	-1.2316	0.0191
Mtmr12	0.0000	0.9601	0.0220	0.0000	1.0242	0.0213
Mtmr4	0.0000	-0.6177	0.0238	0.0000	-1.6442	0.0034
Mtmr6	0.0000	-0.7796	0.0212	0.0000	-1.0795	0.0133
Mtrr	0.0006	0.4257	0.0512	0.0000	1.1807	0.0174
Mtx3	0.0000	-0.7045	0.0370	0.0000	-1.2387	0.0202
Mvb12a	0.0684	0.2255	0.0685	0.0000	1.1097	0.0190
Mycbp2	0.0000	-0.3557	0.0402	0.0000	-1.3615	0.0046
Myo10	0.0000	0.8504	0.0118	0.0000	1.2143	0.0059
Myo16	0.0000	-0.9022	0.0259	0.0000	-1.9901	0.0069
Myo9a	0.0000	-0.4600	0.0364	0.0000	-1.1585	0.0100
Myom2	0.9456	-0.0319	0.0984	0.0000	2.0298	0.0416

Naaa	0.0014	0.5201	0.0533	0.0000	1.0205	0.0308
Nacc1	0.0000	0.8413	0.0114	0.0000	1.3056	0.0044
Nagpa	0.0000	0.8877	0.0444	0.0000	1.4438	0.0279
Nalcn	0.0312	-0.3298	0.0646	0.0000	-1.3724	0.0207
Narf	0.0000	-0.9910	0.0100	0.0000	-1.2215	0.0071
Nars	0.0000	0.9922	0.0082	0.0000	1.1545	0.0066
Nbea	0.0000	-0.7418	0.0221	0.0000	-2.2731	0.0018
Ncald	0.7210	-0.0382	0.0927	0.0000	-1.2201	0.0139
Nceh1	0.0000	0.7626	0.0361	0.0000	1.5354	0.0145
Ncoa5	0.0111	-0.2196	0.0601	0.0000	-1.1322	0.0101
Ndn	0.0000	-0.9801	0.0094	0.0000	-1.2258	0.0063
Ndrg2	0.0001	-0.6399	0.0460	0.0000	1.0684	0.0282
Ndst1	0.0000	-0.9639	0.0104	0.0000	-1.2999	0.0060
Ndst3	0.0266	0.4979	0.0639	0.0000	-1.4818	0.0323
Ndufa13	0.0000	0.8116	0.0284	0.0000	1.0307	0.0220
Ndufb11	0.0000	0.9573	0.0100	0.0000	1.0924	0.0085
Nebl	0.0016	0.5787	0.0537	0.0000	-1.2181	0.0318
Nedd4l	0.0000	-0.9517	0.0203	0.0000	-1.3293	0.0125
Nek7	0.0000	0.7843	0.0222	0.0000	1.0201	0.0157
Nell1	0.0000	-0.9452	0.0426	0.0000	-1.6608	0.0254
Neto1	0.4632	0.2837	0.0854	0.0000	-2.1935	0.0393
Neto2	0.0012	-0.3703	0.0530	0.0000	-1.9444	0.0057
Neu3	0.0463	0.4929	0.0665	0.0000	1.3651	0.0346
Neurl4	0.0000	-0.5881	0.0270	0.0000	-1.0673	0.0109
Nf1	0.0000	-0.4281	0.0417	0.0000	-1.3224	0.0089
Nfasc	0.0000	-0.5462	0.0312	0.0000	-1.9065	0.0029
Nfe2l2	0.0000	0.7168	0.0263	0.0000	1.7160	0.0049
Nfe2l3	0.0214	-0.7366	0.0628	0.0000	-1.5033	0.0423
Nfkb2	0.0000	0.9503	0.0317	0.0000	1.5001	0.0180
Nfkbiz	0.0014	-0.8173	0.0533	0.0000	1.1383	0.0400
Nhlh2	0.0039	-0.6901	0.0565	0.0000	-2.7625	0.0131
Nid2	0.0000	0.5573	0.0280	0.0000	1.3880	0.0054
Nin	0.0000	-0.6816	0.0312	0.0000	-1.3649	0.0119
Nkain2	0.0650	-0.6224	0.0682	0.0000	-1.4910	0.0443
Nkain4	0.0080	-0.3740	0.0590	0.0000	-1.0656	0.0257
Nle1	0.0000	0.8884	0.0319	0.0000	1.0886	0.0262
Nmnat2	0.0000	-0.9124	0.0192	0.0000	-1.0879	0.0155
Nnat	0.0000	-0.6577	0.0186	0.0000	-1.5793	0.0026
Npl	0.0001	0.9432	0.0456	0.0000	1.0109	0.0442
Nr2c2	0.0000	-0.6684	0.0250	0.0000	-1.1281	0.0113
Nr4a1	0.0001	0.7008	0.0468	0.0000	1.6230	0.0187
Nrg1	0.2782	-0.1848	0.0795	0.0000	-1.5506	0.0207
Nrip1	0.0011	-0.4173	0.0525	0.0000	-1.0635	0.0223
Nsd2	0.0000	-0.4389	0.0318	0.0000	-1.4552	0.0034
Nsdhl	0.0000	-0.9649	0.0140	0.0000	-1.1822	0.0103
Nsl1	0.0000	0.8483	0.0322	0.0000	1.2062	0.0216
Nt5c2	0.0053	-0.2653	0.0576	0.0000	-1.0430	0.0146
Nthl1	0.0015	0.5103	0.0536	0.0000	1.3702	0.0208
Ntpcr	0.0000	0.9073	0.0372	0.0000	1.0971	0.0318
Ntrk1	0.0078	0.7163	0.0589	0.0000	-1.3242	0.0426
Nuak1	0.0000	-0.7282	0.0285	0.0000	-1.2293	0.0141
Nudt10	0.0000	-0.7838	0.0383	0.0000	-1.3107	0.0227
Nupr1	0.0001	0.6980	0.0460	0.0000	4.1393	0.0011
Nxn	0.0001	-0.3713	0.0457	0.0000	-1.4837	0.0064
Nyap2	0.0000	-0.8965	0.0440	0.0000	-1.6890	0.0245
Onecut2	0.8343	0.0285	0.0956	0.0000	-2.5303	0.0048
Opn3	0.3360	-0.2311	0.0815	0.0000	-1.0052	0.0460
Orai2	0.0000	-0.8509	0.0344	0.0000	-1.1000	0.0270
Orai3	0.0534	0.2695	0.0672	0.0000	1.3327	0.0169
Orc5	0.0000	0.9677	0.0194	0.0000	1.0045	0.0197
Osbp2	0.0033	-0.8007	0.0560	0.0000	-1.4777	0.0370
Osbpl8	0.0000	-0.4718	0.0406	0.0000	-1.1131	0.0145
Padi2	0.9951	-0.0021	0.0999	0.0000	2.7113	0.0198
Pafah1b3	0.0000	-0.9296	0.0168	0.0000	-1.5417	0.0066
Pag1	0.6734	-0.0534	0.0913	0.0000	-1.3007	0.0168
Pak1	0.0000	-0.6394	0.0227	0.0000	-1.3824	0.0054
Pak4	0.0000	0.7985	0.0218	0.0000	1.2619	0.0102
Panx1	0.0000	-0.6521	0.0266	0.0000	-1.7239	0.0042
Papss1	0.0000	-0.9193	0.0136	0.0000	-1.0978	0.0105
Parp11	0.0000	-0.8717	0.0218	0.0000	-1.3052	0.0119
Pax7	0.0153	-0.5265	0.0614	0.0000	-2.9079	0.0111
Pbx1	0.0000	-0.9677	0.0107	0.0000	-1.1700	0.0080
Pcbp4	0.0000	-0.8744	0.0161	0.0000	-1.0912	0.0118
Pcdh1	0.0000	-0.5320	0.0450	0.0000	-1.2370	0.0185
Pcdh15	0.3150	-0.4340	0.0808	0.0000	-2.4853	0.0379
Pcdh7	0.0115	0.7755	0.0602	0.0000	-1.3985	0.0429
Pcdh8	0.0000	-0.6223	0.0394	0.0000	-1.6377	0.0109

Pcdha1	0.0833	-0.4682	0.0697	0.0000	-1.1329	0.0458
Pcdha9	0.3325	-0.2119	0.0814	0.0000	-1.5660	0.0285
Pcdhb11	0.0061	-0.3386	0.0580	0.0000	-1.1461	0.0193
Pcdhb18	0.0000	-0.7660	0.0395	0.0000	-1.0473	0.0301
Pcdhb2	0.0046	-0.3988	0.0571	0.0000	-1.6301	0.0138
Pcdhb6	0.7035	-0.0676	0.0923	0.0000	-1.0833	0.0334
Pck2	0.0000	0.9710	0.0169	0.0000	2.0467	0.0029
Pcsk2	0.9666	-0.0067	0.0990	0.0000	-1.7707	0.0155
Pcsk5	0.5612	0.0953	0.0883	0.0000	-1.6557	0.0179
Pde2a	0.6078	0.0885	0.0895	0.0000	-1.1799	0.0299
Pde4c	0.1161	0.8096	0.0721	0.0000	3.2375	0.0282
Pde4dip	0.0000	-0.5262	0.0358	0.0000	-1.0447	0.0155
Pde7b	0.5387	-0.3474	0.0876	0.0000	2.2658	0.0426
Pdgfb	0.3633	0.3377	0.0824	0.0002	1.3008	0.0498
Pdgfrl	0.9358	-0.0205	0.0981	0.0000	2.2615	0.0176
Pdlim2	0.2247	0.3062	0.0776	0.0000	1.1290	0.0419
Pdp1	0.0000	-0.9376	0.0203	0.0000	-1.3642	0.0114
Pdyn	0.0888	0.5908	0.0702	0.0000	2.5277	0.0240
Peli1	0.0000	-0.8704	0.0161	0.0000	-1.3594	0.0072
Perp	0.8324	-0.0332	0.0955	0.0000	1.5819	0.0147
Pex2	0.0000	-0.4848	0.0417	0.0000	-1.1981	0.0141
Pex5l	0.0022	0.6954	0.0547	0.0000	-2.0276	0.0242
Pfn2	0.0000	-0.9174	0.0104	0.0000	-1.4664	0.0037
Pgghg	0.0000	0.7330	0.0359	0.0000	1.0626	0.0243
Phc1	0.0000	0.7811	0.0141	0.0000	1.5613	0.0027
Phc2	0.0000	-0.7398	0.0171	0.0000	-1.3173	0.0055
Phf14	0.0004	-0.2798	0.0499	0.0000	-1.1282	0.0081
Phf19	0.0006	0.7292	0.0511	0.0000	2.8930	0.0076
Phf2	0.0000	-0.7885	0.0186	0.0000	-1.4116	0.0063
Phf20l1	0.0001	-0.3892	0.0465	0.0000	-1.3041	0.0101
Phf6	0.0000	-0.4674	0.0373	0.0000	-1.3142	0.0082
Phldb1	0.0000	-0.8600	0.0190	0.0000	-1.1087	0.0133
Pif1	0.0000	0.6903	0.0426	0.0000	1.2322	0.0237
Pigl	0.0000	0.8856	0.0331	0.0000	1.0090	0.0299
Pik3c2b	0.0032	-0.3550	0.0559	0.0000	-1.2824	0.0155
Pik3ca	0.0000	-0.7066	0.0237	0.0000	-1.1532	0.0114
Pik3cd	0.0000	-0.9223	0.0378	0.0000	-1.2648	0.0283
Pik3r1	0.0000	-0.7322	0.0303	0.0000	-1.3075	0.0140
Pik3r3	0.0000	-0.8178	0.0110	0.0000	-2.3040	0.0004
Pim1	0.0000	0.5662	0.0363	0.0000	1.6334	0.0067
Pim3	0.0000	0.8074	0.0254	0.0000	1.6702	0.0069
Pip4k2c	0.0000	0.5799	0.0346	0.0000	1.0896	0.0155
Pja2	0.0000	-0.8616	0.0120	0.0000	-1.2470	0.0060
Pla2g12a	0.0502	-0.3145	0.0669	0.0000	1.2947	0.0211
Pla2g4a	0.0658	0.4656	0.0683	0.0000	1.9929	0.0229
Plagl2	0.1982	-0.1444	0.0766	0.0000	-1.1327	0.0170
Plaur	0.1934	0.4220	0.0764	0.0000	1.6629	0.0367
Plch1	0.0001	-0.4575	0.0462	0.0000	-1.6286	0.0089
Plch2	0.0065	-0.7538	0.0583	0.0000	-1.7761	0.0321
Plcxd2	0.0000	-0.9436	0.0262	0.0000	-1.8652	0.0090
Plekha4	0.0677	0.6678	0.0685	0.0001	1.4200	0.0467
Plekha6	0.3671	-0.0834	0.0825	0.0000	-1.9457	0.0032
Plekha7	0.0000	0.9684	0.0250	0.0000	1.6057	0.0115
Plekhf1	0.0000	0.8365	0.0433	0.0000	2.0363	0.0143
Plk3	0.0001	-0.6843	0.0454	0.0000	-1.0789	0.0310
Plxna1	0.0000	-0.7373	0.0162	0.0000	-1.0796	0.0084
Plxnb1	0.0000	-0.7252	0.0203	0.0000	-1.8053	0.0029
Plxnc1	0.0000	-0.8326	0.0288	0.0000	-1.5631	0.0118
Pmm1	0.0000	0.8600	0.0164	0.0000	1.4416	0.0058
Pogk	0.0000	-0.8386	0.0147	0.0000	-1.6288	0.0034
Polr2e	0.0000	0.6711	0.0235	0.0000	1.4923	0.0050
Polr3k	0.0000	-0.7771	0.0187	0.0000	-1.1591	0.0096
Pou3f3	0.0000	-0.9382	0.0151	0.0000	-1.7228	0.0042
Ppargc1b	0.0167	0.6666	0.0618	0.0000	1.6701	0.0317
Pphln1	0.0017	-0.3365	0.0539	0.0000	-1.0764	0.0169
Ppm1m	0.0000	0.7101	0.0419	0.0000	1.4170	0.0193
Ppme1	0.0000	-0.3809	0.0416	0.0000	-1.1407	0.0094
Ppp1r14a	0.2377	0.3416	0.0781	0.0000	1.4951	0.0366
Ppp1r15a	0.0348	0.2961	0.0652	0.0000	1.0608	0.0247
Ppp1r26	0.0000	-0.8860	0.0259	0.0000	-1.1261	0.0196
Ppp1r3b	0.3051	0.1732	0.0804	0.0000	1.6530	0.0163
Ppp1r9a	0.0000	-0.6679	0.0260	0.0000	-1.3417	0.0082
Ppp2r5a	0.0000	0.5460	0.0328	0.0000	1.1806	0.0103
Ppp6r2	0.0000	-0.9468	0.0163	0.0000	-1.0528	0.0147
Prdm2	0.0000	-0.6553	0.0232	0.0000	-1.0267	0.0119
Prelp	0.0047	-0.5445	0.0571	0.0000	2.0611	0.0116
Prepl	0.0000	-0.8058	0.0173	0.0000	-1.2016	0.0087

Prex1	0.0000	-0.5857	0.0325	0.0000	-1.3648	0.0094
Prima1	0.1357	-0.7673	0.0733	0.0002	-1.9934	0.0497
Prkch	0.0017	-0.6793	0.0540	0.0000	-1.4934	0.0291
Prkci	0.0000	0.8411	0.0168	0.0000	1.1414	0.0102
Prkg1	0.0203	-0.5272	0.0626	0.0000	-1.3511	0.0338
Prkra	0.0000	-0.6161	0.0342	0.0000	-1.1391	0.0162
Prmt2	0.0000	-0.8806	0.0172	0.0000	-1.5087	0.0062
Prr36	0.0000	-0.8946	0.0240	0.0000	-1.7419	0.0081
Prss35	0.0000	0.9676	0.0447	0.0000	2.1421	0.0186
Prx	0.0000	0.7324	0.0422	0.0000	1.2980	0.0233
Psat1	0.0000	0.9194	0.0165	0.0000	1.0885	0.0135
Psrc1	0.0000	0.5909	0.0345	0.0000	1.1519	0.0147
Ptbp2	0.0000	-0.7018	0.0198	0.0000	-1.7065	0.0029
Ptchd4	0.6541	-0.3610	0.0908	0.0003	-3.8454	0.0500
Ptgds	0.9184	-0.0518	0.0977	0.0002	1.7342	0.0492
Ptprd	0.0000	-0.7257	0.0340	0.0000	-2.5066	0.0039
Ptprm	0.0002	-0.6183	0.0478	0.0000	-1.1597	0.0276
Pttg1	0.0000	0.9938	0.0256	0.0000	1.4689	0.0147
Ptx3	0.8511	-0.0432	0.0960	0.0000	1.9056	0.0209
R3hdm4	0.0000	-0.9990	0.0111	0.0000	-1.0327	0.0116
Rab8b	0.0000	-0.7126	0.0248	0.0000	-1.2353	0.0106
Rabgap1l	0.0000	-0.9703	0.0187	0.0000	-1.2276	0.0136
Rac3	0.0000	-0.6104	0.0388	0.0000	-1.2468	0.0173
Rad18	0.0000	0.9893	0.0214	0.0000	1.4582	0.0113
Rad51b	0.0012	0.9156	0.0528	0.0000	1.1205	0.0466
Rad51c	0.0000	0.9160	0.0292	0.0000	1.3064	0.0189
Rad9a	0.0000	0.7893	0.0321	0.0000	1.0652	0.0233
Rae1	0.0000	0.7533	0.0190	0.0000	1.0582	0.0109
Rai1	0.0000	-0.7790	0.0188	0.0000	-1.0687	0.0115
Ralgapa1	0.5707	-0.0521	0.0885	0.0000	-1.3515	0.0080
Ranbp6	0.0000	-0.8758	0.0216	0.0000	-1.5752	0.0079
Rapgef6	0.0000	-0.3779	0.0444	0.0000	-1.2866	0.0086
Rarg	0.0050	0.4434	0.0574	0.0000	1.5634	0.0166
Rasal2	0.0005	-0.3446	0.0503	0.0000	-1.0267	0.0158
Rasgef1b	0.0000	-0.9097	0.0417	0.0000	-1.0500	0.0375
Rassf4	0.0001	0.6245	0.0454	0.0000	-1.0355	0.0304
Rassf9	0.2338	0.9472	0.0779	0.0000	3.6844	0.0382
Rbm4b	0.0000	-0.8088	0.0227	0.0000	-1.4263	0.0090
Rbm5	0.0000	-0.3358	0.0431	0.0000	-1.0233	0.0099
Rc3h2	0.0000	-0.7068	0.0288	0.0000	-1.2125	0.0139
Rcn2	0.0000	-0.8412	0.0105	0.0000	-1.0079	0.0080
Rcor2	0.0000	-0.4426	0.0420	0.0000	-1.1204	0.0137
Reep3	0.0000	-0.6481	0.0283	0.0000	-1.2679	0.0102
Reep4	0.0023	0.4317	0.0548	0.0000	1.0518	0.0251
Reep6	0.5286	0.1182	0.0873	0.0000	1.3791	0.0246
Reln	0.0409	0.2471	0.0659	0.0000	-4.4298	0.0007
Rerg	0.1749	0.3567	0.0755	0.0000	1.2813	0.0386
Rfx3	0.7930	-0.0298	0.0946	0.0000	-1.1742	0.0163
Rgs12	0.0000	-0.5887	0.0303	0.0000	-1.0979	0.0129
Rgs5	0.2225	-0.4813	0.0775	0.0000	2.1966	0.0311
Rhbdd1	0.0000	0.7936	0.0302	0.0000	1.2171	0.0174
Rhbdf2	0.0002	0.6110	0.0486	0.0000	1.1517	0.0273
Rhobtb1	0.0000	-0.7766	0.0302	0.0000	-1.2305	0.0172
Rhpn1	0.0000	-0.7877	0.0443	0.0000	-1.6601	0.0211
Rimbp2	0.0001	-0.7045	0.0475	0.0000	-1.3758	0.0263
Rims2	0.0000	-0.9796	0.0183	0.0000	-1.1912	0.0141
Ripk2	0.0000	0.7058	0.0441	0.0000	1.2035	0.0264
Rmnd5a	0.0000	0.7243	0.0180	0.0000	1.0205	0.0101
Rnasel	0.0001	-0.5551	0.0457	0.0000	-1.5209	0.0148
Rnf128	0.0005	0.7780	0.0508	0.0000	1.8623	0.0210
Rnf130	0.0000	-0.8605	0.0143	0.0000	-1.1284	0.0092
Rnf180	0.0000	-0.9213	0.0419	0.0000	-1.0254	0.0389
Rnf181	0.0000	0.7947	0.0208	0.0000	1.3589	0.0079
Rnf217	0.0000	-0.9991	0.0293	0.0000	-1.2673	0.0229
Rnf219	0.0000	-0.6464	0.0343	0.0000	-1.0823	0.0191
Rnf38	0.0000	-0.9519	0.0118	0.0000	-1.8254	0.0026
Rnh1	0.0000	0.6562	0.0333	0.0000	1.1174	0.0173
Robo3	0.0000	-0.8437	0.0316	0.0000	-2.9230	0.0032
Rpgrip1l	0.4006	0.1054	0.0837	0.0000	-1.1460	0.0204
Rpl8	0.0000	0.6732	0.0173	0.0000	1.0197	0.0084
Rrad	0.0180	0.8109	0.0621	0.0000	1.9291	0.0336
Rras	0.1808	0.1954	0.0757	0.0000	1.0965	0.0244
Rrnad1	0.0000	-0.9010	0.0352	0.0000	-1.0455	0.0312
Rrs1	0.0001	0.3824	0.0457	0.0000	1.4669	0.0064
Rsbn1	0.0000	-0.9769	0.0129	0.0000	-1.1242	0.0108
Rsrp1	0.0116	0.2253	0.0603	0.0000	1.1820	0.0093
Rtn3	0.0000	-0.7090	0.0133	0.0000	-1.0655	0.0060

Rtn4r	0.0000	-0.9252	0.0434	0.0000	-2.0029	0.0202
Rundc3b	0.0000	-0.9998	0.0249	0.0000	-1.8139	0.0099
S100a10	0.0000	0.7487	0.0316	0.0000	1.6334	0.0094
Samd1	0.0000	0.4569	0.0418	0.0000	1.1919	0.0121
Samd12	0.0000	-0.7849	0.0374	0.0000	-1.2738	0.0228
Sat2	0.0825	0.4498	0.0696	0.0000	1.1113	0.0434
Satb1	0.0000	-0.9620	0.0189	0.0000	-1.5457	0.0084
Scai	0.0000	-0.6464	0.0378	0.0000	-1.1326	0.0208
Scara5	0.4545	0.1560	0.0852	0.0000	2.3408	0.0119
Scarb1	0.0008	0.3729	0.0518	0.0000	1.2680	0.0125
Scn1b	0.0003	-0.7947	0.0495	0.0000	2.1542	0.0134
Scn8a	0.0000	-0.8735	0.0312	0.0000	-1.2036	0.0221
Scpep1	0.0000	0.5195	0.0291	0.0000	1.7730	0.0022
Scrn1	0.0000	-0.3245	0.0441	0.0000	-1.2472	0.0061
Scrn2	0.0044	-0.4505	0.0569	0.0000	-1.0659	0.0294
Scrn3	0.0000	0.6146	0.0445	0.0000	1.1989	0.0222
Secisbp2l	0.0000	-0.6469	0.0235	0.0000	-1.1012	0.0101
Selenbp1	0.0823	0.4398	0.0696	0.0000	1.2110	0.0396
Sema4c	0.0000	-0.6295	0.0301	0.0000	-1.2350	0.0115
Sema6a	0.0000	-0.8595	0.0211	0.0000	-1.3115	0.0108
Sema7a	0.0116	-0.3983	0.0603	0.0000	-1.9205	0.0129
Serinc1	0.0000	-0.7614	0.0146	0.0000	-1.0415	0.0086
Serpinf1	0.0596	-0.2898	0.0678	0.0000	1.0708	0.0271
Setd4	0.0910	0.4298	0.0703	0.0000	1.0208	0.0457
Sfpq	0.4077	-0.0644	0.0838	0.0000	-1.1525	0.0075
Sfrp5	0.7553	0.2060	0.0937	0.0002	2.2712	0.0489
Sh3d19	0.0000	0.9206	0.0204	0.0000	1.4668	0.0090
Sh3gl3	0.0000	-0.8537	0.0329	0.0000	-1.3288	0.0202
Sh3kbp1	0.0000	-0.8262	0.0302	0.0000	-1.2082	0.0195
Sh3pxd2b	0.0000	-0.7505	0.0190	0.0000	-1.3519	0.0064
Shank2	0.0002	-0.6372	0.0480	0.0000	-1.0966	0.0306
Simc1	0.0000	-0.9103	0.0306	0.0000	-1.1440	0.0243
Sirt7	0.0021	-0.5576	0.0545	0.0000	-1.1622	0.0312
Siva1	0.0000	0.7977	0.0264	0.0000	1.1362	0.0164
Six4	0.0000	0.9258	0.0311	0.0000	1.8957	0.0104
Skida1	0.8129	-0.0275	0.0950	0.0000	-1.1918	0.0169
Skor1	0.0075	-0.7196	0.0587	0.0000	-2.8766	0.0178
Slc12a4	0.0000	0.4701	0.0446	0.0000	1.5073	0.0088
Slc15a2	0.6040	0.1345	0.0894	0.0000	-1.2667	0.0419
Slc16a1	0.0000	0.7237	0.0258	0.0000	1.5412	0.0067
Slc16a12	0.0008	-0.9334	0.0518	0.0000	-1.8210	0.0311
Slc16a13	0.0573	-0.3100	0.0676	0.0000	-1.0959	0.0298
Slc16a14	0.0001	0.6924	0.0455	0.0000	1.0919	0.0303
Slc16a7	0.9023	-0.0333	0.0973	0.0002	-1.0207	0.0498
Slc17a6	0.1236	-0.2304	0.0726	0.0000	-1.3217	0.0211
Slc20a1	0.0000	0.6970	0.0210	0.0000	1.4174	0.0051
Slc22a23	0.0000	-0.6734	0.0270	0.0000	-1.6403	0.0056
Slc25a12	0.0000	-0.5947	0.0329	0.0000	-1.0721	0.0159
Slc25a33	0.0210	0.3713	0.0628	0.0000	1.3873	0.0201
Slc25a35	0.0834	0.5960	0.0697	0.0001	1.3068	0.0475
Slc25a39	0.0000	0.8477	0.0175	0.0000	1.5407	0.0052
Slc25a40	0.0007	0.5922	0.0514	0.0000	1.0723	0.0312
Slc25a42	0.9637	0.0096	0.0989	0.0000	1.2318	0.0321
Slc27a4	0.0000	-0.7428	0.0221	0.0000	-1.1492	0.0113
Slc2a13	0.0089	-0.4942	0.0593	0.0000	-1.1931	0.0318
Slc35f1	0.0001	0.3934	0.0457	0.0000	-1.4637	0.0081
Slc38a4	0.0000	0.8396	0.0330	0.0000	1.7890	0.0110
Slc39a6	0.0000	-0.9463	0.0112	0.0000	-1.5782	0.0036
Slc44a5	0.0009	-0.4426	0.0520	0.0000	-2.6596	0.0042
Slc45a1	0.1814	-0.4060	0.0758	0.0002	-1.1442	0.0495
Slc45a4	0.0000	0.5139	0.0363	0.0000	1.1642	0.0118
Slc46a3	0.7913	-0.0790	0.0945	0.0002	1.0297	0.0499
Slc5a3	0.0000	-0.6419	0.0366	0.0000	-1.0651	0.0213
Slc8b1	0.0000	0.9656	0.0344	0.0000	1.4729	0.0212
Slco3a1	0.0000	-0.8633	0.0330	0.0000	-1.5046	0.0171
Slco4a1	0.0003	0.8698	0.0493	0.0000	1.7142	0.0263
Slx4ip	0.0002	-0.4807	0.0486	0.0000	-1.1928	0.0199
Smarcd2	0.0000	0.5278	0.0398	0.0000	1.1902	0.0143
Smpd3	0.0000	-0.4322	0.0429	0.0000	-1.9241	0.0041
Smtn	0.5844	-0.0679	0.0889	0.0000	1.1283	0.0183
Snhg3	0.4023	0.2685	0.0837	0.0000	1.4324	0.0415
Sntb2	0.0000	0.9147	0.0191	0.0000	1.0223	0.0171
Socs7	0.0000	-0.8750	0.0144	0.0000	-1.0368	0.0114
Soga1	0.0000	-0.8775	0.0138	0.0000	-1.3375	0.0061
Sorbs2	0.0970	0.1662	0.0709	0.0000	-1.2384	0.0120
Sorbs3	0.0186	0.4849	0.0622	0.0000	1.1246	0.0352
Sorcs3	0.3310	0.1601	0.0813	0.0000	-2.7996	0.0075

Sort1	0.0000	-0.4740	0.0291	0.0000	-1.1158	0.0071
Sox6	0.0003	-0.6479	0.0490	0.0000	-1.6836	0.0191
Spast	0.0000	-0.7445	0.0167	0.0000	-1.3082	0.0055
Spcs3	0.0000	0.7003	0.0180	0.0000	1.0706	0.0086
Spef1	0.3979	-0.1478	0.0836	0.0000	-1.5226	0.0222
Spock3	0.0007	-0.5118	0.0516	0.0000	-2.3880	0.0078
Spr	0.0000	0.7642	0.0278	0.0000	1.2557	0.0137
Sptssa	0.0000	-0.6624	0.0263	0.0000	-1.0002	0.0151
Srfbp1	0.0041	0.3191	0.0567	0.0000	1.0072	0.0188
Srgap1	0.0000	-0.5976	0.0307	0.0000	-1.5137	0.0069
Srxn1	0.3656	-0.1158	0.0825	0.0000	1.0383	0.0215
Ssbp3	0.0000	-0.9743	0.0078	0.0000	-1.2713	0.0046
Ssh2	0.0000	-0.8295	0.0214	0.0000	-1.6774	0.0059
Sspn	0.2210	-0.2685	0.0775	0.0000	2.0242	0.0170
St6gal1	0.0000	-0.4077	0.0449	0.0000	-1.0607	0.0152
St7	0.0000	-0.6235	0.0397	0.0000	-1.6252	0.0118
Stambpl1	0.2100	0.3491	0.0771	0.0001	1.0720	0.0471
Stard8	0.1946	-0.3445	0.0764	0.0000	1.5514	0.0309
Steap2	0.0003	0.5096	0.0491	0.0000	1.5335	0.0133
Stk26	0.0591	0.4056	0.0678	0.0000	1.6488	0.0232
Stmn1	0.3088	-0.0654	0.0806	0.0000	-1.2846	0.0032
Stxbp4	0.0000	-0.5839	0.0339	0.0000	-1.4137	0.0094
Susd1	0.1134	0.4652	0.0719	0.0001	1.1363	0.0470
Syne2	0.0000	-0.3901	0.0435	0.0000	-1.9348	0.0029
Syngr2	0.3293	-0.1458	0.0812	0.0000	1.2844	0.0195
Synj1	0.0000	-0.7017	0.0267	0.0000	-1.3936	0.0089
Synrg	0.0000	-0.6199	0.0268	0.0000	-1.3501	0.0072
Syt13	0.0111	-0.4396	0.0601	0.0000	-1.0832	0.0320
Syt17	0.0000	-0.9044	0.0433	0.0000	-1.0539	0.0389
Tango2	0.0001	-0.6397	0.0468	0.0000	-1.0562	0.0309
Taok1	0.0000	-0.3879	0.0405	0.0000	-1.0155	0.0117
Taok2	0.0000	-0.6125	0.0239	0.0000	-1.0038	0.0114
Tbc1d8	0.0000	0.8349	0.0388	0.0000	1.3092	0.0246
Tbc1d9	0.0000	-0.8091	0.0174	0.0000	-1.4331	0.0058
Tcerg1l	0.0005	-0.8513	0.0507	0.0000	-2.0160	0.0241
Tcf12	0.0000	-0.5616	0.0276	0.0000	-1.1679	0.0085
Tcf7l1	0.0000	0.4958	0.0414	0.0000	1.3646	0.0103
Tcirg1	0.0079	0.5043	0.0589	0.0000	1.7029	0.0186
Tdrkh	0.0000	-0.4374	0.0443	0.0000	-1.6815	0.0064
Tek	0.0025	0.9477	0.0551	0.0000	1.3114	0.0451
Tenm2	0.0000	-0.5209	0.0420	0.0000	-2.0122	0.0056
Tfap4	0.0000	0.5814	0.0443	0.0000	1.1192	0.0225
Tfrc	0.9766	-0.0021	0.0994	0.0000	-1.4106	0.0037
Tgfb1	0.5723	0.1362	0.0886	0.0000	1.6965	0.0254
Tgfbr1	0.0000	-0.7212	0.0179	0.0000	-1.5783	0.0033
Thap2	0.0000	-0.9550	0.0198	0.0000	-1.1687	0.0153
Thap4	0.0000	0.6704	0.0287	0.0000	1.1672	0.0128
Thbs2	0.0029	-0.9390	0.0556	0.0000	1.3668	0.0442
Them6	0.0056	0.6641	0.0578	0.0000	2.0717	0.0194
Thsd7b	0.1709	-0.4647	0.0753	0.0000	-1.8695	0.0379
Thy1	0.1730	-0.3584	0.0754	0.0000	2.8154	0.0107
Tia1	0.0000	-0.6023	0.0244	0.0000	-1.2854	0.0065
Tie1	0.8172	0.1306	0.0951	0.0000	2.1582	0.0457
Tigd3	0.0020	-0.8698	0.0544	0.0000	-1.7068	0.0335
Tinf2	0.0000	0.6275	0.0374	0.0000	1.0319	0.0218
Tle6	0.0340	0.9116	0.0650	0.0001	1.6752	0.0467
Tlr2	0.1182	-0.4423	0.0723	0.0000	1.5360	0.0333
Tm7sf3	0.0088	-0.2303	0.0592	0.0000	1.2976	0.0067
Tmem107	0.0003	0.5811	0.0489	0.0000	1.0651	0.0285
Tmem108	0.3433	-0.1745	0.0817	0.0000	-1.4193	0.0260
Tmem117	0.6456	0.1230	0.0906	0.0000	1.7257	0.0283
Tmem132l	0.6479	-0.0851	0.0906	0.0000	-1.2062	0.0314
Tmem132c	0.0569	0.2673	0.0675	0.0000	-1.0586	0.0259
Tmem150c	0.0541	-0.4522	0.0672	0.0000	-1.6165	0.0294
Tmem176a	0.0000	0.6627	0.0426	0.0000	1.5321	0.0155
Tmem200a	0.5664	-0.0833	0.0884	0.0000	-1.0244	0.0283
Tmem243	0.0715	0.4147	0.0688	0.0000	1.2393	0.0354
Tmem255a	0.4634	0.2798	0.0855	0.0000	-2.6091	0.0296
Tmem33	0.0000	-0.4413	0.0338	0.0000	-1.0309	0.0099
Tmem39a	0.0000	0.5327	0.0351	0.0000	1.1768	0.0114
Tmem62	0.0028	-0.9159	0.0555	0.0000	-2.0280	0.0312
Tmem74	0.0654	-0.6286	0.0683	0.0000	-1.7109	0.0405
Tmub1	0.0003	-0.6562	0.0488	0.0000	-1.1466	0.0311
Tmub2	0.0000	-0.9229	0.0186	0.0000	-1.0471	0.0162
Tmx4	0.0000	-0.6021	0.0241	0.0000	-1.3087	0.0060
Tnfaip2	0.9706	0.0108	0.0992	0.0000	2.0724	0.0246
Tnfrsf10b	0.0074	0.3114	0.0587	0.0000	1.4351	0.0102

Tnik	0.1866	-0.1404	0.0761	0.0000	-1.4238	0.0098
Tnrc6b	0.0000	-0.4517	0.0359	0.0000	-1.0114	0.0123
Tomm40	0.0000	0.8646	0.0154	0.0000	1.0246	0.0124
Top2b	0.0000	-0.5929	0.0188	0.0000	-1.6526	0.0016
Tox3	0.0000	-0.7320	0.0278	0.0000	-1.3774	0.0108
Tpgs2	0.0000	-0.6423	0.0325	0.0000	-1.0087	0.0194
Tph2	0.8120	0.0749	0.0950	0.0000	1.2783	0.0446
Trim13	0.0000	0.7167	0.0344	0.0000	1.4437	0.0132
Trim2	0.0000	-0.6729	0.0204	0.0000	-1.8632	0.0021
Trim7	0.0457	0.5632	0.0664	0.0000	1.5279	0.0352
Trrap	0.0029	-0.2156	0.0555	0.0000	-1.1410	0.0065
Tspan18	0.0000	-0.8595	0.0246	0.0000	-1.5127	0.0103
Tspan3	0.0000	-0.7628	0.0129	0.0000	-1.0796	0.0068
Ttc39c	0.0000	-0.9767	0.0318	0.0000	-1.2550	0.0248
Ttf2	0.0000	0.9941	0.0209	0.0000	1.0579	0.0204
Ttll1	0.0000	-0.9494	0.0223	0.0000	-1.4373	0.0121
Ttyh3	0.0000	-0.8840	0.0085	0.0000	-1.2197	0.0042
Tubb3	0.0000	-0.6650	0.0161	0.0000	-2.3097	0.0003
Tubb6	0.0000	0.4639	0.0367	0.0000	1.1764	0.0094
Tulp3	0.0000	0.5396	0.0370	0.0000	1.1337	0.0142
Twf2	0.0000	0.8249	0.0242	0.0000	1.0986	0.0168
Ube2d1	0.0000	-0.6896	0.0243	0.0000	-1.4640	0.0067
Ube2e3	0.0000	-0.3603	0.0434	0.0000	-1.0703	0.0106
Ube4a	0.0003	-0.3527	0.0495	0.0000	-1.0373	0.0155
Uchl1	0.0000	-0.8148	0.0128	0.0000	-1.4391	0.0037
Unc13a	0.0000	-0.8437	0.0192	0.0000	-1.2039	0.0109
Unc80	0.4440	0.1818	0.0849	0.0000	-1.0883	0.0437
Uros	0.0445	-0.3707	0.0662	0.0000	-1.2633	0.0294
Usp20	0.0000	-0.6479	0.0360	0.0000	-1.2063	0.0175
Usp22	0.0000	-0.7734	0.0134	0.0000	-1.0347	0.0081
Usp3	0.0000	-0.7926	0.0235	0.0000	-1.2899	0.0112
Usp44	0.0776	0.3935	0.0693	0.0000	1.4495	0.0290
Usp47	0.0000	-0.4504	0.0298	0.0000	-1.0048	0.0084
Vapb	0.0000	-0.8972	0.0138	0.0000	-1.0118	0.0122
Vasp	0.0000	0.8424	0.0146	0.0000	1.2263	0.0072
Vav2	0.0000	-0.7836	0.0208	0.0000	-1.2564	0.0096
Vezf1	0.0000	-0.9681	0.0109	0.0000	-1.3836	0.0054
Vgll3	0.1283	-0.2700	0.0729	0.0000	-1.1119	0.0323
Vldlr	0.0000	-0.5868	0.0353	0.0000	-1.1209	0.0161
Vps37b	0.0000	-0.9961	0.0197	0.0000	-1.2205	0.0152
Vps53	0.0001	-0.4298	0.0460	0.0000	-1.0618	0.0176
Vta1	0.0000	-0.7336	0.0205	0.0000	-1.0709	0.0114
Vwa5b2	0.1013	-0.6569	0.0711	0.0000	-2.0255	0.0410
Wasf1	0.0000	-0.4241	0.0418	0.0000	-1.0698	0.0136
Wasf2	0.0000	0.4784	0.0356	0.0000	1.2137	0.0089
Wdfy3	0.0000	-0.6072	0.0229	0.0000	-1.5982	0.0031
Wdpcp	0.1812	0.3567	0.0758	0.0002	-1.0500	0.0489
Wdr17	0.9146	0.0317	0.0975	0.0001	-1.1893	0.0481
Wdr37	0.0000	-0.8136	0.0157	0.0000	-1.2676	0.0069
Wdr7	0.0000	-0.9263	0.0158	0.0000	-1.5660	0.0057
Wnk3	0.0000	-0.8142	0.0241	0.0000	-1.5427	0.0085
Wsb1	0.0000	-0.7360	0.0156	0.0000	-1.5289	0.0030
Wscd1	0.0018	-0.3500	0.0541	0.0000	-1.1339	0.0168
Wwc2	0.0000	0.9148	0.0121	0.0000	1.1619	0.0081
Xkr6	0.0017	-0.5789	0.0540	0.0000	-1.3133	0.0277
Xpr1	0.0000	-0.4035	0.0391	0.0000	-1.6323	0.0035
Ypel1	0.0000	-0.6049	0.0357	0.0000	-1.7214	0.0077
Zbtb18	0.0022	-0.3184	0.0546	0.0000	-1.0166	0.0177
Zbtb38	0.0002	0.4801	0.0483	0.0000	1.1230	0.0202
Zbtb39	0.0000	-0.6096	0.0367	0.0000	-1.0402	0.0206
Zbtb5	0.0000	-0.8957	0.0157	0.0000	-1.4471	0.0064
Zbtb8a	0.0016	0.5329	0.0539	0.0000	1.2298	0.0256
Zc3h12c	0.0000	-0.8125	0.0343	0.0000	-1.7120	0.0131
Zcchc18	0.0009	-0.3381	0.0521	0.0000	-1.1387	0.0140
Zcchc2	0.0000	0.7034	0.0300	0.0000	1.0501	0.0183
Zdbf2	0.0184	-0.1909	0.0622	0.0000	-1.0359	0.0104
Zdhhc17	0.0000	-0.5139	0.0378	0.0000	-1.2372	0.0121
Zdhhc9	0.0001	-0.4493	0.0462	0.0000	-1.0241	0.0201
Zeb2	0.0000	-0.6897	0.0266	0.0000	-1.3178	0.0095
Zfhx2	0.0000	-0.7668	0.0197	0.0000	-1.1641	0.0099
Zfhx4	0.0000	-0.8614	0.0190	0.0000	-3.2228	0.0005
Zfp2	0.0000	-0.9503	0.0270	0.0000	-1.1462	0.0220
Zfp37	0.0000	-0.9924	0.0198	0.0000	-1.1516	0.0167
Zfp64	0.0000	0.5741	0.0341	0.0000	1.0965	0.0146
Zik1	0.0000	-0.6872	0.0305	0.0000	-1.5243	0.0092
Zkscan1	0.0000	-0.6902	0.0249	0.0000	-1.5644	0.0058
Zkscan3	0.0000	-0.6799	0.0243	0.0000	-1.0635	0.0129

Znrf1	0.0000	-0.9025	0.0171	0.0000	-1.1307	0.0124
Zscan21	0.0000	-0.6232	0.0256	0.0000	-1.1609	0.0095
Zswim4	0.0000	-0.4633	0.0375	0.0000	-1.0352	0.0137
Zswim8	0.0000	-0.9084	0.0122	0.0000	-1.1267	0.0088
Zyg11b	0.0000	-0.8421	0.0142	0.0000	-1.2355	0.0071
Zzef1	0.0051	-0.3140	0.0575	0.0000	-1.0884	0.0180
