# Supplementary material for: DNMT1 downregulation as well as its overexpression distinctly affect mostly overlapping genes implicated in schizophrenia, autism spectrum, epilepsy, and bipolar disorders
Source: Front Mol Neurosci. 2023 Dec 6;16:1275697. doi: 10.3389/fnmol.2023.1275697 (PMC10731955; doi:10.3389/fnmol.2023.1275697)
Supplement: Supplementary file 2 [file Data_Sheet_2.pdf]

Supplementary figure 1. Raw western blot images for Figure 1B. Yellow line indicates the lanes used for the figure 1B with corresponding molecular weights given in right. Minus and plus represents untreated and doxycycline treated *Tet/Tet* neurons, respectively.

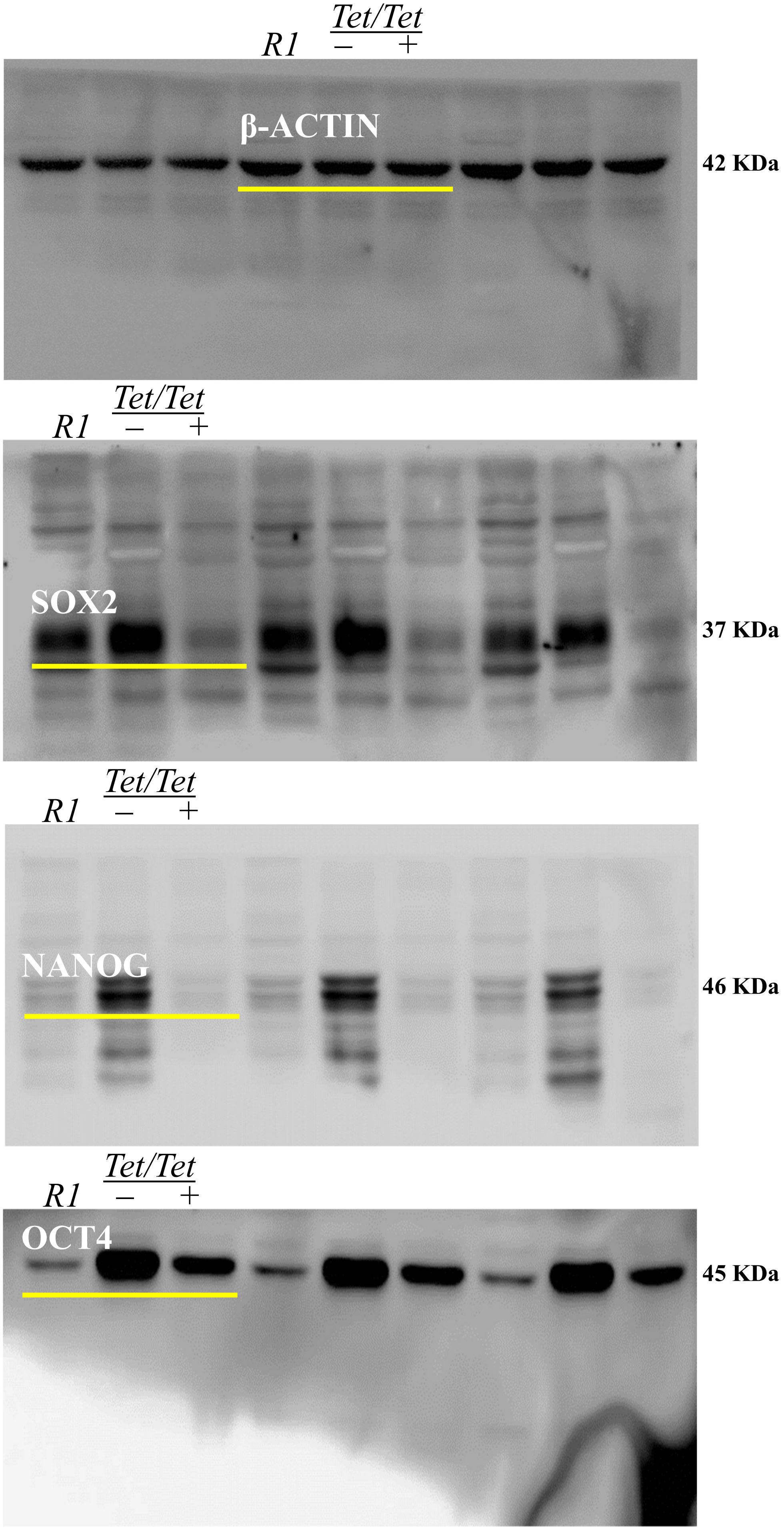

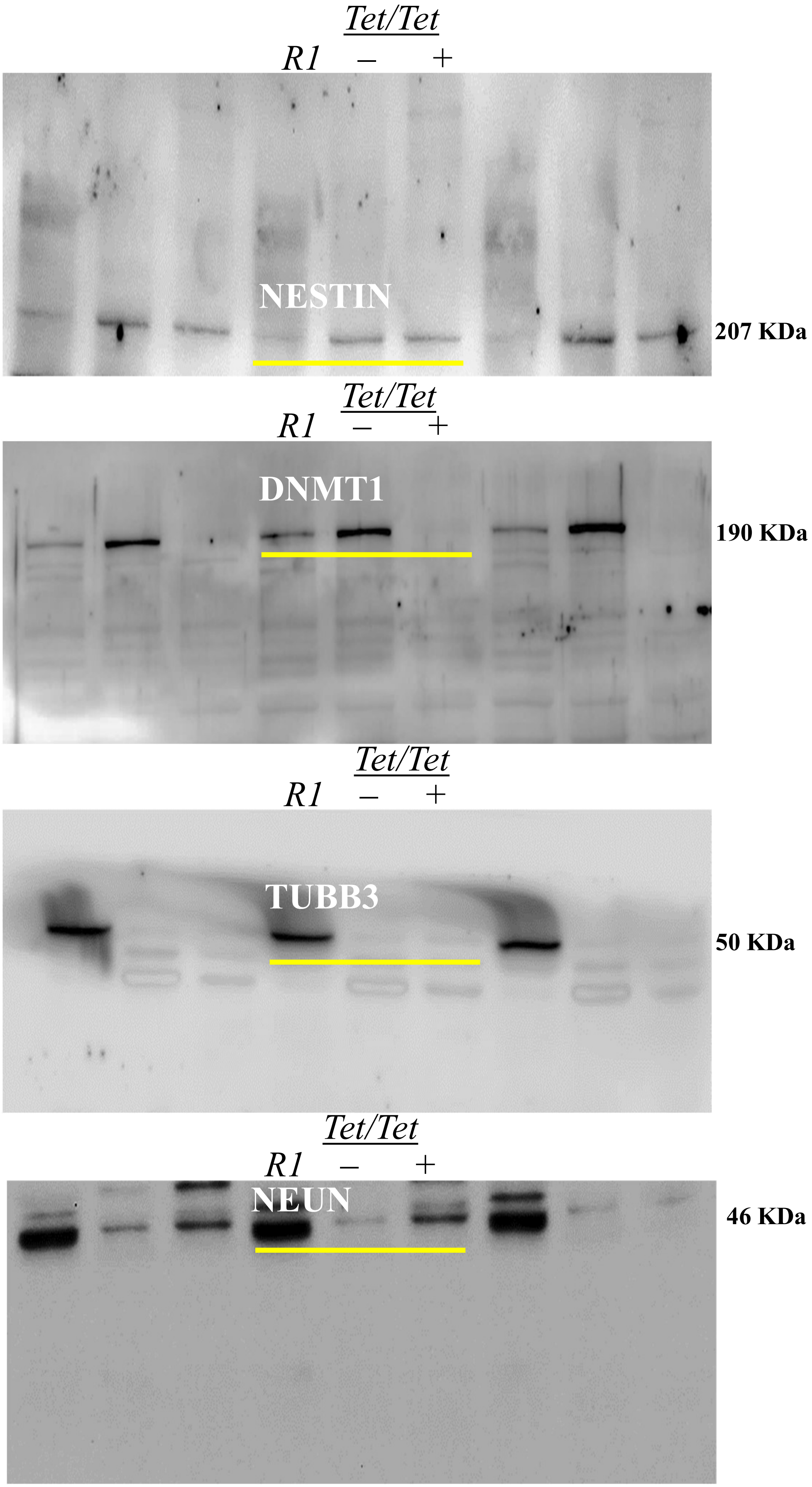

Tet/Tet
R1 – +
NESTIN
207 KDa
Tet/Tet
R1 – +
DNMT1
190 KDa
Tet/Tet
R1 – +
TUBB3
50 KDa
Tet/Tet
R1 – +
NEUN
46 KDa

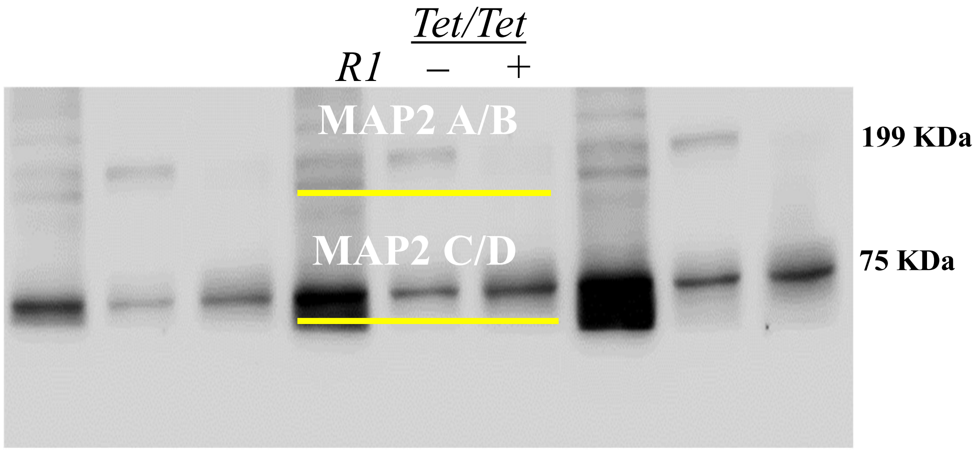
Tet/Tet
R1 – +
MAP2 A/B
MAP2 C/D
199 KDa
75 KDa
